# Senescent Schwann cells induced by aging and chronic denervation impair axonal regeneration following peripheral nerve injury

**DOI:** 10.15252/emmm.202317907

**Published:** 2023-10-20

**Authors:** Andrés Fuentes‐Flores, Cristian Geronimo‐Olvera, Karina Girardi, David Necuñir‐Ibarra, Sandip Kumar Patel, Joanna Bons, Megan C Wright, Daniel Geschwind, Ahmet Hoke, Jose A Gomez‐Sanchez, Birgit Schilling, Daniela L Rebolledo, Judith Campisi, Felipe A Court

**Affiliations:** ^1^ Center for Integrative Biology, Faculty of Sciences Universidad Mayor Santiago Chile; ^2^ Geroscience Center for Brain Health and Metabolism (GERO) Santiago Chile; ^3^ Buck Institute for Research on Aging Novato CA USA; ^4^ Departments of Neurology and Neuroscience Johns Hopkins School of Medicine Baltimore MD USA; ^5^ Departments of Neurology, Psychiatry, and Human Genetics, David Geffen School of Medicine University of California Los Angeles Los Angeles CA USA; ^6^ Instituto de Investigación Sanitaria y Biomédica de Alicante (ISABIAL) Alicante Spain; ^7^ Instituto de Neurociencias de Alicante UMH‐CSIC San Juan de Alicante Spain; ^8^ Present address: Arcadia University Philadelphia PA USA

**Keywords:** aging, chronic denervation, nerve regeneration, Schwann cell, senescence, Neuroscience

## Abstract

Following peripheral nerve injury, successful axonal growth and functional recovery require Schwann cell (SC) reprogramming into a reparative phenotype, a process dependent upon c‐Jun transcription factor activation. Unfortunately, axonal regeneration is greatly impaired in aged organisms and following chronic denervation, which can lead to poor clinical outcomes. While diminished c‐Jun expression in SCs has been associated with regenerative failure, it is unclear whether the inability to maintain a repair state is associated with the transition into an axonal growth inhibition phenotype. We here find that reparative SCs transition into a senescent phenotype, characterized by diminished c‐Jun expression and secretion of inhibitory factors for axonal regeneration in aging and chronic denervation. In both conditions, the elimination of senescent SCs by systemic senolytic drug treatment or genetic targeting improved nerve regeneration and functional recovery, increased c‐Jun expression and decreased nerve inflammation. This work provides the first characterization of senescent SCs and their influence on axonal regeneration in aging and chronic denervation, opening new avenues for enhancing regeneration and functional recovery after peripheral nerve injuries.

## Graphical abstract

Following nerve damage of aged nerves or delayed repair, Schwann cells (SCs) are less able to transition into the reparative phenotype necessary for axonal regrowth. Here, SCs from chronically denervated or aged conditions are characterized to better understand their influence on regeneration.

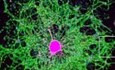

**The paper explained**

**Problem**

Despite the robust regenerative capacity of the peripheral nervous system, nerve injuries remain a major cause of clinical morbidity, particularly for older patients and those with chronic denervation. Less than half of nerve injury patients will recover useful function, and up to one‐third will experience little or no recovery even after surgical intervention. After peripheral nerve injuries, successful axonal growth and functional recovery require that Schwann cells, glial cells which surround and support neurons, transition into a reparative phenotype. However, the characteristics and influence of aged and chronically denervated Schwann cells that fail to elicit regeneration are not well defined.

**Results**

Repair Schwann cells transition into a senescent phenotype, a cellular state induced in response to various stressors, characterized by irreversible cell cycle arrest and resistance to programed cell death. In this senescent state, Schwann cells express high inflammation markers, diminished c‐Jun expression, and secrete several factors inhibitory for axonal regeneration in aged and chronic denervation contexts. In both conditions, senescent Schwann cell elimination, by systemic senolytic drug treatment or genetic targeting, improves nerve regeneration and functional recovery, associated with an upregulation of c‐Jun expression and decreased nerve inflammation.

**Impact**

This work provides the first characterization of senescent Schwann cells and their impact on axonal regeneration in aging and chronic denervation. Elimination of senescent cells to increase axon regeneration and functional recovery during aging and in conditions of chronic denervation will provide new avenues for clinical applications.

## Introduction

The peripheral nervous system (PNS) exhibits an effective regenerative capacity after nerve injury due to a coordinated tissue response. After nerve damage, injured neurons activate an intrinsic growth response and extend over a permissive regenerative environment generated by Schwann cells (SC), the glial cell component of the PNS. This response to nerve damage is critically dependent on the capacity of SC to reprogram into a repair phenotype (rSC) which supports axonal regeneration. SC transition into rSC is triggered by the injury‐induced degeneration of their associated axons and depends on the activation of the transcription factor c‐Jun (Arthur‐Farraj *et al*, 2012; Jessen & Mirsky, 2016). SC reprogramming is characterized by cell proliferation, myelin phagocytosis, and the secretion of pro‐regenerative factors, including exosomes (Arthur‐Farraj *et al*, 2012; Lopez‐Verrilli *et al*, 2013; Gomez‐Sanchez *et al*, 2015). In addition, rSC recruit macrophages and generate cell tracks that guide regenerating axons (Gomez‐Sanchez *et al*, 2017).

Despite the good regenerative capacity of the PNS, especially in laboratory setups, nerve injuries remain a major cause of morbidity in the clinic (Kallio & Vastamäki, 1993; Bergmeister *et al*, 2020; Lanier *et al*, 2021). Less than half of the patients suffering nerve injuries will recover useful function, and up to one‐third will experience little or no recovery after appropriate surgical intervention (Kallio & Vastamäki, 1993; Noble *et al*, 1998; Lanier *et al*, 2021). Importantly, previous work has established that age and chronic SC denervation are the major factors affecting peripheral regeneration in laboratory models and human patients (Sulaiman & Gordon, 2000; Verdú *et al*, 2000; Painter *et al*, 2014; Ronchi *et al*, 2017). Age‐dependent regeneration deficits have been associated with enhanced inflammatory response in the nerve and decreased capacity of SC to maintain their reparative phenotype (Scheib & Höke, 2016b). Interestingly, this age‐dependent decline in regeneration is not a consequence of a reduced growth capacity of regenerating neurons (Verdú *et al*, 2000; Painter *et al*, 2014; Scheib & Höke, 2016a; Büttner *et al*, 2018). In the case of chronic denervation, associated to damaged nerves that are not reconnected for several weeks, months, or years, it has been reported that rSC gradually lose their ability to secrete pro‐regenerative trophic factors such as BDNF or GDNF (Sulaiman & Gordon, 2009; Ronchi *et al*, 2017). Importantly, chronic SC denervation arises not only as a consequence of delayed surgical repair, but also as a result of axons regenerating for months or even years in long human nerves (Sulaiman & Gordon, 2009; Ronchi *et al*, 2017; Jessen & Mirsky, 2019). It has been recently demonstrated that aged and chronically denervated rSC exhibit diminished c‐Jun expression after injury, and forced upregulation of this transcription factor restores axonal regeneration in these two conditions (Wagstaff *et al*, 2021). Even though high c‐Jun expression is sufficient to activate axonal regeneration, the phenotype acquired by low‐c‐Jun expressing SC has not been clearly defined, nor a possible inhibitory effect they might have over axonal regeneration. Interestingly, it has been shown that SC invading long acellularized nerve allografts express markers of senescent cells (Saheb‐Al‐Zamani *et al*, 2013; Poppler *et al*, 2016). This raises the question of whether SC undergo senescence in the context of aging and chronic denervation, and if this senescent SC phenotype is associated with downregulation of c‐Jun expression and the production of inhibitory factor(s) for axonal regeneration and functional recovery.

Senescence is a cellular state induced in response to various stressors, such as irreparable DNA damage, oxidative stress and oncogenic activation, and characterized by an irreversible cell cycle arrest and resistance to apoptosis (Rodier & Campisi, 2011; Muñoz‐Espín & Serrano, 2014; Gorgoulis *et al*, 2019). Senescent cells show chromatin instability, increased cellular size, enhanced lysosomal activity, abnormalities in nuclear morphology, and a specific senescence‐associated secretory phenotype (SASP) composed of pro‐inflammatory angiogenic and extracellular matrix‐degrading factors (Itahana *et al*, 2007; Rodier & Campisi, 2011; Morgunova *et al*, 2015). It has been shown that senescent cells can have both, beneficial and detrimental roles in tissue repair, depending if the exposure to the stressing stimuli is transient or chronic (Rhinn *et al*, 2019; Wilkinson & Hardman, 2020). After acute tissue damage, senescent cells are necessary to inhibit the proliferation of damaged cells and prevent tumor formation through the wound healing process, after which they are cleared by macrophages (Demaria *et al*, 2014; Rhinn *et al*, 2019). In contrast, long‐lasting exposure to stressor agents or aging generates a chronic accumulation of senescent cells. Chronic senescent cells are inefficiently cleared, contributing to abnormal SASP accumulation in the tissue. This affects neighboring cells in a paracrine fashion, leading to tissue dysfunction or age‐related pathologies (Muñoz‐Espín & Serrano, 2014; Childs *et al*, 2015; Schafer *et al*, 2017; Rhinn *et al*, 2019). In this regard, chronically denervated SC stop proliferating through cell cycle arrest and also show reduced expression of essential pro‐regenerative factors such as BDNF, GDNF, and NRG1 (Gomez‐Sanchez *et al*, 2013), with increased secretion of proinflammatory cytokines (Gordon, 2009; Ronchi *et al*, 2017; Jessen & Mirsky, 2019).

Here, we characterized senescent Schwann cells (sSC) and their role in axonal regeneration after peripheral nerve injury. We found that c‐Jun‐negative SC accumulate after damage in aged and chronically denervated nerves exhibiting *bona fide* cell senescence markers. In contrast to the pro‐regenerative effect of rSC secreted factors, the senescent SC‐associated secretory component strongly inhibits the axonal growth of sensory neurons. Importantly, we found that systemic treatment with a senolytic eliminates sSC and enhances axonal regeneration in aged and chronically denervated animals. Furthermore, the elimination of sSC downregulates pro‐inflammatory factors present in injured nerves from aged animals and after chronic denervation, restoring c‐Jun levels in the SC present in these nerves. Taken together, our data demonstrate that sSC have an inhibitory effect over axonal regeneration in aging and chronic denervation. Therefore, eliminating sSC, or neutralizing their inhibitory secreted factors, stands out as a novel opportunity for therapeutic intervention to elimination enhance axonal regeneration and functional recovery after peripheral injuries.

## Results

### Senescent Schwann cells accumulate in peripheral nerves after chronic denervation and in aged animals

**Figure 1:**
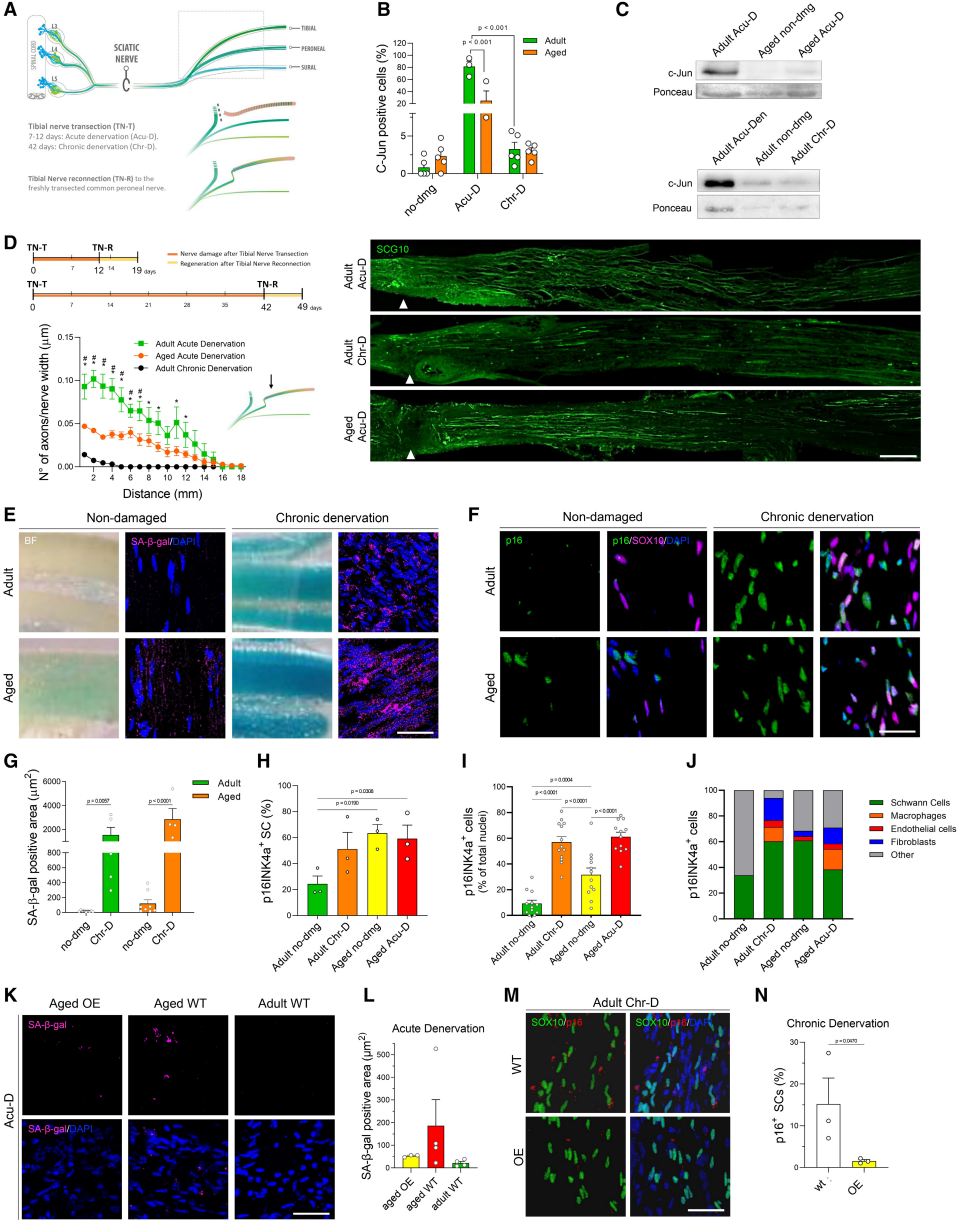
Aged and chronically denervated sciatic nerves show reduced regeneration and higher senescent cell accumulation ASchematic representation of the sciatic nerve transection model. The sciatic nerve divides into three branches, the tibial, peroneal and sural nerves (upper panel). The tibial branch is first transected (Tibial Nerve Transection, TN‐T) and the distal stump is sutured to the nearest muscle to prevent reconnection (middle panel). The time of denervation until analysis of the tissue defines acute (Acu‐D) or chronic (Chr‐D) denervation. To evaluate regeneration, the distal tibial nerve is detached from the muscle and reconnected to the freshly transected common peroneal nerve (Tibial Nerve reconnection, TN‐R, bottom panel).Bc‐Jun expression in the nucleus of SC on longitudinal cryostat sections of adult and aged mice sciatic nerves. We compared non‐damaged nerves, and sciatic nerves distal to the nerve cut in acute (7 dpi) and chronic (42 dpi) denervation conditions. *N* = 3–5 animals per group. One‐way ANOVA and Fisher's LSD multicomparison post‐test.CRepresentative western blot against c‐Jun comparing Acu‐D in adult mice (*N* = 5) to Acu‐D in aged mice (*N* = 4) and to Chr‐D in adult mice (*N* = 3).DThe scheme in the upper left show the timeline for transection and reconnection surgeries to evaluate axonal regeneration. Comparison of axonal density and distance 7 days after reconnection surgery in aged (*N* = 4) and adult mice with Acu‐D (*N* = 5), or adult mice with Chr‐D (*N* = 3). Multiple *t*‐test for each distance point (*x*‐axis) was performed, comparing the difference among Acu‐D in adult versus aged mice (#), and adult mice with Acu‐D versus Chr‐D (*). Significative differences are shown with symbols, and the exact *P*‐values can be seen in Appendix Table S1. Data is presented as mean ± SEM. Representative IF images of reconnected sciatic nerves are shown to the right using SCG10 marker in green. Arrowheads indicate the reconnection site (see Materials and Methods for details). The dataset used for this panel (D) corresponds to control conditions of the experiment shown in Fig 5B–D. Scale bar, 500 μm.E, GBrightfield and fluorescence confocal acquisition of β‐galactosidase activity in adult and aged animals, measured on longitudinal sections from non‐damaged and Chr‐D damaged nerves, distal to the injury. Scale bar, 42 μm. *N* = 5 animals per group. Complete nerves in brightfield can be seen in Fig EV1.F, HImmunofluorescence against p16INK4a and SOX10 in contralateral non‐injured nerves and chronically transected sciatic nerves from adult and aged mice. Scale bar, 50 μM. Quantification of p16INK4a‐positive SC correspond to *N* = 3 mice per condition. One‐way ANOVA and Fisher's LSD multicomparison post‐test.IQuantification of total p16INK4a‐positive cells in contralateral non injured nerves and damaged nerves from adult animals (*N* = 3 mice per condition with 4 micrographs per animal each).JProportion of different p16INK4a‐positive cell types in uninjured and injured nerves from adult and aged mice (*N* = 3). The total 100% percent of each condition corresponds to total p16INK4a^+^ cells quantified in (I). Detailed quantification and immunofluorescence for each cell type can be seen in Fig EV2.K, LFluorescence confocal acquisition and quantification of β‐galactosidase activity assay on longitudinal sections of injured sciatic nerves, distal to the nerve cut, after acute denervation in adult wild type and c‐Jun OE animals. *N* = 3 or 4 mice. Scale bar, 40 μM.M, NImmunofluorescent staining against p16INK4a in distal sciatic nerves after chronic denervation in adult wild type and c‐Jun OE animals. Scale bar, 40 μM. Quantification of p16INK4a‐positice Schwann cells shown in (M) are quantified in (N). *N* = 3 animals per group. Data information: For all (A–N), when two groups were compared, a non‐paired, one‐tailed Student's *t*‐test was performed; when more than two groups were compared, One‐way ANOVA and Fisher's LSD multicomparison post‐test. Data is presented as mean ± SEM. Source data are available online for this figure.

**Figure EV1:**
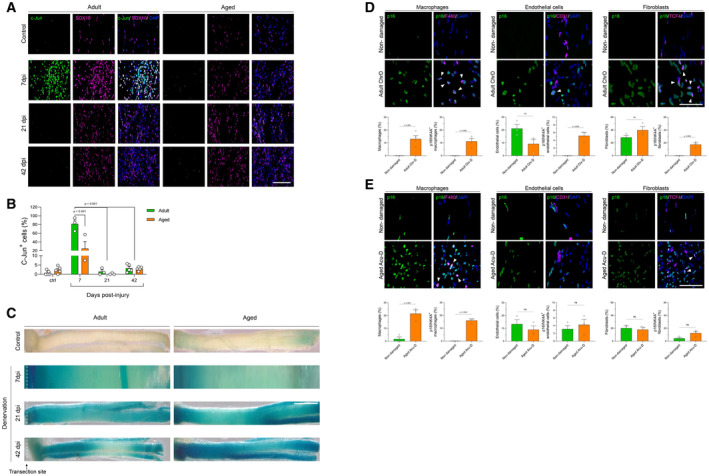
Sciatic nerves from chronically denervated and aged animals have diminished c‐Jun expression and axonal regeneration in SCs after injury compared to adult animals, with changes in macrophages, endothelial cells and fibroblasts A, BRepresentative IF confocal images (SOX10, magenta; c‐Jun, red; DAPI, blue) on longitudinal cryostat sections of adult and aged mice sciatic nerves 7, 21 and 42 dpi. The quantification graph shows the % of c‐Jun positive nuclei of SCs Scale bar, 100 μM.CRepresentative brightfield images of β‐galactosidase activity on non‐injured nerves, and transected nerves at different times points after damage. Scale bar, 1,000 μM.D, ERepresentative IF for p16^+^ (cell senescence marker), F480^+^ (macrophages), CD31^+^ (endothelial cells), and TCF‐4^+^ (fibroblasts) cells in longitudinal sections of nerves from chronically denervated in adult mice (D) or acutely denervated in aged mice (E). For each marker, the quantification shows in the left bar the percentage of the specific cell type among total nuclei in the nerve, and the right bar represents the percentage of the specific cell type among the total number of p16‐positive senescent cells. This data was used to generate the quantifications in Fig 1J. Source data are available online for this figure.

As up‐regulation of c‐Jun in rSC after damage is essential for effective axonal regeneration, we first evaluated the dynamics of c‐Jun expression in SC after sciatic nerve transection (Fig 1A) under two conditions in which regeneration is impaired, aging and chronic denervation. Consistent with previous studies (Wagstaff *et al*, 2021), in adult mice (2–4 months old), we detected a significant increase in the expression of c‐Jun in SC distal to the sciatic nerve transection zone (i.e., denervated SC) after acute denervation, 7 days post‐injury (dpi), when compared to undamaged control nerves. By contrast, in aged animals (20–22 months old), SC denervated for 7 days fail to up‐regulate c‐Jun to levels comparable to adult denervated SC (Figs 1B and C, and EV1A and B). Furthermore, after chronic SC denervation (i.e., SC distal to the sciatic nerve cut at 21 and 42 dpi), c‐Jun levels drop dramatically in both aged and adult nerves (Figs 1B and EV1A and B).

In order to study axonal regeneration, we used a model of nerve repair in which the tibial branch is first transected (Tibial Nerve Transection, TN‐T) and the distal stump is sutured to the nearest muscle to prevent reconnection (Fig 1A). Then, at 12 dpi (acute denervation, Acu‐D) or 42 dpi (chronic denervation, Chr‐D), the distal tibial nerve is detached from the muscle and reconnected to the freshly transected common peroneal nerve (Tibial Nerve reconnection, TN‐R). Axonal regeneration was evaluated 7 days post‐reconnection in our setting (Fig 1A and D). This model (Wagstaff *et al*, 2021) allows one to perform denervation for desired periods (acute or chronic denervation) and then be able to connect the distal (denervated stump) to the proximal one without the need for a nerve bridge. In addition, as neurons projecting to the peroneal branch are damaged at the same time in all groups, possible influences of chronic neuronal damage are eliminated (Fig 1A). As expected, we observed that in both, chronic denervation in adult mice and acute denervation in aged mice, axonal regeneration was strongly inhibited compared to adult animals reconnected after acute denervation (Fig 1D).

As chronic proliferative phenotypes and aging have been associated with senescent cell accumulation (Childs *et al*, 2015), we assessed if the impairment in axonal regeneration in aging and chronic denervation correlated with the accumulation of senescent cells in these conditions. To this end, we tested a battery of senescence markers in chronically denervated nerves from adult or aged mice. SA‐β‐galactosidase (β‐gal) expression, a canonical senescent‐cell marker, was increased in uninjured aged nerves compared to adult animals (Fig 1E and F). When β‐gal was evaluated in the distal nerve stump after acute denervation, adult and aged animals showed equivalent β‐gal activity (Fig EV1C), which increased in the distal nerve stump after chronic denervation (Figs 1E and G, and EV1C). As senescence characterization requires the combination of several markers (Roy *et al*, 2020), we measured the expression and localization of the cyclin‐dependent kinase (CDK) inhibitor p16INK4a (cdkn2a), critical for the cell cycle arrest and senescence development (Hernandez‐Segura *et al*, 2018) on SOX10‐positive SC. We observed that SC positive for p16INK4a were significantly increased in chronically denervated nerves of both adult and aged animals, reaching above of 50% of all resident SC (Fig 1F and H). Furthermore, total p16INK4a‐positive cells are increased in injured nerves compared to contralateral ones (Fig 1I), and the total protein levels of p16INK4a were increased after chronic denervation (Fig 4J). Importantly, the percentage of total p16INK4a‐positive cells (Fig 1I), and p16INK4a‐positive SC (Fig 1H) was strongly increased in uninjured aged nerves in comparison to uninjured adult ones, evidencing increased basal senescence markers in aged mice. In addition to SC, other senescent cell types, including macrophages, endothelial cells, and fibroblasts were also increased upon nerve damage, although they represent a smaller population compared to senescent SC (Figs 1J and EV1D and E).

As a decrease in c‐Jun expression is associated with impaired nerve regeneration, and previous reports have shown an inhibitory feedback loop between p16INK4a and c‐Jun (Li *et al*, 2011; Nakano *et al*, 2020), we next evaluated whether the appearance of senescent SC is associated to the expression levels of c‐Jun. To determine the functional significance of this observation, we studied the SC response in mice in which c‐Jun is overexpressed in SC (*Mpz* ^Cre+^; R26c‐Junstop^ff/+^ mice, referred to as c‐Jun OE mice). Importantly, it is known that c‐Jun overexpression in this model restores regeneration after injury in aged animals and after chronic denervation (Arthur‐Farraj *et al*, 2017). Although satellite glial cells in dorsal root ganglia (DRG) probably overexpress c‐Jun in this transgenic mouse line, it has been previously demonstrated that the effect over axonal regeneration is due to c‐Jun overexpression in Schwann cells in the distal stump (Wagstaff *et al*, 2021). After acute denervation, we observed a decreased tendency for SA‐β‐gal activity in the distal stump from aged (1‐year old) c‐Jun OE mice compared to wild type littermates (Fig 1K and L). Furthermore, a robust and significant decrease in p16INK4a‐positive SC after chronic denervation was observed in the distal stump of c‐Jun OE mice compared to wild type mice (Fig 1M and N), suggesting a c‐Jun dependent control of SC senescence.

Taken together, our results indicate an increased accumulation of senescent SC in both aged and chronically denervated neuronal tracts, which correlates with decreased c‐Jun expression and impaired regenerative capabilities.

### Injury‐induced nerve inflammation is enhanced in aged and chronically denervated animals

As senescent cells express a secretory phenotype (SASP) enriched in proinflammatory cytokines (Childs *et al*, 2015; Ferreira‐Gonzalez *et al*, 2018) and previous studies have shown an increase in inflammation in aged and chronically denervated nerves (Scheib & Höke, 2016b), we studied if senescent cells could be associated with this inflammatory reaction in aged and chronically denervated nerves. We first evaluated the increase in cytokines in the distal stump of transected nerves from aged mice submitted to acute denervation, and chronically denervated nerves from adult mice, both conditions characterized in our previous experiments (Fig 1A–D) as leading to poor regenerative outcomes.

**Figure 2:**
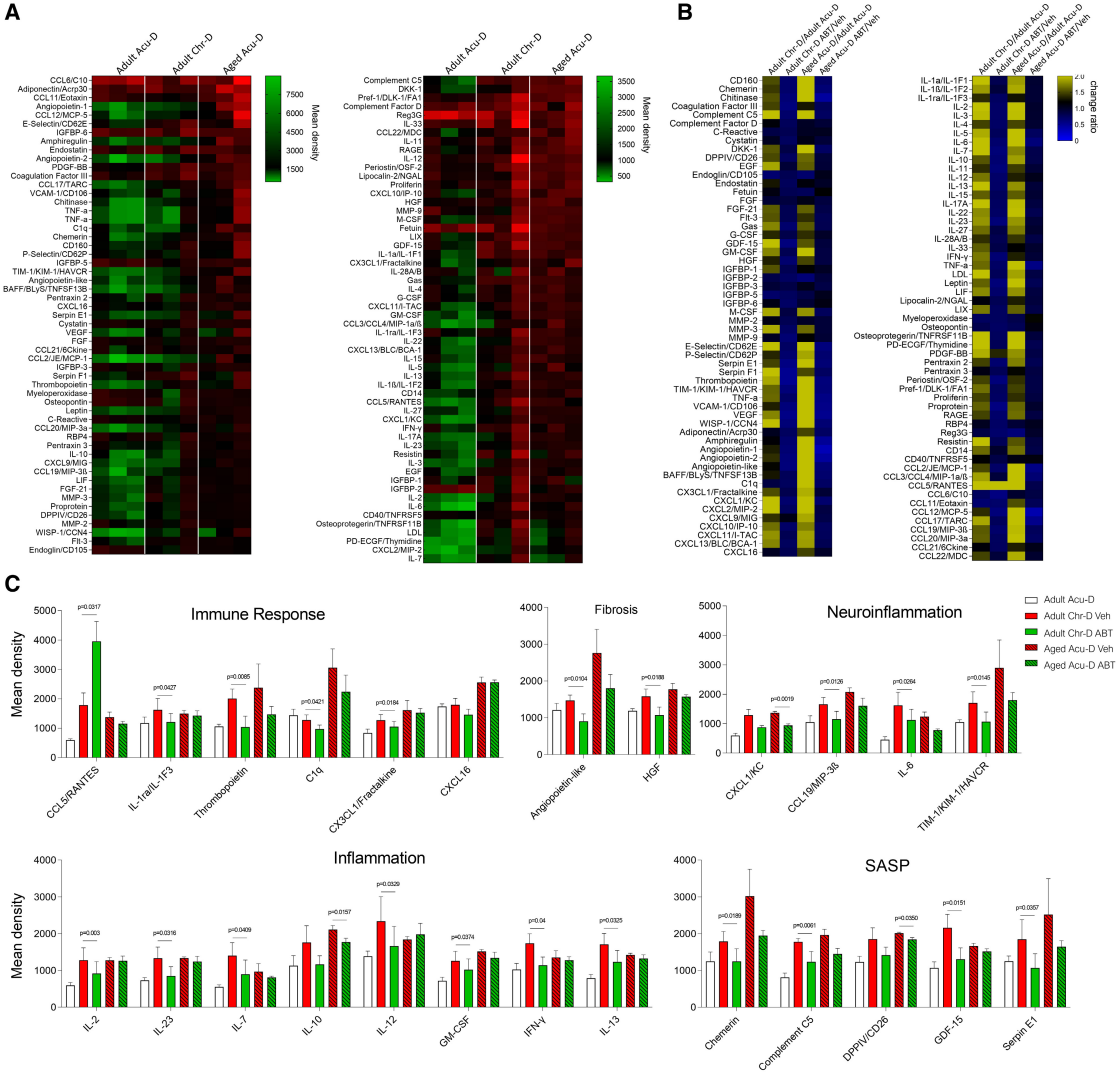
Elimination of senescent SC in aged and chronically denervated nerves alters cytokine profiling *in vivo* Expression analysis of 111 different cytokines in the context of aging or chronic denervation. Comparison between adult chronic denervation (Adult Chr‐D) or acute denervation in aged mice (Aged Acu‐D).Fold change comparison of 111 different cytokines in the context of chronic denervation in adults and acute denervation in aged mice and after ABT‐263 treatment. Comparison between adult mice after Chr‐D and aged mice after Acu‐D in the context of ABT‐263 treatment, vehicle treatment, and compared to adult Acu‐D regenerative response.Expression analysis of 25 different cytokines in the context of aging or chronic denervation. Comparison between Chr‐D in adults or Acu‐D in aging conditions, in mice treated with vehicle or ABT‐263 compared to adult Acu‐D regenerative response. Data are expressed as mean density (*N* = 3 animals per group, *P < 0.05 by Student's *t*‐test compared between conditions; error bars indicate SEM). Source data are available online for this figure.

**Figure EV2:**
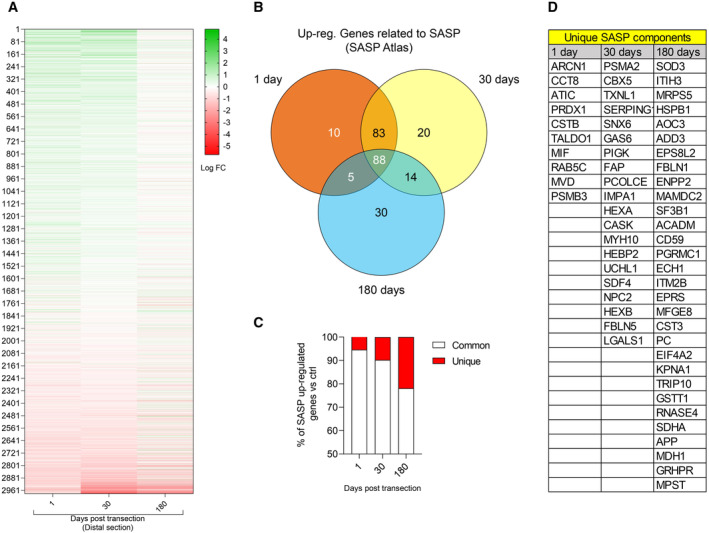
Differentially expressed genes after chronic denervation and their comparison to senescence‐associated secretory phenotype (SASP) Differentially expressed genes in adult distal nerves evaluated 1, 30 and 180 days after sciatic nerve transection.Venn diagram of up‐regulated genes evaluated 1, 30 and 180 days after sciatic nerve transection, contrasted against the SASP‐ATLAS database.Graph comparison of percentage of unique SASP genes against common SASP genes after 1‐, 30‐ or 180‐days post transection.Identification of the unique SASP genes at different times post‐transection. Source data are available online for this figure.

These samples were compared to the distal stump of a nerve of adult mice submitted to acute denervation, which, our data show, have the higher c‐Jun expression in SC, and therefore, a high regenerative potential. From a total of 111 analytes assessed using a cytokine array, 99 and 101 were increased at least twofold in the distal nerve stump after chronic denervation and in aged animals, respectively, compared to adults after acute denervation (Fig 2A, Datasets EV1 and EV2). Of these analytes, 17 were previously described as SASP components, leaving 82 (chronic denervation) and 84 (aging) proteins as new molecular components of aged and chronically denervated nerves. Next, we performed a transcriptomic analysis to search for the expression of SASP‐associated genes in chronically denervated nerves. Our analysis revealed that 250 of 1,646 differentially upregulated genes ranging from acute (1 day) to chronically denervated nerves (180 days), were previously reported as SASP‐related genes (Fig EV2A and B, Dataset EV3). This further validated the increase in SASP components at the transcriptional level in chronically denervated conditions. Interestingly, the proportion of unique SASP components increases with the time of denervation (Fig EV2C). These unique SASP genes range from 10 to 30% of the total SASP as denervation progresses, defining a unique SASP signature for chronic denervation (Fig EV2D). Taken together, our data complement previous results on nerve inflammation in aging and chronic denervation, identifying novel molecules upregulated in these two conditions and providing evidence for the development of an inflammatory condition in the nerve. Importantly, the identification of an important number of SASP‐associated proteins and transcripts strongly suggests that the increase in nerve inflammation could be closely related to the increase in cell senescence after nerve damage in aging and chronic denervation.

### Senescent Schwann cells are inhibitory for axonal growth of sensory neurons *in vitro*

**Figure 3:**
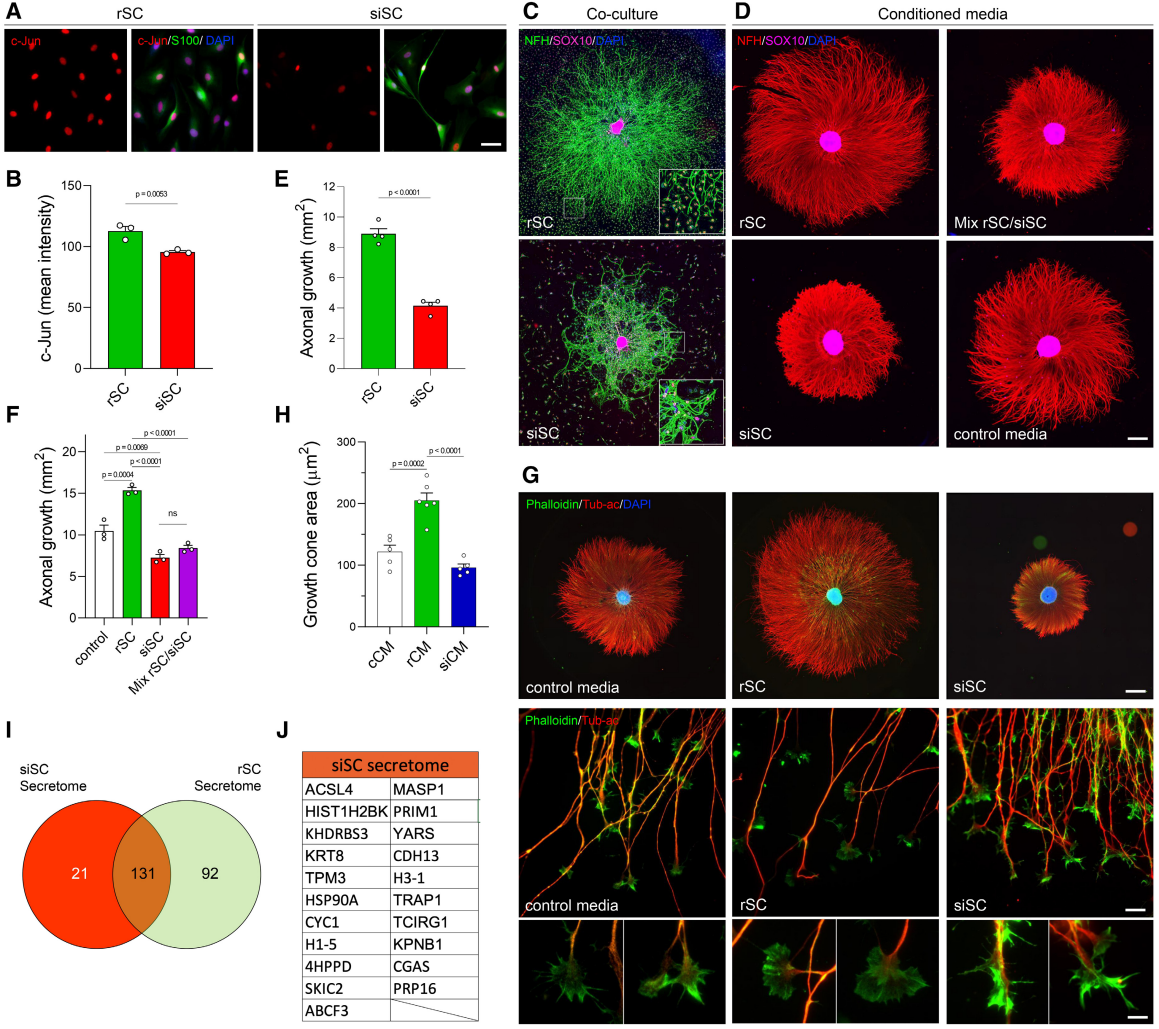
siSC impairs c‐Jun expression and neurite outgrowth *in vitro* A, BRepresentative IF images (A), and quantitative comparison (B) of c‐Jun positive nuclei from primary cultures of rat repair Schwann cells (rSC) and Schwann cells induced to a senescent phenotype (siSC) after doxorubicin treatment (see Materials and Methods for details). *N* = 3. rSC and siSC prepared in this manner were used for following experiments (S100, green; c‐Jun, red; DAPI, blue). Scale bar, 100 μm.C, DRepresentative IF images of DRG reaggregated neurons. In (C) DRG were cocultured either with rSC or siSC. Scale bar 500 μm. In (D), DRG neurons were treated for 72 h with conditioned media derived from rSC, siSC, or a 1:1 mix of both conditioned media. No supplemented media was used as control. Scale bar 1,000 μm.E, FQuantification of axonal growth of DRG neurons cocultured with rSC or siSC (E, *N* = 4) or treated with conditioned media from repair (rCM) or senescent (sCM) Schwann cells (F, *N* = 3) for 72 h.GRepresentative IF images of DRG explants treated for 72 h with conditioned media from rSC or siSC and stained for acetylated tubulin, phalloidin and DAPI. Scale bar 670 μm. Middle and bottom panels, higher magnification images of axonal growth cones present at the tip of the explants. Scale bars, 20 μm for middle panels, and 7 μm for bottom panels.HQuantification of growth cone area (*N* = 5).ISecretome analysis of conditioned media from siSC and rSC compared with SASP‐ATLAS database.JProtein description of the 21 proteins exclusively secreted by siSC. Data information: For all experiments in this figure, *N* = 3–4 DRG reaggregates per group. When two groups were compared, a non‐paired, one‐tailed Student's *t*‐test was performed; when more than two group where compared, One‐way ANOVA with Bonferroni multicomparison post‐test. Data is presented as mean ± SEM. Source data are available online for this figure.

**Figure EV3:**
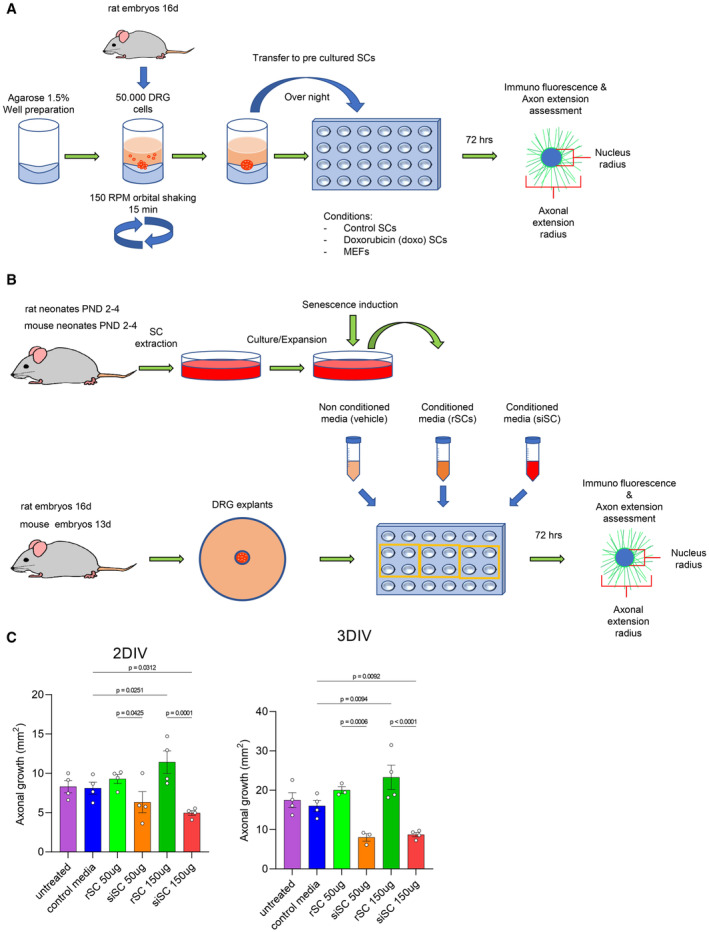
Schematic representation of *in vitro* experiments Scheme of the DRG and SC co‐culture protocol.Methodological scheme of the obtention of conditioned media from SC and the treatment of DRG with treatment with conditioned media protocol.Comparison between DRG re‐aggregates after 2 or 3 days *in vitro* (DIV) of exposure to control media, and conditioned media from SC or siSCs in concentrations of 50 or 150 μg of proteins from collected media, compared to untreated DRGs. *N* = 3–4 re‐aggregates per group; *P < 0.05 by Student's *t*‐test compared between conditions; error bars indicate SEM. Source data are available online for this figure.

**Figure EV4:**
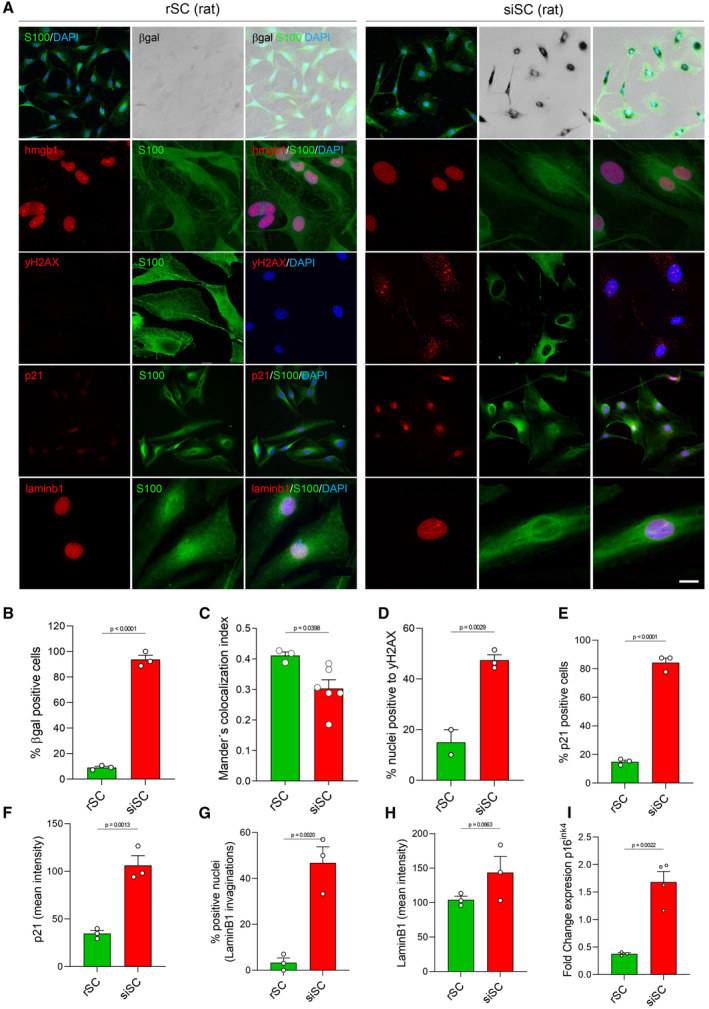
Markers of senescence in rat primary Schwann cell culture after doxorubicin treatment ARepresentative IF images in rSC and siSC, S100, green, βgal, black; Hmgb‐1/yH2AX, red; DAPI, blue. Scale bar, βgal, 100 μm; p21, 100 μm; yH2AX, 50 μm; hmbgb‐1, 25 μm; laminb1, 25 μm.B–IGraph comparison of β‐gal^+^ cells (B), Mander's co‐localization index of HMGB1 (C), yH2AX foci/nucleus (D), p21 positive cells (E) and expression levels (F), nuclei positive for LaminB1 marked invaginations (G) and expression (mean intensity) (H), p16INK4a fold change (qRT‐PCR) (I) between non‐senescent and siSCs and rSCs. *N* = 3–6 per condition; *P < 0.05, **P < 0.01 by Student's *t*‐test compared between conditions; error bars indicate SEM. Source data are available online for this figure.

To establish the effect of senescent Schwann cells on axonal growth, we first used an *in vitro* model. rSC were treated with the anti‐carcinogenic drug doxorubicin, a widely used senescence inducer (Childs *et al*, 2015) (Fig EV3). After doxorubicin treatment for 9 days, more than 90% of SC were positive for SA‐β‐gal activity (Fig EV4A and B). SC treated with doxorubicin show significant translocation of HMGB1 from the nucleus towards the cytosol, increased p‐γH2AX foci, and increased p21^CIP1^ positive nuclei, compared to rSC (Fig EV4A–G), all well‐described markers for cell senescence (Hernandez‐Segura *et al*, 2018; Ogrodnik, 2021; Heckenbach *et al*, 2022; Matias *et al*, 2022). In addition, lamin B1 expression was increased, together with an increase in lamin B1‐positive nuclear invaginations (Fig EV4A, H and I), which is considered a change in nuclear morphology associated with the cellular senescence (Matias *et al*, 2022). Importantly these senescence‐induced SC (siSC) retain their SC identity, assessed by the expression of S100 and SOX10 (Figs 3A and EV4A). Interestingly, the induction of a senescent phenotype decreases c‐Jun expression in siSC compared to rSC (Fig 3A and B).

**Figure EV5:**
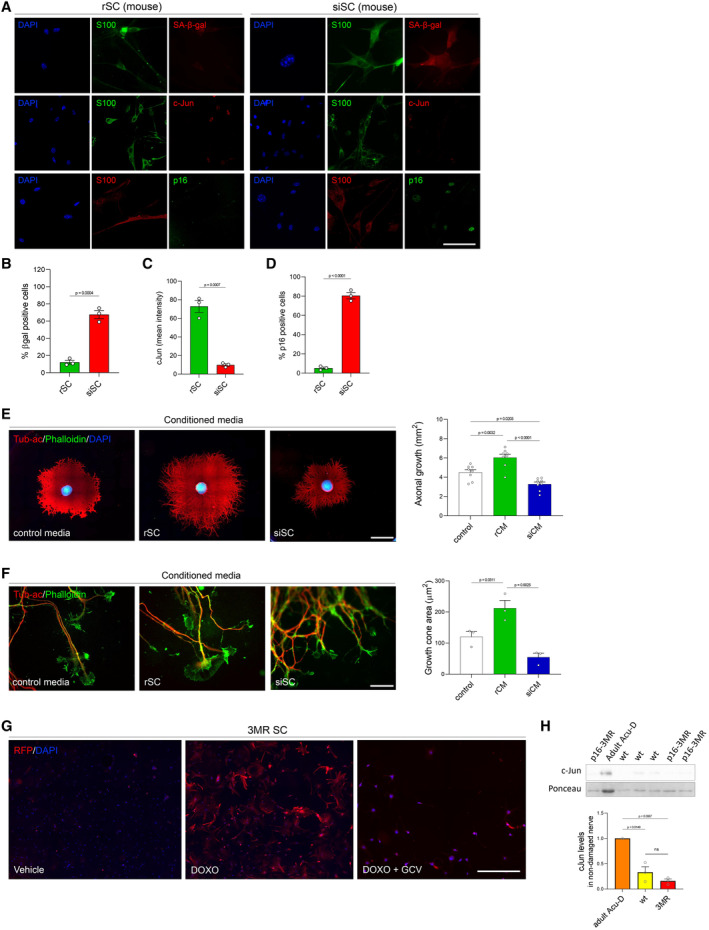
Markers of senescence in mouse primary Schwann cell culture after doxorubicin treatment. Mouse siSC impairs c‐Jun expression and neurite outgrowth *in vitro* ARepresentative IF images of mouse rSC and siSC stained with different senescent markers (SA‐β‐gal, p16) or c‐Jun together with the SC marker S100 and the nuclei marker DAPI. Scale bar, 50 μm.B–DGraph comparison of β‐gal positive cells (B), c‐Jun nuclei intensity (C) and % of p16 positive cells (D). *N* = 3 per condition; *P < 0.05, **P < 0.01 by Student's *t*‐test compared between conditions; error bars indicate SEM.ERepresentative IF images of mouse DRG neurons. In (A) DRG were treated for 72 h with conditioned media derived from control, rSC, and siSC. Scale bar, 500 μm. To the right, the graph shows the quantification of axonal growth of DRG neurons comparing the different treatments.FHigher magnification images of axonal growth cones present at the tip of the explants from DRGs treated in (A). Scale bars, 50 μm. To the right, the graph shows the quantification of the size of the growth cone between treatments. *N* = 3 for each condition. One‐way ANOVA with Bonferroni multicomparison post‐test. *P < 0.05, **P < 0.01, ***P < 0.001, ****P < 0.0001. Data is presented as mean ± SEM.GPrimary cultures of SC from p16‐3MR mice were treated with doxorubicin to induce senescence (see Materials and Methods for details). After senescence induction (DOXO), red fluorescent protein (RFP) is robustly expressed compared to vehicle‐treated Schwann cells. Ganciclovir (GCV) treatment eliminates most RFP‐expressing siSC. Scale bar, 200 μm.HBasal expression of c‐Jun by Western blot in undamaged sciatic nerves from wild type and p16‐3MR mice. Besides the already low expression of c‐Jun in undamaged nerves in wild type mice compared to injured nerves (adult Acu‐D), the levels of c‐Jun are comparable between p16‐3MR and wild type mice. Source data are available online for this figure.

We next used siSC to establish their effect on axonal growth. We first cultured explants of sensory neurons over monolayers of rSC or siSC, measuring axonal growth after 72 h (Fig EV3). Importantly, siSC have a strong inhibitory effect over axonal growth compared to neurons co‐cultured with rSC (Fig 3C and E). As the effect of senescent cells is usually associated with their secretory phenotype (Ferreira‐Gonzalez *et al*, 2018), we evaluated the impact of the secreted component of siSC on axonal growth. We first performed proteomic analysis of the rSC and siSC secretome. Our results indicate that a total of 21 proteins are exclusively expressed in siSC (Fig 3I, Dataset EV4), and all of them are reported as SASP components by the SASP‐Atlas identification tool (Basisty *et al*, 2020). We next treated sensory neuron explants with conditioned media (CM) from rSC, siSC, and a mix of both (rSC/siSC) and axonal growth was measured 72 h after CM treatment (Fig EV3). Conditioned media from rSC enhances axonal growth compared to control media. By contrast, conditioned media from siSC strongly inhibits axonal growth (Fig 3D and F) and could even overcome the pro‐regenerative effects of rSC media, with levels of inhibition comparable to siSC media alone (Fig 3D–F). Since our *in vivo* experiments were performed in mice, we aimed to validate if the *in vitro* characteristics of rSC and siSC from rats were replicated in cultured SC from mice. We confirmed that SC from mice exhibits senescence markers when treated with doxorubicin (Fig EV5), and that conditioned media from siSC have an inhibitory effect over axonal growth (Fig EV5).

As growth cone morphology is tightly coupled to axonal growth, we analyzed these structures after CM treatment. After treatment with CM derived from rSC, growth cones adopted a lamellipodium‐like structure (Gallo, 2013; Roselló‐Busquets *et al*, 2019), which was quantitatively different from the more filopodia‐like shape exhibited by untreated neurons when the growth cone area was measured (Fig 3F and G). In contrast, CM derived from siSC led to a collapsed morphology of the whole growth cone, suggesting a retraction process of the filopodium and lamellipodium, in both rat and mice neurons (Fig 3F and G).

Altogether, these data demonstrate that the secreted molecules from siSC, enriched in SASP‐components, negatively affect axonal growth and modify structural characteristics at the level of the growth cone.

### Senescent cell elimination restores c‐Jun levels in SC, and injury‐induced nerve inflammation in aged and chronically denervated animals

**Figure 4:**
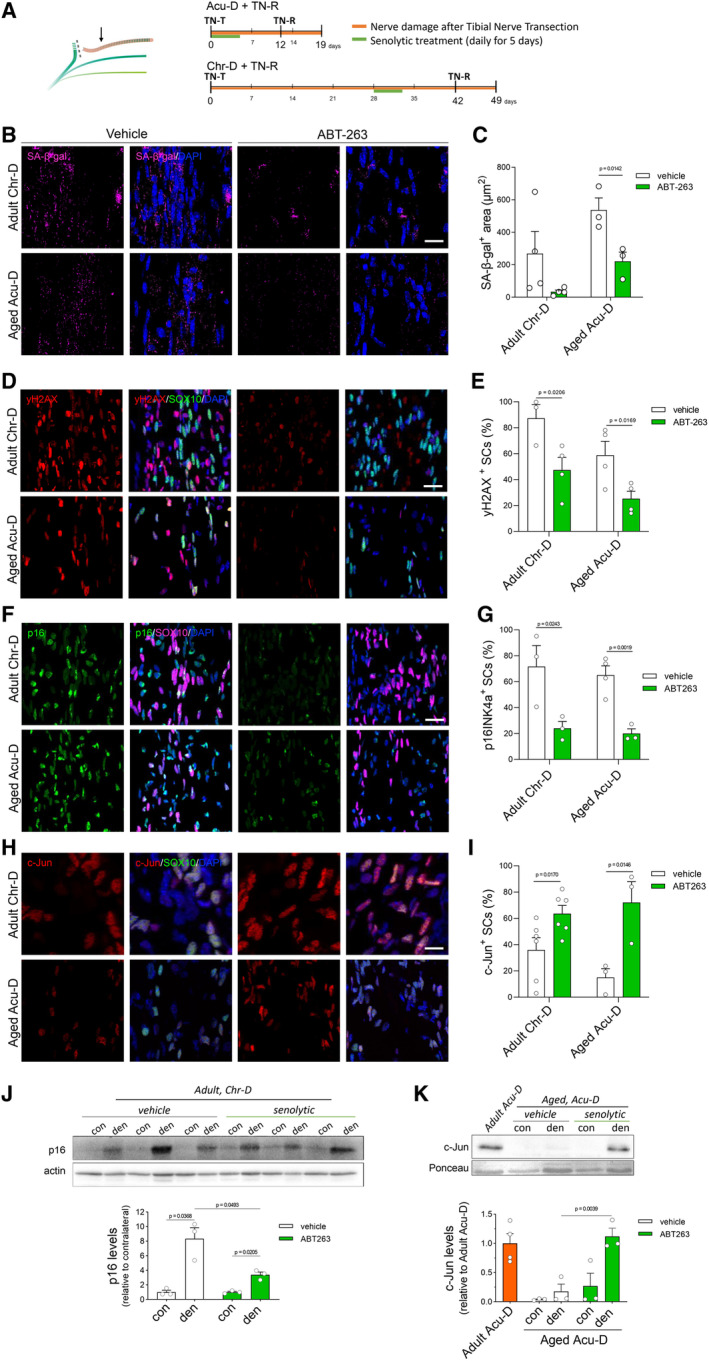
ABT‐263 treatment reduces the expression of senescence markers and increases c‐Jun translocation in SC ASchematic representation of the experimental condition and timeline for tibial nerve transection (TN‐T) and senolytic treatment in adult mice submitted to Chr‐D and aged mice submitted to Acu‐D. Groups were treated with vehicle or senolytic drug ABT‐263, given by daily gavage for 5 days, at a 50 mg/kg dose. Arrow in the left scheme corresponds to the site of imaging.B, CConfocal microscopy and quantification graph comparing β‐galactosidase activity between treatments (Vehicle and ABT‐263) in adult mice with Chr‐D (*N* = 4) and aged mice with to Acu‐D (*N* = 3).D–IRepresentative IF confocal images of longitudinal cryostat sections and quantification graphs indicating SC positive for senesce markers γ‐H2AX (D, E), p16INK4a (F, G), and transcription factor c‐Jun (H, I), comparing vehicle and ABT‐263 treated adult mice with Chr‐D and aged mice with Acu‐D (*N* = 3–6. Specific n is noted as dots in each graph).J, KImmunodetection by western blot of p16INK4a (J, *N* = 3 per group) and c‐Jun (K, *N* = 3 per group) in nerve homogenates from adult mice with Chr‐D and aged mice with ACu‐D, respectively, that were treated with vehicle or ABT‐263. Data information: For all experiments in this figure: Scale bars, 50 μm. Non‐paired, one‐tailed Student's *t*‐test was used to compared vehicle versus ABT treated pairs. Data is presented as mean ± SEM. Source data are available online for this figure.

Having established that siSC strongly inhibits axonal growth *in vitro*, we aimed to assess the impact of senescent cells over injury‐induced nerve changes *in vivo*. To this end, we eliminated senescent cells in wild type mice using the senolytic drug ABT‐263, a specific inhibitor of the anti‐apoptotic proteins BCL‐2 and BCL‐xL (Chang *et al*, 2016). We performed systemic senolytic treatment in aged mice submitted to acute denervation (Fig 4A, top timeline) or adult mice submitted to chronic denervation (Fig 4A, bottom timeline). In both conditions, a clear reduction in β‐gal staining was observed in damaged nerves from treated mice compared with nerves from vehicle‐treated animals (Fig 4B and C). Furthermore, the number of SC positive for the senescence markers y‐H2AX and p16INK4a were significantly reduced after the treatment with ABT‐263 in both aged mice submitted to acute denervation or adult mice submitted to chronic denervation (Fig 4D–G), Additionally, we evaluated protein levels of p16INK4a in homogenates from damaged and contralateral nerves, and we observed that p16INK4a total levels were also reduced after senolytic treatment (Fig 4J). Remarkably, the number of c‐Jun‐positive SC was significantly increased after senolysis in nerves from chronically denervated adult mice and acutely denervated aged mice (Fig 4H and I), and the total protein levels for c‐Jun in aged with Acu‐D returned to levels comparable to nerves with high regeneration capacity (i.e., adults submitted to Acu‐D) (Fig 4K).

We then used the senolytic drug ABT‐263 to evaluate the contribution of senescent cells in the inflammatory reaction of aged and chronically denervated nerves. Interestingly, 25 of 111 proteins that were up‐regulated in both aging and chronic denervation (adult Chr‐D/aged Acu‐D vs. adult Acu‐D) were significantly decreased after ABT‐263 treatment in either one or both conditions (Fig 2B, Datasets EV1 and EV2). Interestingly, these factors belong to gene ontology (GO) annotations closely related to cell senescence, including immune response, inflammation, fibrosis, neuroinflammation, and SASP (Hernandez‐Segura *et al*, 2018; Paramos‐de‐Carvalho *et al*, 2021). Of the 25 identified factors, five were previously described as SASP factors, including C5, DPP4, IGFBP‐3, Serpin E1, Chemerin/RARRES2, and GDF15 (Basisty *et al*, 2020), which in both conditions showed a significant decrease after treatment with ABT‐263 (Fig 2B). Some identified factors, such as CXCL1, CCL19, IL‐6, and TIM‐1, are significantly decreased in both aging and chronic denervation (Fig 2C) and are tightly related to neuroinflammation upon nerve injury (Scheib & Höke, 2016b; Deftu *et al*, 2019; Zheng *et al*, 2019; Hu *et al*, 2020; Guo *et al*, 2021). Particularly, CXCL1 is reported to inhibit axonal outgrowth of sensory neurons (Deftu *et al*, 2019). These results confirm the elevation of proinflammatory factors previously described in aged nerves after injury and chronically denervated ones and identify several new cytokines that are upregulated in these conditions. Importantly, our data demonstrate that this proinflammatory reaction is associated with a senescent cell population that appears in injured nerves from aged animals and after chronic denervation.

### The elimination of senescent cells improves axonal regeneration and functional recovery after nerve injury in aging and chronic denervation

**Figure 5:**
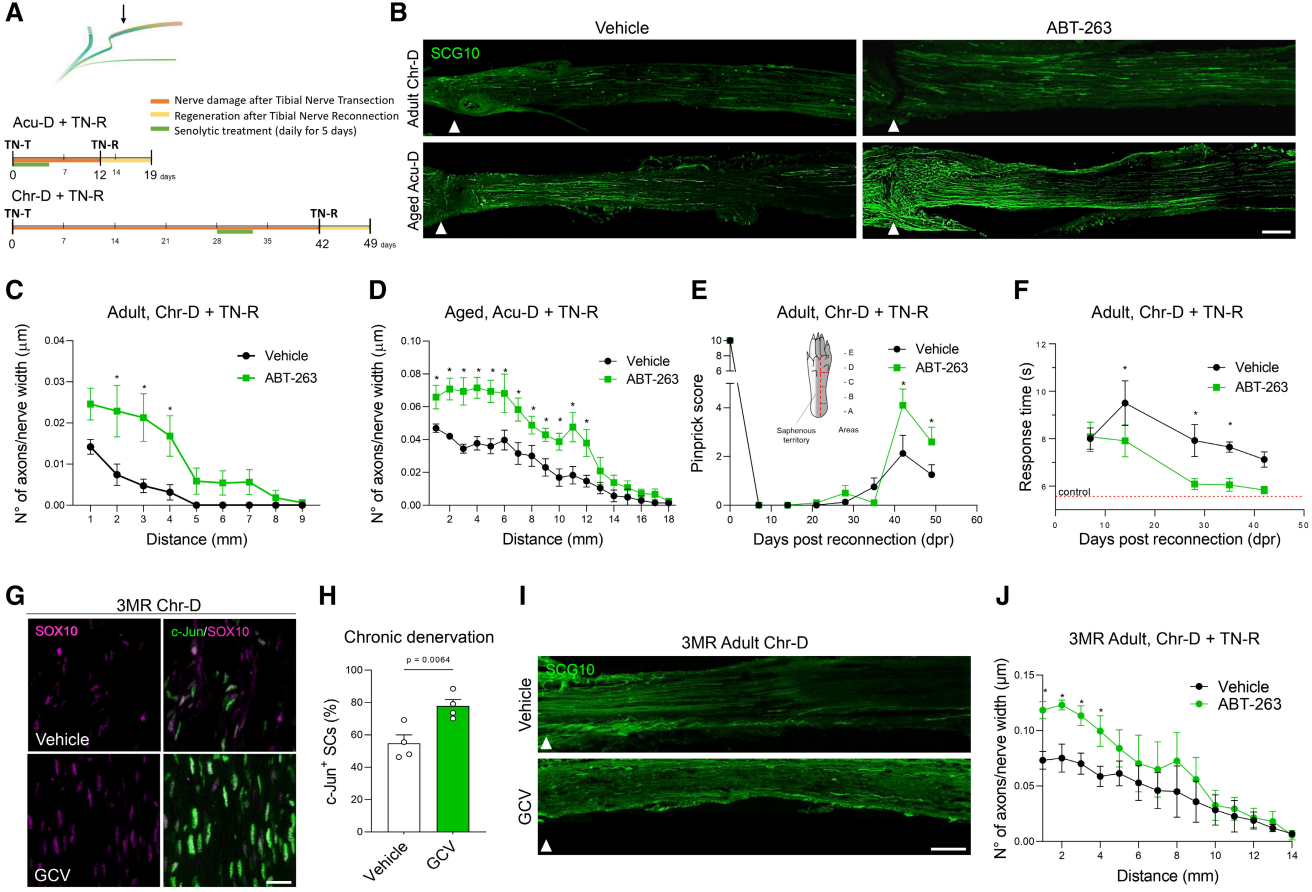
ABT‐263 treatment increases axonal regeneration and c‐jun expression in SC in aging or chronically denervated sciatic nerves ASchematic representation of the experimental condition and timeline for tibial nerve transection (TN‐T), senolytic treatment and tibial nerve reconnection (TN‐R), performed in adult mice submitted to Chr‐D and aged mice submitted to Acu‐D. Groups were treated with vehicle or senolytic drug ABT‐263 for wild type mice, given by daily gavage for 5 days, at 50 mg/kg, or Ganciclovir (GCV) for P16‐3MR mice, given by daily IP, at 25 mg/kg. Arrow in the scheme corresponds to the site of imaging.BRepresentative IFI (SCG10) of nerve longitudinal sections from vehicle and senolytic‐treated mice, evaluated 7 days post TN‐R. Arrowheads indicate the reconnection site (see Materials and Methods for details). Scale bar, 500 μm.C, DQuantification of the density of SCG10‐positive from the reconnection site to distal regions in nerves from vehicle and ABT‐263 treated mice, in adult mice with Chr‐D (C, *N* = 3 for vehicle and *N* = 5 for treated) and aged mice with Acu‐D (D, *N* = 4 per group). The dataset used for the vehicle controls of (B–D) was used for basal values shown in Fig 1B and C.E, FFunctional evaluation of hindlimb sensitivity to mechanical (Pinprick assay, E) and thermal (Hargreaves assay, F) stimuli. Inset in (E) shows in gray the area of the hind paw innervated by the tibial branch. Tests were performed in this region of the hind paw in mice treated with vehicle or ABT‐263, up to 48 days post reconnection in adult mice that were chronically denervated. *N* = 8–10 animals per group. In F, the response time to thermal stimuli in uninjured hind paw is shown as a red dotted line.G, HRepresentative IF confocal images and quantification graph comparing c‐Jun‐positive SC (stained for SOX10) on longitudinal cryostat sections of adult 3MR mice submitted to Chr‐D, treated with vehicle or GCV. *N* = 4 animals per group. Scale bar, 50 μm.IRepresentative IF (SCG10) of nerve longitudinal sections from vehicle and senolytic (GCV) treated P16‐3MR mice that were chronically denervated, evaluated 7 days post TN‐R. Arrowheads indicate the reconnection site (see Materials and Methods for details). Scale bar, 400 μm.JQuantification of the density of SCG10‐positive from the reconnection site to distal regions in nerves from vehicle (*N* = 5) and GCV‐treated (*N* = 6) P16‐3MR mice. Data information: Non paired, one tailed, Student's *t*‐test was used to compare vehicle versus ABT treated pairs. If not noted in the graph, exact *P*‐values (*from those *P* < 0.05), can be seen in Appendix Table S2. Data is presented as mean ± SEM. Source data are available online for this figure.

As senescent cell elimination leads to increased c‐Jun levels and dampens nerve inflammation in injured nerves from aged animals and after chronic denervation, we assessed the impact of senolytic treatment on axonal regeneration after nerve repair. As our results demonstrate a basal accumulation of senescent cells in aged nerves before nerve injury (Fig 1E–J), we treated aged mice with senolytic (ABT‐263) immediately after transection, aiming to eliminate resident senescent cells. We then performed tibial nerve reconnection to the freshly transected common peroneal nerve 7 days after the end of the senolytic treatment, and analyzed regeneration 7 days after, as shown in the upper scheme of Fig 5A. Notably, treatment with ABT‐263 strongly improved axonal regeneration compared to vehicle‐treated animals (Fig 5B and C) in aged mice submitted to acute denervation, suggesting that resident senescent cells within aged nerves inhibit axonal regeneration.

To assess the effect of senescent cell elimination over regeneration in chronically denervated nerves, ABT‐263 treatment was applied to adult mice 28 days post‐injury, and 7 days after the end of the treatment, nerves were reconnected to analyze regeneration 7 days later (lower scheme in Fig 5A). Impressively, ABT‐263 treatment increased regenerating axons by twofold compared to vehicle‐treated animals, and almost double the distance of the regenerating front (Fig 5B–D). To establish if the improvement in axonal regeneration after ABT‐263 was associated with a better functional outcome, we evaluated the recovery of nociceptive sensitivity after reconnection of chronically denervated nerves in adult animals. Light touch and temperature sensitivity in the skin territory innervated by the tibial nerve (see inset in Fig 5E) was assessed by the Pinprick and Hargreaves tests (Deuis *et al*, 2017), respectively. Notably, compared to vehicle‐treated animals, ABT‐263 treatment led to a better functional recovery of touch and temperature sensitivity at 4 and 6 weeks post‐reconnection, respectively (Fig 5E and F).

To further confirm the role of senescent cells in inhibiting axonal regeneration, we use the p16‐3MR transgenic mice to genetically eliminate senescent cells. Senescent cells in p16‐3MR mice express red fluorescent protein (RFP) and the herpes simplex virus 1 thymidine kinase (HSV‐TK), thereby sensitizing p16Ink4a‐expressing cells to ganciclovir (GCV) (Demaria *et al*, 2014). Importantly, purified SC from p16‐3MR mice induced to senescence by doxorubicin treatment express RFP and are eliminated by GCV (Fig EV5A). After denervation of adult p16‐3MR mice for 28 days, we treated with GCV for 5 days, and after 7 days, nerves were reconnected (same treatment design as for ABT‐263 described in Fig 5A, upper scheme). Importantly, the elimination of senescent cells after GCV treatment in p16‐3MR mice leads to increased c‐Jun expression in SC from chronically denervated nerves (Fig 5G and H), as it was found after senolysis with ABT‐263 in wild type mice (Fig 4H and I). Furthermore, assessment of axonal regeneration reveals an increase in regenerating axons of GCV‐treated p16‐3MR mice compared to vehicle‐treated ones (Fig 5I and J). Importantly, the basal levels of c‐Jun in p16‐3MR and wild type mice are statistically indistinguishable (Fig EV5H). These results confirm that the selective removal of senescent SC by pharmacological and genetic approaches improves both regeneration and functional recovery after chronic denervation.

## Discussion

For the past two decades, it has been recognized that the pro‐regenerative effect of SC over injured axons is reduced in the context of aging and chronic denervation (Vaughan, 1992; Jessen & Mirsky, 2019). Here, we demonstrated for the first time the presence and functional role of senescent SC in injured peripheral nerves and found that this senescent SC phenotype has detrimental effects over axonal regeneration in aged or chronically denervated animals, opening novel possibilities to improve functional recovery after peripheral nerves injuries.

Our results demonstrate that senescent Schwann cells arise in peripheral nerves after injury in aging and chronic denervation, and probably correspond to previous observations of SC exhibiting senescent markers, and populating long acellularized grafts (Saheb‐Al‐Zamani *et al*, 2013; Poppler *et al*, 2016), demonstrating now that these cells appear in uninjured and injured nerves, and are inhibitory for axonal regeneration. The diminished expression of c‐Jun on senescent SC confirms previous works in the context of chronic denervation and aging (Wagstaff *et al*, 2021), but now defining the identity of these low c‐Jun expressing Schwann cells. Given that c‐Jun is essential for the reprogramming and maintenance of SC in a repair phenotype (Arthur‐Farraj *et al*, 2012), it will be important to define the relationship between c‐Jun expression and SC senescence. Previous works have shown that p16 inhibits c‐Jun phosphorylation by associating to the N‐terminal region of JNK kinases (Choi *et al*, 2005), and that c‐Jun suppresses p16 expression by binding to its promoter region (Li *et al*, 2011; Nakano *et al*, 2020). As c‐Jun expression declines after SC remain chronically denervated (Arthur‐Farraj *et al*, 2012), a transition into a senescent SC phenotype could be associated with the upregulation of p16, which in turn could generate a feedforward loop, further preventing c‐Jun expression. Indeed, we found that forced overexpression of c‐Jun prevents the increase in p16‐positive SC in chronically denervated nerves (Fig 1M and N), an intervention that has been shown to rescue the failure in nerve regeneration caused by aging or chronic denervation (Wagstaff *et al*, 2021). These assumptions are backed by our experiments, as after genetic or pharmacological elimination of senescent cells, the nerve exhibit a population of non‐senescent SC with increased levels of c‐Jun (Figs 4H and I, and 5G and H). If these are cells already present in the nerve that upregulate c‐Jun or is associated with SC re‐entering into a proliferative, repair phenotype remains to be investigated. These data indicate that the interaction with senescent cells not only negatively affects c‐Jun expression in non‐senescent SC but is also a transient effect that can be reversed or ameliorated by targeted elimination of the population of senescent cells within the nerve. In addition, it suggests a cross regulation between p16 and c‐Jun in sSC. As senescence affects a wide range of processes within the cell, further experiments are needed to dissect the specific mechanisms underlying the transition of Schwann cells from repair into a senescent phenotype.

Our data consistently show the presence of senescent SC in both chronic denervation and aging after nerve transection, although the mechanism associated with the senescent phenotype induction is unclear. Injury‐induced SC transition into a repair phenotype is associated with re‐entry into a replicative state, which is inhibited by regenerating axons invading the distal nerve stump (Jessen & Mirsky, 2016). Nevertheless, in chronically denervated nerves, rSC continue in a high replicative state, which is one of the triggers of cell senescence (Herranz & Gil, 2018; Jessen & Mirsky, 2019). In addition, constant replication is associated with the upregulation of a DNA damage response (DDR), which can also lead to cell senescence (Gomez‐Sanchez *et al*, 2013; Herranz & Gil, 2018). In aged nerves, SC have shown to have defects in their transcriptional machinery associated with DNA damage, especially in regeneration‐associated genes (RAGs) (Scheib & Höke, 2016a; Painter, 2017).

In damaged nerves from adult (Chr‐D) and aged (Acu‐D) p16‐positive senescence nuclei were found in SOX‐positive and SOX10‐negative cells, composing up to 60% of the total cells within the nerve (Fig 1I). Peripheral nerves are also composed of fibroblasts, perineural, and endothelial cells, as well as resident macrophages (Gerber *et al*, 2021; Yim *et al*, 2022). After nerve injury, changes in the number and phenotype of some of these cells, especially macrophages, have been reported (Qian *et al*, 2018; Shen *et al*, 2022). Our detailed analysis of senescent cells using specific markers for macrophages, fibroblasts and endothelial cells demonstrate that these cells together contribute to the total senescent population in damaged nerves in both aging and chronic denervation, albeit in a lower percentage than Schwann cells (Fig 1J). Indeed, in damaged nerves from adult and aged animals, it has been shown that the activity of infiltrating macrophages is characterized by an attenuated phagocytosis and the secretion of proinflammatory factors within the nerve (Scheib & Höke, 2016b; Painter, 2017; Büttner *et al*, 2018). Importantly, it has been reported that some of these factors, such as iNOS, TNFα, IL‐1β, and IL‐6, are expressed in both SC and macrophages (Wang *et al*, 2009; Xue *et al*, 2018). Specifically, high levels of NO have been shown to induce cellular senescence (Bagheri *et al*, 2017). This raises the question of whether macrophages contribute to SC senescence or become senescent by the SASP released by sSC. Although our experiments *in vitro* demonstrate that siSC‐derived SASP directly inhibits axonal growth, secreted molecules from senescent cells other than SC within the nerve might also contribute to the inhibition of axonal regeneration, or to the induction of cell senescence by a bystander effect, as it has been reported in other conditions (Acosta *et al*, 2013). More detailed analysis on the effect of different cells types over SC senescence and axonal regeneration in controlled *in vitro* conditions will be necessary to dissect different contributions. In addition, cell‐specific senolysis (Wang *et al*, 2021) will be required to fully understand the contribution of different cell populations in *in vivo* conditions in chronically denervated and aged nerves after injury.

The current hypothesis in the field is that low c‐Jun expressing SC lose their pro‐regenerative capacity after chronic denervation and aging (Wagstaff *et al*, 2021). However, our data suggest a more complex scenario, in which sSC acquires a phenotype inhibitory for axonal growth. Although the elimination of sSC is sufficient to rescue regenerative capacities in aged and chronically denervated nerves, the appearance of c‐Jun expressing Schwann cells after senolysis suggests the possibility that the elimination of the inhibitory sSC component plus the activation of rSC phenotype is associated with the regenerative response.

Associated with the inhibitory effect of sSC on axonal growth, our *in vitro* data demonstrate an important contribution of secreted factors, which are able to overcome the pro‐regenerative effect of secreted factors released by rSC. Our results demonstrate a high abundance of SASP‐related transcripts, proinflammatory cytokines, and proteins in both aged and chronically denervated nerves (Figs 2A–C and EV2). These are probably associated with sSC as well as other senescent cells populating the damaged nerve. In addition to SC, it is important to consider macrophages, as although they are necessary for regeneration at early stages following nerve injury (Jessen & Mirsky, 2016), their prolonged presence may inhibit regeneration and has been previously associated with tissue dysfunction in aging (Büttner *et al*, 2018). Our cytokine array analysis suggests several candidates that might contribute to the inhibitory environment. Interestingly, of 111 proinflammatory cytokines analyzed, 25 were downregulated in aged or chronically denervated nerves after senolytic treatment, and only three showed clear downregulation in both conditions, IL‐6, CCL19, and CXCL1. Both SC and macrophages secrete IL‐6 upon injury, and has been associated with several age‐related diseases such as multiple sclerosis, Alzheimer's disease, diabetes, and systemic lupus (Choy *et al*, 2020). Interestingly, macrophages suppress SC proliferation and maturation through IL‐6 secretion (Stratton *et al*, 2018). Particularly, CXCL1, which is also secreted by both SC and macrophages, has been associated with physical inhibition of axonal outgrowth in DRG neuron (Deftu *et al*, 2019). CXCL1 interferes with the functioning of TRPV1 (Transient Receptor Potential 1) receptors in TRPV1^+^/IB4 (Isolectin B4)^+^ DRG neurons (Deftu *et al*, 2017). These effects are important as studies have shown that TRPV1 receptors are physically and functionally present at dynamic neuronal extensions, including growth cones of embryonic and adult DRG neurons (Goswami *et al*, 2007). Strikingly, this effect can be related with the changes in growth cone morphology observed in response to conditioned media from sSC (Fig 3F and G). This data demonstrate for the first time that the growth cone morphology can be a target of secreted factors from senescent cells, which could have important consequences in other injured tissues that require innervation for proper function.

Taken together, our results show that the development of senescence in SC undermines axonal regeneration, by preventing or counteracting the activation of the reparative program in non‐senescent SC and through direct influence on growth cone dynamics. This effect can be reversed after the pharmacological and genetic elimination of senescent cells within the nerve, increasing levels of c‐Jun and thus the activity of the reparative phenotype of SC. Chronic denervation and aging are the main clinical problems associated with peripheral nerve injuries, even though effective treatment has been elusive due to the incomplete understanding of the underlying causes of this regenerative failure (Lanier *et al*, 2021). Our data strongly suggest that SC senescence can be regarded as one of the principal mechanisms associated with inhibition of axonal regeneration. Our approach, using FDA‐approved drugs currently in clinical trials for their application as senotherapeutics (Kirkland & Tchkonia, 2020), effectively broadens the spectrum of its clinical use and effectivity. Finally, this work shows that the pharmacological ablation of the sSC population using a senolytic drug can significantly enhance regeneration performance after nerve injury in aged and chronic denervation conditions, leading us one step closer to improved clinical applications.

## Materials and Methods

### Wild type and transgenic mice

C57BL/6J mice were obtained from The Jackson Laboratory, and maintained a s a colony in the animal facility of Universidad Mayor. Mice that overexpress c‐Jun selectively in Schwann cells was generated as described (Fazal *et al*, 2017), conformed to UK Home Office guidelines under the supervision of University College London (UCL) Biological Services under Protocol No. PPL/70/7900. p16‐3MR transgenic mice (a C57BL/6J strain) were generated as previously described (Demaria *et al*, 2014) (BUCK Institute for Research on Aging, Novato CA), and were bred in the animal facility of the Universidad Mayor. For all experiments, mice were housed in cages with three to five individual, with 12 dark/12 light cycle and controlled temperature, with *ad libitum* access to food and water. Mice groups were randomized for surgical and/or treatments. All animal procedures were carried out in accordance with the protocol approved by the Universidad Mayor Animal Care Committee Guidelines.

### Tibial nerve transection and reconnection

C57BL6 mice from different ages (adult: 2–4 months old and aged: 20–22 months old) were anesthetized with isoflurane (Baxter, Illinois, USA) and the right sciatic nerve was exposed between the hipbone and the sciatic notch (Fig 1A). Afterwards, the sciatic branches were isolated, and the tibial nerve was transected, and the distal tibial nerve sutured using an 11‐0 suture to the nearest muscle to prevent reconnection. Acute denervation (Acu‐D) was defined as 7–12 days post‐injury (dpi) and chronic denervation (Chr‐D) was defined as 42 dpi. For reconnection surgery, the tibial nerve was detached from the muscle and reconnected with a 10‐0 suture to the freshly transected proximal common peroneal nerve. In all conditions, the nerve was reconnected 12 or 42 days post‐injury (dpi), and reconnected nerves were collected for analysis 7 days post reconnection. Contralateral uninjured nerves were used as non‐damaged controls.

### Senolysis assays

For senolysis assays, the sciatic nerves of adult (2–4 months old) and aged (20–22 months old) C57BL6 mice were transected as detailed above. Treatment with senolytic ABT‐263 was performed as follows: animals were submitted to gavage with a dose vehicle or ABT‐263 50 mg/kg (navitoclax) diluted on DMSO and corn oil in a 1:10 proportion respectively, for 5 consecutive days. For acute denervation assays, animals were treated immediately after injury. For chronic denervation assays, mice were treated 28 days after injury. After senolytic treatment, mice were left for 7 days to allow senolysis to develop. After both treatments, reconnection surgery was performed in the treatment and vehicle groups as described previously. Treatment with Ganciclovir in p16‐3MR mice was performed by intraperitoneal injection of vehicle or GCV 25 mg/kg, at the same time points indicated for ABT‐263 treatment.

### Assessment of sensory function

Pinprick assay was performed as previously described (Ma *et al*, 2011; Deuis *et al*, 2017). An Austerlitz insect pin (size 000) (FST) was gently applied to the plantar surface of the paw without moving the paw or penetrating the skin. The most lateral part of the plantar surface of the hind paw (sensory field of the sciatic nerve) was divided into five areas. The pinprick was applied (twice) from the most lateral toe (area E) to the heel (area A). A response was considered positive when the animal briskly removed its paw, and the mouse was graded 1 for this area, and then tested for the next one. If none of the applications elicited a positive response, the overall grade was 0. In that case, the saphenous territory of the same paw was tested as a positive control, which in all cases elicited a positive response. Thermal allodynia was assessed using the Hargreaves apparatus (Ugo Basile, Cat. 37370, IT) as previously described (Hargreaves *et al*, 1988; Deuis *et al*, 2017). Mice were habituated in the Hargreaves apparatus in individual polyvinyl boxes (10 × 10 × 14 cm) placed on glass. A radiant light heat source (45 IR) was focused on the plantar skin of the hind paw, and the latency to a withdrawal response was recorded. The mean time to withdrawal was determined from the average of three tests, separated by at least 2 min. A cut‐off time of 20 s was used to avoid tissue damage. All measurements were conducted by an observer blind to the treatment received for each mouse.

### Nerve processing and axon regeneration analysis

For β‐galactosidase and immunofluorescence (IF) analysis, nerves were fixed by immersion in 4% in PFA. Cellular senescence of fixed nerves was measured by SA‐β‐gal activity (Itahana *et al*, 2007) and documented under stereomicroscope photography. Later, nerves were embedded in OCT (Sakura Finetek), cryostat sections were cut longitudinally at 20 or 10 μm thickness and mounted on Superfrost Plus slides (Thermo Fisher Scientific) for IF on confocal microscopy (Leica TCS SP8) against the markers of senescence: histone y‐H2AX, H3K9me3, HMGB1, lamin‐b1, p16, and P19arf in parallel to SOX‐10 SC marker.

### Immunofluorescence

Nerve samples for cellular labeling and markers of senescence quantification were fresh frozen during embedding in OCT. Ten micrometre cryosections (3–6) were mounted on Superfrost slides Plus®. Sections were fixed in 4% PFA (5 min, RT°), and blocked in 5% donkey serum, 1% BSA, 0.3% Triton X‐100, in PBS. Primary antibodies were incubated in blocking overnight at 4°C. For senescence markers, antibodies against histone y‐H2AX, H3K9me3, HMGB1, lamin‐b1, p16, and P19arf in parallel to SOX‐10 SC marker were used. For axonal regeneration, c‐Jun (regeneration) or Stathmin (axon) antibodies were used in parallel to SOX‐10 SC marker (see Table EV1 for details on clones and dilutions). Secondary antibodies were incubated on 1 h at RT°. Samples were mounted in fluorescent mounting medium (Fluoromount). Images were taken approximately 200–300 μm from the transection site on a Leica SPEII confocal microscope. The quantification and colocalization was performed using the software Oxford Imaris. Fluorophore mean intensity was measured inside the total nuclei volume of all SOX10 stained cells and used to create a frequency distribution graph (Appendix Fig S1). The highest intersection point between control and experimental conditions was used as positivity criteria.

Nerve samples for axonal outgrowth quantification were fixed on 4% PFA for 1 h RT°, washed twice with PBS and then incubated in 5, 10, and 15% sucrose in intervals of 2, 2, and 16 h, respectively, to be finally embedded in OCT. Twenty micrometre cryosections (3–6) were mounted on Superfrost slides Plus®. For immunofluorescence, sections were incubated at 55°C for 20 min, washed in PBS for 15 min RT°, and blocked in 5% gelfish, 0.5% Triton X‐100 in PBS, for 2 h RT°. Primary antibody (SCG10) was incubated overnight at 4°C. Secondary antibodies were incubated for 2 h RT, and mounted in a fluorescent mounting medium (Fluoromount). Images were taken on a Leica SPEII confocal microscope. Merging of the entire manual z‐acquisition of the nerve were merged using ImageJ FIJI Stitching plugin (De Gregorio *et al*, 2018). The reconnection site was recognized and defined in Z‐stack images as the region of attachment of the two nerves of different sizes also marked by the 10‐0 suture used to reconnect both branches. The quantification was performed using the software Oxford Imaris. The number of axons and nerve width were manually quantified every 200 μm starting 200 μm after the reconnection mark (visually recognized as the point of axonal infiltration from the peroneal to the tibial branch) until more than 1,000 μm of nerve was absent of axons, being the sum of all the 200 μm regions with axonal presence the total neurite length. Then, the average value of axons and width was calculated every 1,000 μm, to finally plot the difference between N° of axons and nerve width in an XY line format graph with intervals of 1,000 μm.

### Transcriptomic analysis of denervated nerves

Two months old adult Sprague–Dawley rats underwent sciatic nerve transection at the upper thigh as described previously (Höke *et al*, 2002). The proximal sciatic nerve was sutured to nearby muscles to prevent reinnervation of the distal denervated sciatic nerve. At different time points from 1 to 180 days, the distal denervated and contralateral control uninjured sciatic nerves were harvested and frozen on dry ice for microarray analysis as described previously (Wright *et al*, 2014). Total RNA was extracted, RNA quantity was assessed with Nanodrop Spectrophotometer (Nanodrop Technologies) and quality with the Agilent Bioanalyzer (Agilent Technologies). As per the manufacturer's protocol, 200 ng of total RNA were amplified, biotinylated and hybridized to Illumina Rat Expression Beadchips, querying the expression of 22,000 RefSeq transcripts. Four replicates were run per sample category. Raw data were analyzed by using Bioconductor packages and contrast analysis of differential expression was performed using the LIMMA package (Smyth, 2004). After QC and linear model fitting, a Bayesian estimate of differential expression was calculated and the false discovery rate was set at 5% all as per standard protocols (Coppola, 2011). Differentially expressed genes were contrasted against SASP‐related genes database, using the SASP query tool (www.saspatlas.com).

### Cytokine array

Aged or chronically denervated nerves treated with ABT263 or vehicle were extracted as described above. An inflammatory cytokine array (RD.ARY028, R&D Systems) was used according to manufacturer's instructions to analyze actual concentration of SASP inflammatory proteins found in our analysis.

### Western blot analysis

Sciatic nerves were homogenized in RIPA buffer supplemented with protease inhibitors. Nerve homogenates were incubated in ice for 30 min and centrifuged for 10 min at 9,300 *g* to pellet insoluble material. Protein concentration in the supernatant was determined using the BCA Assay kit (Pierce, Rockford, IL, USA) with BSA as the standard. Aliquots (25–40 μg) were subjected to SDS‐PAGE and transferred onto PVDF membranes. Membranes were blocked in 5% non‐fat milk in TBS‐T and probed against c‐Jun and p16 antibodies at 4°C overnight. After three washes in TBS‐T, HRP‐conjugated secondary antibodies were incubated for 1 h at room temperature, washed three times, and visualized by enhanced chemiluminescence (Pierce, Rockford, IL, USA). Densitometric analysis and quantification were performed using the ImageJ software (NIH, USA).

### Schwann cell cultures

Rat SC were obtained from newborn P2–P4 SD sciatic nerves as previously described (Andersen & Monje, 2018; Monje, 2020), expanded over laminin coating and maintained on DMEM 10% FBS supplemented with 2 μM forskolin and 20 μg/ml bovine pituitary extract (BPE). The medium was replaced every 3 days until the cells reached 90% confluence. Schwann cell purity was assessed by quantifying DAPI^+^/SOX10^+^ cells to the number of total DAPI^+^ nuclei; the purity of primary culture of rat Schwann cells was 99.3% ± 0.2% (mean ± SD, *N* = 3).

Mouse Schwann cells were obtained from ScienCell Research Laboratories (cat number #1701) and cultured as recommended by the manufacturer. Briefly, the plate was coated with 10 μg/ml poly‐L‐lysine overnight. The medium was changed every 2 days, consisting of 5% FBS, 0.1% medium growth supplement (SCGS, Cat #1752), and 1% penicillin/streptomycin solution (cat# 0503).

For the culture of p16‐3MR mouse Schwann cells, nerve teasing was performed on adult animals to collect the nerve fibers, following a previously described protocol (Monje & Kim, 2018). The epineurium layer was removed, and the nerve fibers were incubated overnight with type I collagenase and dispase II for enzymatic dissociation. To halt enzymatic dissociation, FBS/HBSS medium was added, and the cells were recovered by centrifugation at 200 × *g* for 5 min at 4°C. The cells were maintained in DMEM supplemented with 10% FBS, 1% (v/v) 200 mM l‐alanyl‐l‐glutamine dipeptide in 0.85% NaCl, 1% penicillin–streptomycin, and 25 μg/ml gentamicin, along with 20 nM heregulin‐β1177–244 and 2 μM forskolin. Schwann cell purity was assessed by quantifying DAPI^+^/SOX10^+^ cells to the number of total DAPI^+^ nuclei; the purity of primary cultures of mouse Schwann cells was 89.9% ± 2.4% (mean ± SD, *N* = 3).

### Senescence induction of Schwann cell cultures

For senescence induction, cells were seeded at 50,000 cell/cm^2^ in T175 flasks for exosomes isolation or in 24‐well plate for senescence induction and IF. Senescence induction was carried out by adding 80 nM of doxorubicin to SC cultures for 24 h. Afterwards, the growth medium was replaced with doxorubicin‐free medium and maintained for 9 days in order to achieve senescence induced Schwann cells (siSC). Cells were fixed on 4% PFA and senescence determination was made by measuring SA‐β‐gal activity (KAA02 Merck) and IF against the markers of senescence: p16, p21, histone y‐H2AX, HMGB1, lamin‐b1 and P19arf in parallel to S100 SC marker (Table EV1). For senolysis in p16‐3MR mouse SC, the culture was treated with 0.05 mM of ganciclovir for 3 days.

### Rat embryonic dorsal root ganglia (DRG) culture

Dorsal root ganglia explants were prepared as previously described (Andersen & Monje, 2018; Monje, 2020) (Fig EV3). Briefly, DRG were dissected from spinal cord of E13 (mice) or E16 (rat) embryos. DRG were used as explants or reaggregates, explants were seeded in 96‐well plates coated with poly‐L‐lysine/collagen type I and neurobasal medium supplemented with of 1× B27 (50× Gibco), 20 μM L‐glutamine, 1× antibiotic‐antimycotic solution (100× Gibco), 3.75 μM aphidicolin, 1.25 μM 5‐fluoro‐2‐ deoxyuridine, and 50 ng/ml of nerve growth factor (NGF‐2.5s). For reaggregates, the ganglia were incubated in 0.5% trypsin for 5 min at 37°C, then the tissue was disaggregated and suspended on neurobasal medium supplemented as mentioned above. Later, the disaggregated cells were seeded on 96 wells plates filled with 50 μl of 1× agarose solution and agitated on orbital shaker for 15 min at 37°C then incubated over night at 37°C on CO_2_ chamber. Next day, reaggregated DRGs were carefully transferred using a p200 micro pipette and seeded in 96‐well plates coated with poly‐L‐lysine/collagen type I. After 3 days in culture, DRG explants were documented under transmitted light microscopy (Leica DMi8) and then fixed in 4% PFA, images were quantified for neurite growth area using the ImageJ software, immunofluorescence against medium neurofilament (NF‐M) antibody was performed for representative images.

### DRG co‐culture

For direct interaction DRG reaggregates were co‐cultured on 24 wells plates previously seeded with SC or siSC, after 3 days the co‐culture was fixed with 4% PFA for immunofluorescence. For a scheme of the experimental procedure see Fig EV3. Immunofluorescence of co‐cultures was performed against NF‐M and S100 antibodies for quantification of neurite outgrowth area using ImageJ software. Alternatively, DRG reaggregates were treated with conditioned media from SC or siSC (Fig EV3), and the growth cone area was measure after immunofluorescence against acetylated tubulin and phalloidin staining using Image J software.

### Secretome extraction

Conditioned medium (CM) was collected from SC or siSC incubated with exosome‐depleted FBS/phenol red free SC media, exosomes were depleted as described in (De Gregorio *et al*, 2018), see Fig EV3. The media in every condition was extracted and concentrated using 3 kDa filter units (Millipore) for 1 h at 5,000 *g*. Total protein was quantified by BCA protein assay (Pierce Cat N° 23225). For conditioned media assays, 150 μg of concentrated conditioned media protein was added to 250 μl of culture media.

### Mass spectrometric protein analysis of Schwann SASP

The conditioned media with 2% FBS of Schwann cells (repair and Doxo‐treated cells, *n* = 3 each) were collected as previously described (Basisty *et al*, 2020). Briefly, salt and other media components were removed using 3 kDa cutoff columns (Amicon Centrifugal Filters). Highly abundant proteins, including albumin and IgG, were removed using spin columns (High Select™ Depletion Spin Column). Depleted conditioned media were lysed using lysis buffer (5% SDS and 50 mM TEAB). Each extract was reduced by incubation with 20 mM dithiothreitol in 50 mM TEAB for 10 min at 50°C and subsequently alkylated with 40 mM iodoacetamide in 50 mM TEAB for 30 min at RT in the dark. Extracts were acidified to pH < 1 using phosphoric acid (v/v), and 100 mM TEAB in 90% methanol was added. The protein extract was spun through the micro S‐Trap columns (Protifi), washed with 90% methanol in 100 mM TEAB, and then placed in clean elution tubes for trypsin digestion at a 1:25 ratio in trypsin digestion buffer (50 mM TEAB, pH ~8) (protease: protein, wt:wt), at 37°C overnight. Peptides were then sequentially eluted with 50 mM TEAB and 0.5% formic acid and 50% acetonitrile in 0.5% formic acid. Both fractions were pooled together, vacuum‐dried, and re‐suspended in 0.2% formic acid for desalting. The desalted peptides were concentrated and re‐suspended in aqueous 0.2% formic acid for mass spectrometry‐based quantitative analysis.

Mass Spectrometric Data Independent Acquisition (DIA) (Collins *et al*, 2017; Schilling *et al*, 2017) was performed on a Dionex UltiMate 3000 system coupled to an Orbitrap Eclipse Tribrid mass spectrometer (Thermo Fisher Scientific, San Jose, CA) (detailed parameters provided in Table EV2). The solvent system consisted of 2% ACN, 0.1% FA in H2O (solvent A), and 98% ACN, 0.1% FA in H2O (solvent B). Proteolytic peptides (50 ng) were loaded onto an Acclaim PepMap 100 C18 trap column (0.1 × 20 mm, 5 μm particle size; Thermo Fisher Scientific) for 5 min at 5 μl/min with 100% solvent A. Peptides were eluted on an Acclaim PepMap 100 C18 analytical column (75 μm × 50 cm, 3 μm particle size; Thermo Fisher Scientific) at 300 nl/min using the following gradient of solvent B: 2% for 5 min, linear from 2 to 20% in 125 min, linear from 20 to 32% in 40 min, up to 80% in 1 min, 80% for 9 min, down to 2% in 1 min, and 2% for 29 min, for a total gradient length of 210 min.

Schwann cells SASP peptides described above were acquired in DIA mode. Full MS spectra were collected at 120,000 resolution (AGC target: 3e6 ions, maximum injection time: 60 ms, 350–1,650 *m/z*), and MS2 spectra at 30,000 resolution (AGC target: 3e6 ions, maximum injection time: Auto, NCE: 27, fixed first mass 200 *m/z*). The isolation scheme consisted of 26 variable windows covering the 350–1,650 *m/z* range with an overlap of 1 *m/z* (Table EV2) (Bruderer *et al*, 2017).

Data independent acquisition data were processed in Spectronaut v15 (version 15.1.210713.50606; Biognosys) using directDIA. Acquired data were searched against the *homo sapiens* proteome with 42,789 protein entries (UniProtKB‐TrEMBL), accessed on 12/07/2021. Trypsin/P was set as a digestion enzyme, and two missed cleavages were allowed. Cysteine carbamidomethylation was selected as a fixed modification, while methionine oxidation and protein N‐terminus acetylation as variable modifications. The data extraction parameter was set as dynamic. Identification was performed using a 1% precursor and protein *q*‐value (experiment). Quantification was based on the MS2 area, local normalization was applied, and iRT profiling was selected. Differential protein expression analysis was performed using a paired *t*‐test, and *q*‐values were corrected for multiple testing, specifically applying group‐wise testing corrections using the Storey method (Burger, 2018). Dataset EV4 enlists protein groups with at least one unique peptide, *q*‐value < 0.05, and absolute log_2_ (fold‐change) > 0.58 that are considered significantly altered.

### Data plotting and statistics

Data was plotted and analyzed with Graph Pad Prism Software, and plotted data is available in the Sourcedata for each figure. Outlier data was identified with statistical tools of the same software. D'Agostino–Pearson and Shapiro–Wilk normality test were applied to data to be analyzed with parametric statistical tests. Unless is noticed otherwise, data are presented as mean ± SEM. Data analysis was performed as follows, unless is specified otherwise in the corresponding figure caption. For comparing two groups (e.g., vehicle vs. treated mice), a non‐paired, one‐tailed Student's *t*‐test was performed. When more than two groups of data were compared to each other, One‐way ANOVA was performed. If the *P*‐value for ANOVA was significant (*P* < 0.05), a Bonferroni or Fisher's LSD multicomparison post‐test was performed to make comparison between pairs of data.

## Author contributions

**Andrés Fuentes‐Flores:** Formal analysis; investigation; methodology; writing – original draft. **Cristian Geronimo‐Olvera:** Formal analysis; investigation; methodology; writing – original draft. **Karina Girardi:** Formal analysis; investigation; methodology. **David Necuñir‐Ibarra:** Formal analysis; investigation. **Sandip Kumar Patel:** Formal analysis; investigation. **Joanna Bons:** Formal analysis; investigation. **Megan C Wright:** Formal analysis; investigation. **Daniel Geschwind:** Formal analysis; investigation. **Ahmet Hoke:** Conceptualization; resources; supervision; funding acquisition; project administration. **Jose A Gomez‐Sanchez:** Conceptualization; resources; formal analysis; supervision; investigation. **Birgit Schilling:** Conceptualization; formal analysis; supervision; funding acquisition; methodology. **Daniela L Rebolledo:** Conceptualization; formal analysis; supervision; investigation; methodology; writing – review and editing. **Judith Campisi:** Conceptualization; resources; supervision; funding acquisition; validation; project administration. **Felipe A Court:** Conceptualization; formal analysis; supervision; funding acquisition; validation; methodology; writing – original draft; project administration; writing – review and editing.

## Disclosure and competing interests statement

The authors declare that they have no conflict of interest.

## For more information

- Peripheral nerve disorders: https://medlineplus.gov/peripheralnervedisorders.html.
- Peripheral Nerve Society: https://pnsociety.com/.
- American Society for Peripheral Nerve: https://peripheralnerve.org/.
- EANS Section of Peripheral Nerve Neurosurgery: https://www.eans.org/page/peripheral‐nerve‐section.
- American Academy of Physical Medicine and Rehabilitation: https://now.aapmr.org/peripheral‐neurological‐recovery‐and‐regeneration/.

## Supporting information



Appendix

Expanded View Figures PDF

Table EV1

Table EV2

Dataset EV1

Dataset EV2

Dataset EV3

Dataset EV4

Source Data for Expanded View

PDF+

Source Data for Figure 1

Source Data for Figure 2

Source Data for Figure 3

Source Data for Figure 4

Source Data for Figure 5

## Data Availability

Raw data for all the images used in this work are available in the EBI BioStudies database (https://www.ebi.ac.uk/biostudies/) with the accession number S‐BIAD652 (https://www.ebi.ac.uk/biostudies/studies/S‐BIAD652).
