# Supplementary material for: Senescent Schwann cells induced by aging and chronic denervation impair axonal regeneration following peripheral nerve injury
Source: EMBO Mol Med. 2023 Oct 20;15(12):e17907. doi: 10.15252/emmm.202317907 (PMC10701627; doi:10.15252/emmm.202317907)
Supplement: Supplementary file 1 — Appendix [file EMMM-15-e17907-s004.pdf]

Appendix

Table of contents

1. **Appendix Figure S1**
2. **Appendix Table S1**
3. **Appendix Table S2**

Appendix Figure S1

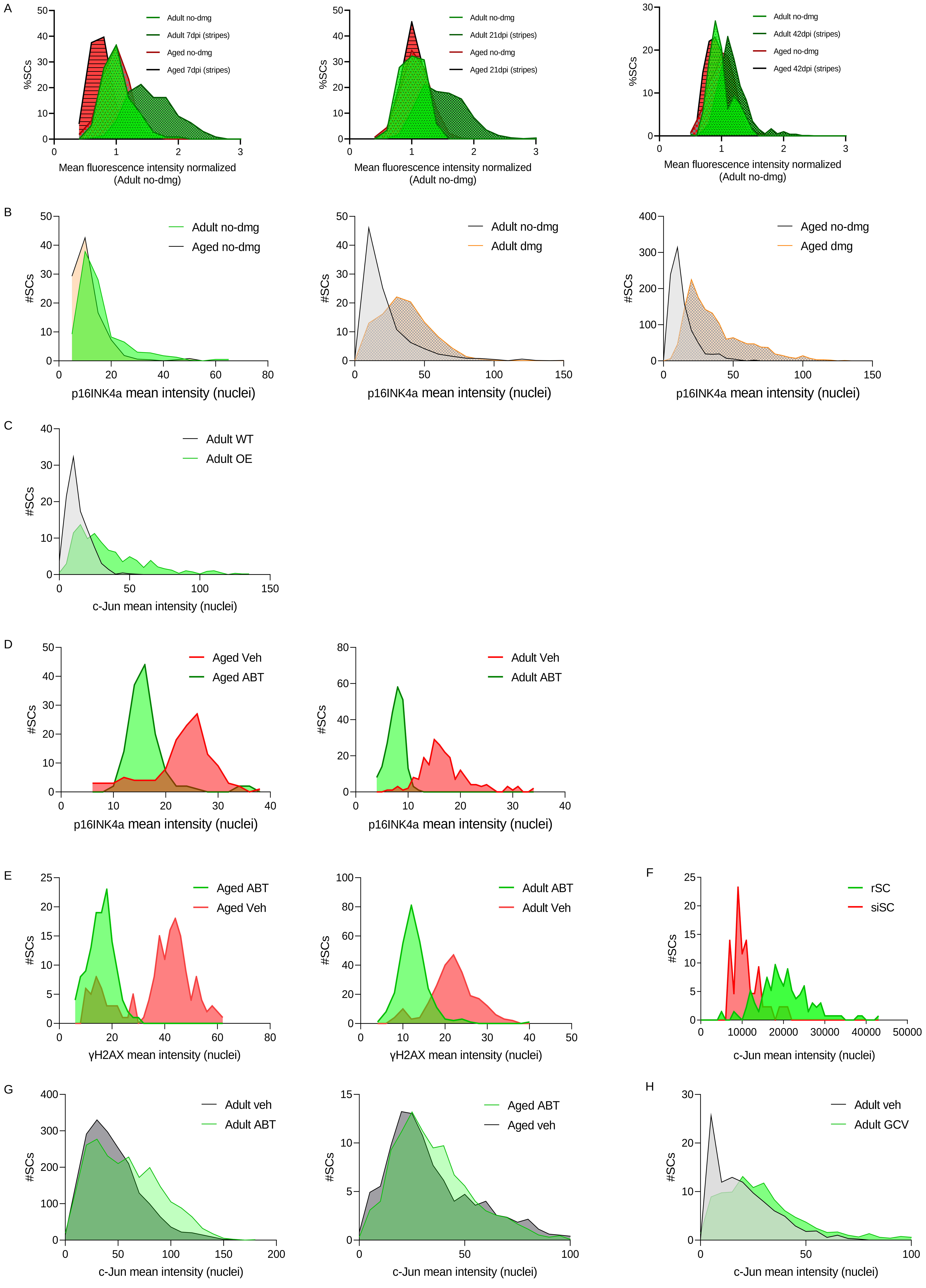

Appendix Figure S1. Frequency distribution of individual cells used in Immunofluorescence assays. (A) c-Jun mean intensity measured inside SC nuclei from Figures 1B and S1B. (B) p16INK4a mean intensity measured inside SC nuclei from Figure 1H (C) p16INK4a mean intensity measured inside SC nuclei from Figure 1N. (D) p16INK4a mean intensity measured inside SC nuclei from Figure 4G. (E) γH2AX mean intensity measured inside SC nuclei from Figure 4E. (F) c-Jun mean intensity measured inside SC nuclei from Figure 3B. (G) c-Jun mean intensity measured inside SC nuclei from Figure 4I. (H) c-Jun mean intensity measured inside SC nuclei from Figure 5H.

Appendix Table S1

Exact p-values for Figure 1D

	Comparing Adult Acu-D vs Aged Acu-D		Comparing Adult Acu-D vs Adult Chr-D	
X axis	*p-value*	*symbol*	*p-value*	*symbol*
1000,000	*0,027*	#	0,092980	
2000,000	*0,001*	###	*<0,000001*	****
3000,000	*0,008*	##	*<0,000001*	****
4000,000	*0,007*	##	*<0,000001*	****
5000,000	*0,038*	#	*0,000003*	****
6000,000	*0,040*	#	*0,000078*	****
7000,000	*0,050*	#	*0,000083*	****
8000,000	0,227		*0,000964*	****
9000,000	0,128		*0,001820*	***
10000,000	0,169		*0,023469*	*
11000,000	0,163		*0,001624*	**
12000,000	0,224		*0,021165*	*
13000,000	0,410		0,098159	
14000,000	0,527		0,347381	
15000,000	0,771		0,591728	
16000,000	0,220		>0,999999	
17000,000	0,117		>0,999999	
18000,000			>0,999999	

Appendix Table S2

Exact p-values for Figure 5

	x-axis	symbol	p-value
Figure 5C	1000,000		0,080342
	2000,000	*	*0,011024*
	3000,000	**	*0,006643*
	4000,000	*	*0,023428*
	5000,000		0,319237
	6000,000		0,460122
	7000,000		0,442312
	8000,000		0,803091
	9000,000		0,931761
Figure 5D	1000,000	*	*0,02091*
	2000,000	***	*0,000573*
	3000,000	****	*0,000037*
	4000,000	****	*0,00006*
	5000,000	****	*0,000075*
	6000,000	****	*0,000643*
	7000,000	**	*0,0013*
	8000,000	*	*0,022196*
	9000,000	*	*0,015835*
	10000,000	**	*0,008232*
	11000,000	***	*0,000462*
	12000,000	**	*0,005001*
	13000,000		0,198894
	14000,000		0,323766
	15000,000		0,50135
	16000,000		0,630578
	17000,000		0,599748
	18000,000		0,935414
Figure 5I	1000,000	**	***0,0014***
	2000,000	**	***0,002***
	3000,000	**	***0,0044***
	4000,000	*	***0,0221***
	5000,000		***0,1558***
	6000,000		
	7000,000		
	8000,000		
	9000,000		
	10000,000		

	x-axis dpr	symbol	p-value
Figure 5E	0,000		>0,999999
	7,000		>0,999999
	14,000		>0,999999
	21,000		0,83609
	28,000		0,43832
	35,000		0,18016
	42,000	****	*0,00007*
	49,000	**	*0,00592*
Figure 5F	7,000		8,078
	14,000	*	*0,032*
	28,000	*	*0,014*
	35,000	*	*0,032*
	42,000		0,082
