# Supplementary material for: Senescent Schwann cells induced by aging and chronic denervation impair axonal regeneration following peripheral nerve injury
Source: EMBO Mol Med. 2023 Oct 20;15(12):e17907. doi: 10.15252/emmm.202317907 (PMC10701627; doi:10.15252/emmm.202317907)
Supplement: Supplementary file 2 — Expanded View Figures PDF [file EMMM-15-e17907-s013.pdf]

Expanded View Figures

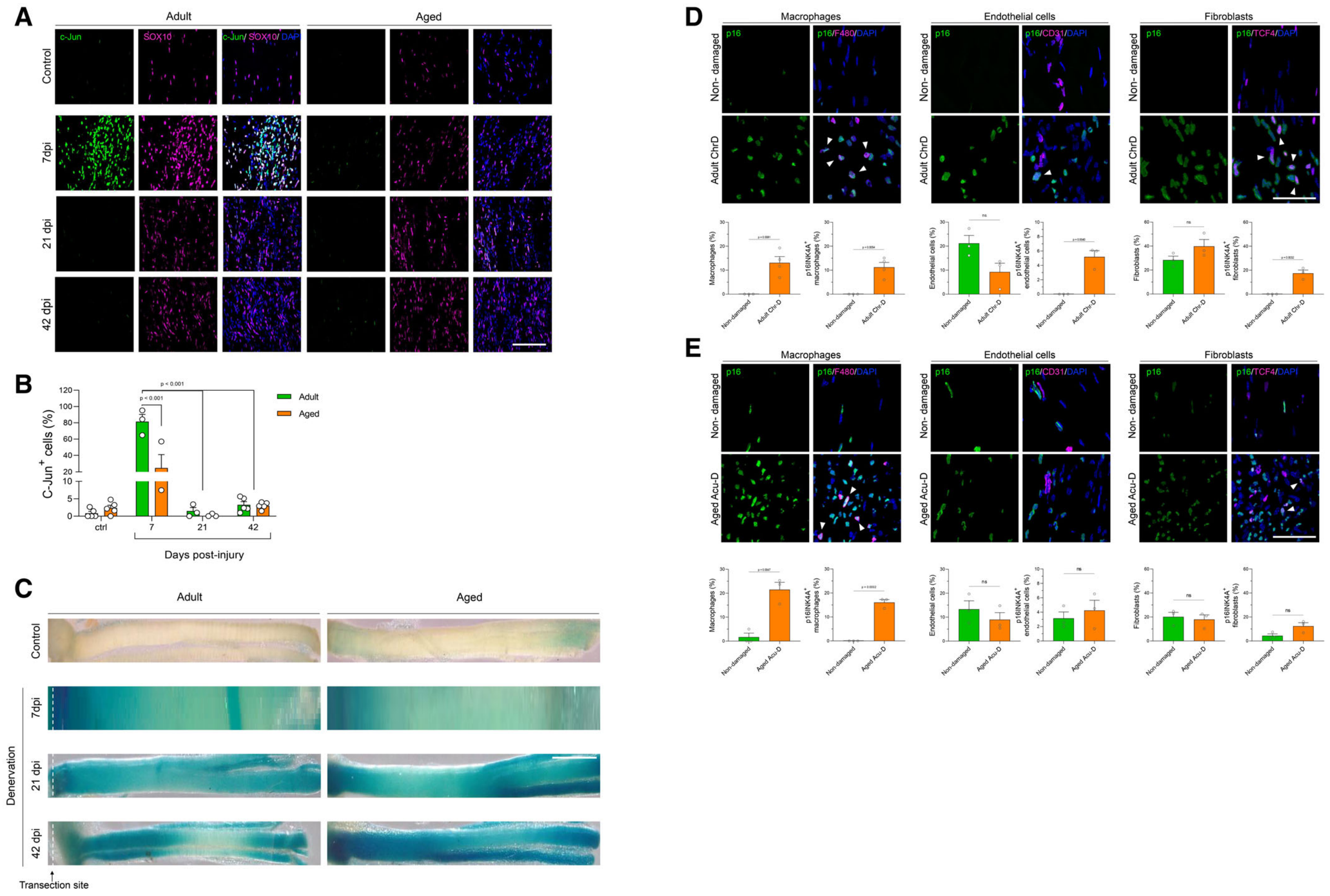

Figure EV1. Sciatic nerves from chronically denervated and aged animals have diminished c-Jun expression and axonal regeneration in SCs after injury compared to adult animals, with changes in macrophages, endothelial cells and fibroblasts.

A, B Representative IF confocal images (SOX10, magenta; c-Jun, red; DAPI, blue) on longitudinal cryostat sections of adult and aged mice sciatic nerves 7, 21 and 42 dpi. The quantification graph shows the % of c-Jun positive nuclei of SCs Scale bar, 100 μM.

C Representative brightfield images of β-galactosidase activity on non-injured nerves, and transected nerves at different times points after damage. Scale bar, 1,000 μM.

D, E Representative IF for $p16^{+}$ (cell senescence marker), $F480^{+}$ (macrophages), $CD31^{+}$ (endothelial cells), and TCF-4^{+} (fibroblasts) cells in longitudinal sections of nerves from chronically denervated in adult mice (D) or acutely denervated in aged mice (E). For each marker, the quantification shows in the left bar the percentage of the specific cell type among total nuclei in the nerve, and the right bar represents the percentage of the specific cell type among the total number of p16-positive senescent cells. This data was used to generate the quantifications in Fig 1J.

Source data are available online for this figure.

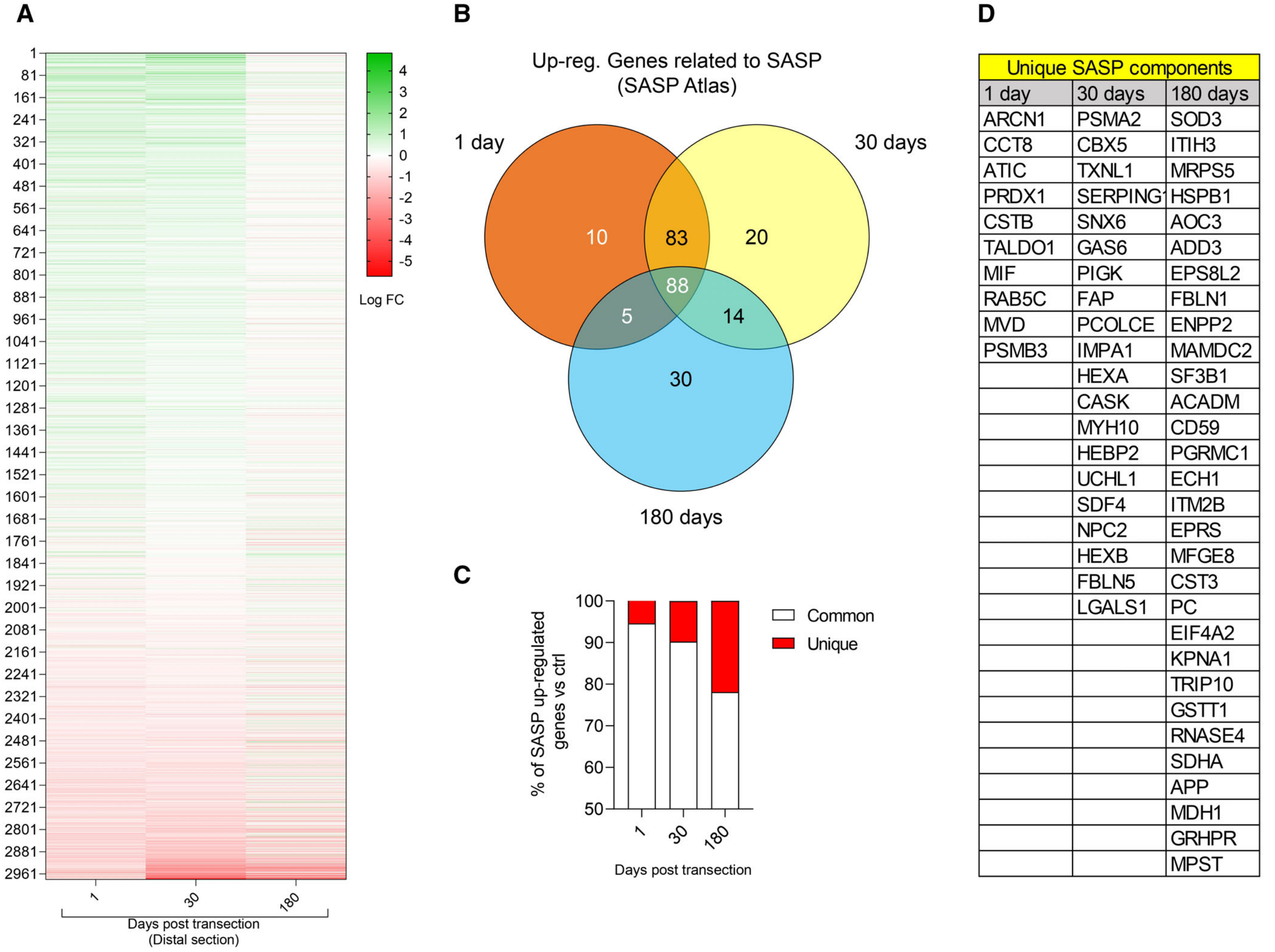

Figure EV2. Differentially expressed genes after chronic denervation and their comparison to senescence-associated secretory phenotype (SASP).

A Differentially expressed genes in adult distal nerves evaluated 1, 30 and 180 days after sciatic nerve transection.
B Venn diagram of up-regulated genes evaluated 1, 30 and 180 days after sciatic nerve transection, contrasted against the SASP-ATLAS database.
C Graph comparison of percentage of unique SASP genes against common SASP genes after 1-, 30- or 180-days post transection.
D Identification of the unique SASP genes at different times post-transection.

Source data are available online for this figure.

Figure EV3. Schematic representation of *in vitro* experiments.

A Scheme of the DRG and SC co-culture protocol.
B Methodological scheme of the obtention of conditioned media from SC and the treatment of DRG with treatment with conditioned media protocol.
C Comparison between DRG re-aggregates after 2 or 3 days *in vitro* (DIV) of exposure to control media, and conditioned media from SC or siSCs in concentrations of 50 or 150 μg of proteins from collected media, compared to untreated DRGs. $N = 3$–4 re-aggregates per group; $^*P < 0.05$ by Student's t-test compared between conditions; error bars indicate SEM.

Source data are available online for this figure.

 © 2023 The Authors

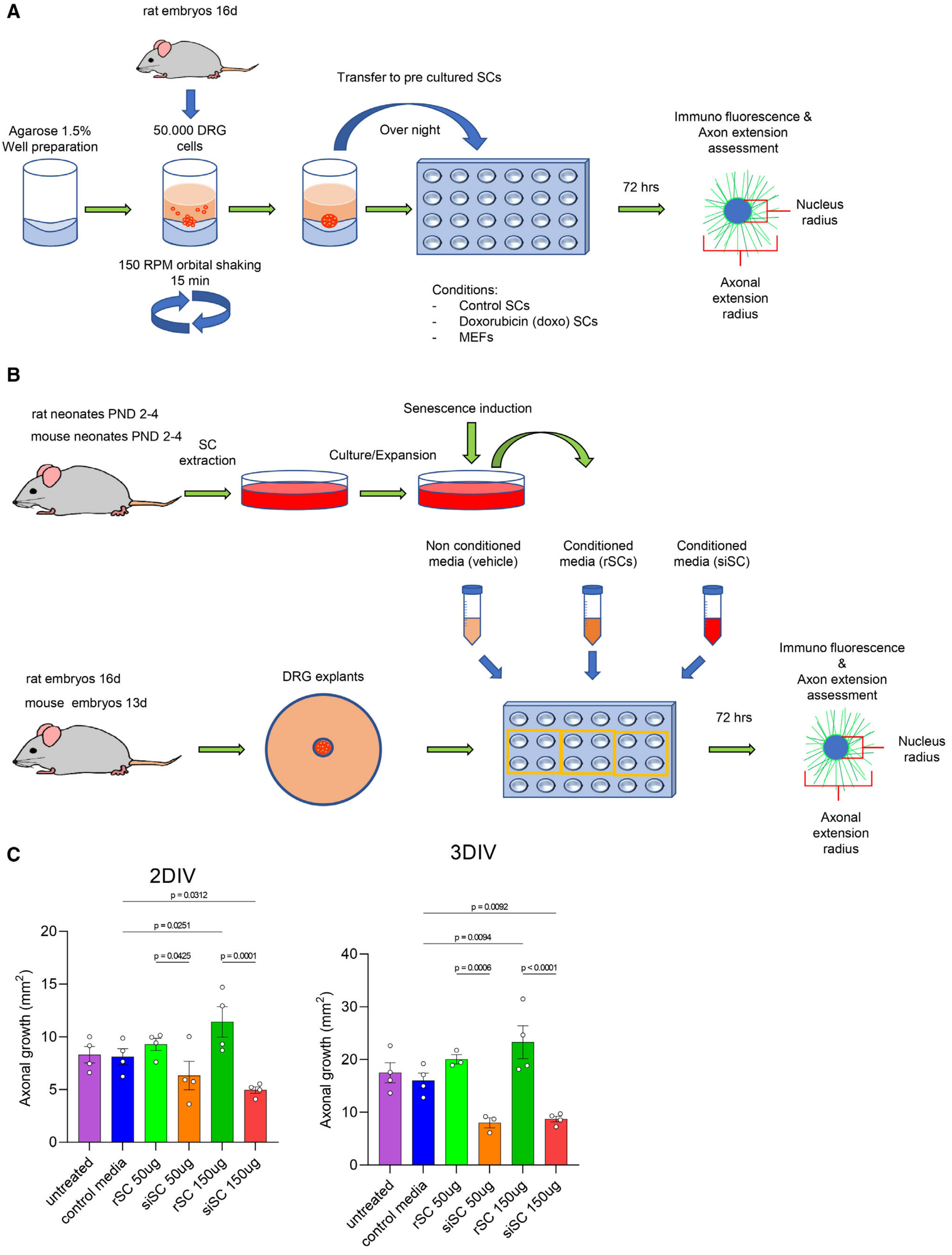
A
rat embryos 16d
Transfer to pre cultured SCs
Agarose 1.5%
Well preparation
50.000 DRG
cells
Over night
Immuno fluorescence &
Axon extension
assessment
72 hrs
Nucleus
radius
150 RPM orbital shaking
15 min
Conditions:
- Control SCs
- Doxorubicin (doxo) SCs
- MEFs
Axonal
extension
radius
B
rat neonates PND 2-4
mouse neonates PND 2-4
SC
extraction
Senescence induction
Culture/Expansion
Non conditioned
media (vehicle)
Conditioned
media (rSCs)
Conditioned
media (siSC)
Immuno fluorescence
&
Axon extension
assessment
rat embryos 16d
mouse embryos 13d
DRG explants
72 hrs
Nucleus
radius
Axonal
extension
radius
C
2DIV
3DIV
p = 0.0312
p = 0.0251
p = 0.0425
p = 0.0001
p = 0.0092
p = 0.0094
p = 0.0006
p < 0.0001
Axonal growth (mm2)
untreated
control media
rSC 50ug
siSC 50ug
rSC 150ug
siSC 150ug

Figure EV3.

Figure EV4. Markers of senescence in rat primary Schwann cell culture after doxorubicin treatment.

A Representative IF images in rSC and siSC, S100, green, βgal, black; Hmgb-1/yH2AX, red; DAPI, blue. Scale bar, βgal, 100 μm; p21, 100 μm; yH2AX, 50 μm; hmbgb-1, 25 μm; laminb1, 25 μm.

B–I Graph comparison of β-gal^{+} cells (B), Mander's co-localization index of HMGB1 (C), yH2AX foci/nucleus (D), p21 positive cells (E) and expression levels (F), nuclei positive for LaminB1 marked invaginations (G) and expression (mean intensity) (H), p16INK4a fold change (qRT-PCR) (I) between non-senescent and siSCs and rSCs. $N = 3$–6 per condition; $^{*}P < 0.05$, $^{**}P < 0.01$ by Student's t-test compared between conditions; error bars indicate SEM.

Source data are available online for this figure.

 © 2023 The Authors

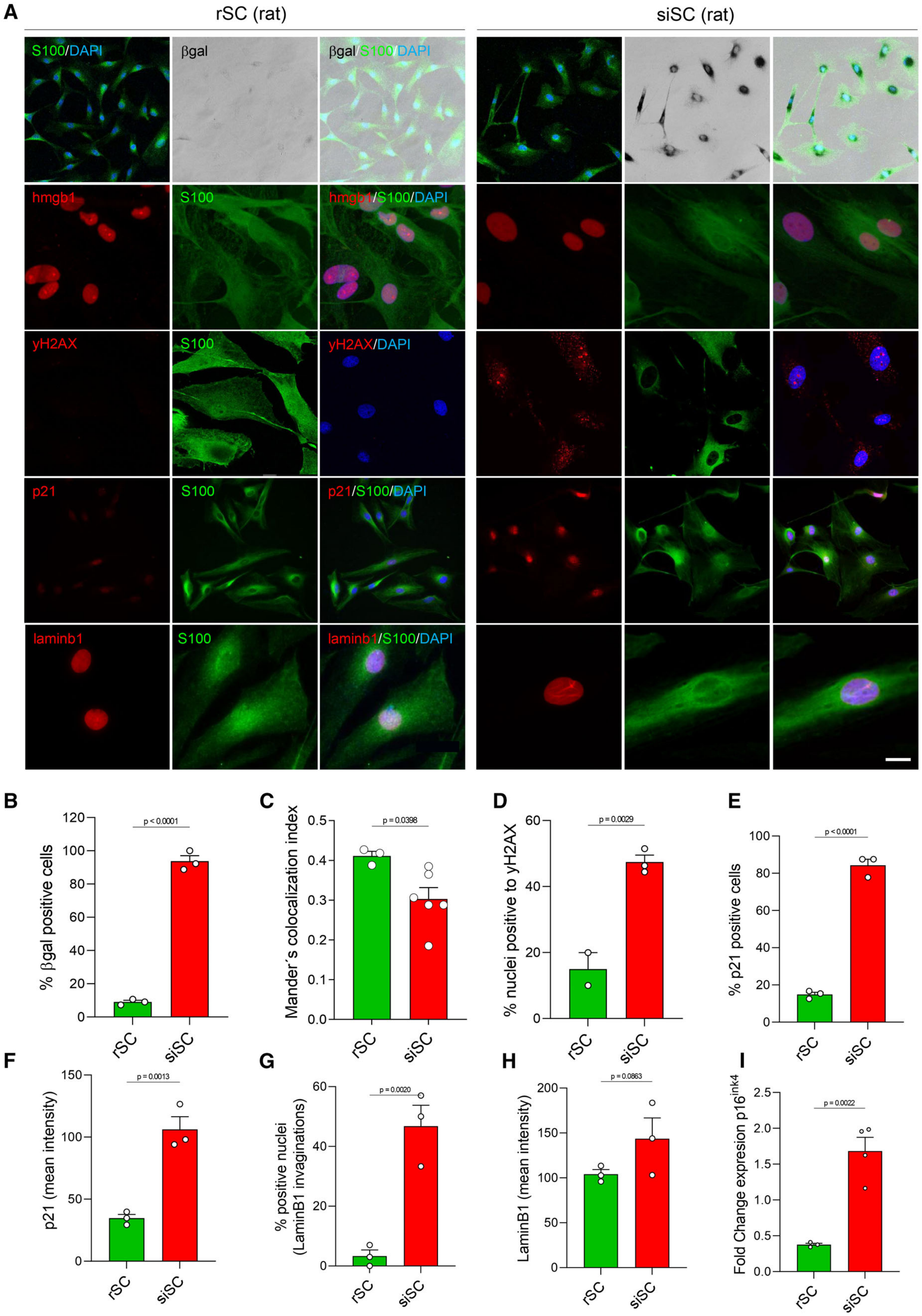
A
rSC (rat)
siSC (rat)
S100/DAPI
βgal
βgal/S100/DAPI
hmgb1
S100
hmgb1/S100/DAPI
yH2AX
S100
yH2AX/DAPI
p21
S100
p21/S100/DAPI
laminb1
S100
laminb1/S100/DAPI
B
% βgal positive cells
p < 0.0001
rSC
siSC
C
Mander´s colocalization index
p = 0.0398
D
% nuclei positive to yH2AX
p = 0.0029
E
% p21 positive cells
p < 0.0001
F
p21 (mean intensity)
p = 0.0013
G
% positive nuclei (LaminB1 invaginations)
p = 0.0020
H
LaminB1 (mean intensity)
p = 0.0863
I
Fold Change expresion p16ink4
p = 0.0022

Figure EV4.

Figure EV5. Markers of senescence in mouse primary Schwann cell culture after doxorubicin treatment. Mouse siSC impairs c-Jun expression and neurite outgrowth in vitro.

A Representative IF images of mouse rSC and siSC stained with different senescent markers (SA-β-gal, p16) or c-Jun together with the SC marker S100 and the nuclei marker DAPI. Scale bar, 50 μm.

B–D Graph comparison of β-gal positive cells (B), c-Jun nuclei intensity (C) and % of p16 positive cells (D). $N = 3$ per condition; *$P < 0.05$, **$P < 0.01$ by Student's *t*-test compared between conditions; error bars indicate SEM.

E Representative IF images of mouse DRG neurons. In (A) DRG were treated for 72 h with conditioned media derived from control, rSC, and siSC. Scale bar, 500 μm. To the right, the graph shows the quantification of axonal growth of DRG neurons comparing the different treatments.

F Higher magnification images of axonal growth cones present at the tip of the explants from DRGs treated in (A). Scale bars, 50 μm. To the right, the graph shows the quantification of the size of the growth cone between treatments. $N = 3$ for each condition. One-way ANOVA with Bonferroni multicomparison post-test. *$P < 0.05$, **$P < 0.01$, ***$P < 0.001$, ****$P < 0.0001$. Data is presented as mean ± SEM.

G Primary cultures of SC from p16-3MR mice were treated with doxorubicin to induce senescence (see Materials and Methods for details). After senescence induction (DOXO), red fluorescent protein (RFP) is robustly expressed compared to vehicle-treated Schwann cells. Ganciclovir (GCV) treatment eliminates most RFP-expressing siSC. Scale bar, 200 μm.

H Basal expression of c-Jun by Western blot in undamaged sciatic nerves from wild type and p16-3MR mice. Besides the already low expression of c-Jun in undamaged nerves in wild type mice compared to injured nerves (adult Acu-D), the levels of c-Jun are comparable between p16-3MR and wild type mice.

Source data are available online for this figure.

© 2023 The Authors

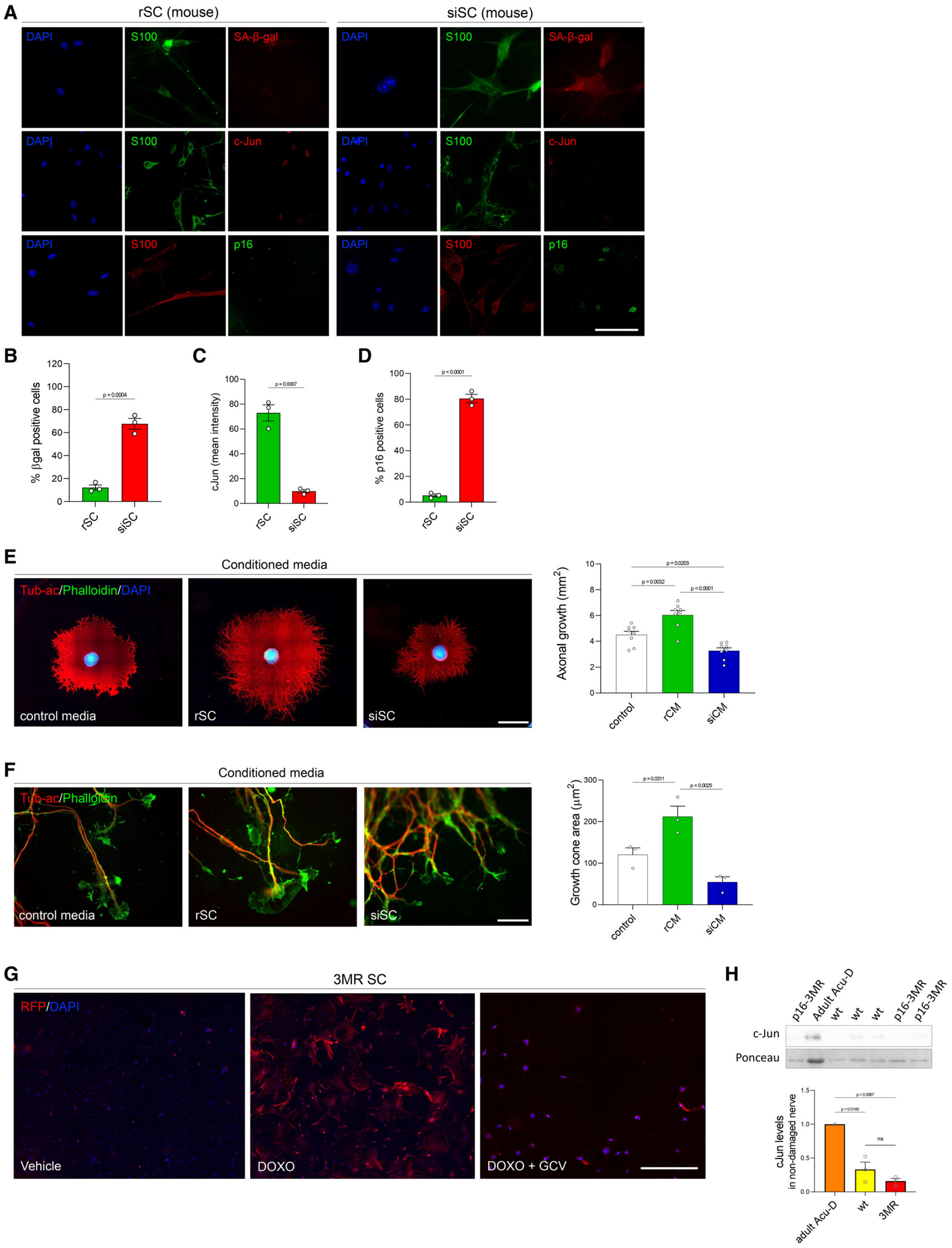
A
rSC (mouse)
siSC (mouse)
DAPI
S100
SA-β-gal
c-Jun
p16
B
% βgal positive cells
p = 0.0004
rSC
siSC
C
cJun (mean intensity)
p = 0.0007
D
% p16 positive cells
p < 0.0001
E
Conditioned media
Tub-ac/Phalloidin/DAPI
control media
rSC
siSC
Axonal growth (mm2)
p = 0.0203
p = 0.0032
p < 0.0001
control
rCM
siCM
F
Conditioned media
Tub-ac/Phalloidin
Growth cone area (μm2)
p = 0.0311
p = 0.0025
G
3MR SC
RFP/DAPI
Vehicle
DOXO
DOXO + GCV
H
p16-3MR
Adult Acu-D
wt
p16-3MR
c-Jun
Ponceau
cJun levels in non-damaged nerve
ns
adult Acu-D
3MR

Figure EV5.
