# Supplementary figures and images for: Senescent Schwann cells induced by aging and chronic denervation impair axonal regeneration following peripheral nerve injury (part 1 of 3)
Source: EMBO Mol Med. 2023 Oct 20;15(12):e17907. doi: 10.15252/emmm.202317907 (PMC10701627; doi:10.15252/emmm.202317907)

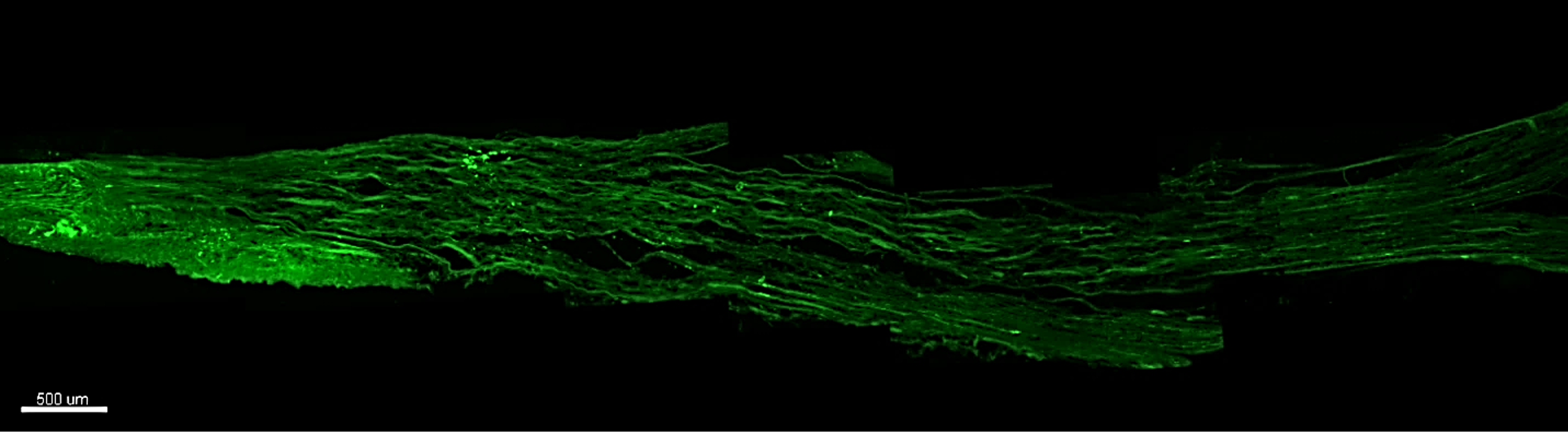

Supplement: Supplementary file 11 — Source Data for Figure 1 [file EMMM-15-e17907-s007.zip › SourceData_Fig_1/Fig_1_Source_Data__images/1D/ADULT.tif]

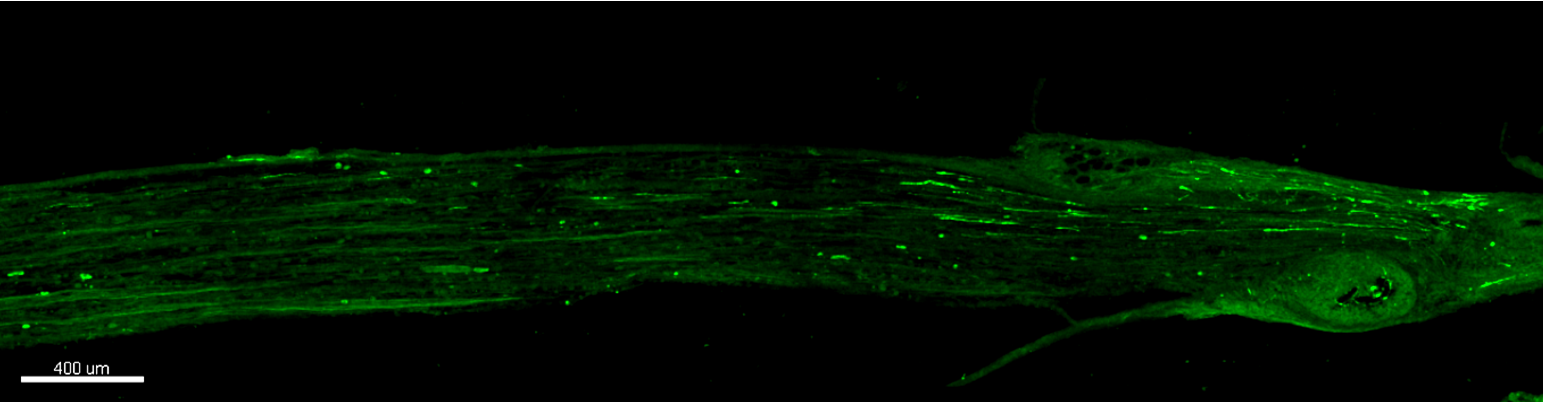

Supplement: Supplementary file 11 — Source Data for Figure 1 [file EMMM-15-e17907-s007.zip › SourceData_Fig_1/Fig_1_Source_Data__images/1D/ADULT_CHRONIC.tif]

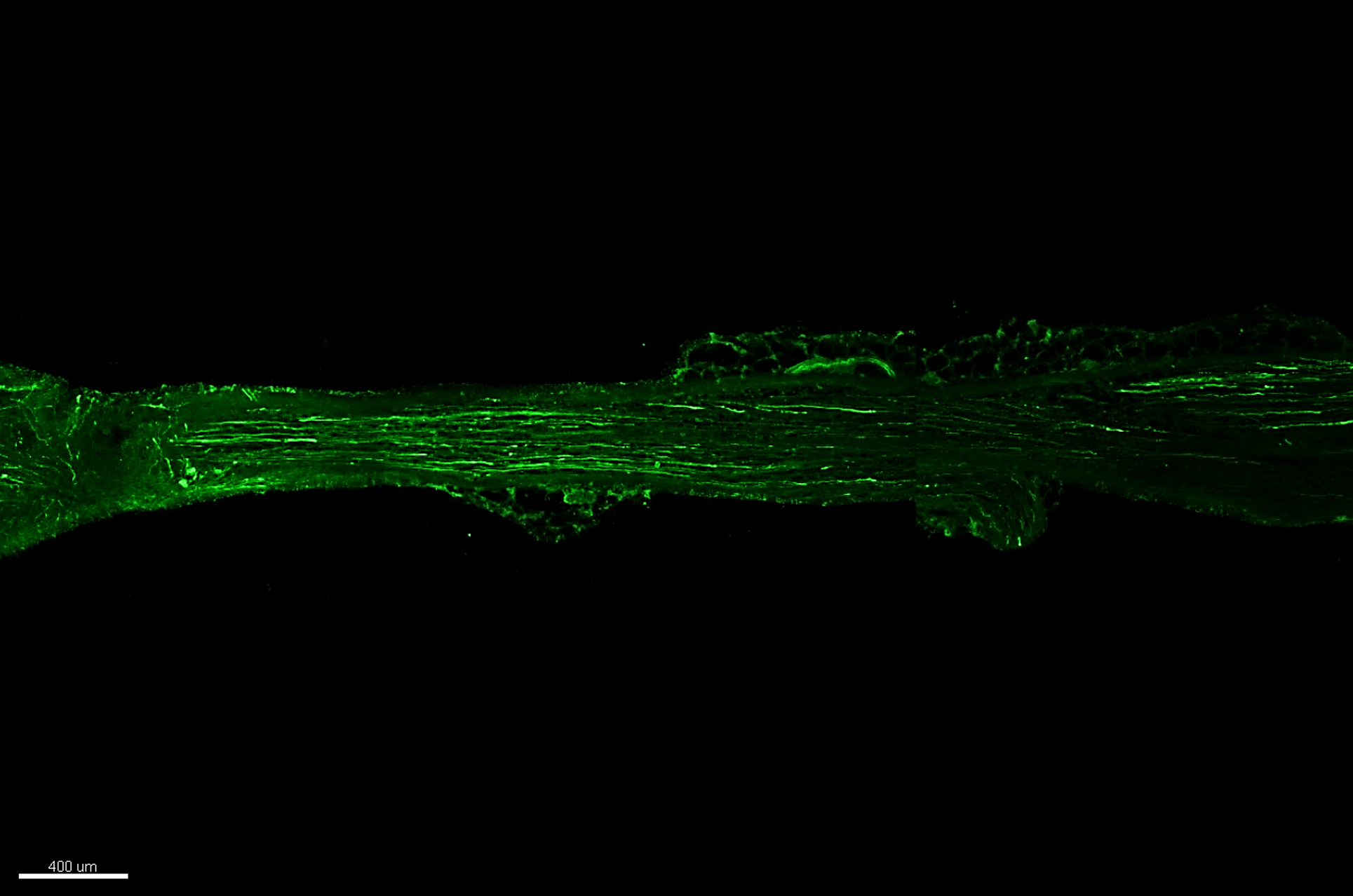

Supplement: Supplementary file 11 — Source Data for Figure 1 [file EMMM-15-e17907-s007.zip › SourceData_Fig_1/Fig_1_Source_Data__images/1D/AGED.tif]

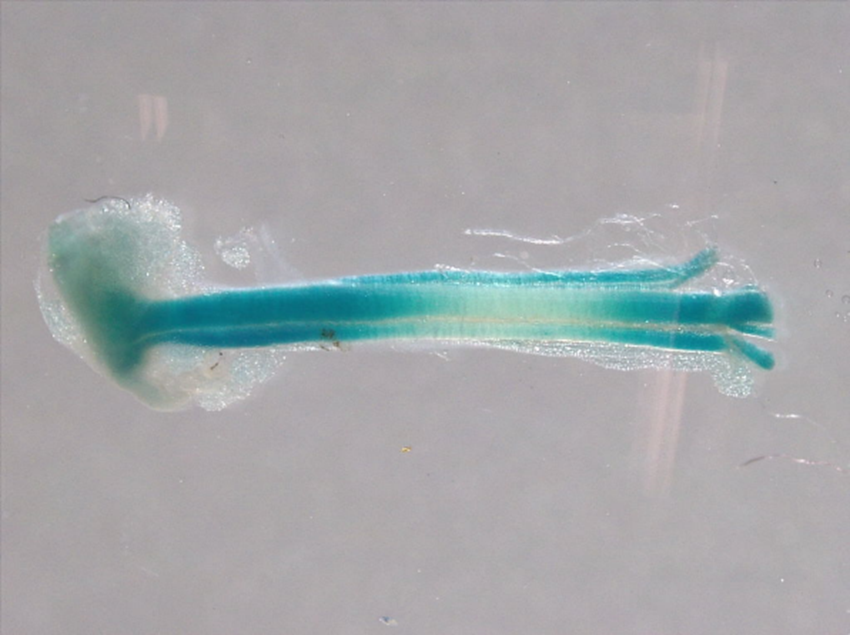

Supplement: Supplementary file 11 — Source Data for Figure 1 [file EMMM-15-e17907-s007.zip › SourceData_Fig_1/Fig_1_Source_Data__images/1E/Brightfield/adult_42dpi.png]

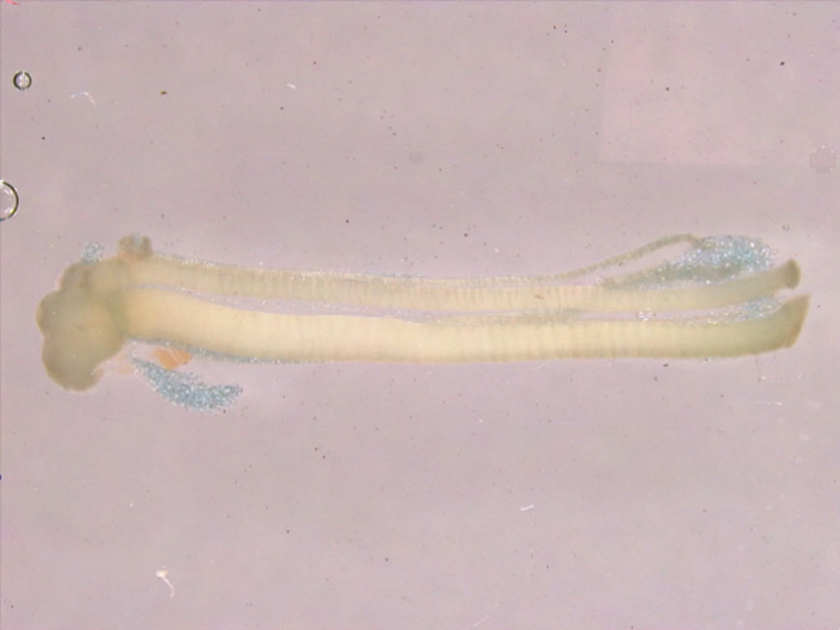

Supplement: Supplementary file 11 — Source Data for Figure 1 [file EMMM-15-e17907-s007.zip › SourceData_Fig_1/Fig_1_Source_Data__images/1E/Brightfield/adult_ctrl.png]

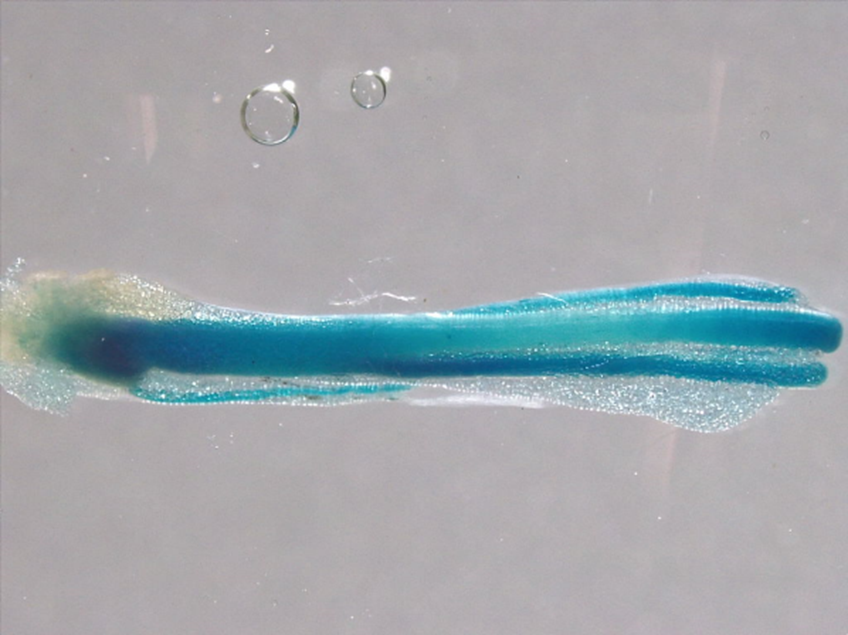

Supplement: Supplementary file 11 — Source Data for Figure 1 [file EMMM-15-e17907-s007.zip › SourceData_Fig_1/Fig_1_Source_Data__images/1E/Brightfield/aged_42dpi.png]

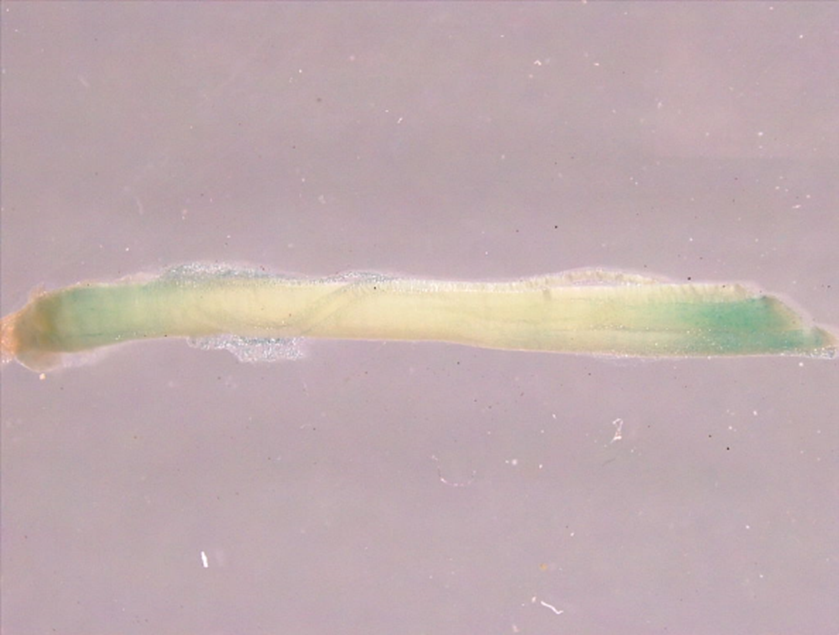

Supplement: Supplementary file 11 — Source Data for Figure 1 [file EMMM-15-e17907-s007.zip › SourceData_Fig_1/Fig_1_Source_Data__images/1E/Brightfield/aged_ctrl.png]

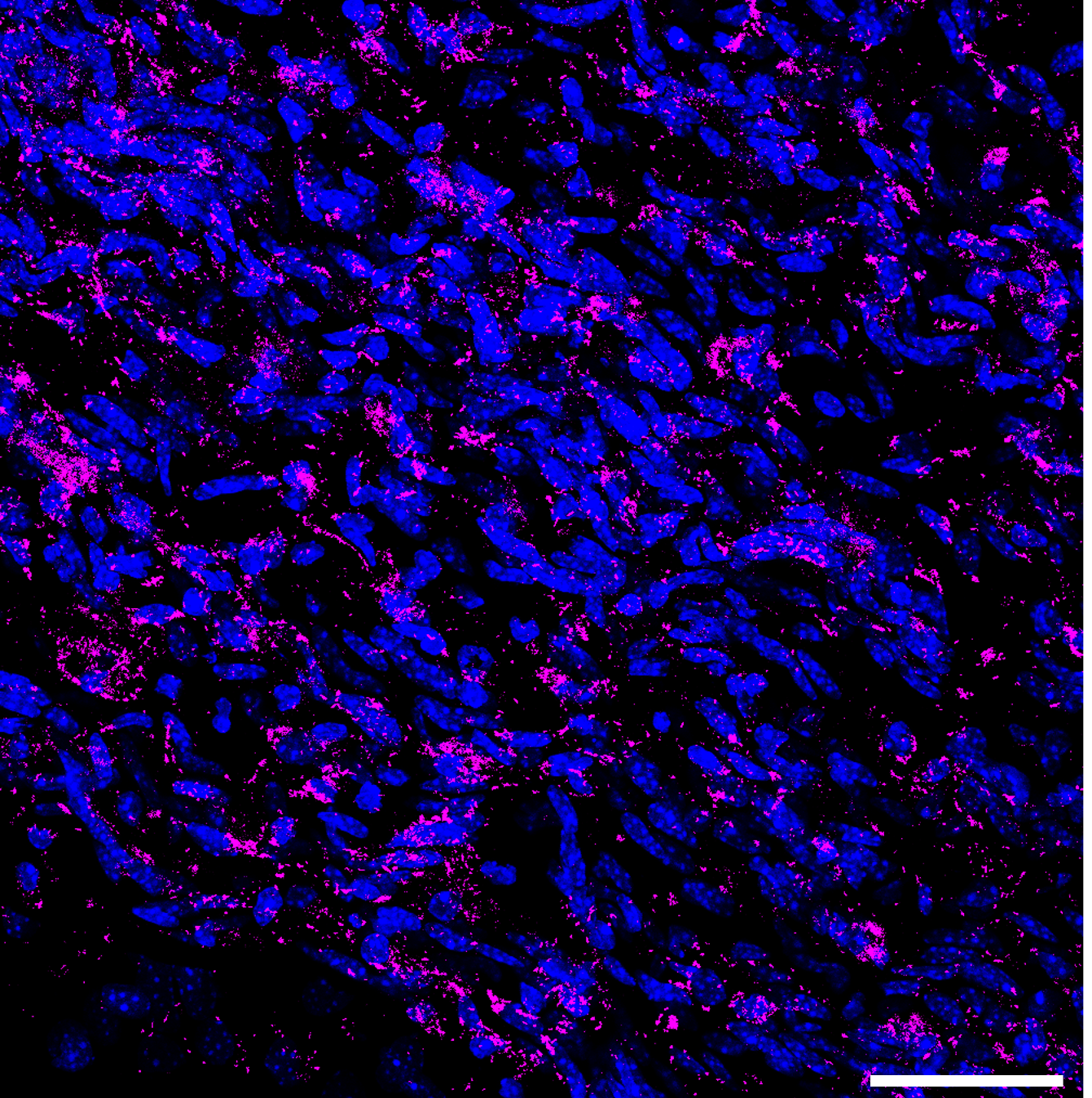

Supplement: Supplementary file 11 — Source Data for Figure 1 [file EMMM-15-e17907-s007.zip › SourceData_Fig_1/Fig_1_Source_Data__images/1E/SA-B-gal/Adult_chronic.tif]

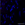

Supplement: Supplementary file 11 — Source Data for Figure 1 [file EMMM-15-e17907-s007.zip › SourceData_Fig_1/Fig_1_Source_Data__images/1E/SA-B-gal/Adult_no_dmg.tif]

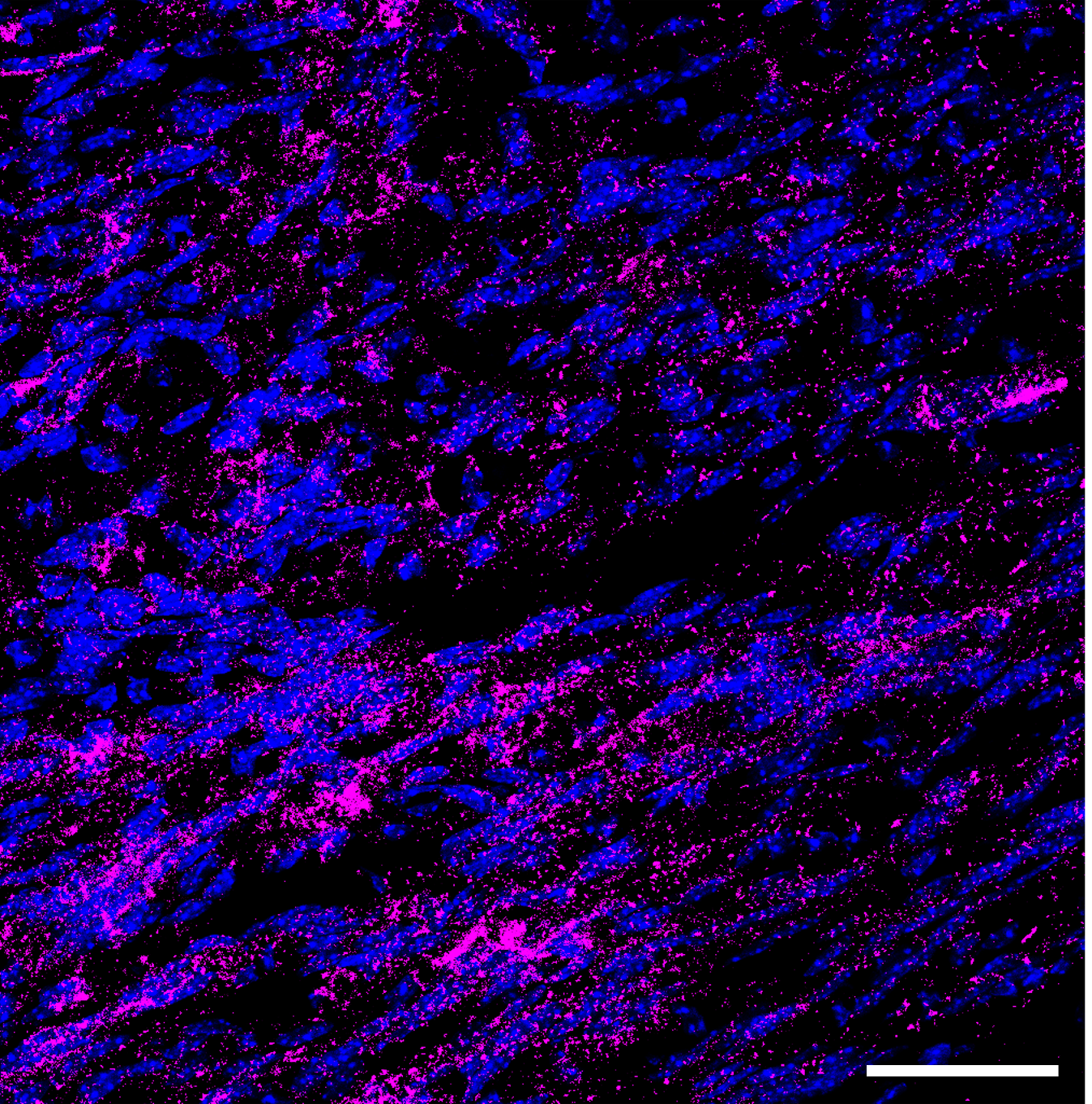

Supplement: Supplementary file 11 — Source Data for Figure 1 [file EMMM-15-e17907-s007.zip › SourceData_Fig_1/Fig_1_Source_Data__images/1E/SA-B-gal/Aged_chronic.tif]

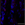

Supplement: Supplementary file 11 — Source Data for Figure 1 [file EMMM-15-e17907-s007.zip › SourceData_Fig_1/Fig_1_Source_Data__images/1E/SA-B-gal/Aged_no_dmg.tif]

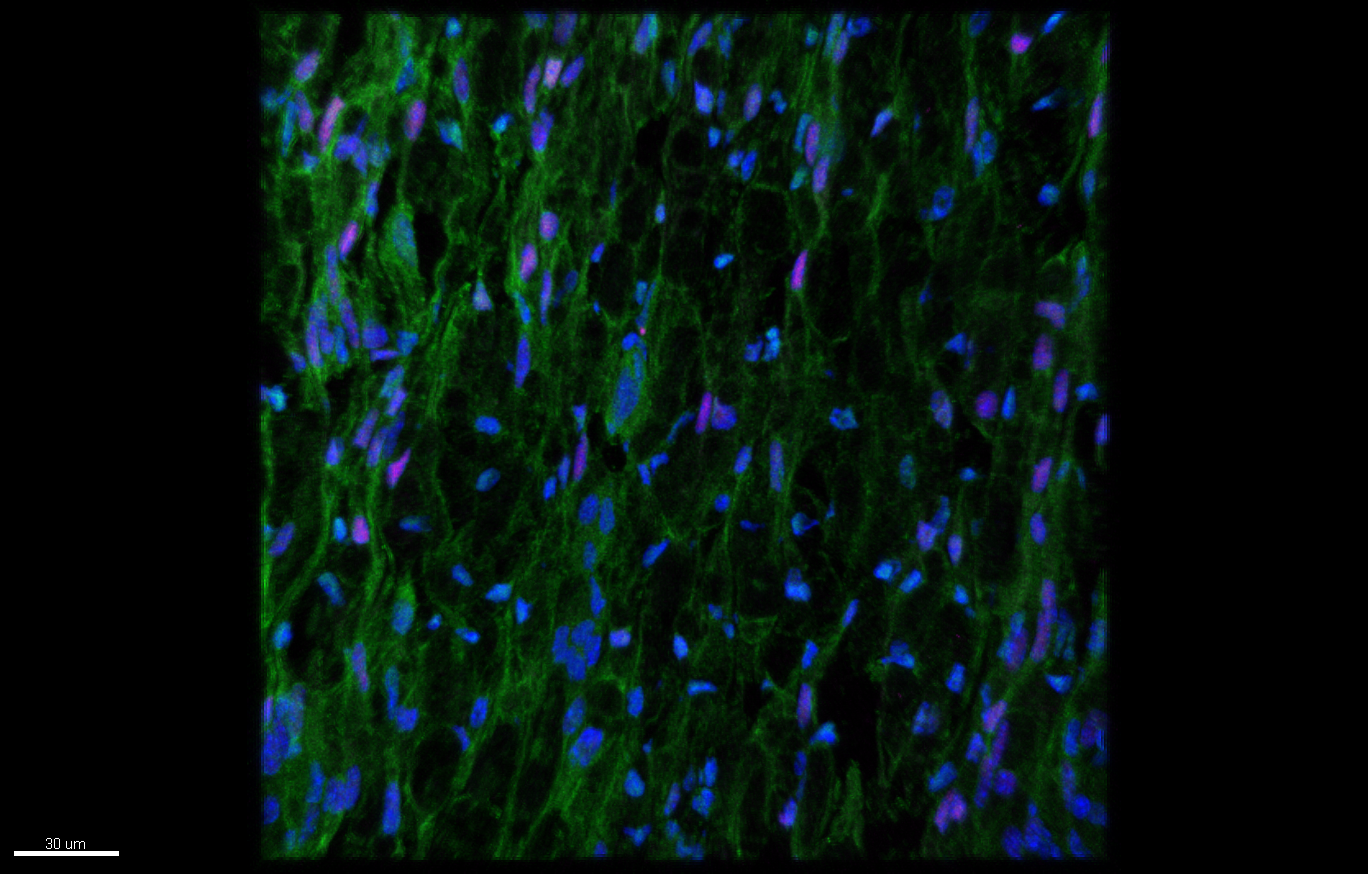

Supplement: Supplementary file 11 — Source Data for Figure 1 [file EMMM-15-e17907-s007.zip › SourceData_Fig_1/Fig_1_Source_Data__images/1F/adult_dmg_1/Adult_I_dmg_p16_2023-02-06T13-54-32.334.tif]

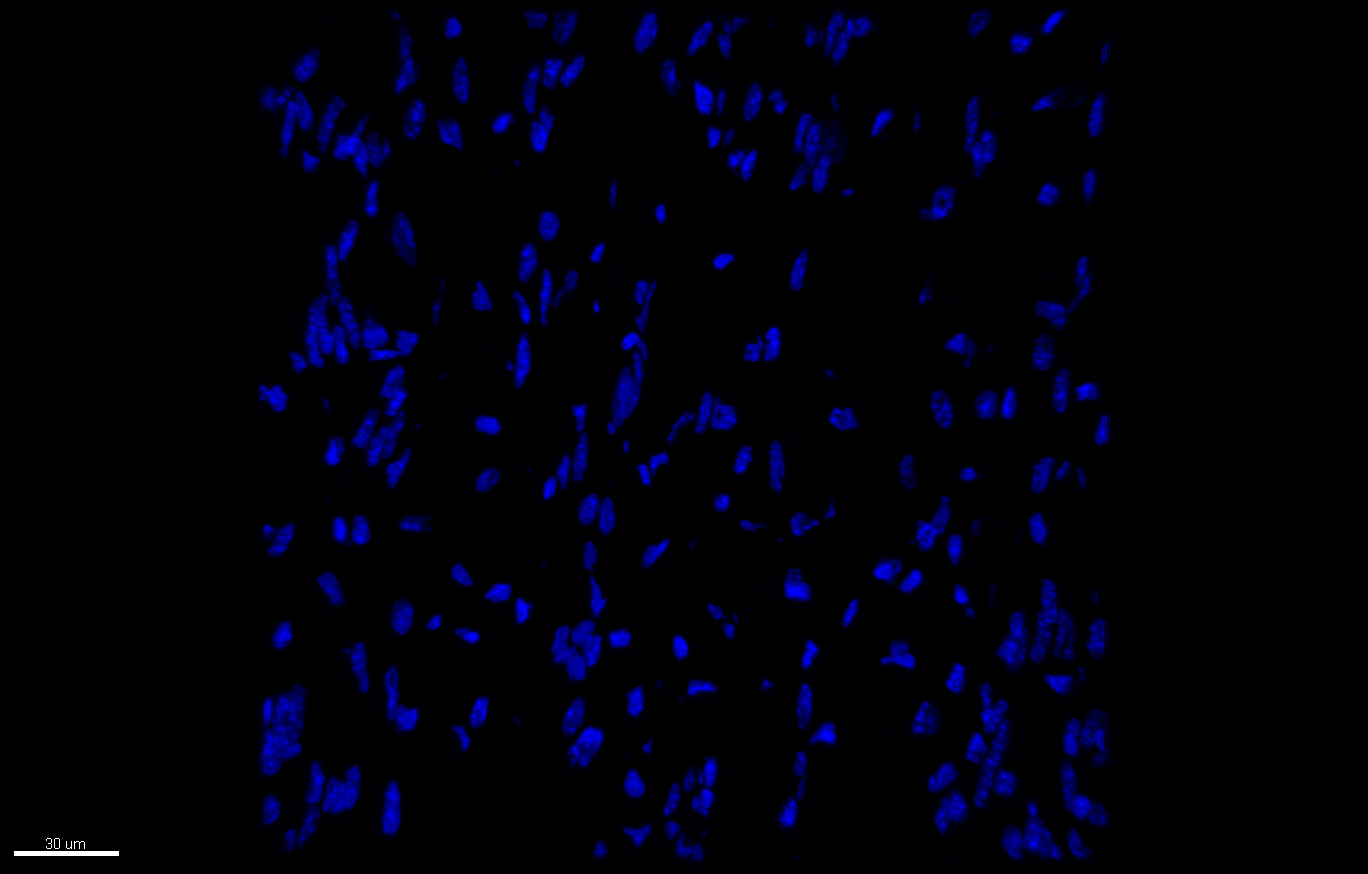

Supplement: Supplementary file 11 — Source Data for Figure 1 [file EMMM-15-e17907-s007.zip › SourceData_Fig_1/Fig_1_Source_Data__images/1F/adult_dmg_1/Adult_I_dmg_p16_2023-02-06T13-54-38.690.tif]

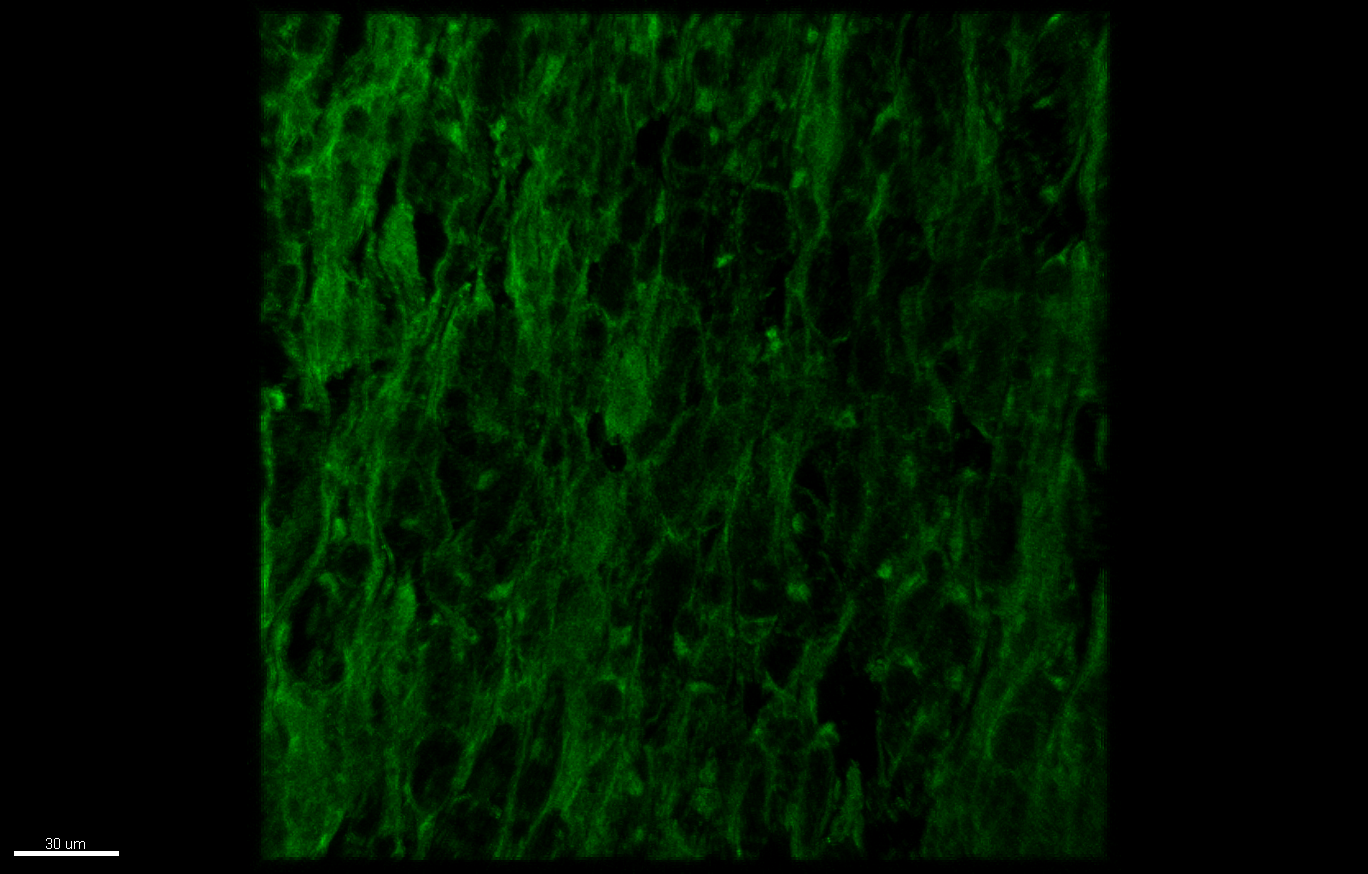

Supplement: Supplementary file 11 — Source Data for Figure 1 [file EMMM-15-e17907-s007.zip › SourceData_Fig_1/Fig_1_Source_Data__images/1F/adult_dmg_1/Adult_I_dmg_p16_2023-02-06T13-54-45.368.tif]

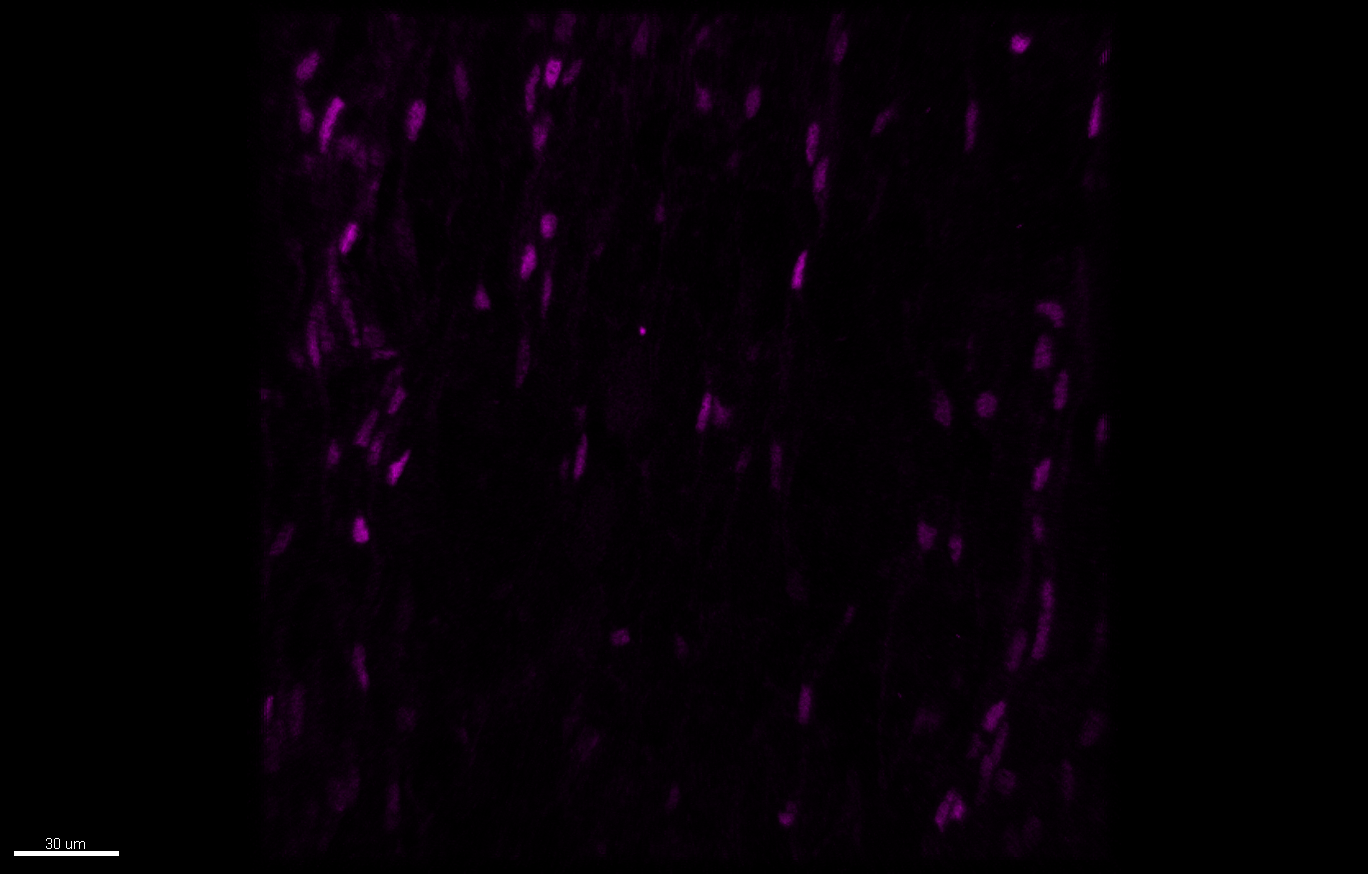

Supplement: Supplementary file 11 — Source Data for Figure 1 [file EMMM-15-e17907-s007.zip › SourceData_Fig_1/Fig_1_Source_Data__images/1F/adult_dmg_1/Adult_I_dmg_p16_2023-02-06T13-54-51.650.tif]

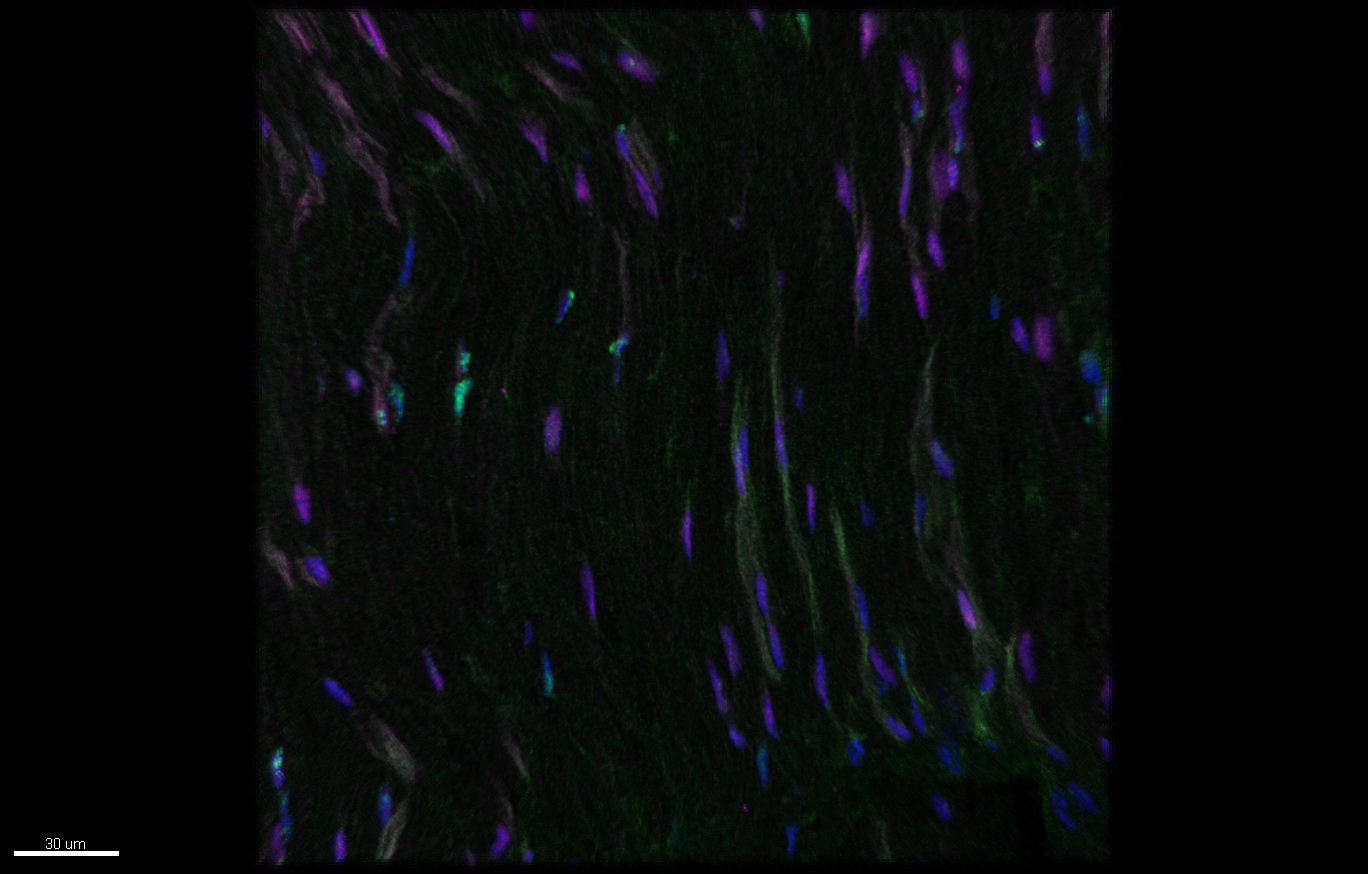

Supplement: Supplementary file 11 — Source Data for Figure 1 [file EMMM-15-e17907-s007.zip › SourceData_Fig_1/Fig_1_Source_Data__images/1F/adult_no_dmg_1/real_Adult_I_nodmg_p16_2023-02-06T13-49-52.902.tif]

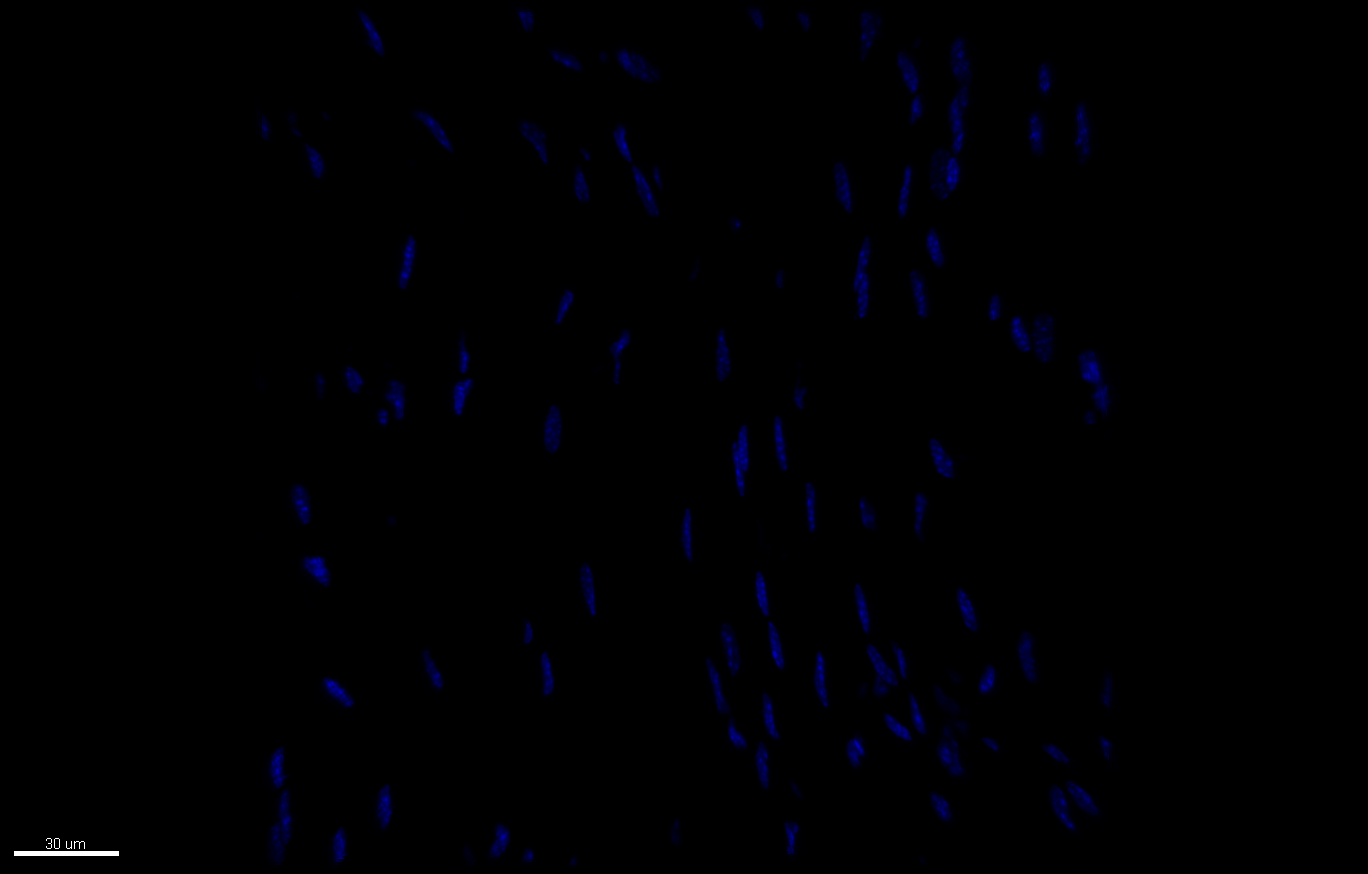

Supplement: Supplementary file 11 — Source Data for Figure 1 [file EMMM-15-e17907-s007.zip › SourceData_Fig_1/Fig_1_Source_Data__images/1F/adult_no_dmg_1/real_Adult_I_nodmg_p16_2023-02-06T13-50-03.519.tif]

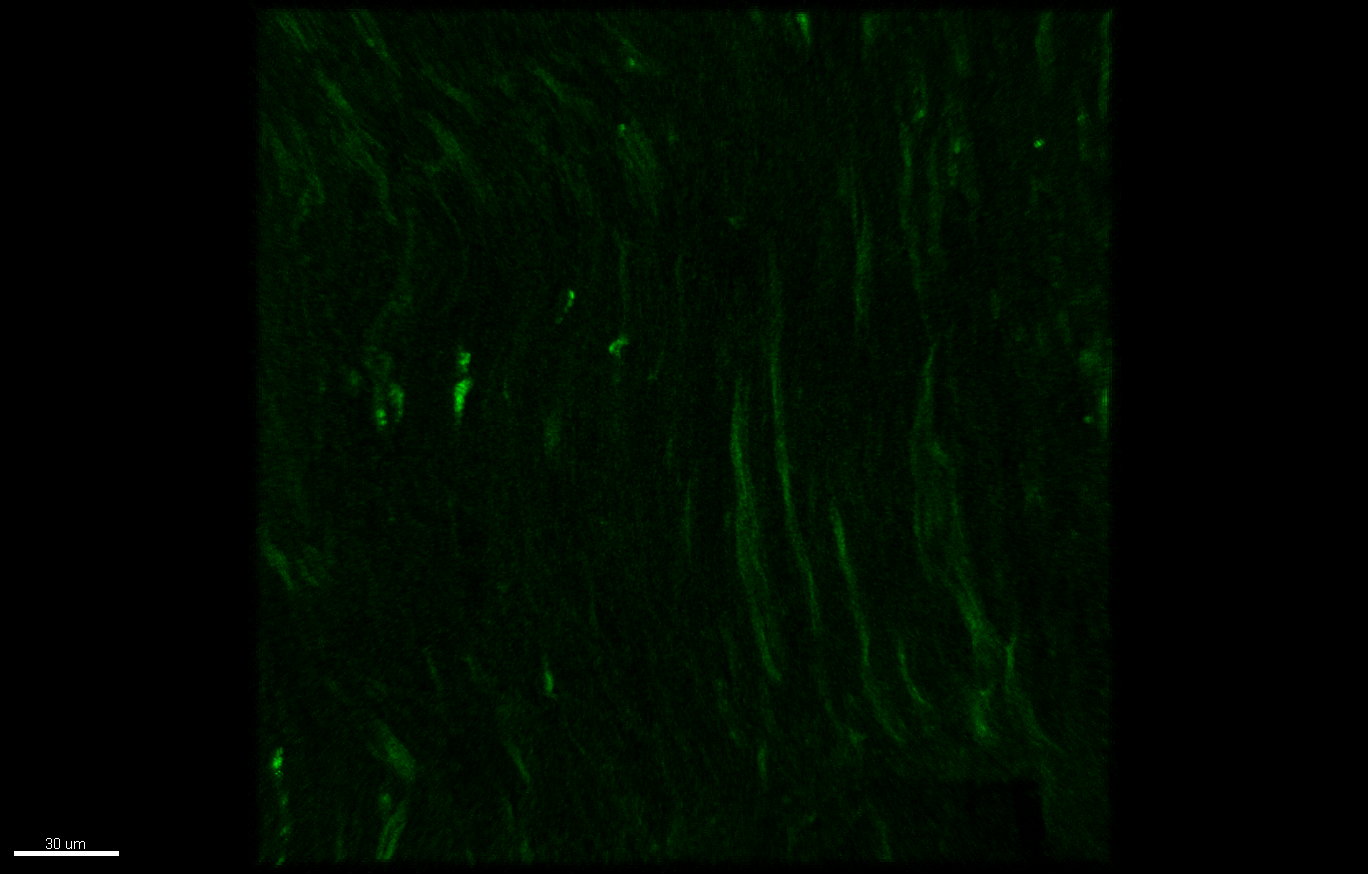

Supplement: Supplementary file 11 — Source Data for Figure 1 [file EMMM-15-e17907-s007.zip › SourceData_Fig_1/Fig_1_Source_Data__images/1F/adult_no_dmg_1/real_Adult_I_nodmg_p16_2023-02-06T13-50-09.608.tif]

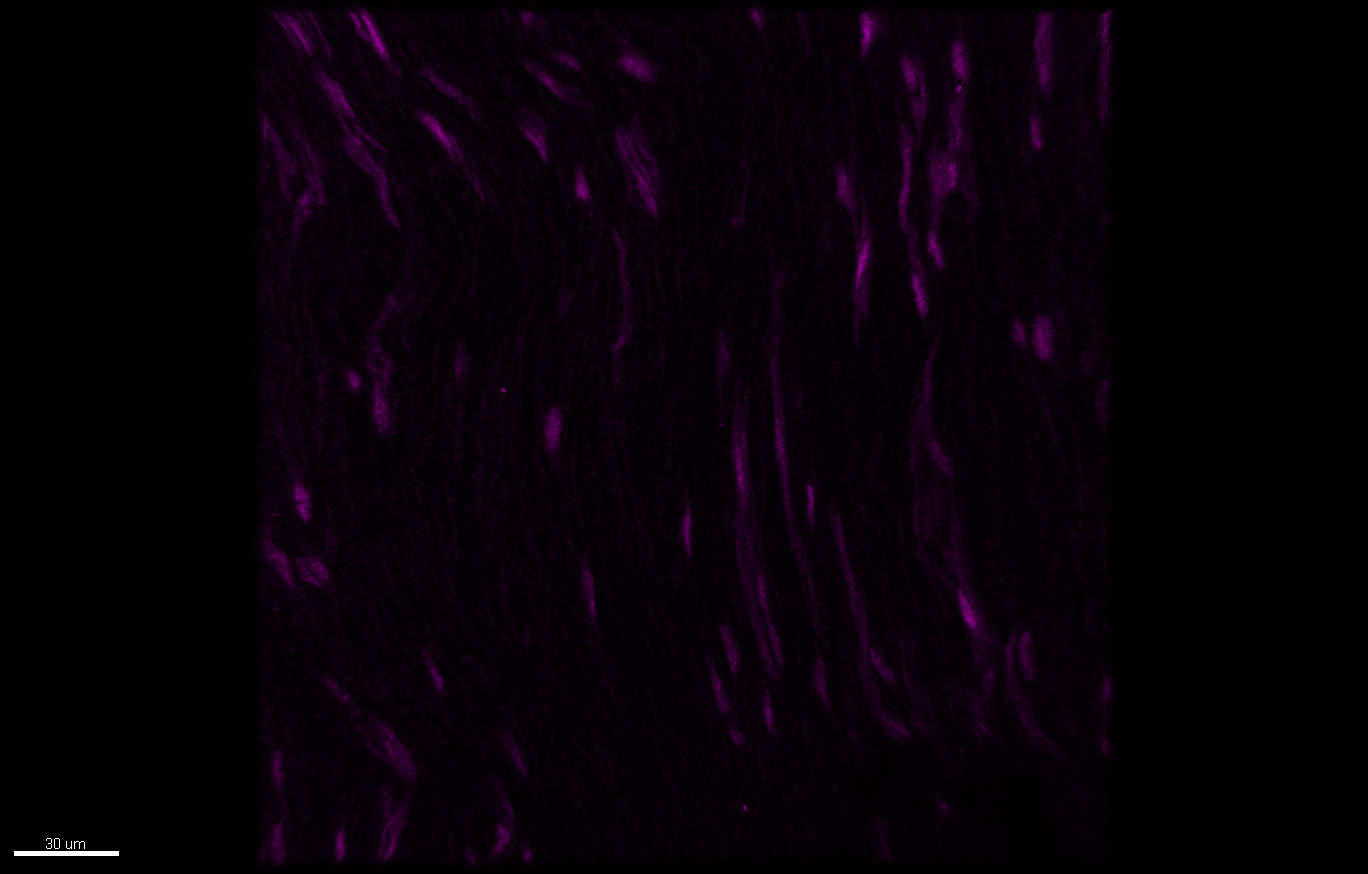

Supplement: Supplementary file 11 — Source Data for Figure 1 [file EMMM-15-e17907-s007.zip › SourceData_Fig_1/Fig_1_Source_Data__images/1F/adult_no_dmg_1/real_Adult_I_nodmg_p16_2023-02-06T13-50-14.963.tif]

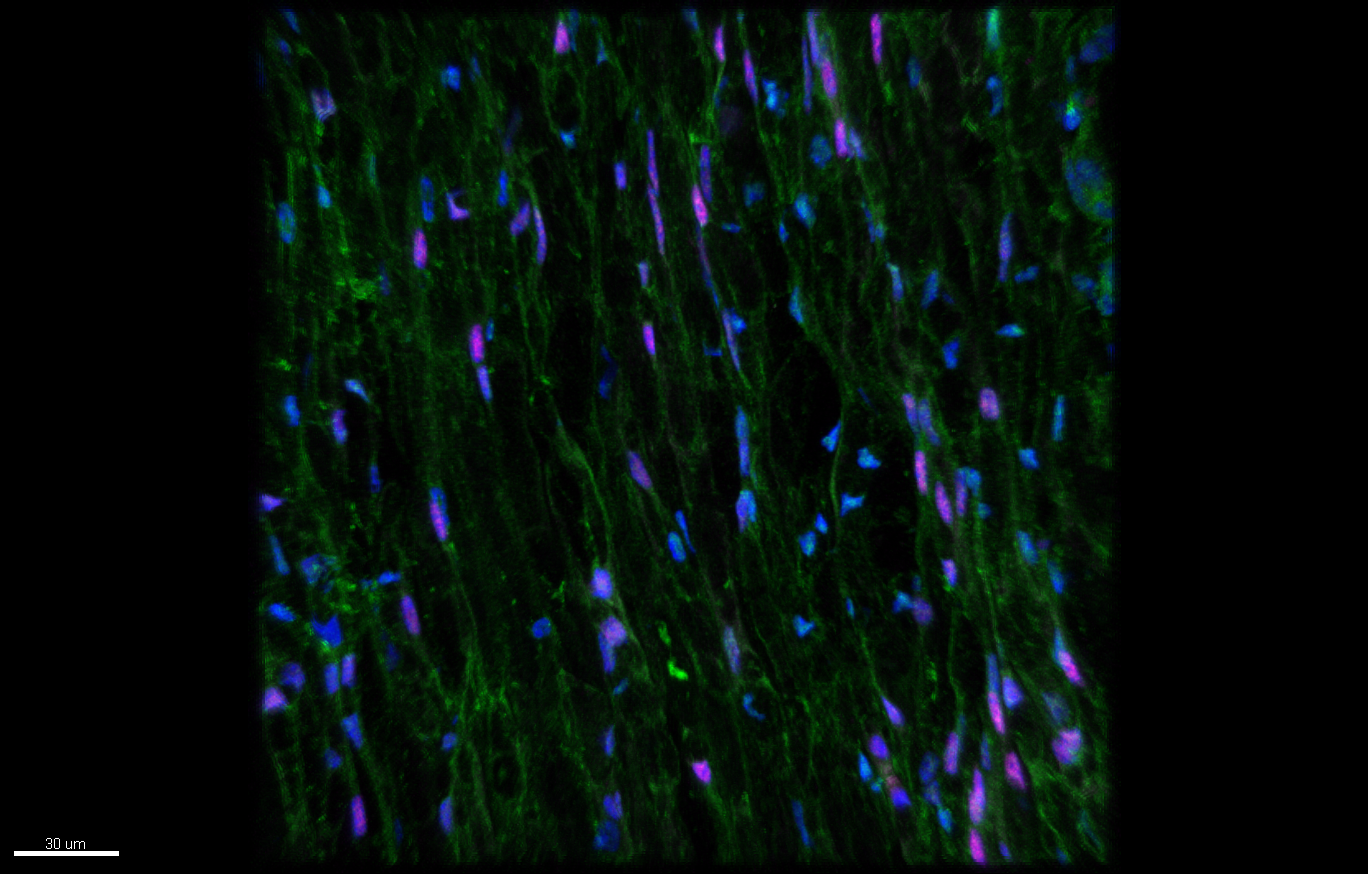

Supplement: Supplementary file 11 — Source Data for Figure 1 [file EMMM-15-e17907-s007.zip › SourceData_Fig_1/Fig_1_Source_Data__images/1F/aged_dmg_2/Aged_II_dmg_p16_2_2023-02-06T14-39-06.349.tif]

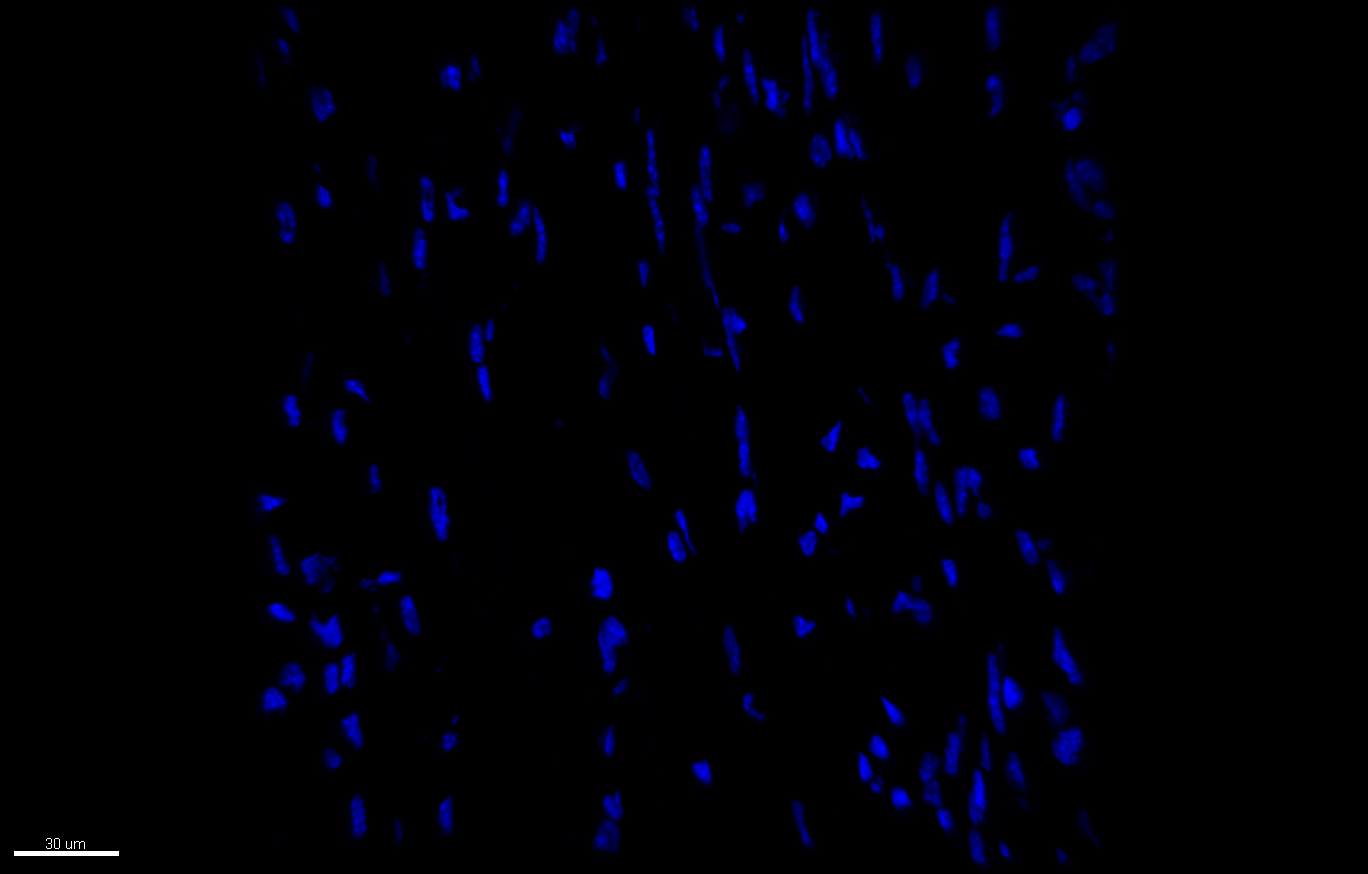

Supplement: Supplementary file 11 — Source Data for Figure 1 [file EMMM-15-e17907-s007.zip › SourceData_Fig_1/Fig_1_Source_Data__images/1F/aged_dmg_2/Aged_II_dmg_p16_2_2023-02-06T14-39-12.405.tif]

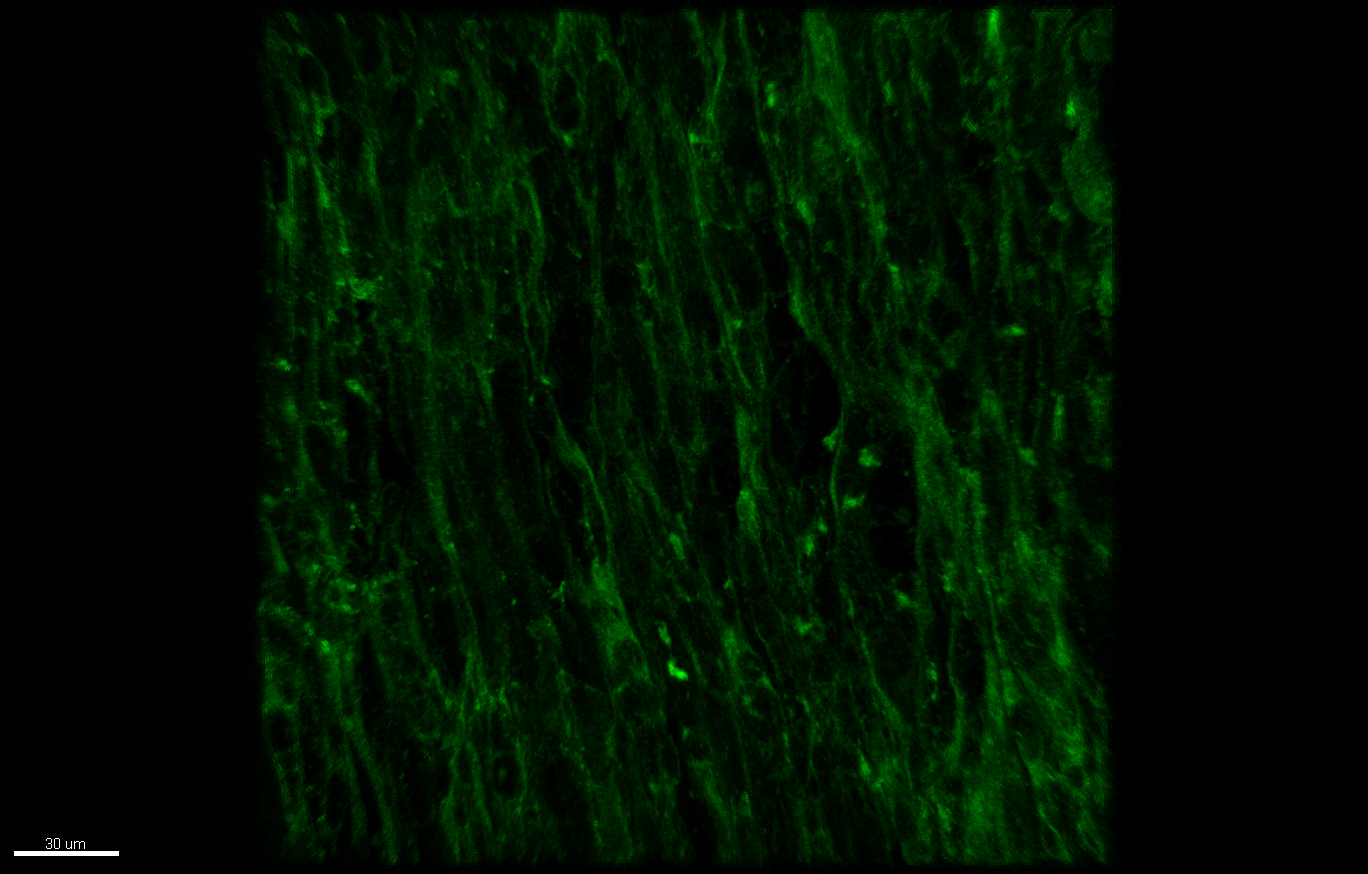

Supplement: Supplementary file 11 — Source Data for Figure 1 [file EMMM-15-e17907-s007.zip › SourceData_Fig_1/Fig_1_Source_Data__images/1F/aged_dmg_2/Aged_II_dmg_p16_2_2023-02-06T14-39-19.456.tif]

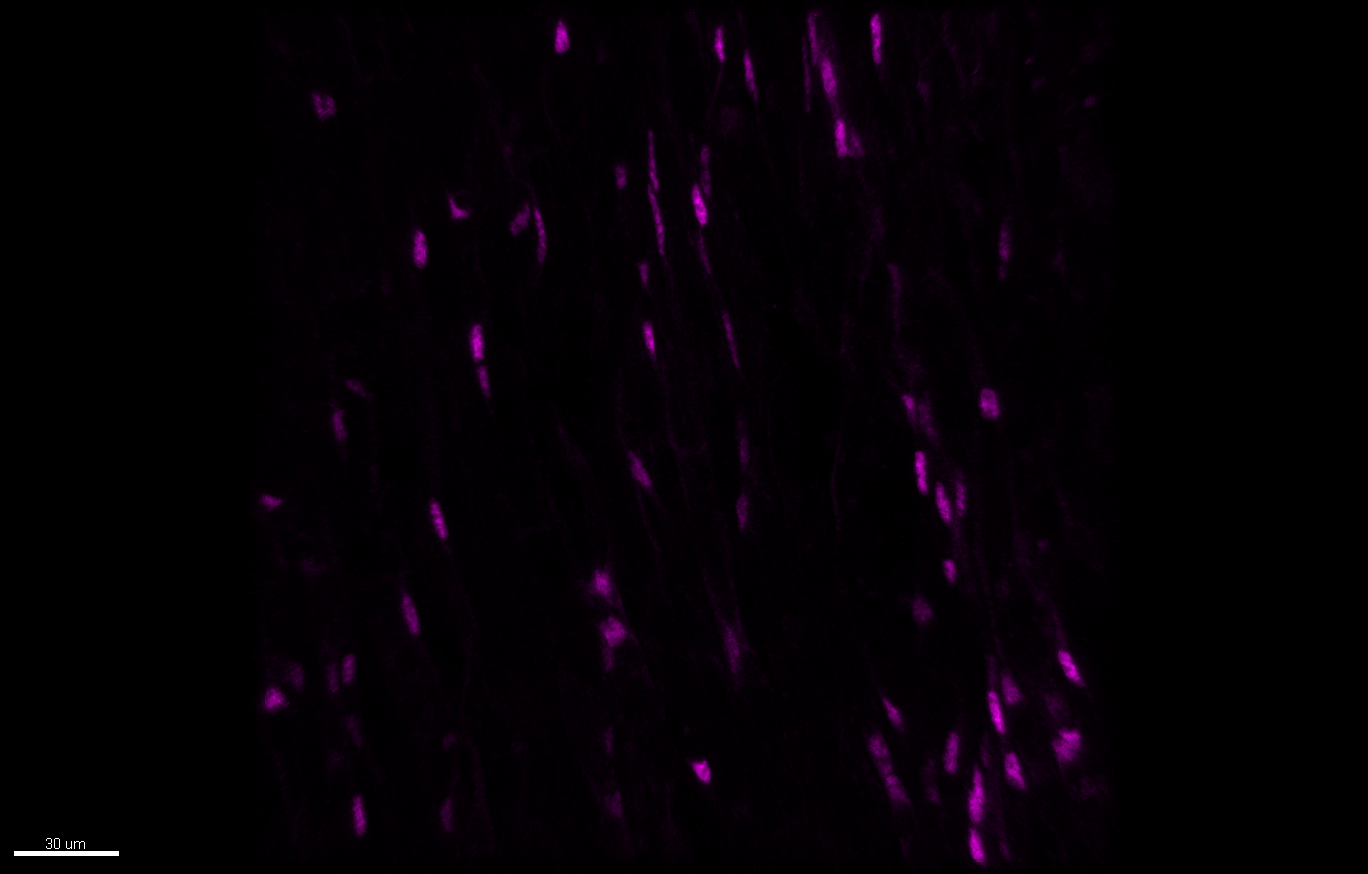

Supplement: Supplementary file 11 — Source Data for Figure 1 [file EMMM-15-e17907-s007.zip › SourceData_Fig_1/Fig_1_Source_Data__images/1F/aged_dmg_2/Aged_II_dmg_p16_2_2023-02-06T14-39-25.506.tif]

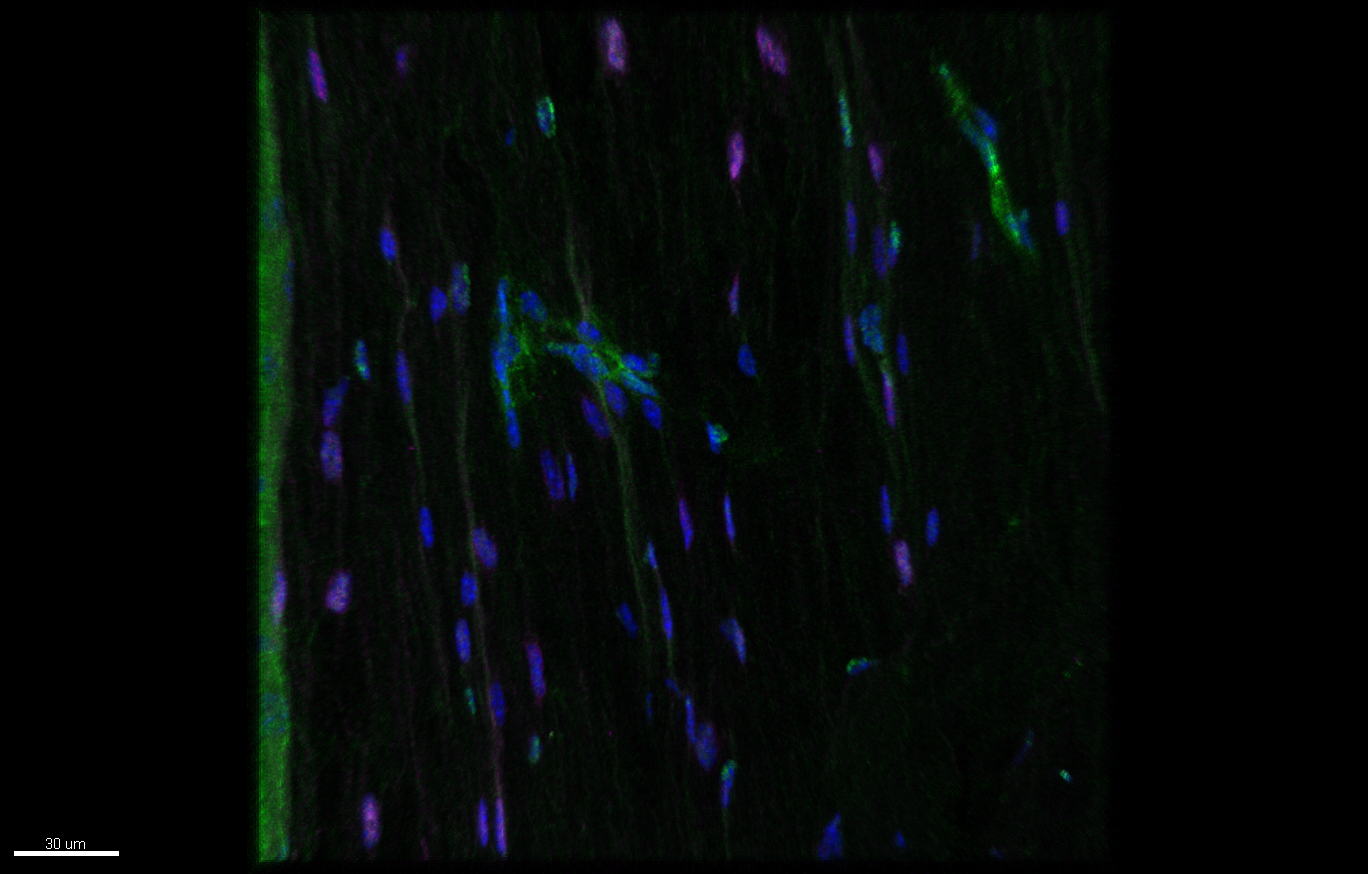

Supplement: Supplementary file 11 — Source Data for Figure 1 [file EMMM-15-e17907-s007.zip › SourceData_Fig_1/Fig_1_Source_Data__images/1F/aged_no_dmg_2/Aged_II_no_dmg_p16_1_2023-02-06T14-03-39.279.tif]

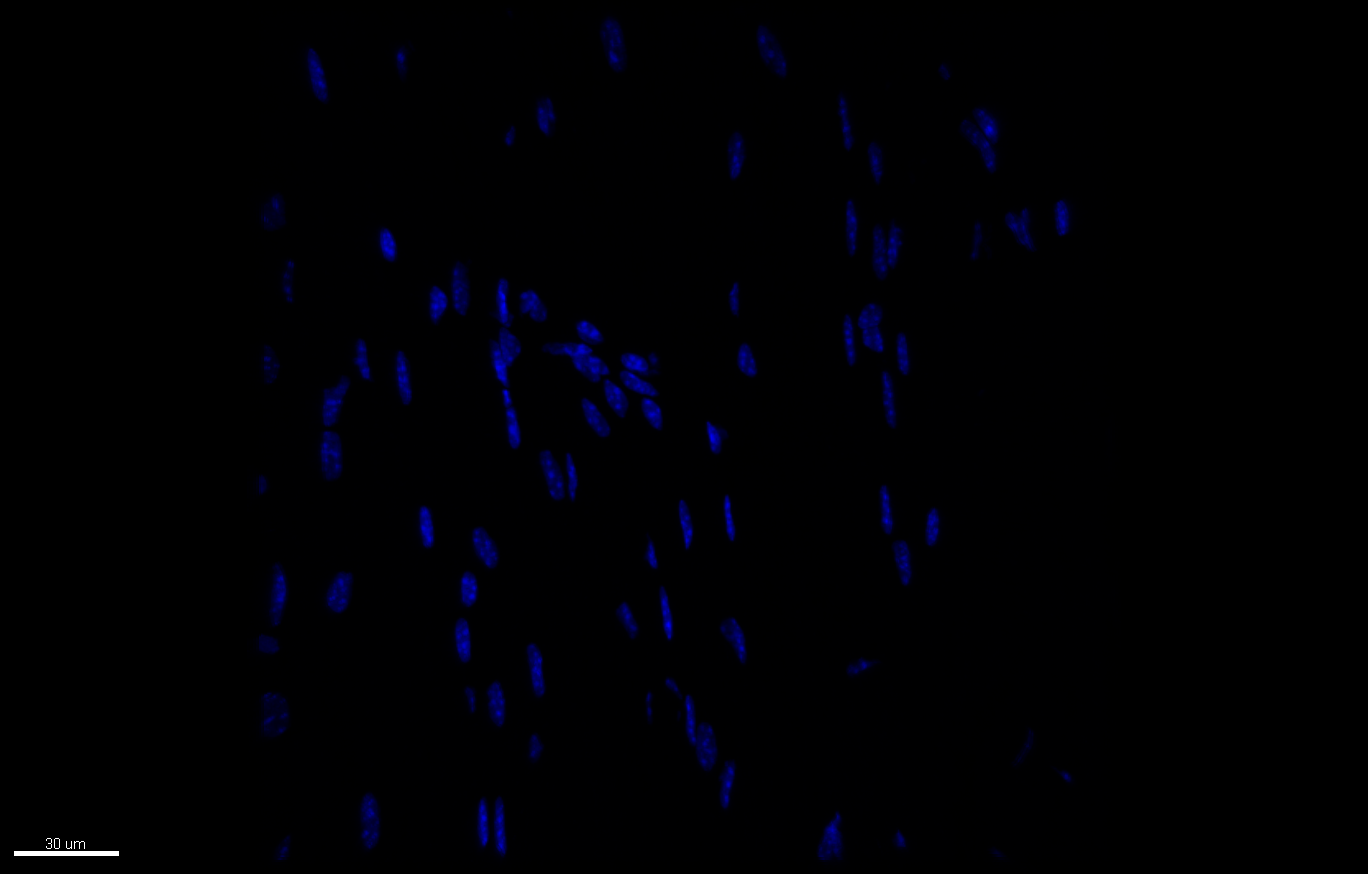

Supplement: Supplementary file 11 — Source Data for Figure 1 [file EMMM-15-e17907-s007.zip › SourceData_Fig_1/Fig_1_Source_Data__images/1F/aged_no_dmg_2/Aged_II_no_dmg_p16_1_2023-02-06T14-03-45.291.tif]

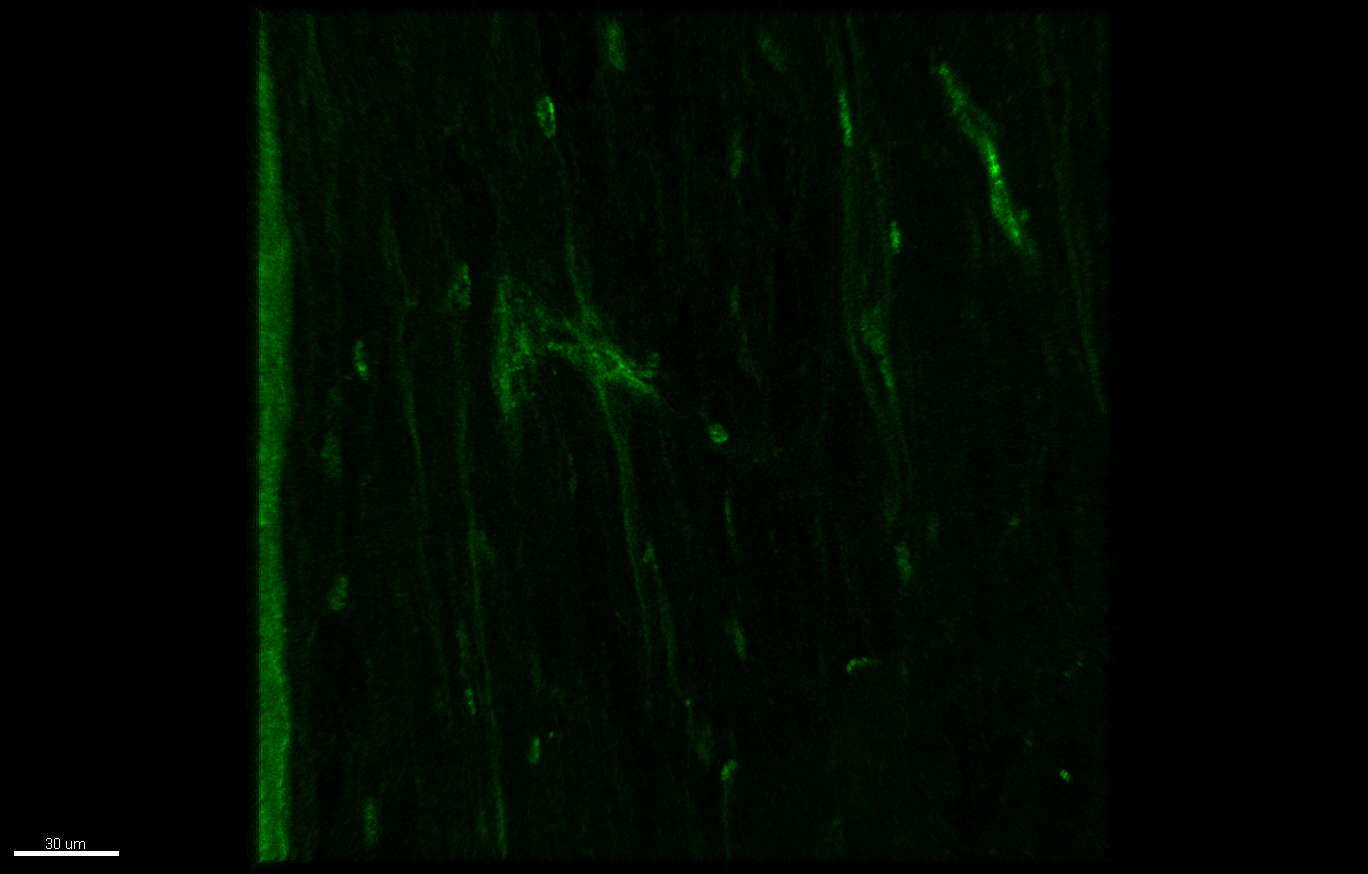

Supplement: Supplementary file 11 — Source Data for Figure 1 [file EMMM-15-e17907-s007.zip › SourceData_Fig_1/Fig_1_Source_Data__images/1F/aged_no_dmg_2/Aged_II_no_dmg_p16_1_2023-02-06T14-03-50.588.tif]

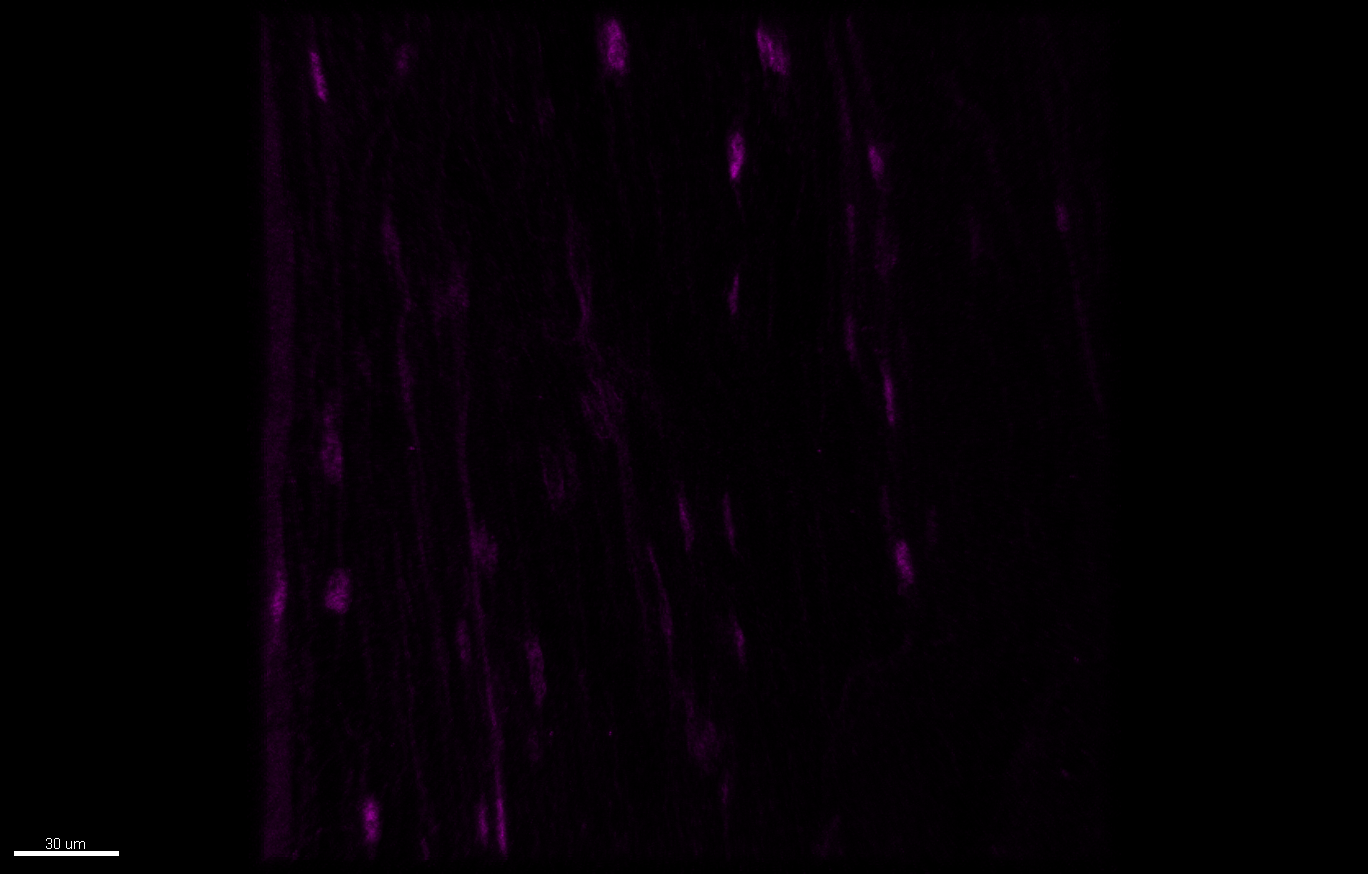

Supplement: Supplementary file 11 — Source Data for Figure 1 [file EMMM-15-e17907-s007.zip › SourceData_Fig_1/Fig_1_Source_Data__images/1F/aged_no_dmg_2/Aged_II_no_dmg_p16_1_2023-02-06T14-03-56.635.tif]

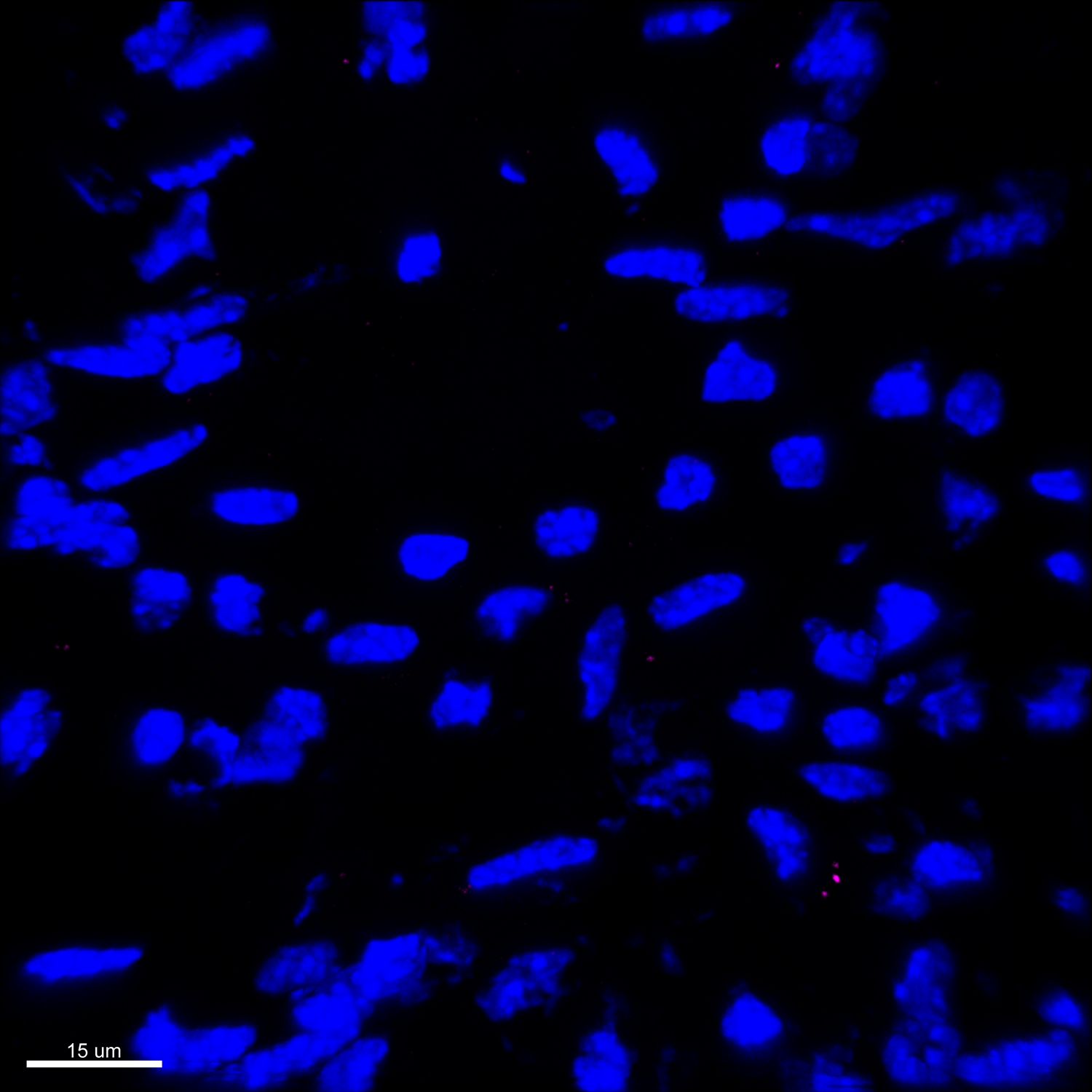

Supplement: Supplementary file 11 — Source Data for Figure 1 [file EMMM-15-e17907-s007.zip › SourceData_Fig_1/Fig_1_Source_Data__images/1K/Adult_WT/YOUNG_ALL.tif]

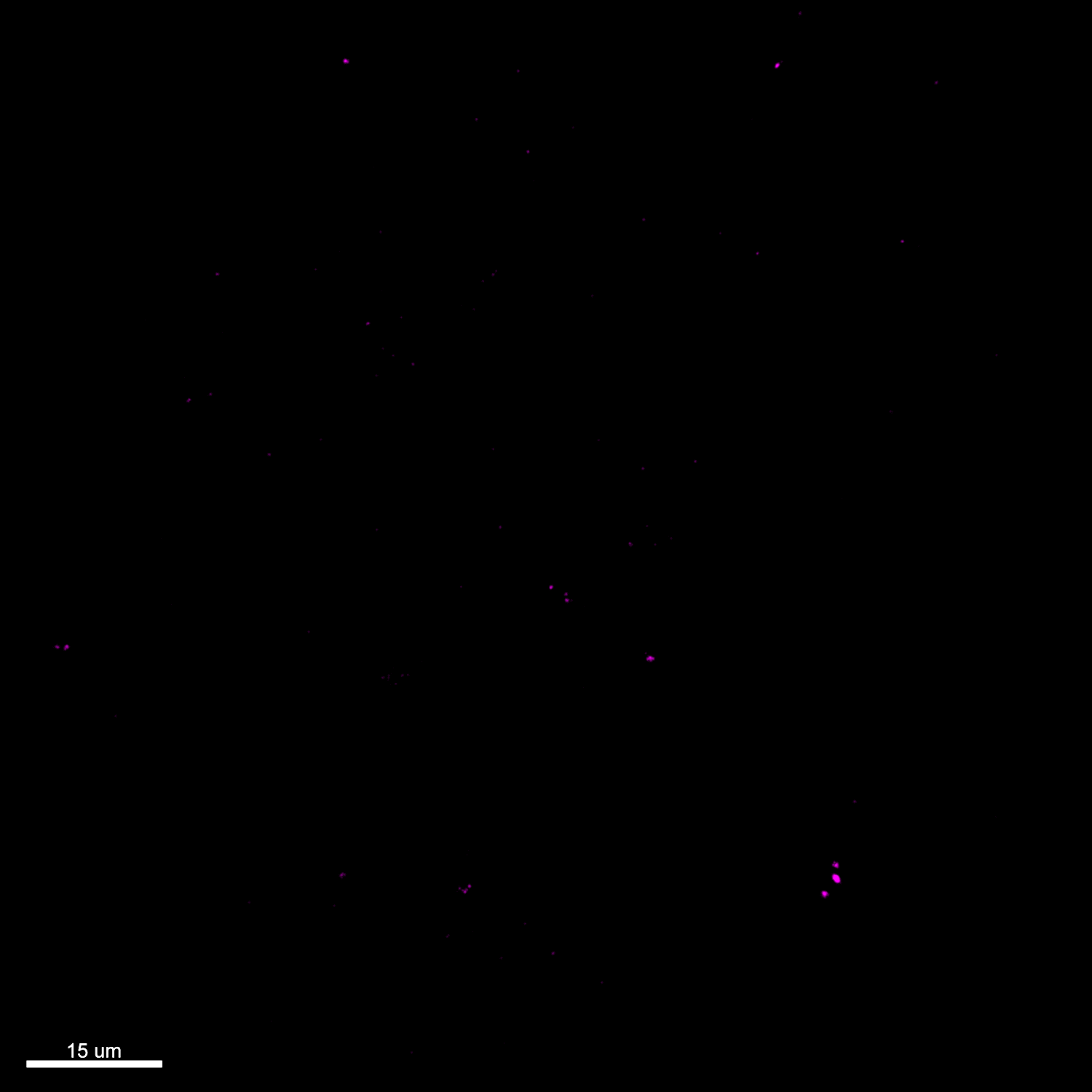

Supplement: Supplementary file 11 — Source Data for Figure 1 [file EMMM-15-e17907-s007.zip › SourceData_Fig_1/Fig_1_Source_Data__images/1K/Adult_WT/YOUNG_magenta.tif]

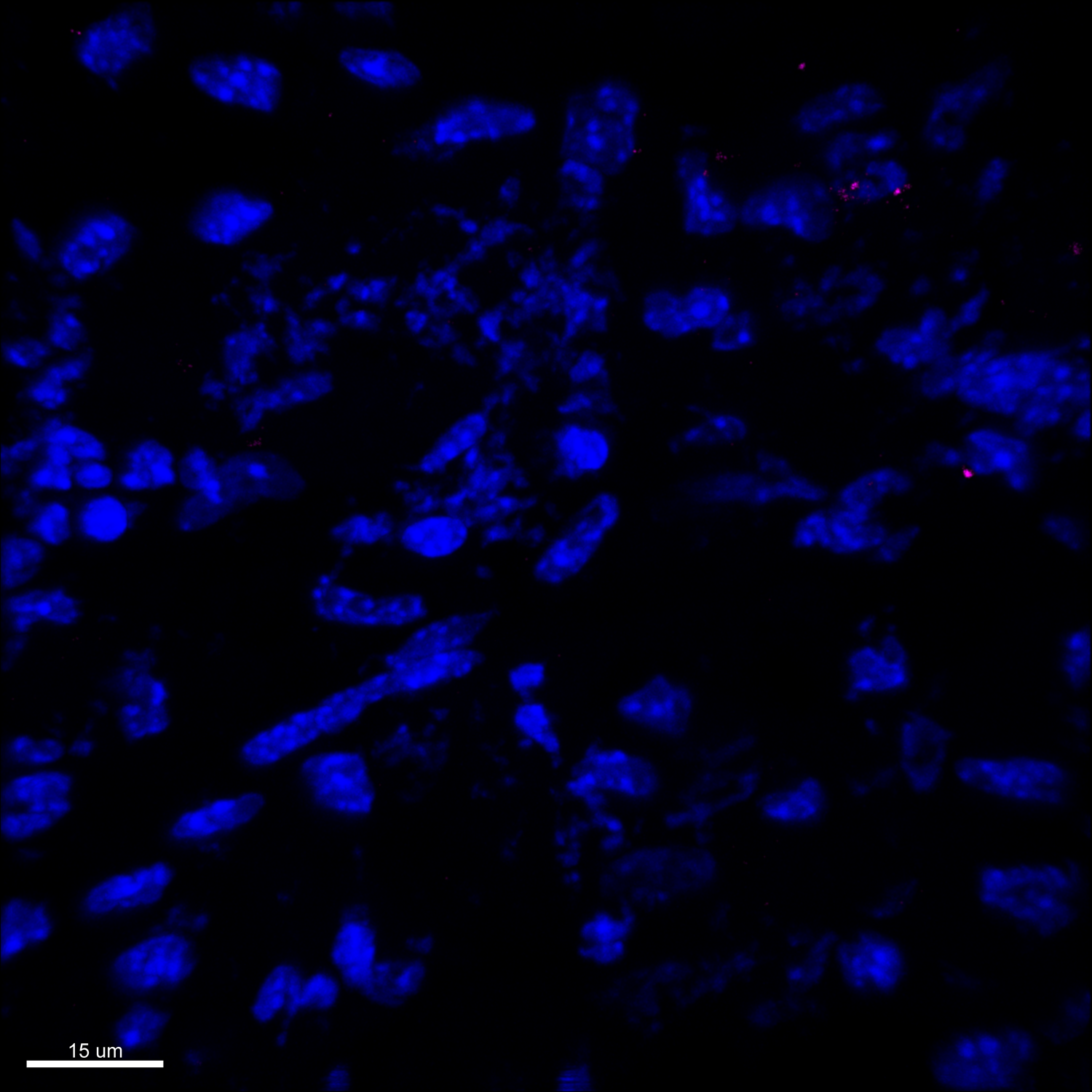

Supplement: Supplementary file 11 — Source Data for Figure 1 [file EMMM-15-e17907-s007.zip › SourceData_Fig_1/Fig_1_Source_Data__images/1K/Aged_OE/OE_1yo_ALL.tif]

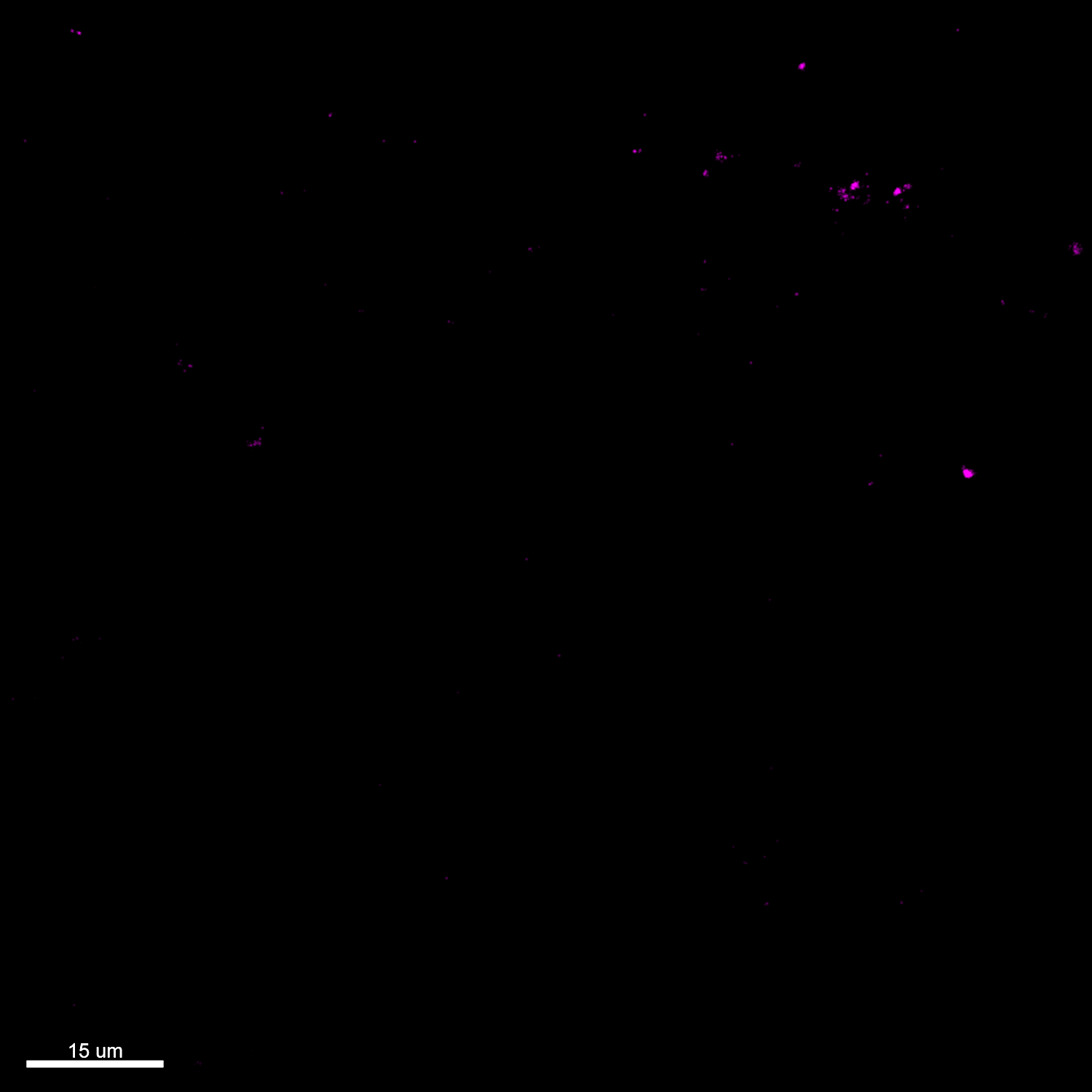

Supplement: Supplementary file 11 — Source Data for Figure 1 [file EMMM-15-e17907-s007.zip › SourceData_Fig_1/Fig_1_Source_Data__images/1K/Aged_OE/OE_1yo_magenta.tif]

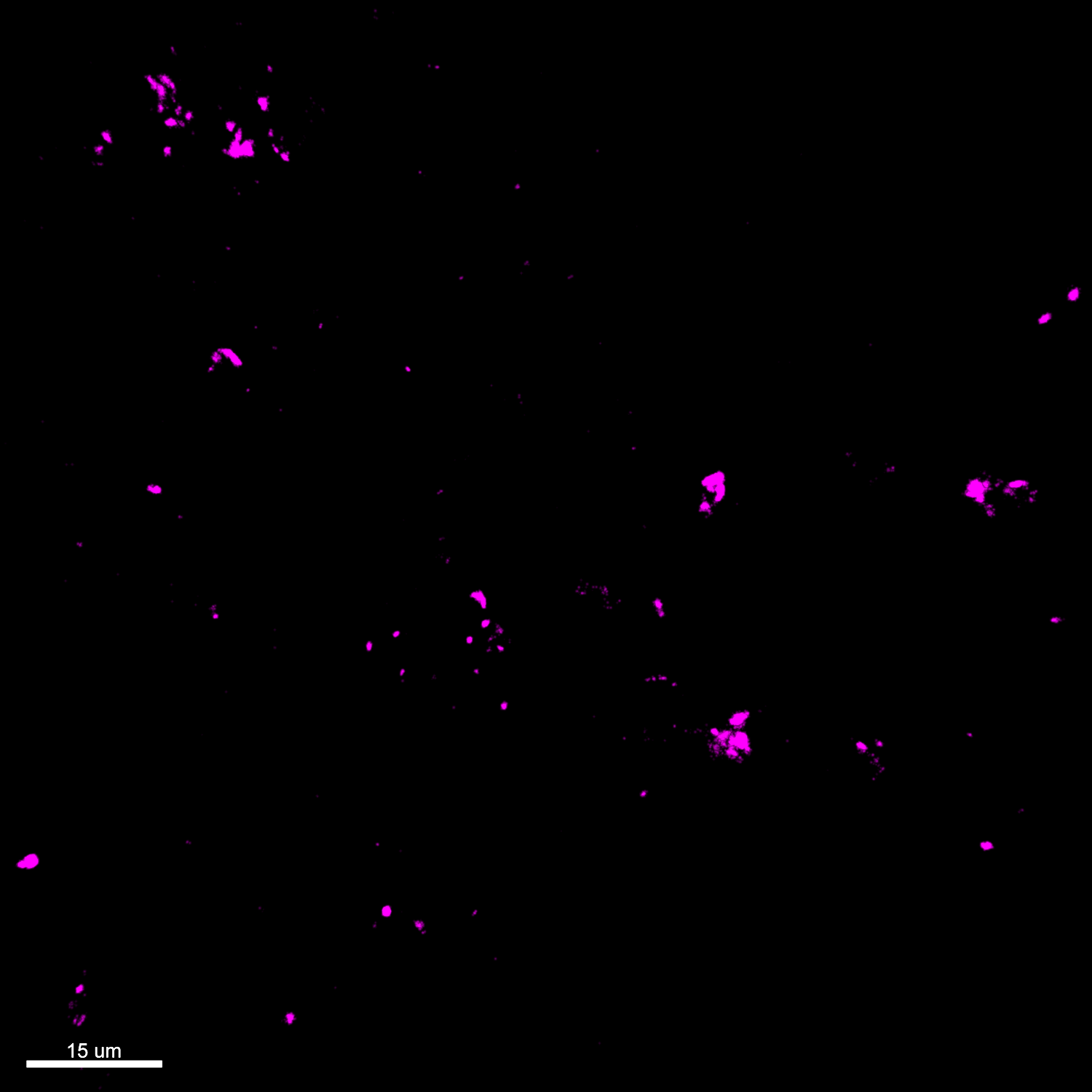

Supplement: Supplementary file 11 — Source Data for Figure 1 [file EMMM-15-e17907-s007.zip › SourceData_Fig_1/Fig_1_Source_Data__images/1K/Aged_WT/WT_1yo_magenta.tif]

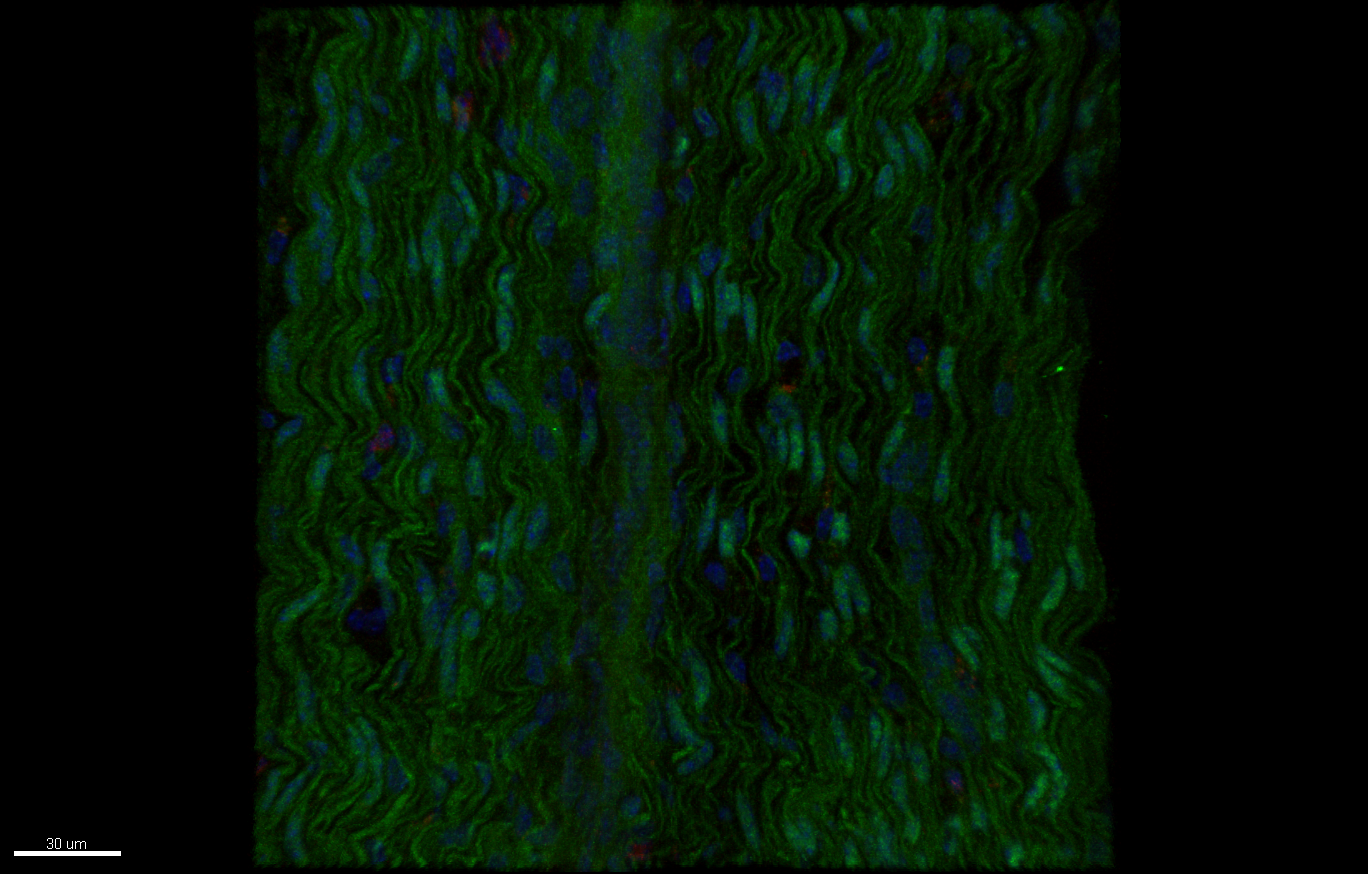

Supplement: Supplementary file 11 — Source Data for Figure 1 [file EMMM-15-e17907-s007.zip › SourceData_Fig_1/Fig_1_Source_Data__images/1M/Adult_OE/Young_42dpi_p16_OE_I_2023-02-06T14-55-45.997.tif]

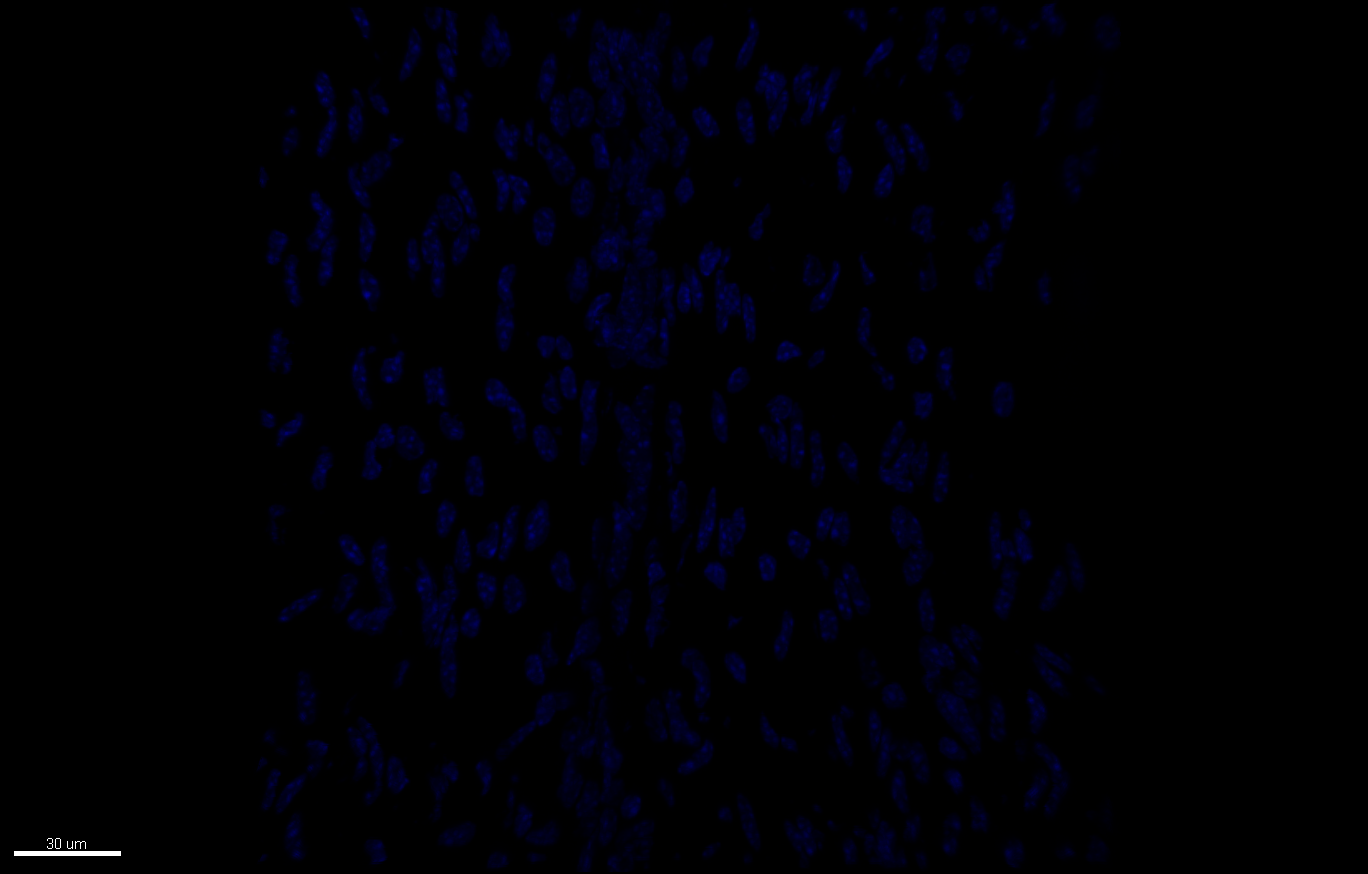

Supplement: Supplementary file 11 — Source Data for Figure 1 [file EMMM-15-e17907-s007.zip › SourceData_Fig_1/Fig_1_Source_Data__images/1M/Adult_OE/Young_42dpi_p16_OE_I_2023-02-06T14-55-52.479.tif]

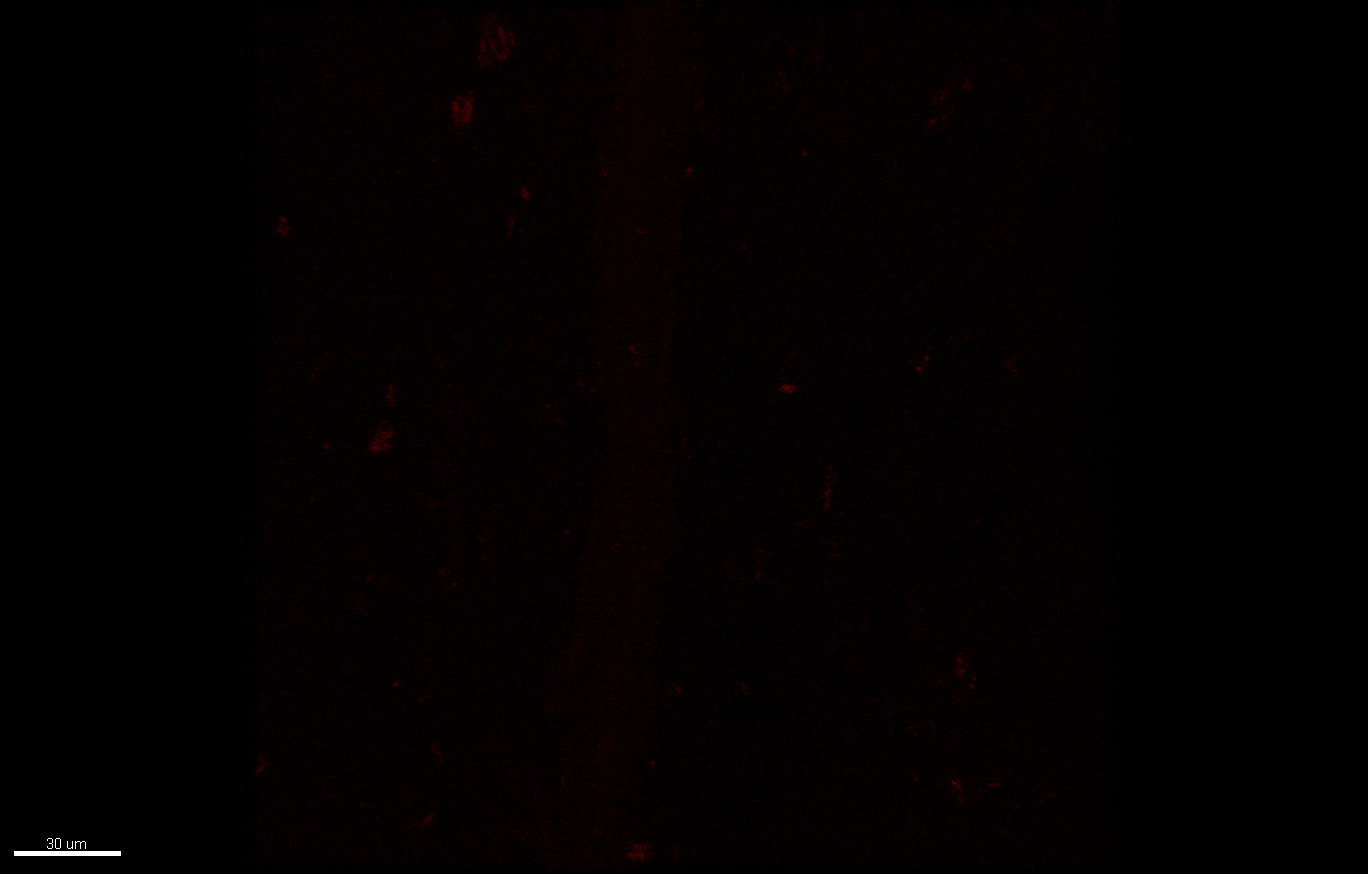

Supplement: Supplementary file 11 — Source Data for Figure 1 [file EMMM-15-e17907-s007.zip › SourceData_Fig_1/Fig_1_Source_Data__images/1M/Adult_OE/Young_42dpi_p16_OE_I_2023-02-06T14-55-59.304.tif]

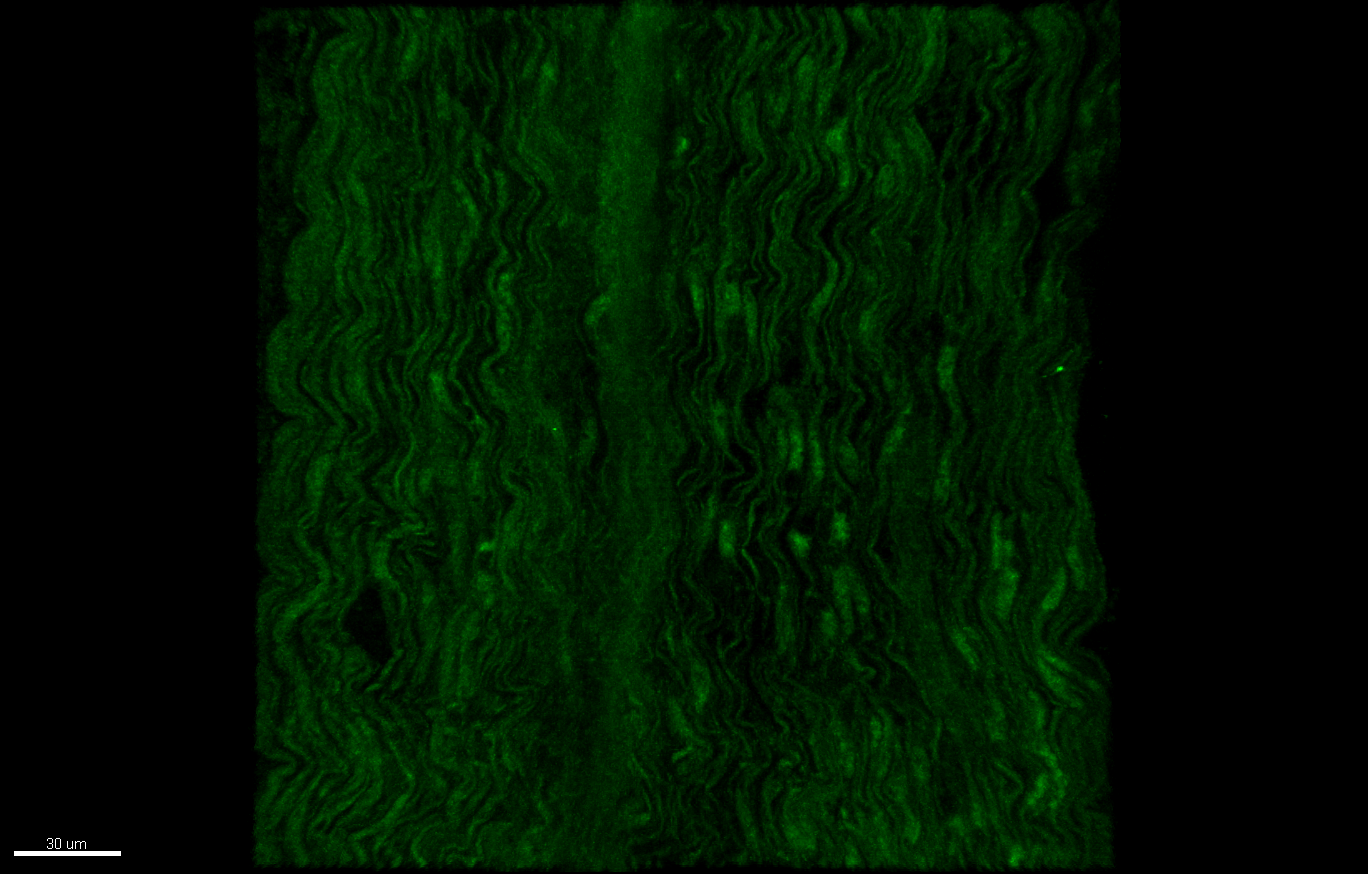

Supplement: Supplementary file 11 — Source Data for Figure 1 [file EMMM-15-e17907-s007.zip › SourceData_Fig_1/Fig_1_Source_Data__images/1M/Adult_OE/Young_42dpi_p16_OE_I_2023-02-06T14-56-06.198.tif]

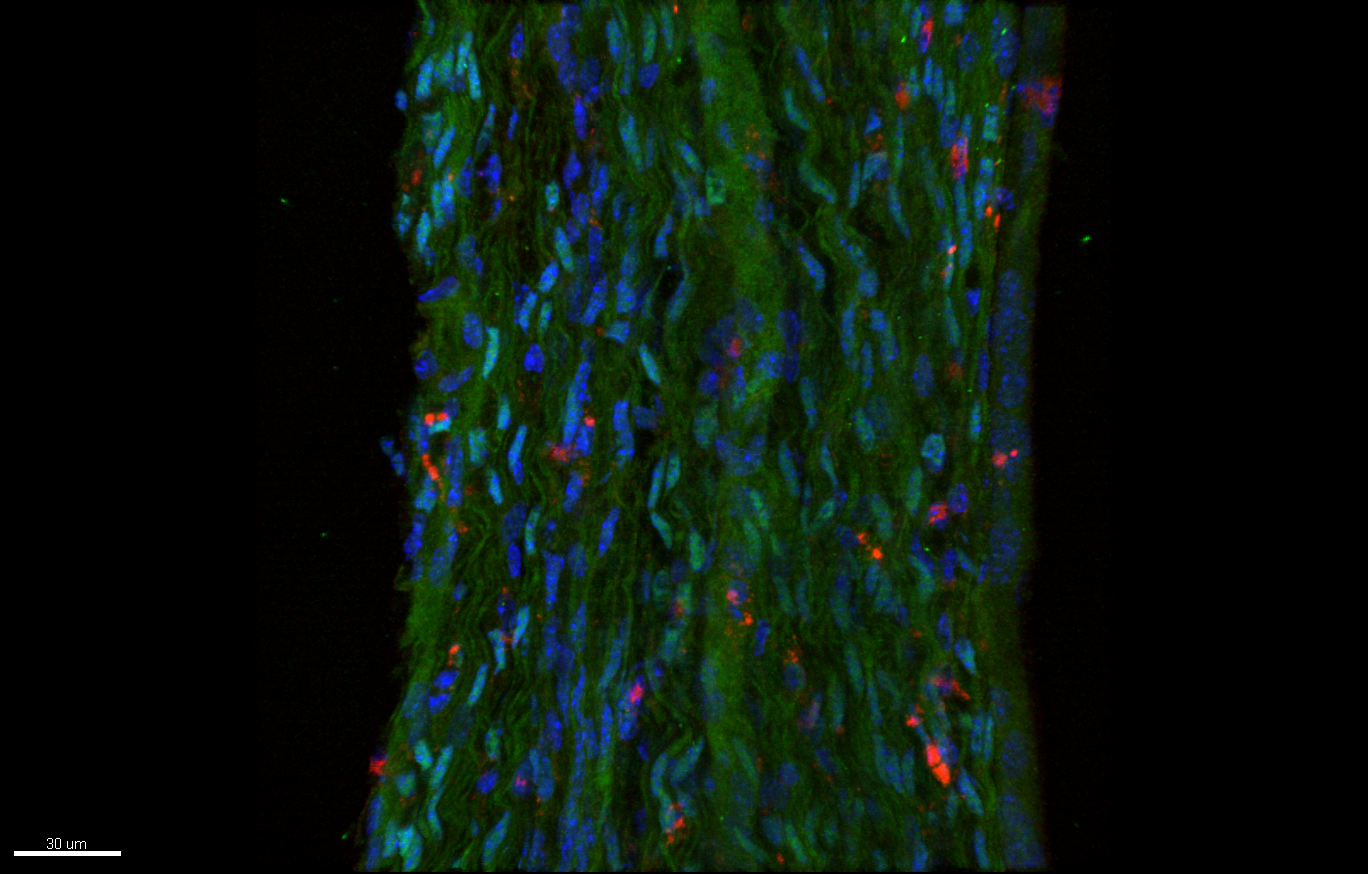

Supplement: Supplementary file 11 — Source Data for Figure 1 [file EMMM-15-e17907-s007.zip › SourceData_Fig_1/Fig_1_Source_Data__images/1M/Adult_wt/Young_42dpi_p16_wt_I_2023-02-06T15-17-19.471.tif]

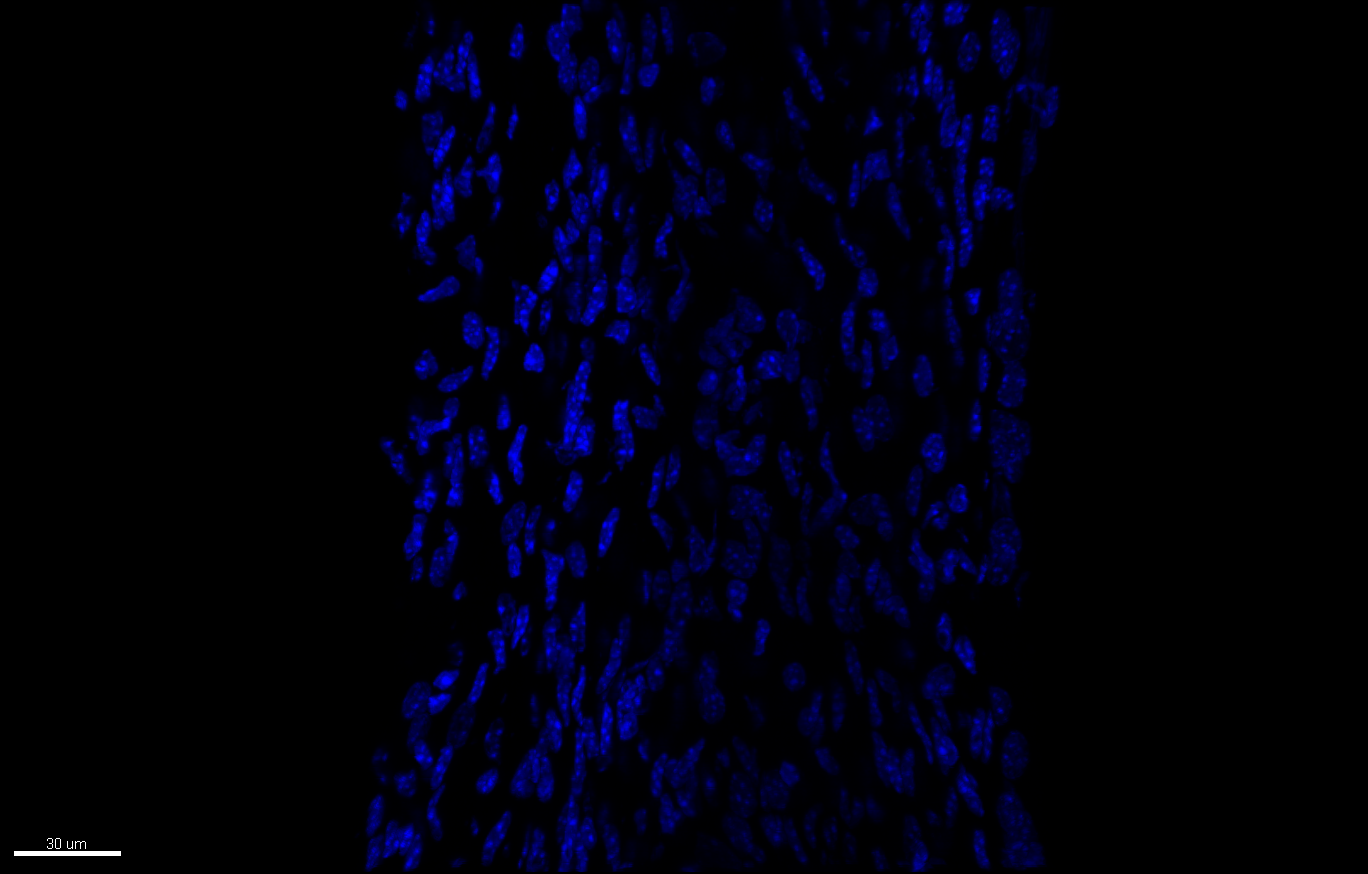

Supplement: Supplementary file 11 — Source Data for Figure 1 [file EMMM-15-e17907-s007.zip › SourceData_Fig_1/Fig_1_Source_Data__images/1M/Adult_wt/Young_42dpi_p16_wt_I_2023-02-06T15-17-28.050.tif]

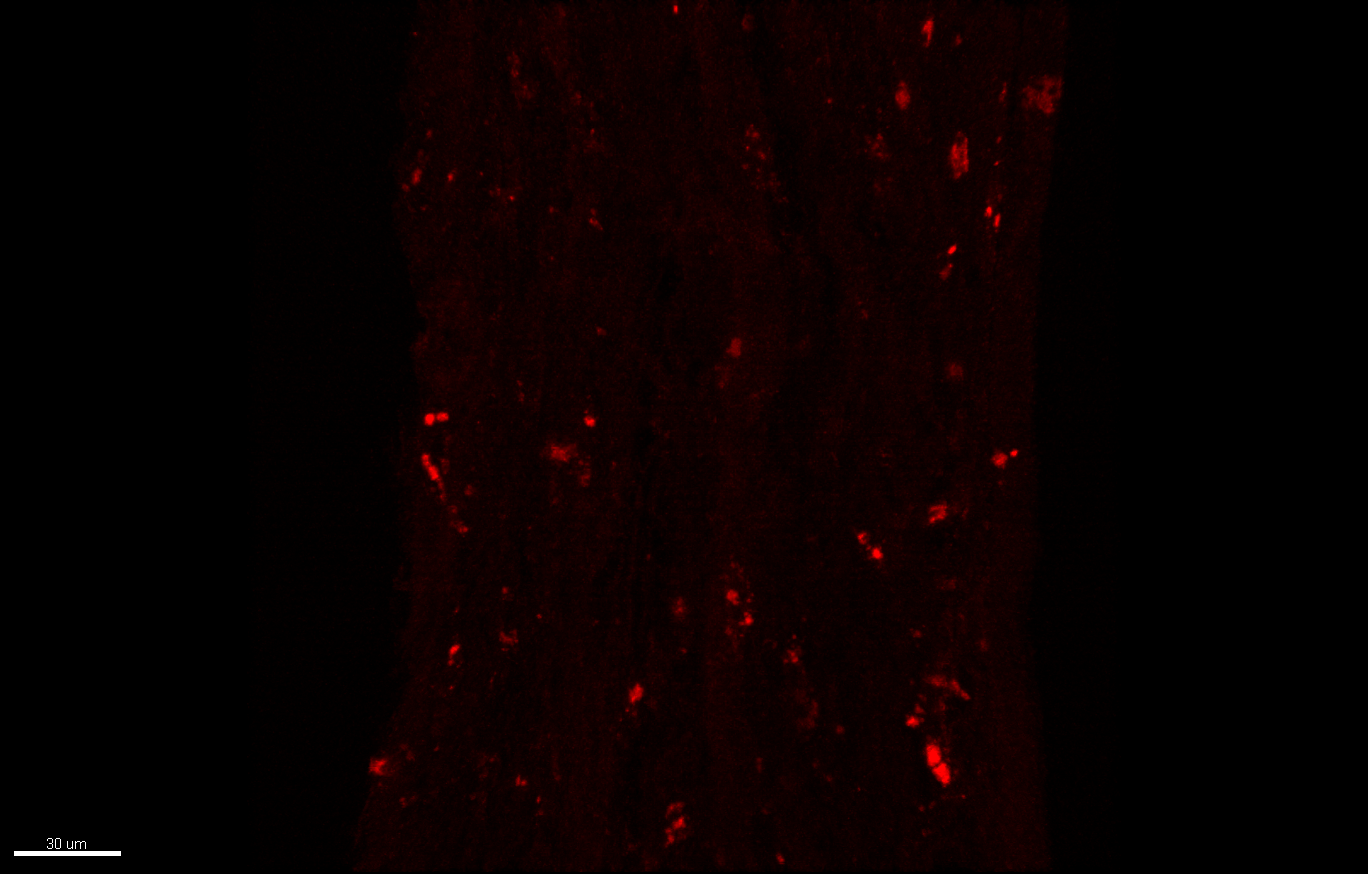

Supplement: Supplementary file 11 — Source Data for Figure 1 [file EMMM-15-e17907-s007.zip › SourceData_Fig_1/Fig_1_Source_Data__images/1M/Adult_wt/Young_42dpi_p16_wt_I_2023-02-06T15-17-35.344.tif]

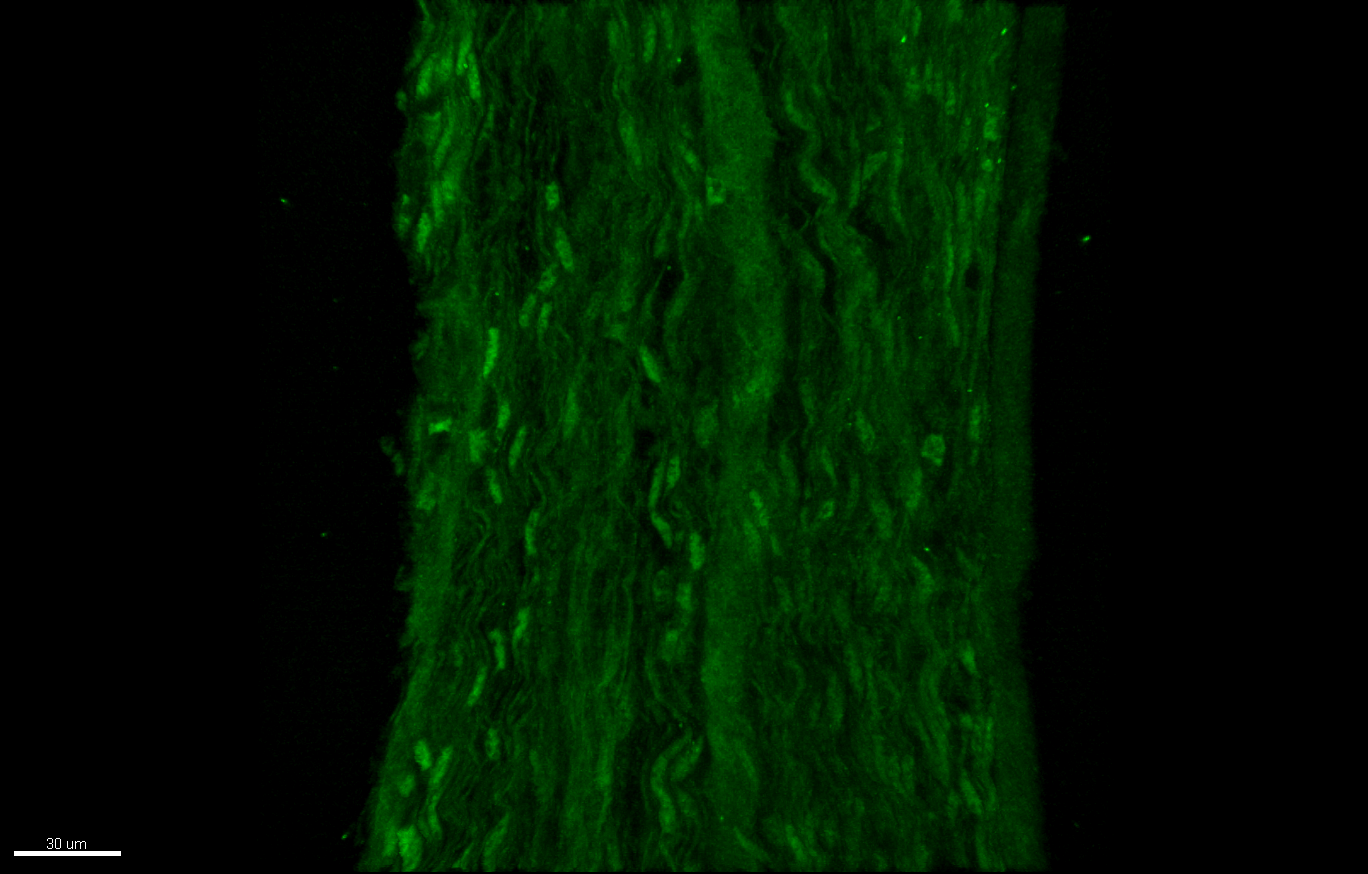

Supplement: Supplementary file 11 — Source Data for Figure 1 [file EMMM-15-e17907-s007.zip › SourceData_Fig_1/Fig_1_Source_Data__images/1M/Adult_wt/Young_42dpi_p16_wt_I_2023-02-06T15-17-41.605.tif]

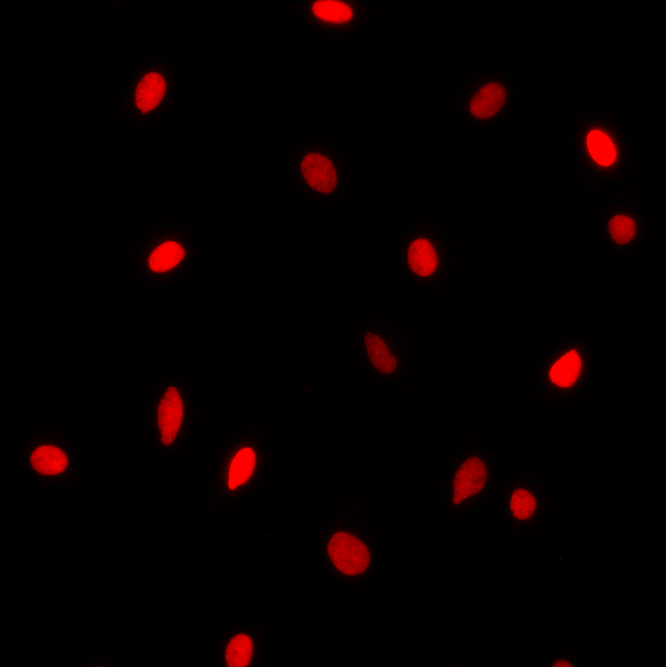

Supplement: Supplementary file 13 — Source Data for Figure 3 [file EMMM-15-e17907-s005.zip › SourceData_Fig_3/Fig_3_Source_Data_images/3A/rSC.tif]

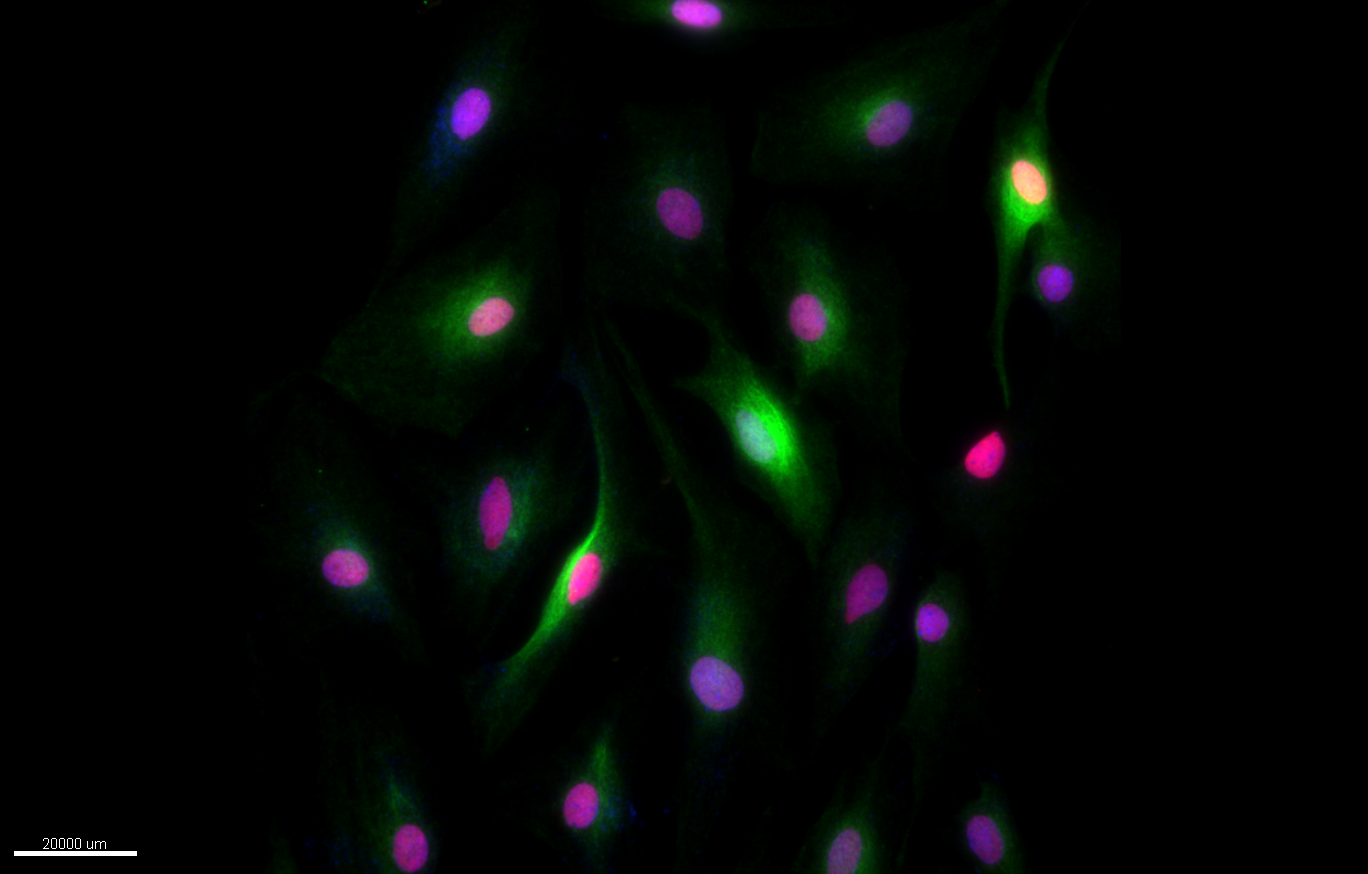

Supplement: Supplementary file 13 — Source Data for Figure 3 [file EMMM-15-e17907-s005.zip › SourceData_Fig_3/Fig_3_Source_Data_images/3A/rSC_all.tif]

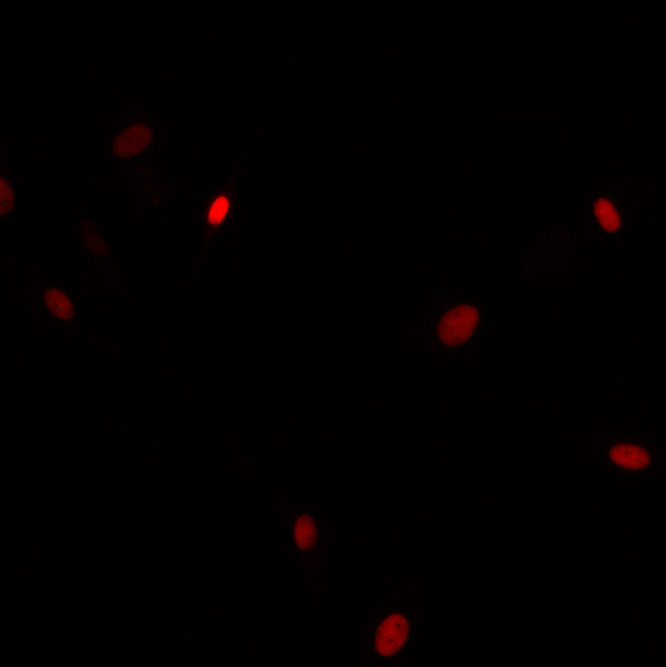

Supplement: Supplementary file 13 — Source Data for Figure 3 [file EMMM-15-e17907-s005.zip › SourceData_Fig_3/Fig_3_Source_Data_images/3A/siSC.tif]

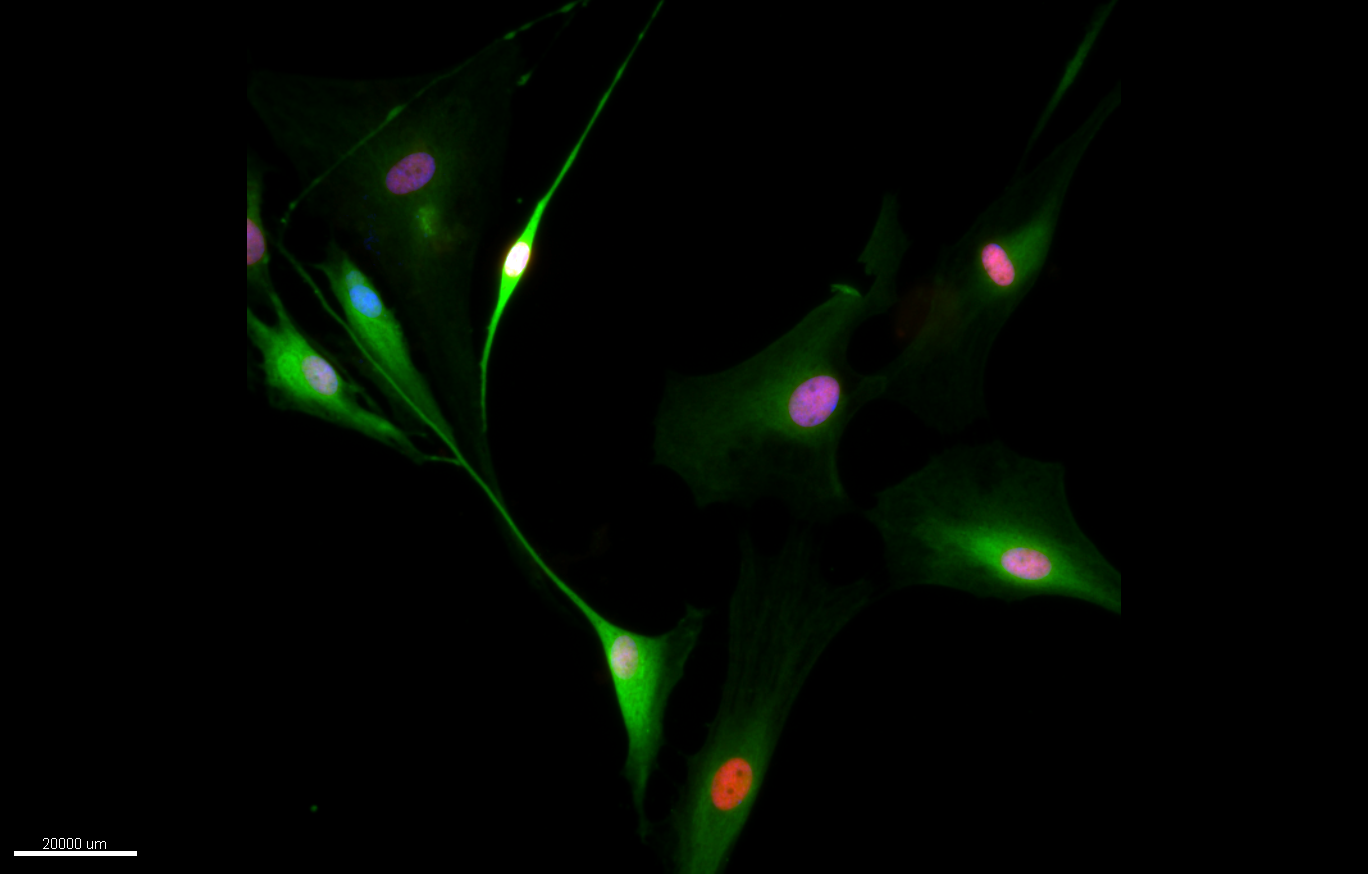

Supplement: Supplementary file 13 — Source Data for Figure 3 [file EMMM-15-e17907-s005.zip › SourceData_Fig_3/Fig_3_Source_Data_images/3A/siSC_all.tif]

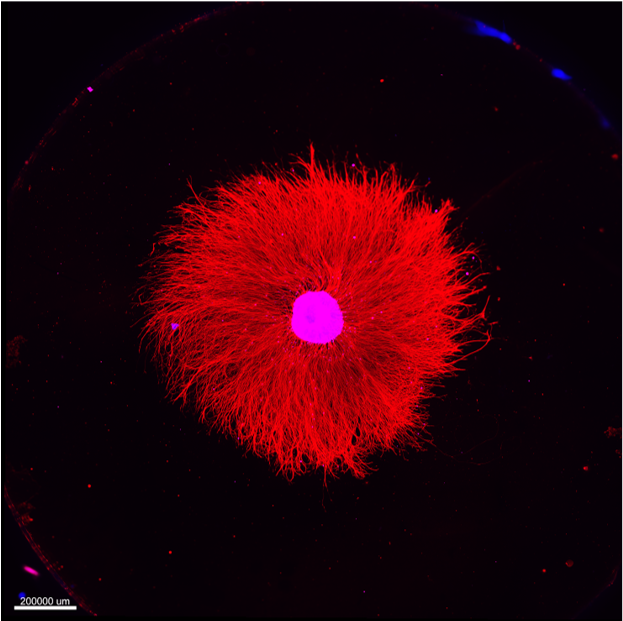

Supplement: Supplementary file 13 — Source Data for Figure 3 [file EMMM-15-e17907-s005.zip › SourceData_Fig_3/Fig_3_Source_Data_images/3C/MIX.tif]

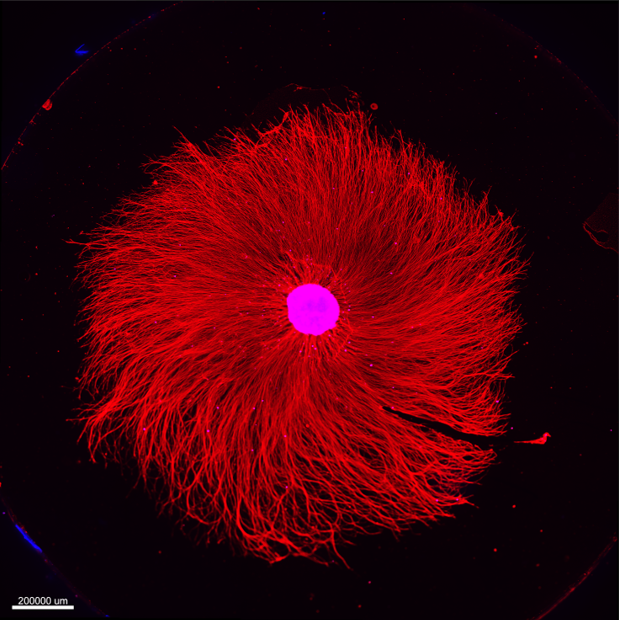

Supplement: Supplementary file 13 — Source Data for Figure 3 [file EMMM-15-e17907-s005.zip › SourceData_Fig_3/Fig_3_Source_Data_images/3C/rSC.tif]

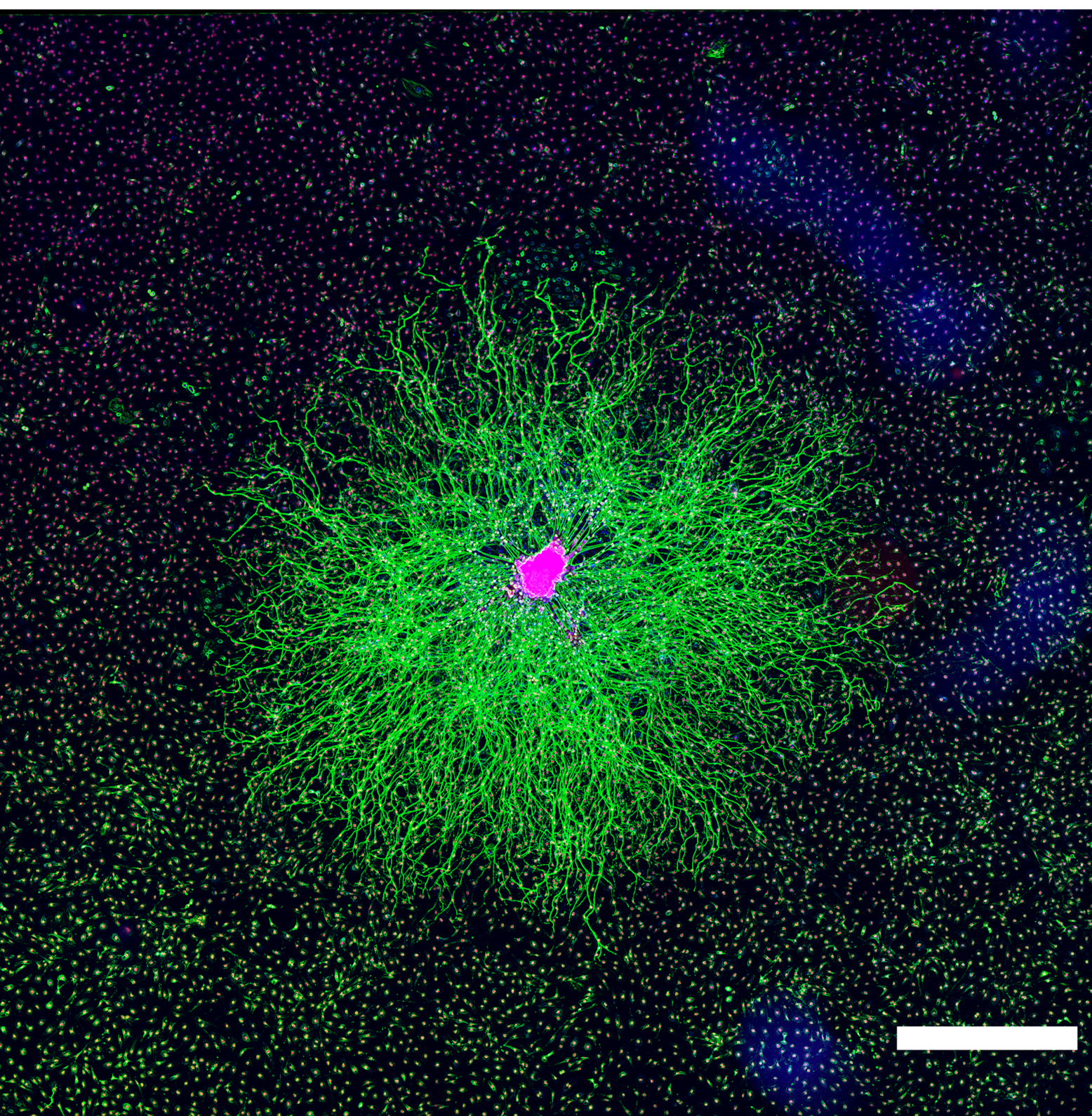

Supplement: Supplementary file 13 — Source Data for Figure 3 [file EMMM-15-e17907-s005.zip › SourceData_Fig_3/Fig_3_Source_Data_images/3C/r_SC.tif]

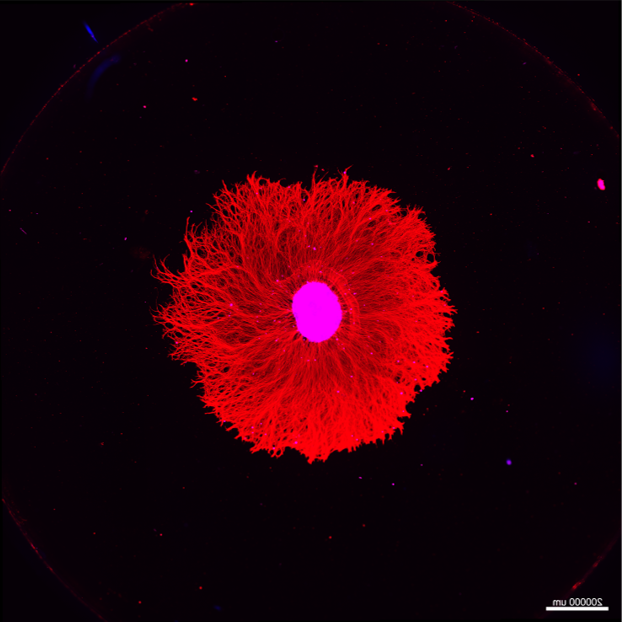

Supplement: Supplementary file 13 — Source Data for Figure 3 [file EMMM-15-e17907-s005.zip › SourceData_Fig_3/Fig_3_Source_Data_images/3C/siSC.tif]

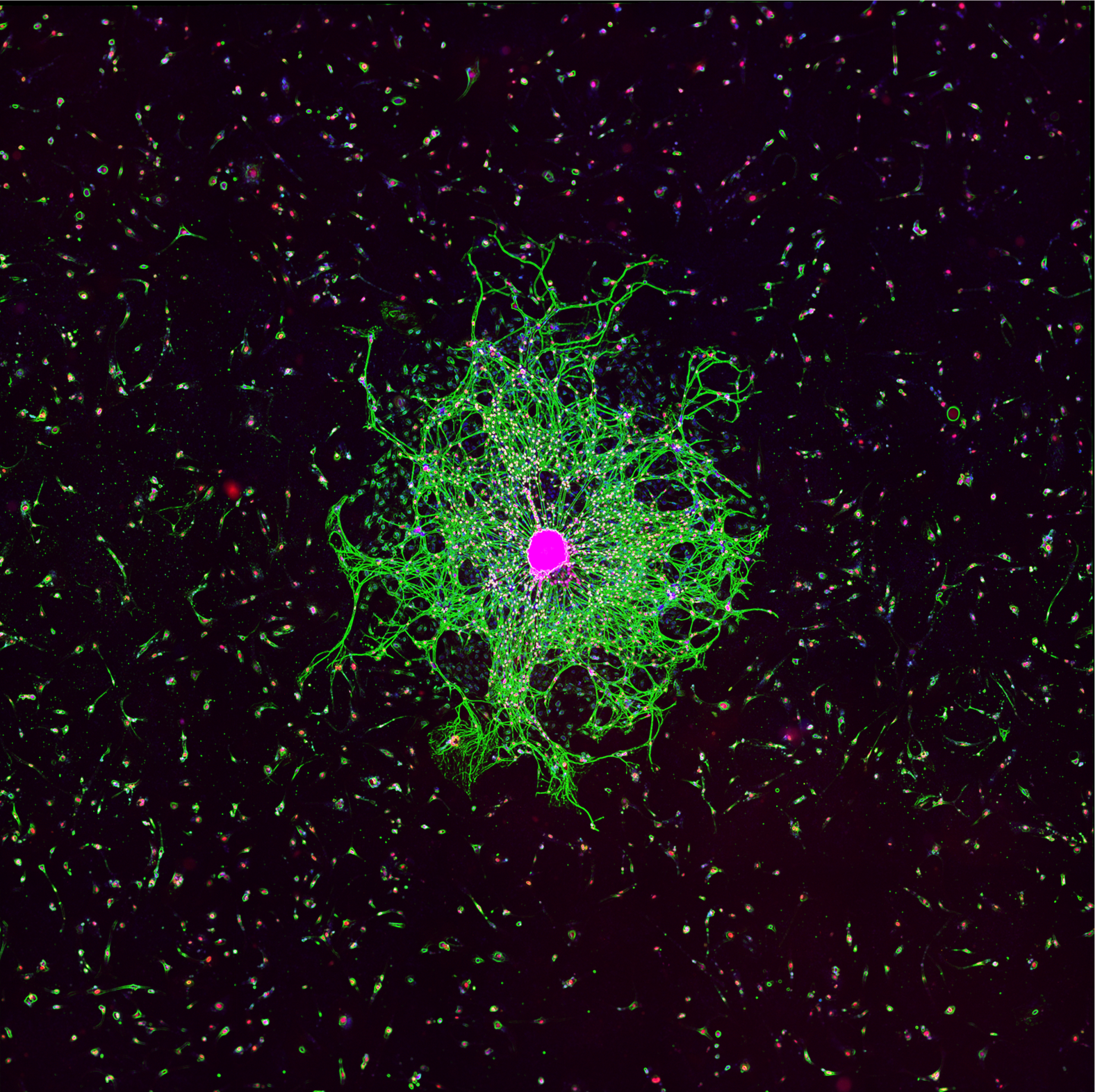

Supplement: Supplementary file 13 — Source Data for Figure 3 [file EMMM-15-e17907-s005.zip › SourceData_Fig_3/Fig_3_Source_Data_images/3C/si_SC.tif]

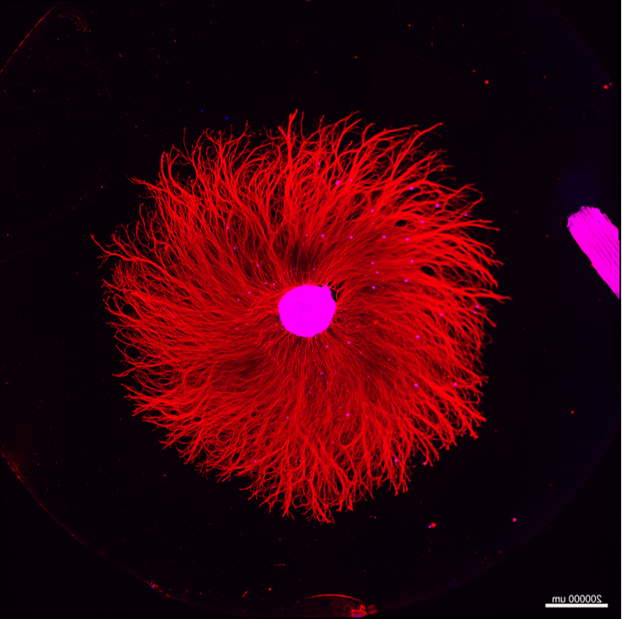

Supplement: Supplementary file 13 — Source Data for Figure 3 [file EMMM-15-e17907-s005.zip › SourceData_Fig_3/Fig_3_Source_Data_images/3C/UC.tif]

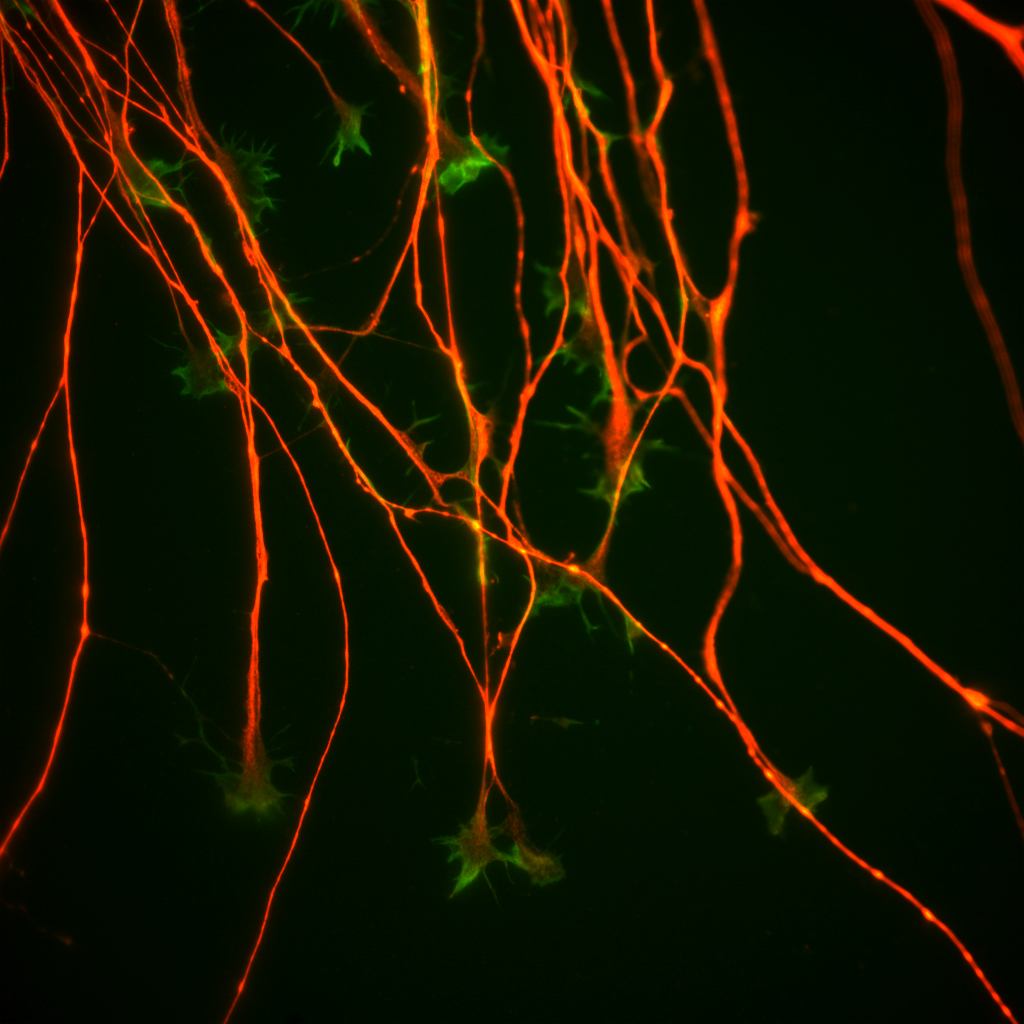

Supplement: Supplementary file 13 — Source Data for Figure 3 [file EMMM-15-e17907-s005.zip › SourceData_Fig_3/Fig_3_Source_Data_images/3G/20220829_control_DRG_aumentos_2.lif_-_Series009_(RGB).tif]

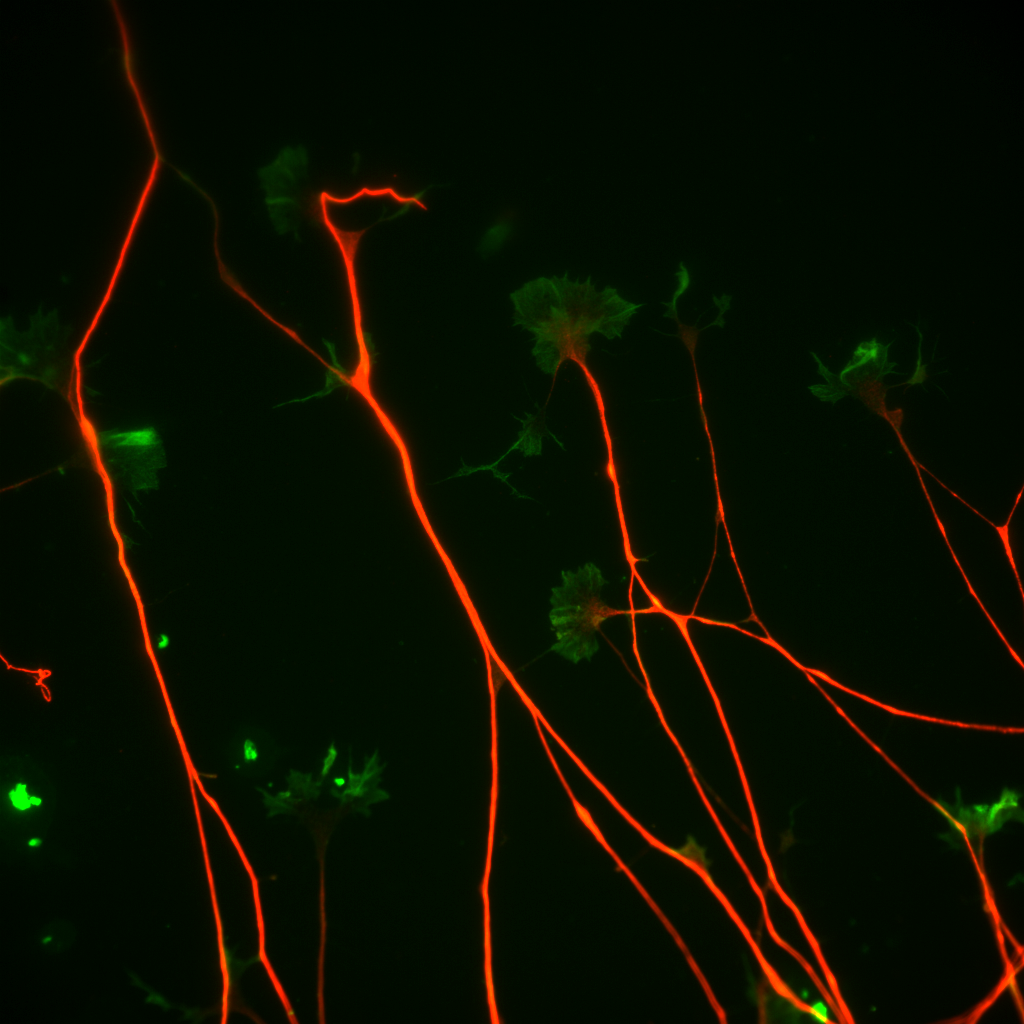

Supplement: Supplementary file 13 — Source Data for Figure 3 [file EMMM-15-e17907-s005.zip › SourceData_Fig_3/Fig_3_Source_Data_images/3G/20220829_rcm_DRG_aumentos.lif_-_Series009_(RGB).tif]

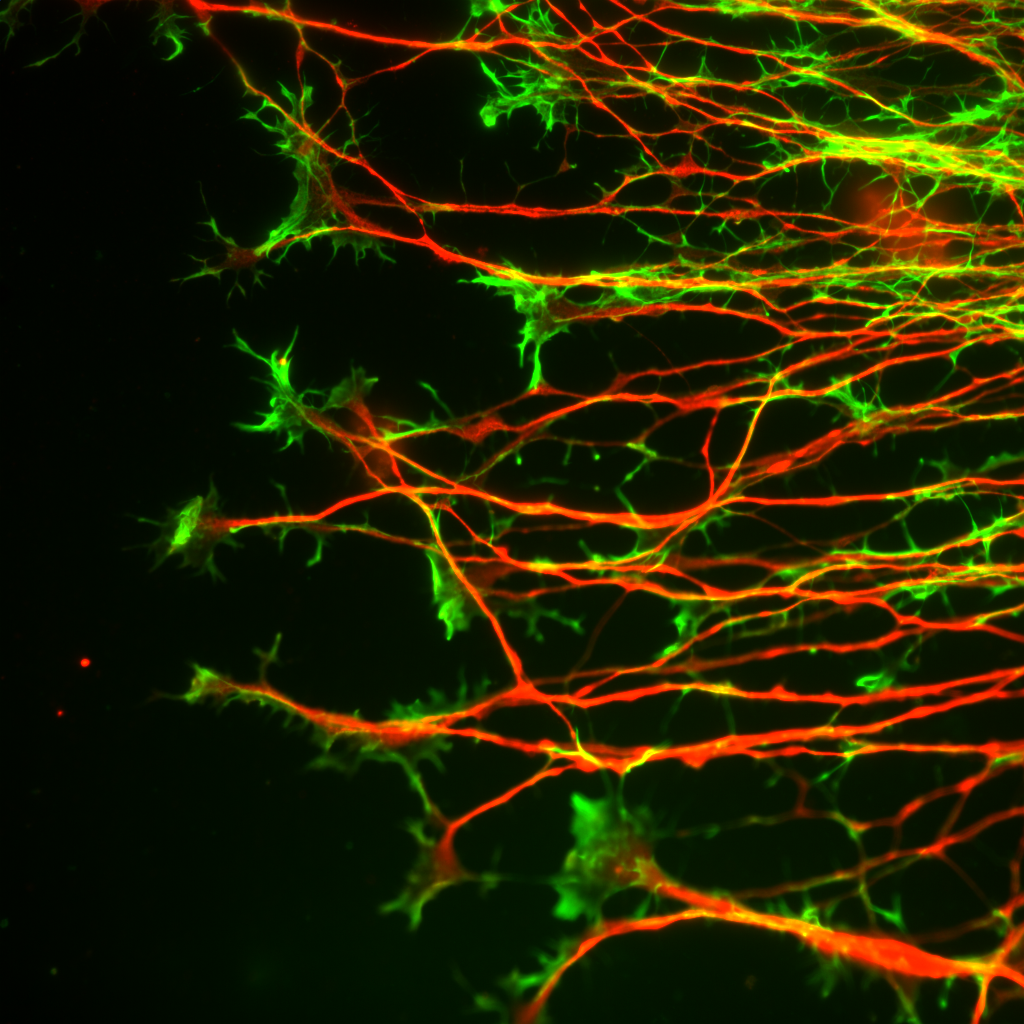

Supplement: Supplementary file 13 — Source Data for Figure 3 [file EMMM-15-e17907-s005.zip › SourceData_Fig_3/Fig_3_Source_Data_images/3G/20220829_Scm_DRG_aumentos_2.lif_-_Series002_(RGB).tif]

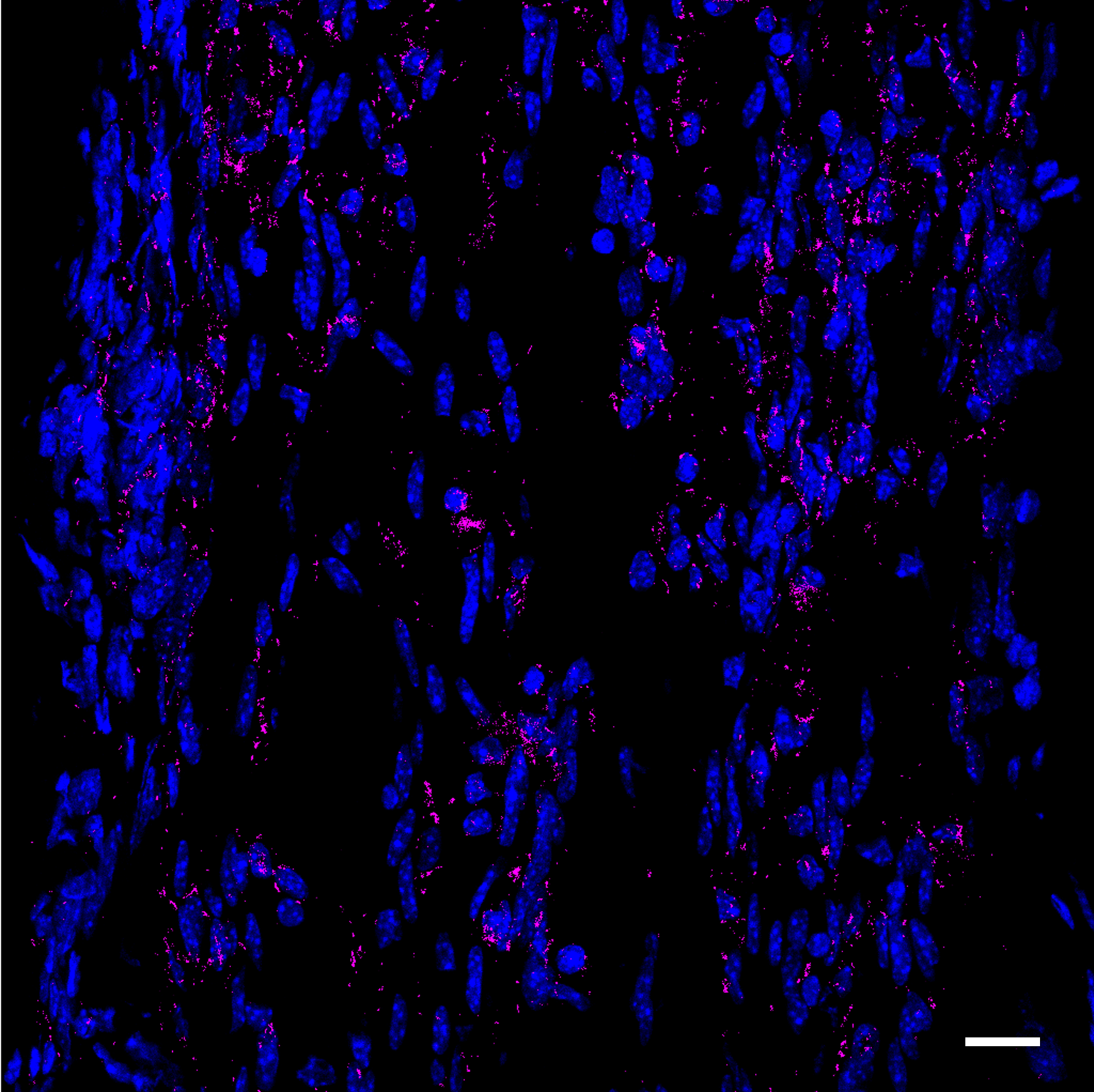

Supplement: Supplementary file 14 — Source Data for Figure 4 [file EMMM-15-e17907-s014.zip › SourceData_Fig_4/Fig_4_SourceData_images/3B/BGAL_ADULT_42_ABT_ALL.tif]

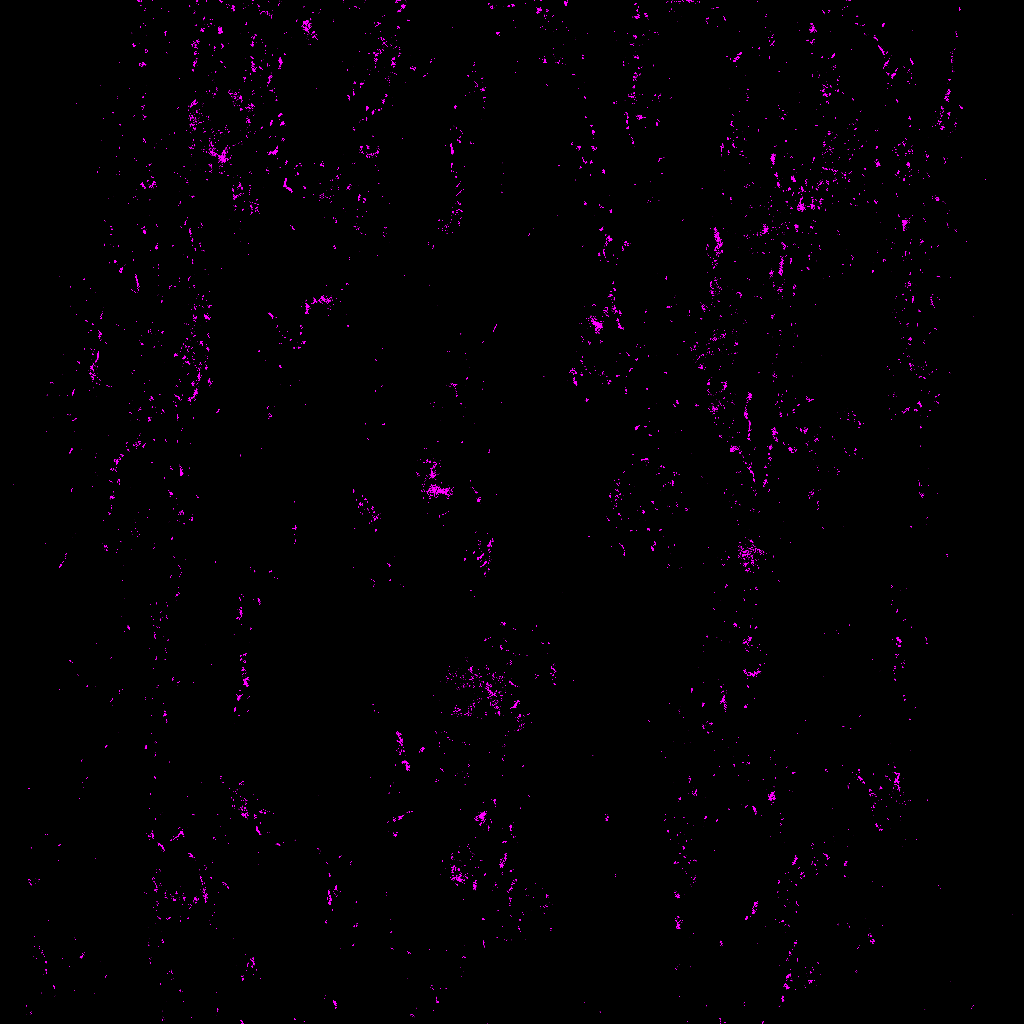

Supplement: Supplementary file 14 — Source Data for Figure 4 [file EMMM-15-e17907-s014.zip › SourceData_Fig_4/Fig_4_SourceData_images/3B/BGAL_ADULT_42_ABT_MAG.tif]

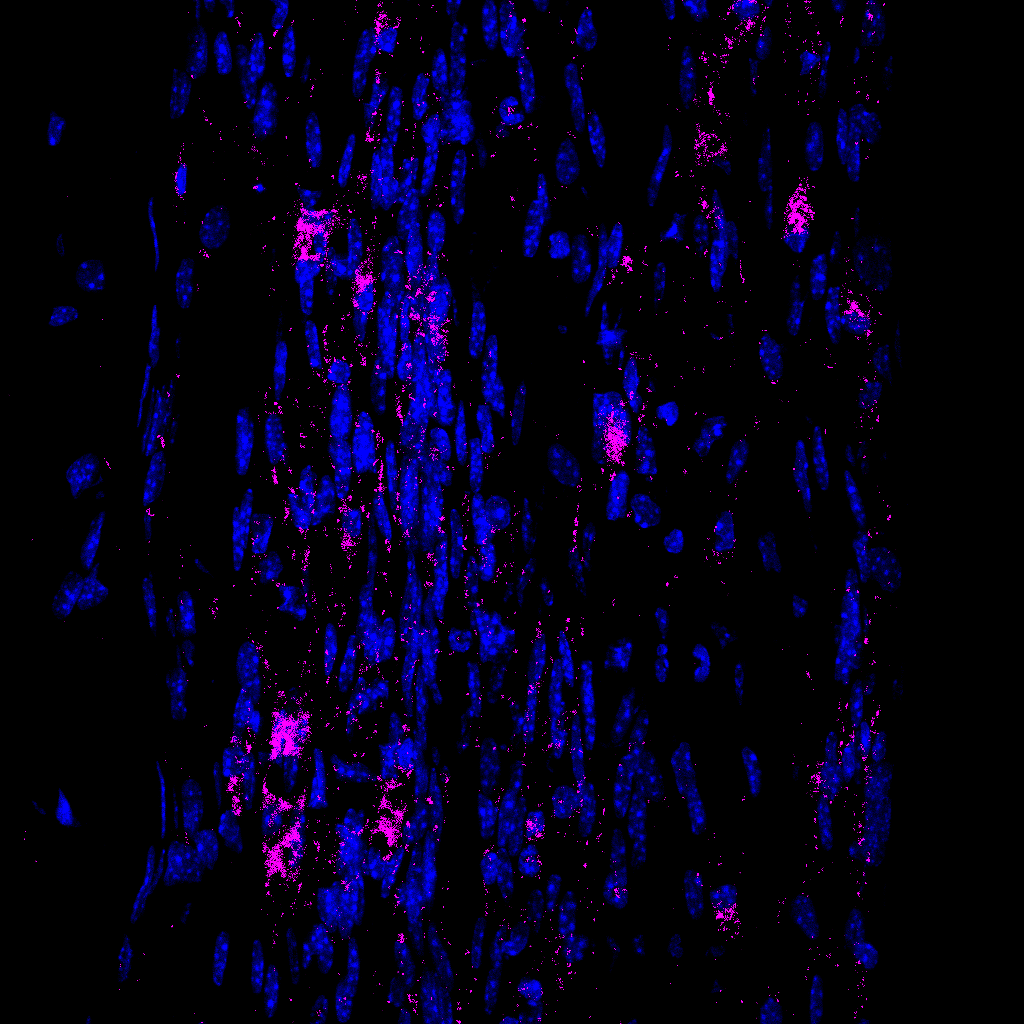

Supplement: Supplementary file 14 — Source Data for Figure 4 [file EMMM-15-e17907-s014.zip › SourceData_Fig_4/Fig_4_SourceData_images/3B/BGAL_ADULT_42_ALL.tif]

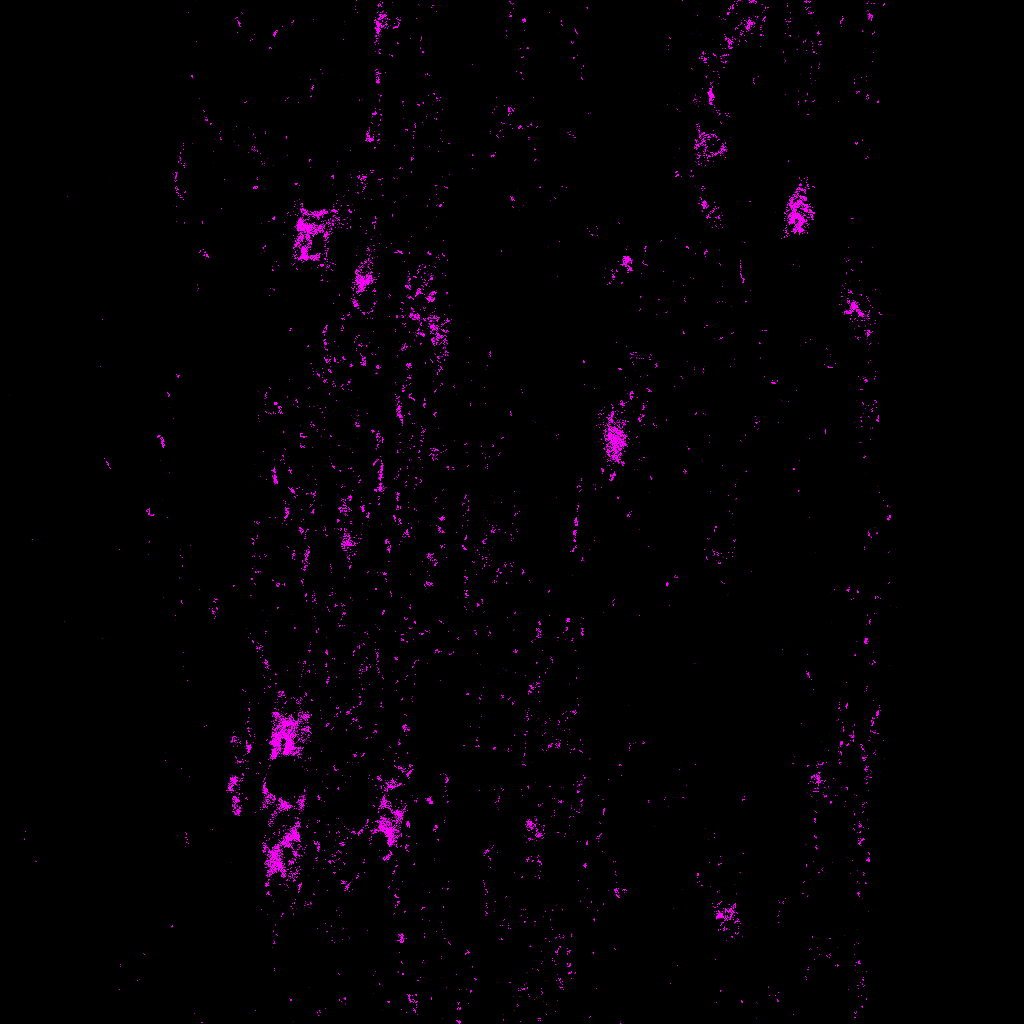

Supplement: Supplementary file 14 — Source Data for Figure 4 [file EMMM-15-e17907-s014.zip › SourceData_Fig_4/Fig_4_SourceData_images/3B/BGAL_ADULT_42_MAG.tif]

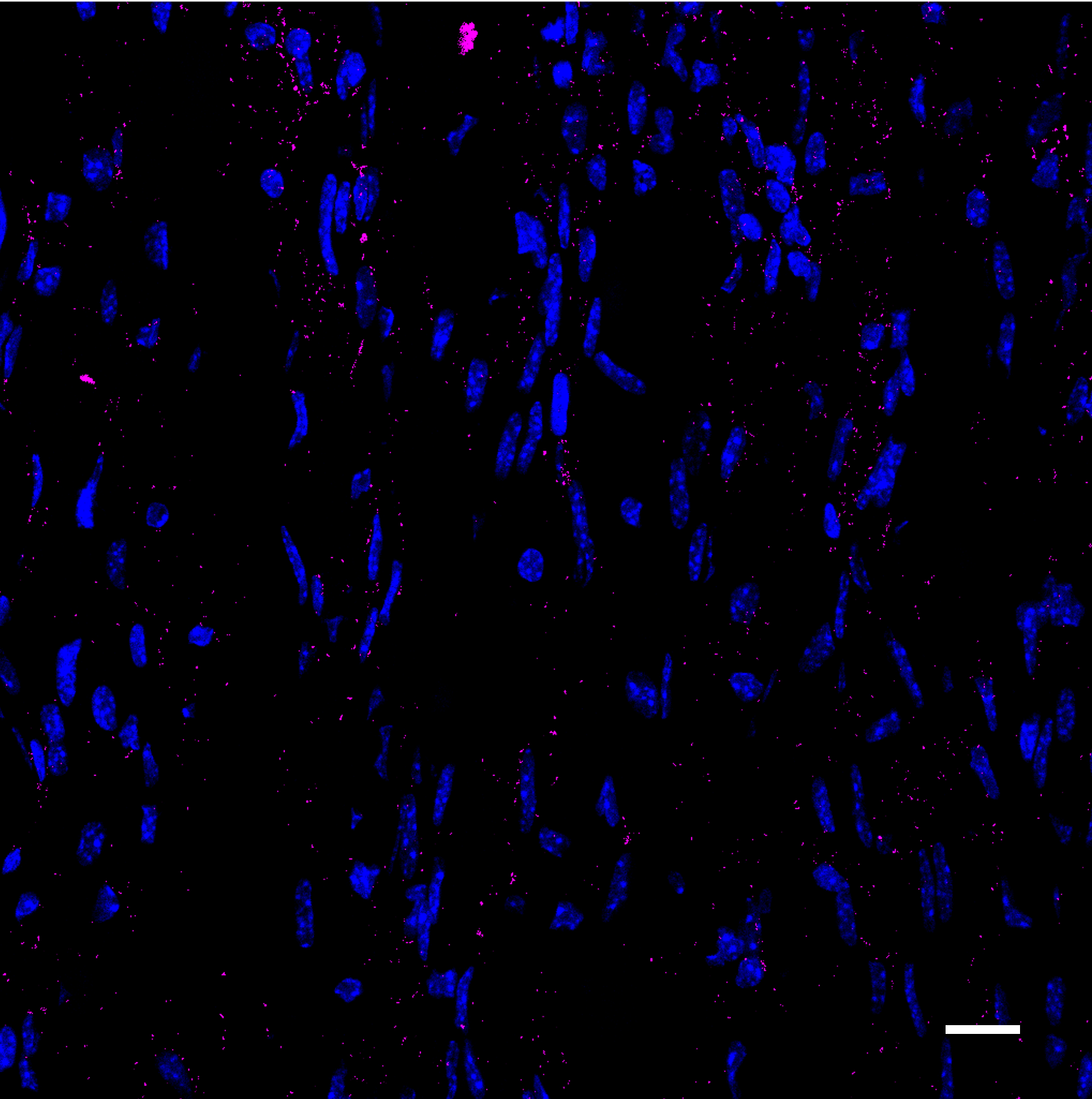

Supplement: Supplementary file 14 — Source Data for Figure 4 [file EMMM-15-e17907-s014.zip › SourceData_Fig_4/Fig_4_SourceData_images/3B/BGAL_AGED_12_ABT_ALL.tif]

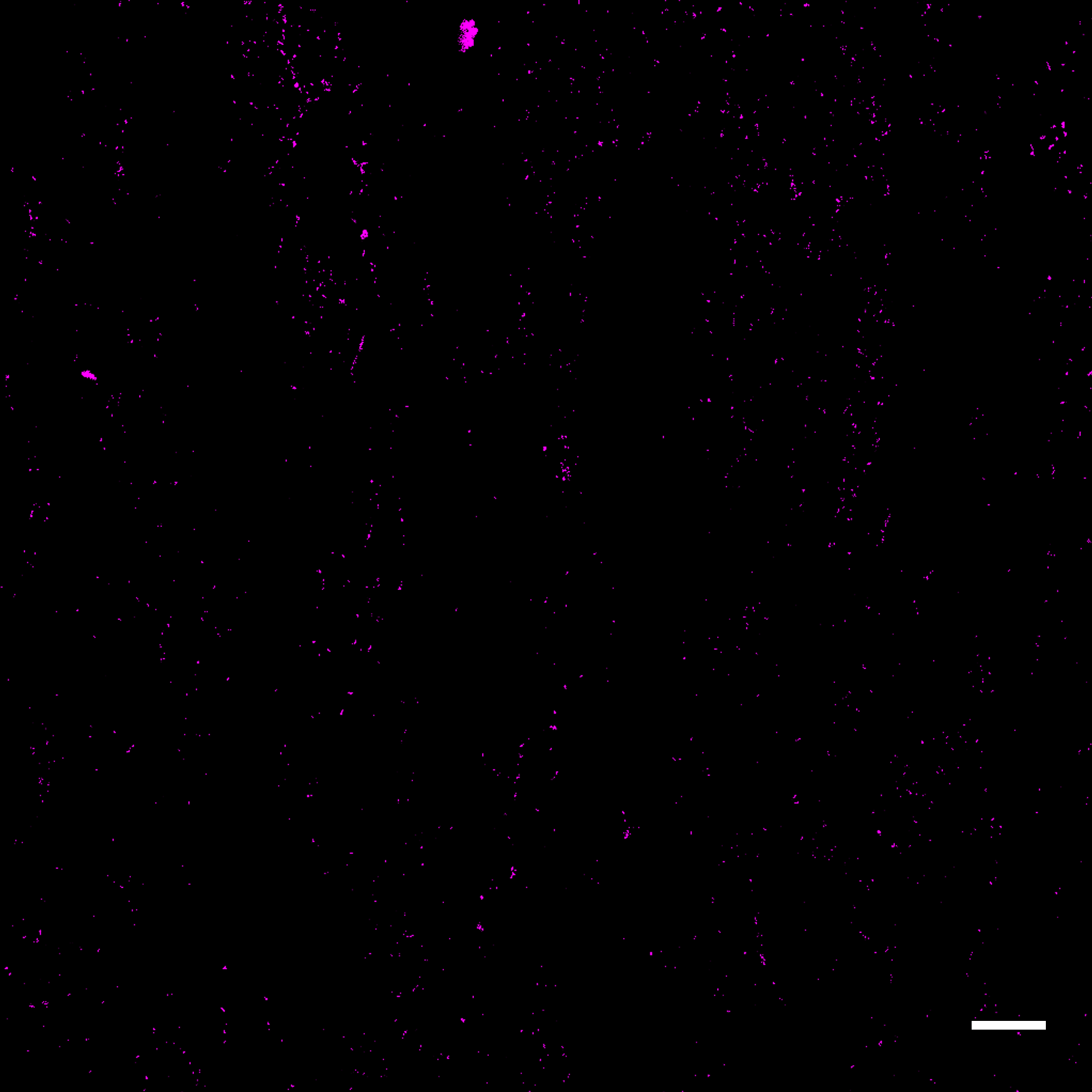

Supplement: Supplementary file 14 — Source Data for Figure 4 [file EMMM-15-e17907-s014.zip › SourceData_Fig_4/Fig_4_SourceData_images/3B/BGAL_AGED_12_ABT_MAG.tif]

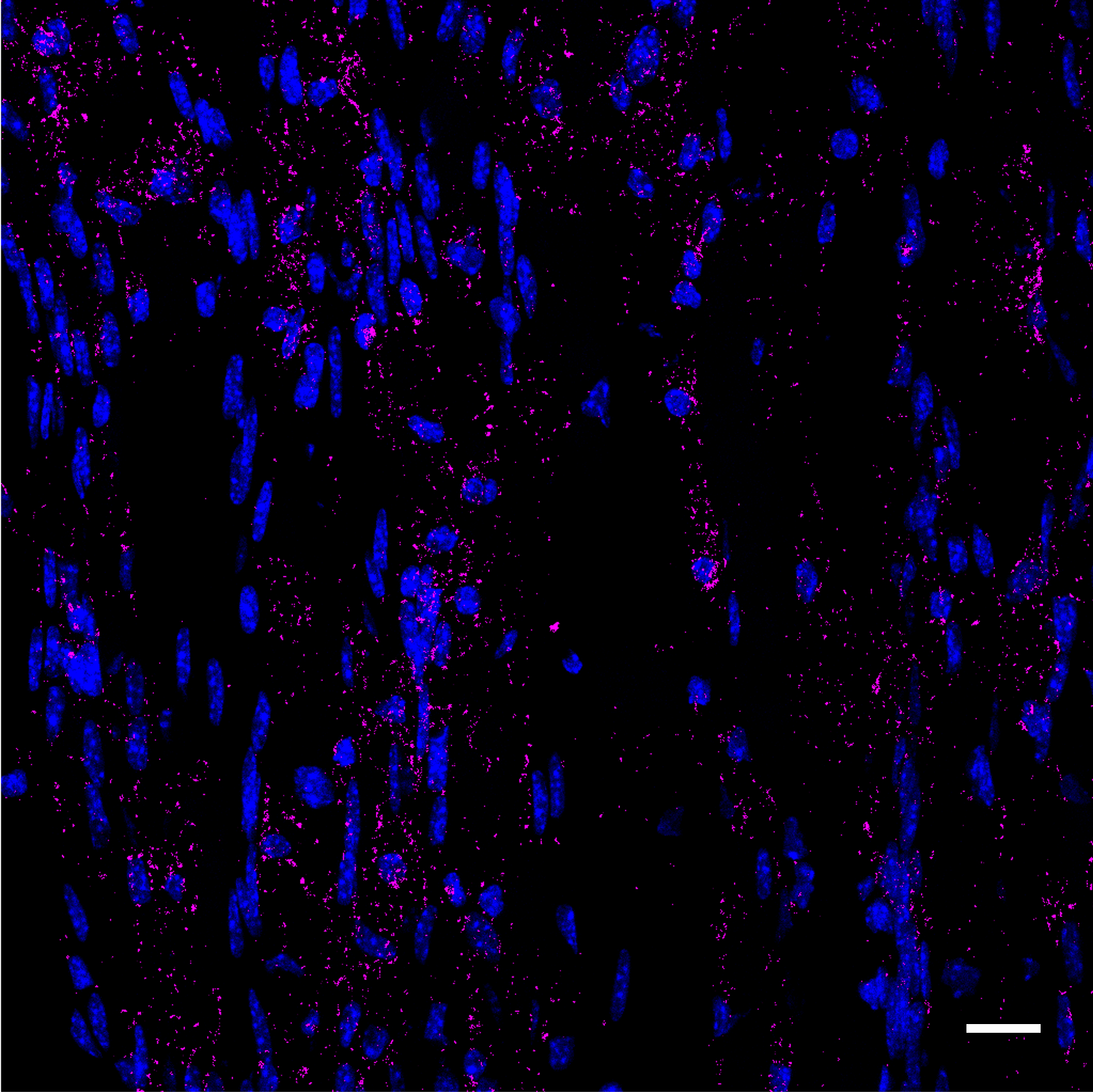

Supplement: Supplementary file 14 — Source Data for Figure 4 [file EMMM-15-e17907-s014.zip › SourceData_Fig_4/Fig_4_SourceData_images/3B/BGAL_AGED_12_VEH_ALL.tif]

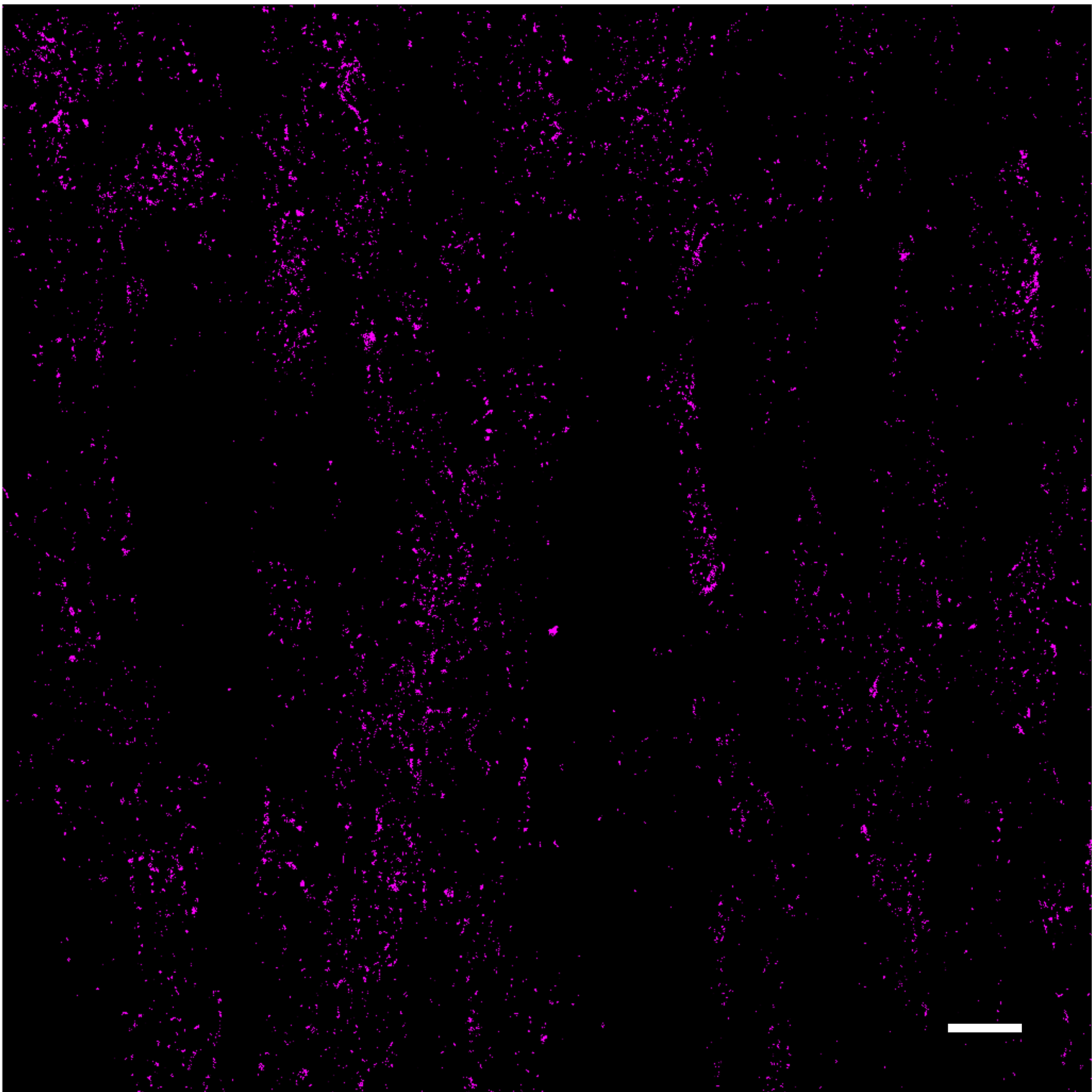

Supplement: Supplementary file 14 — Source Data for Figure 4 [file EMMM-15-e17907-s014.zip › SourceData_Fig_4/Fig_4_SourceData_images/3B/BGAL_AGED_12_VEH_MAG.tif]

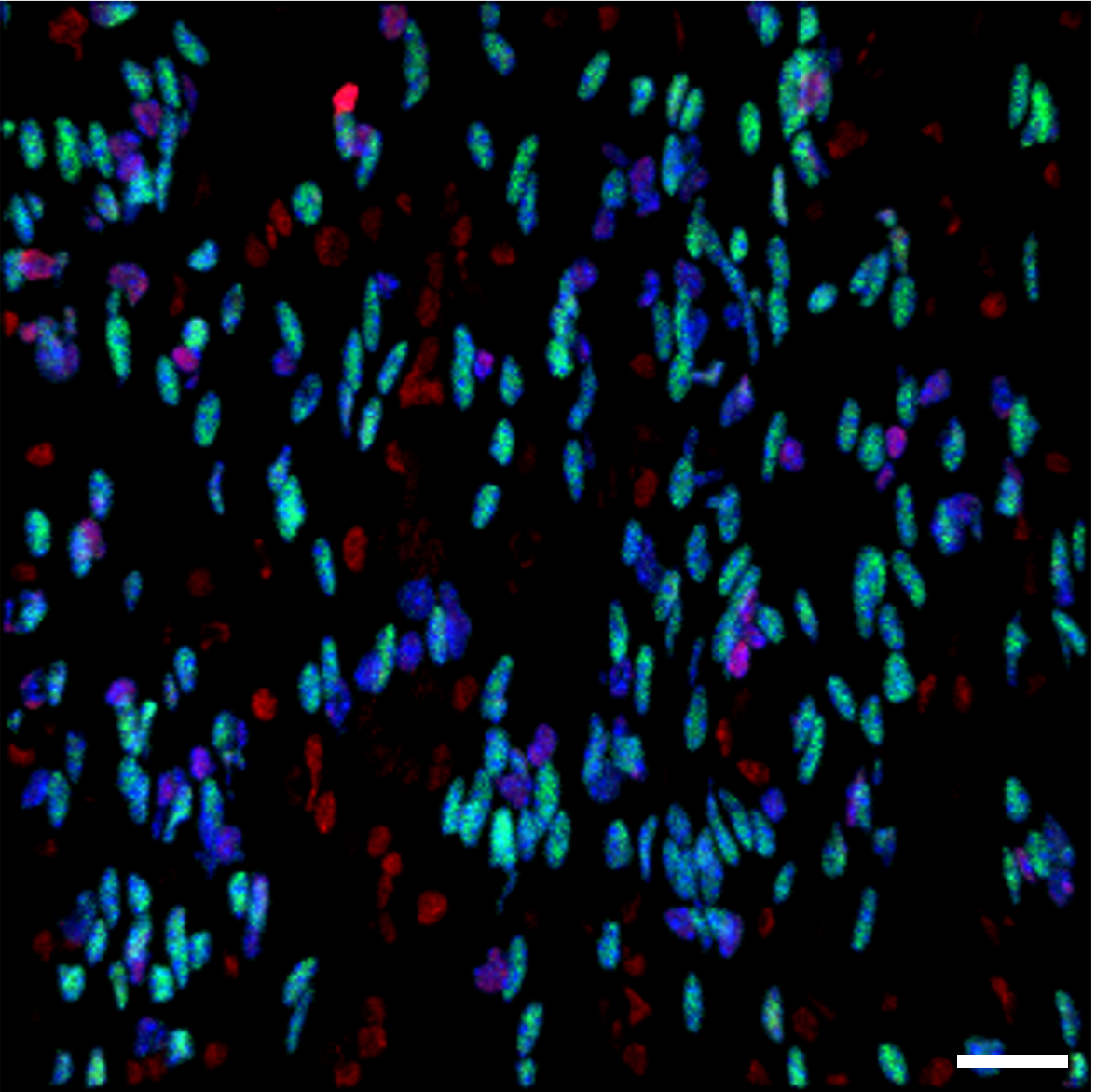

Supplement: Supplementary file 14 — Source Data for Figure 4 [file EMMM-15-e17907-s014.zip › SourceData_Fig_4/Fig_4_SourceData_images/3D/Adult_42_ABT_all.tif]

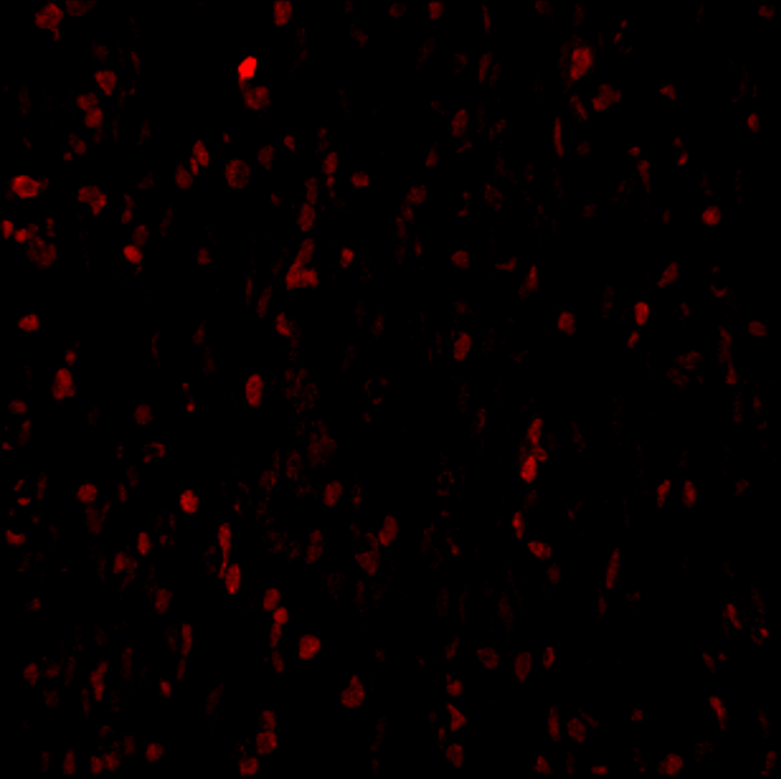

Supplement: Supplementary file 14 — Source Data for Figure 4 [file EMMM-15-e17907-s014.zip › SourceData_Fig_4/Fig_4_SourceData_images/3D/Adult_42_ABT_red.tif]

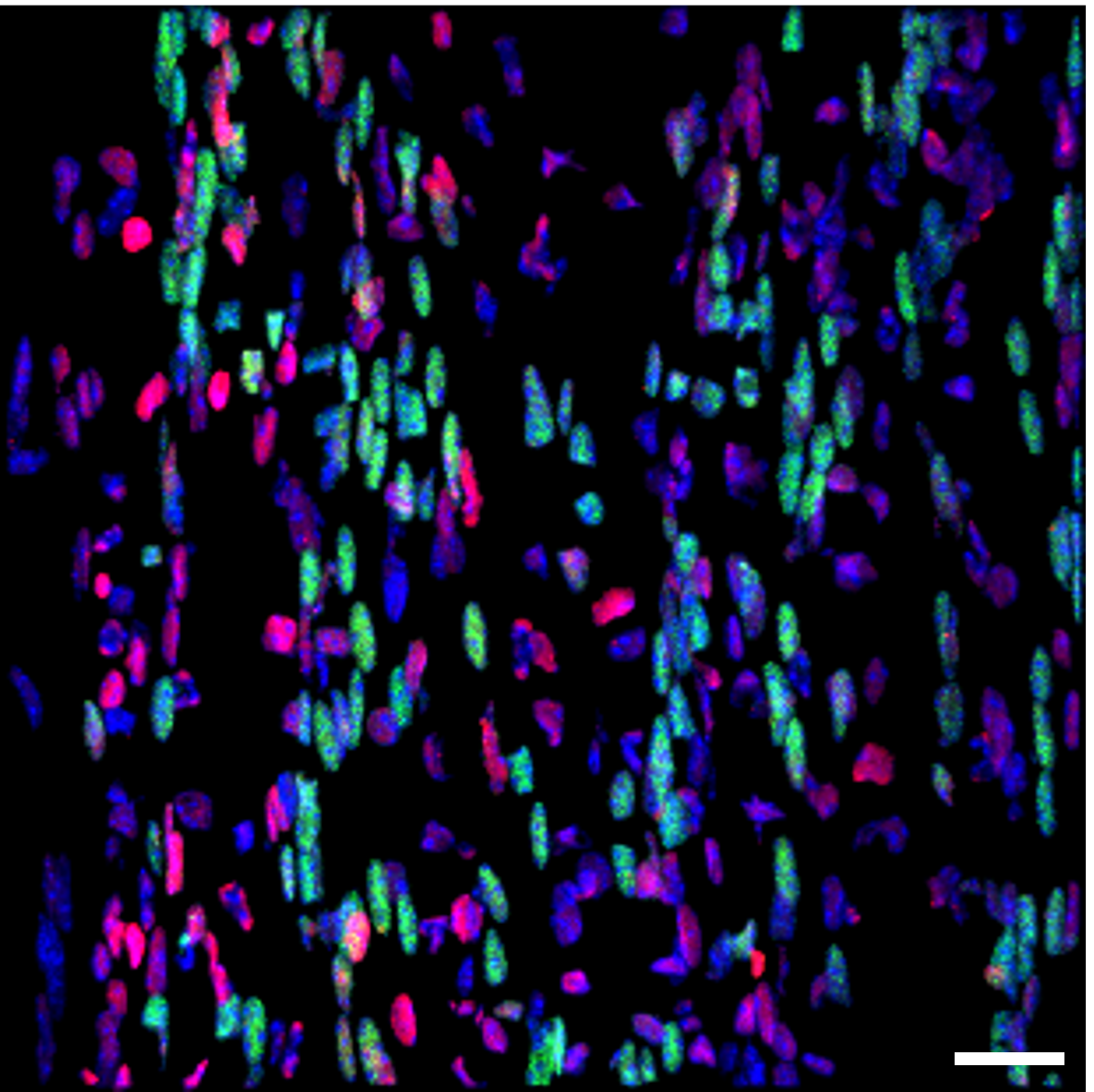

Supplement: Supplementary file 14 — Source Data for Figure 4 [file EMMM-15-e17907-s014.zip › SourceData_Fig_4/Fig_4_SourceData_images/3D/Adult_42_veh_all.tif]

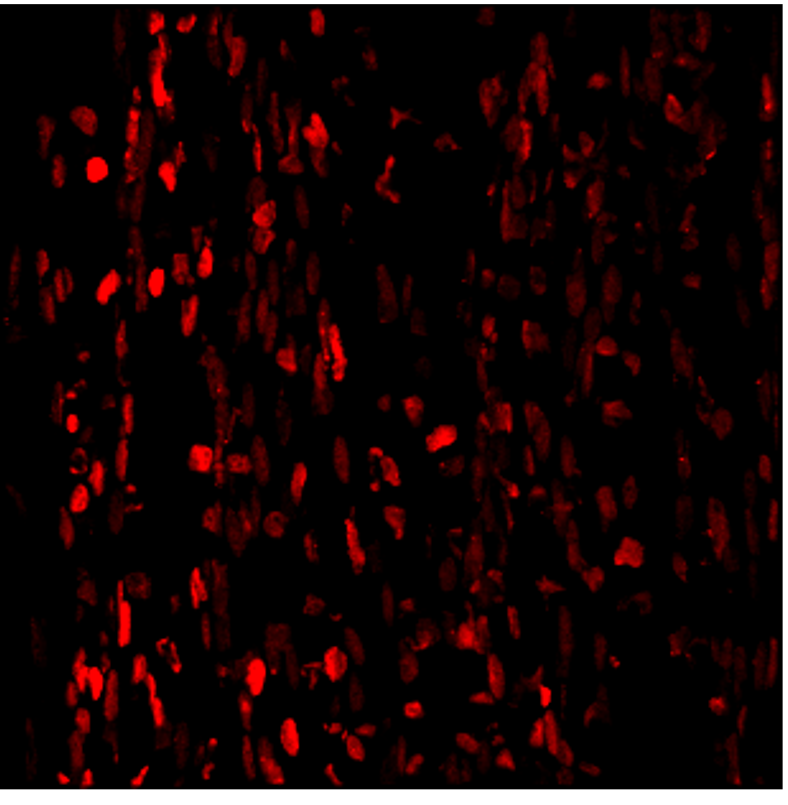

Supplement: Supplementary file 14 — Source Data for Figure 4 [file EMMM-15-e17907-s014.zip › SourceData_Fig_4/Fig_4_SourceData_images/3D/Adult_42_veh_red.tif]

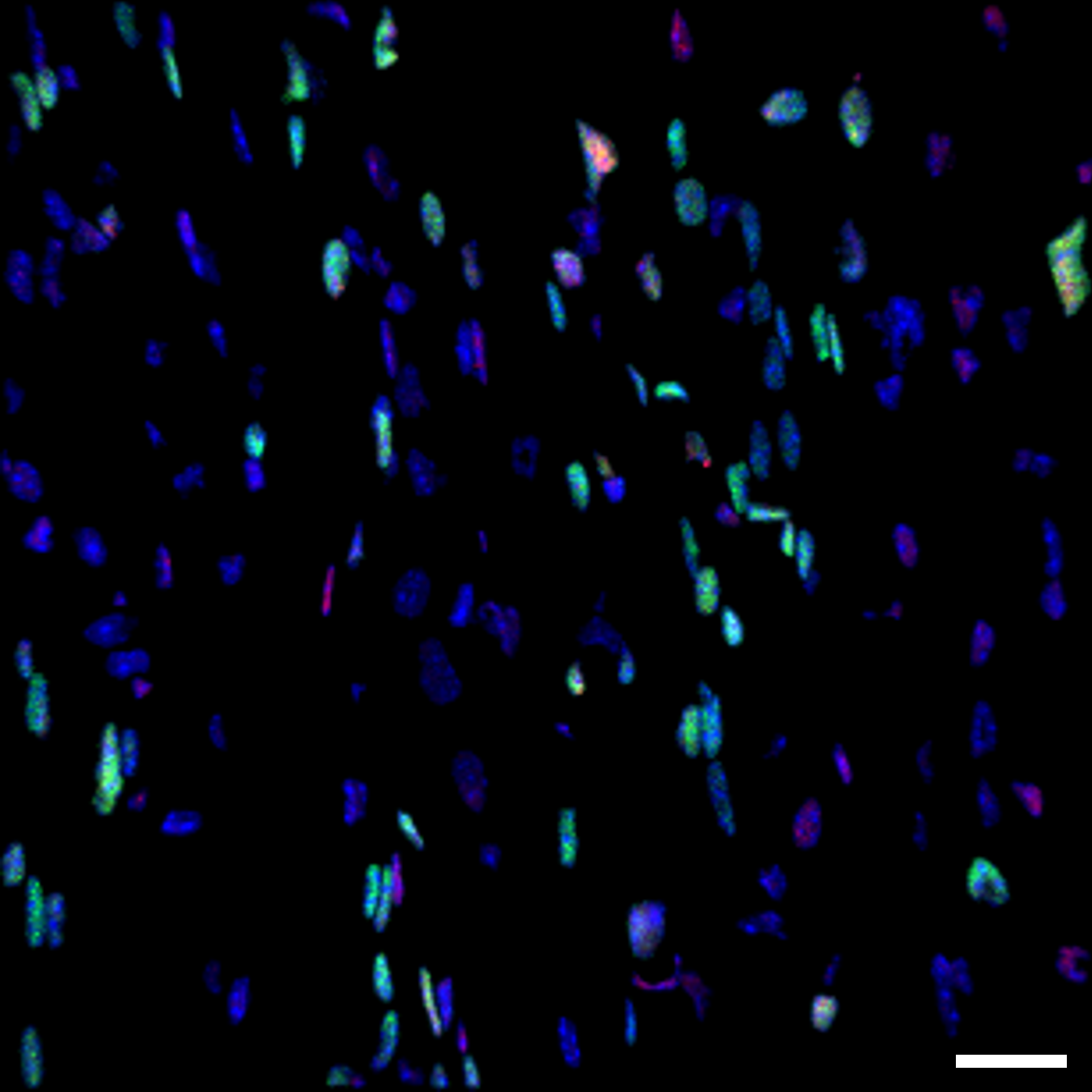

Supplement: Supplementary file 14 — Source Data for Figure 4 [file EMMM-15-e17907-s014.zip › SourceData_Fig_4/Fig_4_SourceData_images/3D/Aged_12_ABT_all.tif]

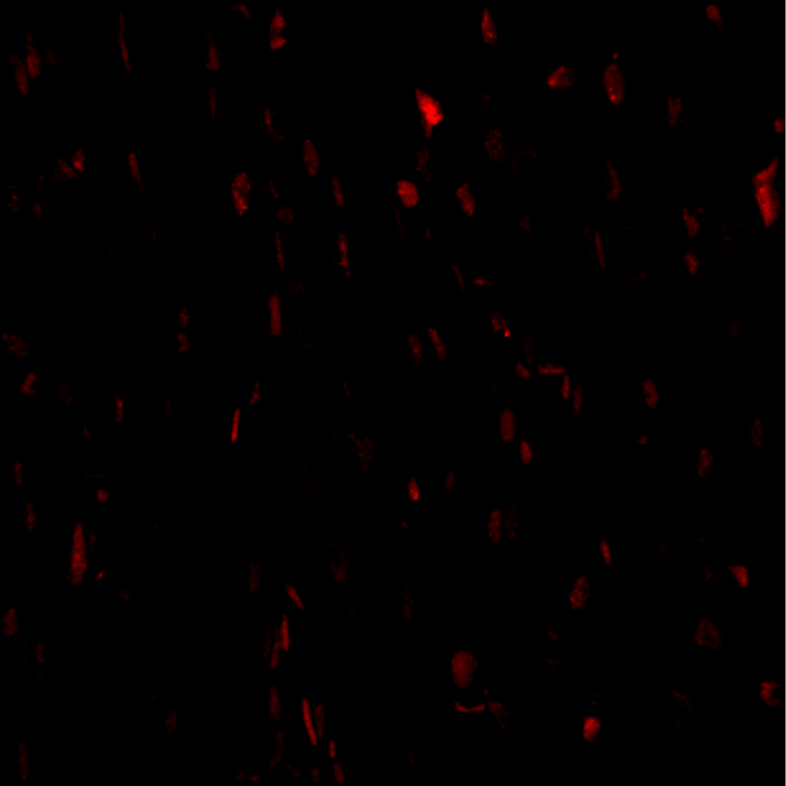

Supplement: Supplementary file 14 — Source Data for Figure 4 [file EMMM-15-e17907-s014.zip › SourceData_Fig_4/Fig_4_SourceData_images/3D/Aged_12_ABT_red.tif]

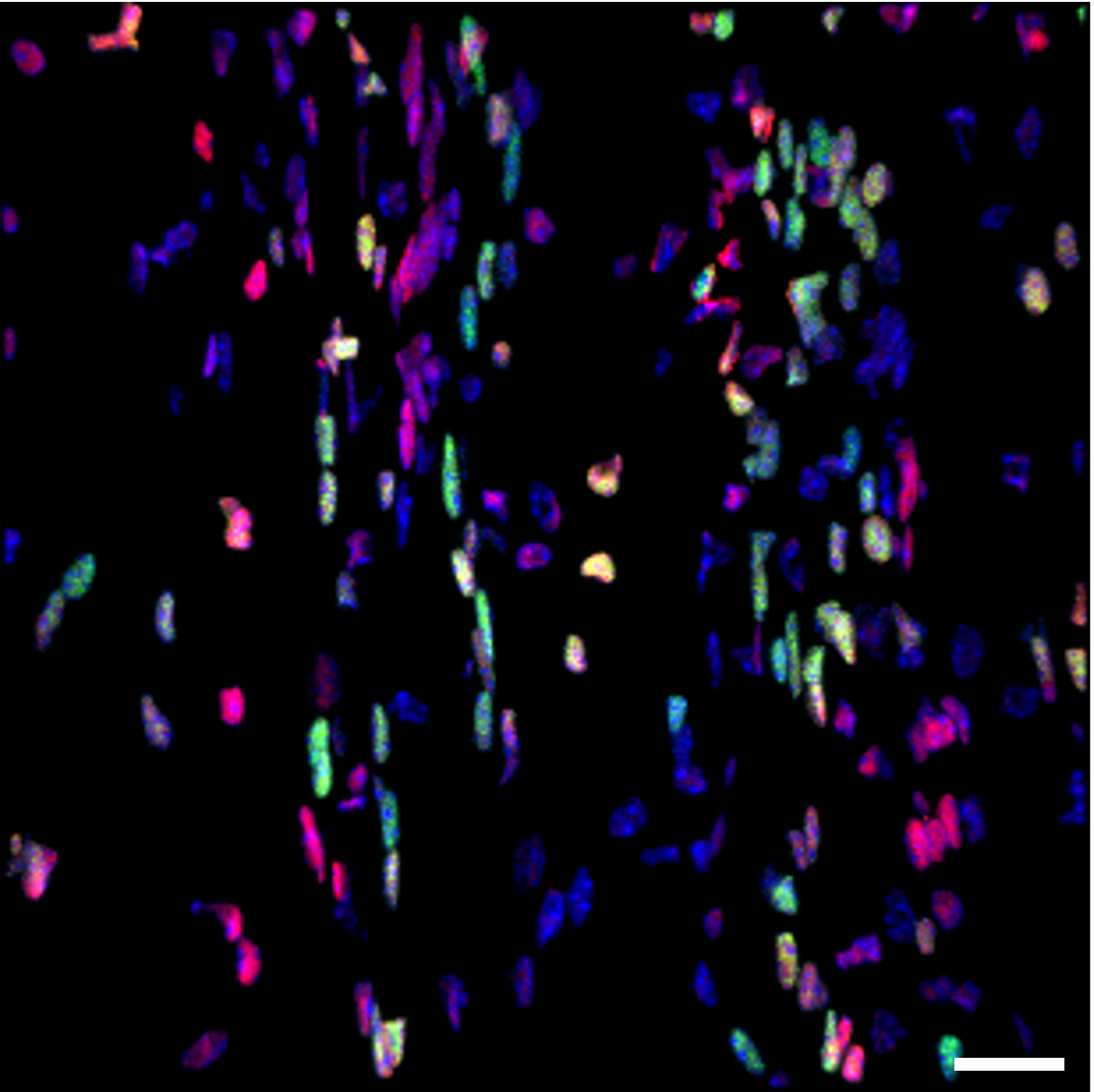

Supplement: Supplementary file 14 — Source Data for Figure 4 [file EMMM-15-e17907-s014.zip › SourceData_Fig_4/Fig_4_SourceData_images/3D/Aged_12_veh_all.tif]

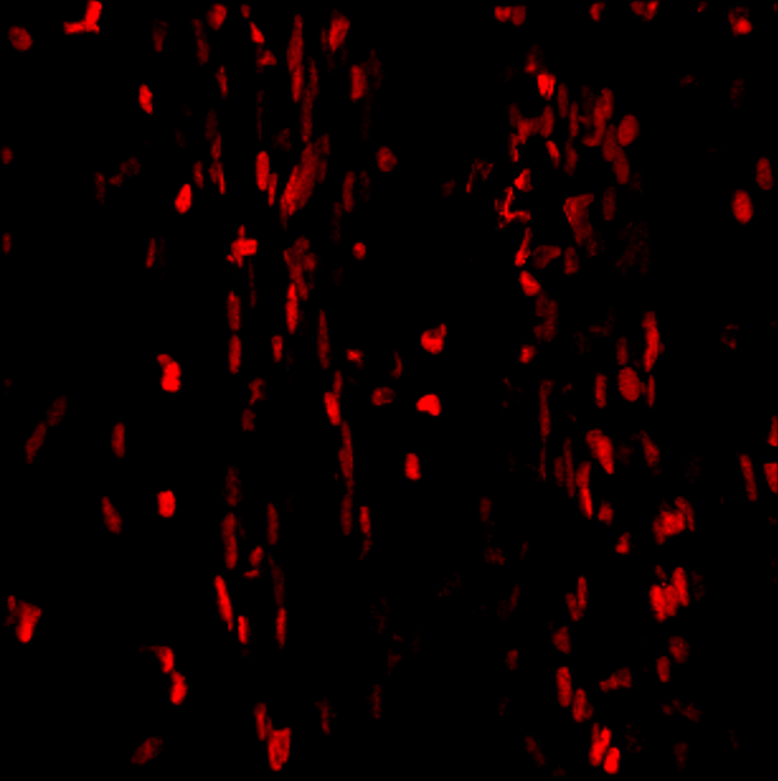

Supplement: Supplementary file 14 — Source Data for Figure 4 [file EMMM-15-e17907-s014.zip › SourceData_Fig_4/Fig_4_SourceData_images/3D/Aged_12_veh_red.tif]

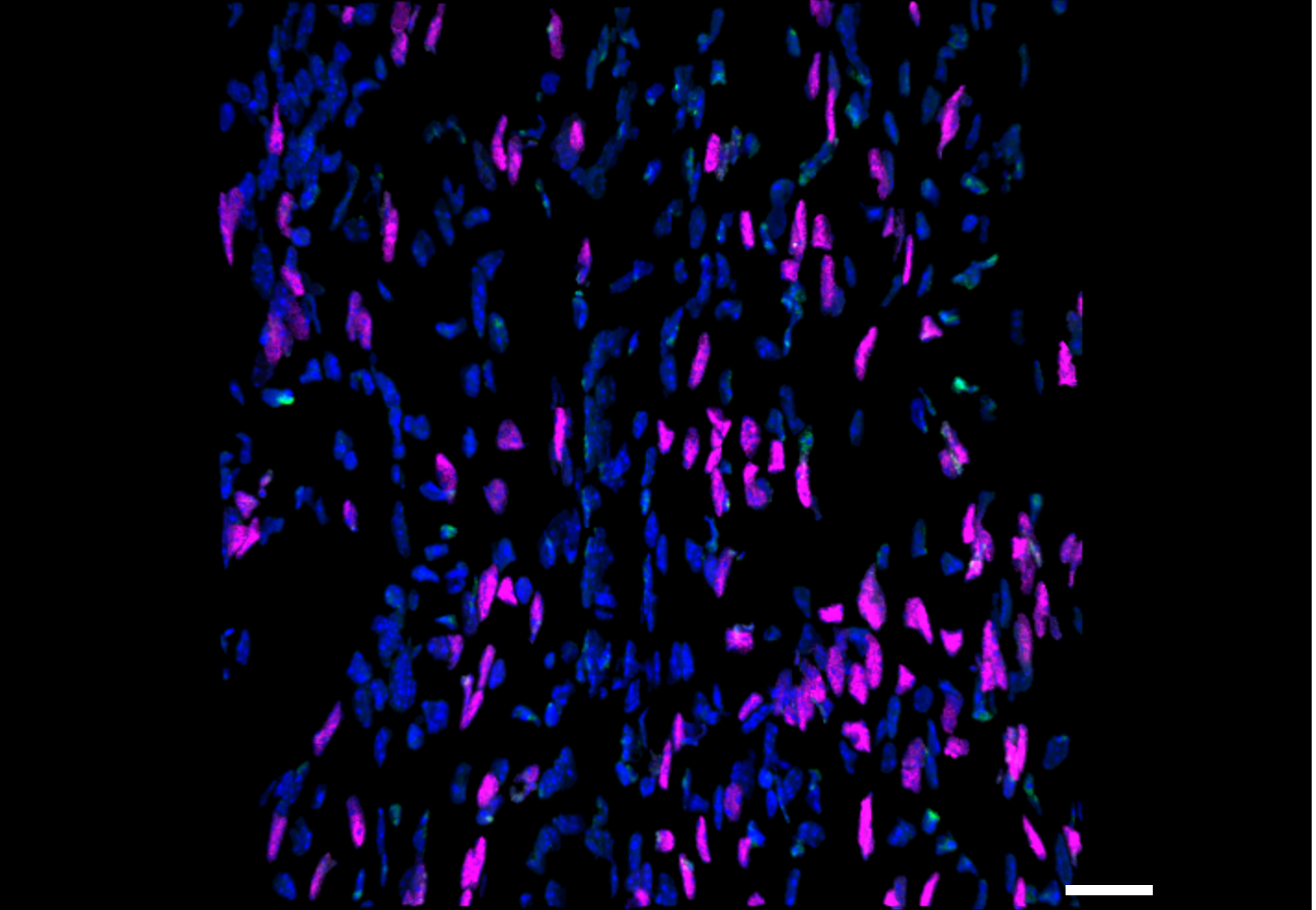

Supplement: Supplementary file 14 — Source Data for Figure 4 [file EMMM-15-e17907-s014.zip › SourceData_Fig_4/Fig_4_SourceData_images/3F/P16_ADULT_42_ABT_ALL.tif]

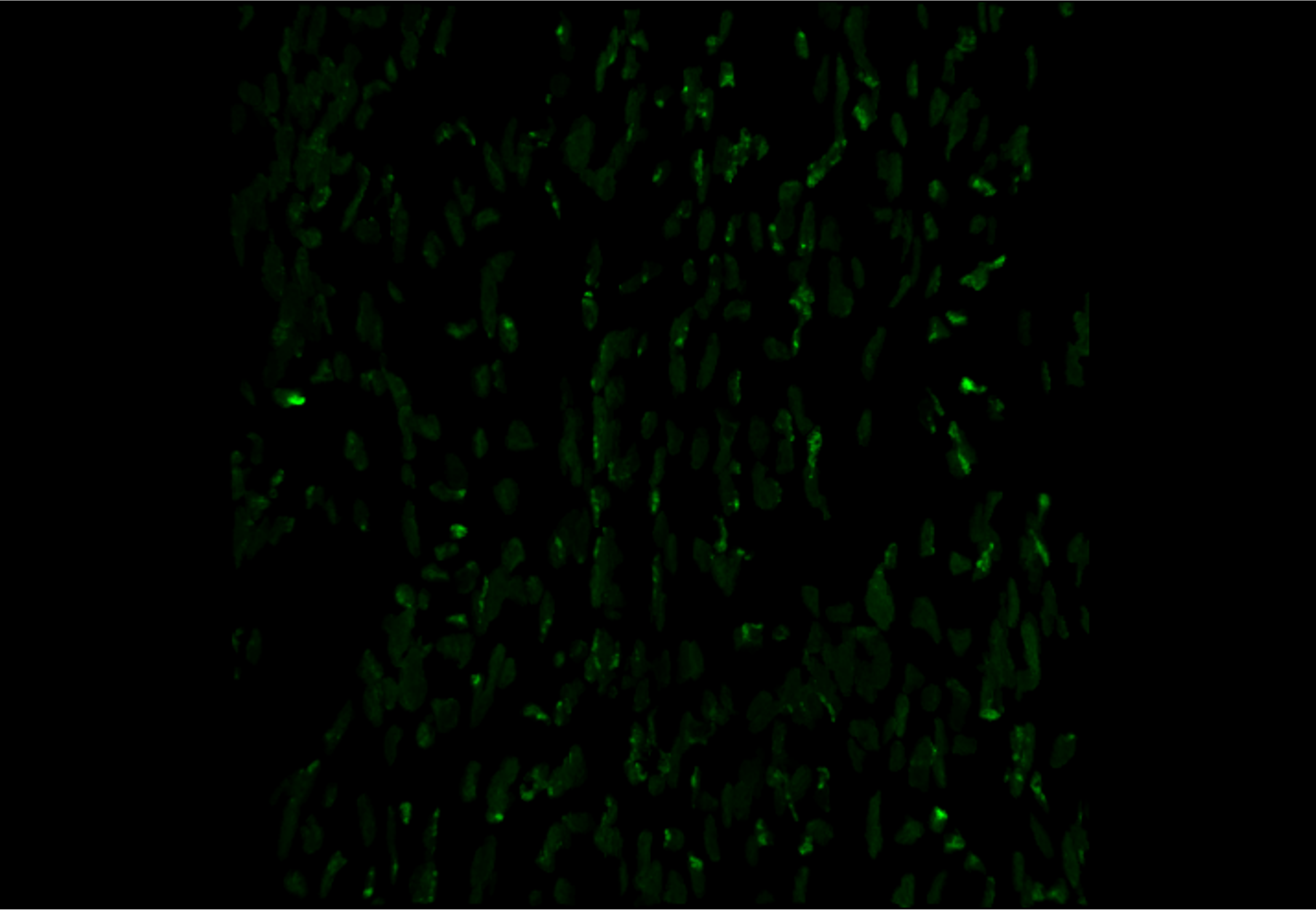

Supplement: Supplementary file 14 — Source Data for Figure 4 [file EMMM-15-e17907-s014.zip › SourceData_Fig_4/Fig_4_SourceData_images/3F/P16_ADULT_42_ABT_GREEN.tif]

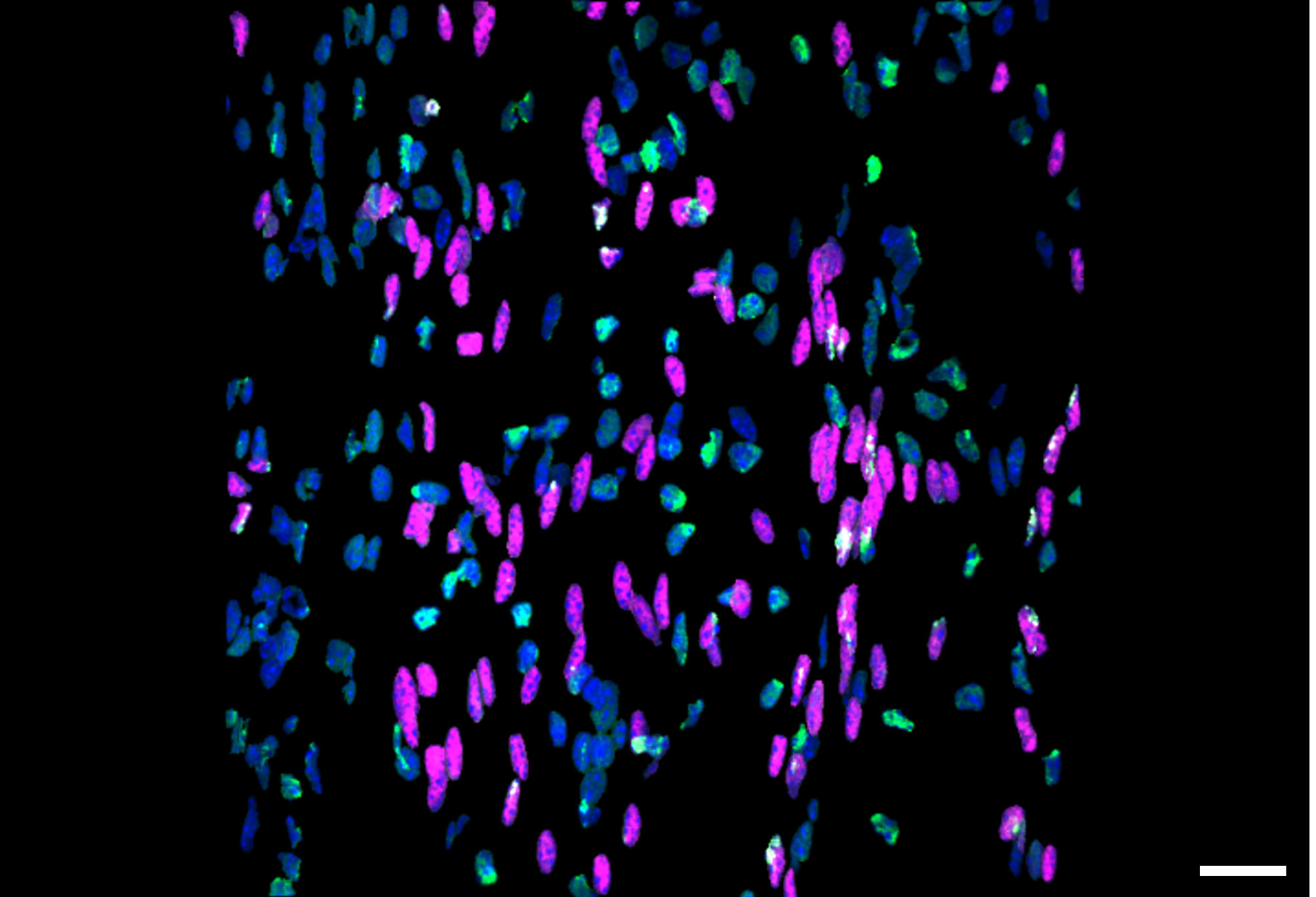

Supplement: Supplementary file 14 — Source Data for Figure 4 [file EMMM-15-e17907-s014.zip › SourceData_Fig_4/Fig_4_SourceData_images/3F/P16_ADULT_42_VEH_ALL.tif]

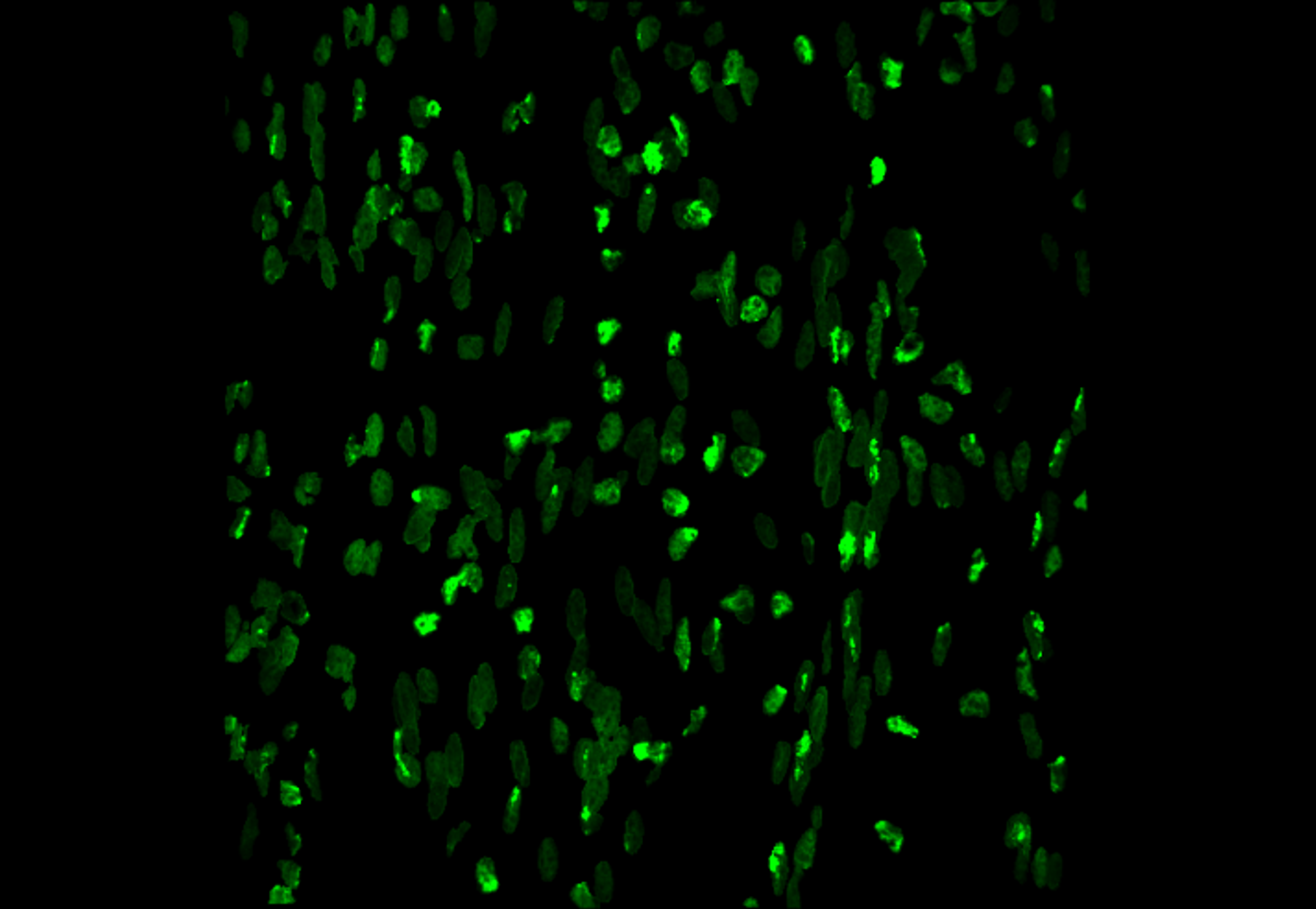

Supplement: Supplementary file 14 — Source Data for Figure 4 [file EMMM-15-e17907-s014.zip › SourceData_Fig_4/Fig_4_SourceData_images/3F/P16_ADULT_42_VEH_GREEN.tif]

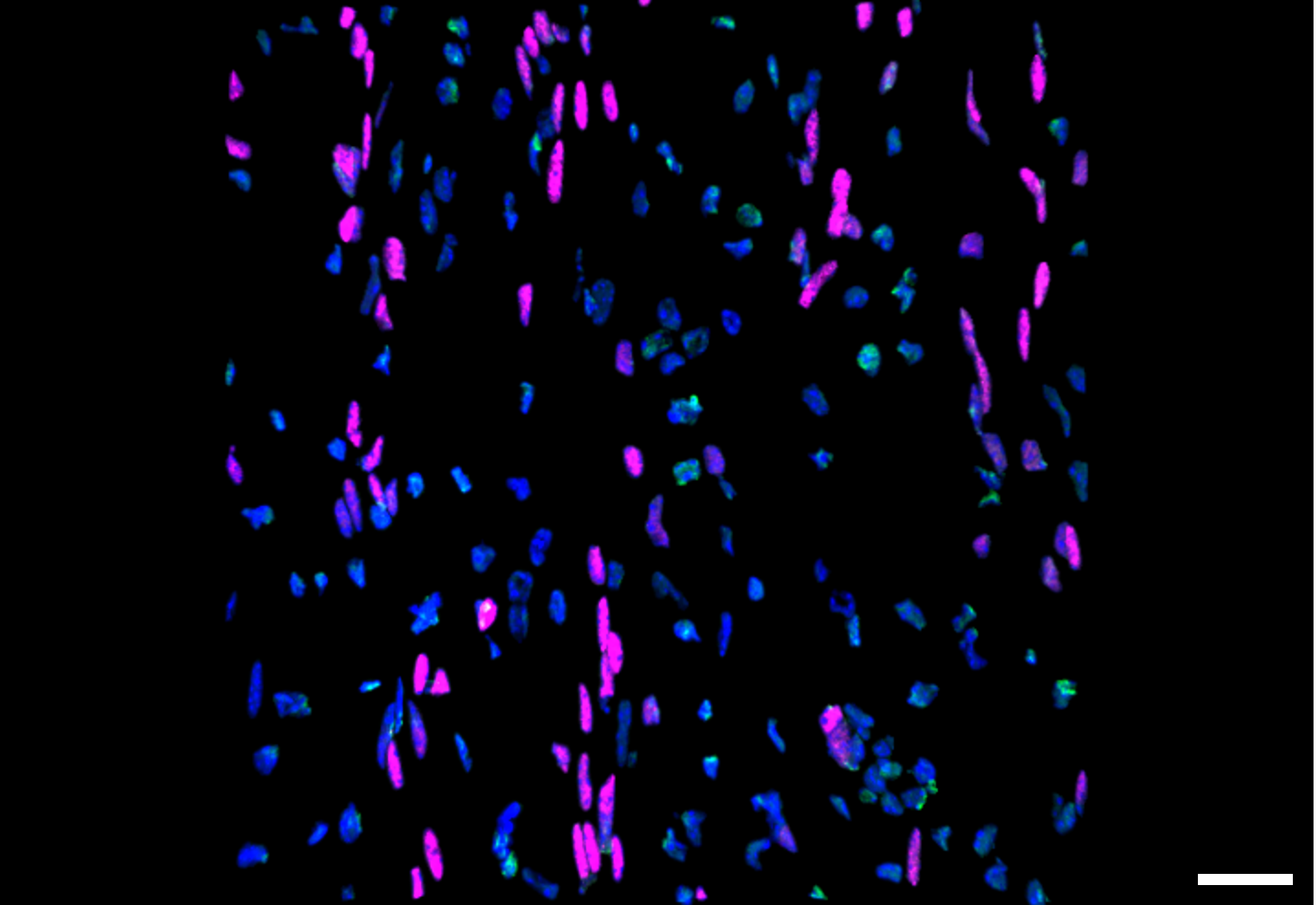

Supplement: Supplementary file 14 — Source Data for Figure 4 [file EMMM-15-e17907-s014.zip › SourceData_Fig_4/Fig_4_SourceData_images/3F/P16_AGED_12_ABT_ALL.tif]

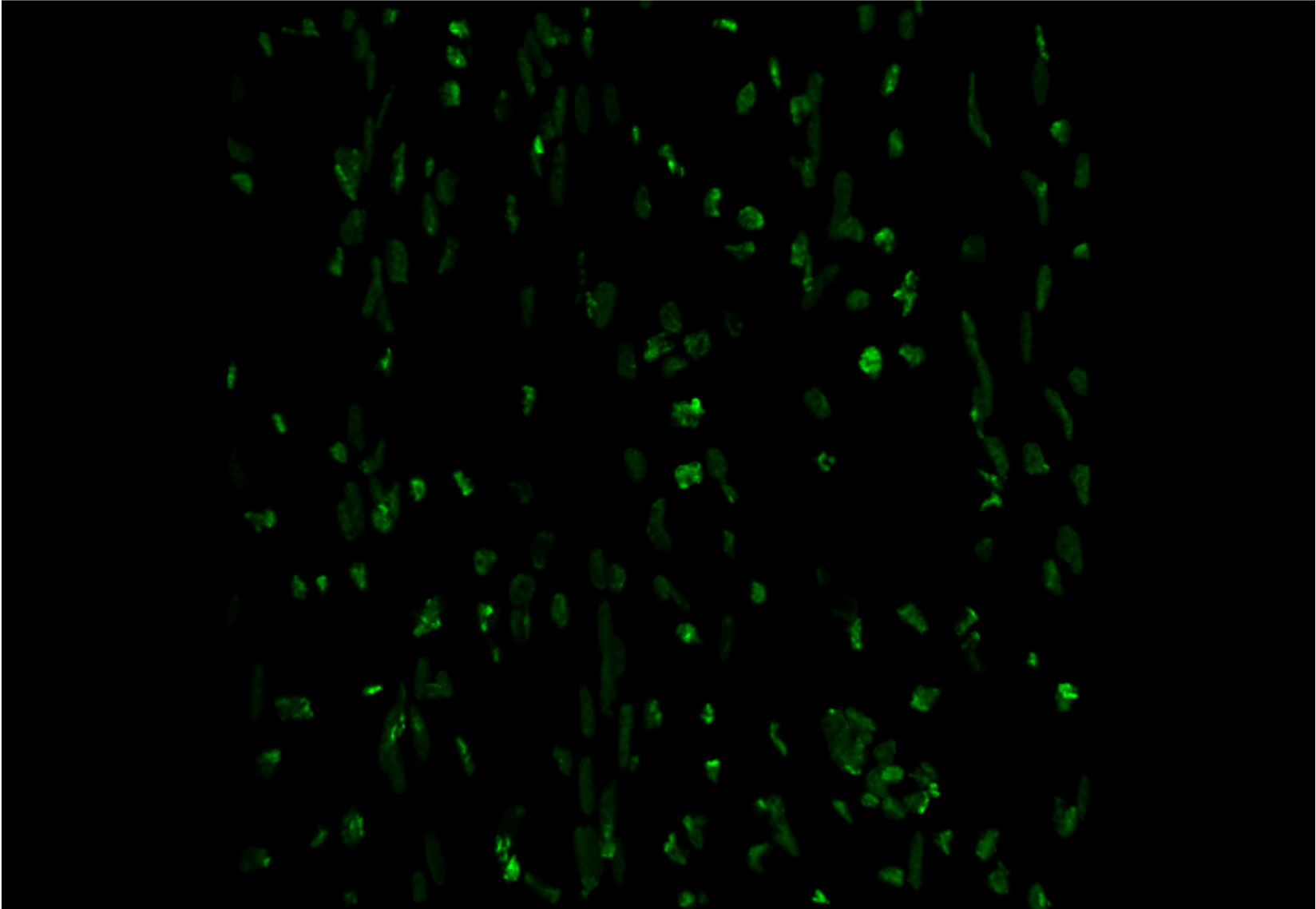

Supplement: Supplementary file 14 — Source Data for Figure 4 [file EMMM-15-e17907-s014.zip › SourceData_Fig_4/Fig_4_SourceData_images/3F/P16_AGED_12_ABT_GREEN.tif]

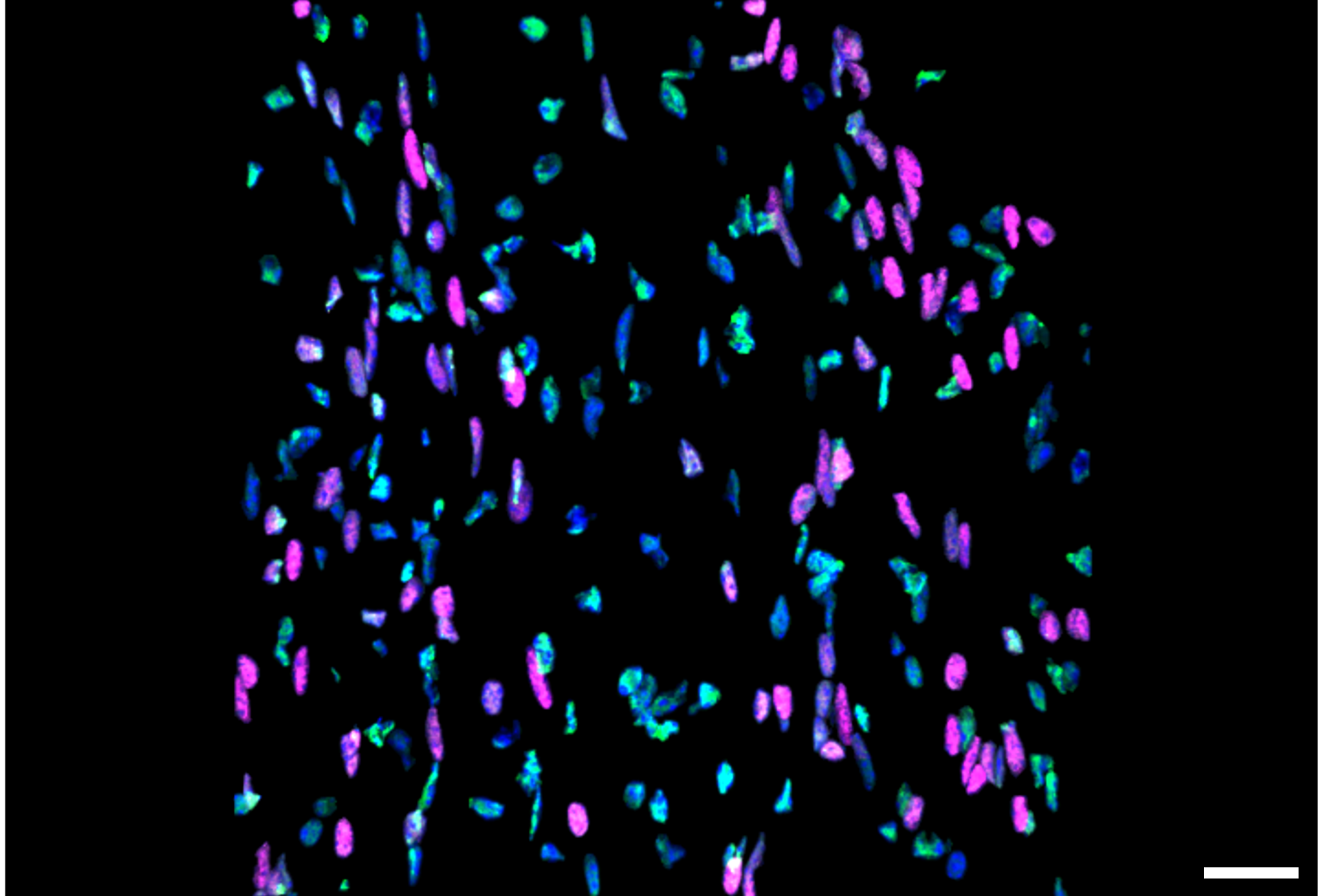

Supplement: Supplementary file 14 — Source Data for Figure 4 [file EMMM-15-e17907-s014.zip › SourceData_Fig_4/Fig_4_SourceData_images/3F/P16_AGED_12_VEH_ALL.tif]

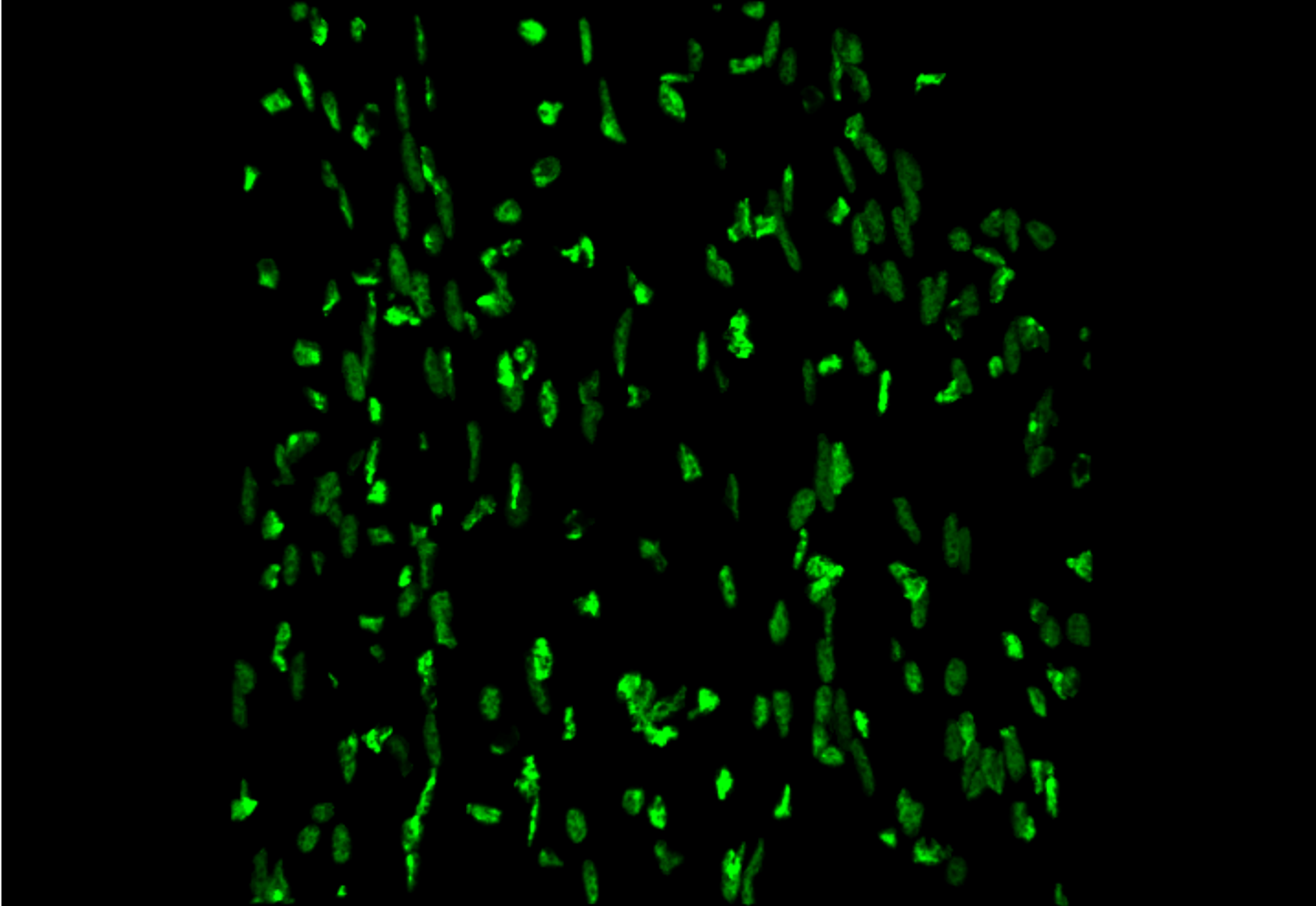

Supplement: Supplementary file 14 — Source Data for Figure 4 [file EMMM-15-e17907-s014.zip › SourceData_Fig_4/Fig_4_SourceData_images/3F/P16_AGED_12_VEH_GREEN.tif]

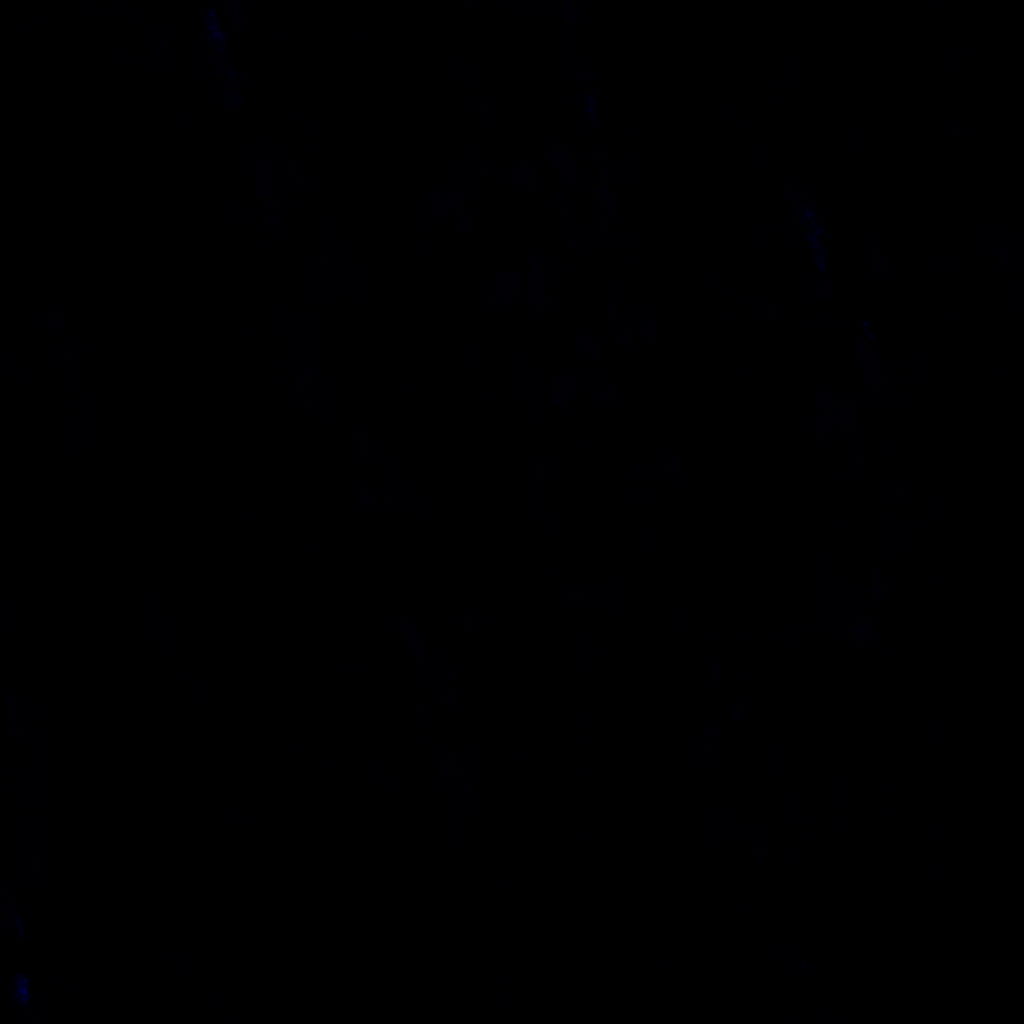

Supplement: Supplementary file 14 — Source Data for Figure 4 [file EMMM-15-e17907-s014.zip › SourceData_Fig_4/Fig_4_SourceData_images/3H/ABT_3_adult_42_dpi_no_reconex_cjun_19.lif_Series003/ABT_3_adult_42_dpi_no_reconex_cjun_19.lif_Series003_z00_ch00.tif]

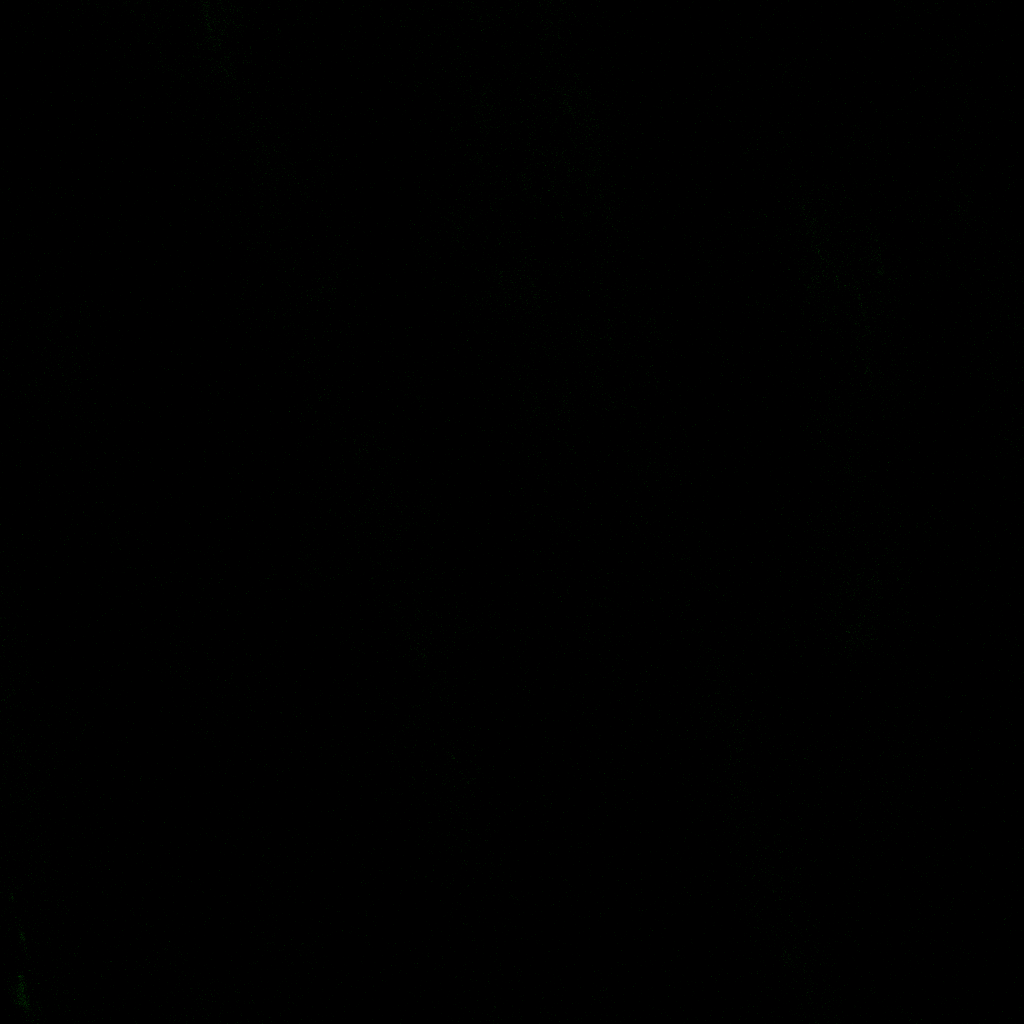

Supplement: Supplementary file 14 — Source Data for Figure 4 [file EMMM-15-e17907-s014.zip › SourceData_Fig_4/Fig_4_SourceData_images/3H/ABT_3_adult_42_dpi_no_reconex_cjun_19.lif_Series003/ABT_3_adult_42_dpi_no_reconex_cjun_19.lif_Series003_z00_ch01.tif]

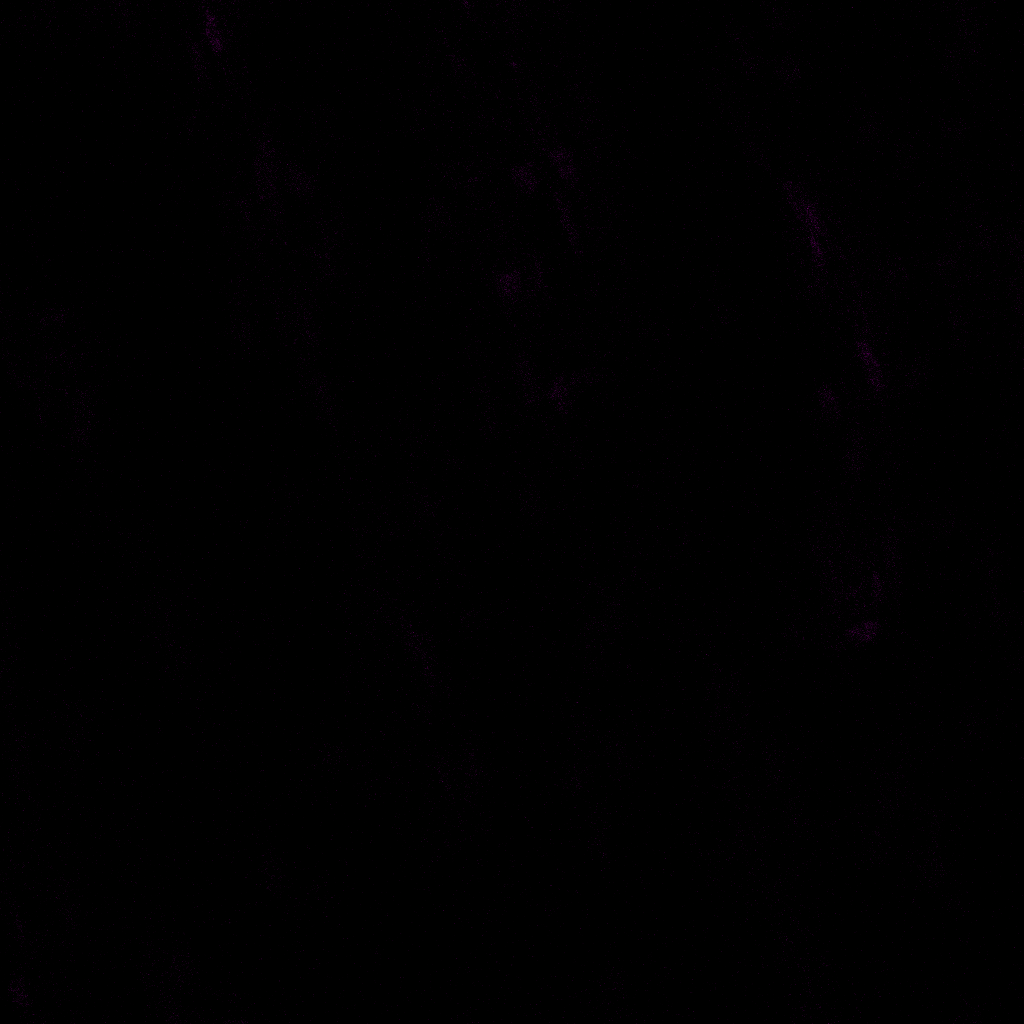

Supplement: Supplementary file 14 — Source Data for Figure 4 [file EMMM-15-e17907-s014.zip › SourceData_Fig_4/Fig_4_SourceData_images/3H/ABT_3_adult_42_dpi_no_reconex_cjun_19.lif_Series003/ABT_3_adult_42_dpi_no_reconex_cjun_19.lif_Series003_z00_ch02.tif]

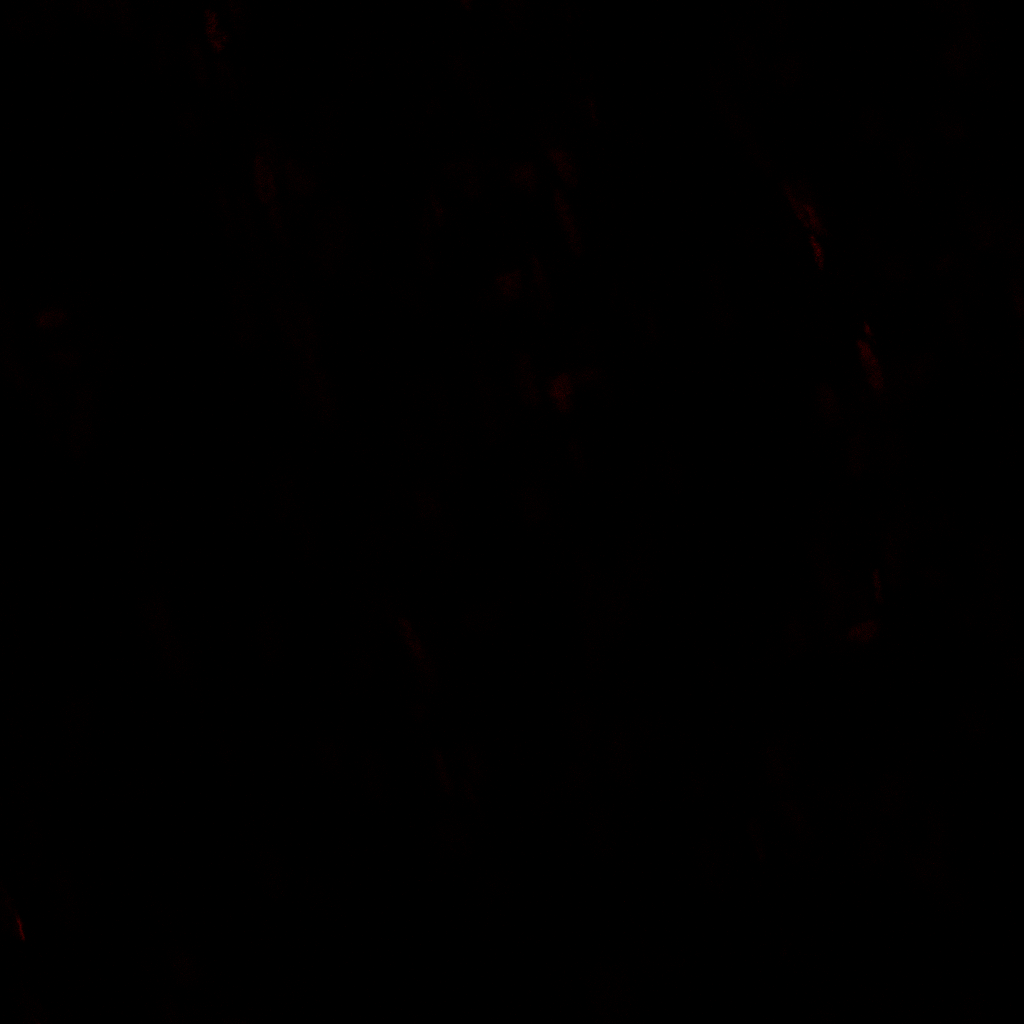

Supplement: Supplementary file 14 — Source Data for Figure 4 [file EMMM-15-e17907-s014.zip › SourceData_Fig_4/Fig_4_SourceData_images/3H/ABT_3_adult_42_dpi_no_reconex_cjun_19.lif_Series003/ABT_3_adult_42_dpi_no_reconex_cjun_19.lif_Series003_z00_ch03.tif]

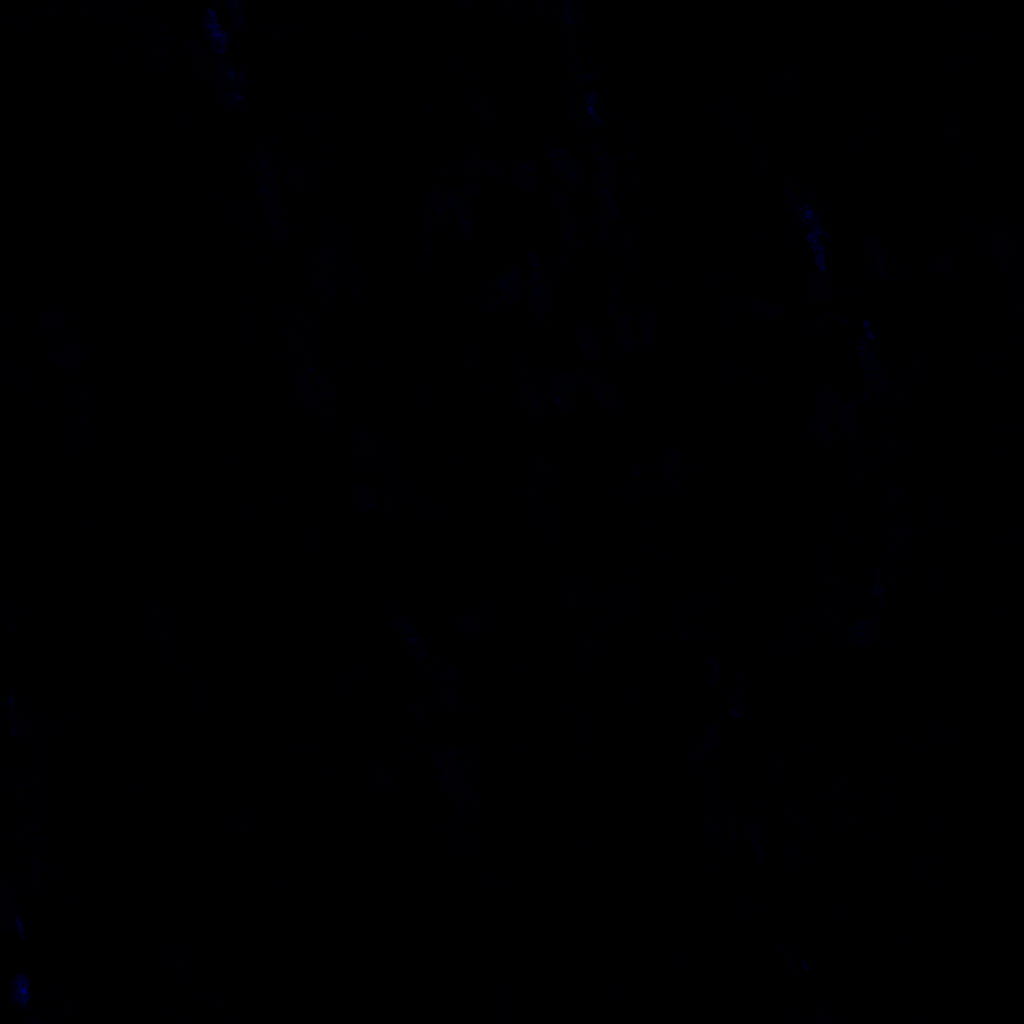

Supplement: Supplementary file 14 — Source Data for Figure 4 [file EMMM-15-e17907-s014.zip › SourceData_Fig_4/Fig_4_SourceData_images/3H/ABT_3_adult_42_dpi_no_reconex_cjun_19.lif_Series003/ABT_3_adult_42_dpi_no_reconex_cjun_19.lif_Series003_z01_ch00.tif]

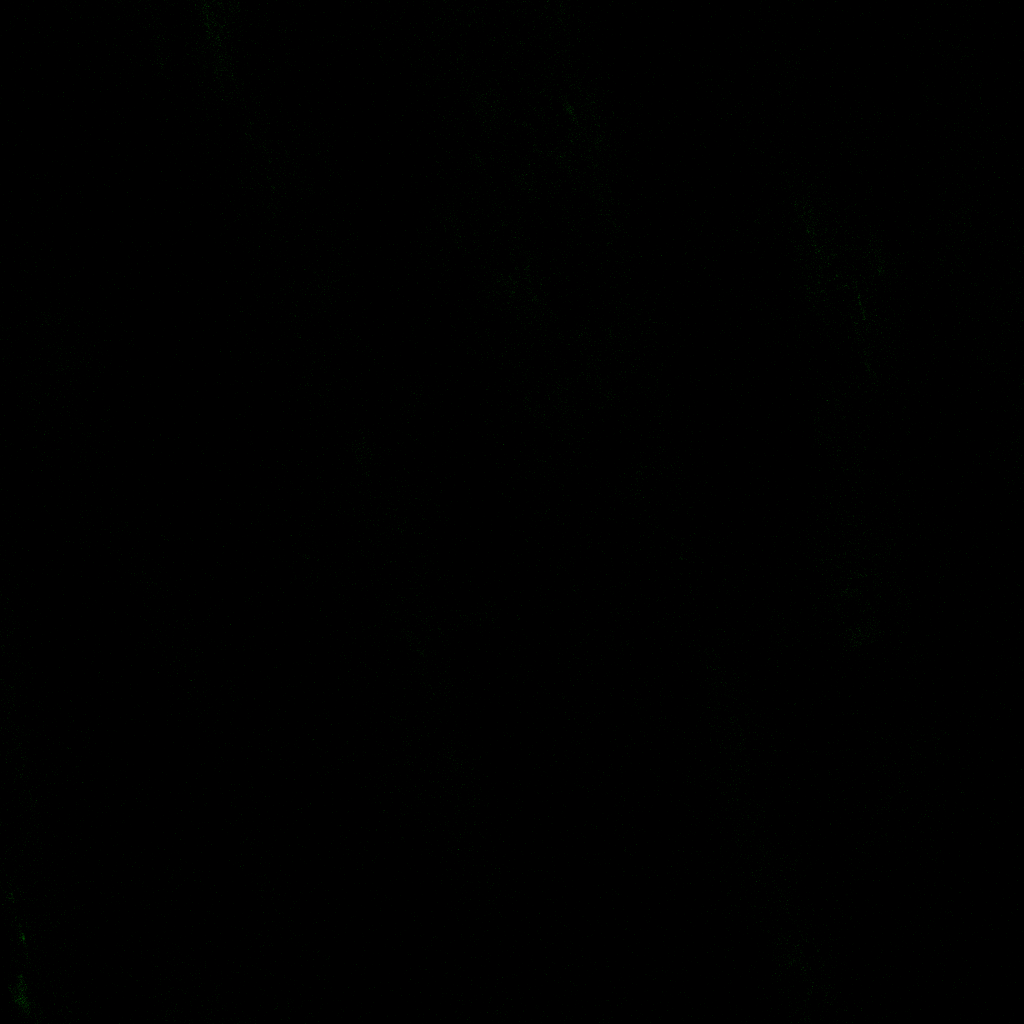

Supplement: Supplementary file 14 — Source Data for Figure 4 [file EMMM-15-e17907-s014.zip › SourceData_Fig_4/Fig_4_SourceData_images/3H/ABT_3_adult_42_dpi_no_reconex_cjun_19.lif_Series003/ABT_3_adult_42_dpi_no_reconex_cjun_19.lif_Series003_z01_ch01.tif]

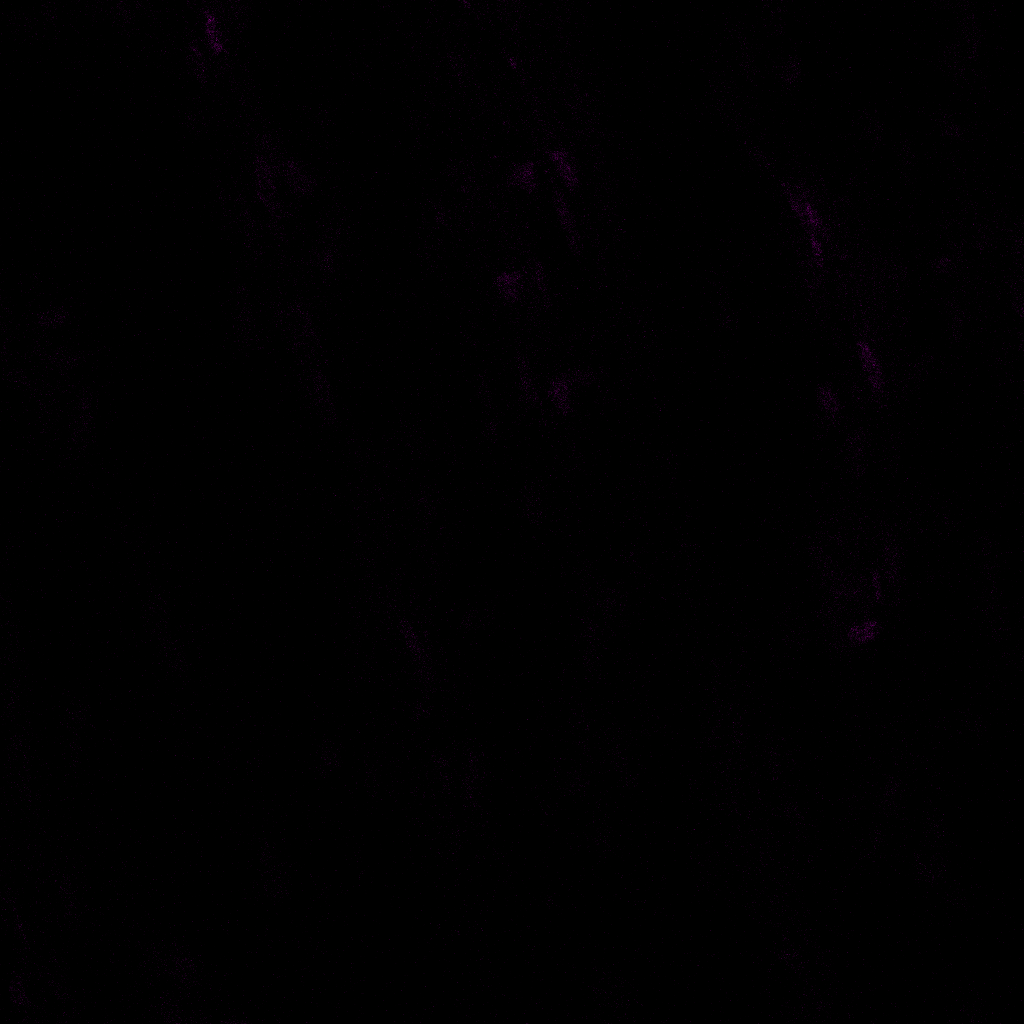

Supplement: Supplementary file 14 — Source Data for Figure 4 [file EMMM-15-e17907-s014.zip › SourceData_Fig_4/Fig_4_SourceData_images/3H/ABT_3_adult_42_dpi_no_reconex_cjun_19.lif_Series003/ABT_3_adult_42_dpi_no_reconex_cjun_19.lif_Series003_z01_ch02.tif]

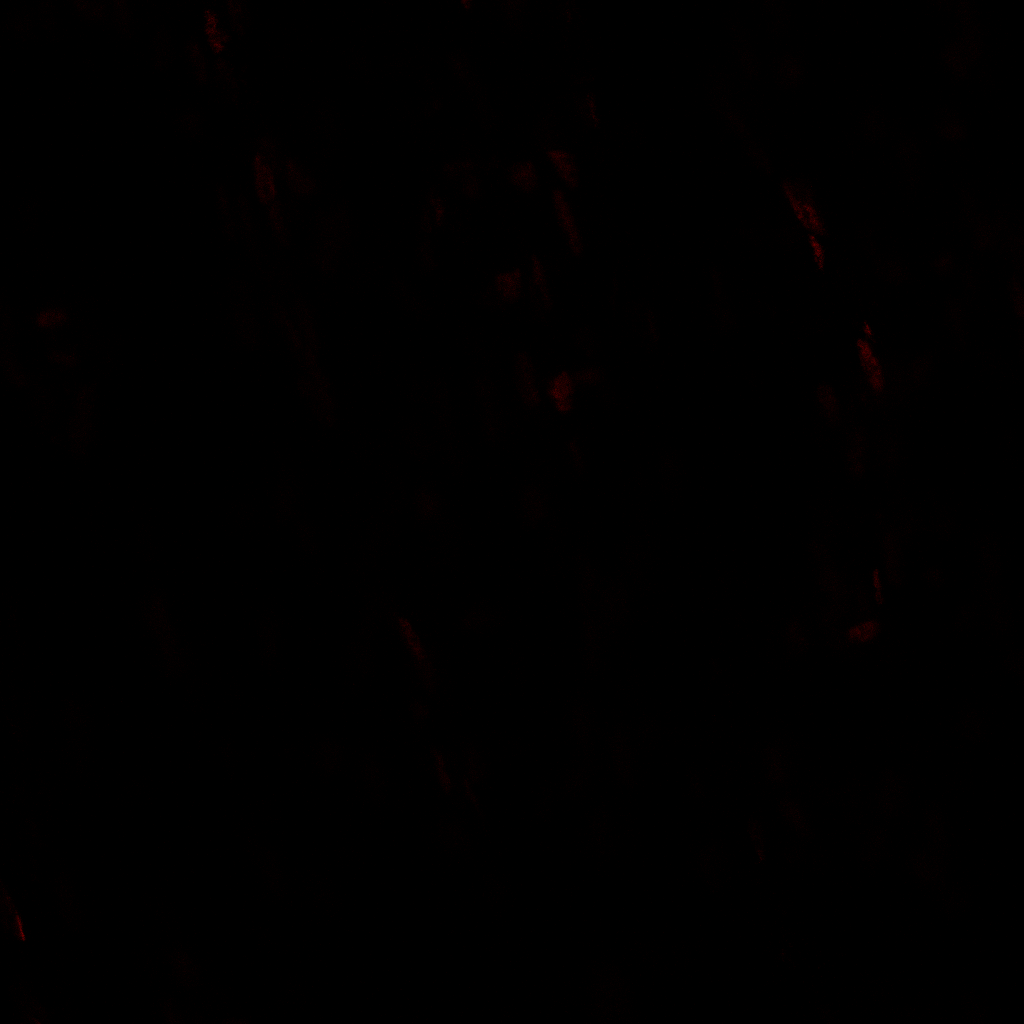

Supplement: Supplementary file 14 — Source Data for Figure 4 [file EMMM-15-e17907-s014.zip › SourceData_Fig_4/Fig_4_SourceData_images/3H/ABT_3_adult_42_dpi_no_reconex_cjun_19.lif_Series003/ABT_3_adult_42_dpi_no_reconex_cjun_19.lif_Series003_z01_ch03.tif]

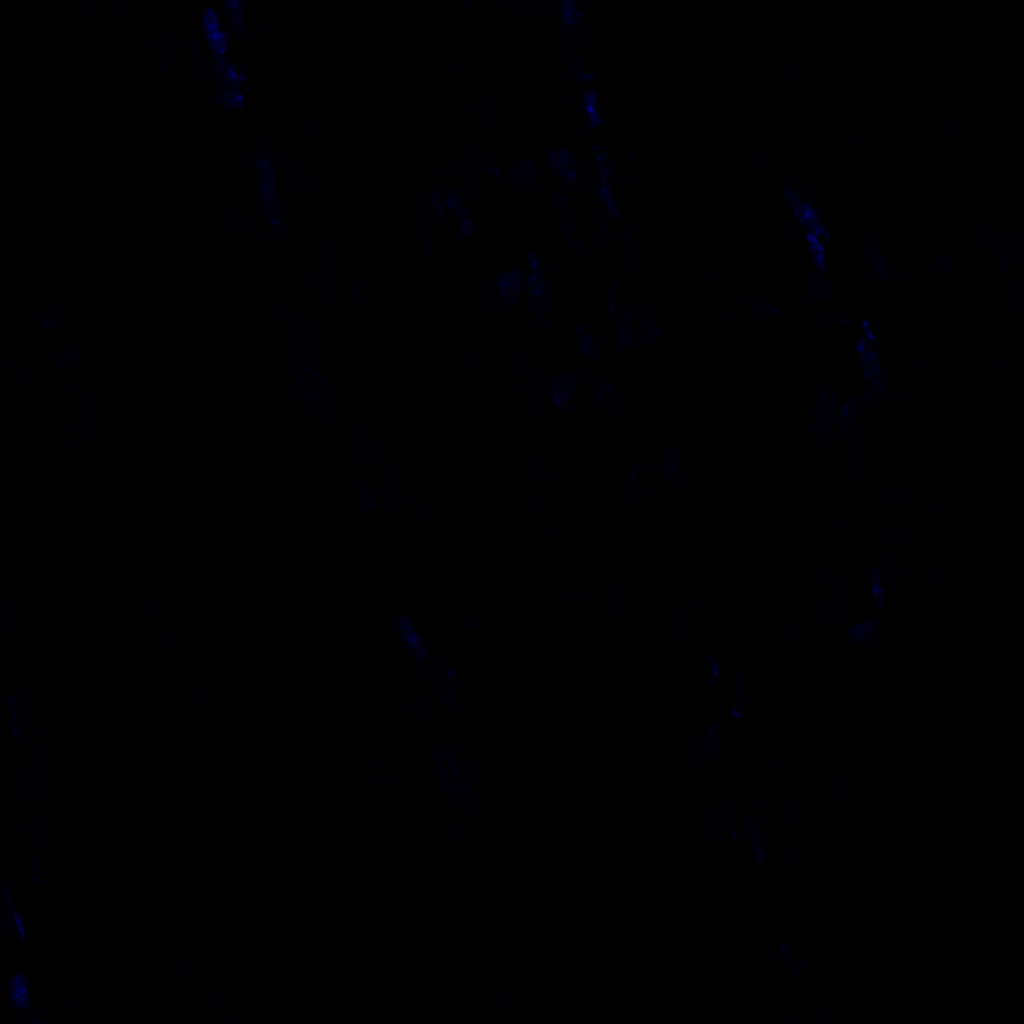

Supplement: Supplementary file 14 — Source Data for Figure 4 [file EMMM-15-e17907-s014.zip › SourceData_Fig_4/Fig_4_SourceData_images/3H/ABT_3_adult_42_dpi_no_reconex_cjun_19.lif_Series003/ABT_3_adult_42_dpi_no_reconex_cjun_19.lif_Series003_z02_ch00.tif]

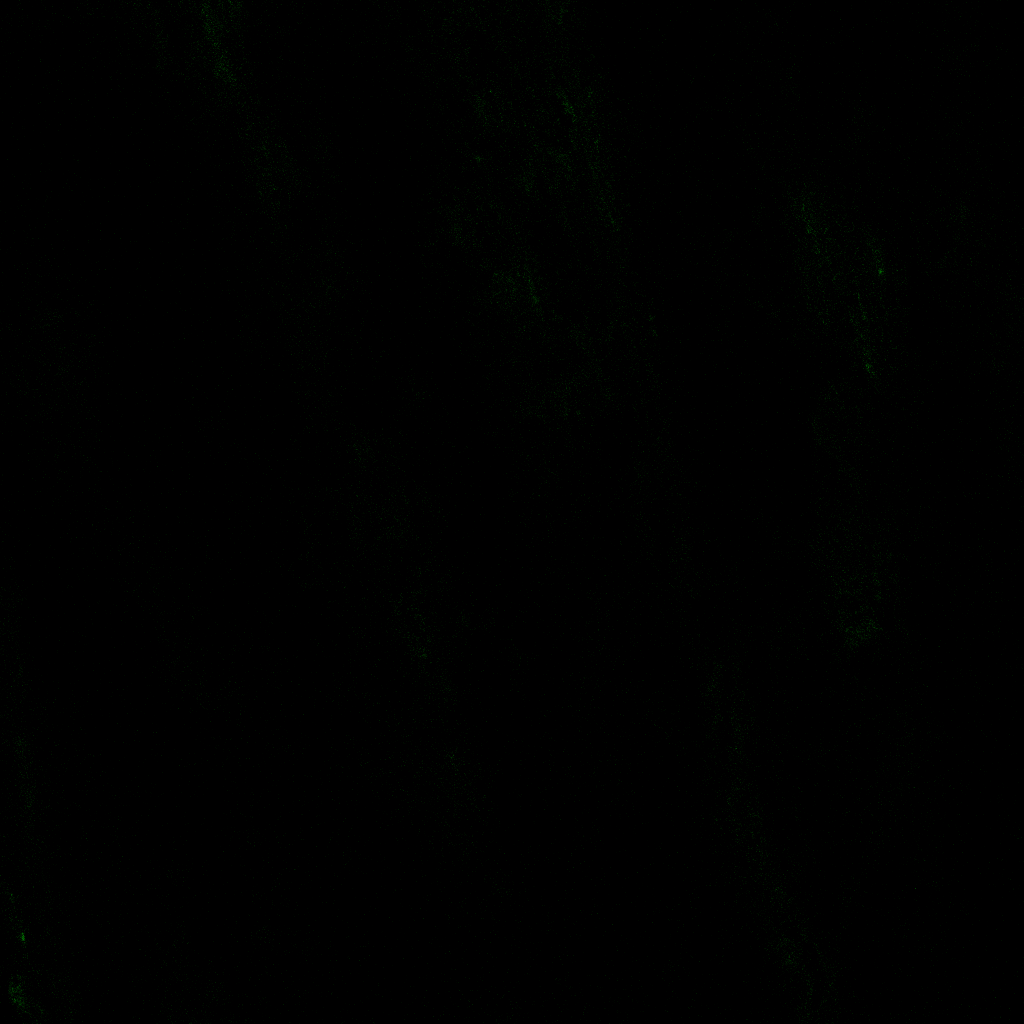

Supplement: Supplementary file 14 — Source Data for Figure 4 [file EMMM-15-e17907-s014.zip › SourceData_Fig_4/Fig_4_SourceData_images/3H/ABT_3_adult_42_dpi_no_reconex_cjun_19.lif_Series003/ABT_3_adult_42_dpi_no_reconex_cjun_19.lif_Series003_z02_ch01.tif]

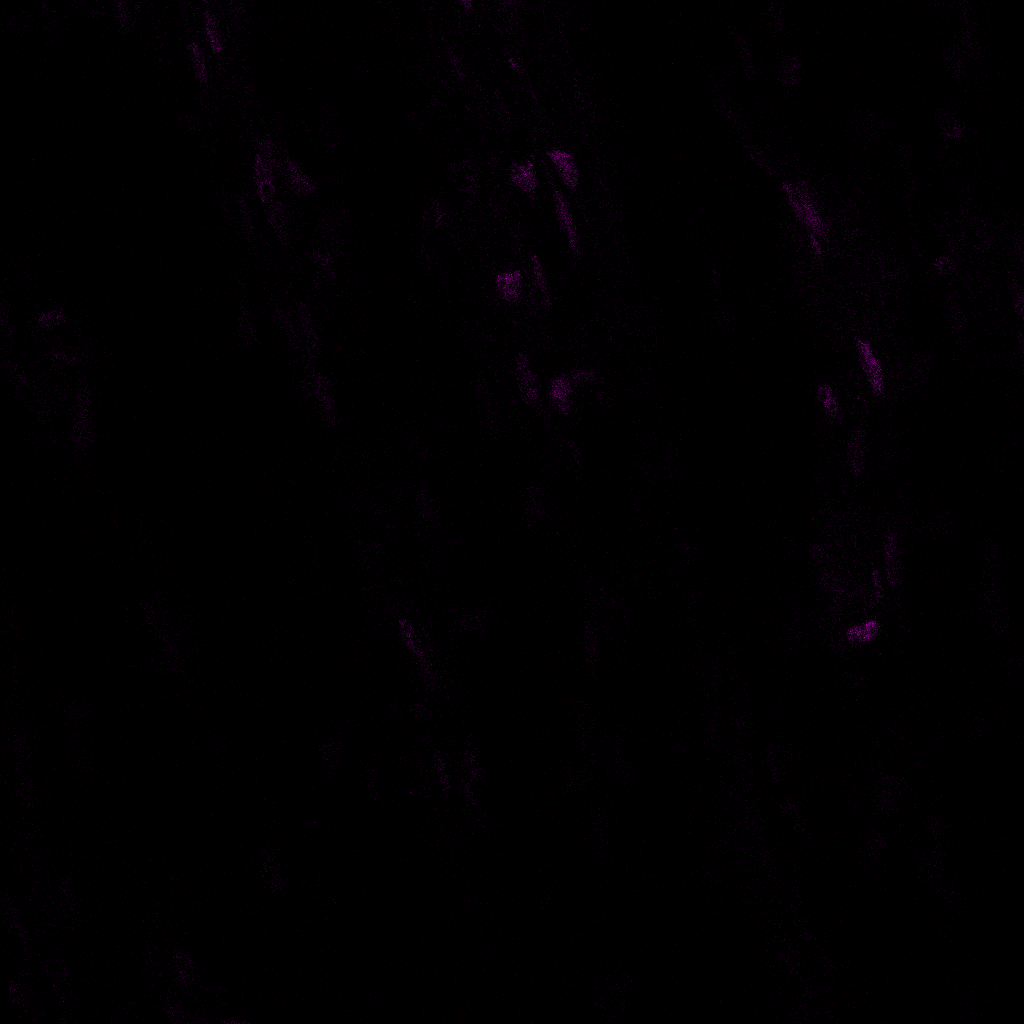

Supplement: Supplementary file 14 — Source Data for Figure 4 [file EMMM-15-e17907-s014.zip › SourceData_Fig_4/Fig_4_SourceData_images/3H/ABT_3_adult_42_dpi_no_reconex_cjun_19.lif_Series003/ABT_3_adult_42_dpi_no_reconex_cjun_19.lif_Series003_z02_ch02.tif]

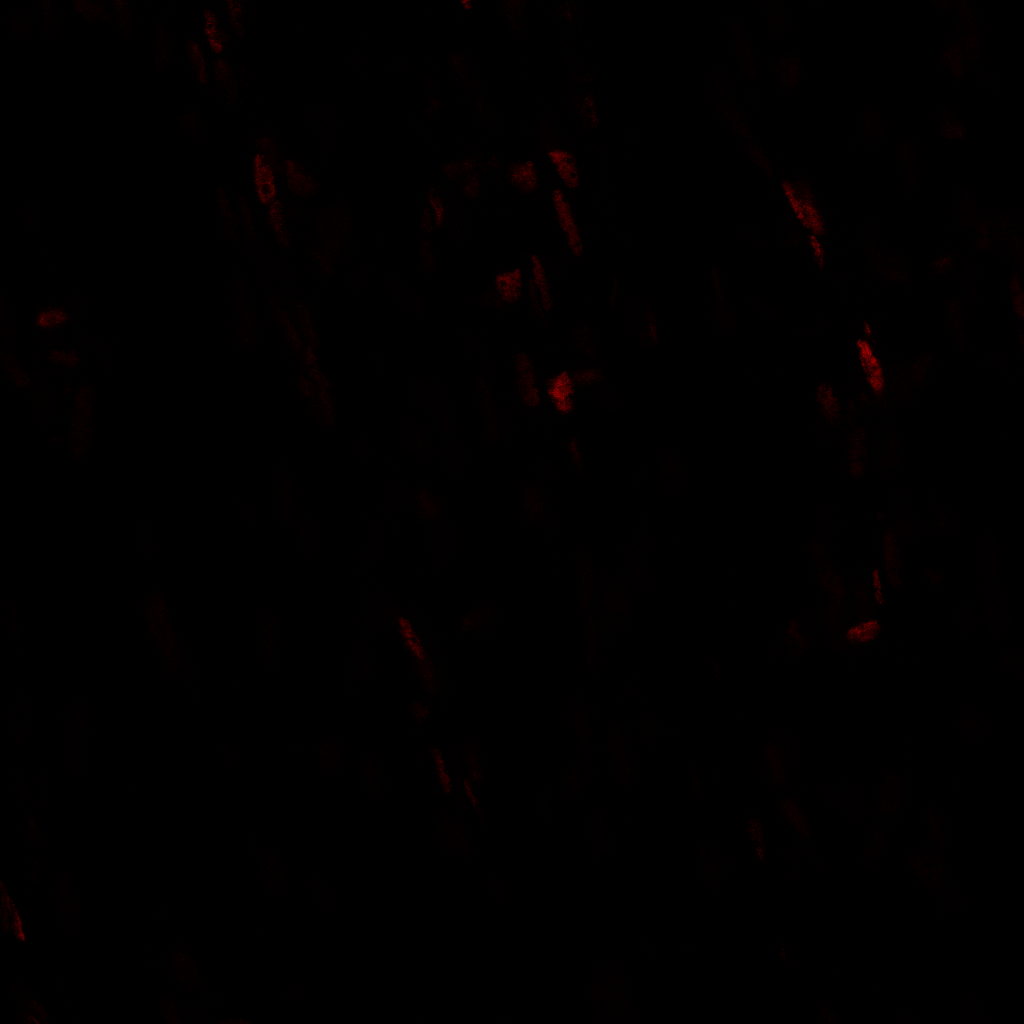

Supplement: Supplementary file 14 — Source Data for Figure 4 [file EMMM-15-e17907-s014.zip › SourceData_Fig_4/Fig_4_SourceData_images/3H/ABT_3_adult_42_dpi_no_reconex_cjun_19.lif_Series003/ABT_3_adult_42_dpi_no_reconex_cjun_19.lif_Series003_z02_ch03.tif]

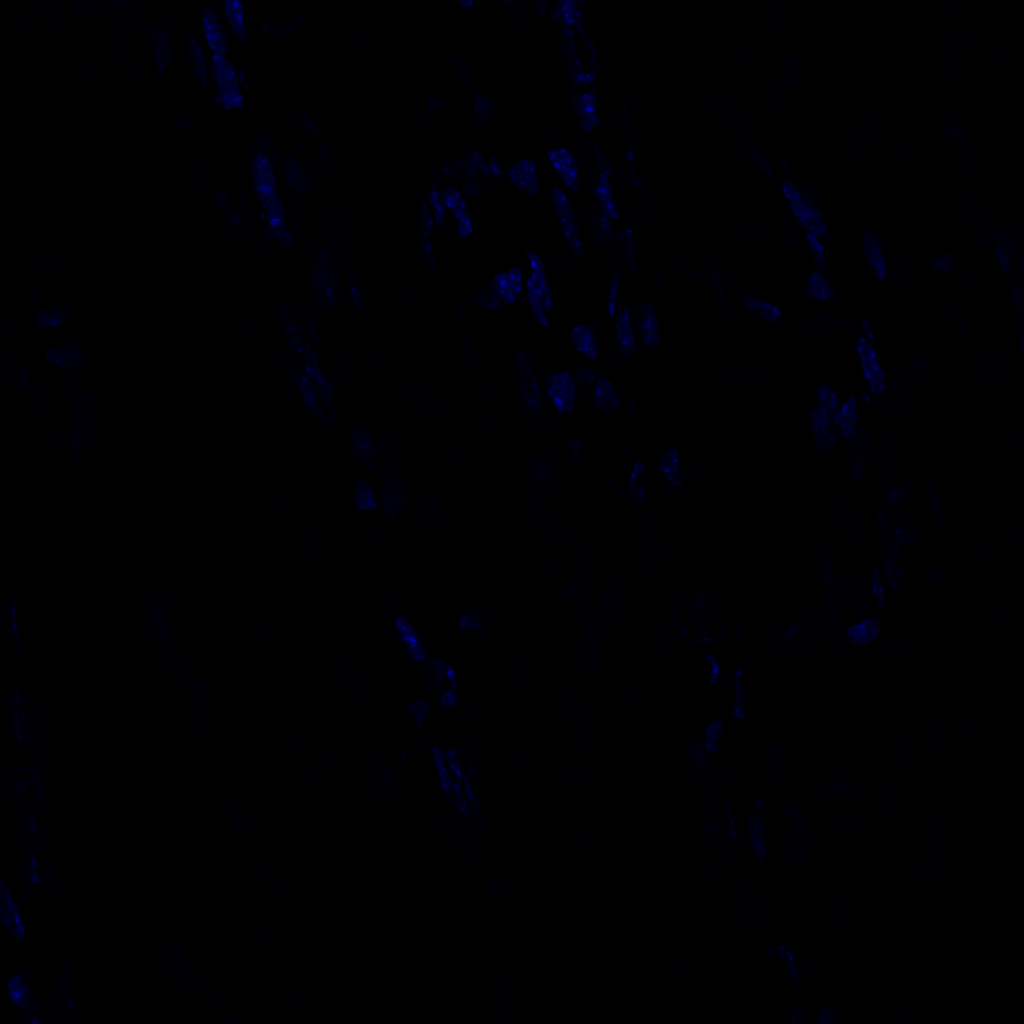

Supplement: Supplementary file 14 — Source Data for Figure 4 [file EMMM-15-e17907-s014.zip › SourceData_Fig_4/Fig_4_SourceData_images/3H/ABT_3_adult_42_dpi_no_reconex_cjun_19.lif_Series003/ABT_3_adult_42_dpi_no_reconex_cjun_19.lif_Series003_z03_ch00.tif]

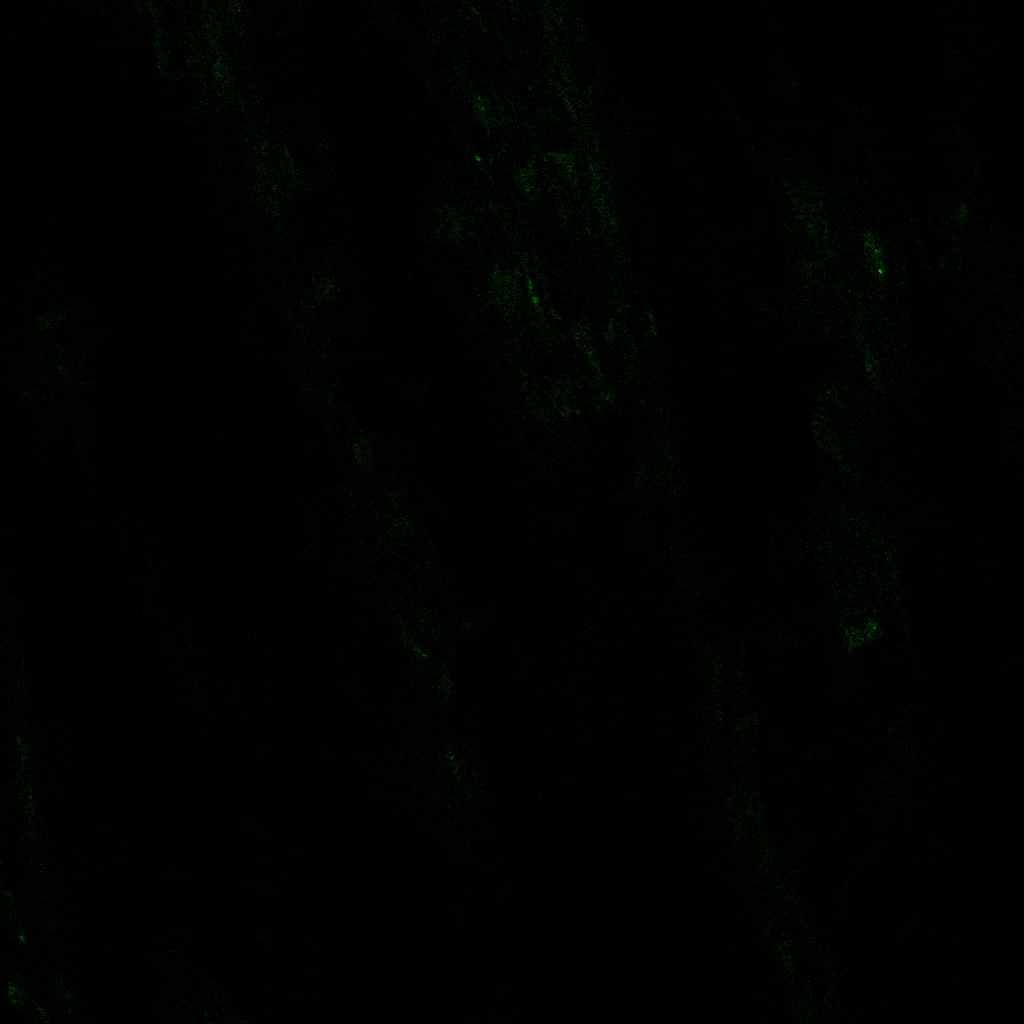

Supplement: Supplementary file 14 — Source Data for Figure 4 [file EMMM-15-e17907-s014.zip › SourceData_Fig_4/Fig_4_SourceData_images/3H/ABT_3_adult_42_dpi_no_reconex_cjun_19.lif_Series003/ABT_3_adult_42_dpi_no_reconex_cjun_19.lif_Series003_z03_ch01.tif]

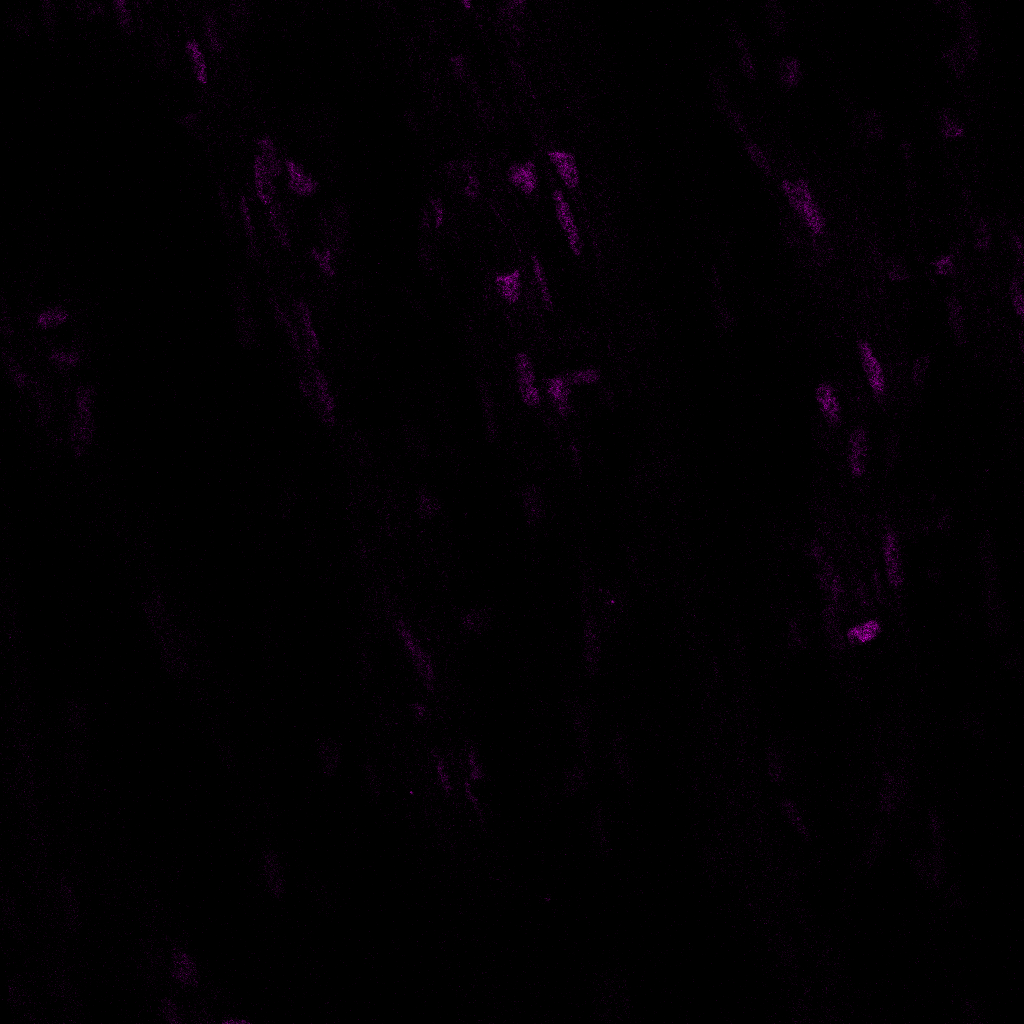

Supplement: Supplementary file 14 — Source Data for Figure 4 [file EMMM-15-e17907-s014.zip › SourceData_Fig_4/Fig_4_SourceData_images/3H/ABT_3_adult_42_dpi_no_reconex_cjun_19.lif_Series003/ABT_3_adult_42_dpi_no_reconex_cjun_19.lif_Series003_z03_ch02.tif]

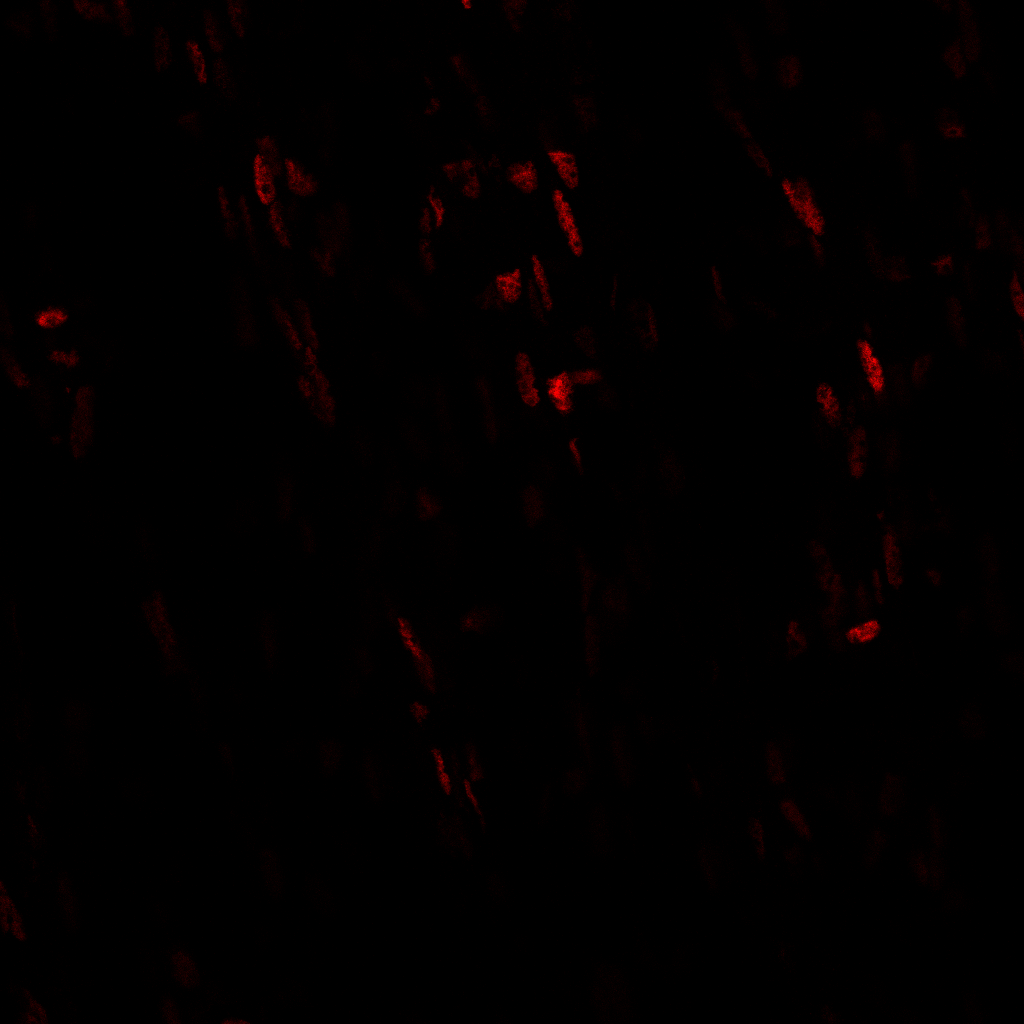

Supplement: Supplementary file 14 — Source Data for Figure 4 [file EMMM-15-e17907-s014.zip › SourceData_Fig_4/Fig_4_SourceData_images/3H/ABT_3_adult_42_dpi_no_reconex_cjun_19.lif_Series003/ABT_3_adult_42_dpi_no_reconex_cjun_19.lif_Series003_z03_ch03.tif]

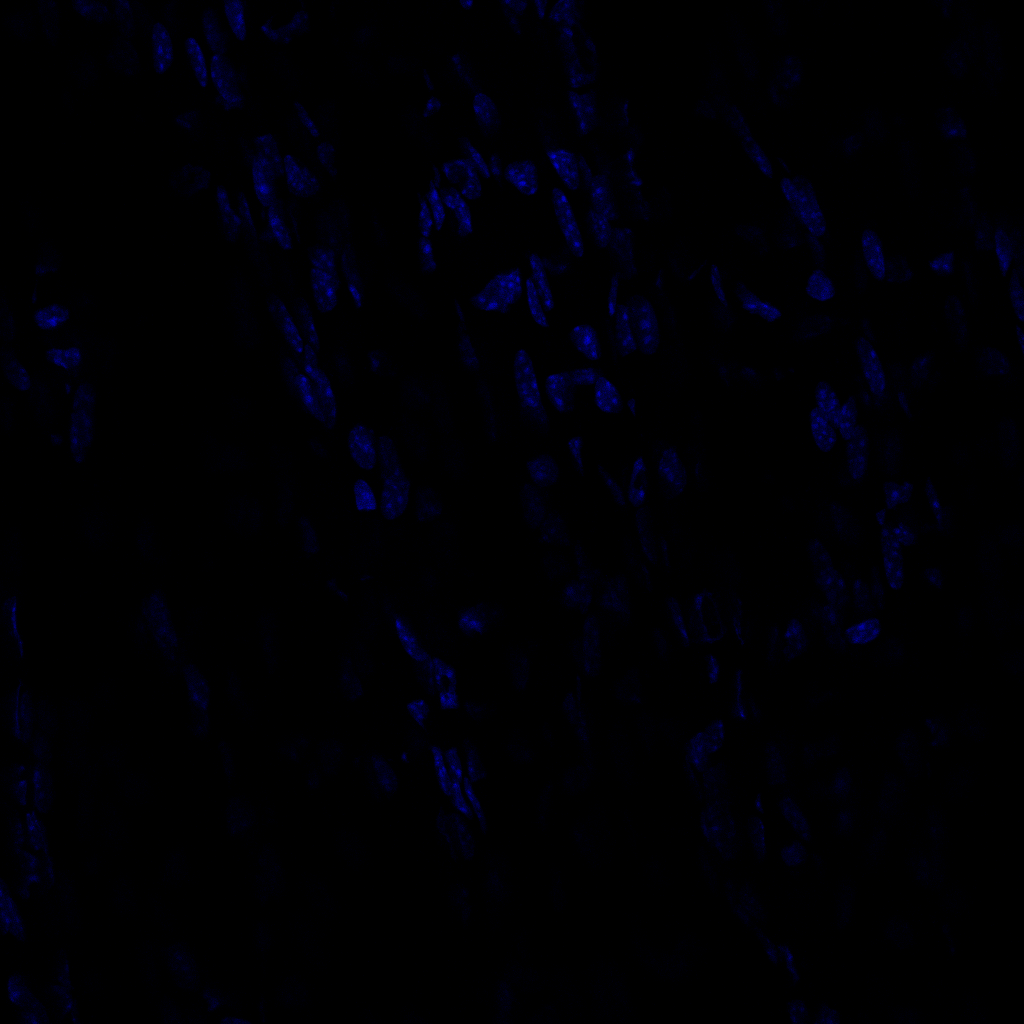

Supplement: Supplementary file 14 — Source Data for Figure 4 [file EMMM-15-e17907-s014.zip › SourceData_Fig_4/Fig_4_SourceData_images/3H/ABT_3_adult_42_dpi_no_reconex_cjun_19.lif_Series003/ABT_3_adult_42_dpi_no_reconex_cjun_19.lif_Series003_z04_ch00.tif]

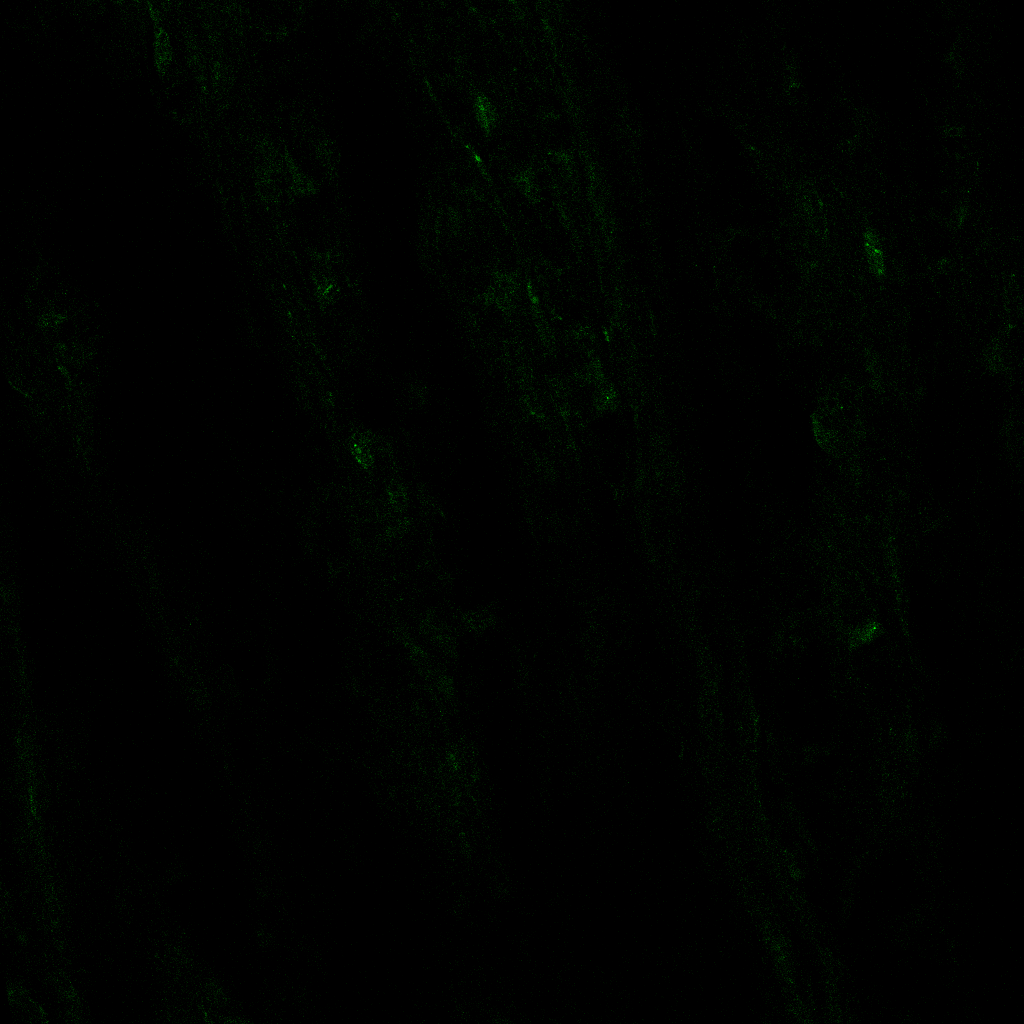

Supplement: Supplementary file 14 — Source Data for Figure 4 [file EMMM-15-e17907-s014.zip › SourceData_Fig_4/Fig_4_SourceData_images/3H/ABT_3_adult_42_dpi_no_reconex_cjun_19.lif_Series003/ABT_3_adult_42_dpi_no_reconex_cjun_19.lif_Series003_z04_ch01.tif]

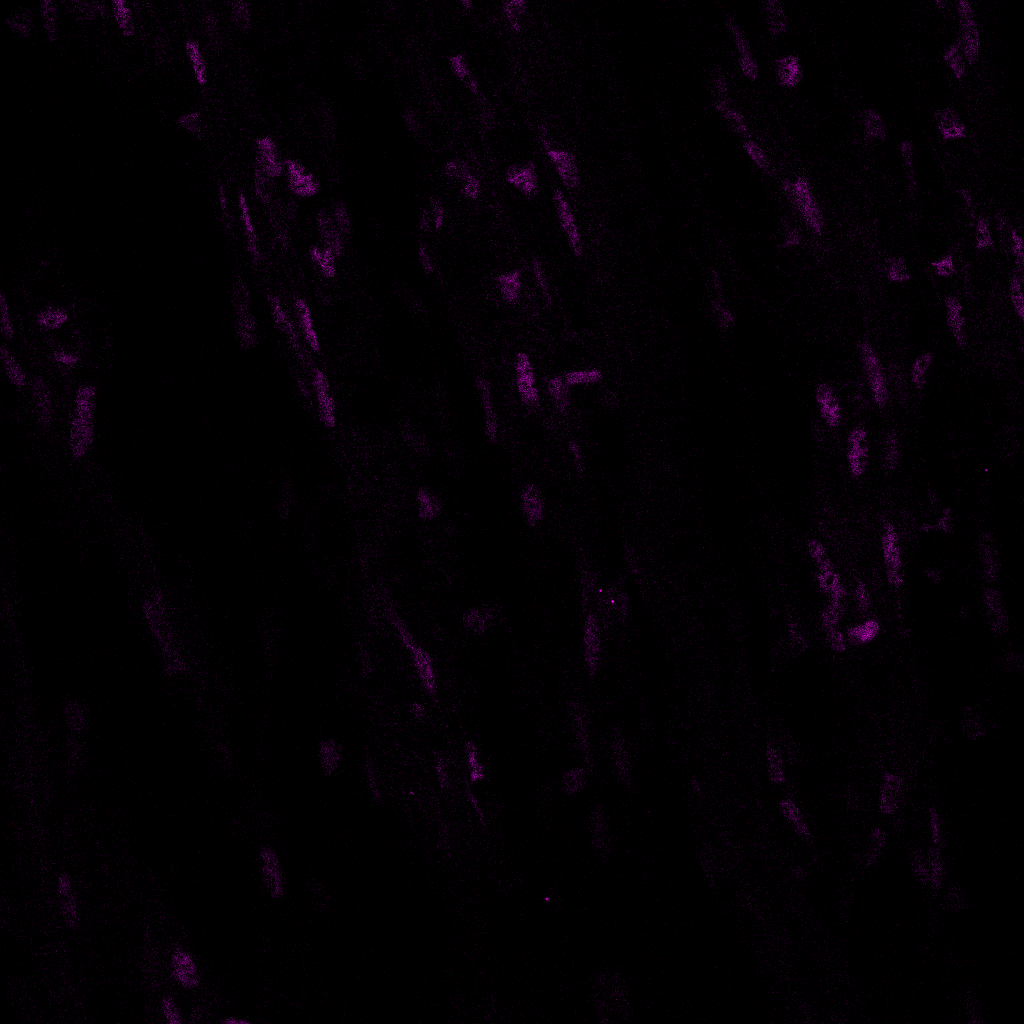

Supplement: Supplementary file 14 — Source Data for Figure 4 [file EMMM-15-e17907-s014.zip › SourceData_Fig_4/Fig_4_SourceData_images/3H/ABT_3_adult_42_dpi_no_reconex_cjun_19.lif_Series003/ABT_3_adult_42_dpi_no_reconex_cjun_19.lif_Series003_z04_ch02.tif]

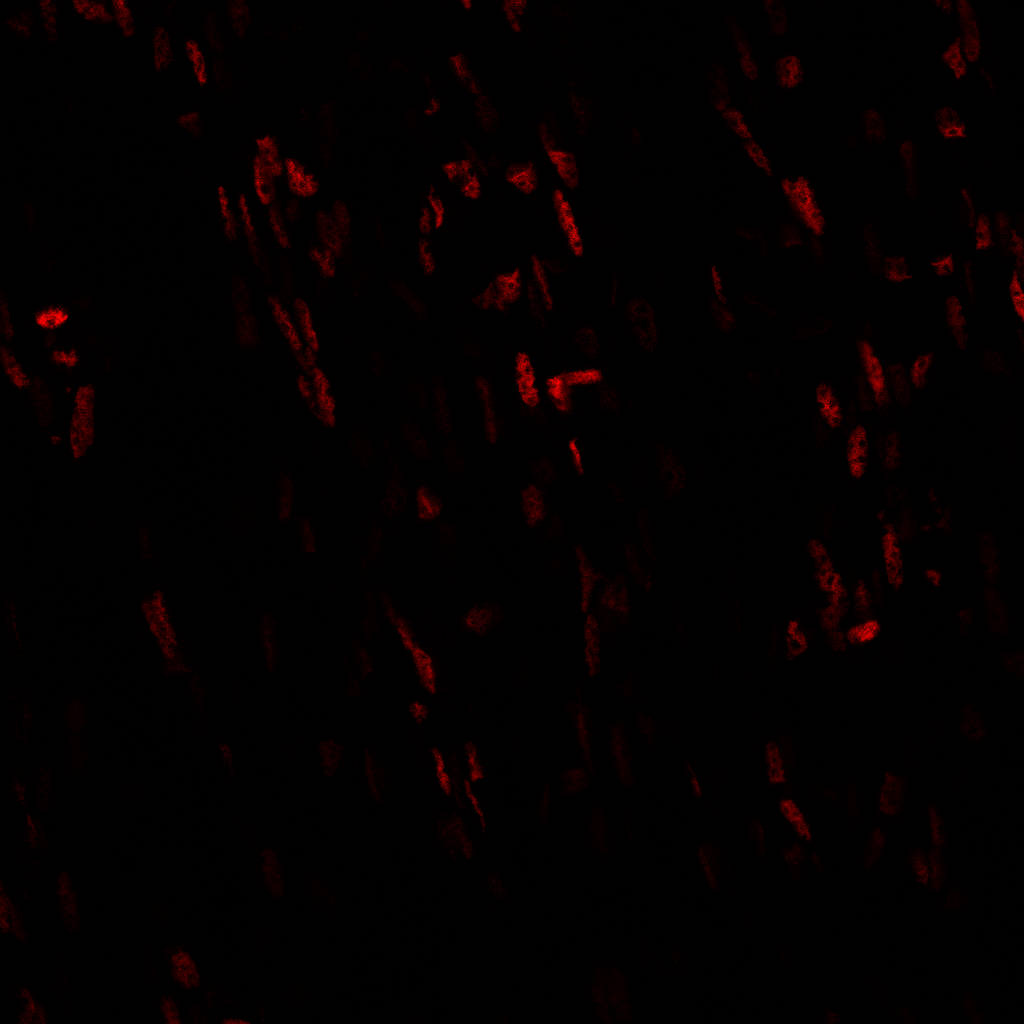

Supplement: Supplementary file 14 — Source Data for Figure 4 [file EMMM-15-e17907-s014.zip › SourceData_Fig_4/Fig_4_SourceData_images/3H/ABT_3_adult_42_dpi_no_reconex_cjun_19.lif_Series003/ABT_3_adult_42_dpi_no_reconex_cjun_19.lif_Series003_z04_ch03.tif]

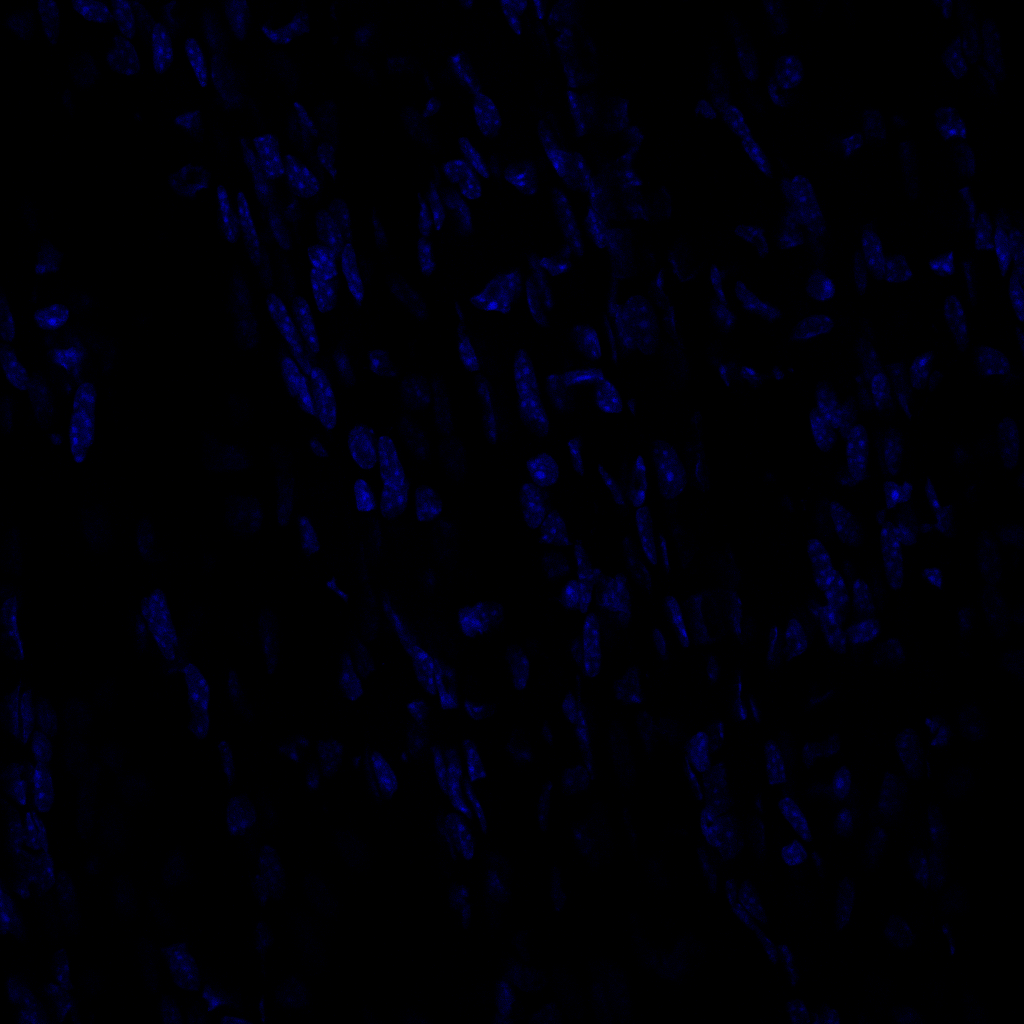

Supplement: Supplementary file 14 — Source Data for Figure 4 [file EMMM-15-e17907-s014.zip › SourceData_Fig_4/Fig_4_SourceData_images/3H/ABT_3_adult_42_dpi_no_reconex_cjun_19.lif_Series003/ABT_3_adult_42_dpi_no_reconex_cjun_19.lif_Series003_z05_ch00.tif]

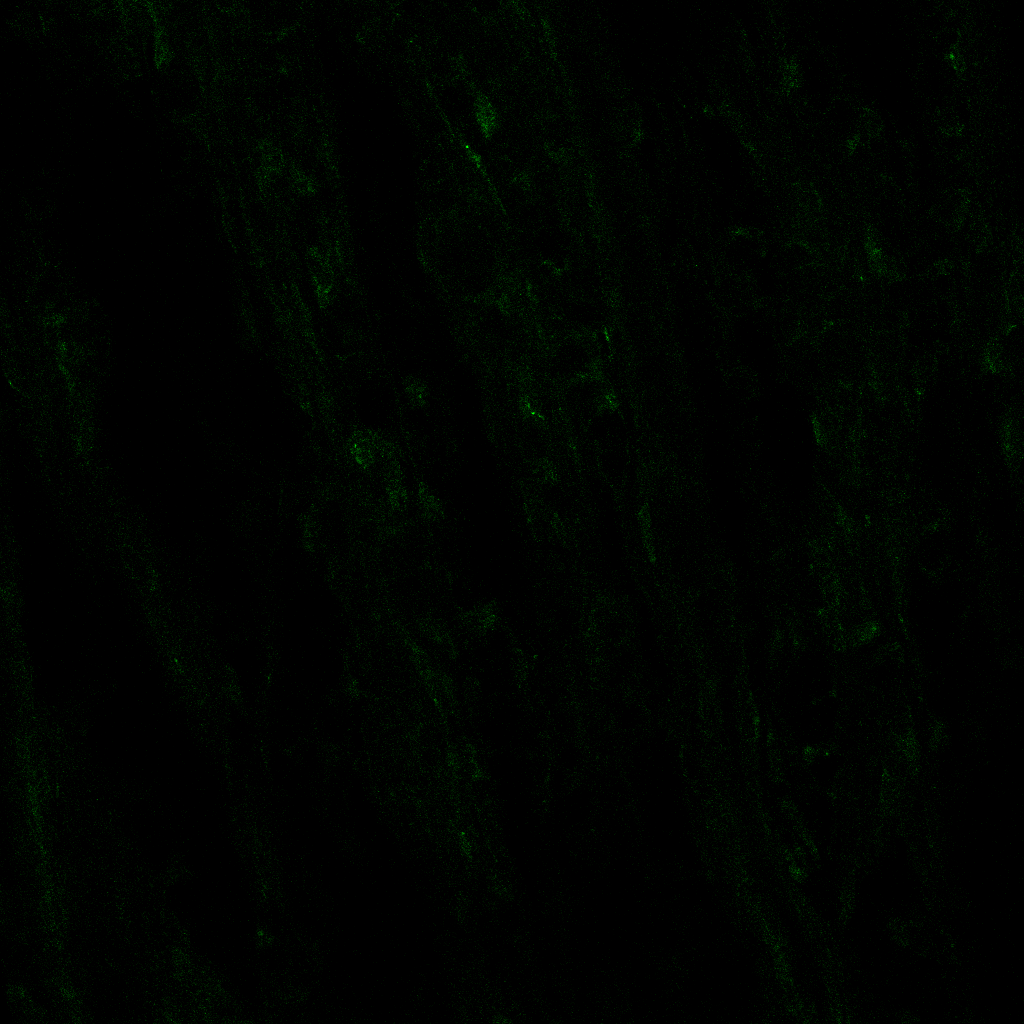

Supplement: Supplementary file 14 — Source Data for Figure 4 [file EMMM-15-e17907-s014.zip › SourceData_Fig_4/Fig_4_SourceData_images/3H/ABT_3_adult_42_dpi_no_reconex_cjun_19.lif_Series003/ABT_3_adult_42_dpi_no_reconex_cjun_19.lif_Series003_z05_ch01.tif]

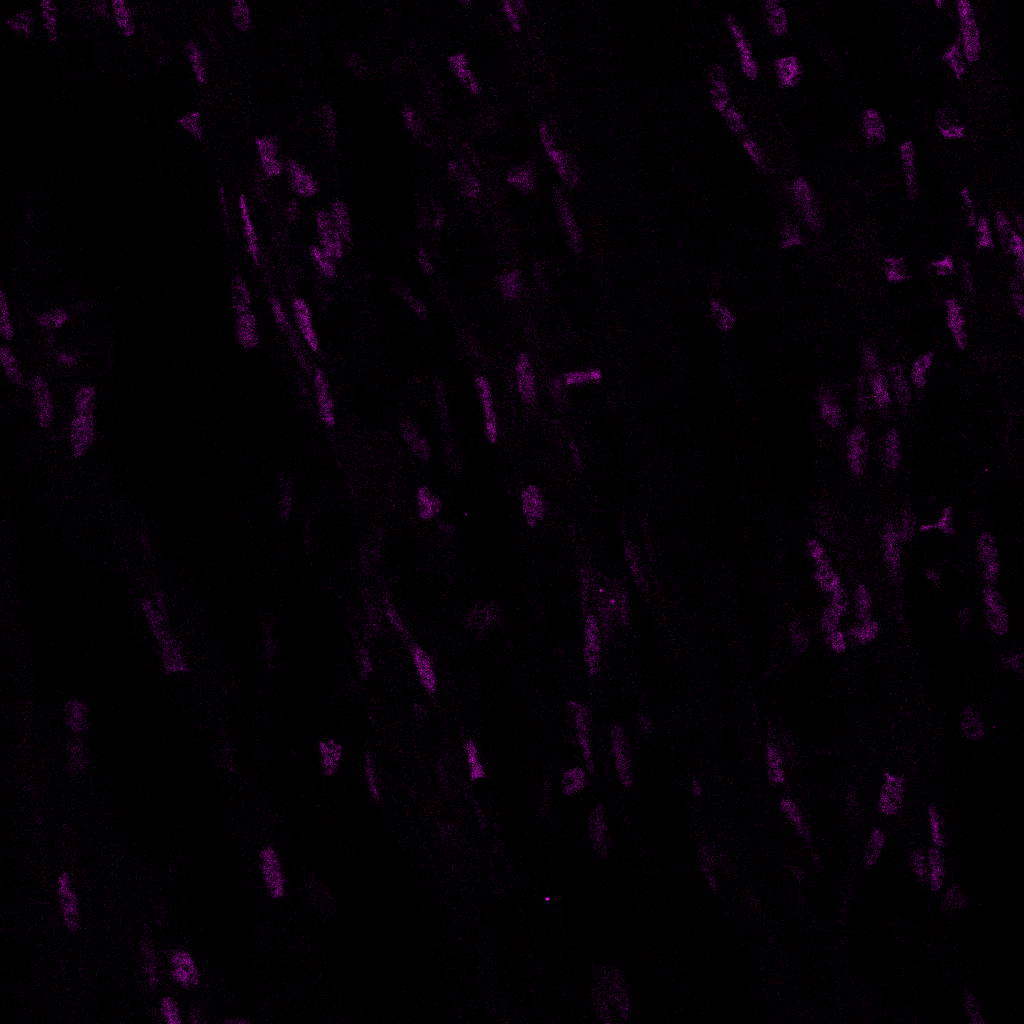

Supplement: Supplementary file 14 — Source Data for Figure 4 [file EMMM-15-e17907-s014.zip › SourceData_Fig_4/Fig_4_SourceData_images/3H/ABT_3_adult_42_dpi_no_reconex_cjun_19.lif_Series003/ABT_3_adult_42_dpi_no_reconex_cjun_19.lif_Series003_z05_ch02.tif]
