# Supplementary material for: Senescent Schwann cells induced by aging and chronic denervation impair axonal regeneration following peripheral nerve injury
Source: EMBO Mol Med. 2023 Oct 20;15(12):e17907. doi: 10.15252/emmm.202317907 (PMC10701627; doi:10.15252/emmm.202317907)

Figure 4K

con: contralateral (no damaged nerve)
Acu-D: acute denervation
Chr-D: chronica denervation

Region included in the Figure

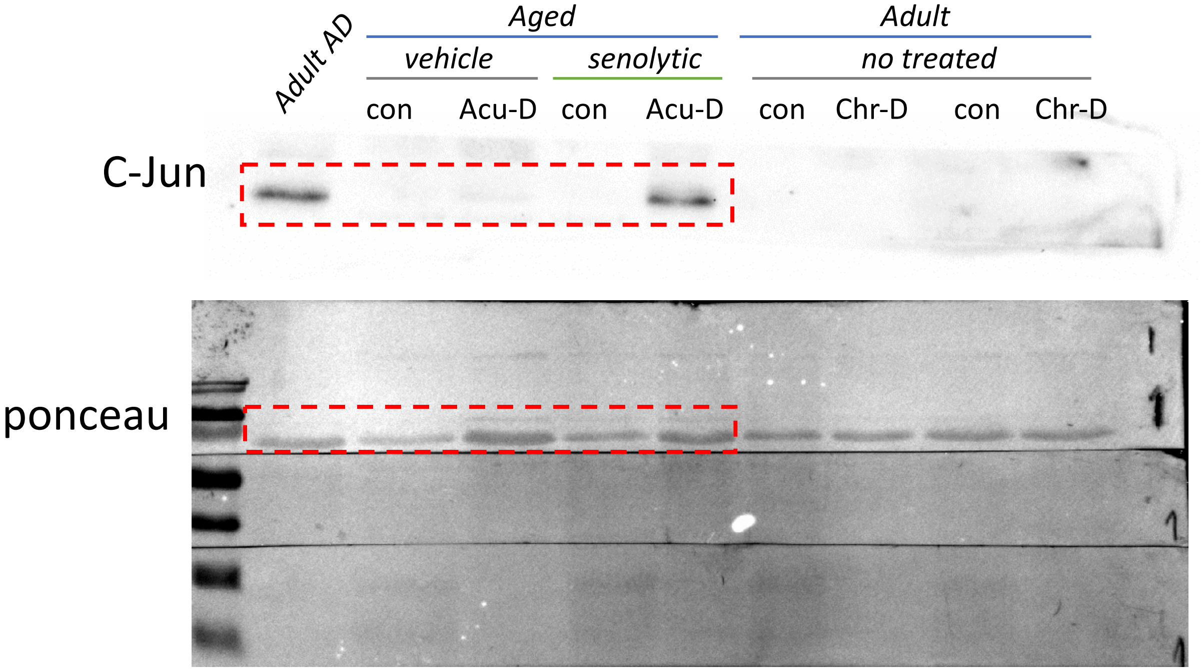

Supplement: Supplementary file 14 — Source Data for Figure 4 [file EMMM-15-e17907-s014.zip › SourceData_Fig_4/SourceData_Figure_4K_wb.pdf]
