# Supplementary figures and images for: Senescent Schwann cells induced by aging and chronic denervation impair axonal regeneration following peripheral nerve injury (part 3 of 3)
Source: EMBO Mol Med. 2023 Oct 20;15(12):e17907. doi: 10.15252/emmm.202317907 (PMC10701627; doi:10.15252/emmm.202317907)

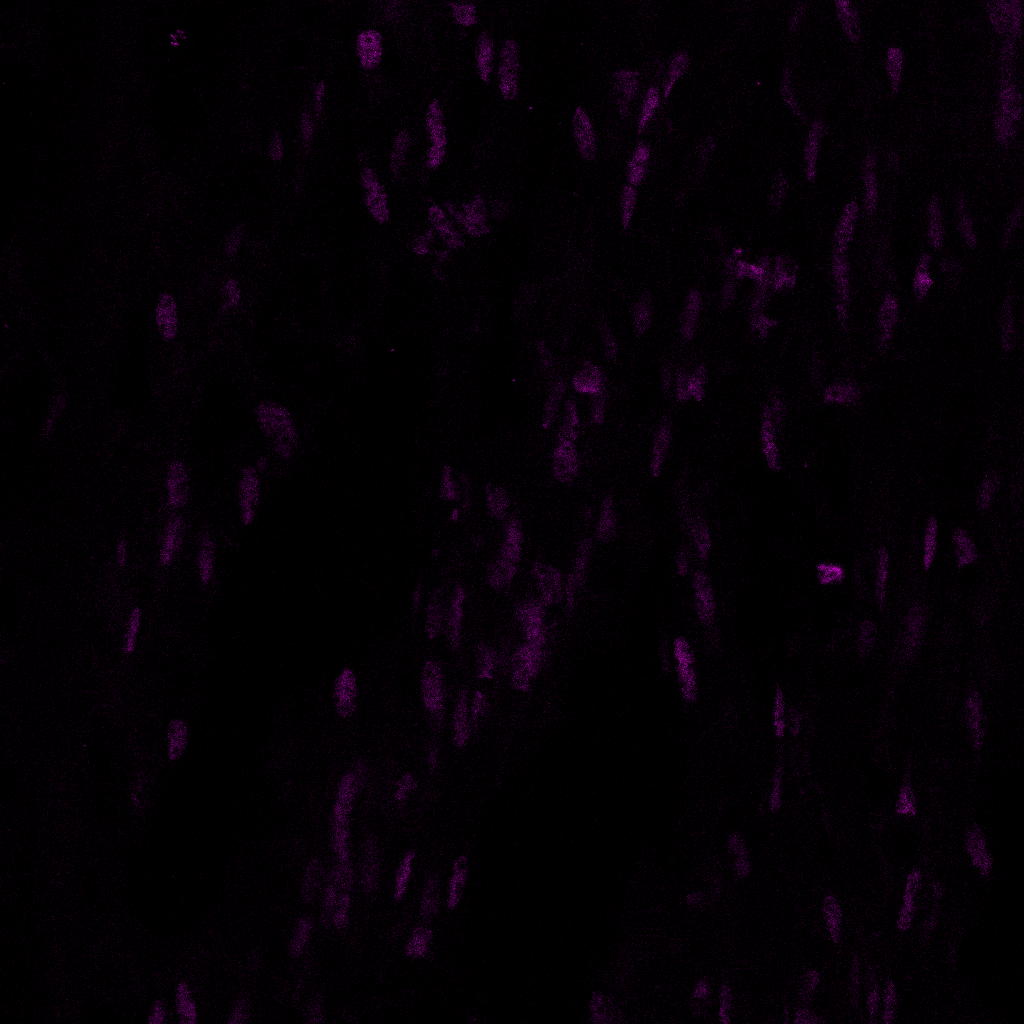

Supplement: Supplementary file 14 — Source Data for Figure 4 [file EMMM-15-e17907-s014.zip › SourceData_Fig_4/Fig_4_SourceData_images/3H/VEH_3_adult_42_dpi_no_reconex_cjun_19.lif_Series001/VEH_3_adult_42_dpi_no_reconex_cjun_19.lif_Series001_z06_ch02.tif]

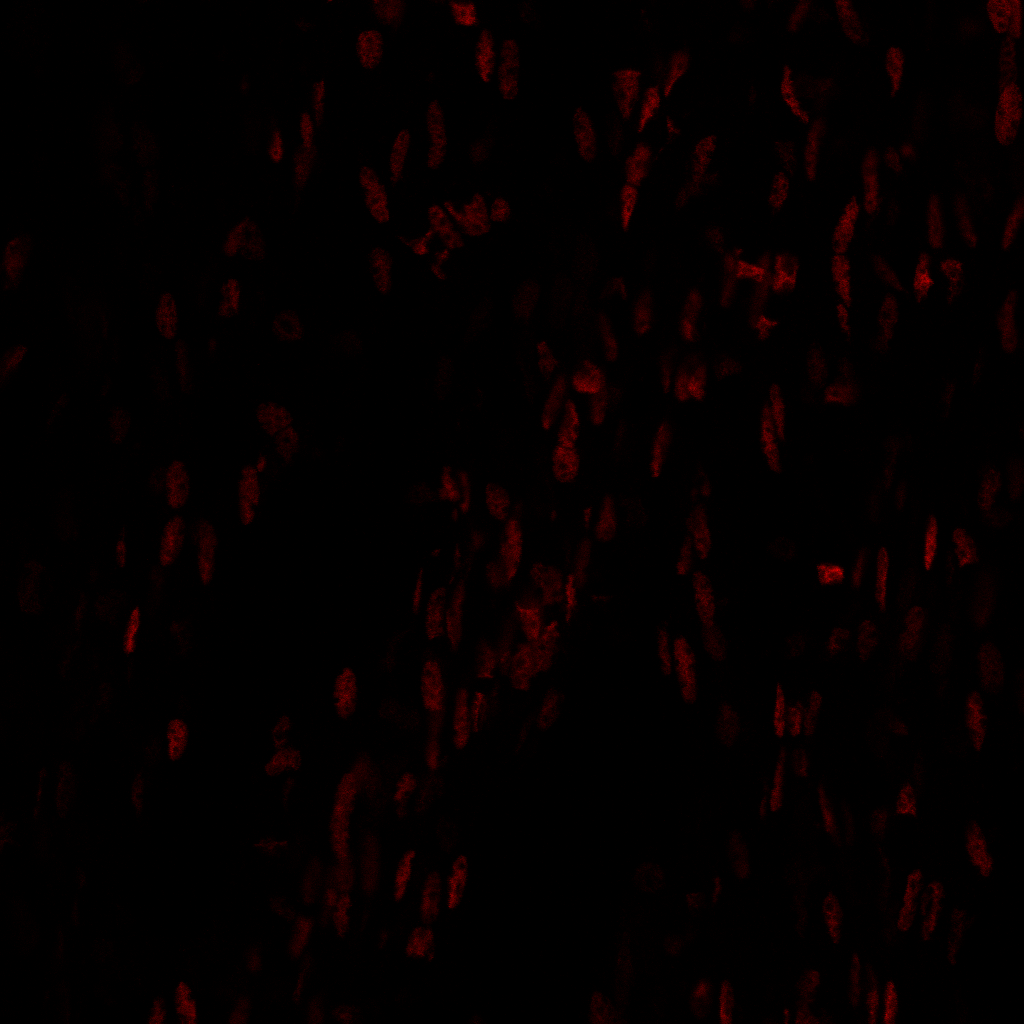

Supplement: Supplementary file 14 — Source Data for Figure 4 [file EMMM-15-e17907-s014.zip › SourceData_Fig_4/Fig_4_SourceData_images/3H/VEH_3_adult_42_dpi_no_reconex_cjun_19.lif_Series001/VEH_3_adult_42_dpi_no_reconex_cjun_19.lif_Series001_z06_ch03.tif]

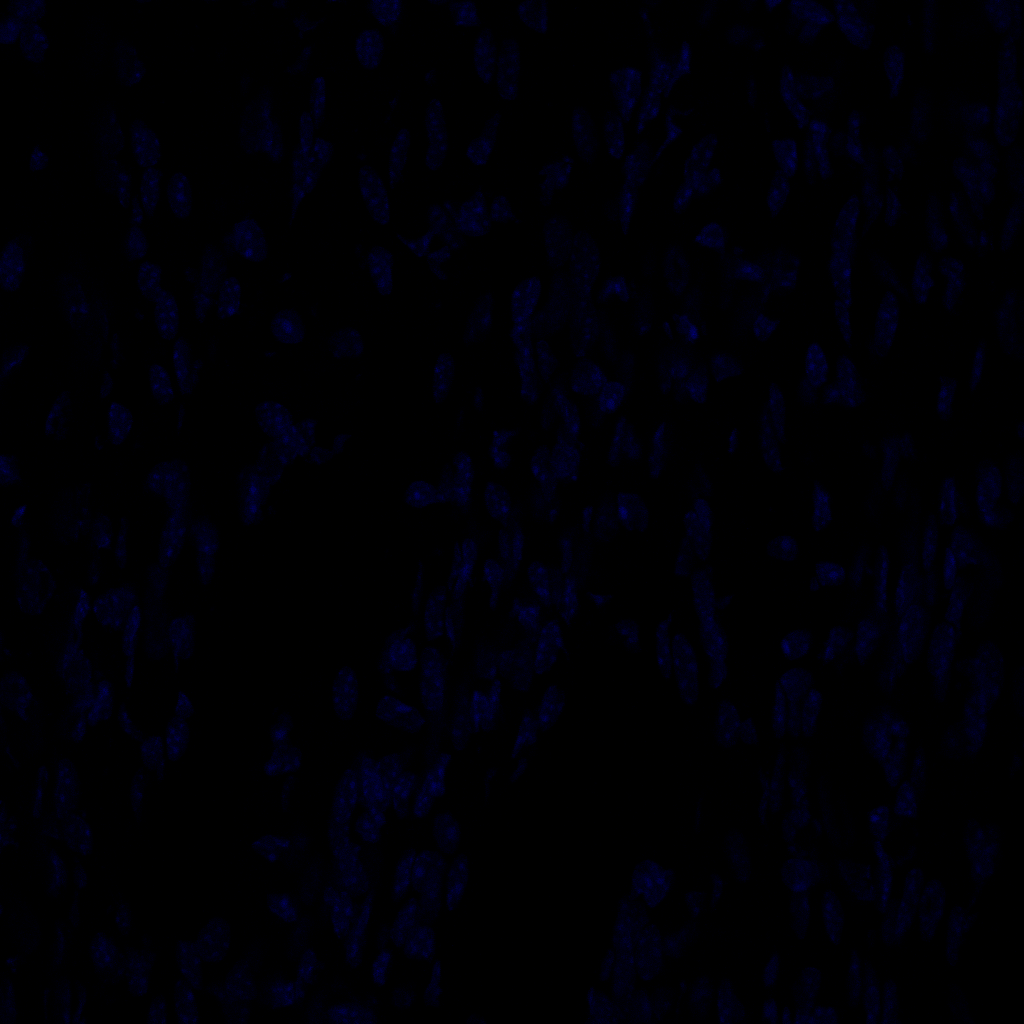

Supplement: Supplementary file 14 — Source Data for Figure 4 [file EMMM-15-e17907-s014.zip › SourceData_Fig_4/Fig_4_SourceData_images/3H/VEH_3_adult_42_dpi_no_reconex_cjun_19.lif_Series001/VEH_3_adult_42_dpi_no_reconex_cjun_19.lif_Series001_z07_ch00.tif]

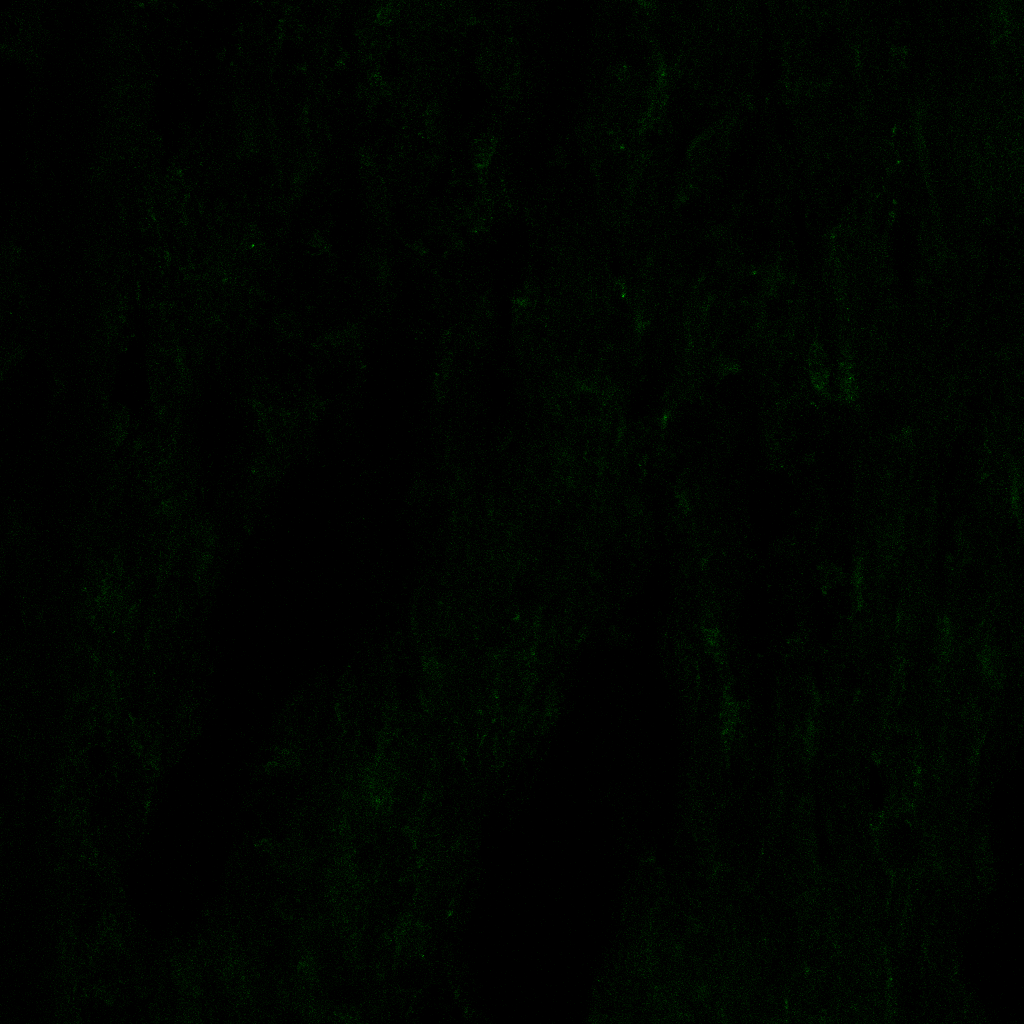

Supplement: Supplementary file 14 — Source Data for Figure 4 [file EMMM-15-e17907-s014.zip › SourceData_Fig_4/Fig_4_SourceData_images/3H/VEH_3_adult_42_dpi_no_reconex_cjun_19.lif_Series001/VEH_3_adult_42_dpi_no_reconex_cjun_19.lif_Series001_z07_ch01.tif]

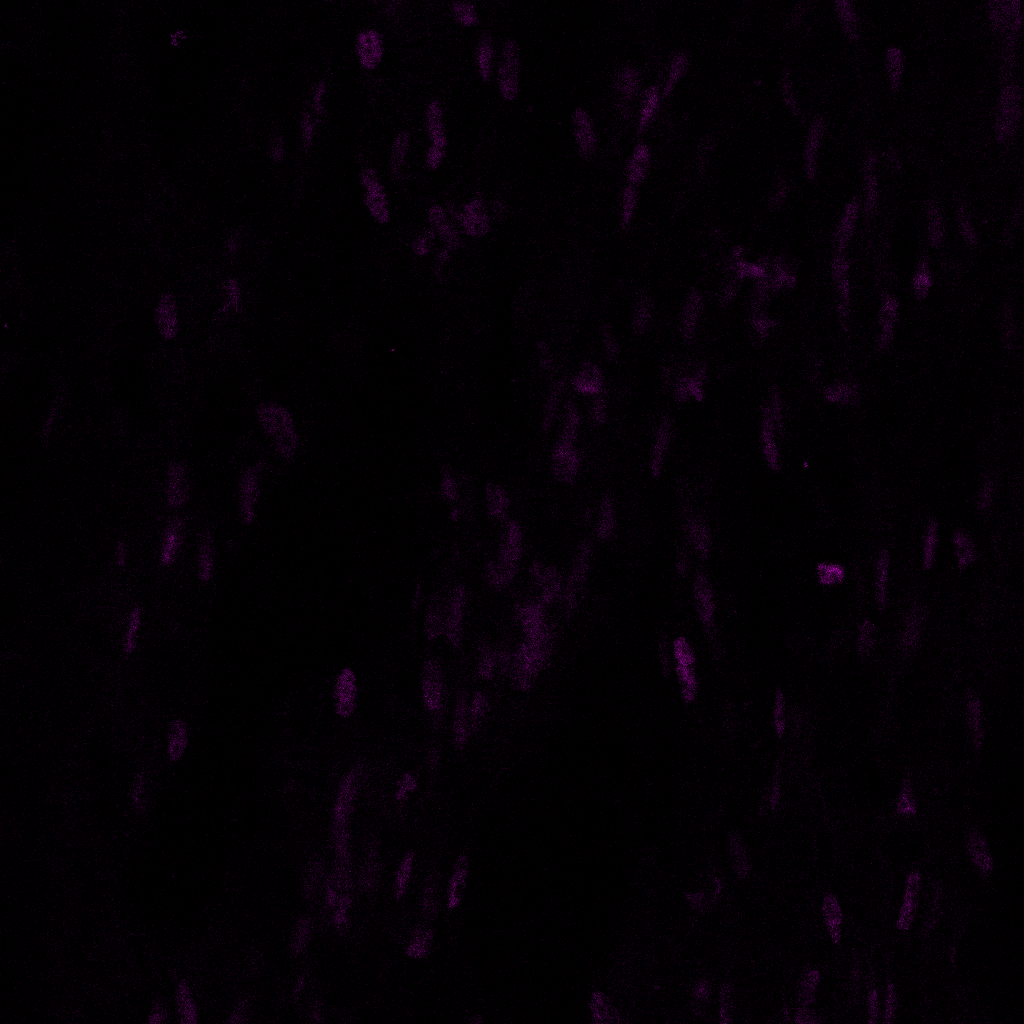

Supplement: Supplementary file 14 — Source Data for Figure 4 [file EMMM-15-e17907-s014.zip › SourceData_Fig_4/Fig_4_SourceData_images/3H/VEH_3_adult_42_dpi_no_reconex_cjun_19.lif_Series001/VEH_3_adult_42_dpi_no_reconex_cjun_19.lif_Series001_z07_ch02.tif]

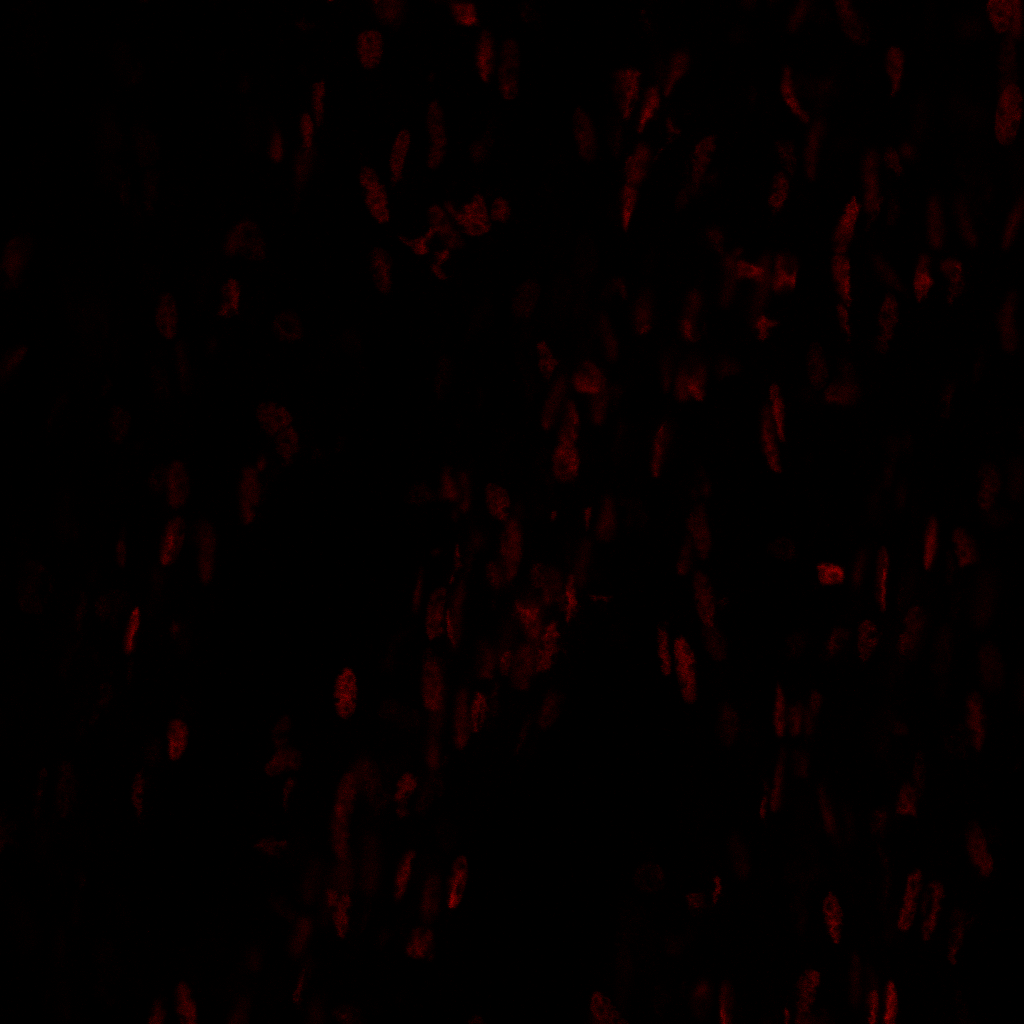

Supplement: Supplementary file 14 — Source Data for Figure 4 [file EMMM-15-e17907-s014.zip › SourceData_Fig_4/Fig_4_SourceData_images/3H/VEH_3_adult_42_dpi_no_reconex_cjun_19.lif_Series001/VEH_3_adult_42_dpi_no_reconex_cjun_19.lif_Series001_z07_ch03.tif]

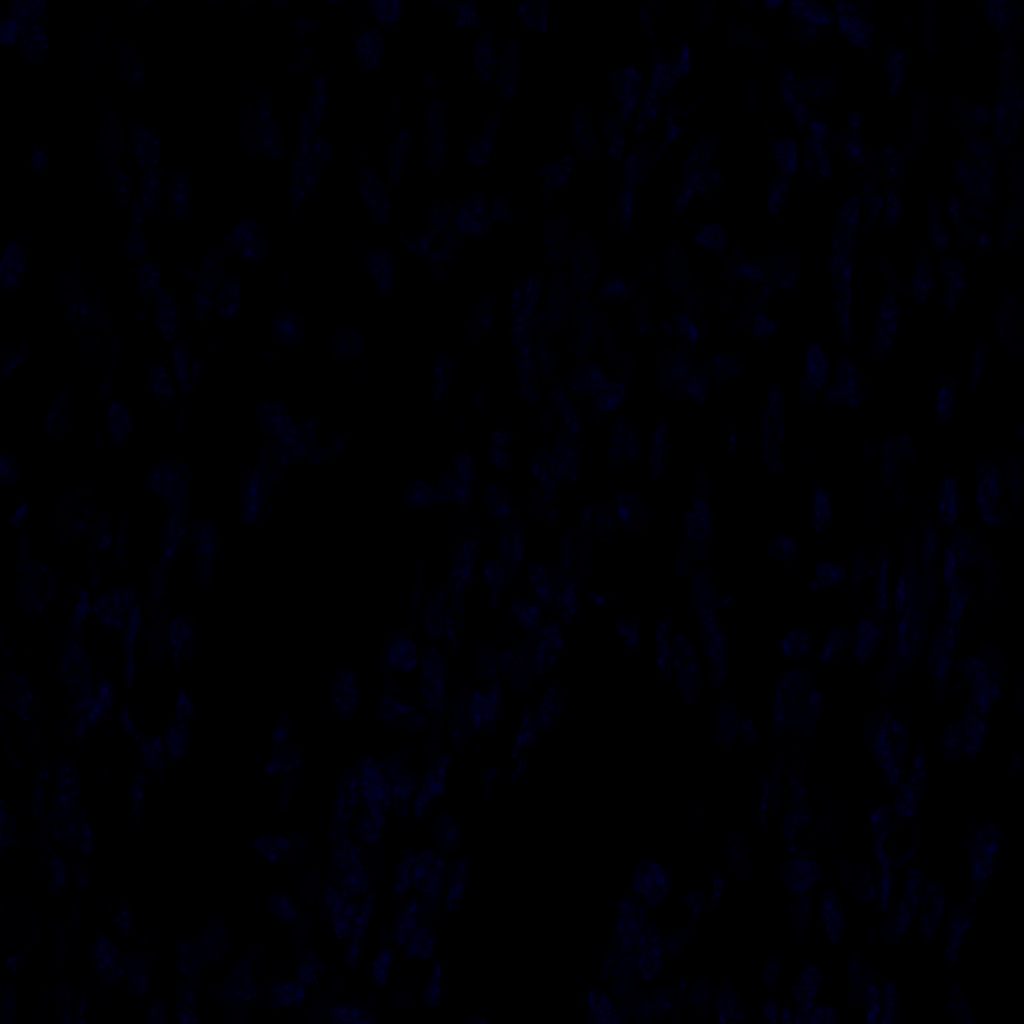

Supplement: Supplementary file 14 — Source Data for Figure 4 [file EMMM-15-e17907-s014.zip › SourceData_Fig_4/Fig_4_SourceData_images/3H/VEH_3_adult_42_dpi_no_reconex_cjun_19.lif_Series001/VEH_3_adult_42_dpi_no_reconex_cjun_19.lif_Series001_z08_ch00.tif]

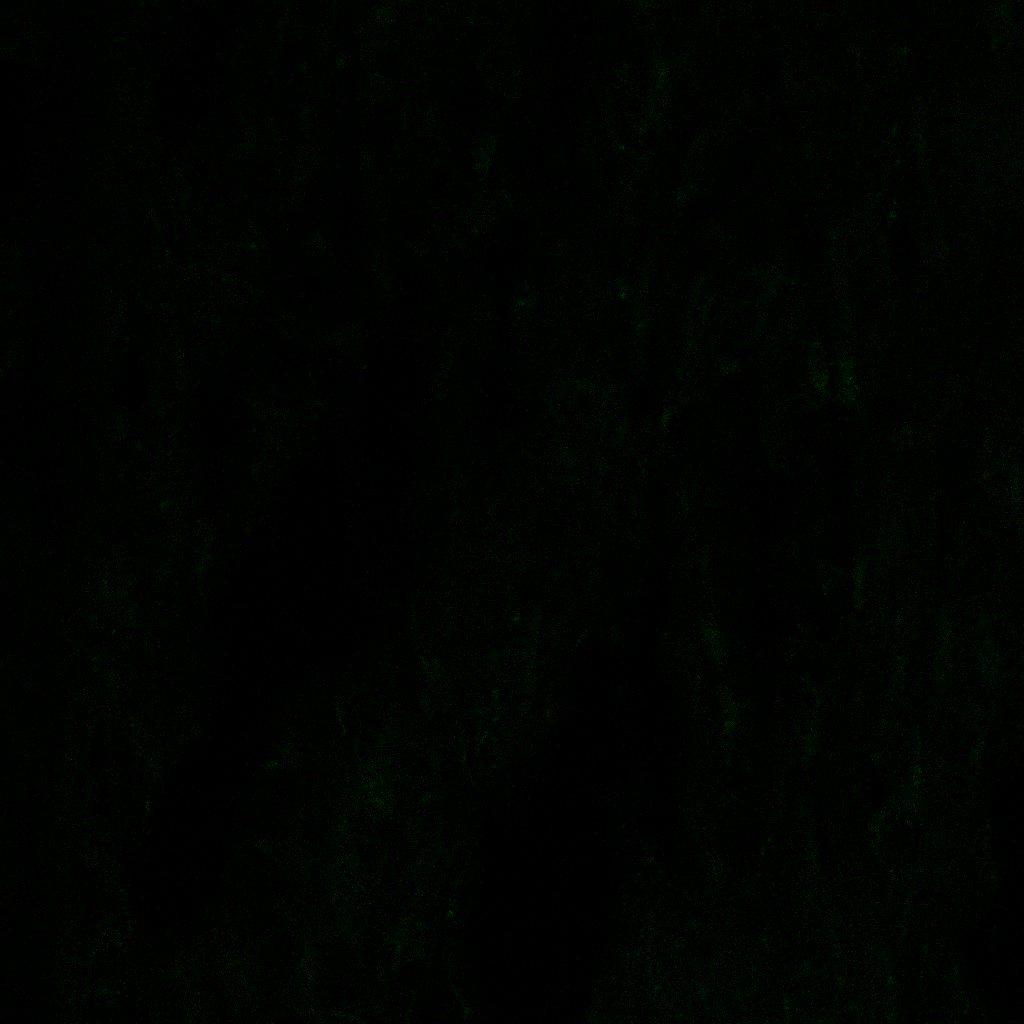

Supplement: Supplementary file 14 — Source Data for Figure 4 [file EMMM-15-e17907-s014.zip › SourceData_Fig_4/Fig_4_SourceData_images/3H/VEH_3_adult_42_dpi_no_reconex_cjun_19.lif_Series001/VEH_3_adult_42_dpi_no_reconex_cjun_19.lif_Series001_z08_ch01.tif]

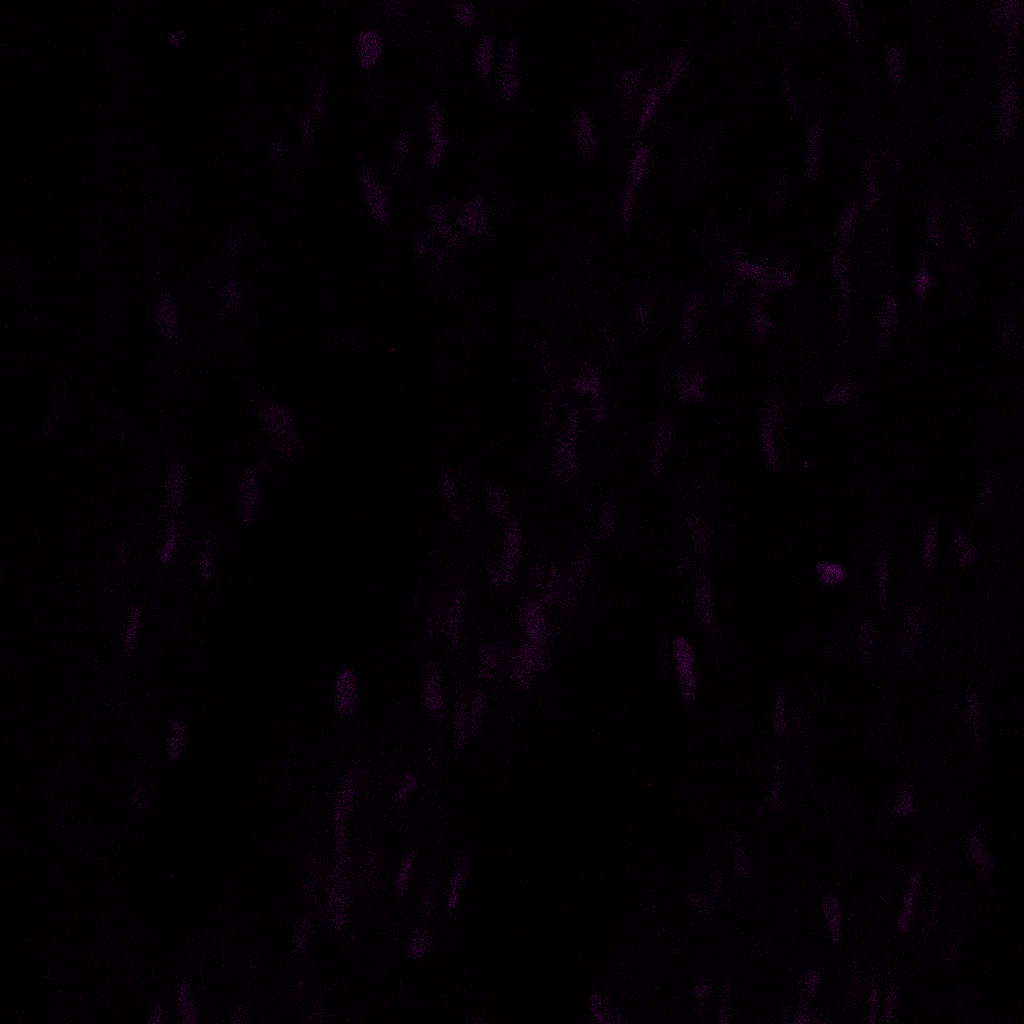

Supplement: Supplementary file 14 — Source Data for Figure 4 [file EMMM-15-e17907-s014.zip › SourceData_Fig_4/Fig_4_SourceData_images/3H/VEH_3_adult_42_dpi_no_reconex_cjun_19.lif_Series001/VEH_3_adult_42_dpi_no_reconex_cjun_19.lif_Series001_z08_ch02.tif]

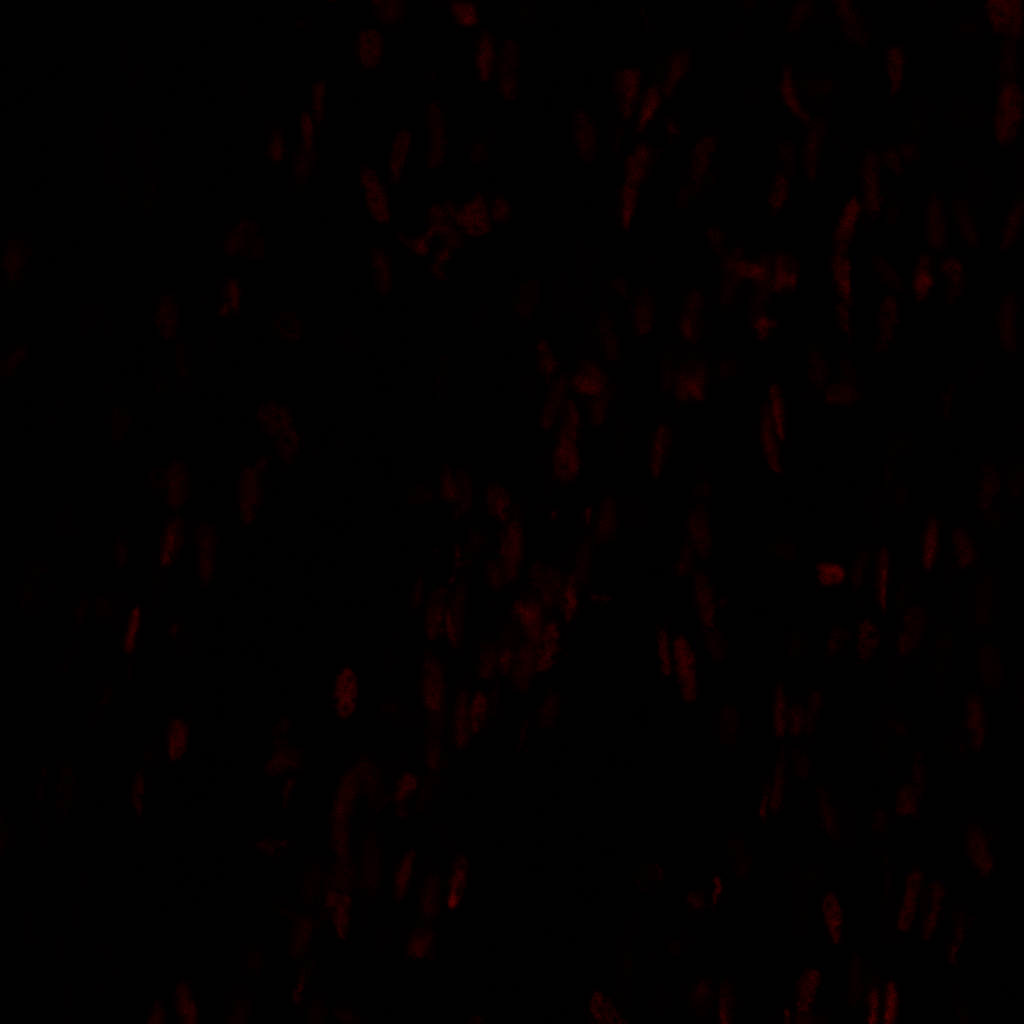

Supplement: Supplementary file 14 — Source Data for Figure 4 [file EMMM-15-e17907-s014.zip › SourceData_Fig_4/Fig_4_SourceData_images/3H/VEH_3_adult_42_dpi_no_reconex_cjun_19.lif_Series001/VEH_3_adult_42_dpi_no_reconex_cjun_19.lif_Series001_z08_ch03.tif]

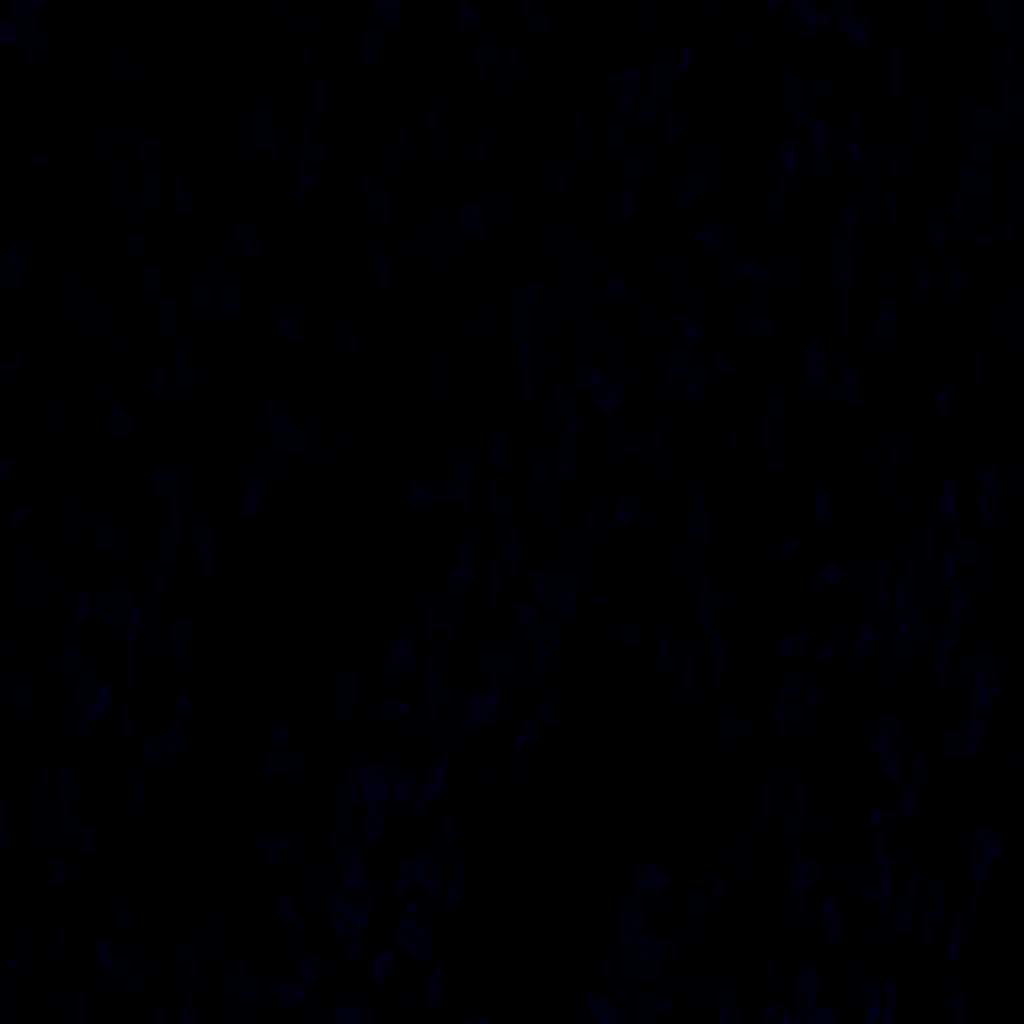

Supplement: Supplementary file 14 — Source Data for Figure 4 [file EMMM-15-e17907-s014.zip › SourceData_Fig_4/Fig_4_SourceData_images/3H/VEH_3_adult_42_dpi_no_reconex_cjun_19.lif_Series001/VEH_3_adult_42_dpi_no_reconex_cjun_19.lif_Series001_z09_ch00.tif]

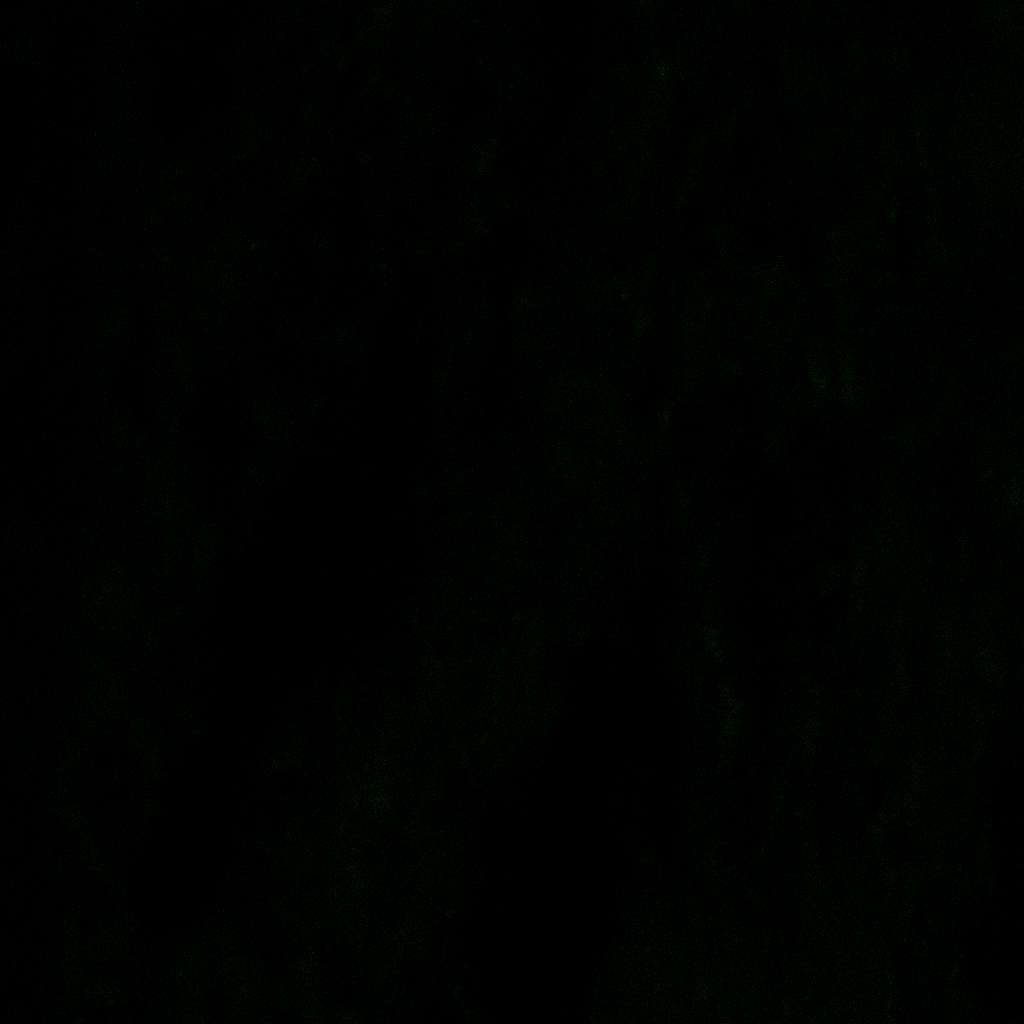

Supplement: Supplementary file 14 — Source Data for Figure 4 [file EMMM-15-e17907-s014.zip › SourceData_Fig_4/Fig_4_SourceData_images/3H/VEH_3_adult_42_dpi_no_reconex_cjun_19.lif_Series001/VEH_3_adult_42_dpi_no_reconex_cjun_19.lif_Series001_z09_ch01.tif]

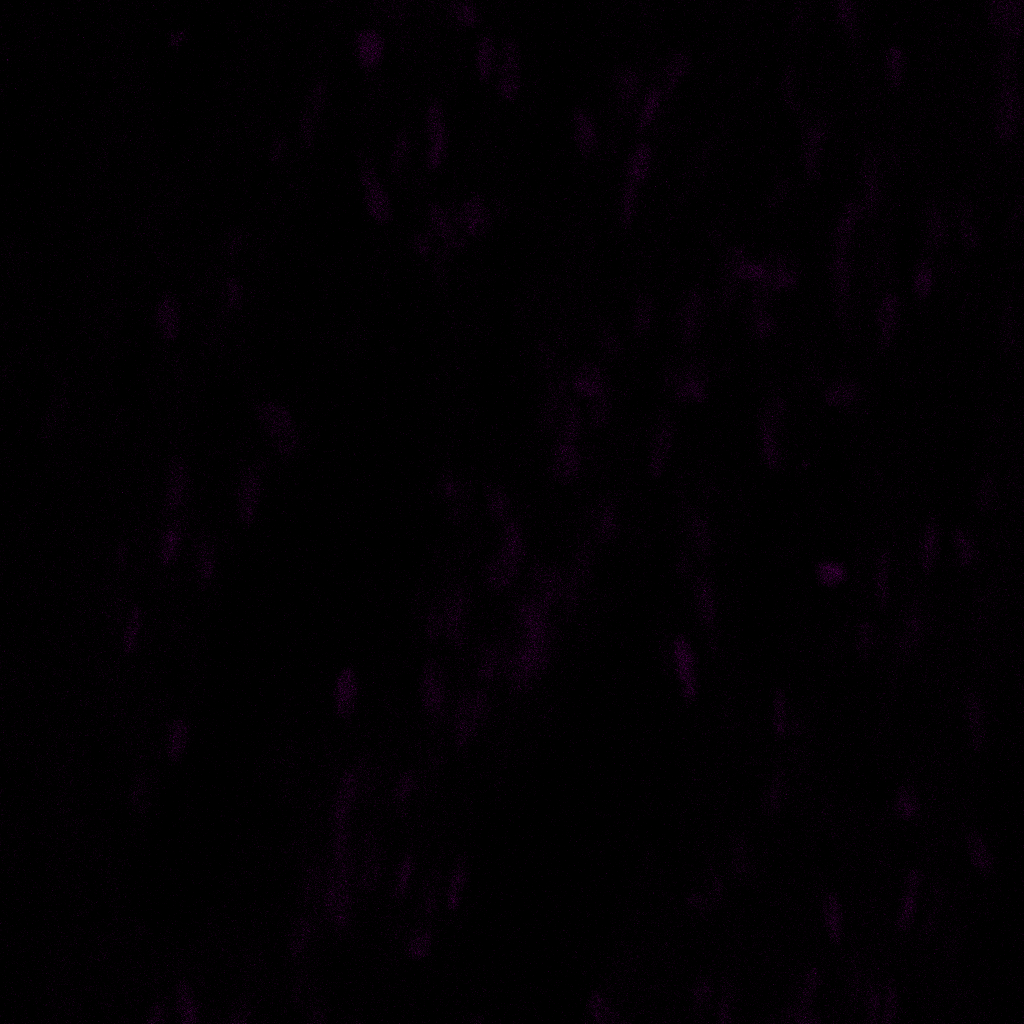

Supplement: Supplementary file 14 — Source Data for Figure 4 [file EMMM-15-e17907-s014.zip › SourceData_Fig_4/Fig_4_SourceData_images/3H/VEH_3_adult_42_dpi_no_reconex_cjun_19.lif_Series001/VEH_3_adult_42_dpi_no_reconex_cjun_19.lif_Series001_z09_ch02.tif]

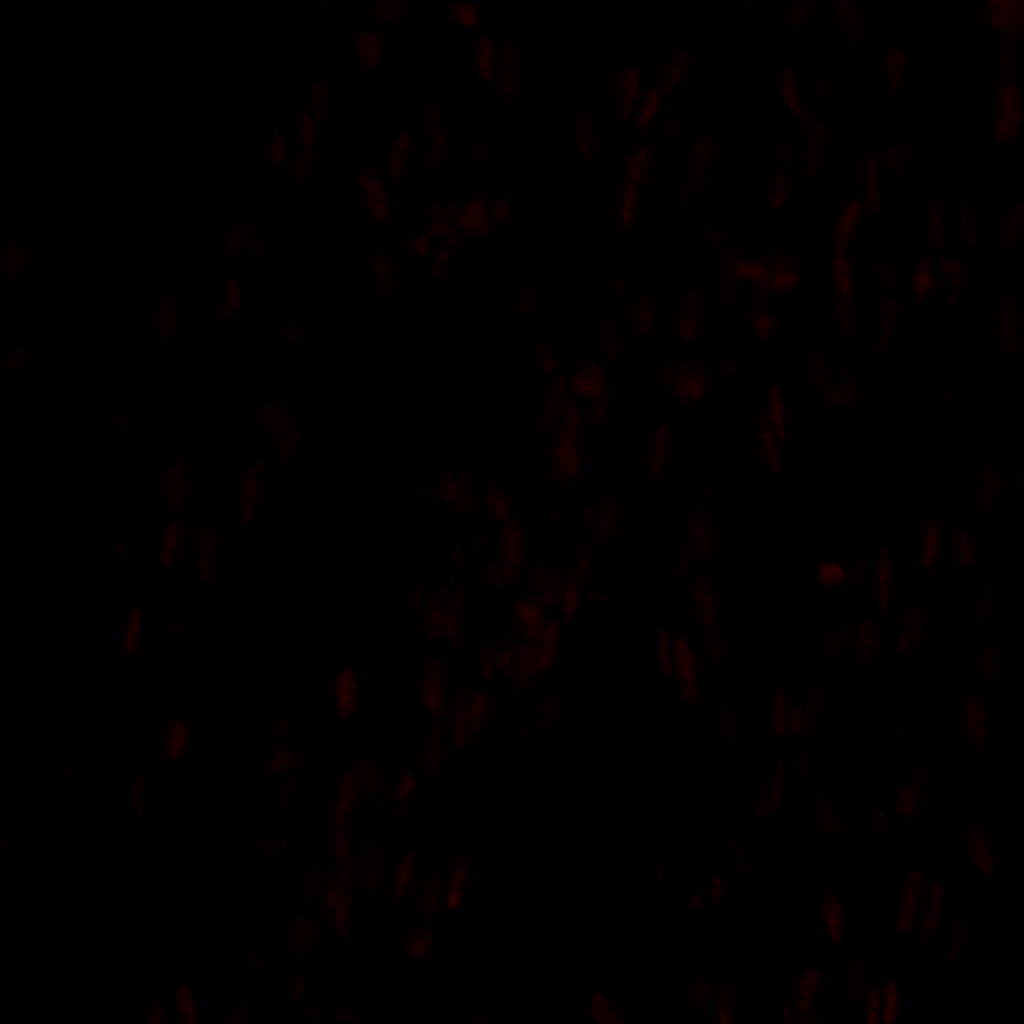

Supplement: Supplementary file 14 — Source Data for Figure 4 [file EMMM-15-e17907-s014.zip › SourceData_Fig_4/Fig_4_SourceData_images/3H/VEH_3_adult_42_dpi_no_reconex_cjun_19.lif_Series001/VEH_3_adult_42_dpi_no_reconex_cjun_19.lif_Series001_z09_ch03.tif]

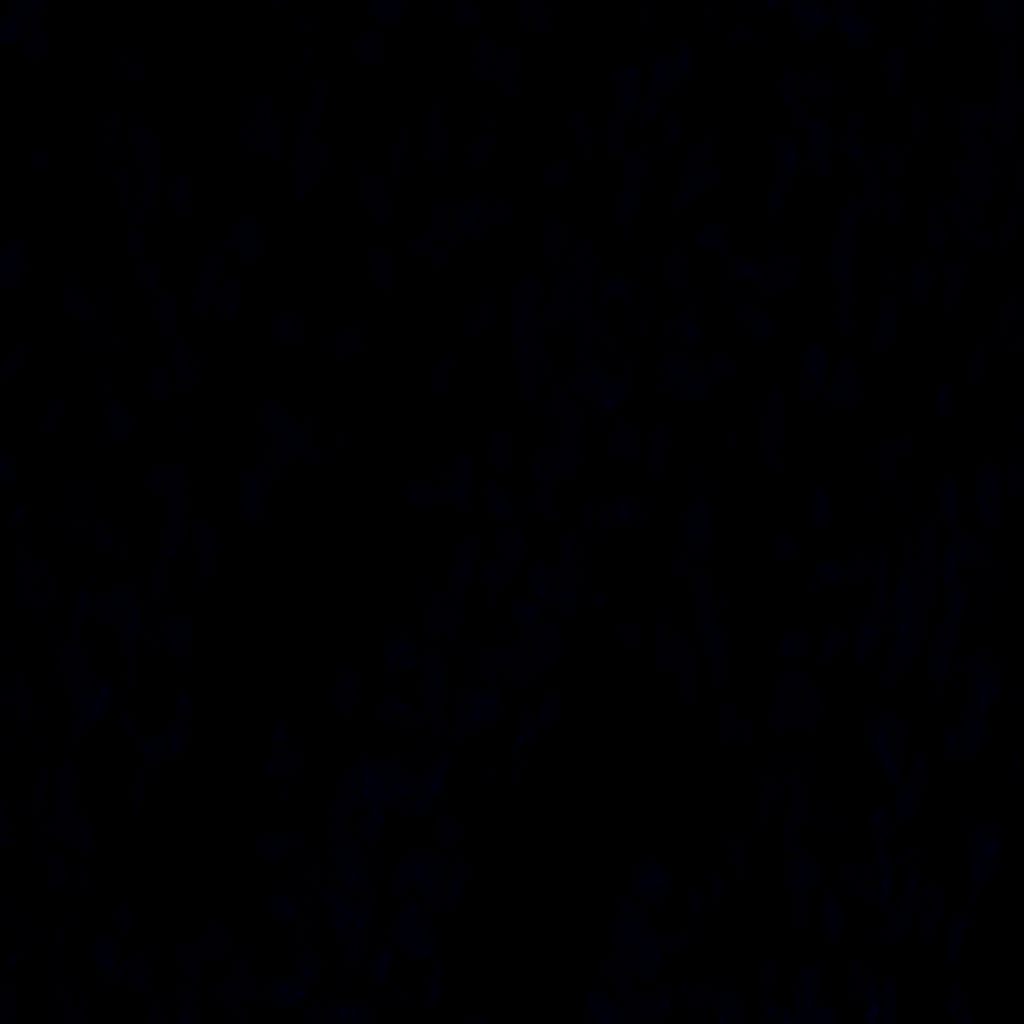

Supplement: Supplementary file 14 — Source Data for Figure 4 [file EMMM-15-e17907-s014.zip › SourceData_Fig_4/Fig_4_SourceData_images/3H/VEH_3_adult_42_dpi_no_reconex_cjun_19.lif_Series001/VEH_3_adult_42_dpi_no_reconex_cjun_19.lif_Series001_z10_ch00.tif]

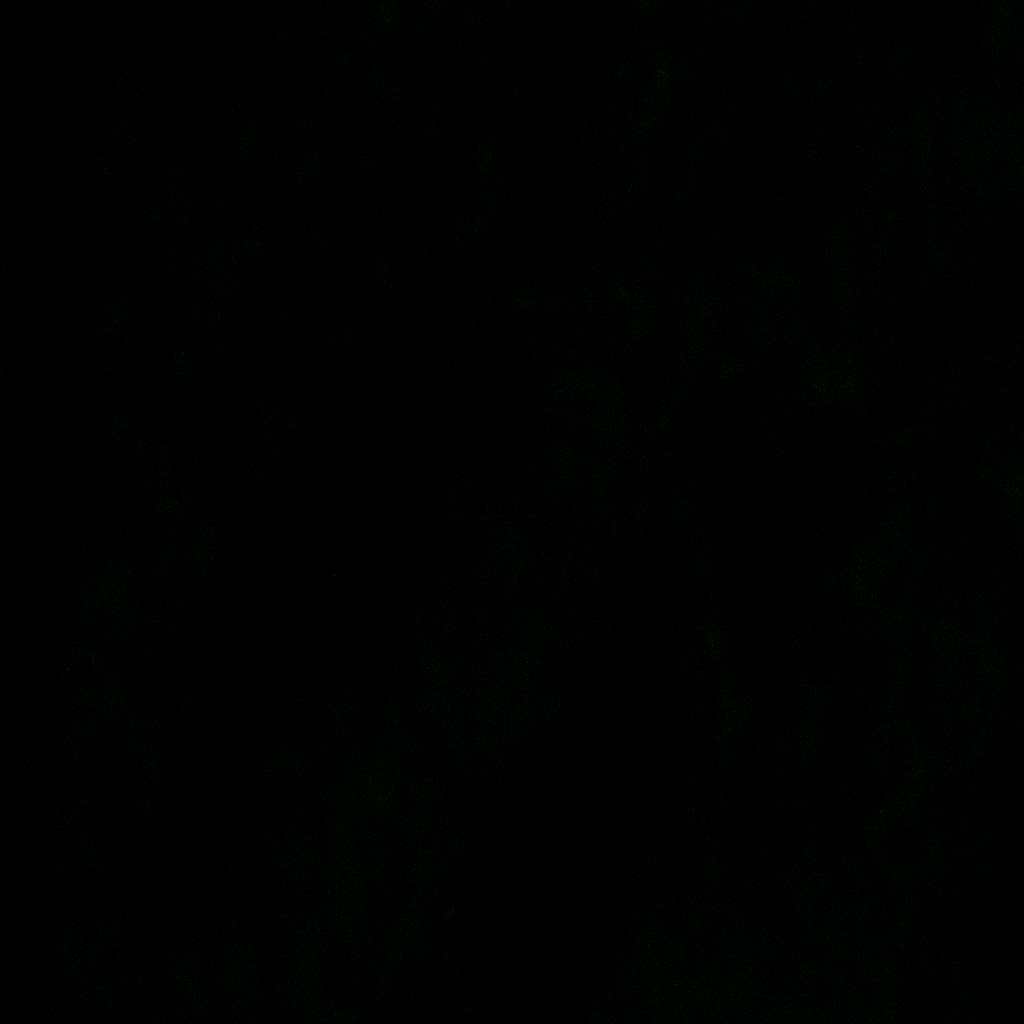

Supplement: Supplementary file 14 — Source Data for Figure 4 [file EMMM-15-e17907-s014.zip › SourceData_Fig_4/Fig_4_SourceData_images/3H/VEH_3_adult_42_dpi_no_reconex_cjun_19.lif_Series001/VEH_3_adult_42_dpi_no_reconex_cjun_19.lif_Series001_z10_ch01.tif]

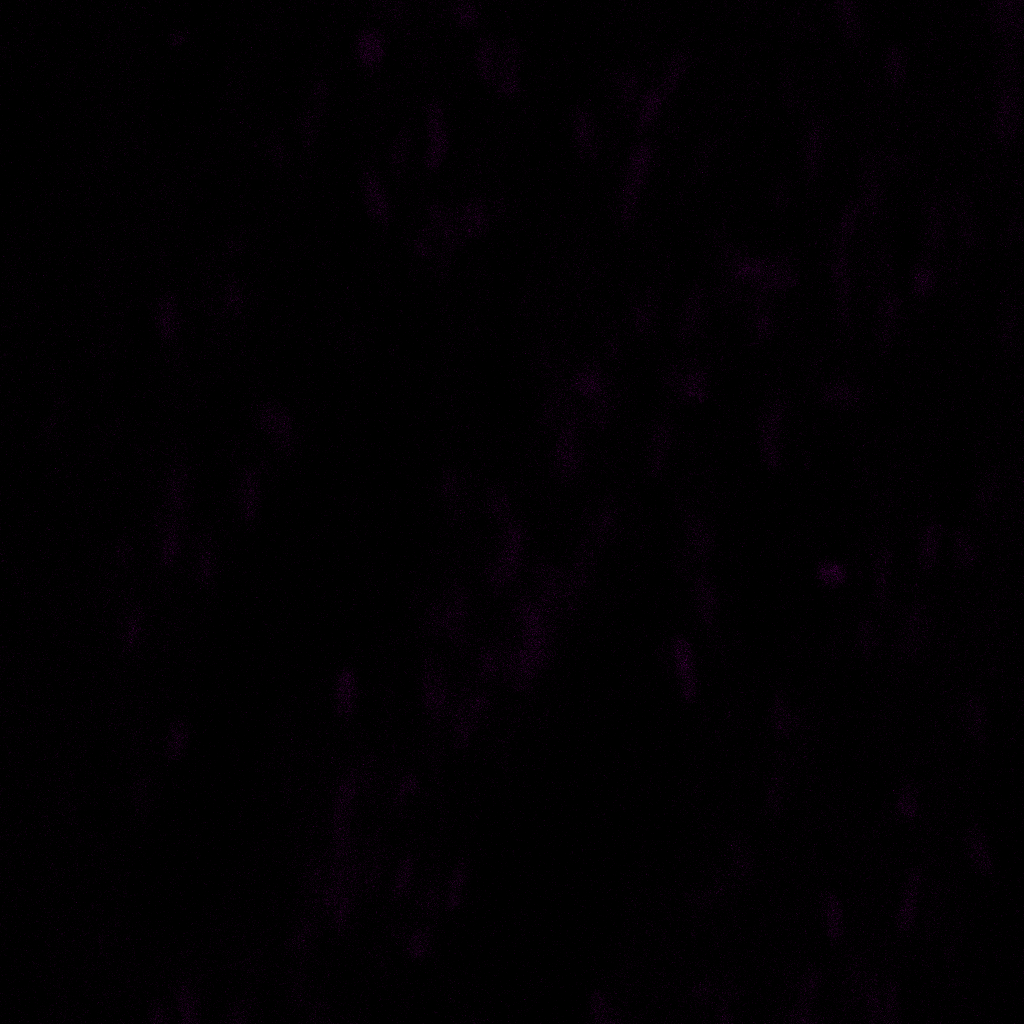

Supplement: Supplementary file 14 — Source Data for Figure 4 [file EMMM-15-e17907-s014.zip › SourceData_Fig_4/Fig_4_SourceData_images/3H/VEH_3_adult_42_dpi_no_reconex_cjun_19.lif_Series001/VEH_3_adult_42_dpi_no_reconex_cjun_19.lif_Series001_z10_ch02.tif]

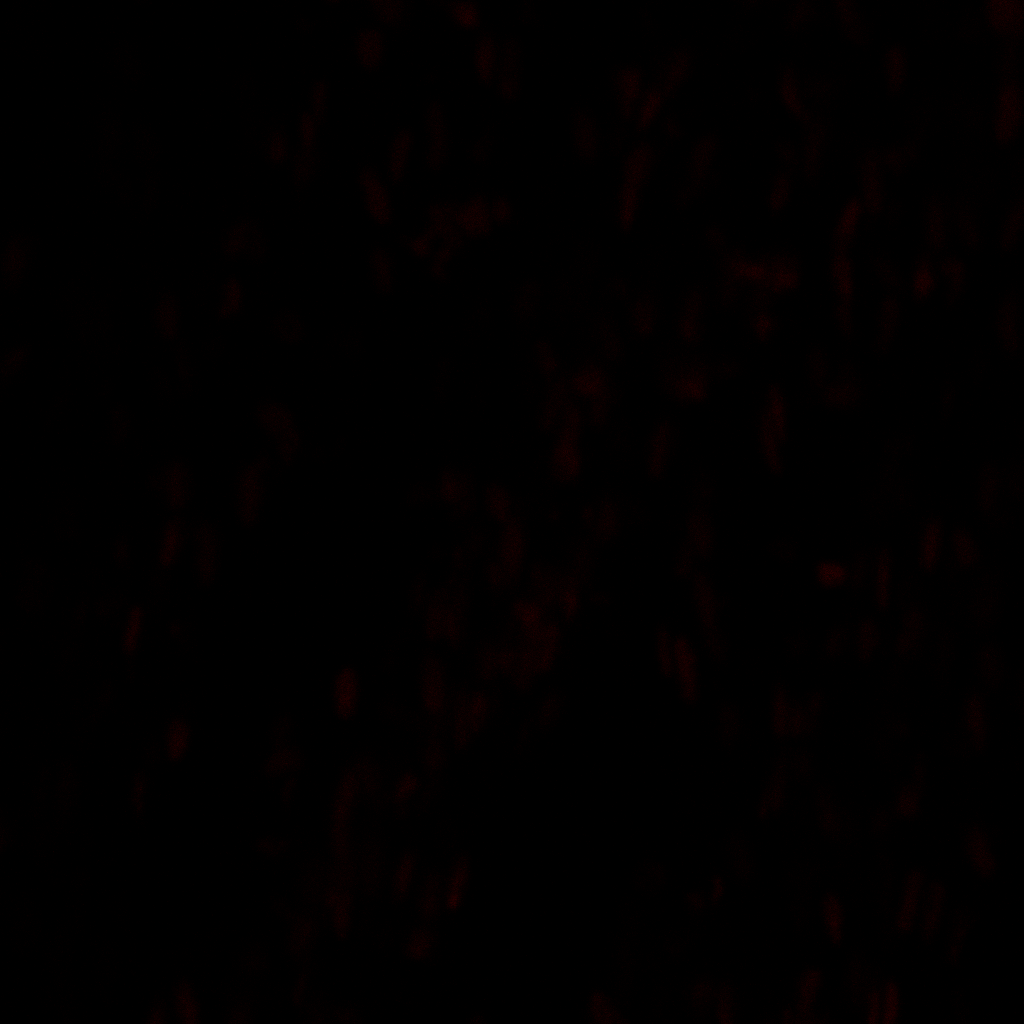

Supplement: Supplementary file 14 — Source Data for Figure 4 [file EMMM-15-e17907-s014.zip › SourceData_Fig_4/Fig_4_SourceData_images/3H/VEH_3_adult_42_dpi_no_reconex_cjun_19.lif_Series001/VEH_3_adult_42_dpi_no_reconex_cjun_19.lif_Series001_z10_ch03.tif]

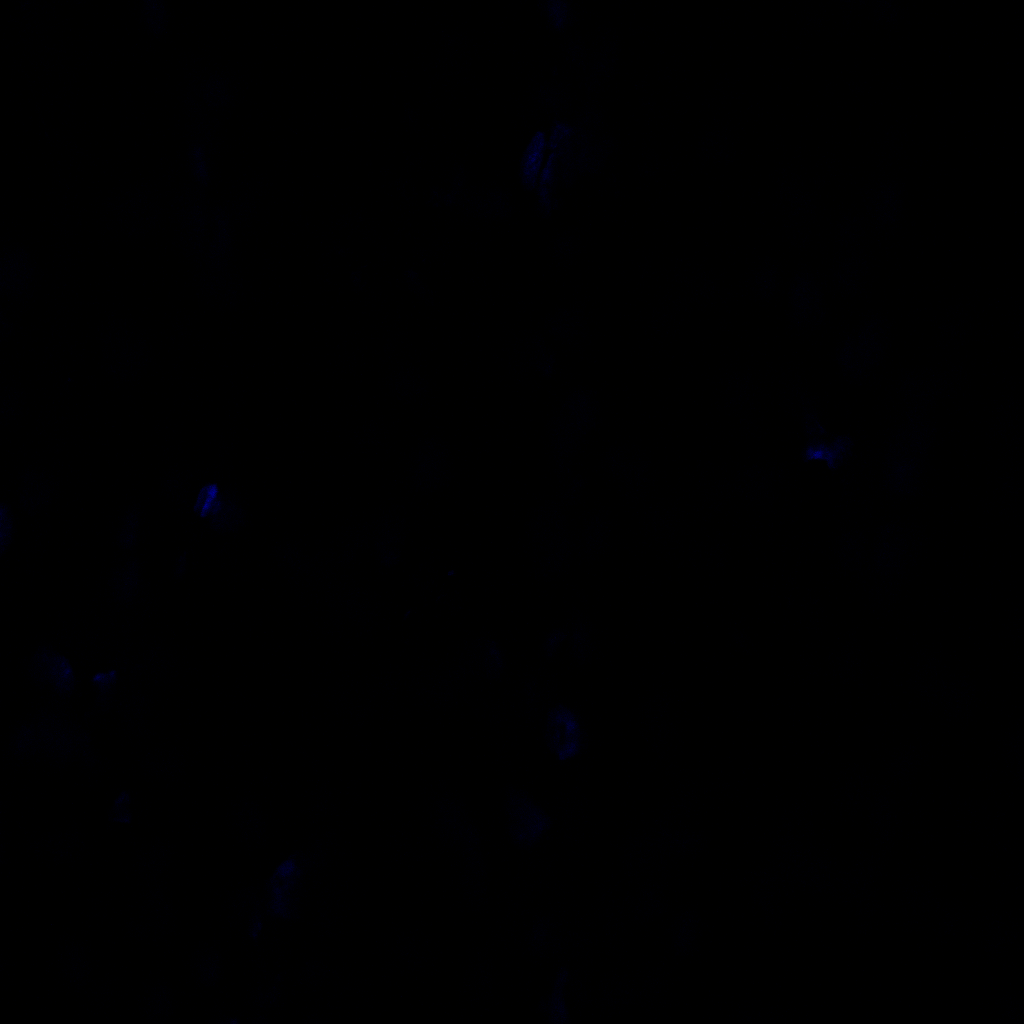

Supplement: Supplementary file 14 — Source Data for Figure 4 [file EMMM-15-e17907-s014.zip › SourceData_Fig_4/Fig_4_SourceData_images/3H/VEH_III_aged_12_dpi_no_reconex_cjun_19.lif_Series001/VEH_III_aged_12_dpi_no_reconex_cjun_19.lif_Series001_z00_ch00.tif]

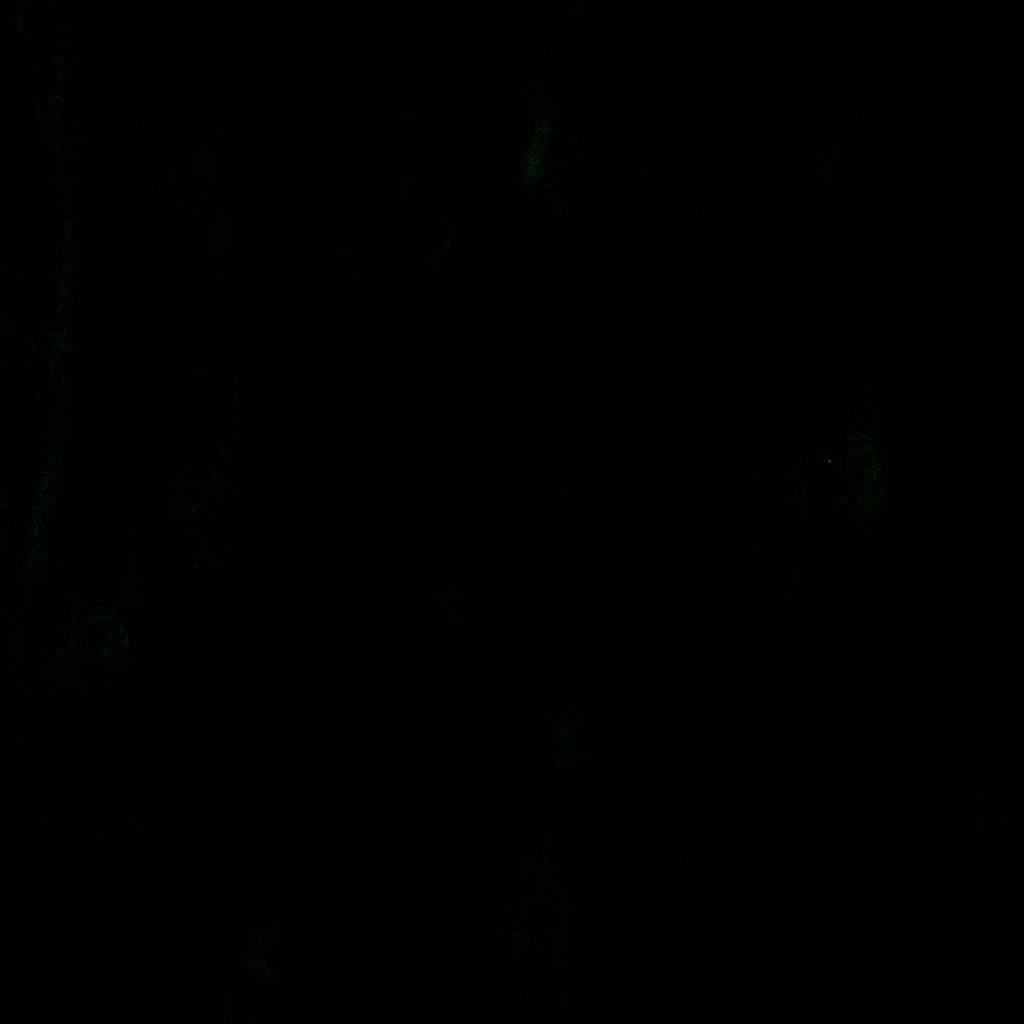

Supplement: Supplementary file 14 — Source Data for Figure 4 [file EMMM-15-e17907-s014.zip › SourceData_Fig_4/Fig_4_SourceData_images/3H/VEH_III_aged_12_dpi_no_reconex_cjun_19.lif_Series001/VEH_III_aged_12_dpi_no_reconex_cjun_19.lif_Series001_z00_ch01.tif]

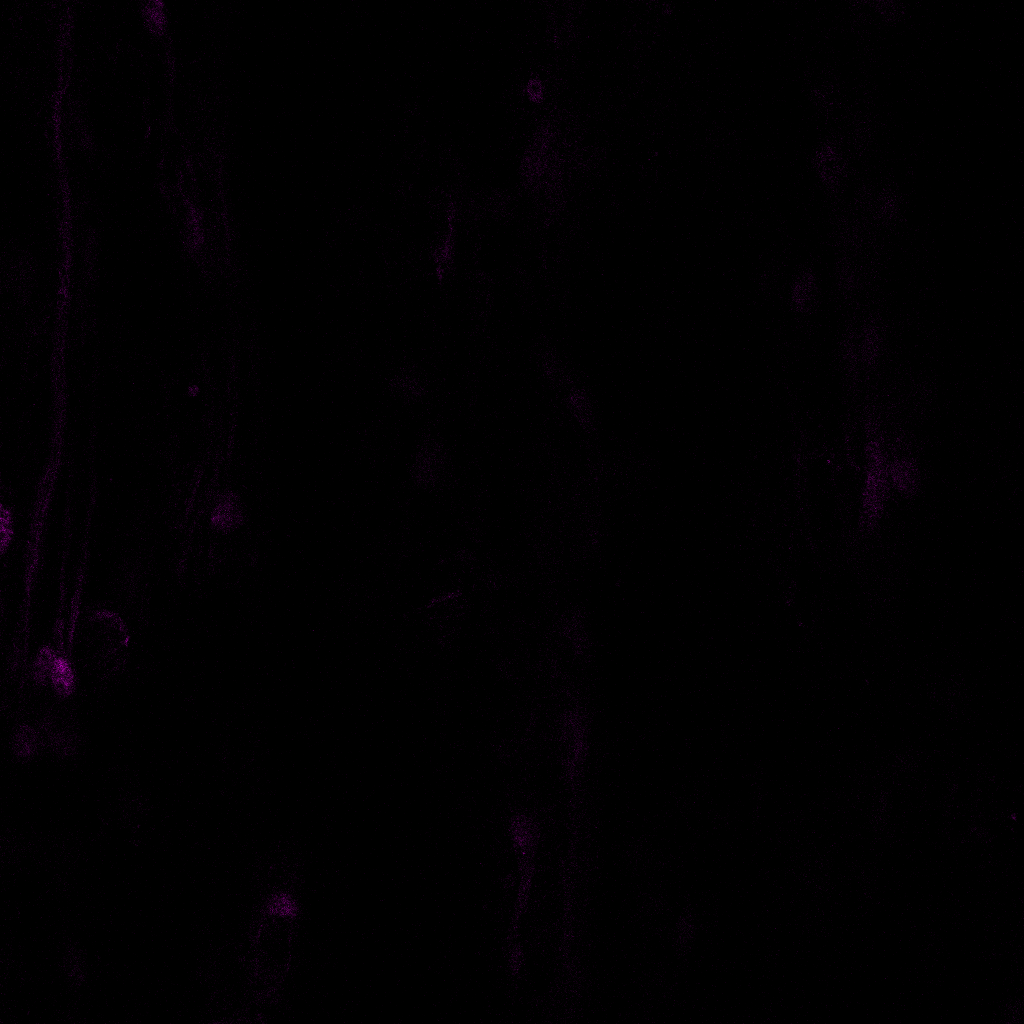

Supplement: Supplementary file 14 — Source Data for Figure 4 [file EMMM-15-e17907-s014.zip › SourceData_Fig_4/Fig_4_SourceData_images/3H/VEH_III_aged_12_dpi_no_reconex_cjun_19.lif_Series001/VEH_III_aged_12_dpi_no_reconex_cjun_19.lif_Series001_z00_ch02.tif]

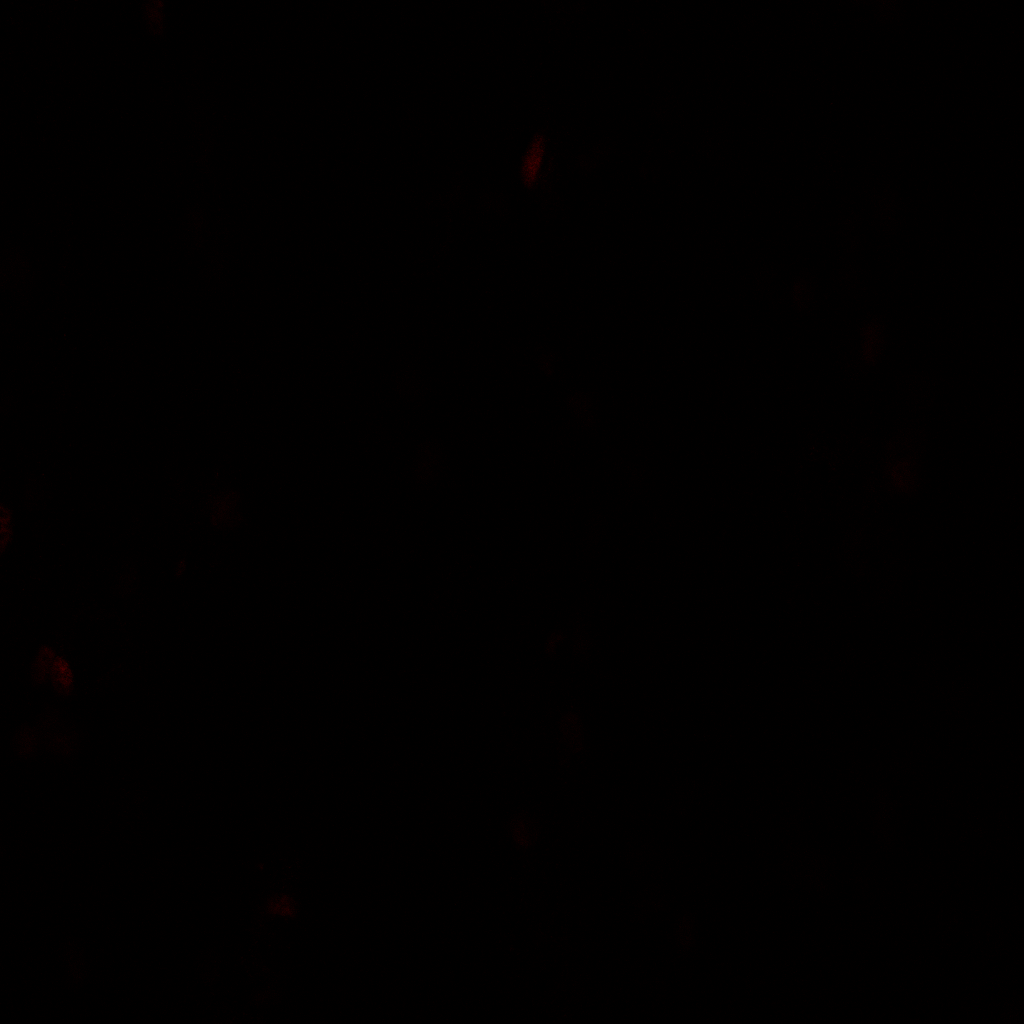

Supplement: Supplementary file 14 — Source Data for Figure 4 [file EMMM-15-e17907-s014.zip › SourceData_Fig_4/Fig_4_SourceData_images/3H/VEH_III_aged_12_dpi_no_reconex_cjun_19.lif_Series001/VEH_III_aged_12_dpi_no_reconex_cjun_19.lif_Series001_z00_ch03.tif]

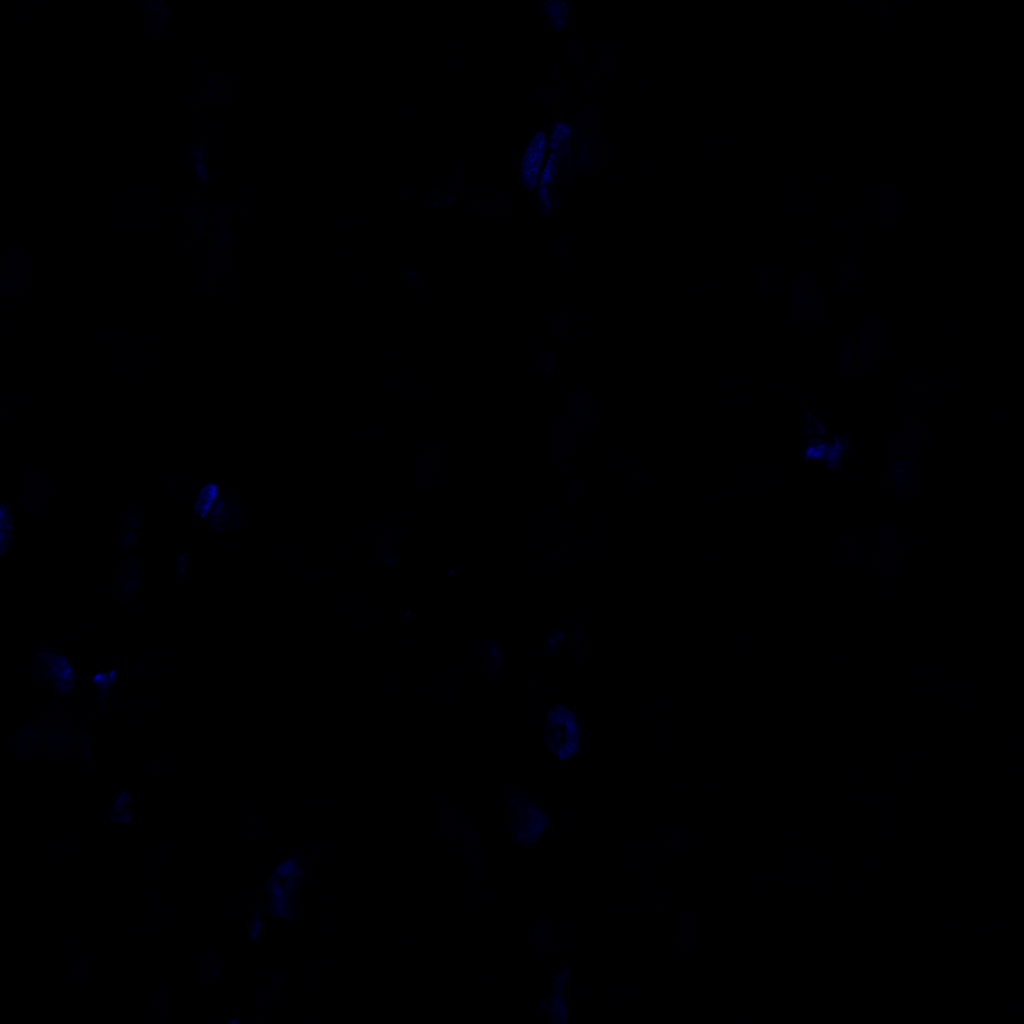

Supplement: Supplementary file 14 — Source Data for Figure 4 [file EMMM-15-e17907-s014.zip › SourceData_Fig_4/Fig_4_SourceData_images/3H/VEH_III_aged_12_dpi_no_reconex_cjun_19.lif_Series001/VEH_III_aged_12_dpi_no_reconex_cjun_19.lif_Series001_z01_ch00.tif]

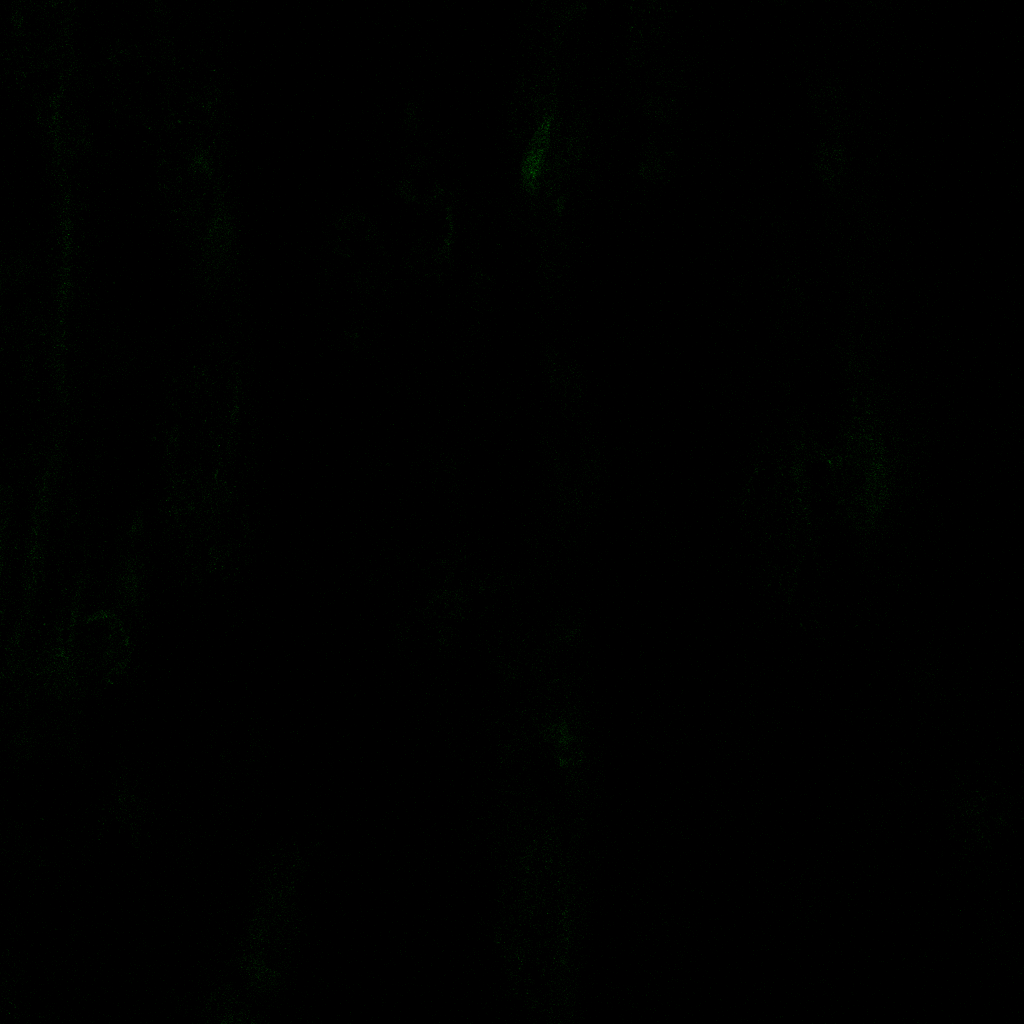

Supplement: Supplementary file 14 — Source Data for Figure 4 [file EMMM-15-e17907-s014.zip › SourceData_Fig_4/Fig_4_SourceData_images/3H/VEH_III_aged_12_dpi_no_reconex_cjun_19.lif_Series001/VEH_III_aged_12_dpi_no_reconex_cjun_19.lif_Series001_z01_ch01.tif]

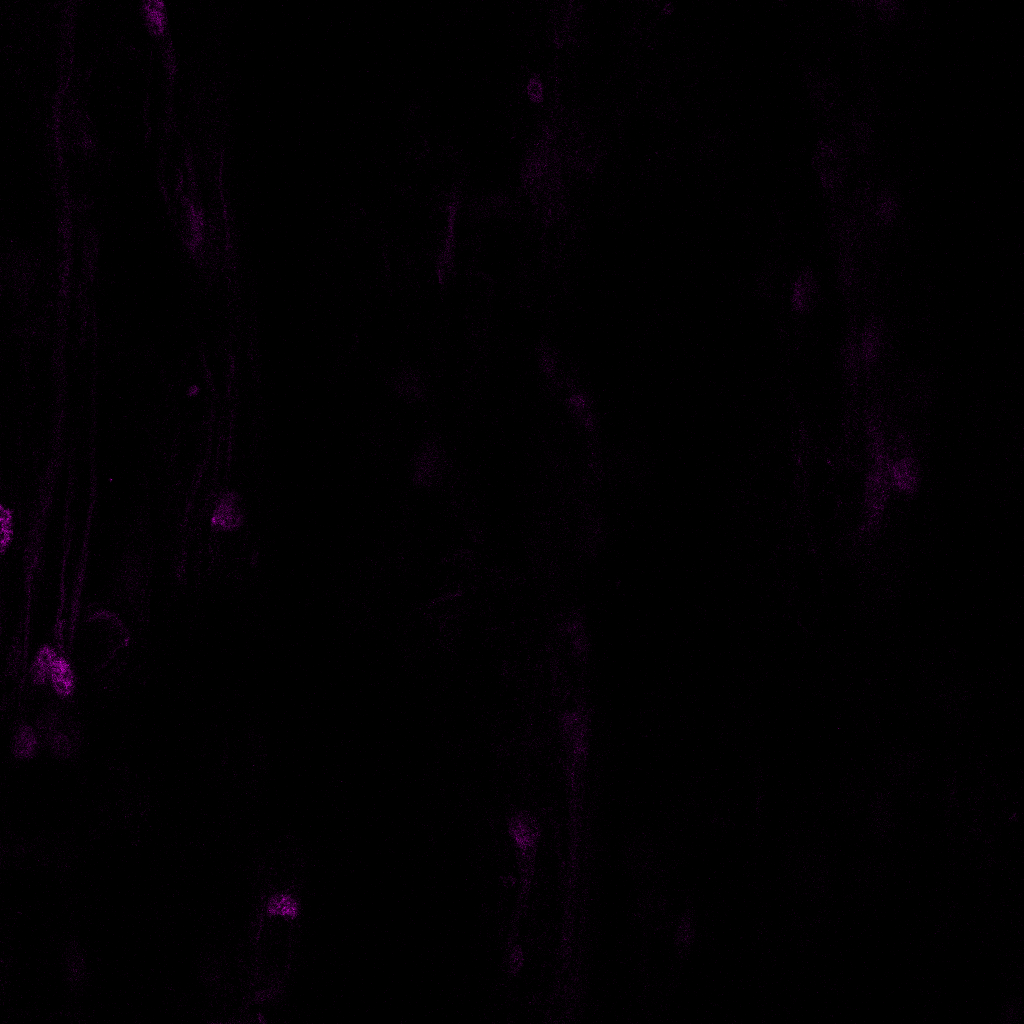

Supplement: Supplementary file 14 — Source Data for Figure 4 [file EMMM-15-e17907-s014.zip › SourceData_Fig_4/Fig_4_SourceData_images/3H/VEH_III_aged_12_dpi_no_reconex_cjun_19.lif_Series001/VEH_III_aged_12_dpi_no_reconex_cjun_19.lif_Series001_z01_ch02.tif]

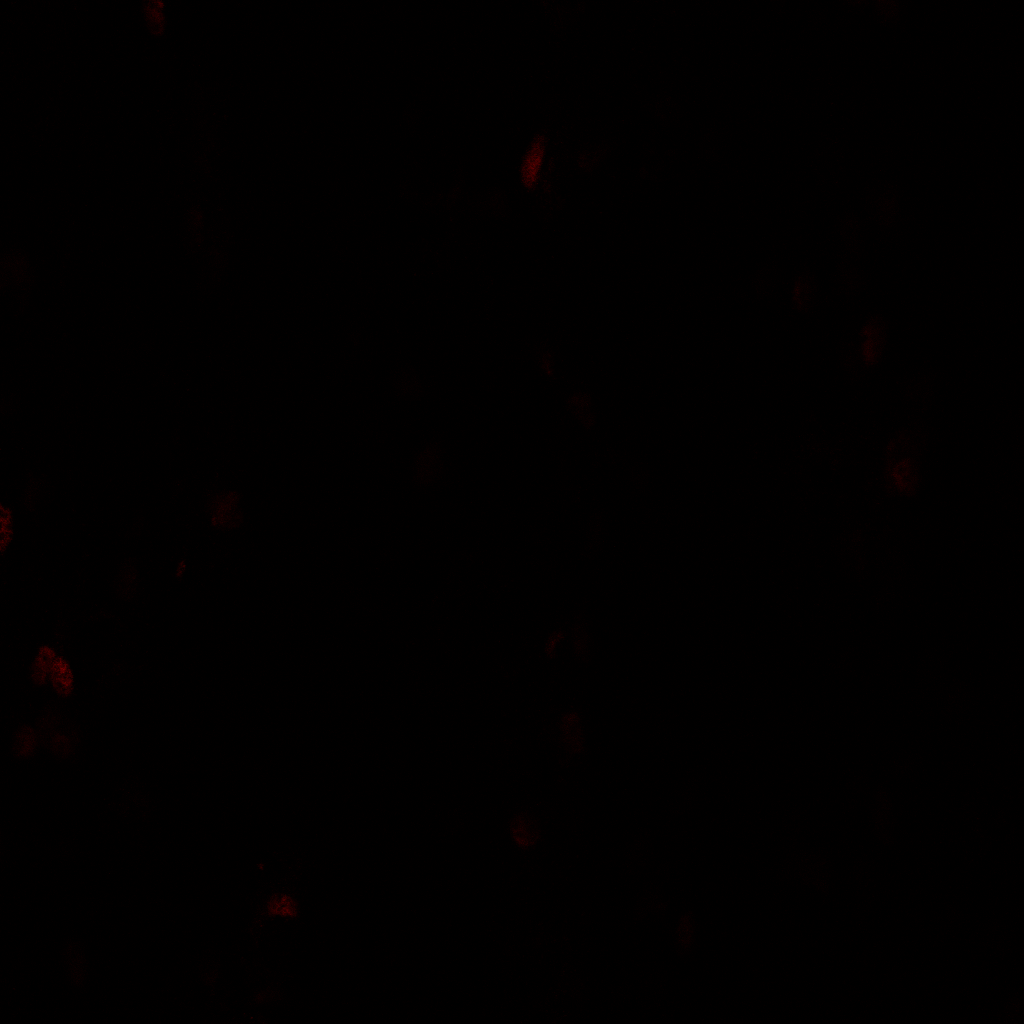

Supplement: Supplementary file 14 — Source Data for Figure 4 [file EMMM-15-e17907-s014.zip › SourceData_Fig_4/Fig_4_SourceData_images/3H/VEH_III_aged_12_dpi_no_reconex_cjun_19.lif_Series001/VEH_III_aged_12_dpi_no_reconex_cjun_19.lif_Series001_z01_ch03.tif]

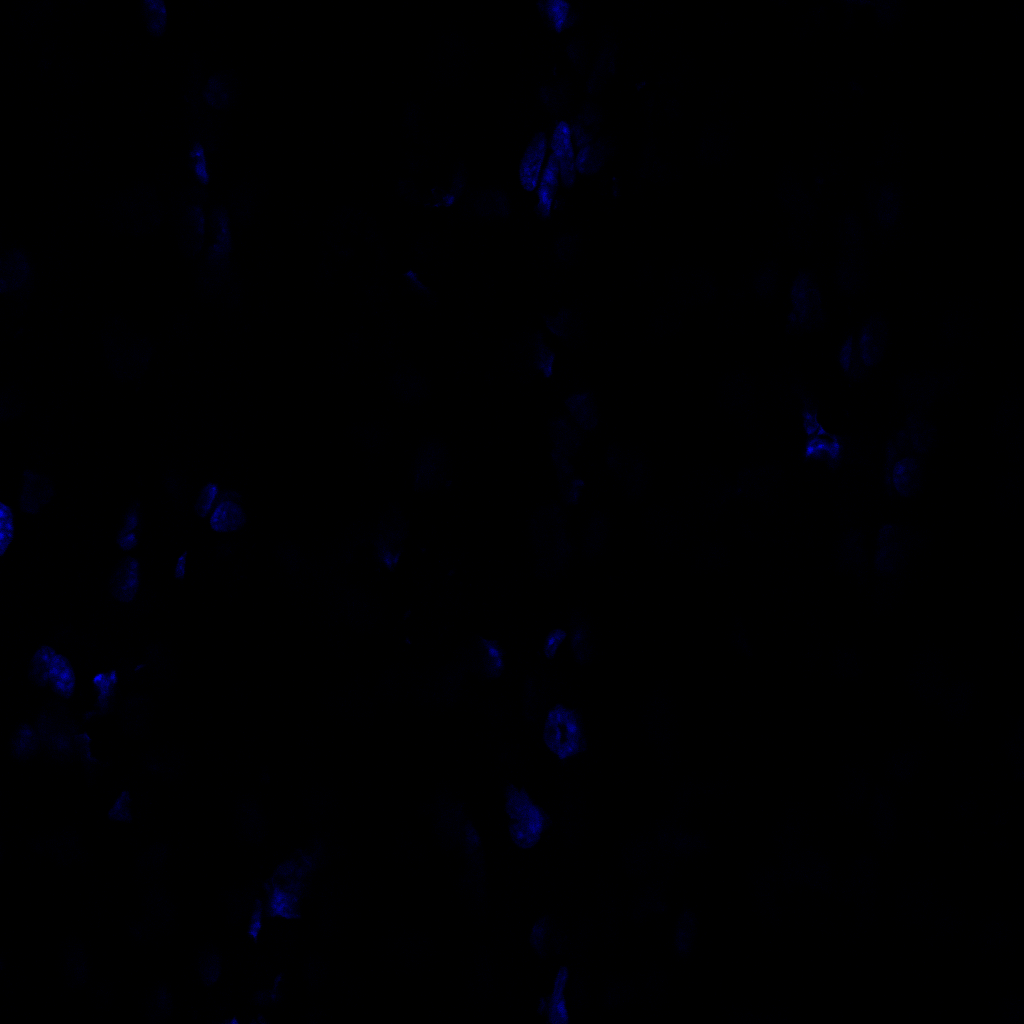

Supplement: Supplementary file 14 — Source Data for Figure 4 [file EMMM-15-e17907-s014.zip › SourceData_Fig_4/Fig_4_SourceData_images/3H/VEH_III_aged_12_dpi_no_reconex_cjun_19.lif_Series001/VEH_III_aged_12_dpi_no_reconex_cjun_19.lif_Series001_z02_ch00.tif]

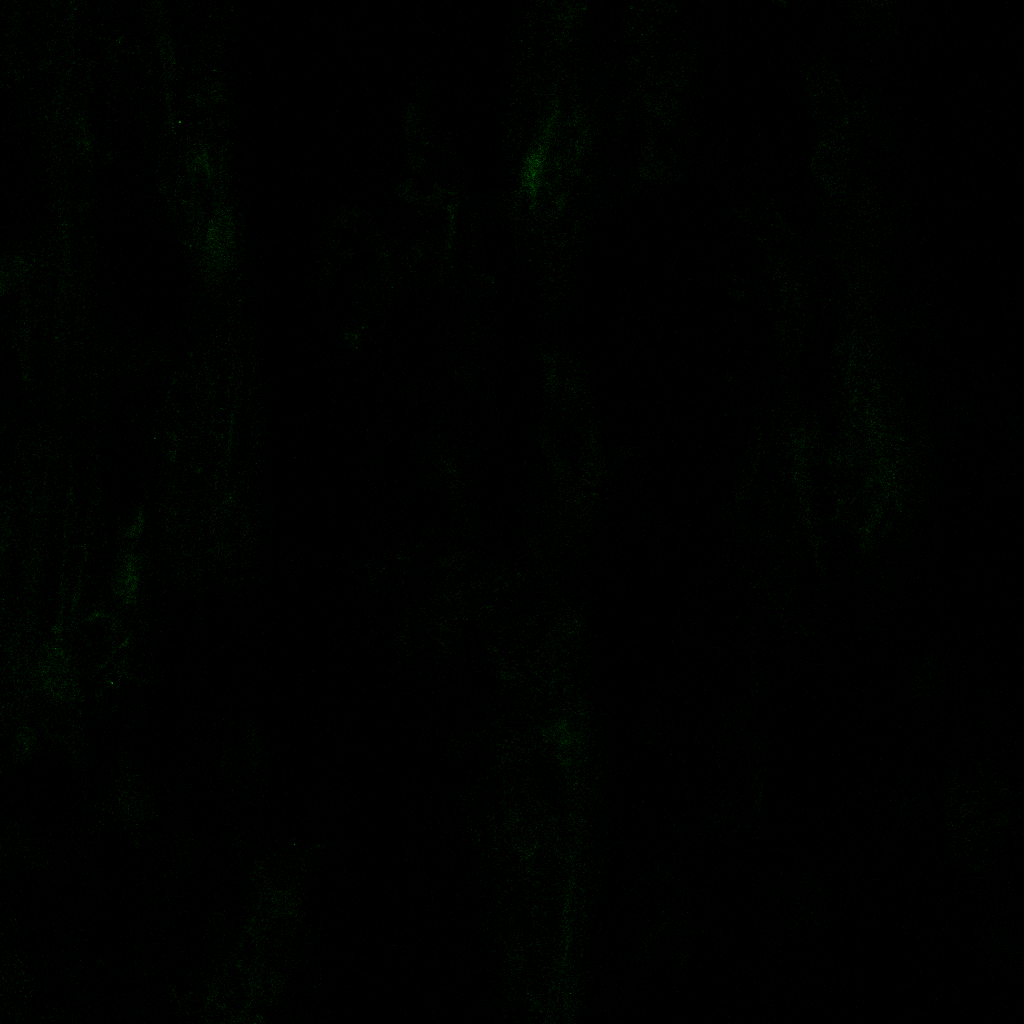

Supplement: Supplementary file 14 — Source Data for Figure 4 [file EMMM-15-e17907-s014.zip › SourceData_Fig_4/Fig_4_SourceData_images/3H/VEH_III_aged_12_dpi_no_reconex_cjun_19.lif_Series001/VEH_III_aged_12_dpi_no_reconex_cjun_19.lif_Series001_z02_ch01.tif]

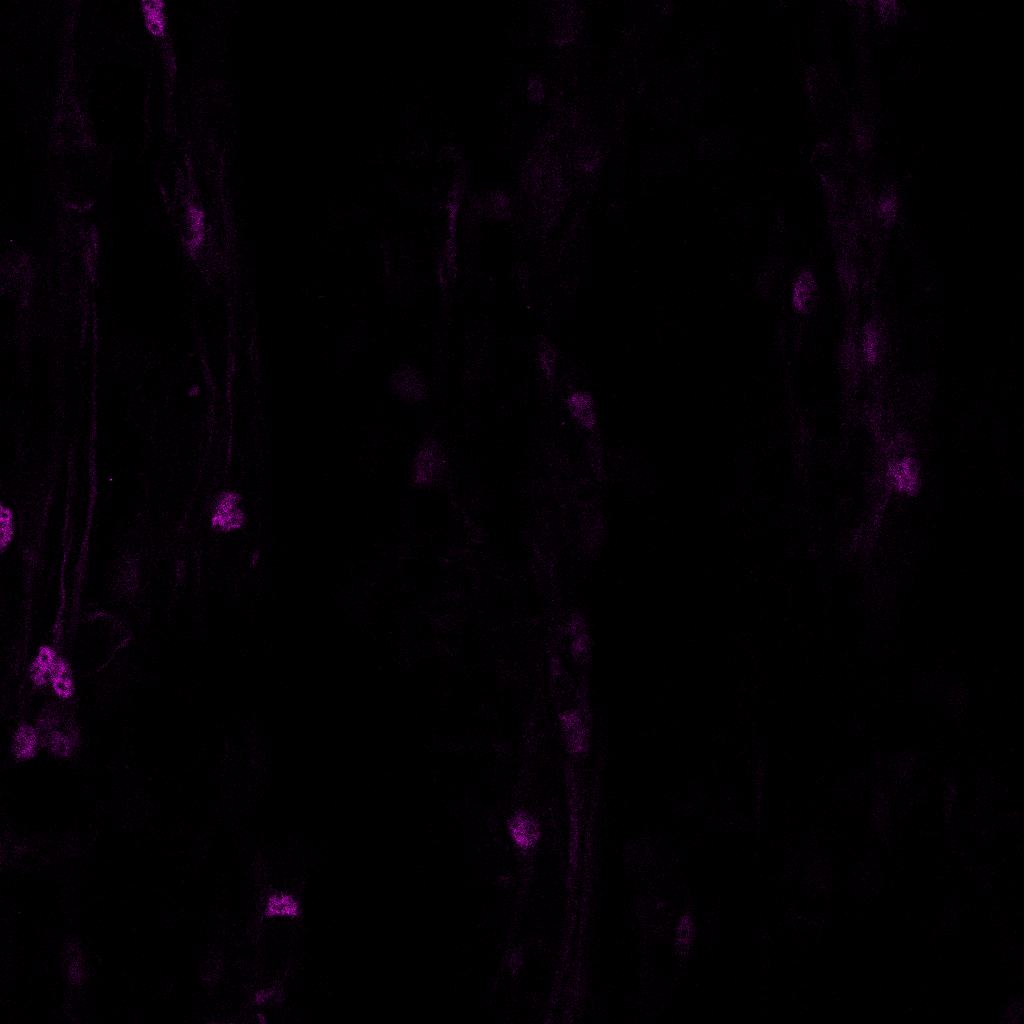

Supplement: Supplementary file 14 — Source Data for Figure 4 [file EMMM-15-e17907-s014.zip › SourceData_Fig_4/Fig_4_SourceData_images/3H/VEH_III_aged_12_dpi_no_reconex_cjun_19.lif_Series001/VEH_III_aged_12_dpi_no_reconex_cjun_19.lif_Series001_z02_ch02.tif]

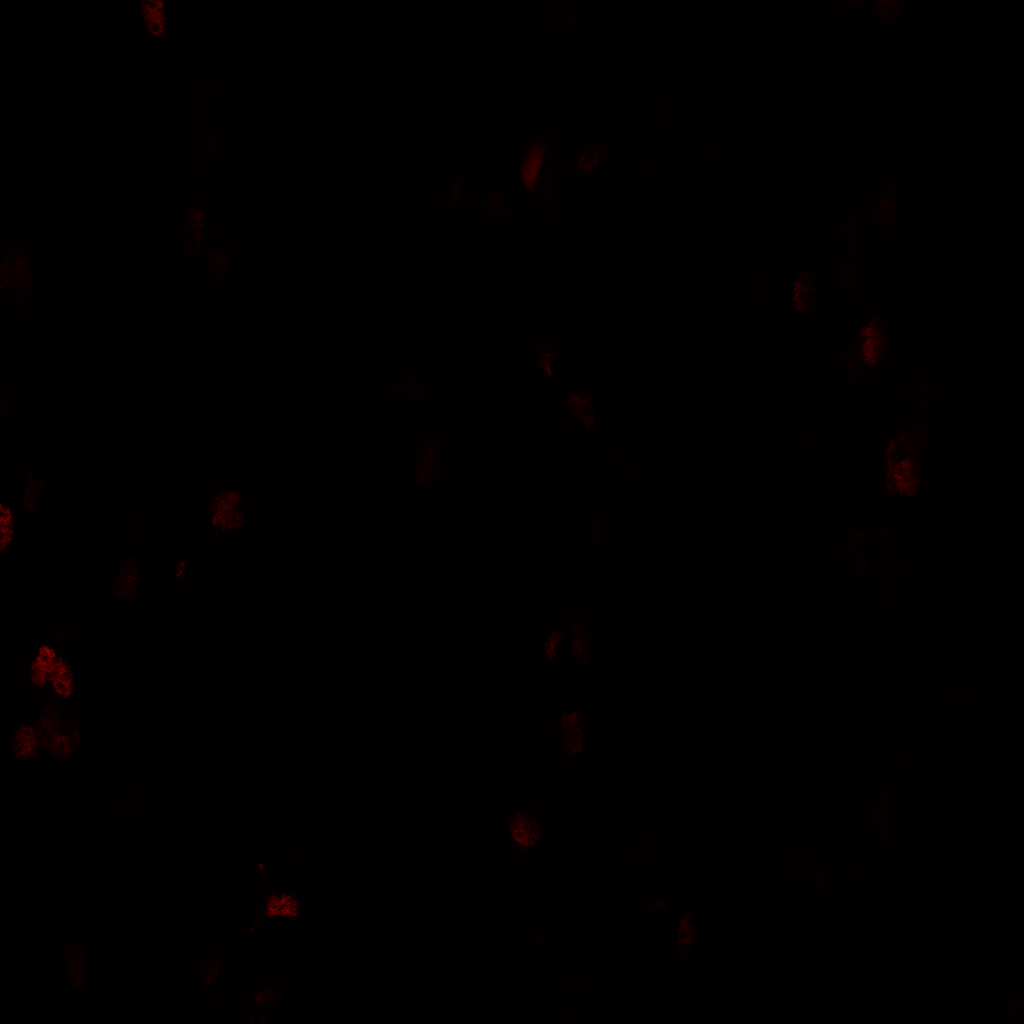

Supplement: Supplementary file 14 — Source Data for Figure 4 [file EMMM-15-e17907-s014.zip › SourceData_Fig_4/Fig_4_SourceData_images/3H/VEH_III_aged_12_dpi_no_reconex_cjun_19.lif_Series001/VEH_III_aged_12_dpi_no_reconex_cjun_19.lif_Series001_z02_ch03.tif]

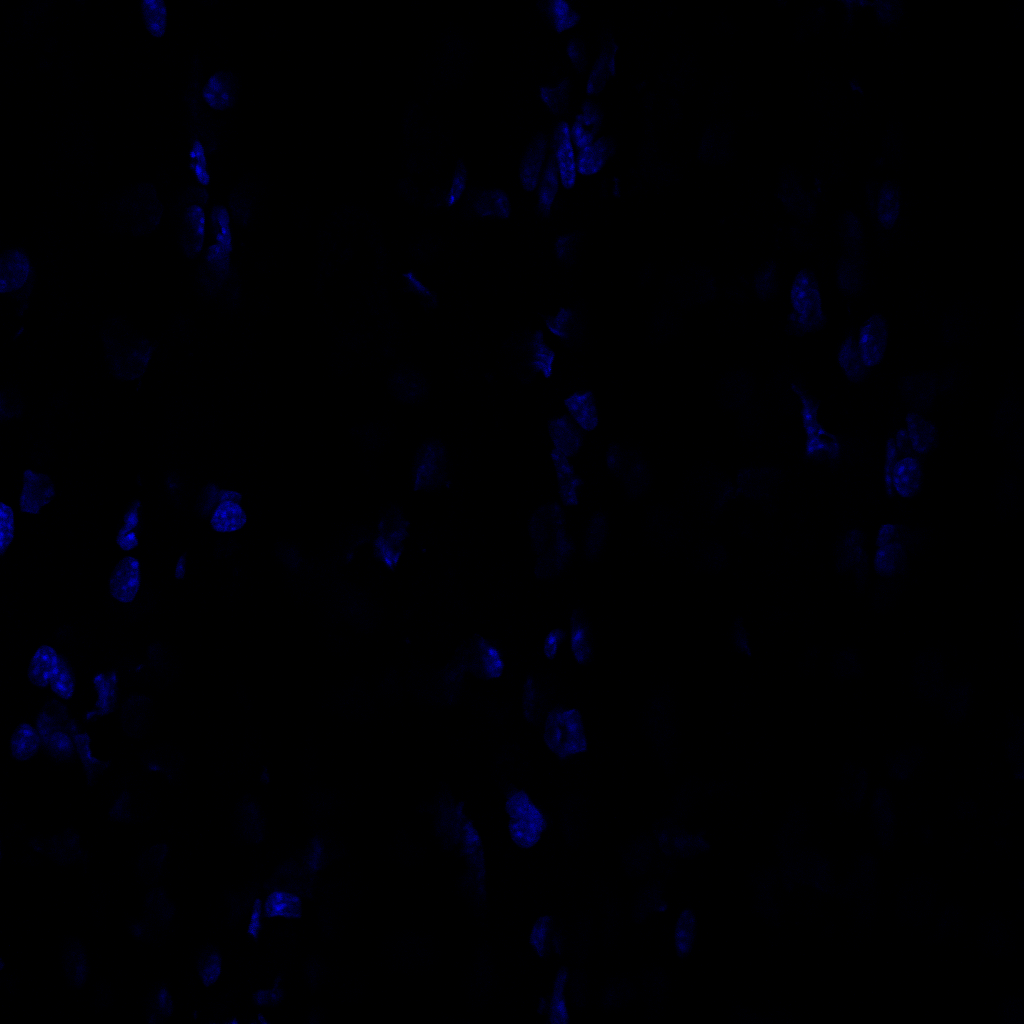

Supplement: Supplementary file 14 — Source Data for Figure 4 [file EMMM-15-e17907-s014.zip › SourceData_Fig_4/Fig_4_SourceData_images/3H/VEH_III_aged_12_dpi_no_reconex_cjun_19.lif_Series001/VEH_III_aged_12_dpi_no_reconex_cjun_19.lif_Series001_z03_ch00.tif]

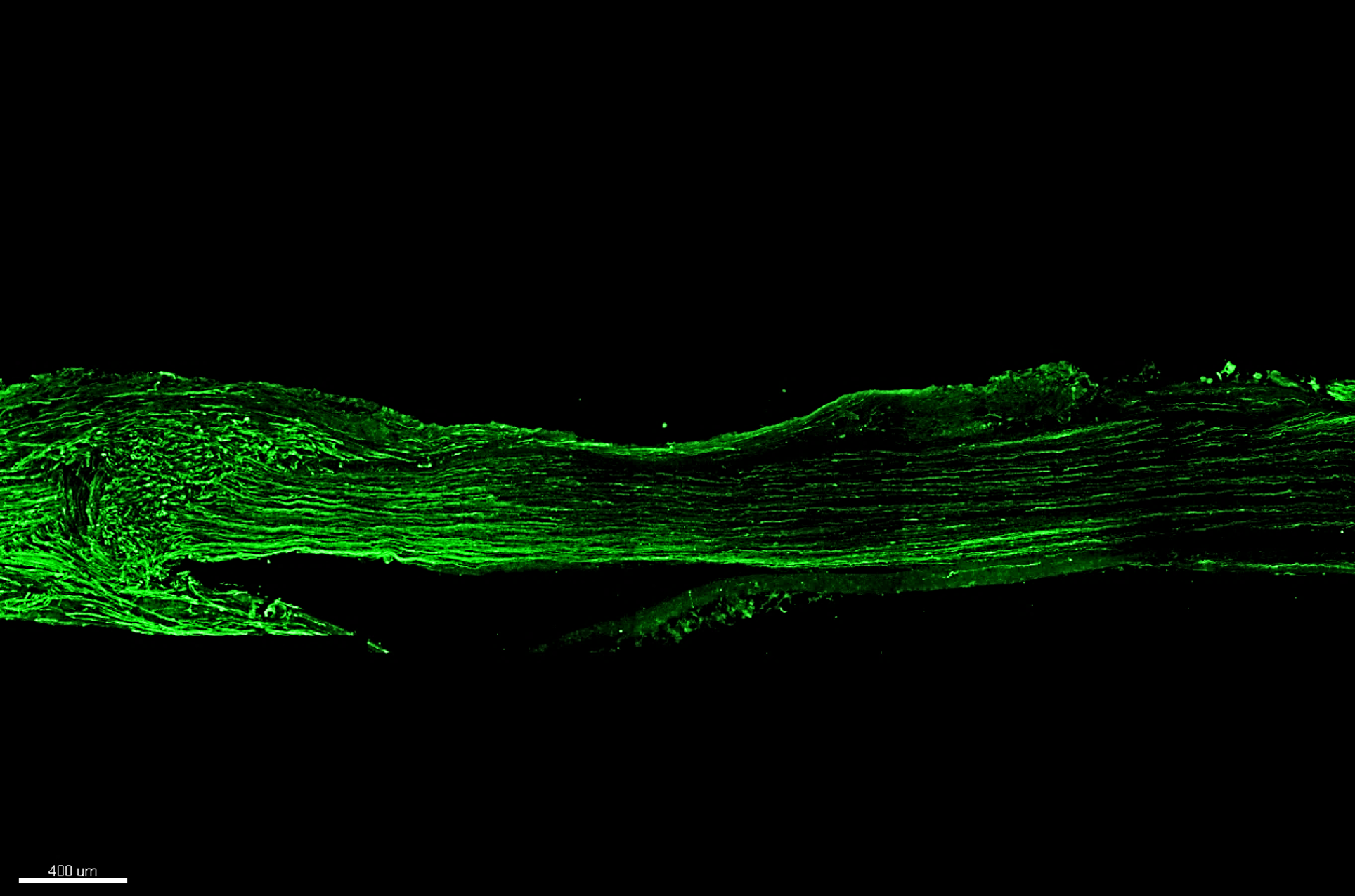

Supplement: Supplementary file 15 — Source Data for Figure 5 [file EMMM-15-e17907-s008.zip › SourceData_Fig_5/Fig_5_SourceData_images/3B/AGED_ABT.tif]

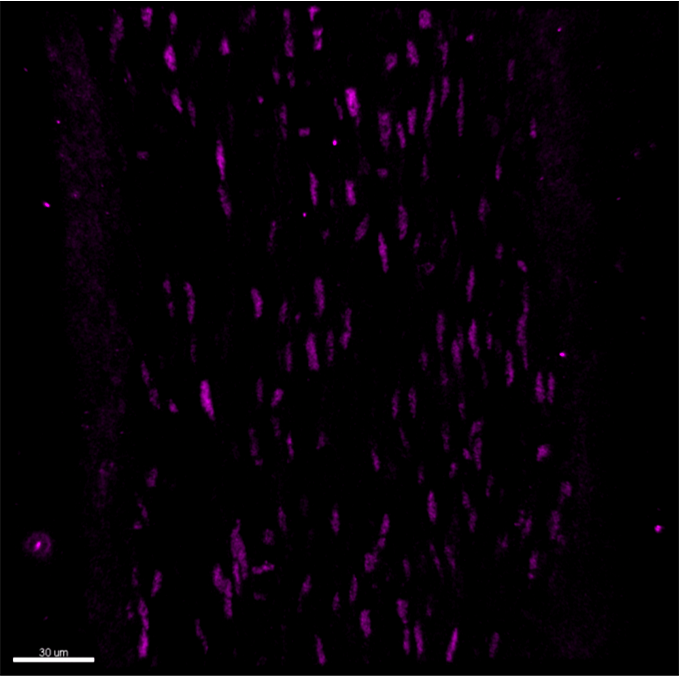

Supplement: Supplementary file 15 — Source Data for Figure 5 [file EMMM-15-e17907-s008.zip › SourceData_Fig_5/Fig_5_SourceData_images/3G/GCV_SOX.tif]

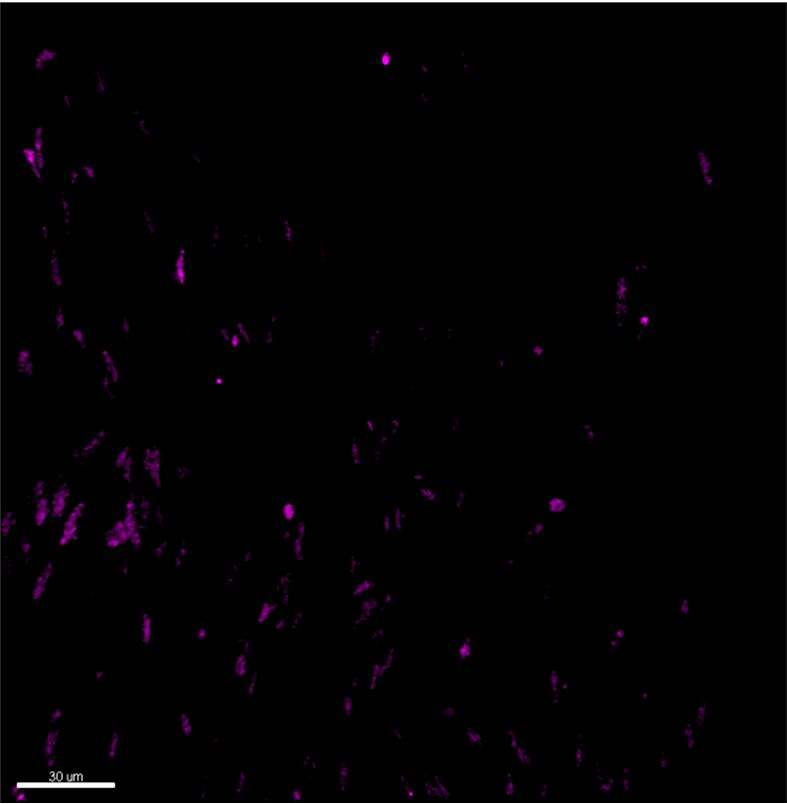

Supplement: Supplementary file 15 — Source Data for Figure 5 [file EMMM-15-e17907-s008.zip › SourceData_Fig_5/Fig_5_SourceData_images/3G/VEHICLE_SOX.tif]

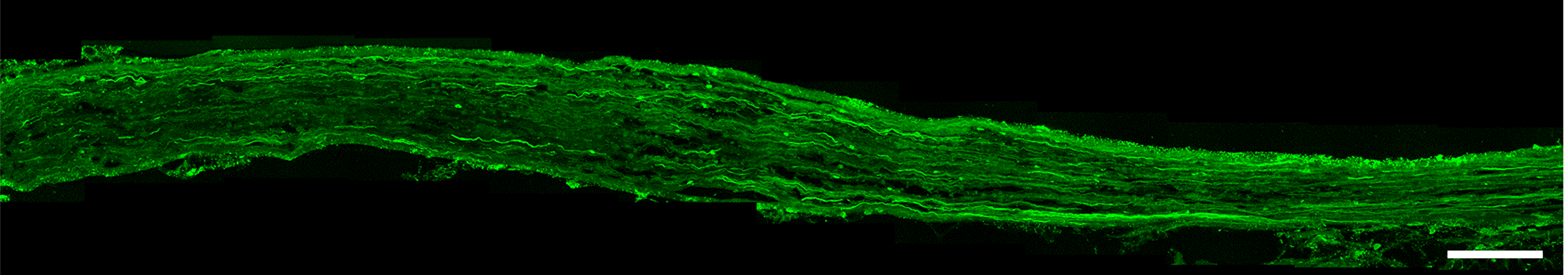

Supplement: Supplementary file 15 — Source Data for Figure 5 [file EMMM-15-e17907-s008.zip › SourceData_Fig_5/Fig_5_SourceData_images/3I/GCV.tif]

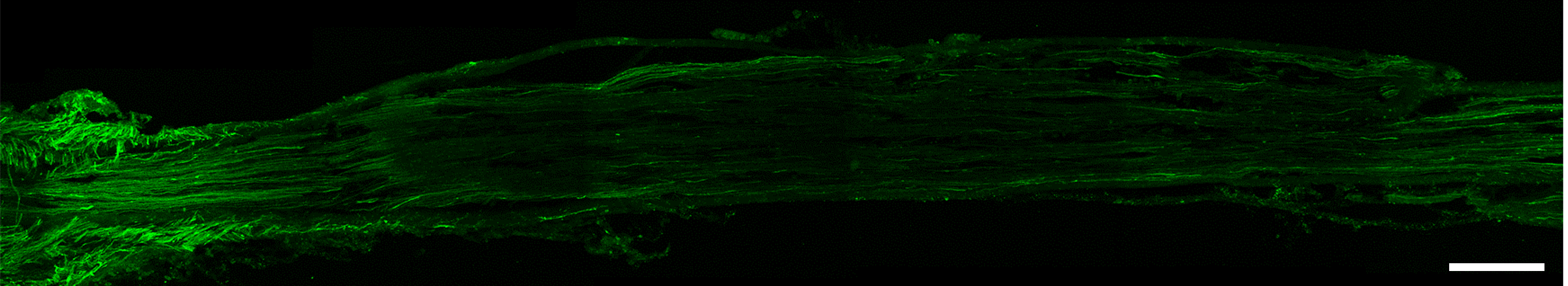

Supplement: Supplementary file 15 — Source Data for Figure 5 [file EMMM-15-e17907-s008.zip › SourceData_Fig_5/Fig_5_SourceData_images/3I/VEH.tif]
