# Supplementary figures and images for: Senescent Schwann cells induced by aging and chronic denervation impair axonal regeneration following peripheral nerve injury (part 2 of 3)
Source: EMBO Mol Med. 2023 Oct 20;15(12):e17907. doi: 10.15252/emmm.202317907 (PMC10701627; doi:10.15252/emmm.202317907)

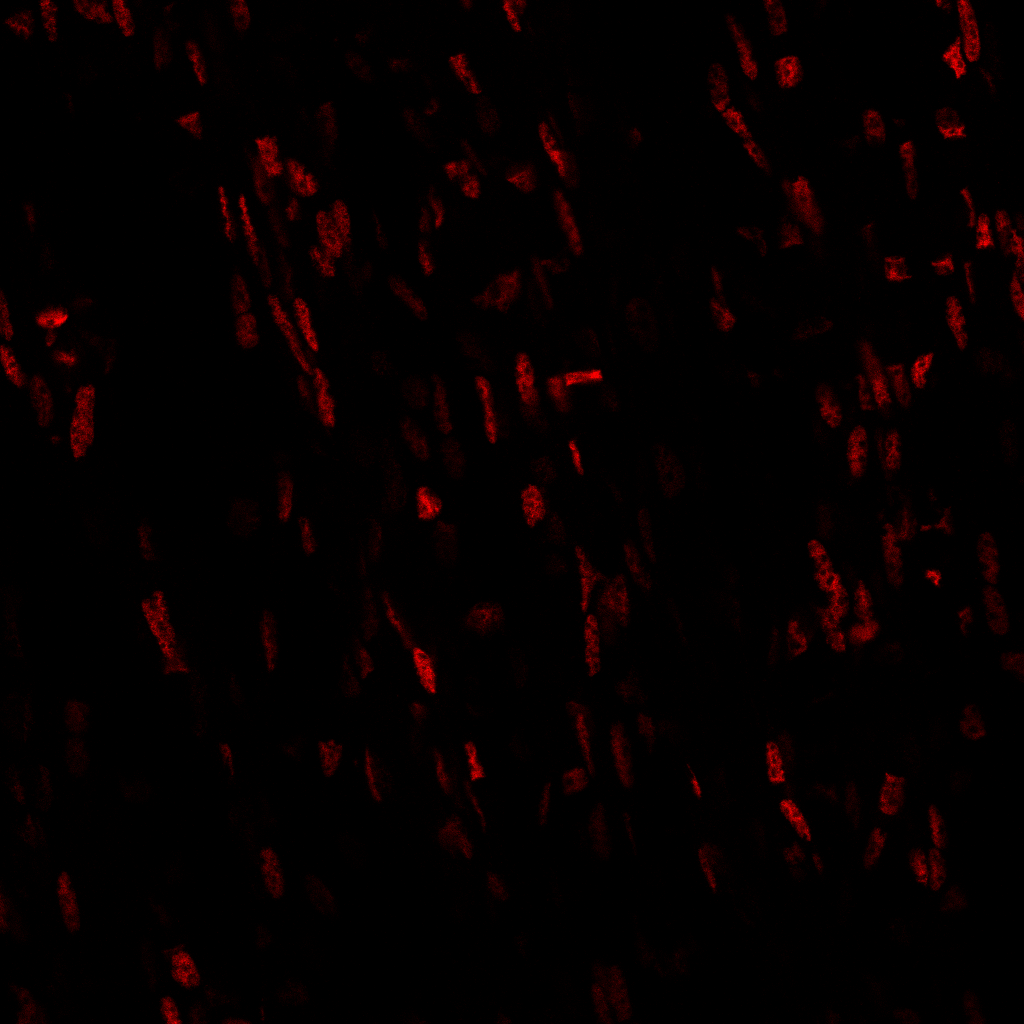

Supplement: Supplementary file 14 — Source Data for Figure 4 [file EMMM-15-e17907-s014.zip › SourceData_Fig_4/Fig_4_SourceData_images/3H/ABT_3_adult_42_dpi_no_reconex_cjun_19.lif_Series003/ABT_3_adult_42_dpi_no_reconex_cjun_19.lif_Series003_z05_ch03.tif]

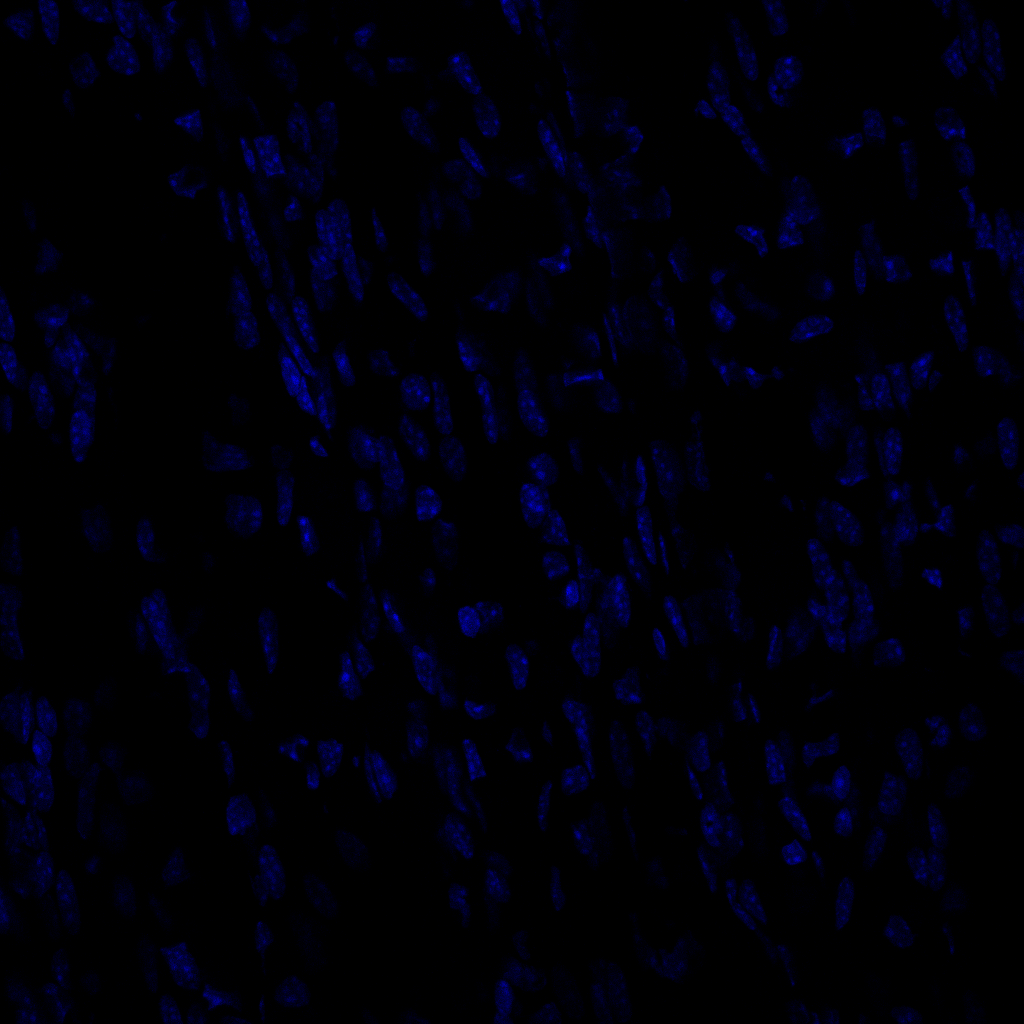

Supplement: Supplementary file 14 — Source Data for Figure 4 [file EMMM-15-e17907-s014.zip › SourceData_Fig_4/Fig_4_SourceData_images/3H/ABT_3_adult_42_dpi_no_reconex_cjun_19.lif_Series003/ABT_3_adult_42_dpi_no_reconex_cjun_19.lif_Series003_z06_ch00.tif]

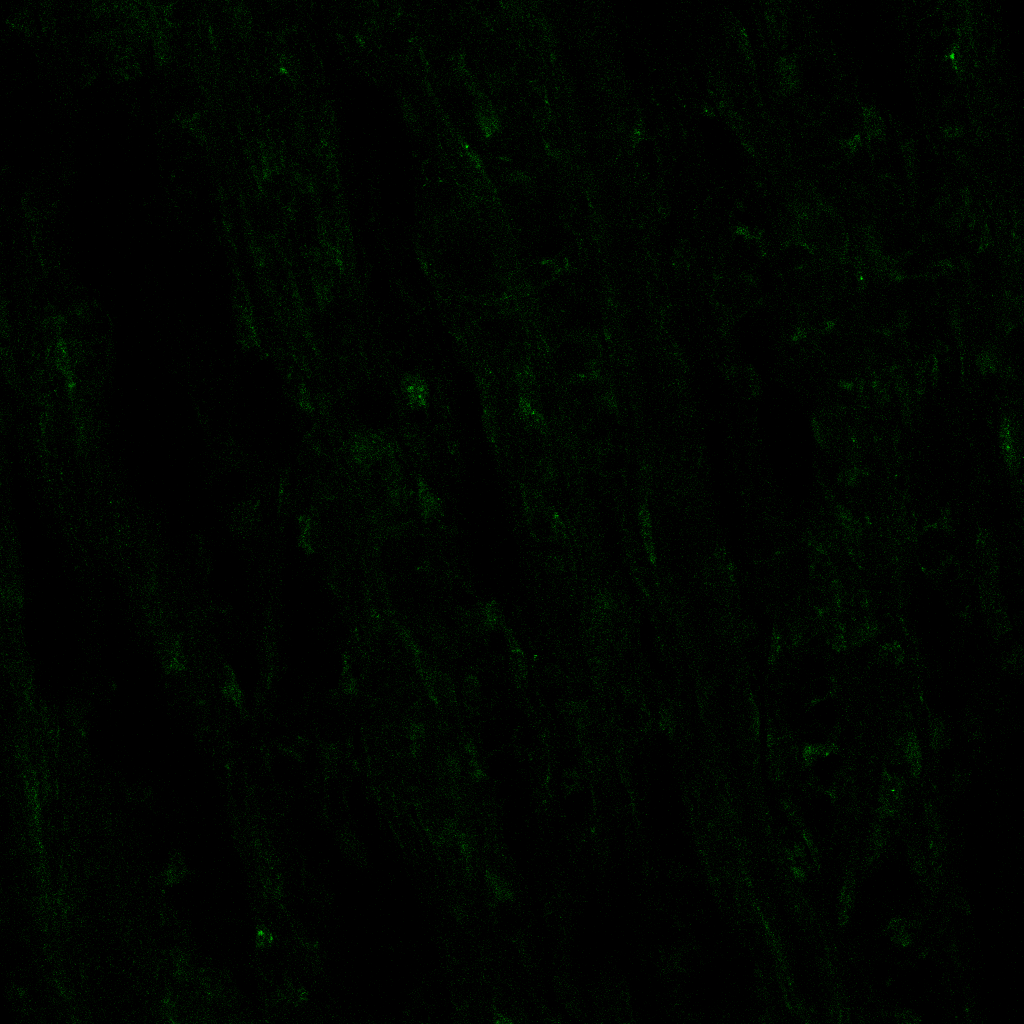

Supplement: Supplementary file 14 — Source Data for Figure 4 [file EMMM-15-e17907-s014.zip › SourceData_Fig_4/Fig_4_SourceData_images/3H/ABT_3_adult_42_dpi_no_reconex_cjun_19.lif_Series003/ABT_3_adult_42_dpi_no_reconex_cjun_19.lif_Series003_z06_ch01.tif]

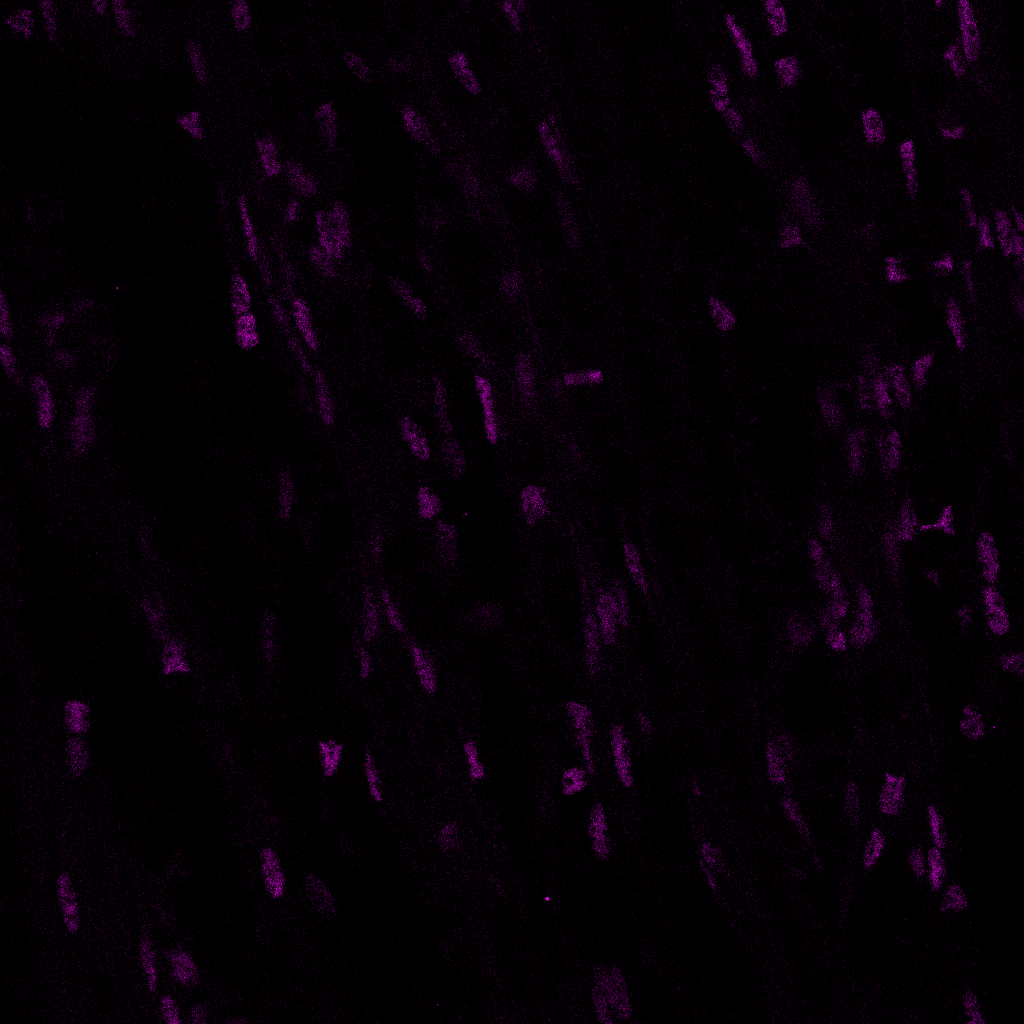

Supplement: Supplementary file 14 — Source Data for Figure 4 [file EMMM-15-e17907-s014.zip › SourceData_Fig_4/Fig_4_SourceData_images/3H/ABT_3_adult_42_dpi_no_reconex_cjun_19.lif_Series003/ABT_3_adult_42_dpi_no_reconex_cjun_19.lif_Series003_z06_ch02.tif]

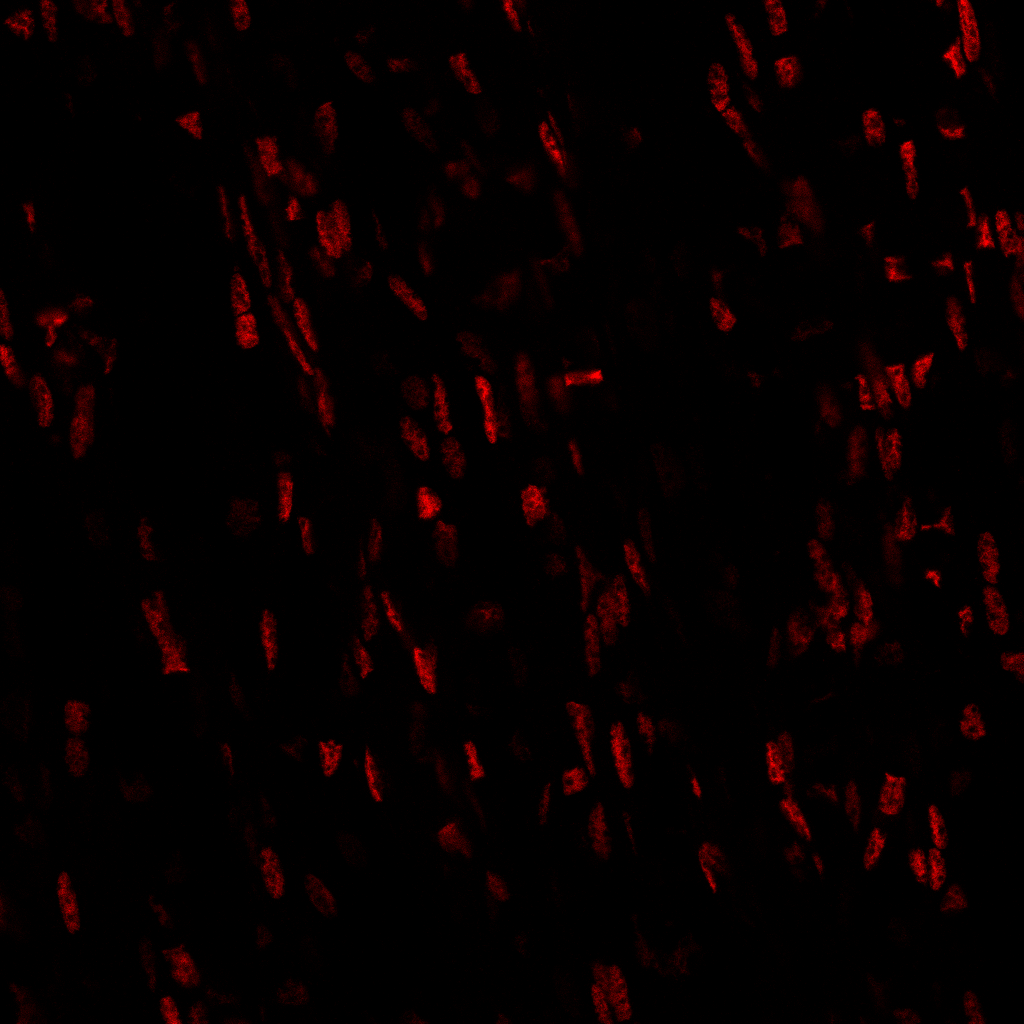

Supplement: Supplementary file 14 — Source Data for Figure 4 [file EMMM-15-e17907-s014.zip › SourceData_Fig_4/Fig_4_SourceData_images/3H/ABT_3_adult_42_dpi_no_reconex_cjun_19.lif_Series003/ABT_3_adult_42_dpi_no_reconex_cjun_19.lif_Series003_z06_ch03.tif]

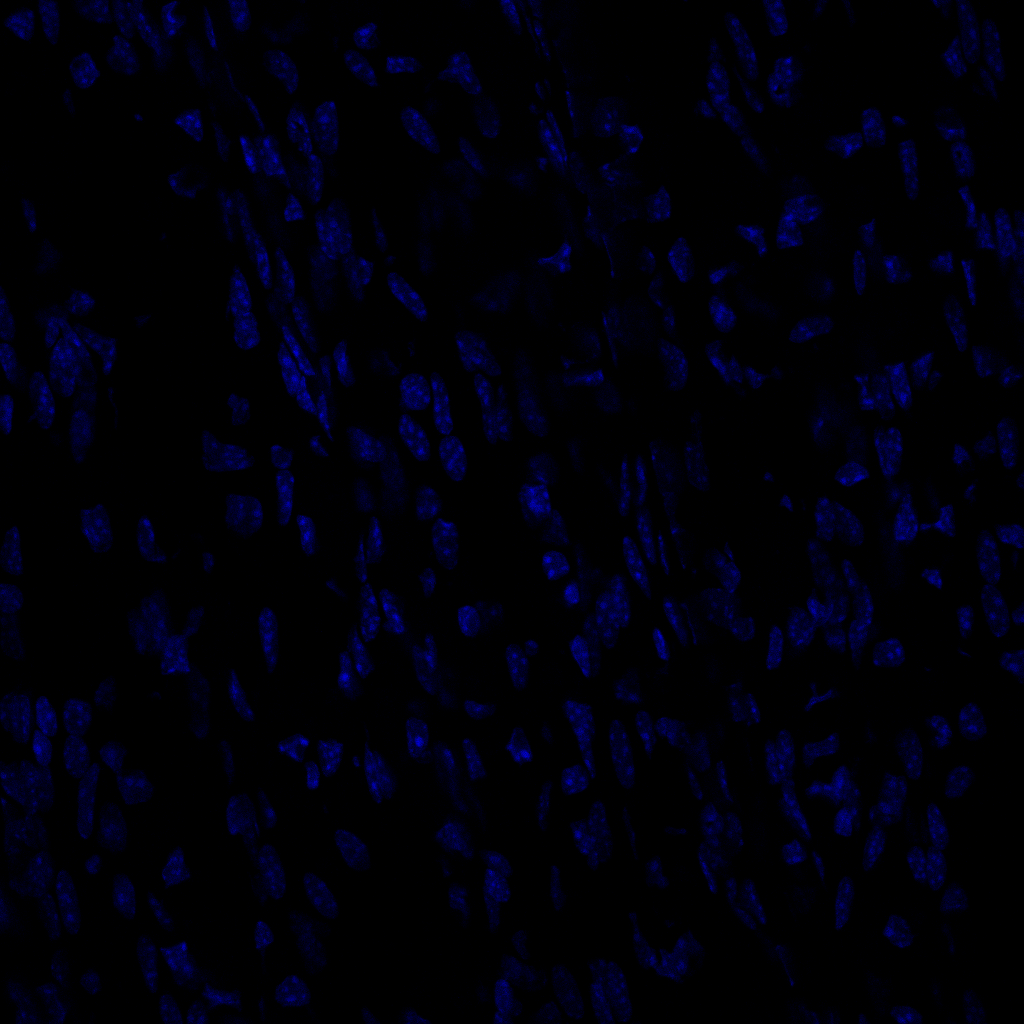

Supplement: Supplementary file 14 — Source Data for Figure 4 [file EMMM-15-e17907-s014.zip › SourceData_Fig_4/Fig_4_SourceData_images/3H/ABT_3_adult_42_dpi_no_reconex_cjun_19.lif_Series003/ABT_3_adult_42_dpi_no_reconex_cjun_19.lif_Series003_z07_ch00.tif]

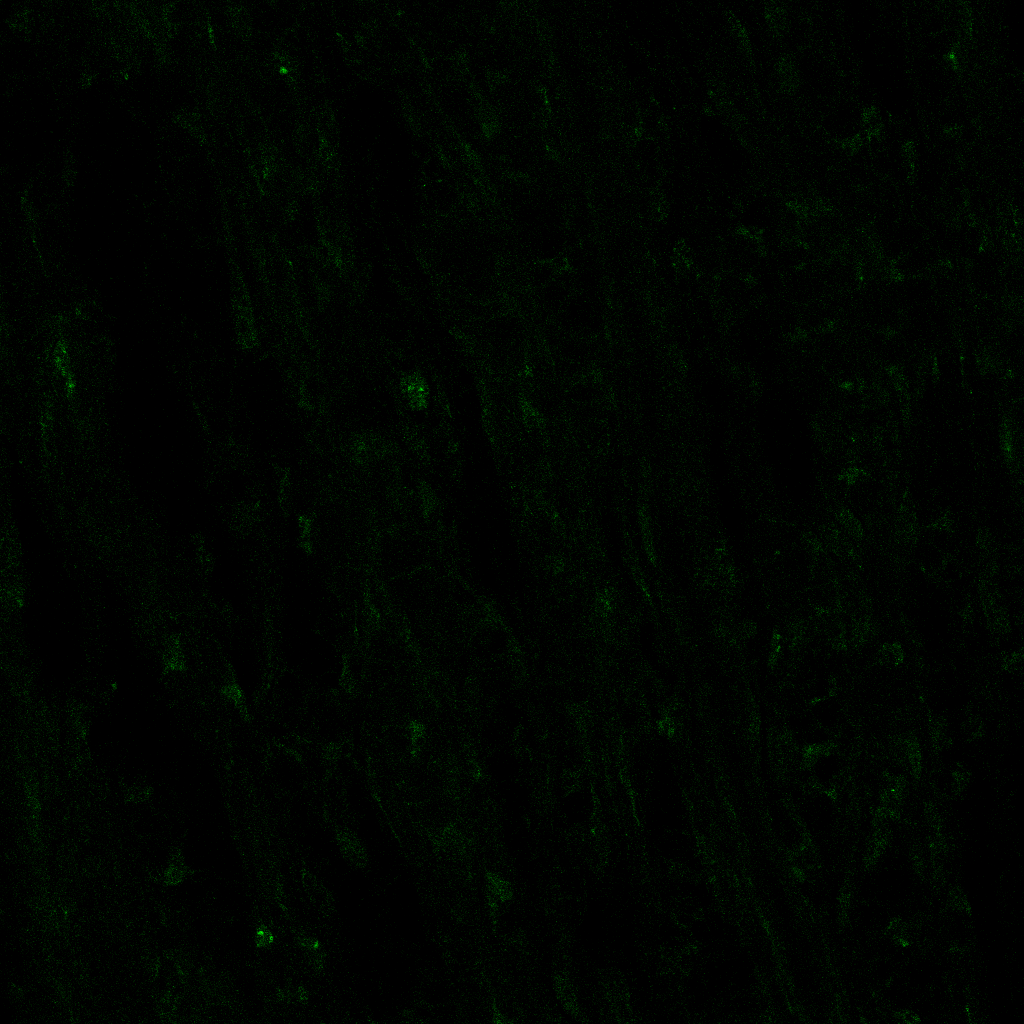

Supplement: Supplementary file 14 — Source Data for Figure 4 [file EMMM-15-e17907-s014.zip › SourceData_Fig_4/Fig_4_SourceData_images/3H/ABT_3_adult_42_dpi_no_reconex_cjun_19.lif_Series003/ABT_3_adult_42_dpi_no_reconex_cjun_19.lif_Series003_z07_ch01.tif]

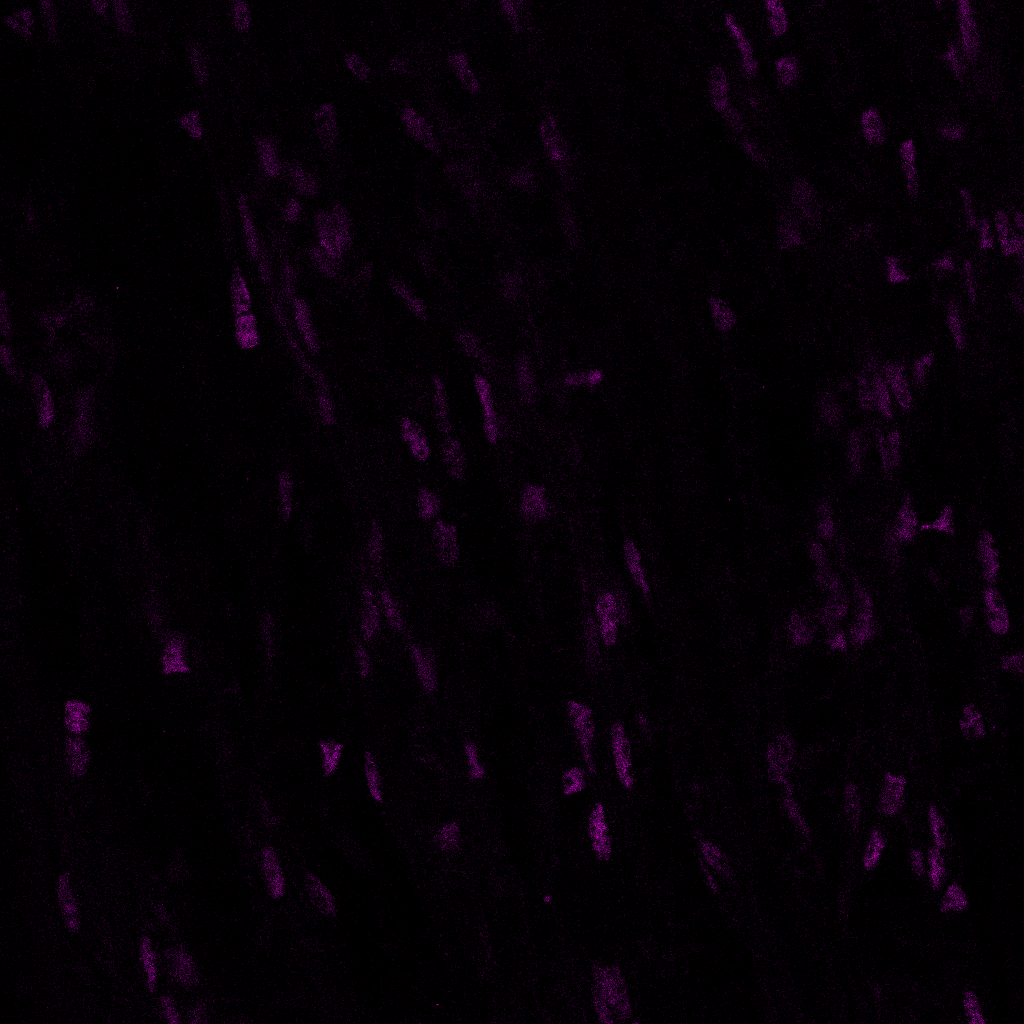

Supplement: Supplementary file 14 — Source Data for Figure 4 [file EMMM-15-e17907-s014.zip › SourceData_Fig_4/Fig_4_SourceData_images/3H/ABT_3_adult_42_dpi_no_reconex_cjun_19.lif_Series003/ABT_3_adult_42_dpi_no_reconex_cjun_19.lif_Series003_z07_ch02.tif]

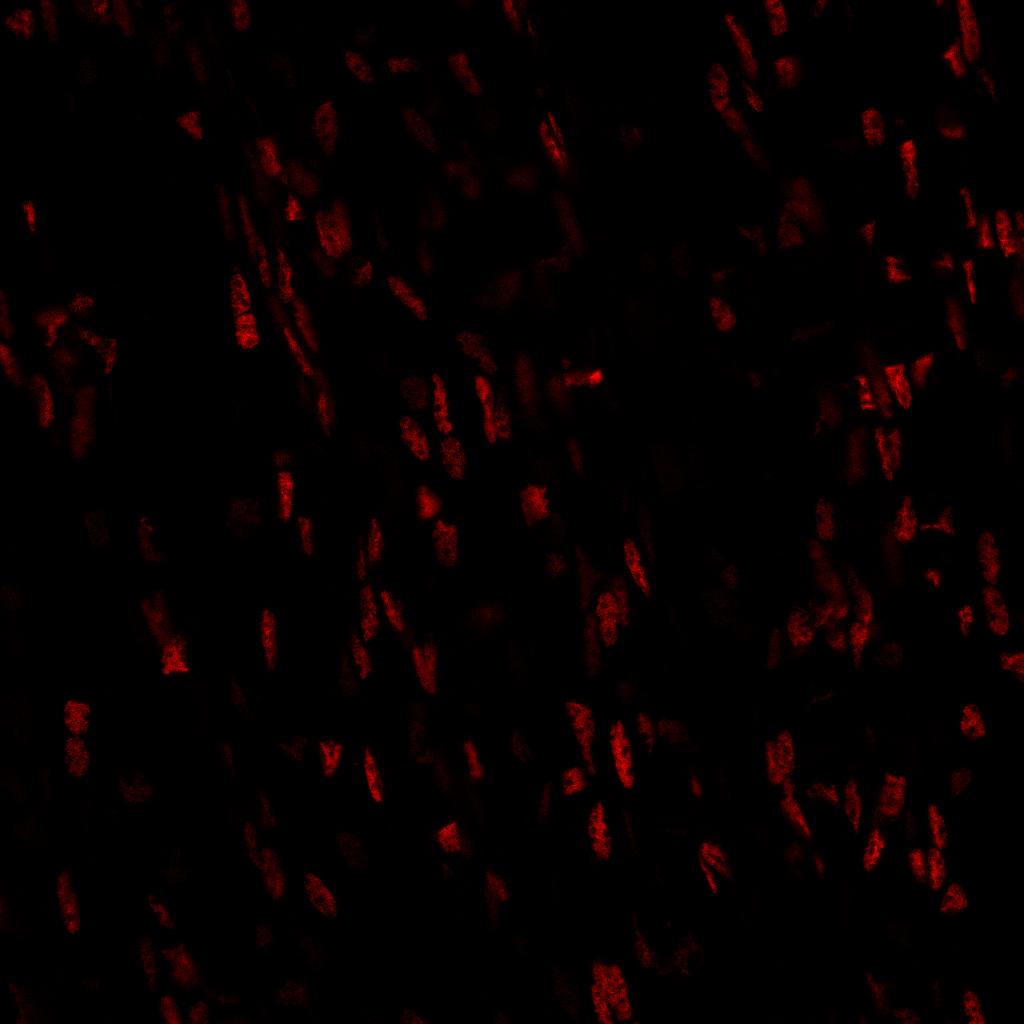

Supplement: Supplementary file 14 — Source Data for Figure 4 [file EMMM-15-e17907-s014.zip › SourceData_Fig_4/Fig_4_SourceData_images/3H/ABT_3_adult_42_dpi_no_reconex_cjun_19.lif_Series003/ABT_3_adult_42_dpi_no_reconex_cjun_19.lif_Series003_z07_ch03.tif]

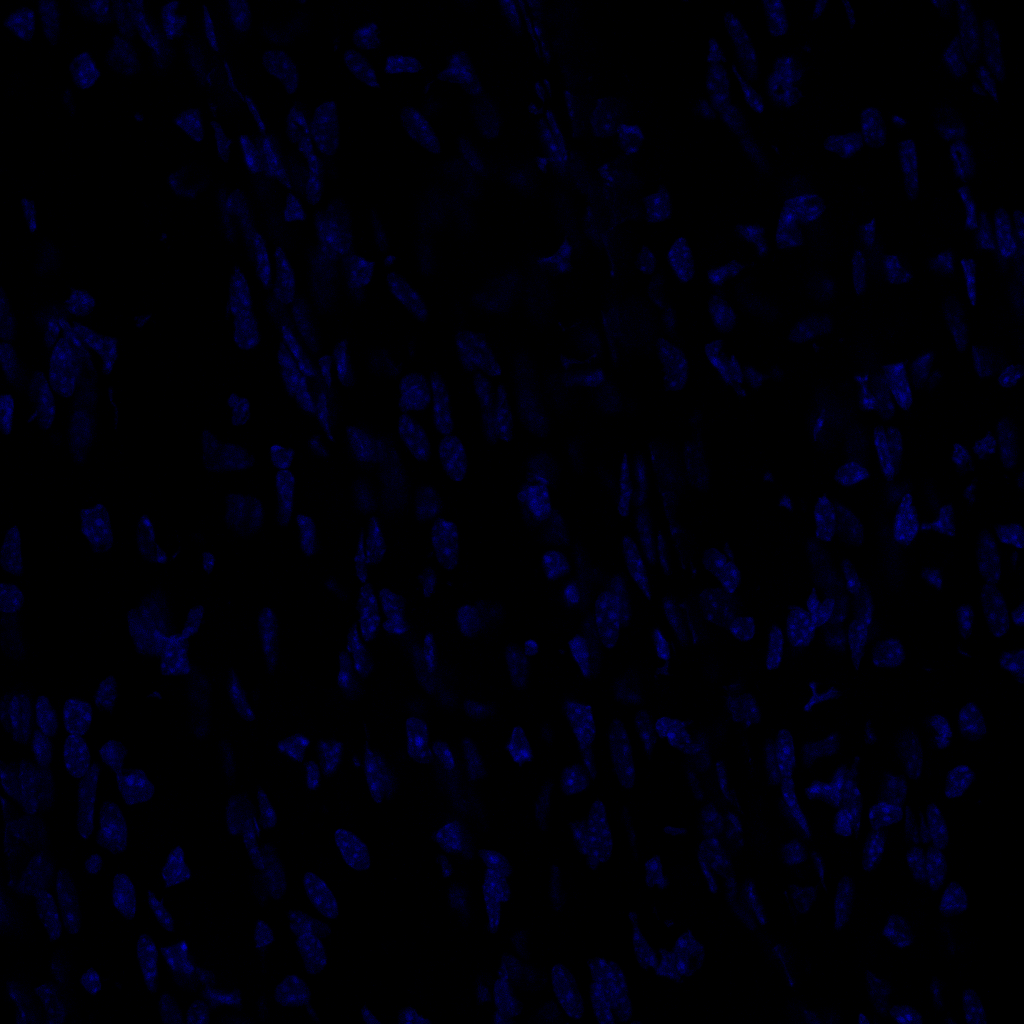

Supplement: Supplementary file 14 — Source Data for Figure 4 [file EMMM-15-e17907-s014.zip › SourceData_Fig_4/Fig_4_SourceData_images/3H/ABT_3_adult_42_dpi_no_reconex_cjun_19.lif_Series003/ABT_3_adult_42_dpi_no_reconex_cjun_19.lif_Series003_z08_ch00.tif]

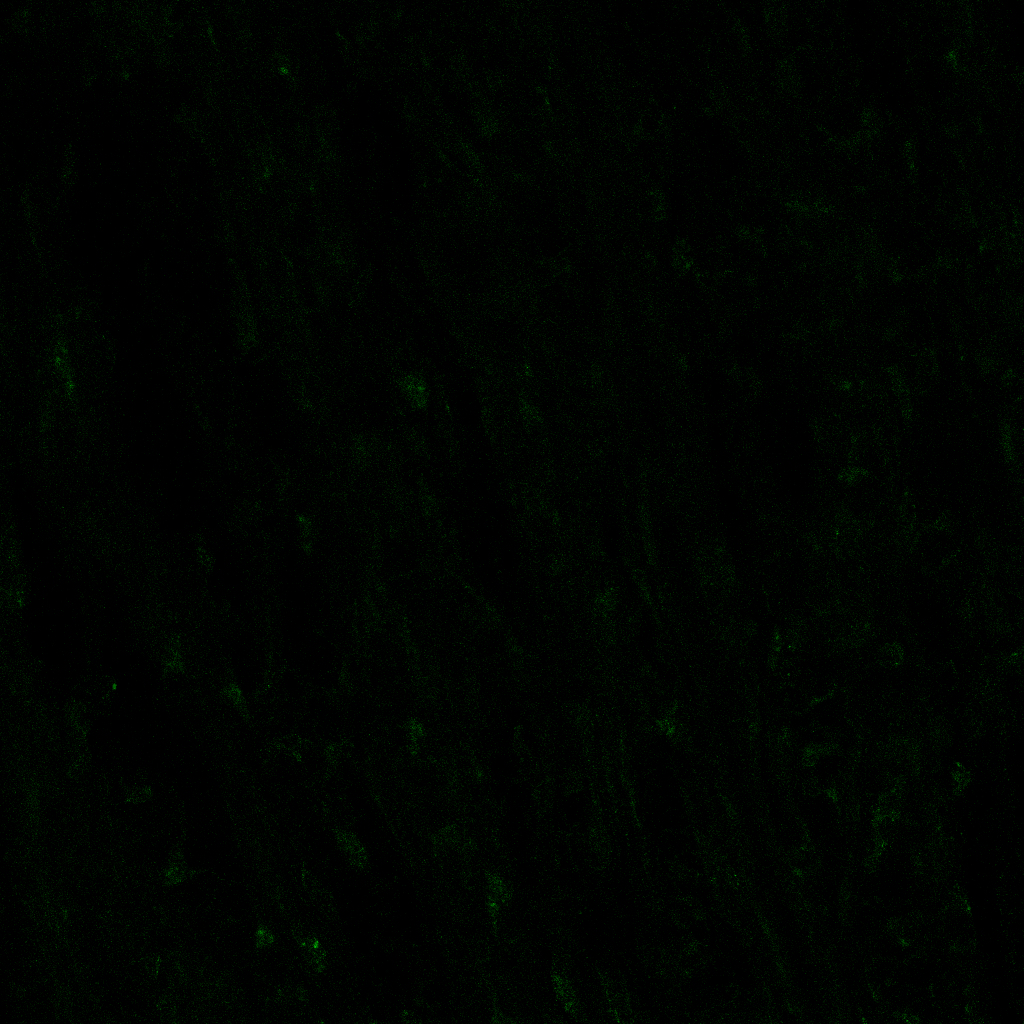

Supplement: Supplementary file 14 — Source Data for Figure 4 [file EMMM-15-e17907-s014.zip › SourceData_Fig_4/Fig_4_SourceData_images/3H/ABT_3_adult_42_dpi_no_reconex_cjun_19.lif_Series003/ABT_3_adult_42_dpi_no_reconex_cjun_19.lif_Series003_z08_ch01.tif]

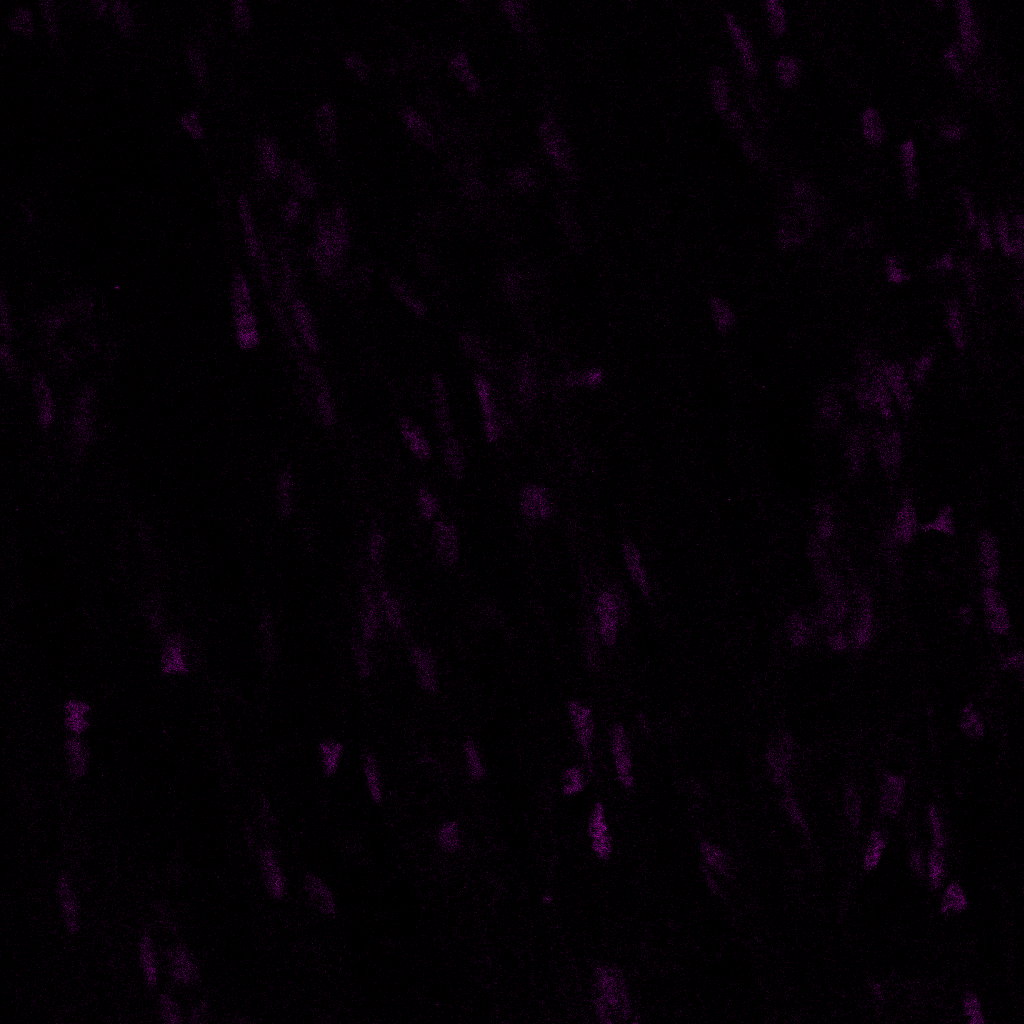

Supplement: Supplementary file 14 — Source Data for Figure 4 [file EMMM-15-e17907-s014.zip › SourceData_Fig_4/Fig_4_SourceData_images/3H/ABT_3_adult_42_dpi_no_reconex_cjun_19.lif_Series003/ABT_3_adult_42_dpi_no_reconex_cjun_19.lif_Series003_z08_ch02.tif]

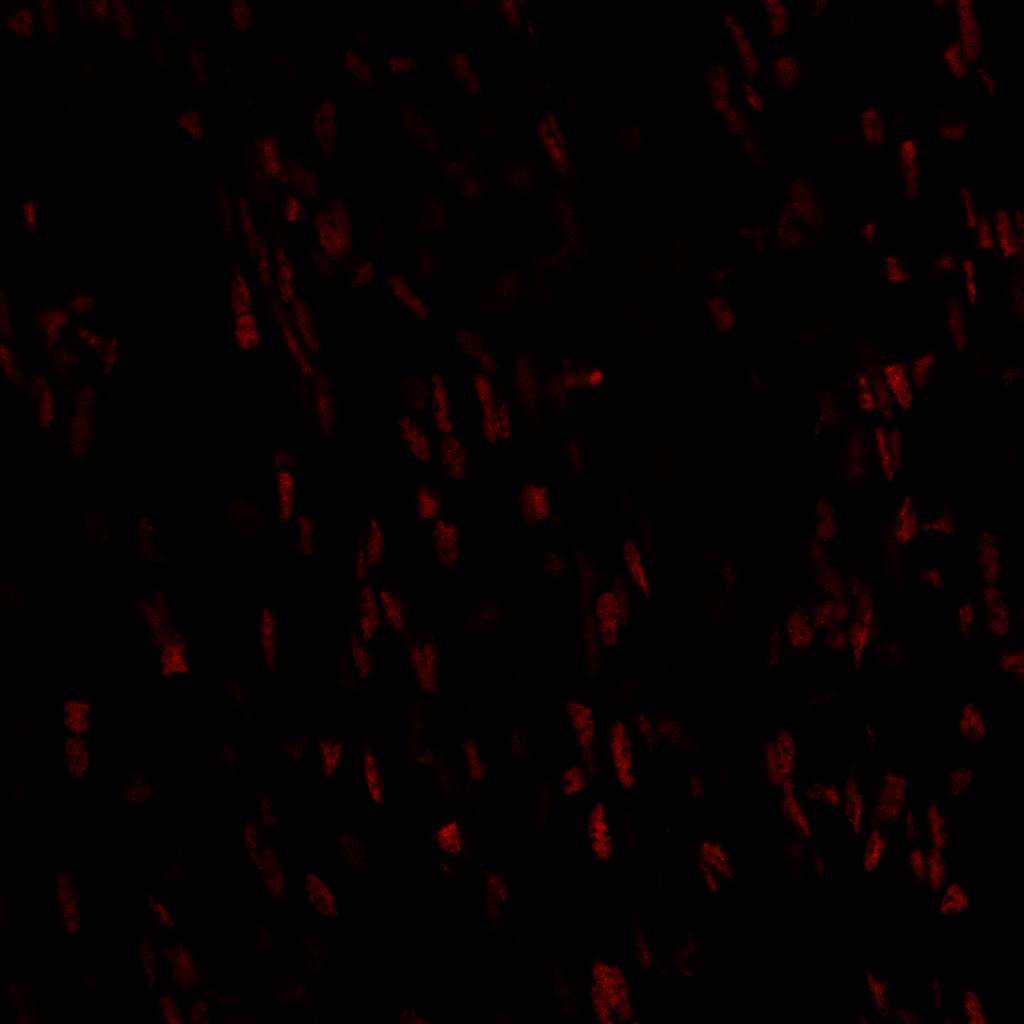

Supplement: Supplementary file 14 — Source Data for Figure 4 [file EMMM-15-e17907-s014.zip › SourceData_Fig_4/Fig_4_SourceData_images/3H/ABT_3_adult_42_dpi_no_reconex_cjun_19.lif_Series003/ABT_3_adult_42_dpi_no_reconex_cjun_19.lif_Series003_z08_ch03.tif]

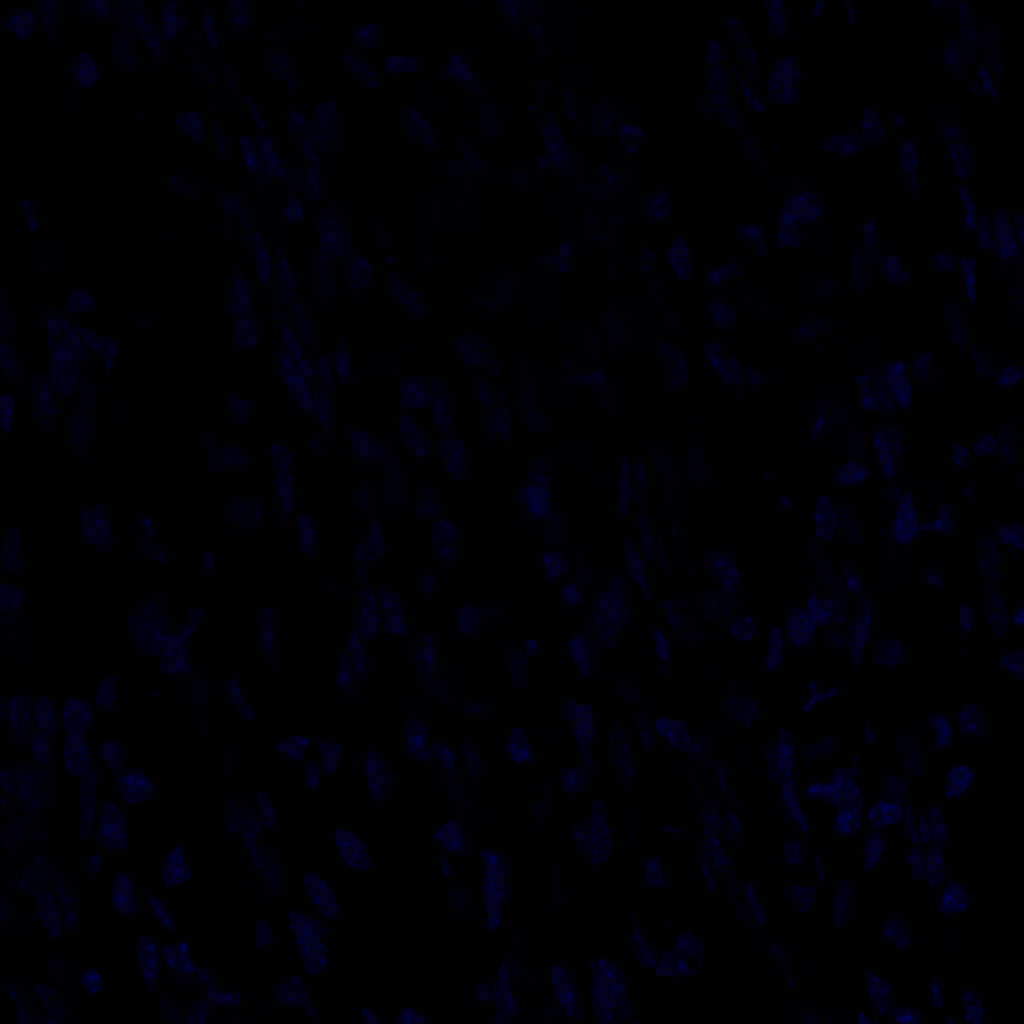

Supplement: Supplementary file 14 — Source Data for Figure 4 [file EMMM-15-e17907-s014.zip › SourceData_Fig_4/Fig_4_SourceData_images/3H/ABT_3_adult_42_dpi_no_reconex_cjun_19.lif_Series003/ABT_3_adult_42_dpi_no_reconex_cjun_19.lif_Series003_z09_ch00.tif]

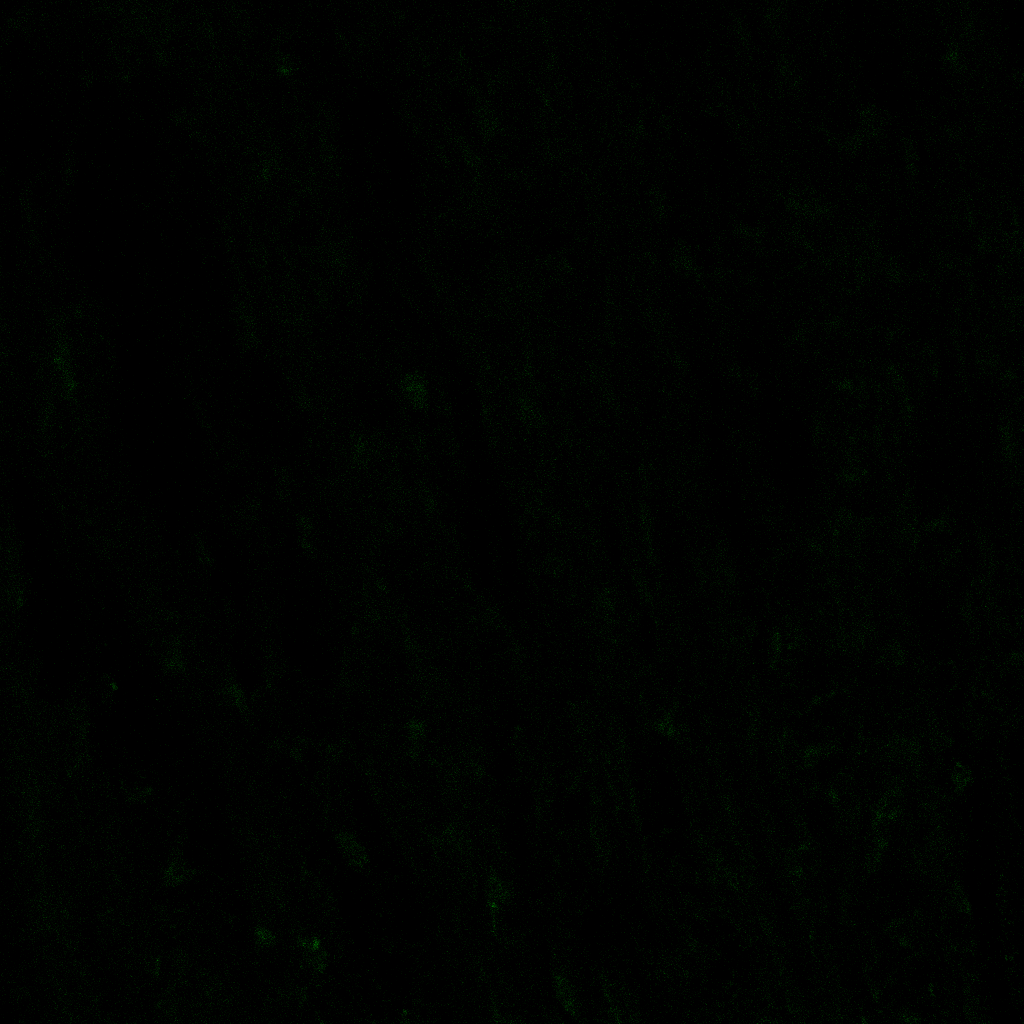

Supplement: Supplementary file 14 — Source Data for Figure 4 [file EMMM-15-e17907-s014.zip › SourceData_Fig_4/Fig_4_SourceData_images/3H/ABT_3_adult_42_dpi_no_reconex_cjun_19.lif_Series003/ABT_3_adult_42_dpi_no_reconex_cjun_19.lif_Series003_z09_ch01.tif]

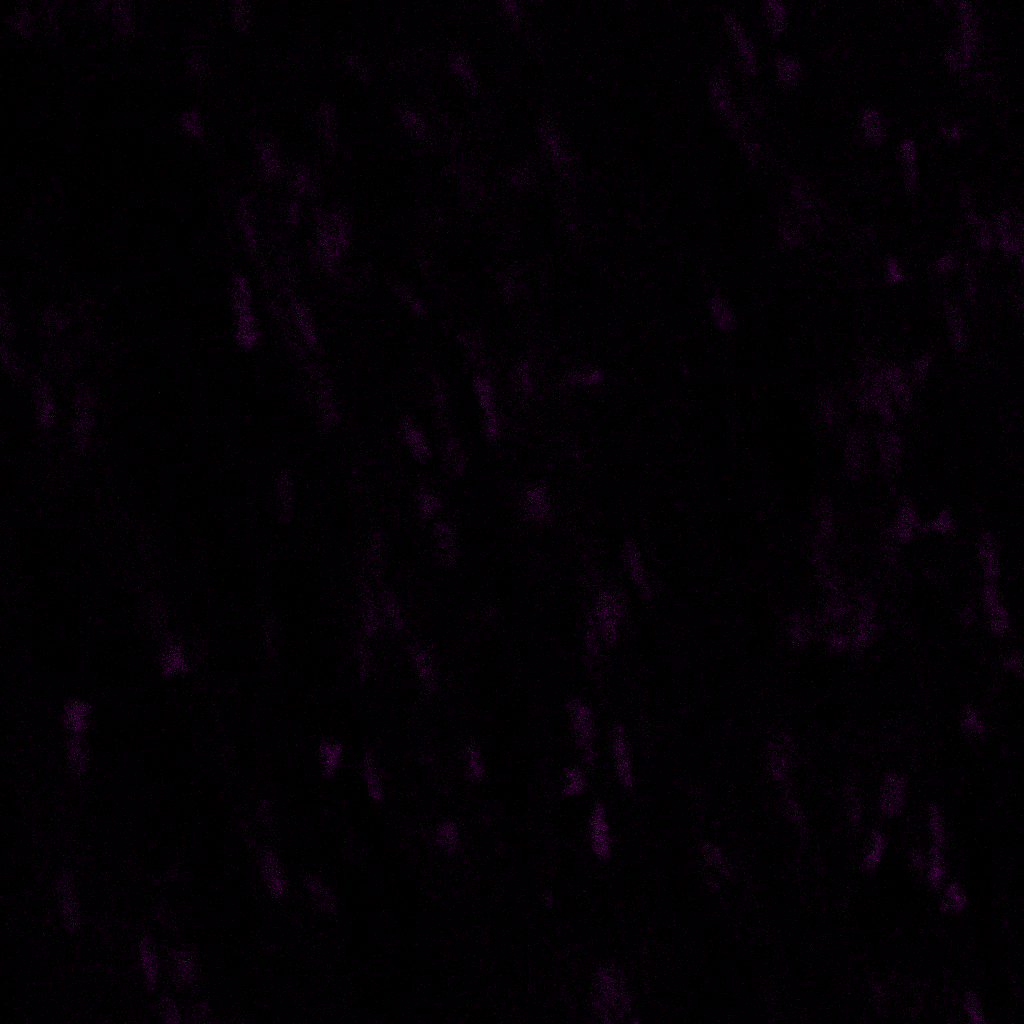

Supplement: Supplementary file 14 — Source Data for Figure 4 [file EMMM-15-e17907-s014.zip › SourceData_Fig_4/Fig_4_SourceData_images/3H/ABT_3_adult_42_dpi_no_reconex_cjun_19.lif_Series003/ABT_3_adult_42_dpi_no_reconex_cjun_19.lif_Series003_z09_ch02.tif]

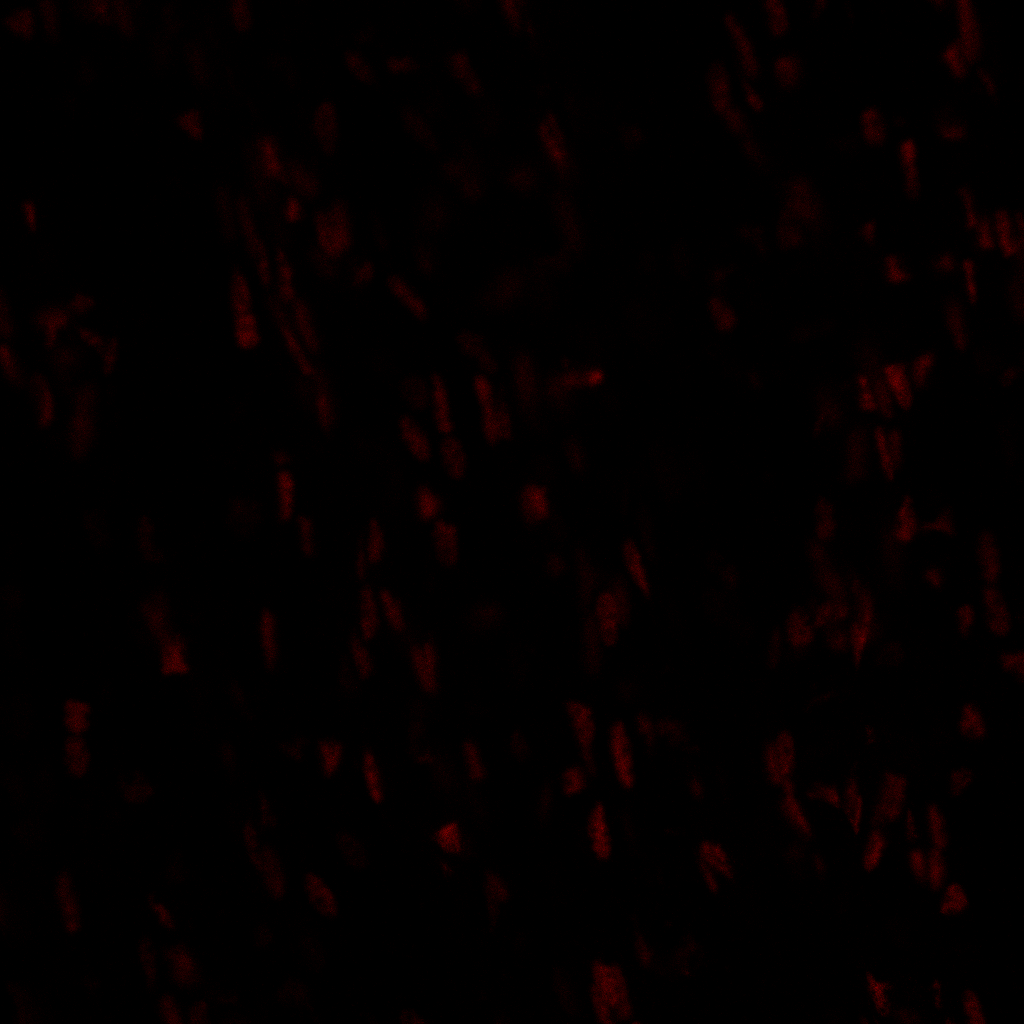

Supplement: Supplementary file 14 — Source Data for Figure 4 [file EMMM-15-e17907-s014.zip › SourceData_Fig_4/Fig_4_SourceData_images/3H/ABT_3_adult_42_dpi_no_reconex_cjun_19.lif_Series003/ABT_3_adult_42_dpi_no_reconex_cjun_19.lif_Series003_z09_ch03.tif]

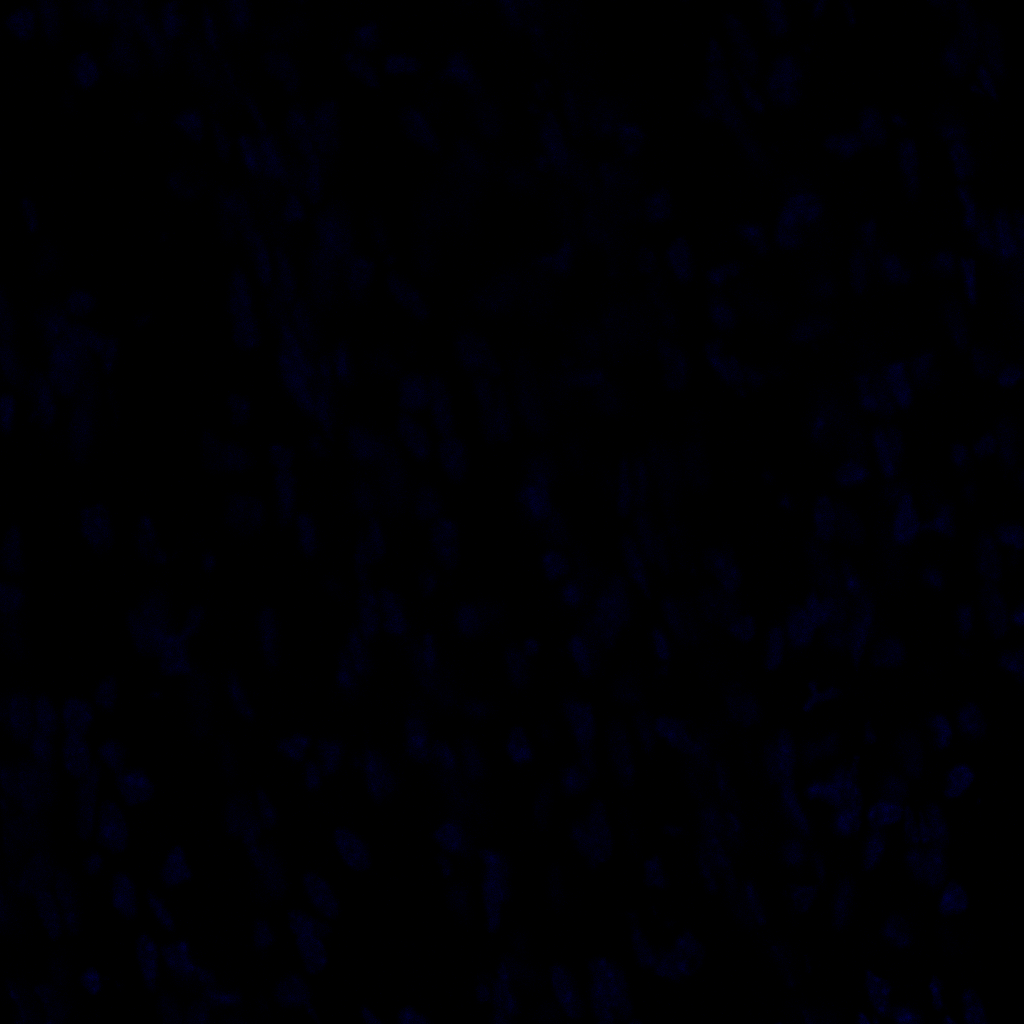

Supplement: Supplementary file 14 — Source Data for Figure 4 [file EMMM-15-e17907-s014.zip › SourceData_Fig_4/Fig_4_SourceData_images/3H/ABT_3_adult_42_dpi_no_reconex_cjun_19.lif_Series003/ABT_3_adult_42_dpi_no_reconex_cjun_19.lif_Series003_z10_ch00.tif]

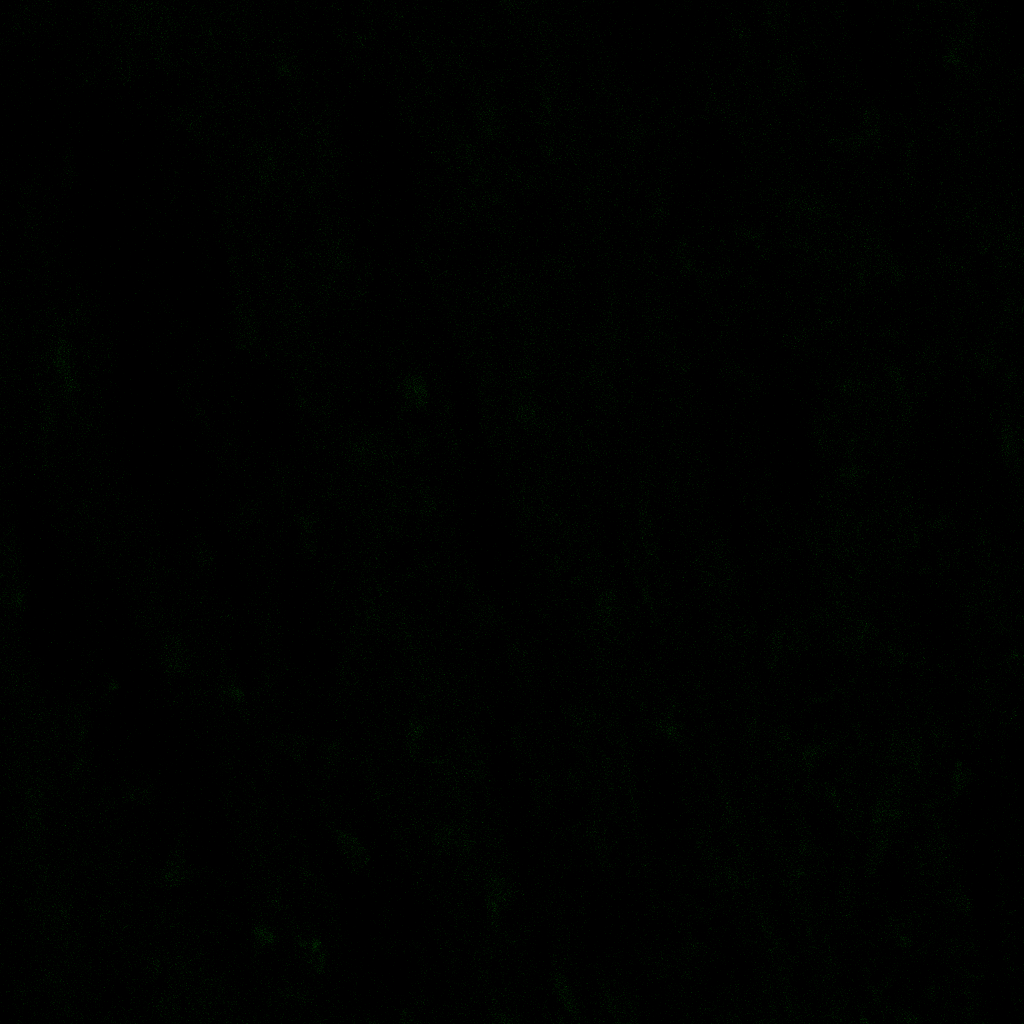

Supplement: Supplementary file 14 — Source Data for Figure 4 [file EMMM-15-e17907-s014.zip › SourceData_Fig_4/Fig_4_SourceData_images/3H/ABT_3_adult_42_dpi_no_reconex_cjun_19.lif_Series003/ABT_3_adult_42_dpi_no_reconex_cjun_19.lif_Series003_z10_ch01.tif]

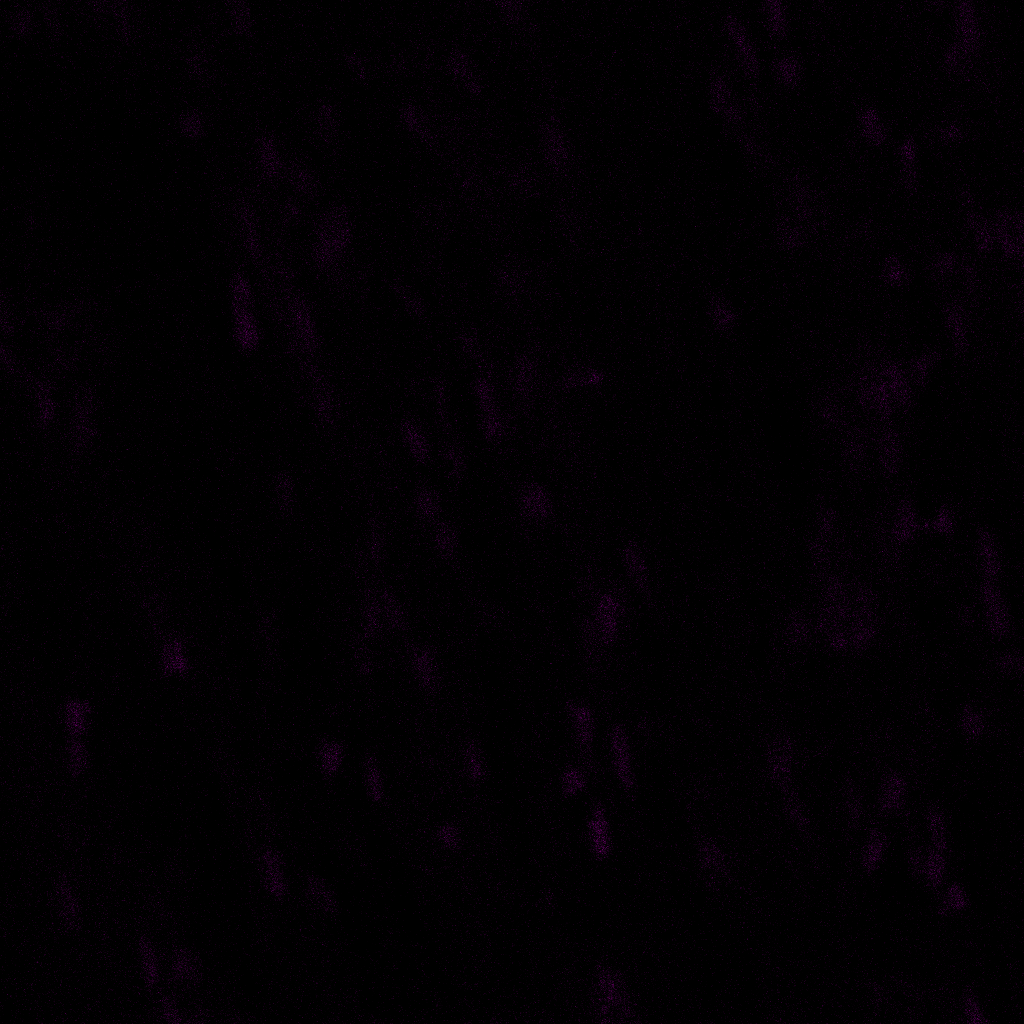

Supplement: Supplementary file 14 — Source Data for Figure 4 [file EMMM-15-e17907-s014.zip › SourceData_Fig_4/Fig_4_SourceData_images/3H/ABT_3_adult_42_dpi_no_reconex_cjun_19.lif_Series003/ABT_3_adult_42_dpi_no_reconex_cjun_19.lif_Series003_z10_ch02.tif]

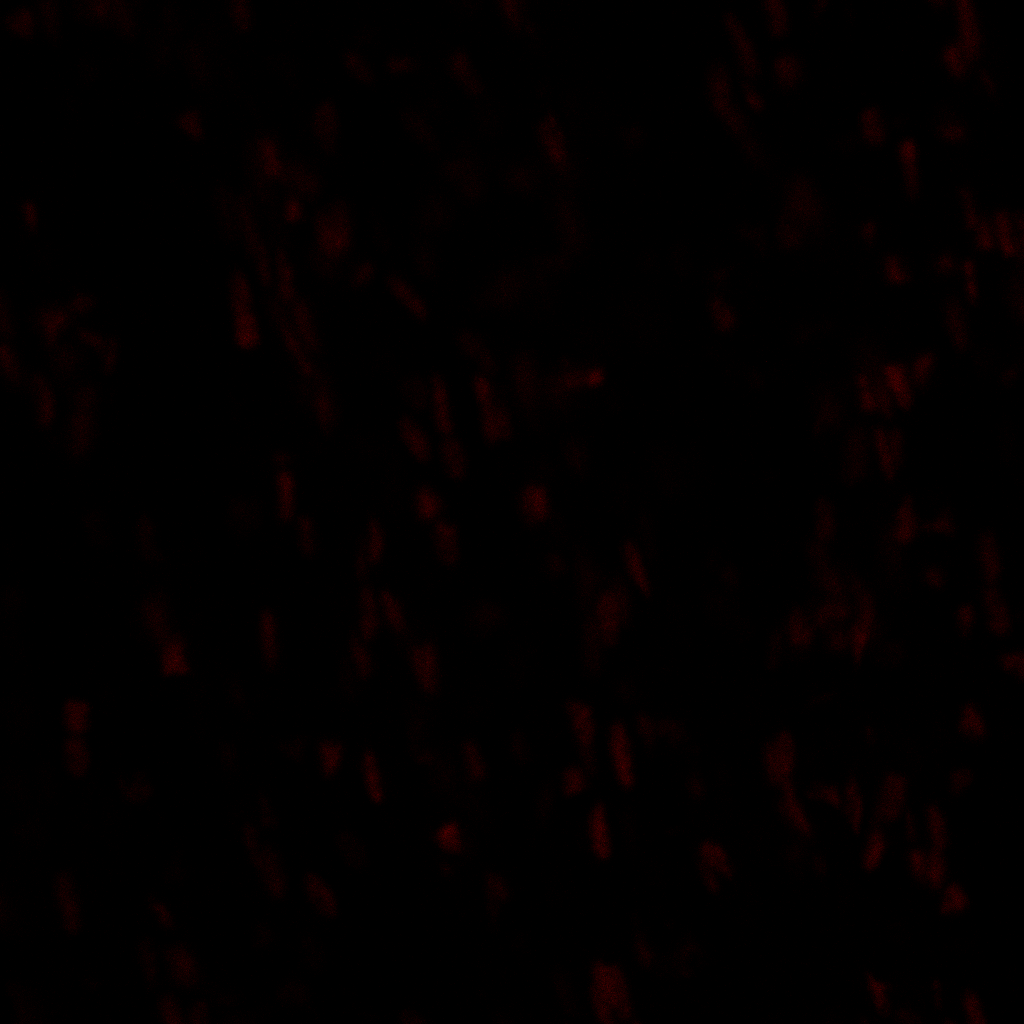

Supplement: Supplementary file 14 — Source Data for Figure 4 [file EMMM-15-e17907-s014.zip › SourceData_Fig_4/Fig_4_SourceData_images/3H/ABT_3_adult_42_dpi_no_reconex_cjun_19.lif_Series003/ABT_3_adult_42_dpi_no_reconex_cjun_19.lif_Series003_z10_ch03.tif]

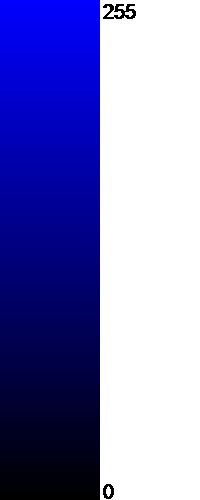

Supplement: Supplementary file 14 — Source Data for Figure 4 [file EMMM-15-e17907-s014.zip › SourceData_Fig_4/Fig_4_SourceData_images/3H/ABT_3_adult_42_dpi_no_reconex_cjun_19.lif_Series003/MetaData/ABT_3_adult_42_dpi_no_reconex_cjun_19.lif_Series003ch0LUT.png]

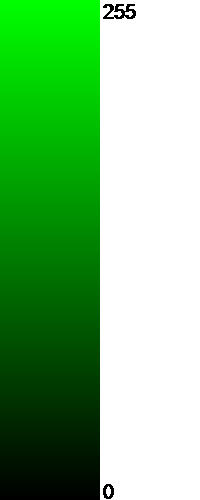

Supplement: Supplementary file 14 — Source Data for Figure 4 [file EMMM-15-e17907-s014.zip › SourceData_Fig_4/Fig_4_SourceData_images/3H/ABT_3_adult_42_dpi_no_reconex_cjun_19.lif_Series003/MetaData/ABT_3_adult_42_dpi_no_reconex_cjun_19.lif_Series003ch1LUT.png]

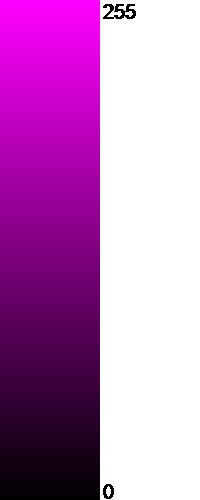

Supplement: Supplementary file 14 — Source Data for Figure 4 [file EMMM-15-e17907-s014.zip › SourceData_Fig_4/Fig_4_SourceData_images/3H/ABT_3_adult_42_dpi_no_reconex_cjun_19.lif_Series003/MetaData/ABT_3_adult_42_dpi_no_reconex_cjun_19.lif_Series003ch2LUT.png]

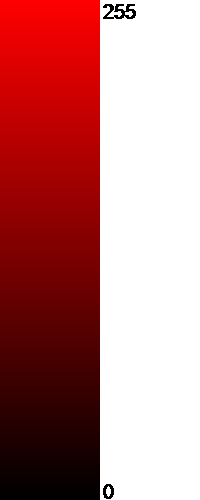

Supplement: Supplementary file 14 — Source Data for Figure 4 [file EMMM-15-e17907-s014.zip › SourceData_Fig_4/Fig_4_SourceData_images/3H/ABT_3_adult_42_dpi_no_reconex_cjun_19.lif_Series003/MetaData/ABT_3_adult_42_dpi_no_reconex_cjun_19.lif_Series003ch3LUT.png]

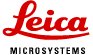

Supplement: Supplementary file 14 — Source Data for Figure 4 [file EMMM-15-e17907-s014.zip › SourceData_Fig_4/Fig_4_SourceData_images/3H/ABT_3_adult_42_dpi_no_reconex_cjun_19.lif_Series003/MetaData/LeicaLogo.jpg]

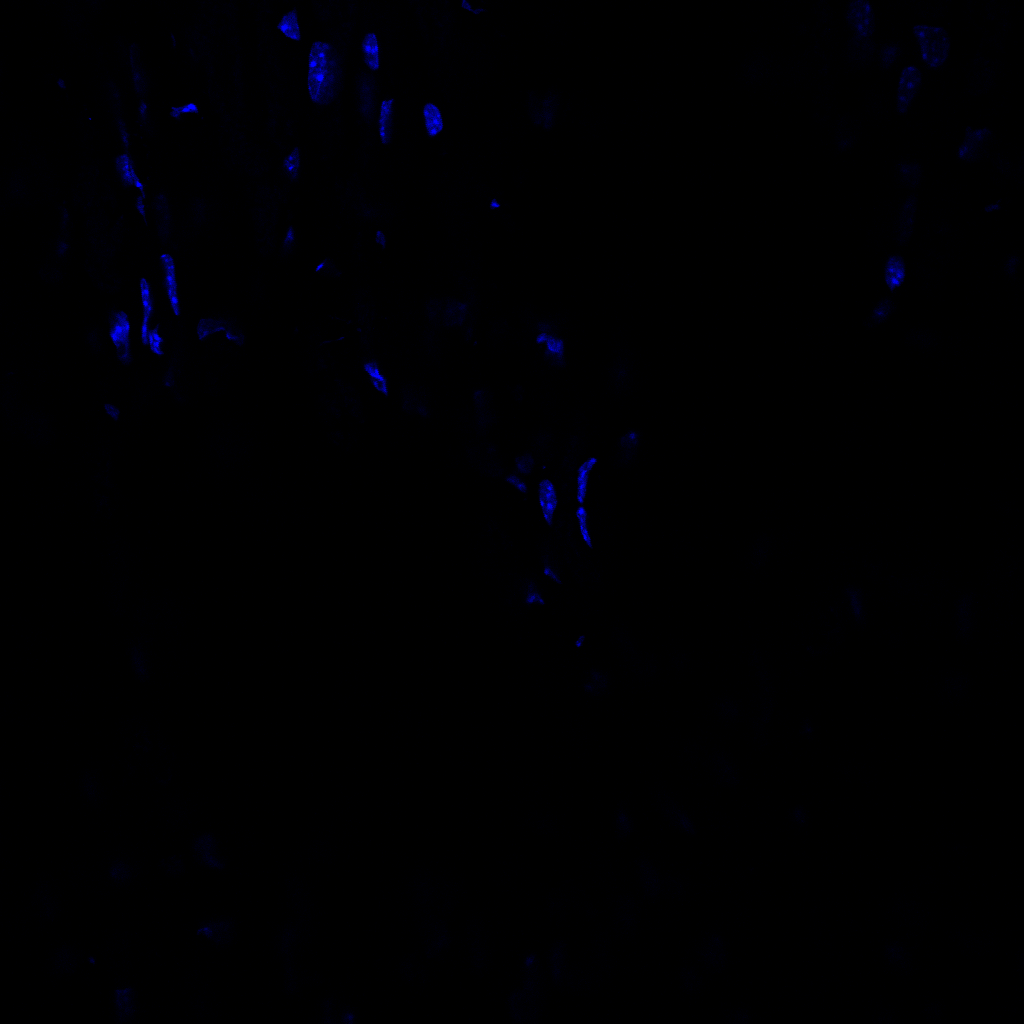

Supplement: Supplementary file 14 — Source Data for Figure 4 [file EMMM-15-e17907-s014.zip › SourceData_Fig_4/Fig_4_SourceData_images/3H/ABT_II_aged_12_dpi_no_reconex_cjun_19.lif_Series001/ABT_II_aged_12_dpi_no_reconex_cjun_19.lif_Series001_z00_ch00.tif]

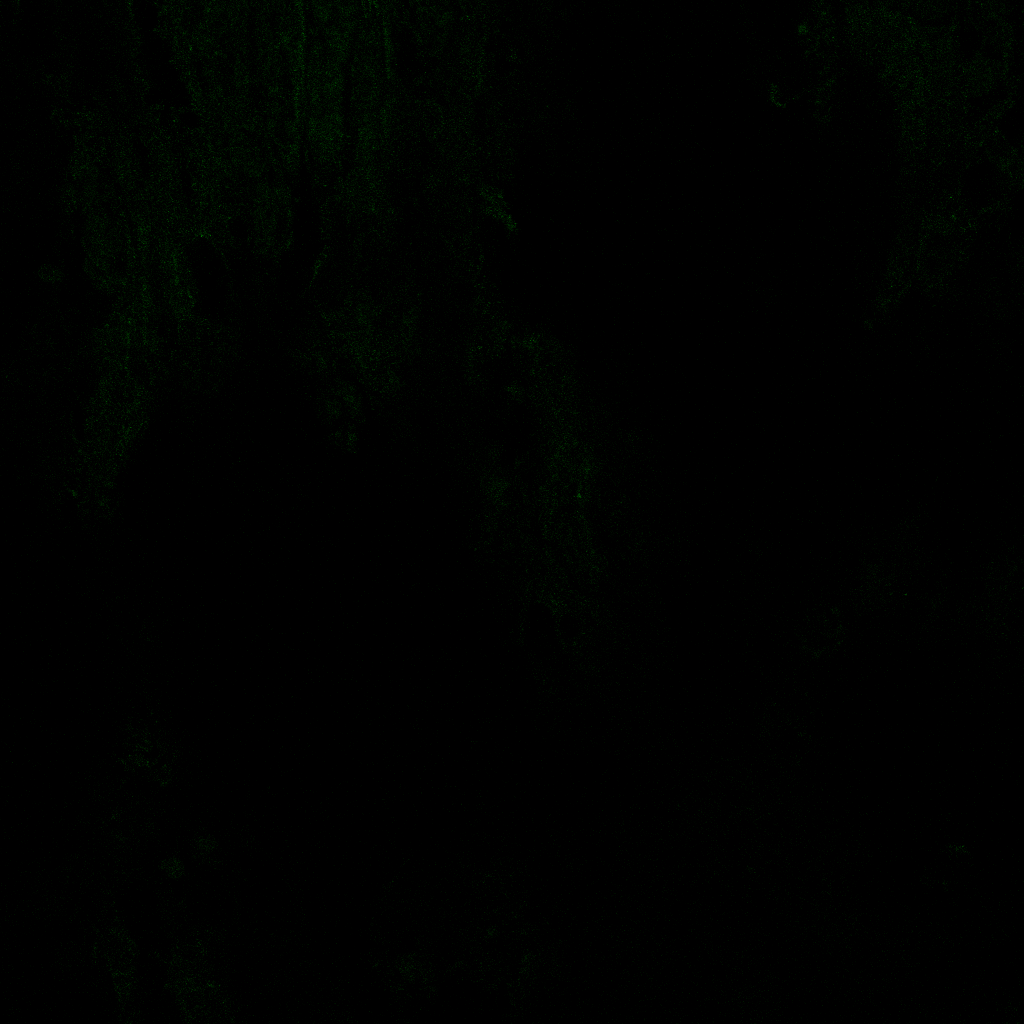

Supplement: Supplementary file 14 — Source Data for Figure 4 [file EMMM-15-e17907-s014.zip › SourceData_Fig_4/Fig_4_SourceData_images/3H/ABT_II_aged_12_dpi_no_reconex_cjun_19.lif_Series001/ABT_II_aged_12_dpi_no_reconex_cjun_19.lif_Series001_z00_ch01.tif]

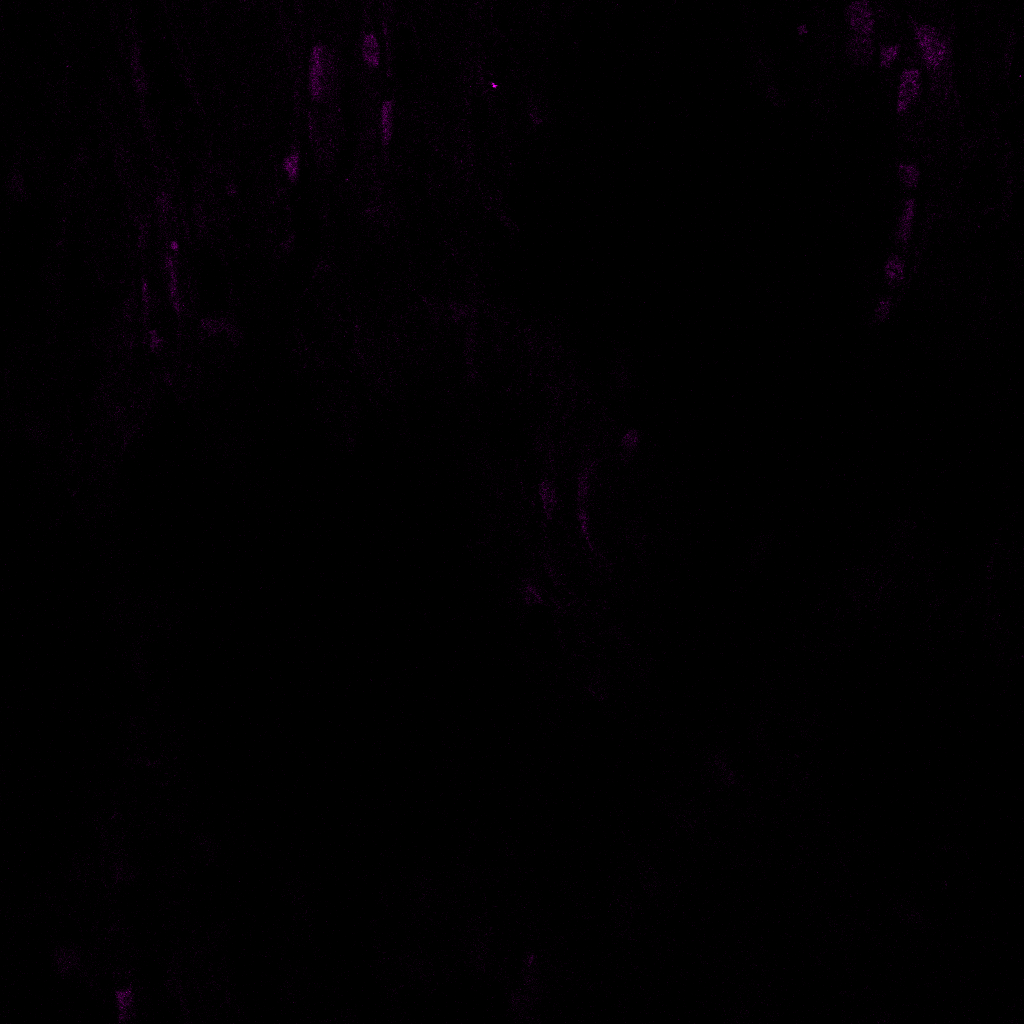

Supplement: Supplementary file 14 — Source Data for Figure 4 [file EMMM-15-e17907-s014.zip › SourceData_Fig_4/Fig_4_SourceData_images/3H/ABT_II_aged_12_dpi_no_reconex_cjun_19.lif_Series001/ABT_II_aged_12_dpi_no_reconex_cjun_19.lif_Series001_z00_ch02.tif]

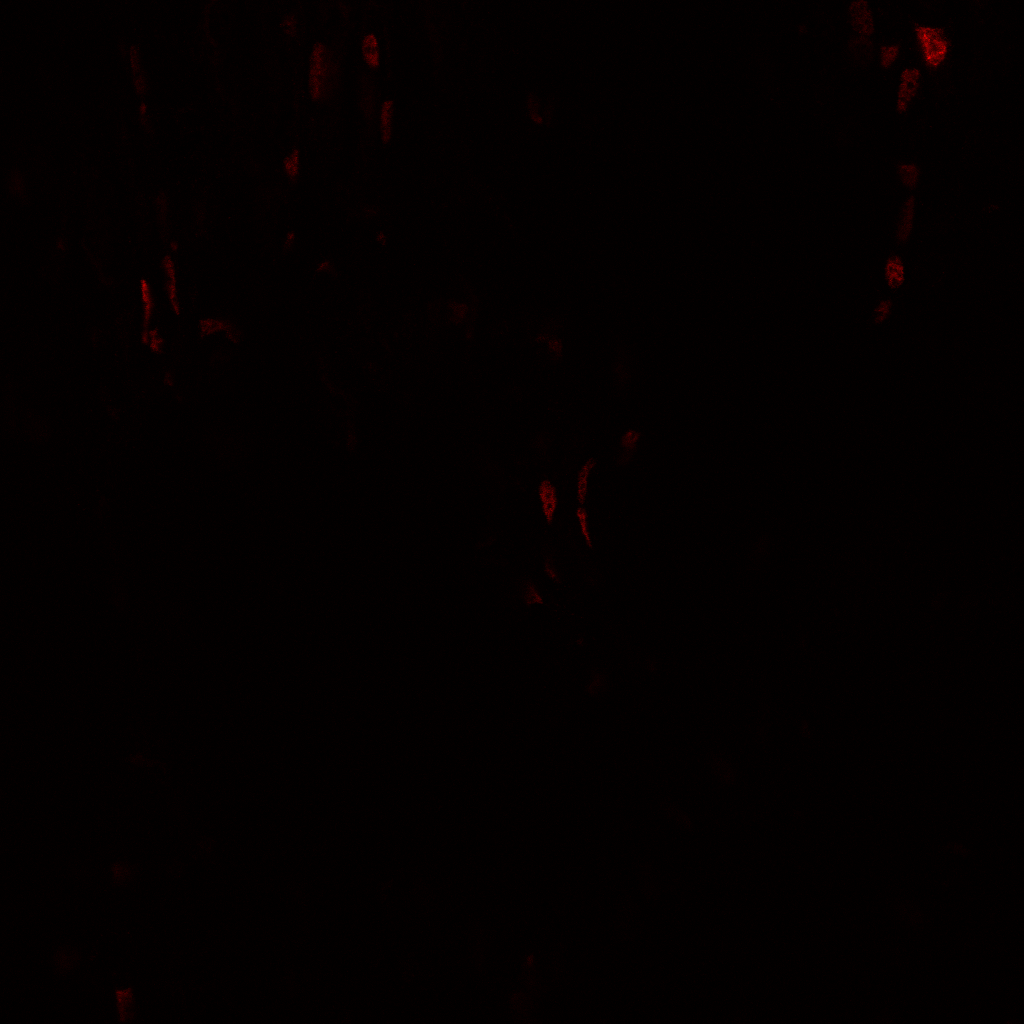

Supplement: Supplementary file 14 — Source Data for Figure 4 [file EMMM-15-e17907-s014.zip › SourceData_Fig_4/Fig_4_SourceData_images/3H/ABT_II_aged_12_dpi_no_reconex_cjun_19.lif_Series001/ABT_II_aged_12_dpi_no_reconex_cjun_19.lif_Series001_z00_ch03.tif]

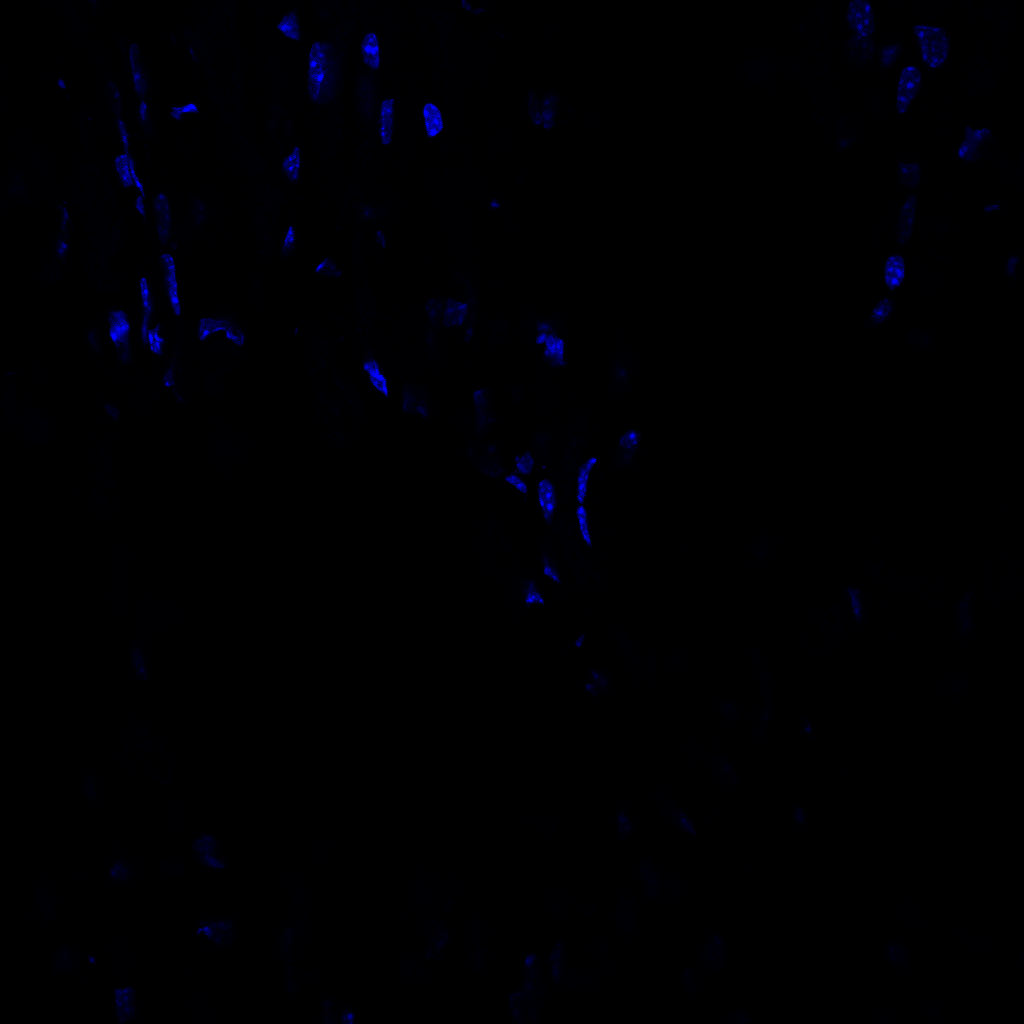

Supplement: Supplementary file 14 — Source Data for Figure 4 [file EMMM-15-e17907-s014.zip › SourceData_Fig_4/Fig_4_SourceData_images/3H/ABT_II_aged_12_dpi_no_reconex_cjun_19.lif_Series001/ABT_II_aged_12_dpi_no_reconex_cjun_19.lif_Series001_z01_ch00.tif]

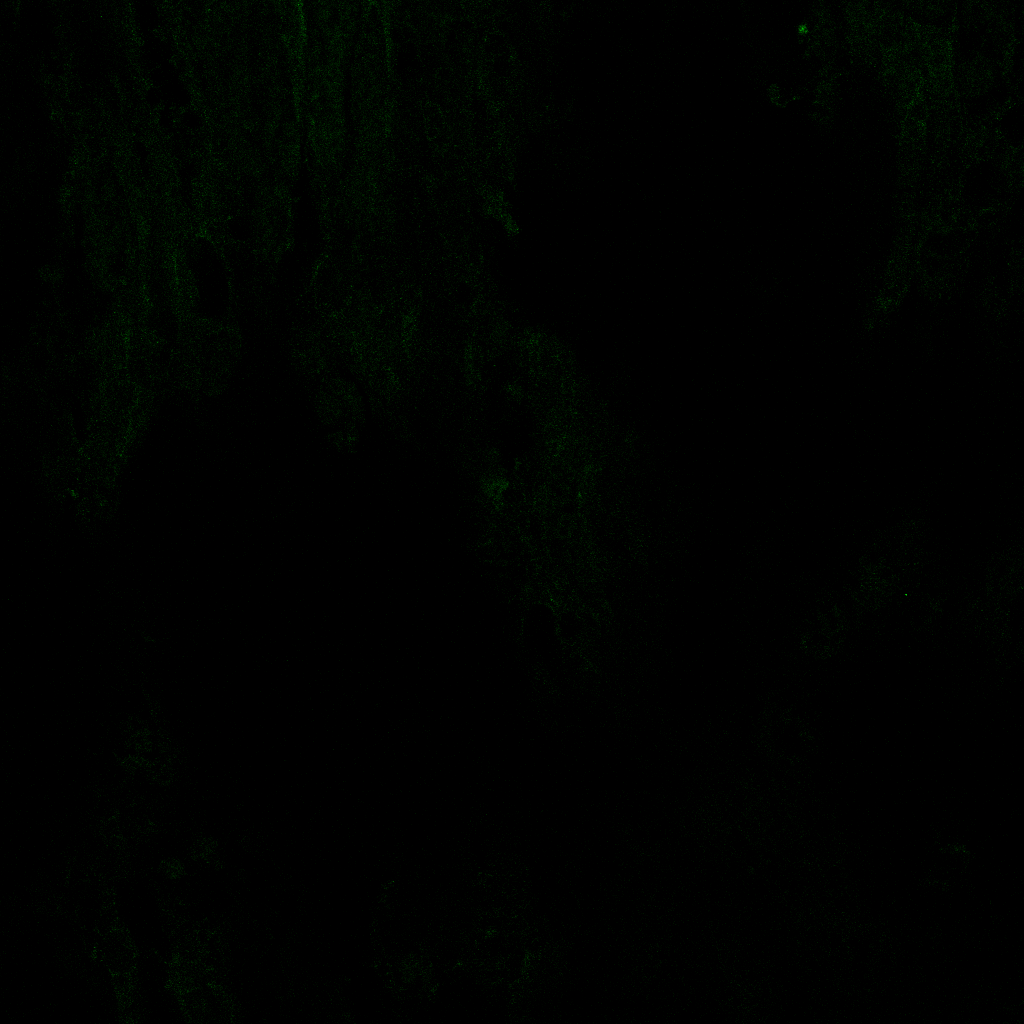

Supplement: Supplementary file 14 — Source Data for Figure 4 [file EMMM-15-e17907-s014.zip › SourceData_Fig_4/Fig_4_SourceData_images/3H/ABT_II_aged_12_dpi_no_reconex_cjun_19.lif_Series001/ABT_II_aged_12_dpi_no_reconex_cjun_19.lif_Series001_z01_ch01.tif]

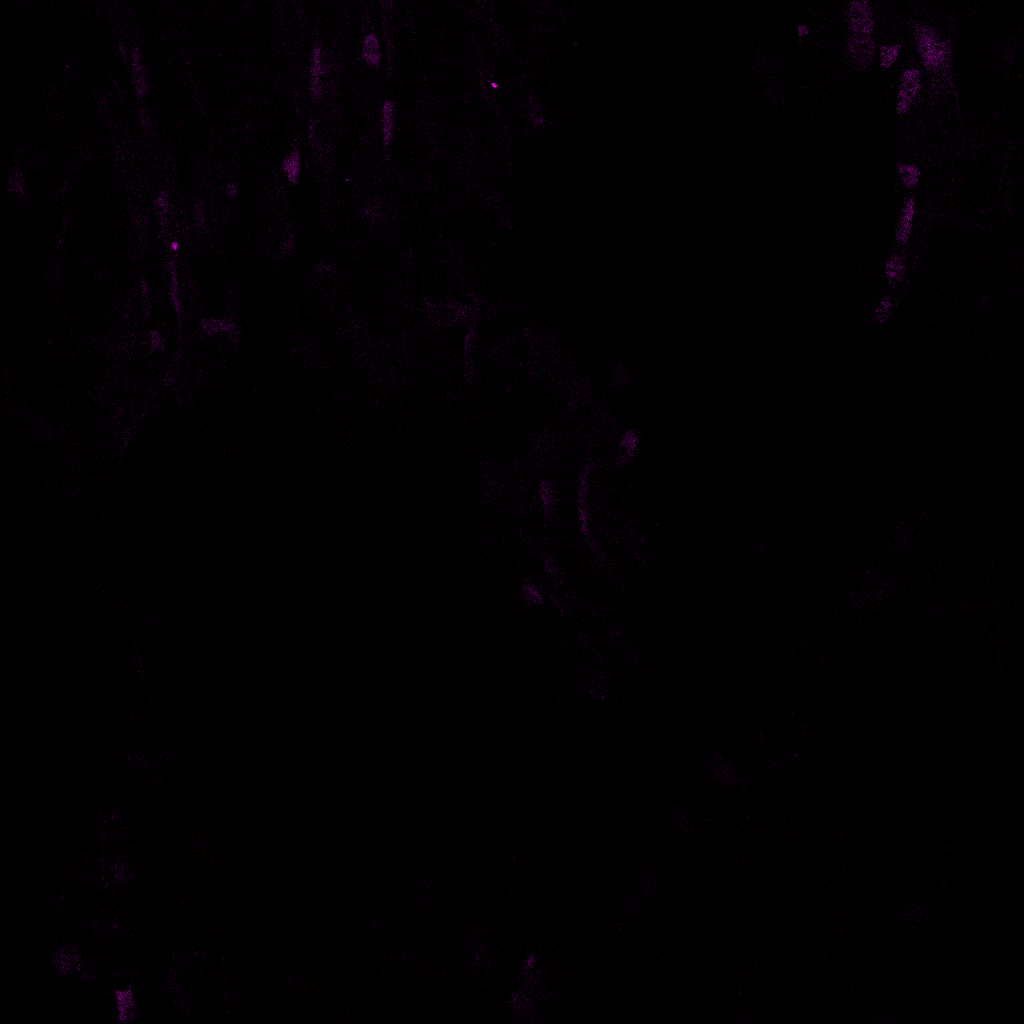

Supplement: Supplementary file 14 — Source Data for Figure 4 [file EMMM-15-e17907-s014.zip › SourceData_Fig_4/Fig_4_SourceData_images/3H/ABT_II_aged_12_dpi_no_reconex_cjun_19.lif_Series001/ABT_II_aged_12_dpi_no_reconex_cjun_19.lif_Series001_z01_ch02.tif]

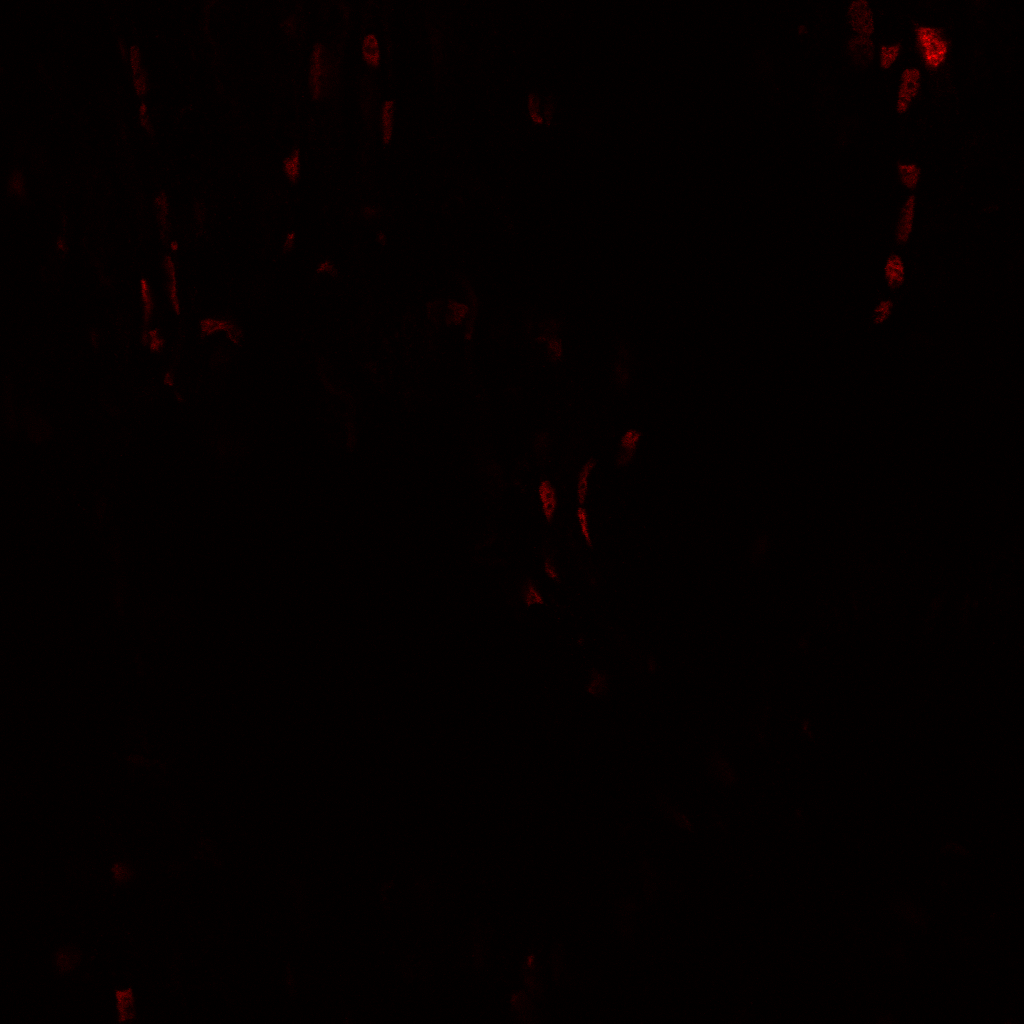

Supplement: Supplementary file 14 — Source Data for Figure 4 [file EMMM-15-e17907-s014.zip › SourceData_Fig_4/Fig_4_SourceData_images/3H/ABT_II_aged_12_dpi_no_reconex_cjun_19.lif_Series001/ABT_II_aged_12_dpi_no_reconex_cjun_19.lif_Series001_z01_ch03.tif]

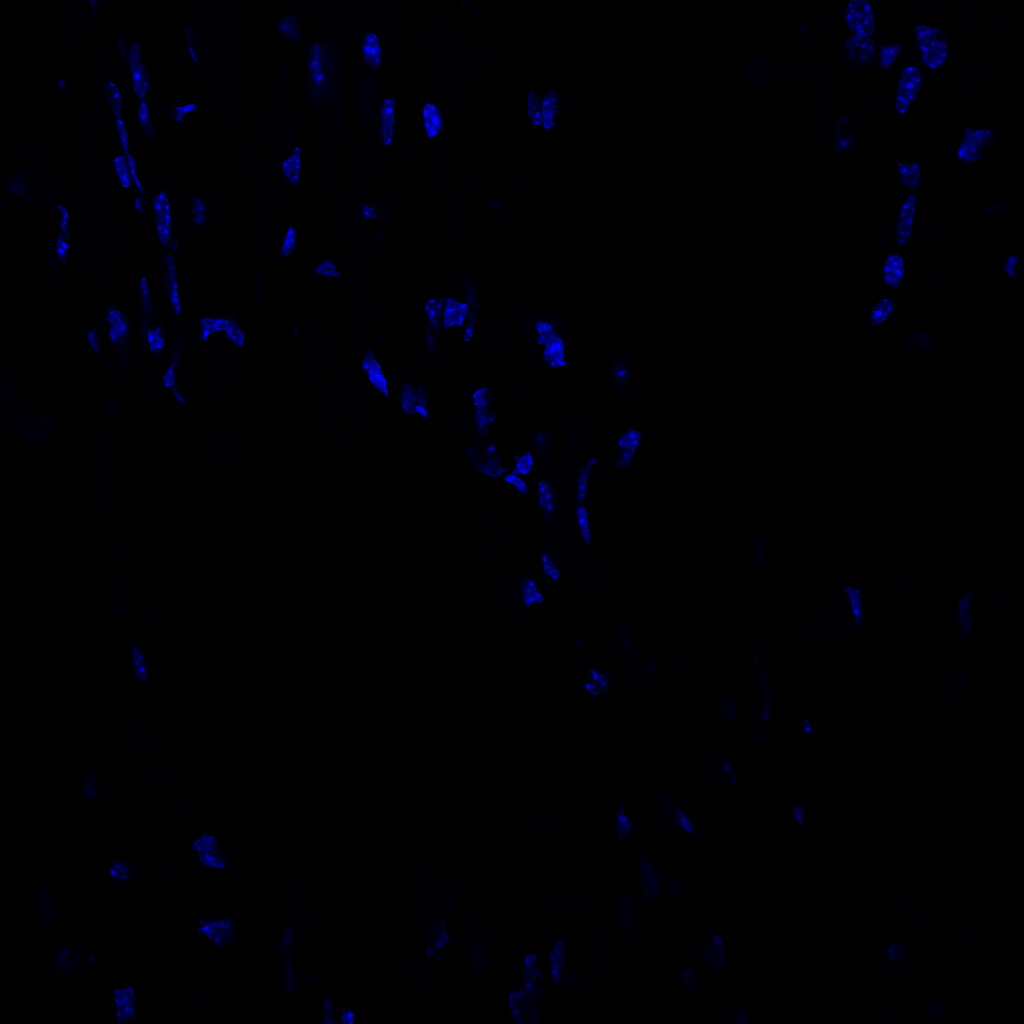

Supplement: Supplementary file 14 — Source Data for Figure 4 [file EMMM-15-e17907-s014.zip › SourceData_Fig_4/Fig_4_SourceData_images/3H/ABT_II_aged_12_dpi_no_reconex_cjun_19.lif_Series001/ABT_II_aged_12_dpi_no_reconex_cjun_19.lif_Series001_z02_ch00.tif]

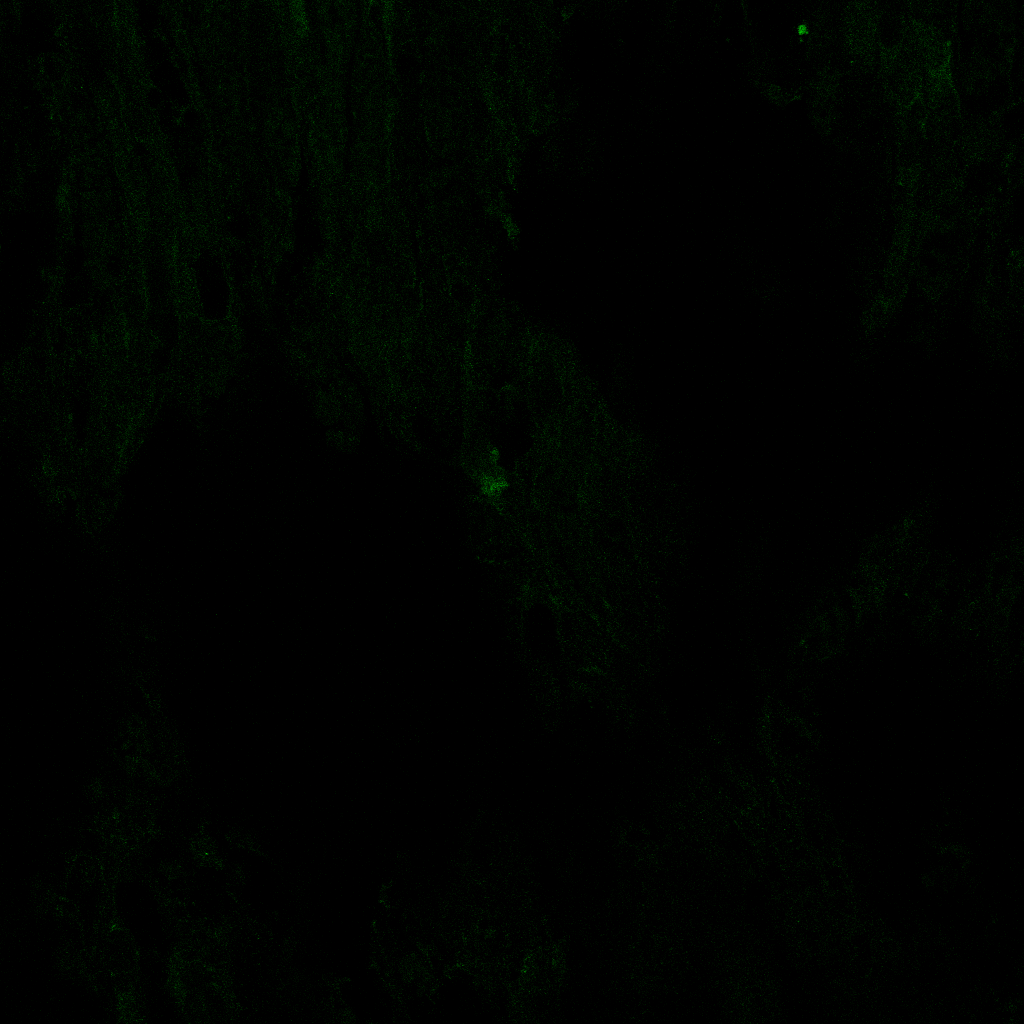

Supplement: Supplementary file 14 — Source Data for Figure 4 [file EMMM-15-e17907-s014.zip › SourceData_Fig_4/Fig_4_SourceData_images/3H/ABT_II_aged_12_dpi_no_reconex_cjun_19.lif_Series001/ABT_II_aged_12_dpi_no_reconex_cjun_19.lif_Series001_z02_ch01.tif]

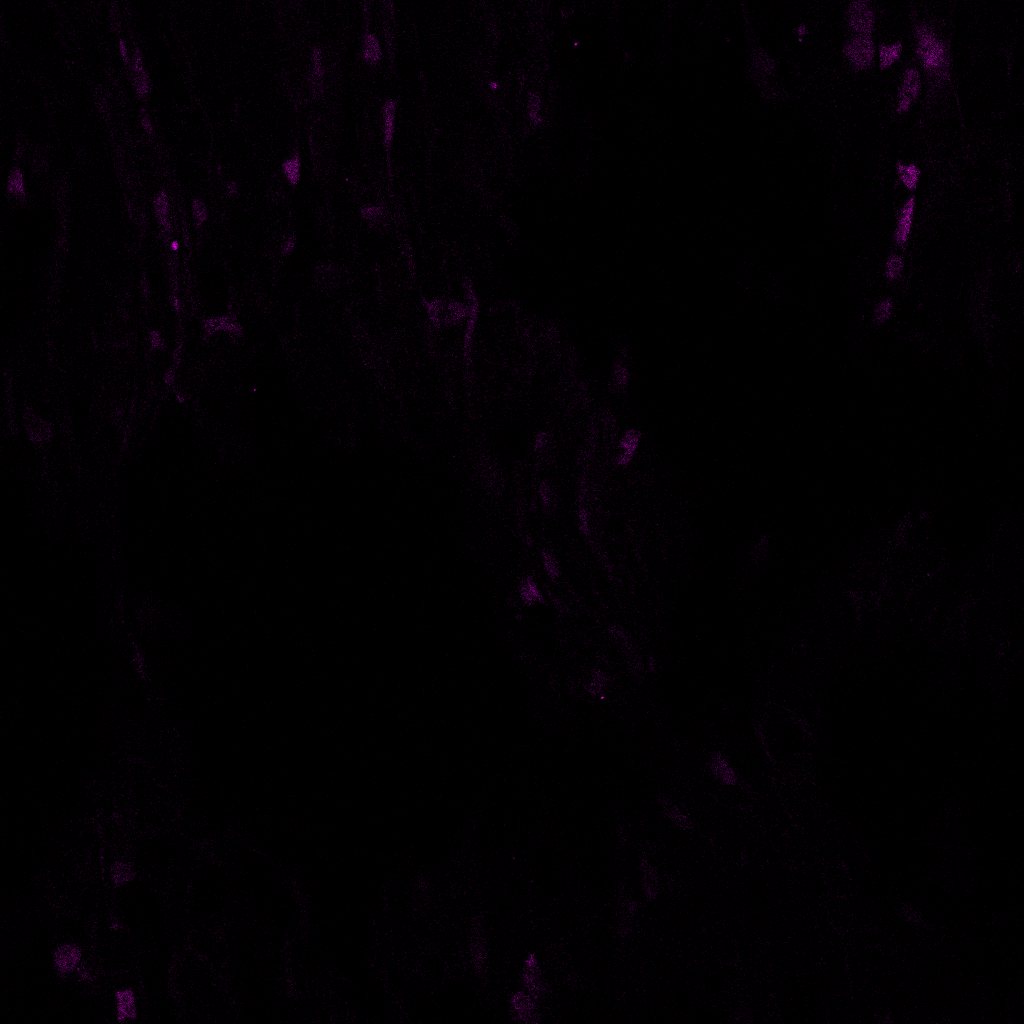

Supplement: Supplementary file 14 — Source Data for Figure 4 [file EMMM-15-e17907-s014.zip › SourceData_Fig_4/Fig_4_SourceData_images/3H/ABT_II_aged_12_dpi_no_reconex_cjun_19.lif_Series001/ABT_II_aged_12_dpi_no_reconex_cjun_19.lif_Series001_z02_ch02.tif]

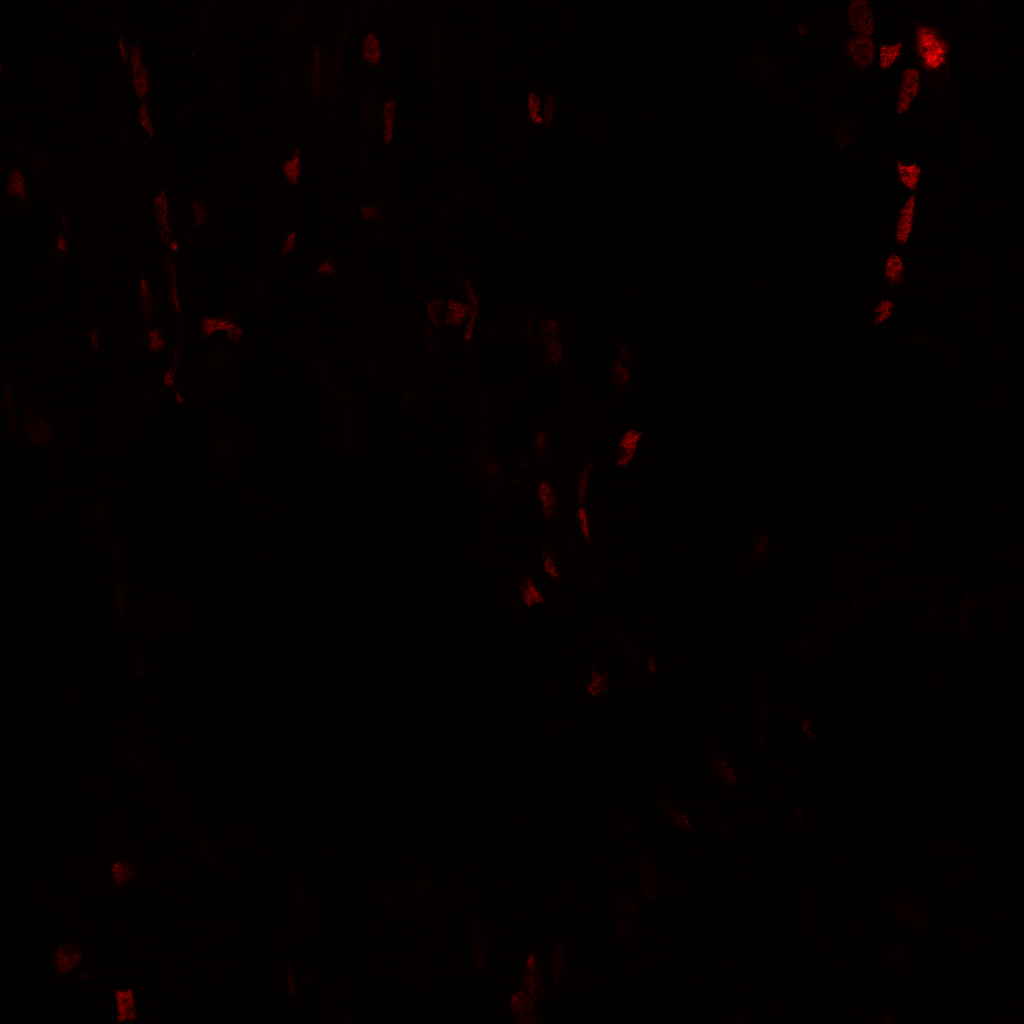

Supplement: Supplementary file 14 — Source Data for Figure 4 [file EMMM-15-e17907-s014.zip › SourceData_Fig_4/Fig_4_SourceData_images/3H/ABT_II_aged_12_dpi_no_reconex_cjun_19.lif_Series001/ABT_II_aged_12_dpi_no_reconex_cjun_19.lif_Series001_z02_ch03.tif]

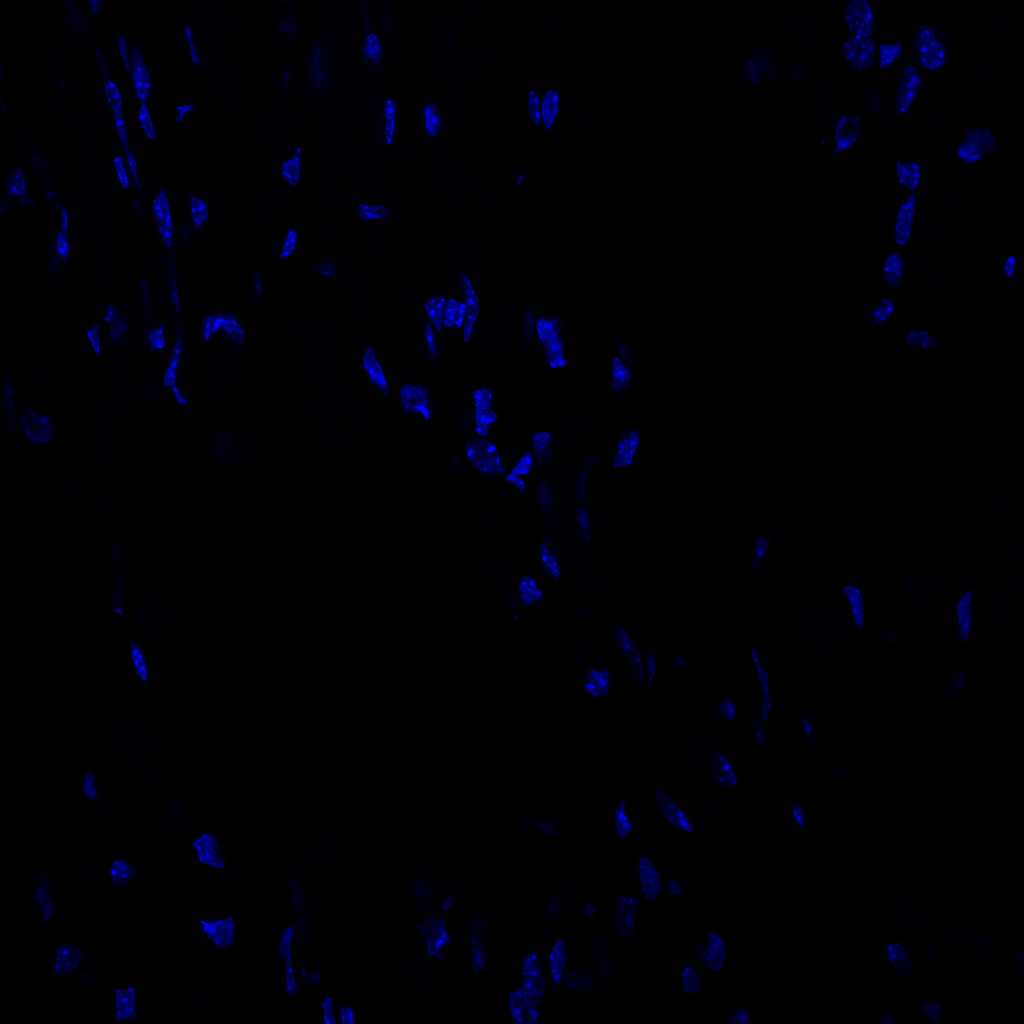

Supplement: Supplementary file 14 — Source Data for Figure 4 [file EMMM-15-e17907-s014.zip › SourceData_Fig_4/Fig_4_SourceData_images/3H/ABT_II_aged_12_dpi_no_reconex_cjun_19.lif_Series001/ABT_II_aged_12_dpi_no_reconex_cjun_19.lif_Series001_z03_ch00.tif]

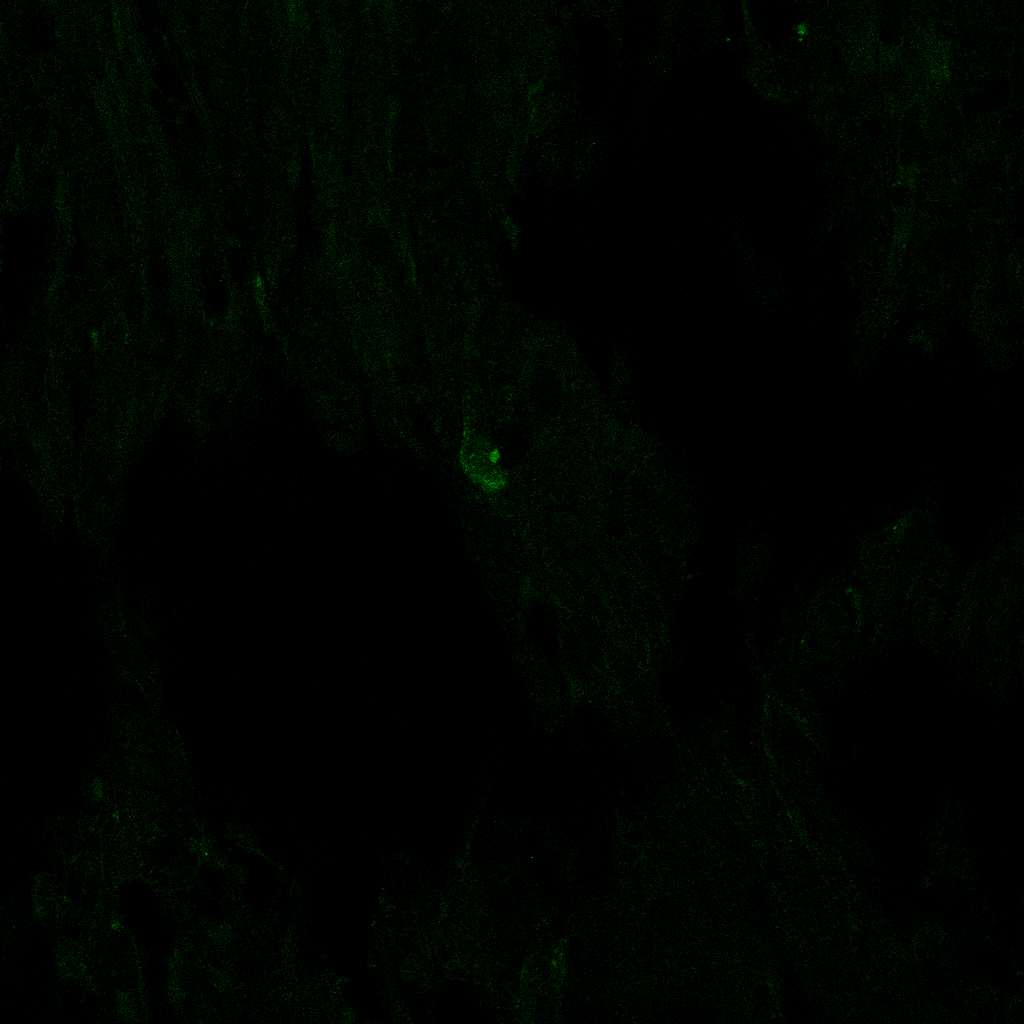

Supplement: Supplementary file 14 — Source Data for Figure 4 [file EMMM-15-e17907-s014.zip › SourceData_Fig_4/Fig_4_SourceData_images/3H/ABT_II_aged_12_dpi_no_reconex_cjun_19.lif_Series001/ABT_II_aged_12_dpi_no_reconex_cjun_19.lif_Series001_z03_ch01.tif]

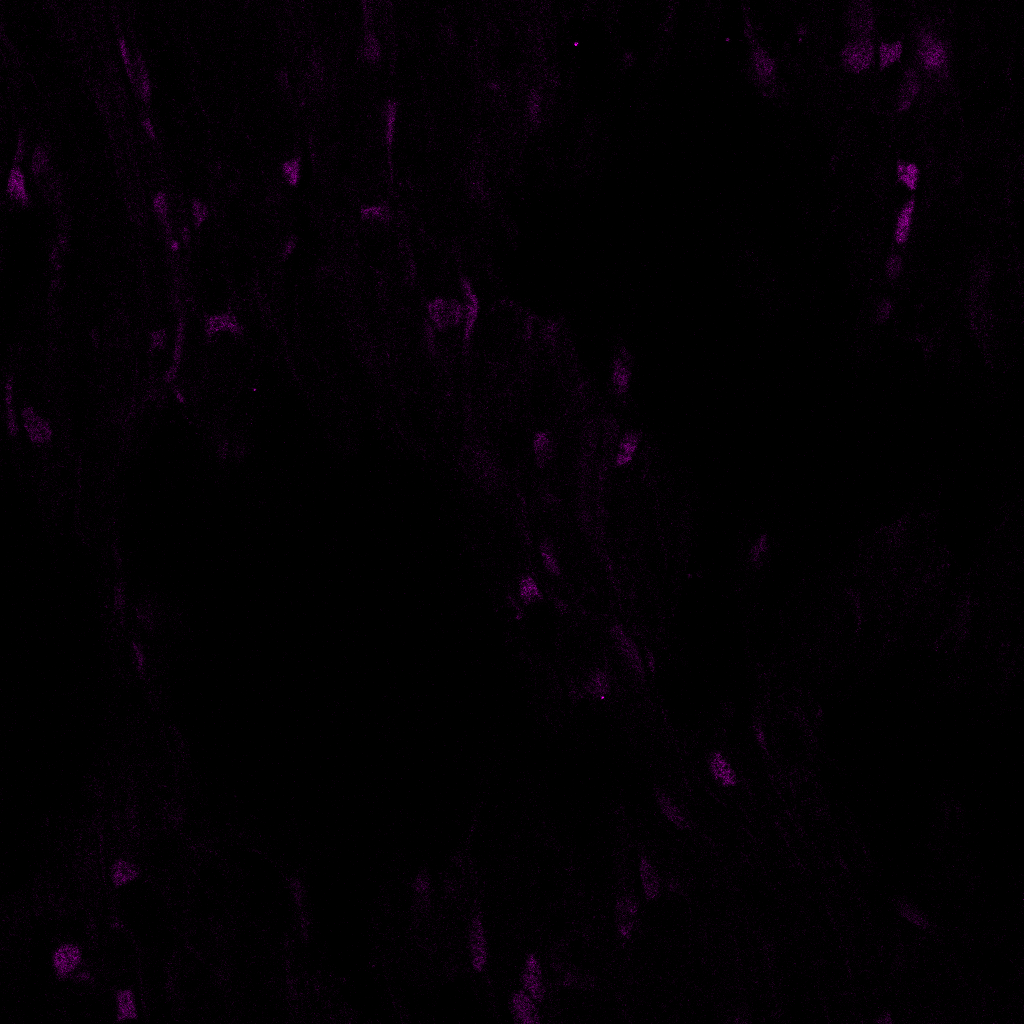

Supplement: Supplementary file 14 — Source Data for Figure 4 [file EMMM-15-e17907-s014.zip › SourceData_Fig_4/Fig_4_SourceData_images/3H/ABT_II_aged_12_dpi_no_reconex_cjun_19.lif_Series001/ABT_II_aged_12_dpi_no_reconex_cjun_19.lif_Series001_z03_ch02.tif]

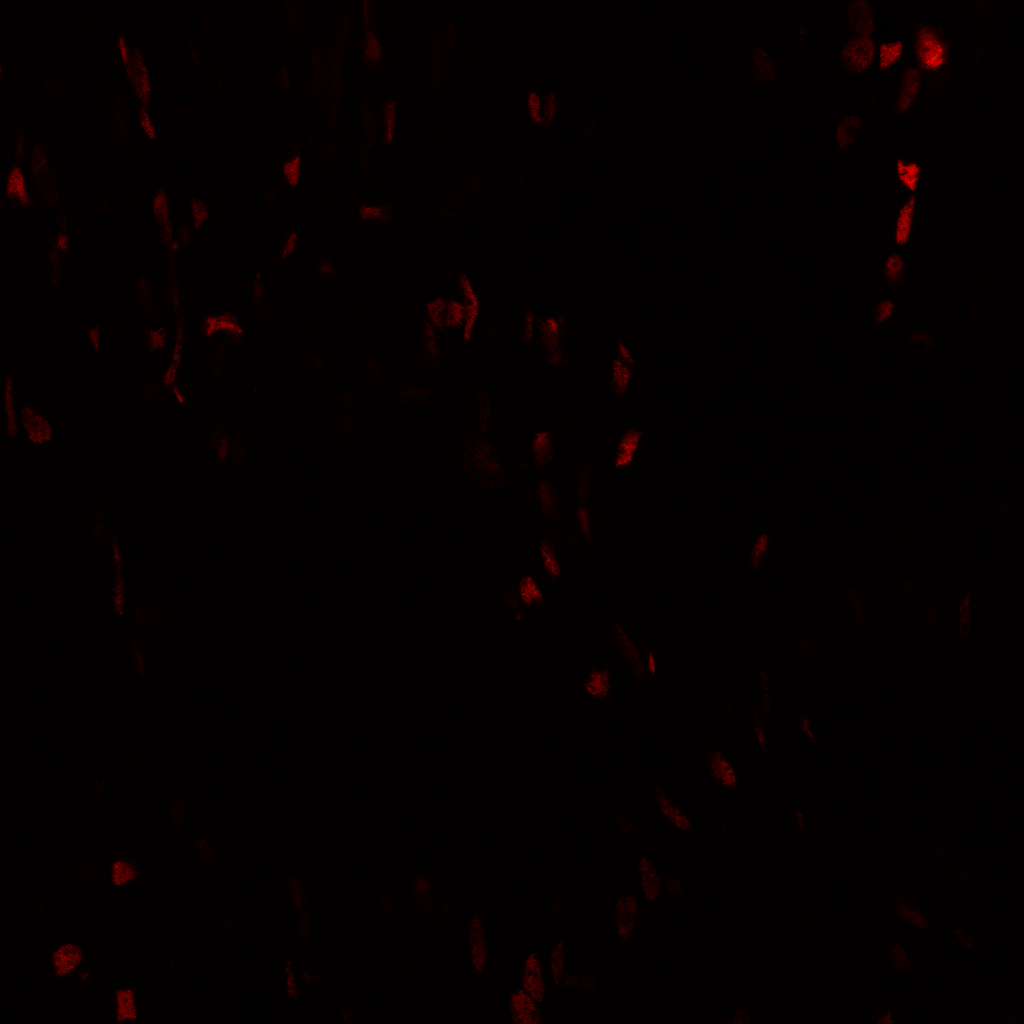

Supplement: Supplementary file 14 — Source Data for Figure 4 [file EMMM-15-e17907-s014.zip › SourceData_Fig_4/Fig_4_SourceData_images/3H/ABT_II_aged_12_dpi_no_reconex_cjun_19.lif_Series001/ABT_II_aged_12_dpi_no_reconex_cjun_19.lif_Series001_z03_ch03.tif]

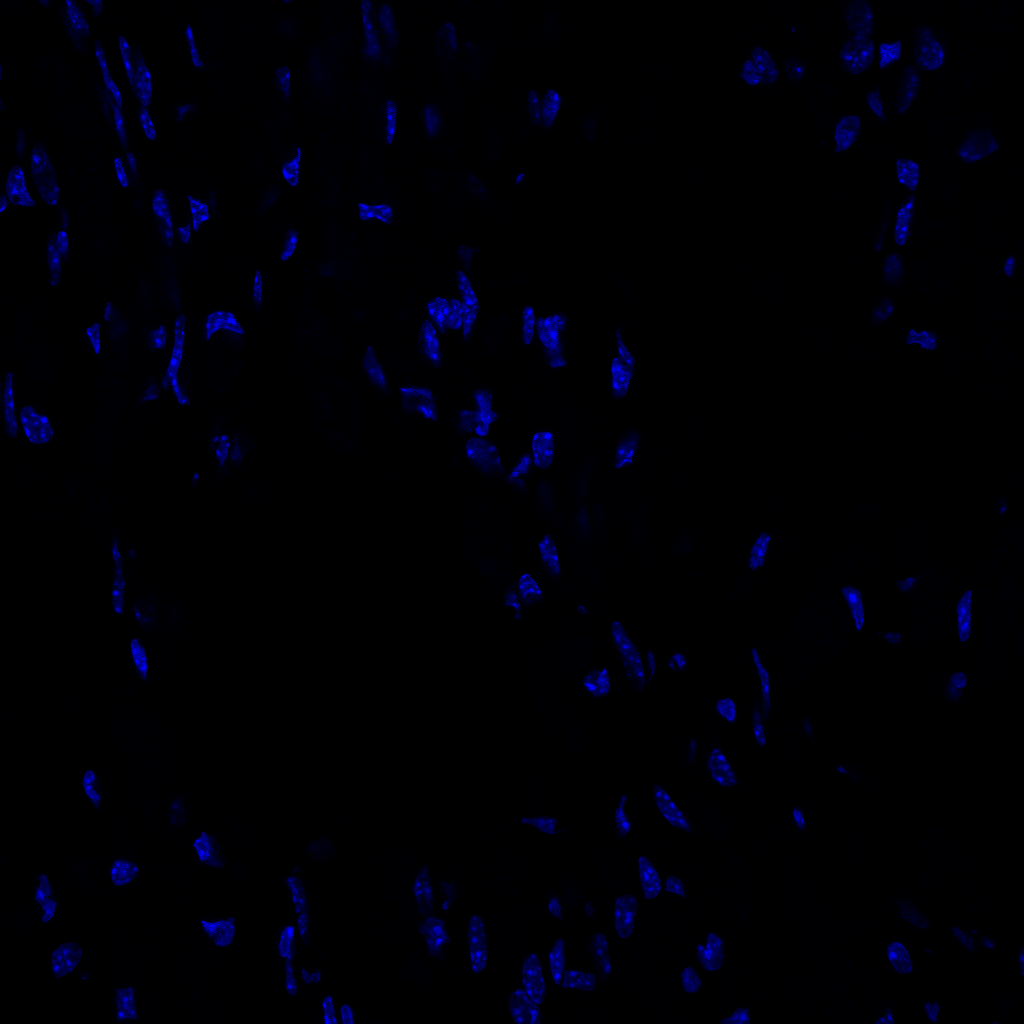

Supplement: Supplementary file 14 — Source Data for Figure 4 [file EMMM-15-e17907-s014.zip › SourceData_Fig_4/Fig_4_SourceData_images/3H/ABT_II_aged_12_dpi_no_reconex_cjun_19.lif_Series001/ABT_II_aged_12_dpi_no_reconex_cjun_19.lif_Series001_z04_ch00.tif]

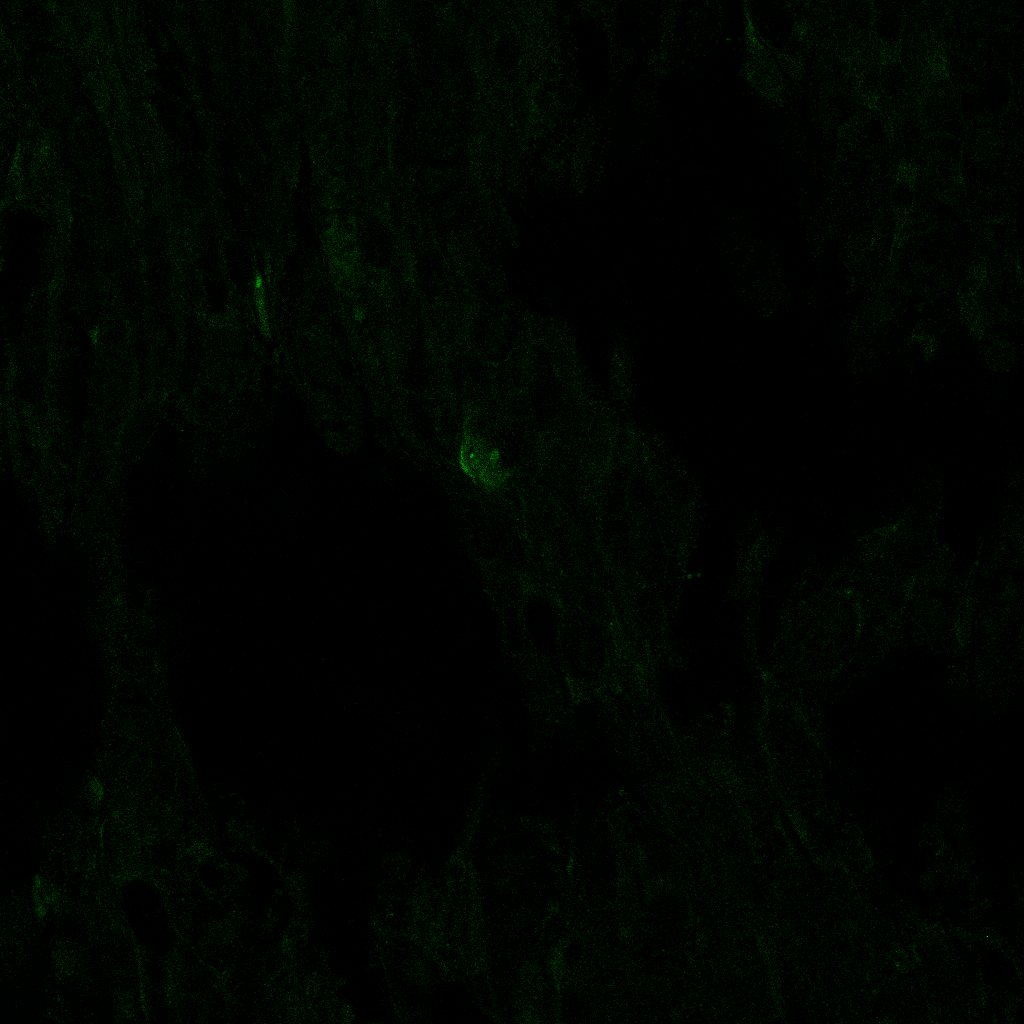

Supplement: Supplementary file 14 — Source Data for Figure 4 [file EMMM-15-e17907-s014.zip › SourceData_Fig_4/Fig_4_SourceData_images/3H/ABT_II_aged_12_dpi_no_reconex_cjun_19.lif_Series001/ABT_II_aged_12_dpi_no_reconex_cjun_19.lif_Series001_z04_ch01.tif]

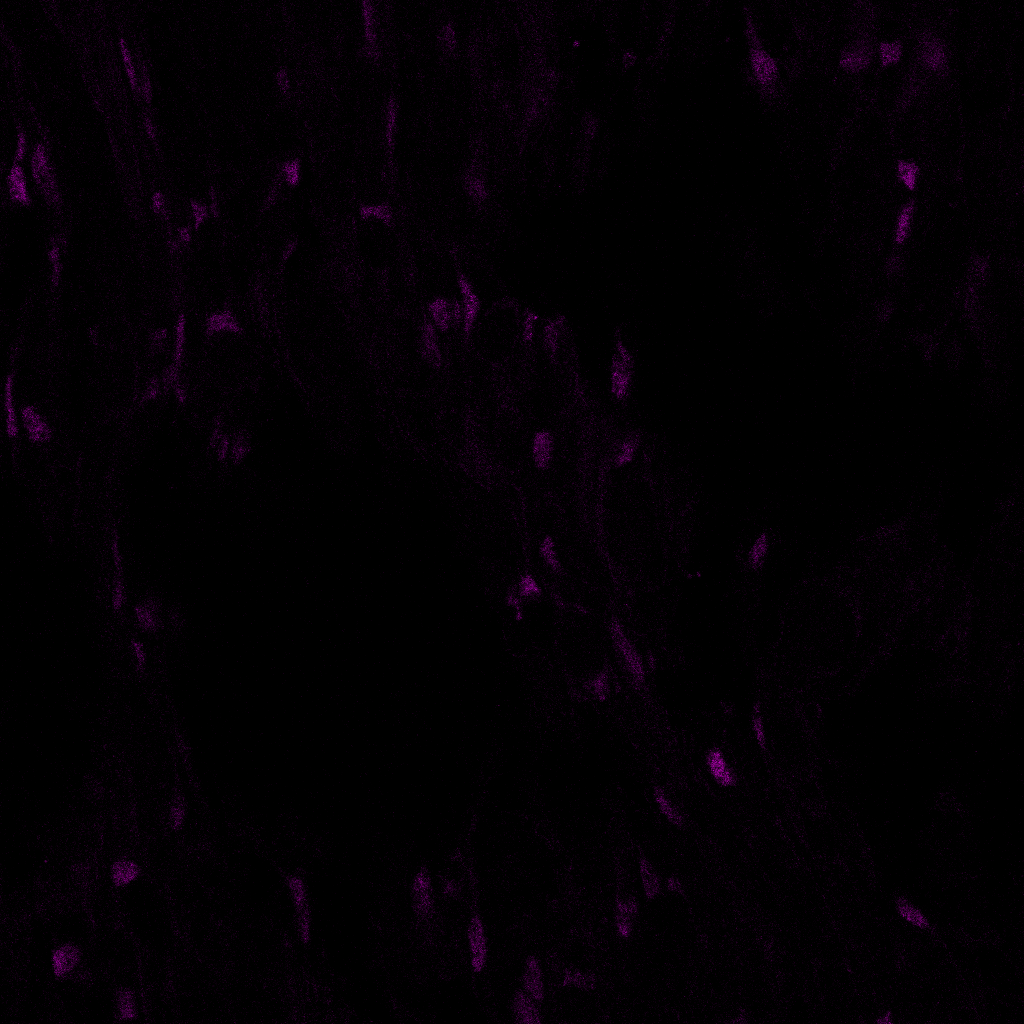

Supplement: Supplementary file 14 — Source Data for Figure 4 [file EMMM-15-e17907-s014.zip › SourceData_Fig_4/Fig_4_SourceData_images/3H/ABT_II_aged_12_dpi_no_reconex_cjun_19.lif_Series001/ABT_II_aged_12_dpi_no_reconex_cjun_19.lif_Series001_z04_ch02.tif]

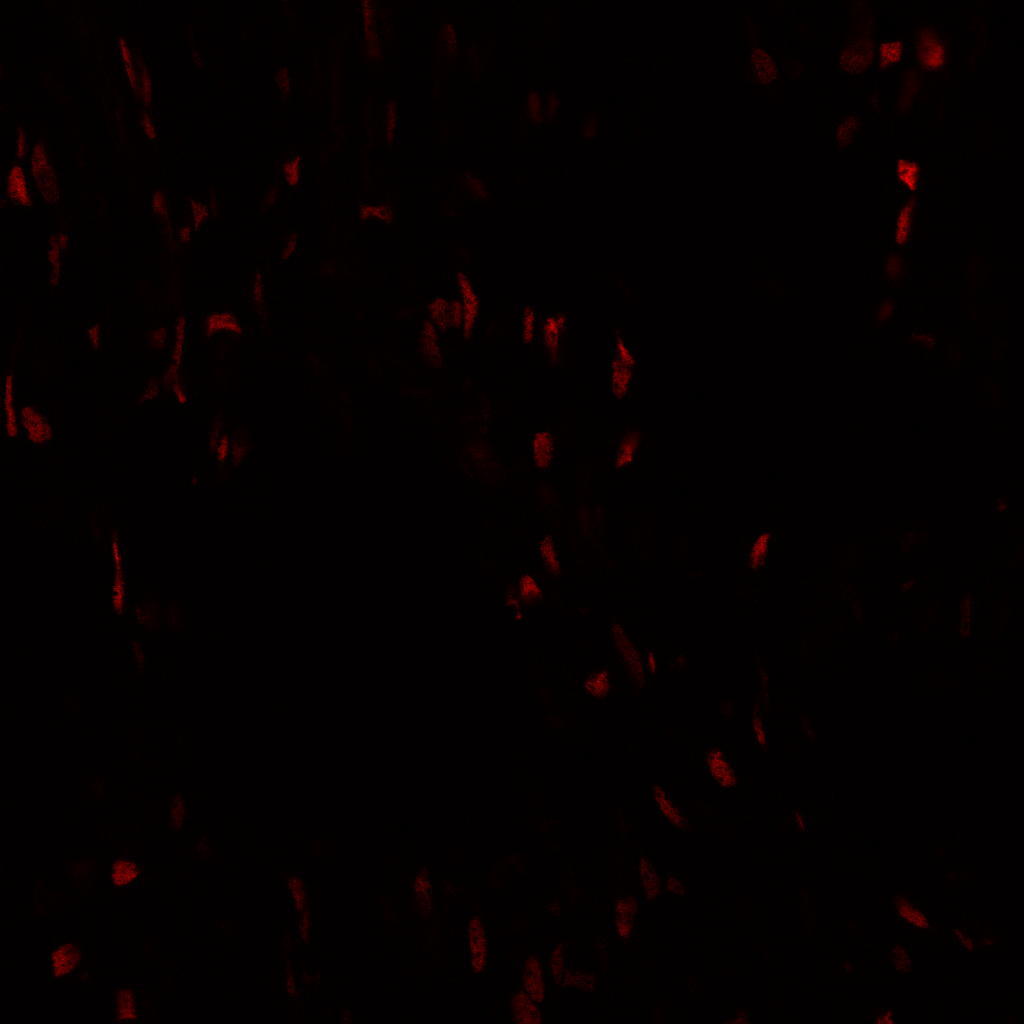

Supplement: Supplementary file 14 — Source Data for Figure 4 [file EMMM-15-e17907-s014.zip › SourceData_Fig_4/Fig_4_SourceData_images/3H/ABT_II_aged_12_dpi_no_reconex_cjun_19.lif_Series001/ABT_II_aged_12_dpi_no_reconex_cjun_19.lif_Series001_z04_ch03.tif]

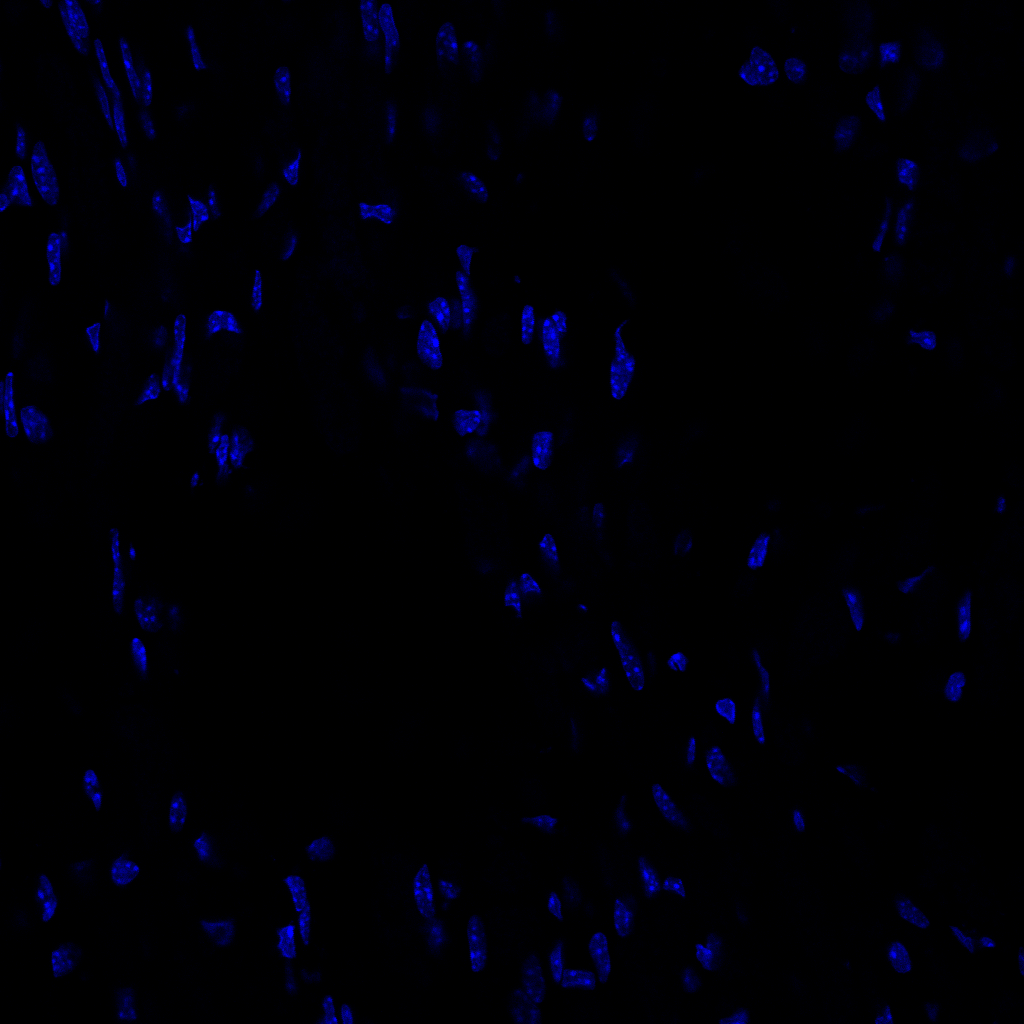

Supplement: Supplementary file 14 — Source Data for Figure 4 [file EMMM-15-e17907-s014.zip › SourceData_Fig_4/Fig_4_SourceData_images/3H/ABT_II_aged_12_dpi_no_reconex_cjun_19.lif_Series001/ABT_II_aged_12_dpi_no_reconex_cjun_19.lif_Series001_z05_ch00.tif]

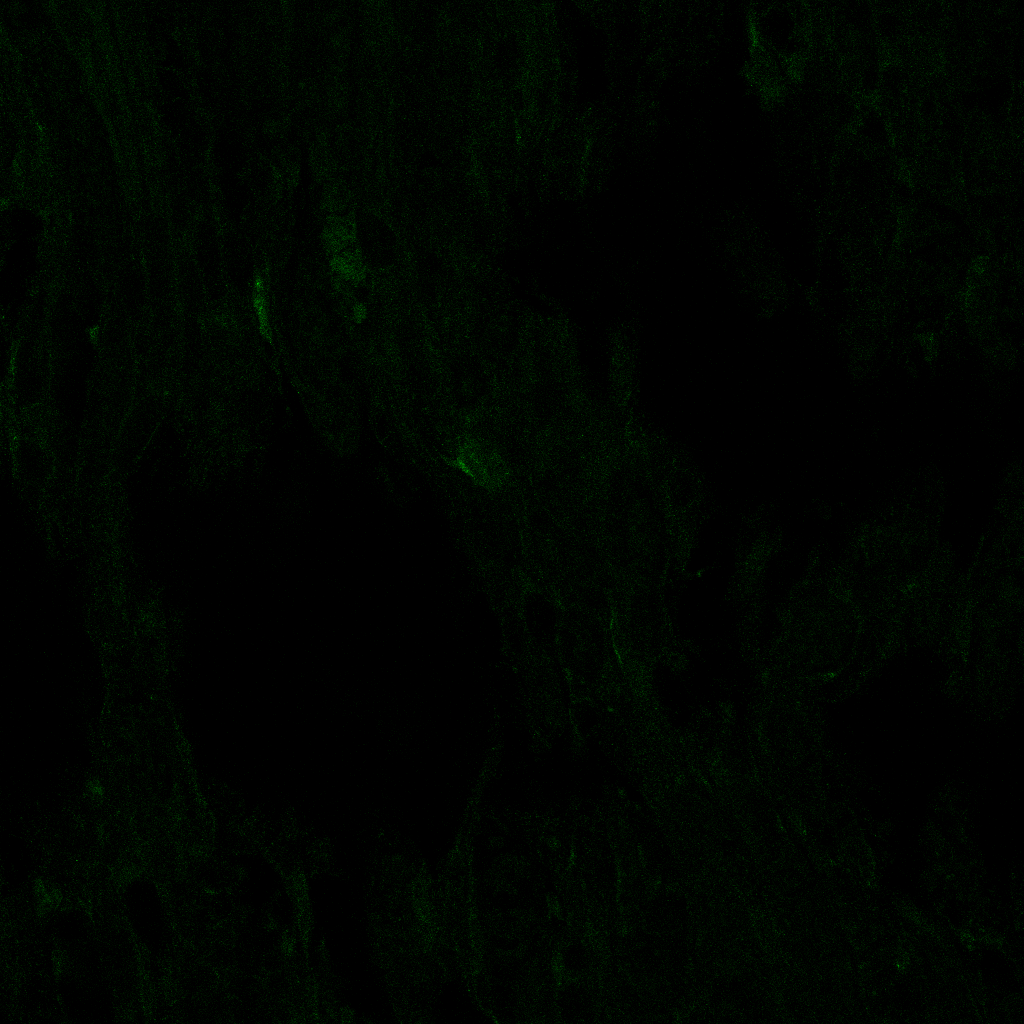

Supplement: Supplementary file 14 — Source Data for Figure 4 [file EMMM-15-e17907-s014.zip › SourceData_Fig_4/Fig_4_SourceData_images/3H/ABT_II_aged_12_dpi_no_reconex_cjun_19.lif_Series001/ABT_II_aged_12_dpi_no_reconex_cjun_19.lif_Series001_z05_ch01.tif]

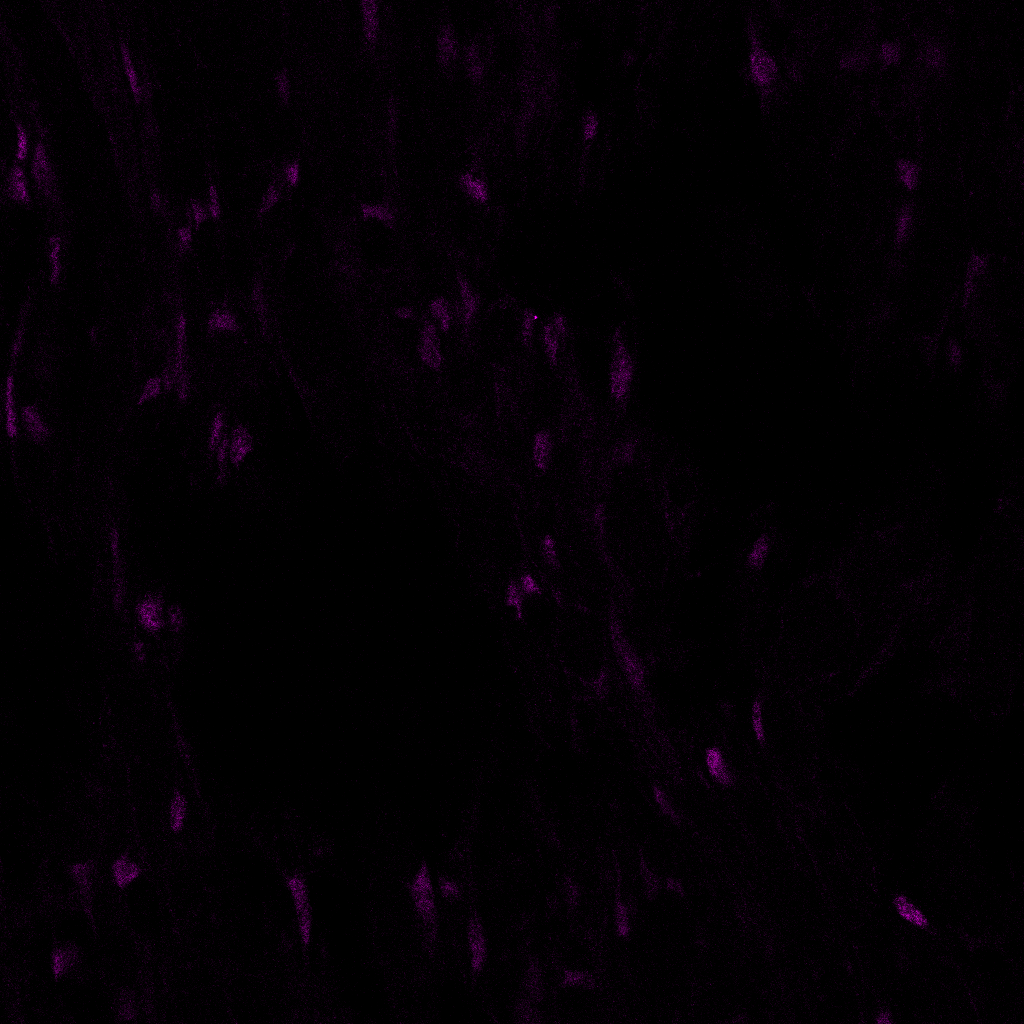

Supplement: Supplementary file 14 — Source Data for Figure 4 [file EMMM-15-e17907-s014.zip › SourceData_Fig_4/Fig_4_SourceData_images/3H/ABT_II_aged_12_dpi_no_reconex_cjun_19.lif_Series001/ABT_II_aged_12_dpi_no_reconex_cjun_19.lif_Series001_z05_ch02.tif]

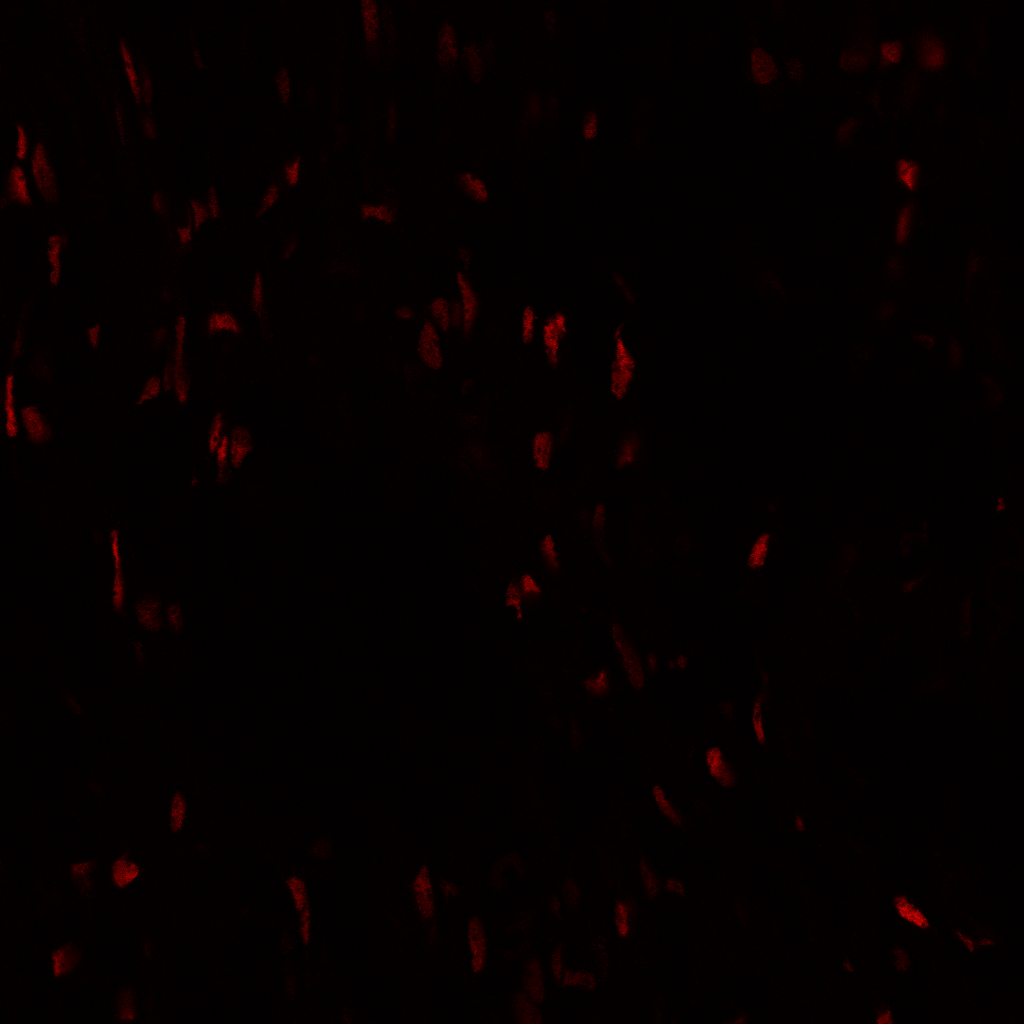

Supplement: Supplementary file 14 — Source Data for Figure 4 [file EMMM-15-e17907-s014.zip › SourceData_Fig_4/Fig_4_SourceData_images/3H/ABT_II_aged_12_dpi_no_reconex_cjun_19.lif_Series001/ABT_II_aged_12_dpi_no_reconex_cjun_19.lif_Series001_z05_ch03.tif]

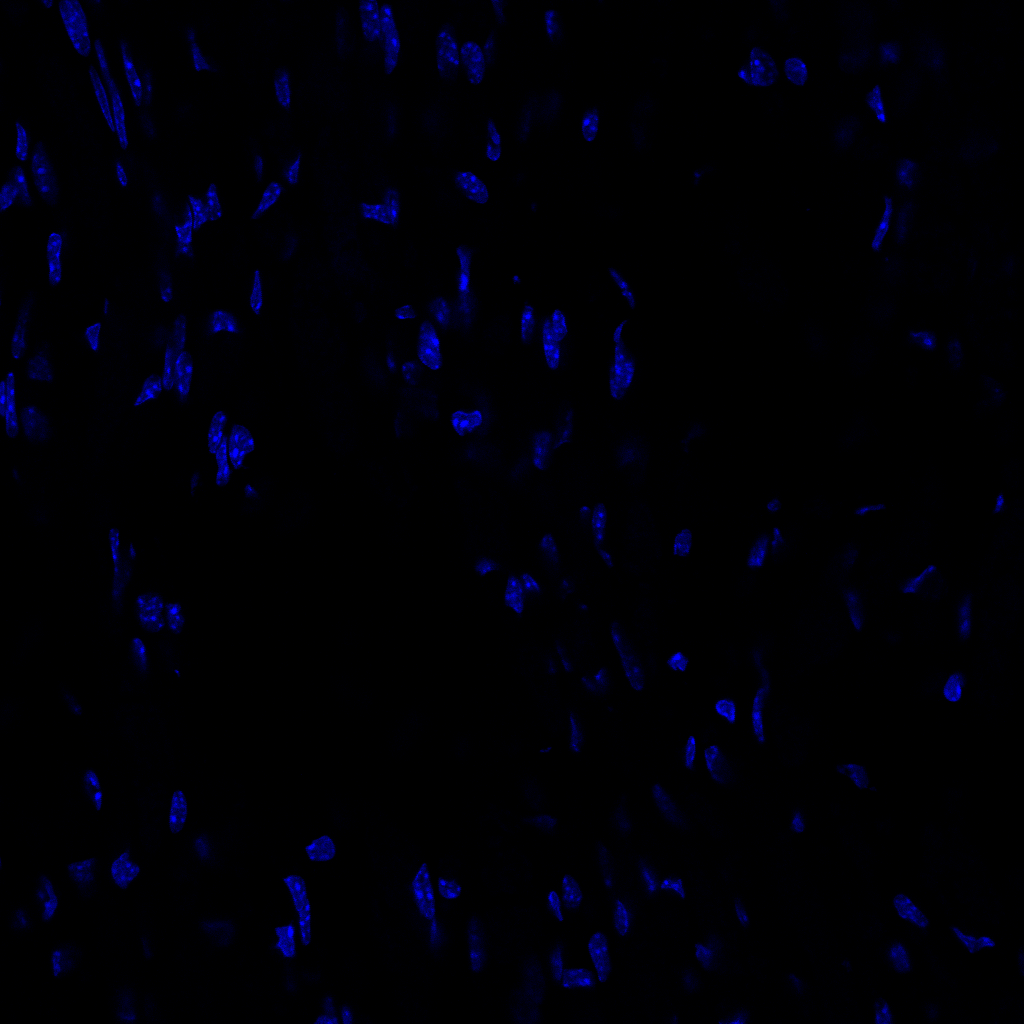

Supplement: Supplementary file 14 — Source Data for Figure 4 [file EMMM-15-e17907-s014.zip › SourceData_Fig_4/Fig_4_SourceData_images/3H/ABT_II_aged_12_dpi_no_reconex_cjun_19.lif_Series001/ABT_II_aged_12_dpi_no_reconex_cjun_19.lif_Series001_z06_ch00.tif]

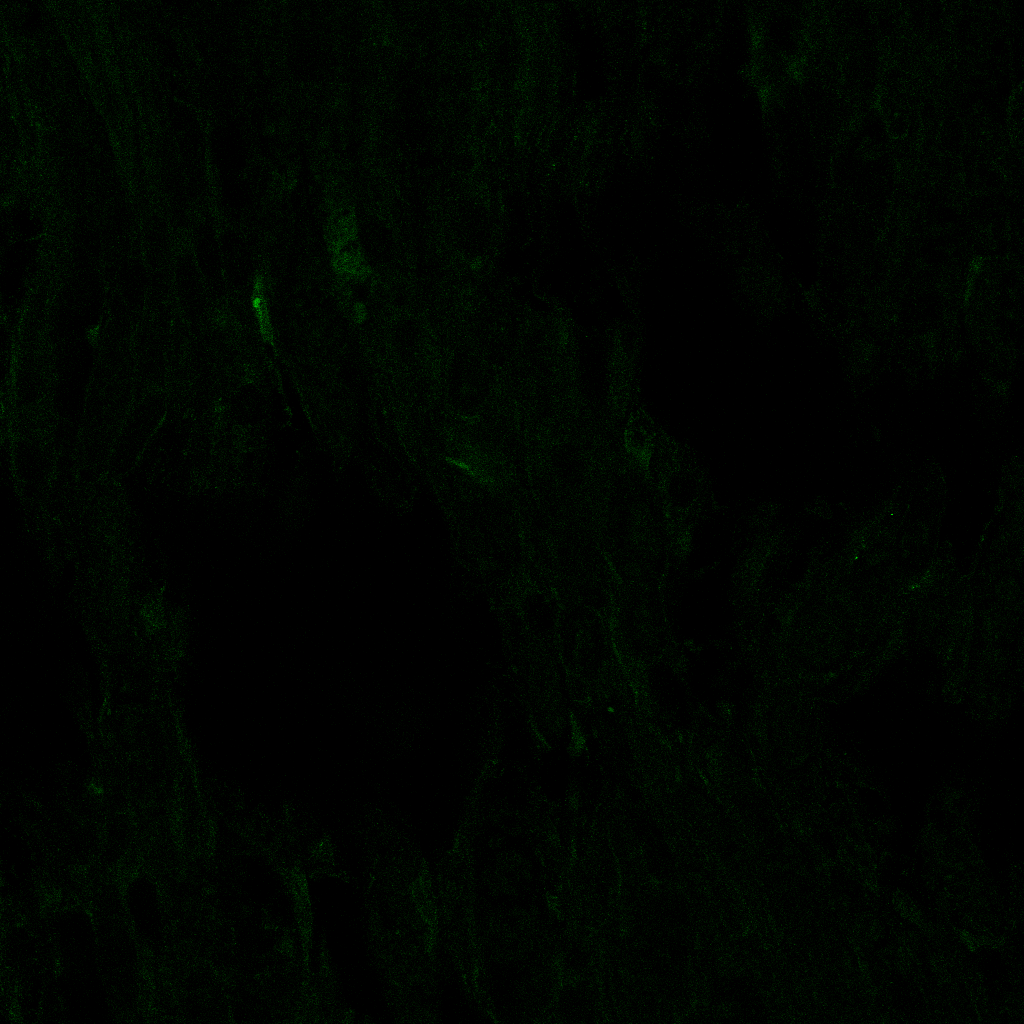

Supplement: Supplementary file 14 — Source Data for Figure 4 [file EMMM-15-e17907-s014.zip › SourceData_Fig_4/Fig_4_SourceData_images/3H/ABT_II_aged_12_dpi_no_reconex_cjun_19.lif_Series001/ABT_II_aged_12_dpi_no_reconex_cjun_19.lif_Series001_z06_ch01.tif]

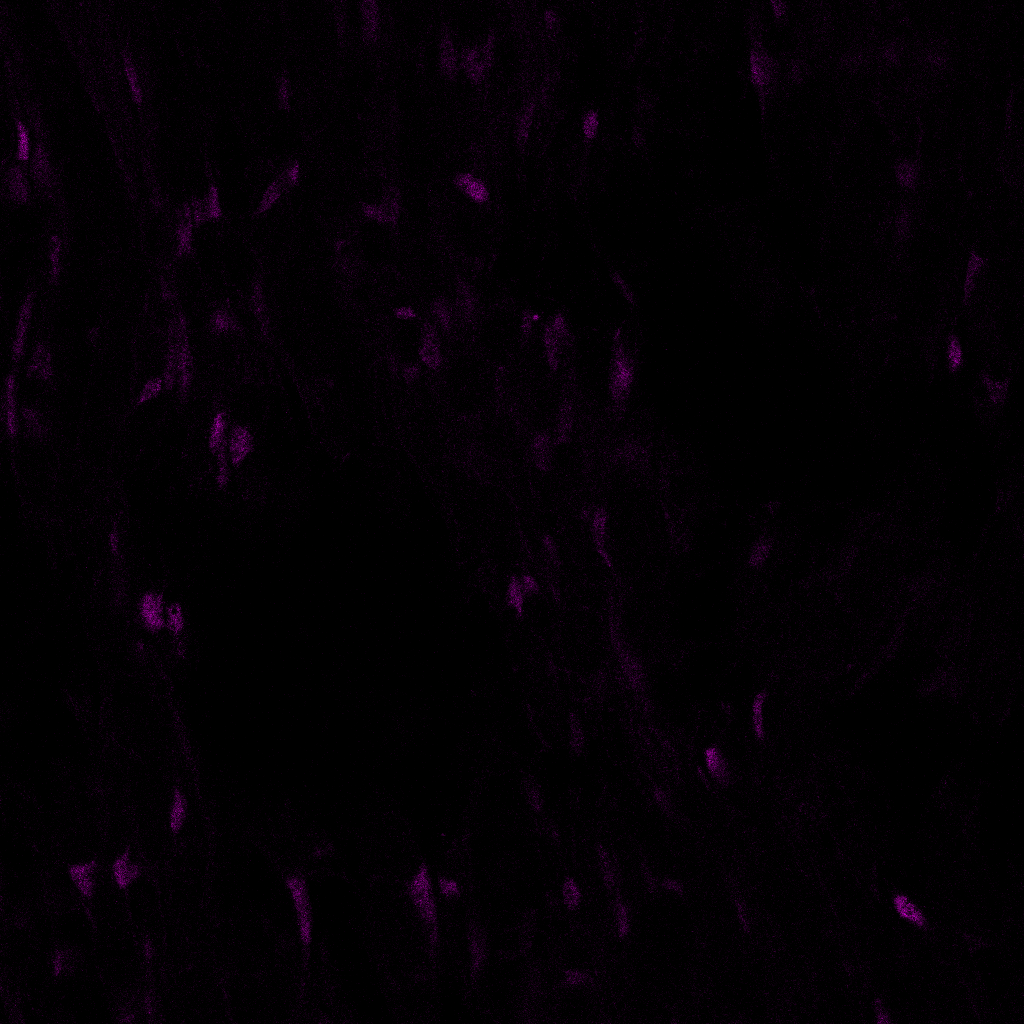

Supplement: Supplementary file 14 — Source Data for Figure 4 [file EMMM-15-e17907-s014.zip › SourceData_Fig_4/Fig_4_SourceData_images/3H/ABT_II_aged_12_dpi_no_reconex_cjun_19.lif_Series001/ABT_II_aged_12_dpi_no_reconex_cjun_19.lif_Series001_z06_ch02.tif]

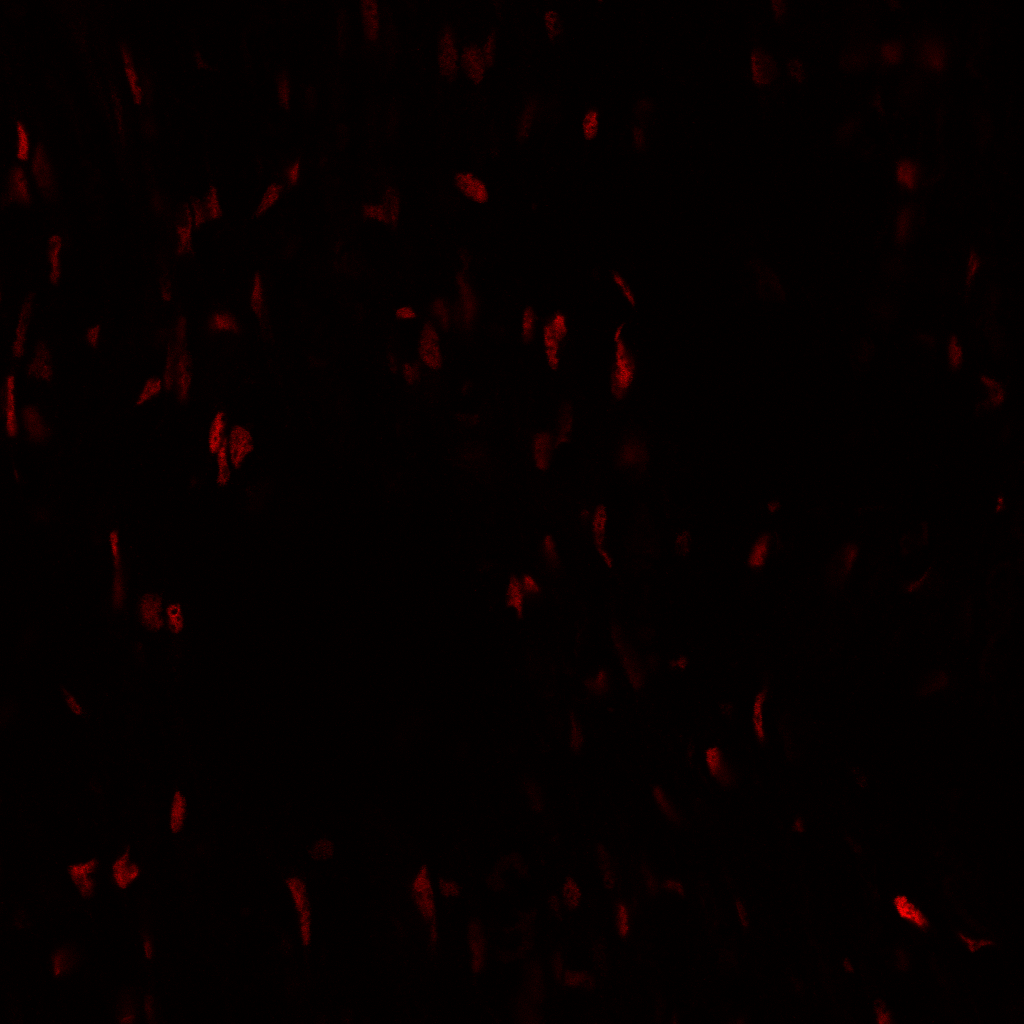

Supplement: Supplementary file 14 — Source Data for Figure 4 [file EMMM-15-e17907-s014.zip › SourceData_Fig_4/Fig_4_SourceData_images/3H/ABT_II_aged_12_dpi_no_reconex_cjun_19.lif_Series001/ABT_II_aged_12_dpi_no_reconex_cjun_19.lif_Series001_z06_ch03.tif]

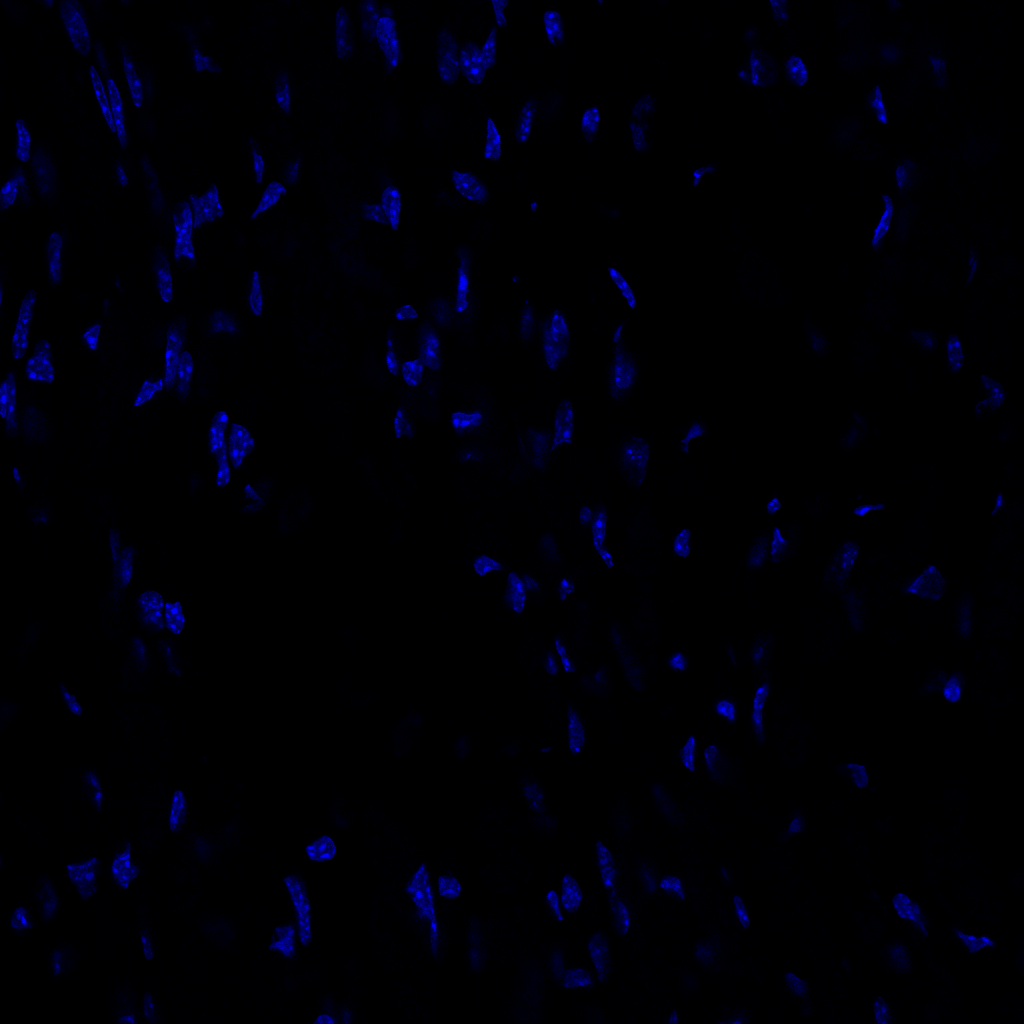

Supplement: Supplementary file 14 — Source Data for Figure 4 [file EMMM-15-e17907-s014.zip › SourceData_Fig_4/Fig_4_SourceData_images/3H/ABT_II_aged_12_dpi_no_reconex_cjun_19.lif_Series001/ABT_II_aged_12_dpi_no_reconex_cjun_19.lif_Series001_z07_ch00.tif]

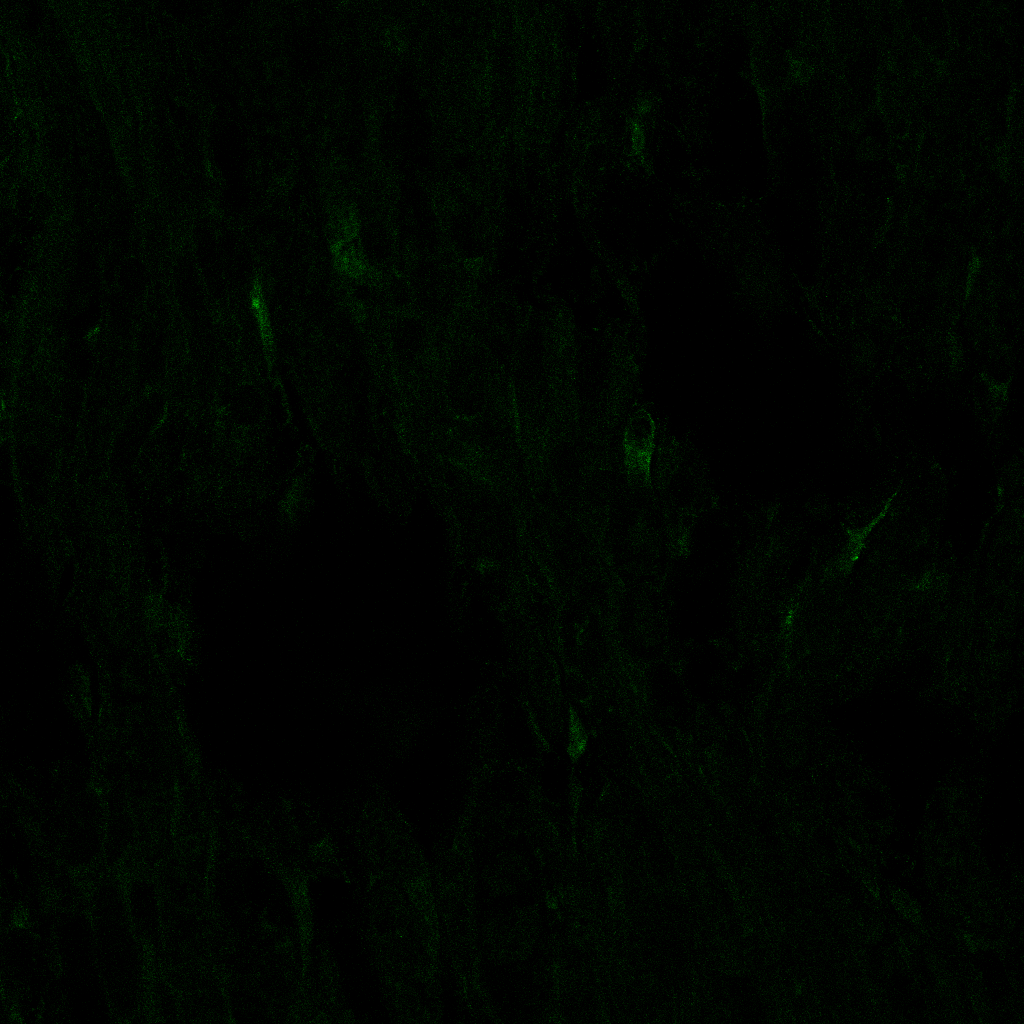

Supplement: Supplementary file 14 — Source Data for Figure 4 [file EMMM-15-e17907-s014.zip › SourceData_Fig_4/Fig_4_SourceData_images/3H/ABT_II_aged_12_dpi_no_reconex_cjun_19.lif_Series001/ABT_II_aged_12_dpi_no_reconex_cjun_19.lif_Series001_z07_ch01.tif]

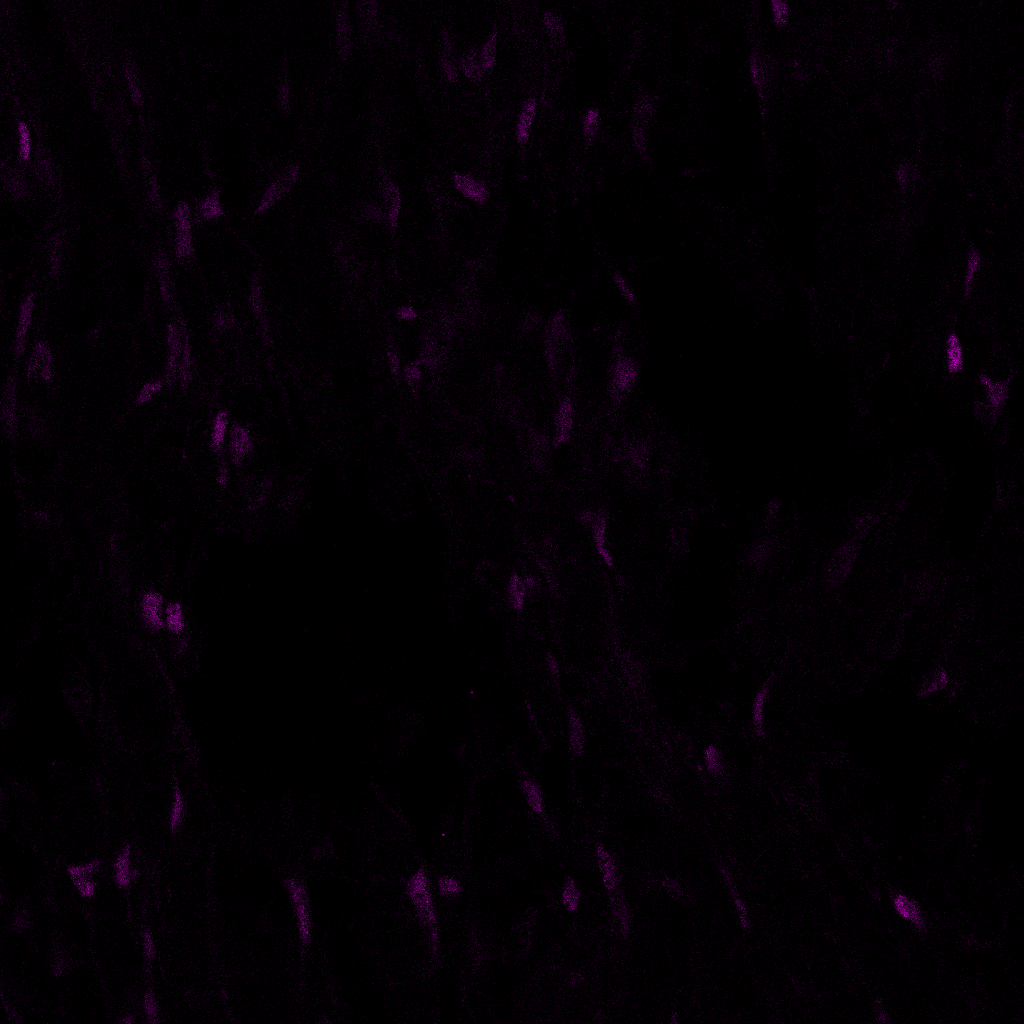

Supplement: Supplementary file 14 — Source Data for Figure 4 [file EMMM-15-e17907-s014.zip › SourceData_Fig_4/Fig_4_SourceData_images/3H/ABT_II_aged_12_dpi_no_reconex_cjun_19.lif_Series001/ABT_II_aged_12_dpi_no_reconex_cjun_19.lif_Series001_z07_ch02.tif]

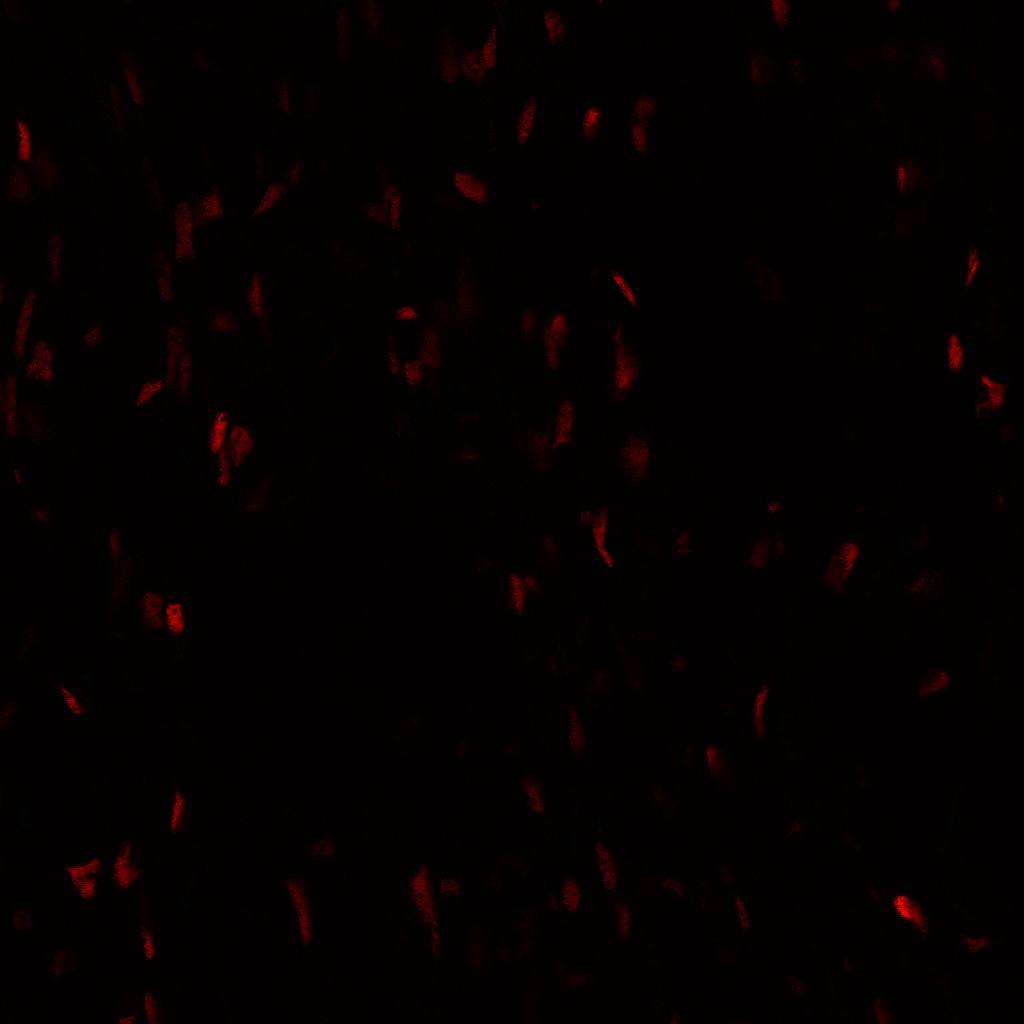

Supplement: Supplementary file 14 — Source Data for Figure 4 [file EMMM-15-e17907-s014.zip › SourceData_Fig_4/Fig_4_SourceData_images/3H/ABT_II_aged_12_dpi_no_reconex_cjun_19.lif_Series001/ABT_II_aged_12_dpi_no_reconex_cjun_19.lif_Series001_z07_ch03.tif]

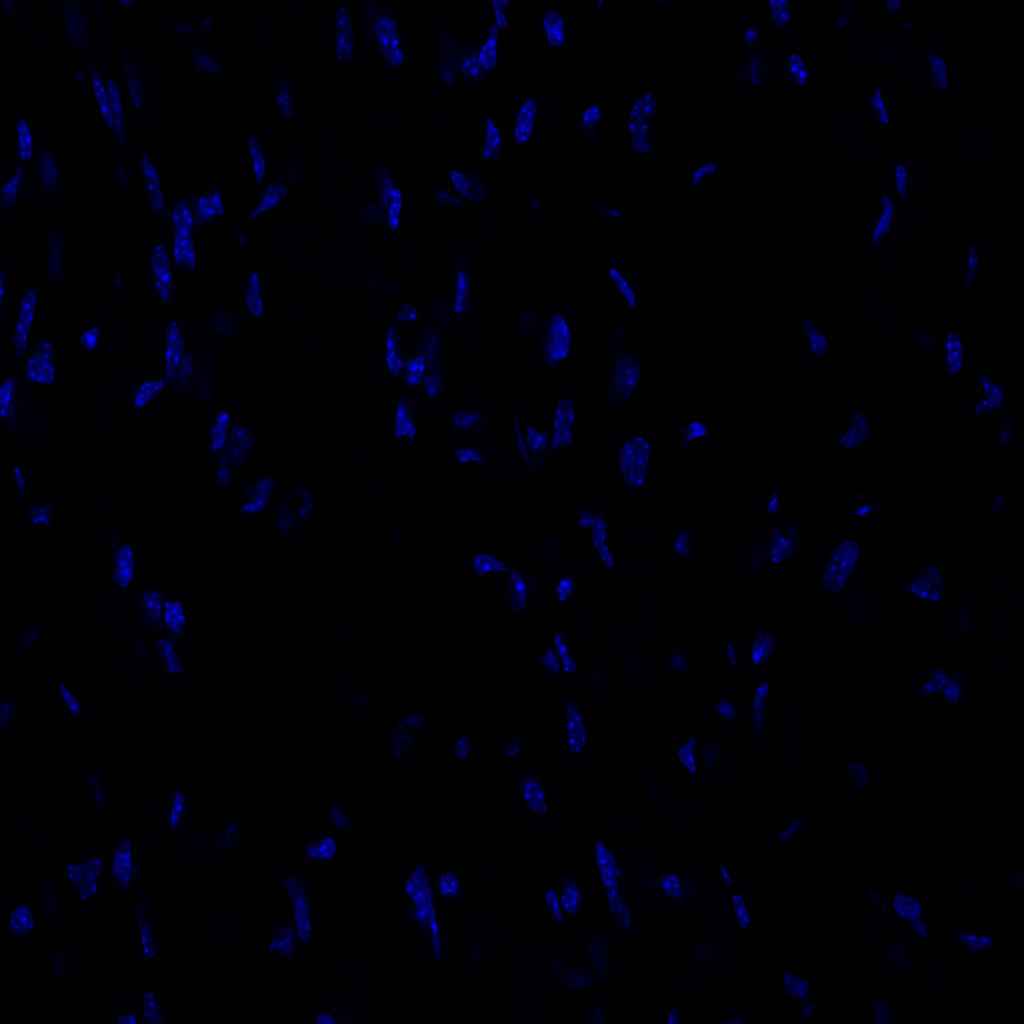

Supplement: Supplementary file 14 — Source Data for Figure 4 [file EMMM-15-e17907-s014.zip › SourceData_Fig_4/Fig_4_SourceData_images/3H/ABT_II_aged_12_dpi_no_reconex_cjun_19.lif_Series001/ABT_II_aged_12_dpi_no_reconex_cjun_19.lif_Series001_z08_ch00.tif]

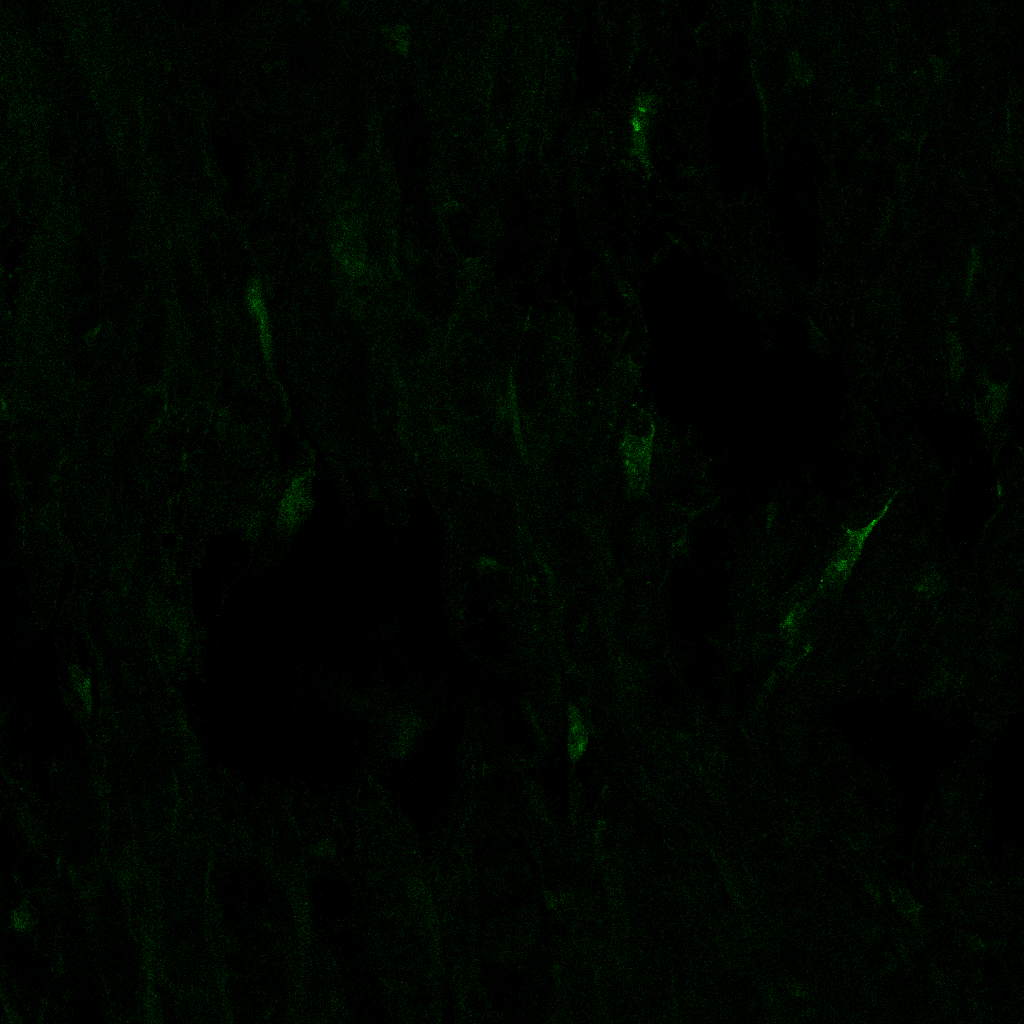

Supplement: Supplementary file 14 — Source Data for Figure 4 [file EMMM-15-e17907-s014.zip › SourceData_Fig_4/Fig_4_SourceData_images/3H/ABT_II_aged_12_dpi_no_reconex_cjun_19.lif_Series001/ABT_II_aged_12_dpi_no_reconex_cjun_19.lif_Series001_z08_ch01.tif]

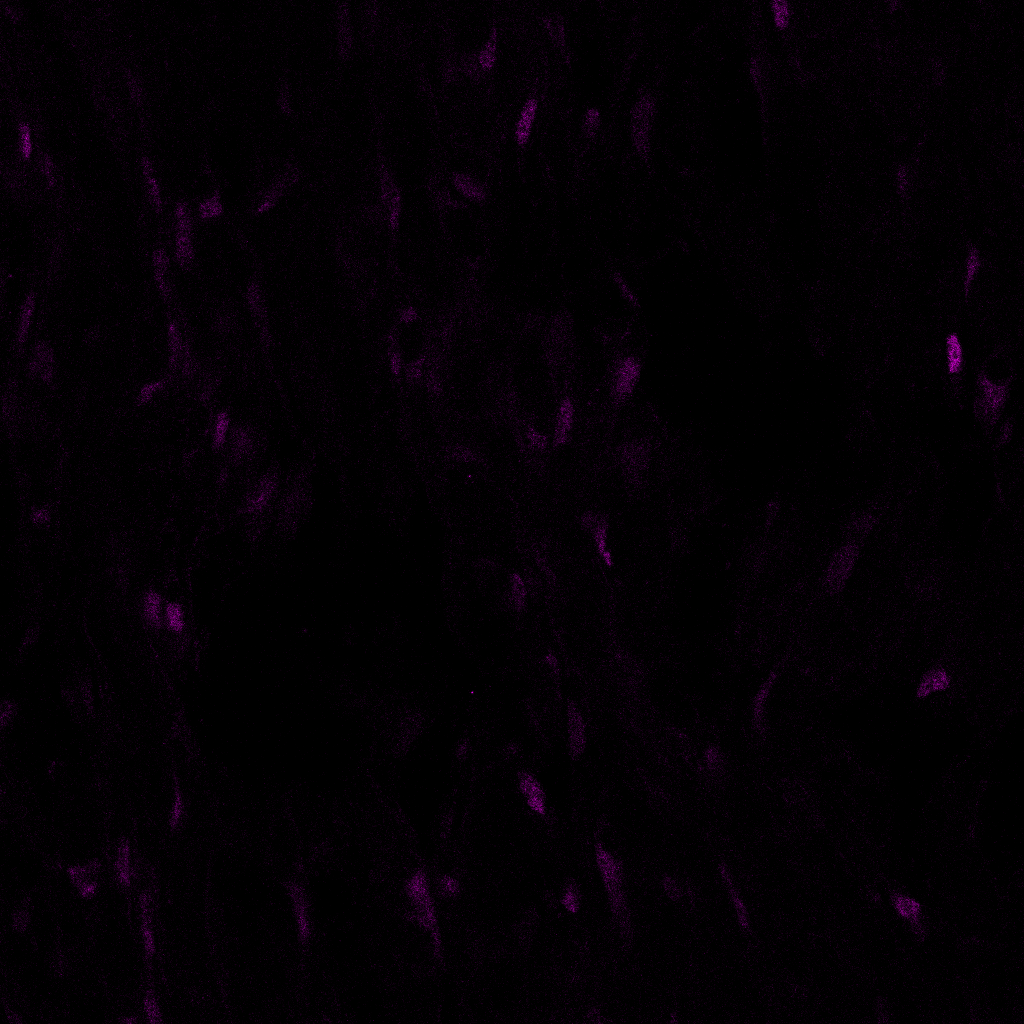

Supplement: Supplementary file 14 — Source Data for Figure 4 [file EMMM-15-e17907-s014.zip › SourceData_Fig_4/Fig_4_SourceData_images/3H/ABT_II_aged_12_dpi_no_reconex_cjun_19.lif_Series001/ABT_II_aged_12_dpi_no_reconex_cjun_19.lif_Series001_z08_ch02.tif]

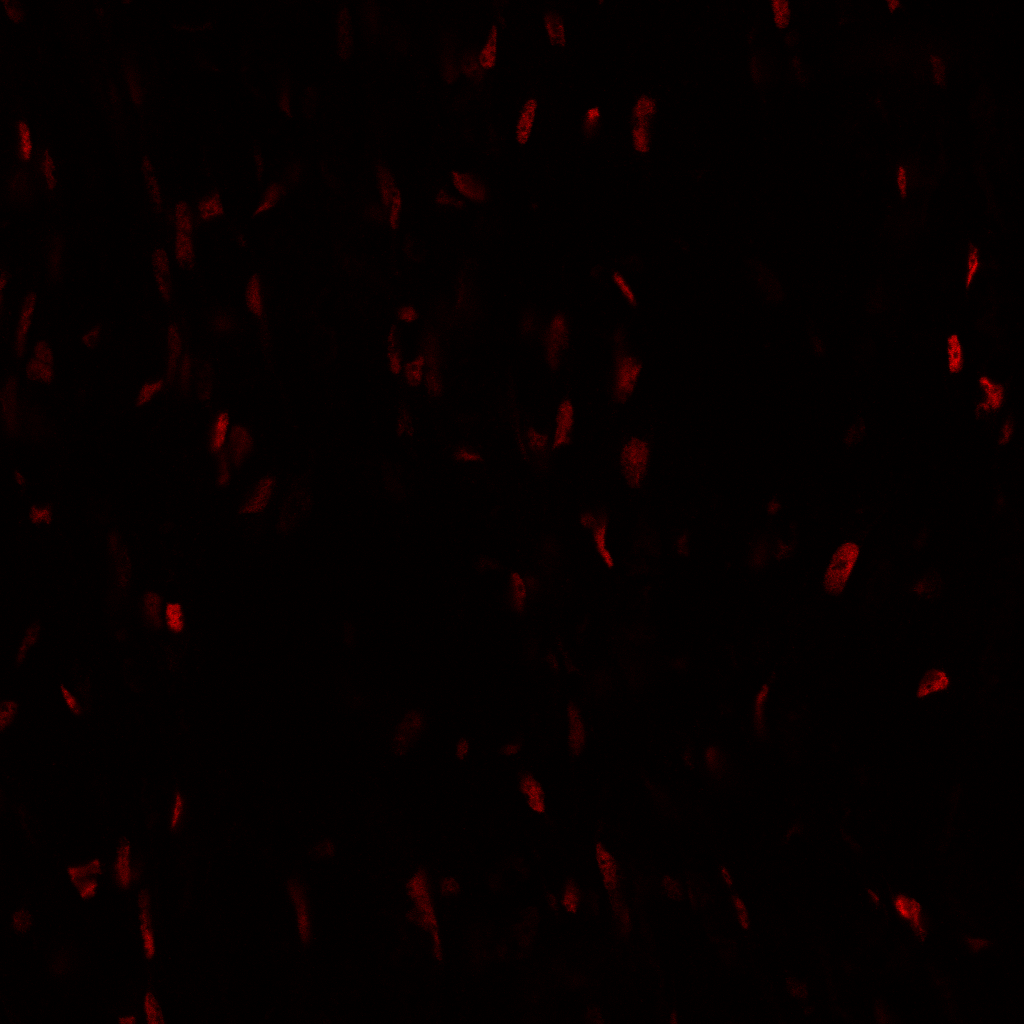

Supplement: Supplementary file 14 — Source Data for Figure 4 [file EMMM-15-e17907-s014.zip › SourceData_Fig_4/Fig_4_SourceData_images/3H/ABT_II_aged_12_dpi_no_reconex_cjun_19.lif_Series001/ABT_II_aged_12_dpi_no_reconex_cjun_19.lif_Series001_z08_ch03.tif]

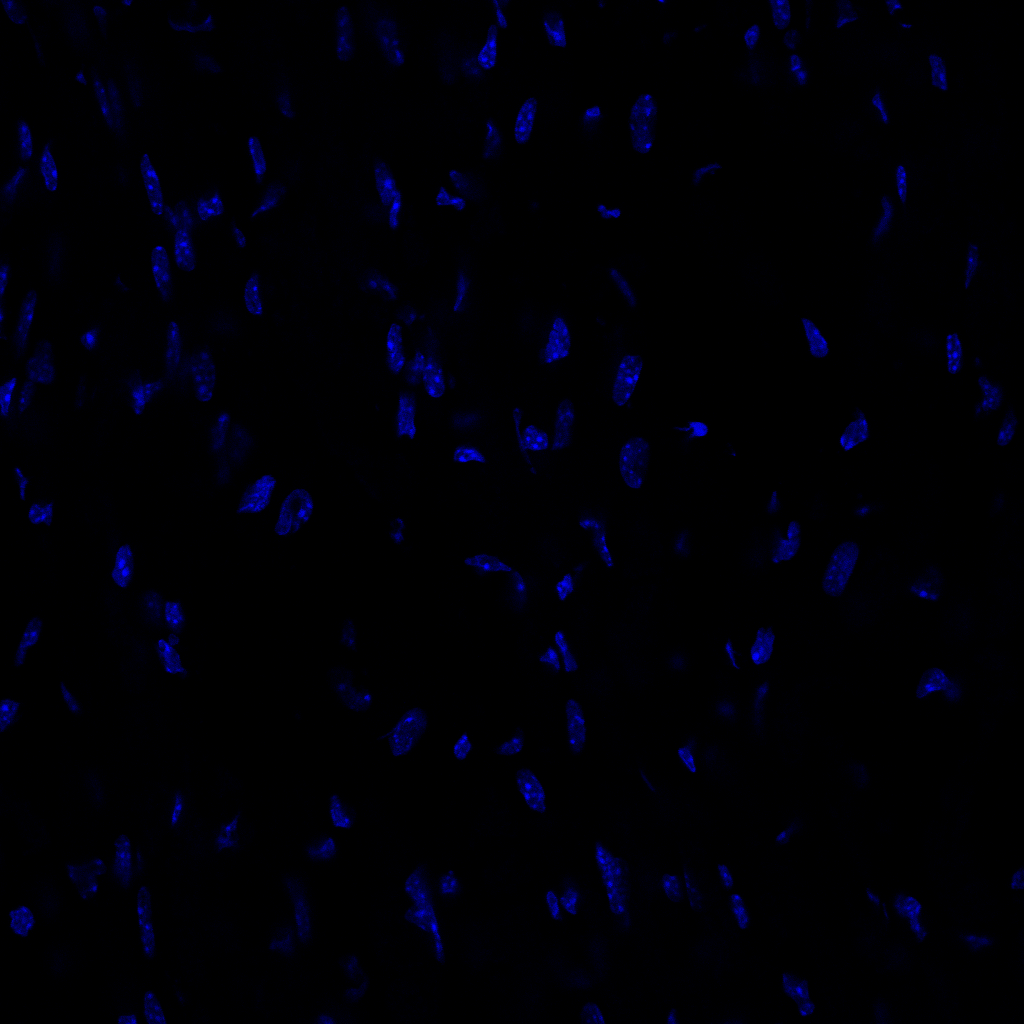

Supplement: Supplementary file 14 — Source Data for Figure 4 [file EMMM-15-e17907-s014.zip › SourceData_Fig_4/Fig_4_SourceData_images/3H/ABT_II_aged_12_dpi_no_reconex_cjun_19.lif_Series001/ABT_II_aged_12_dpi_no_reconex_cjun_19.lif_Series001_z09_ch00.tif]

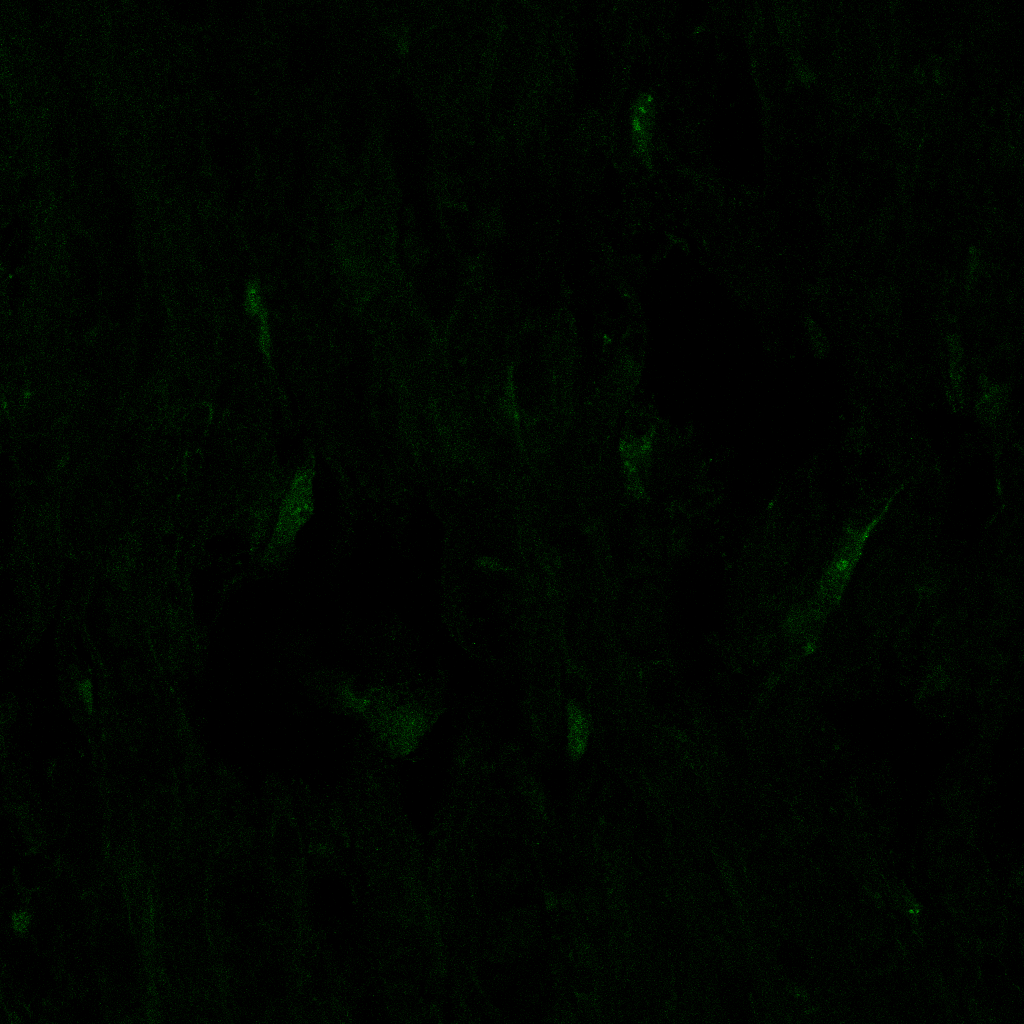

Supplement: Supplementary file 14 — Source Data for Figure 4 [file EMMM-15-e17907-s014.zip › SourceData_Fig_4/Fig_4_SourceData_images/3H/ABT_II_aged_12_dpi_no_reconex_cjun_19.lif_Series001/ABT_II_aged_12_dpi_no_reconex_cjun_19.lif_Series001_z09_ch01.tif]

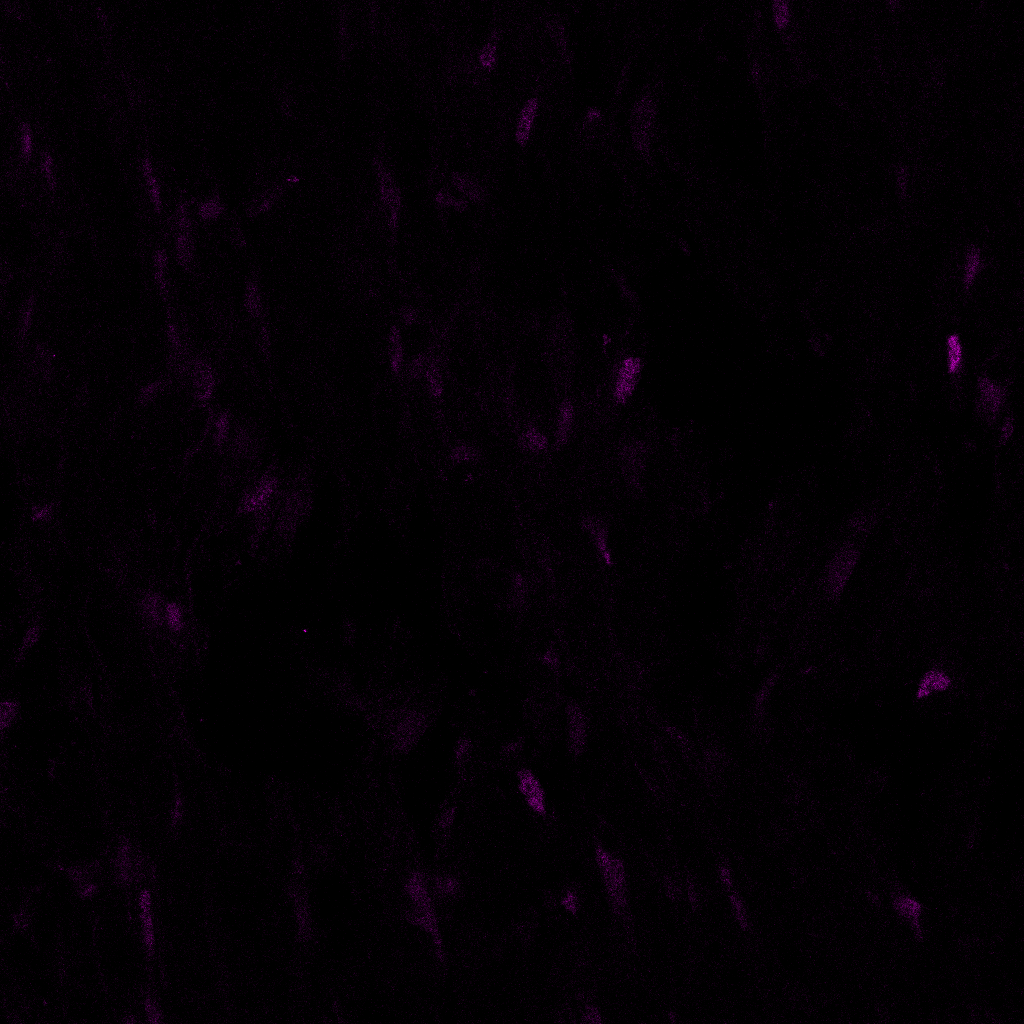

Supplement: Supplementary file 14 — Source Data for Figure 4 [file EMMM-15-e17907-s014.zip › SourceData_Fig_4/Fig_4_SourceData_images/3H/ABT_II_aged_12_dpi_no_reconex_cjun_19.lif_Series001/ABT_II_aged_12_dpi_no_reconex_cjun_19.lif_Series001_z09_ch02.tif]

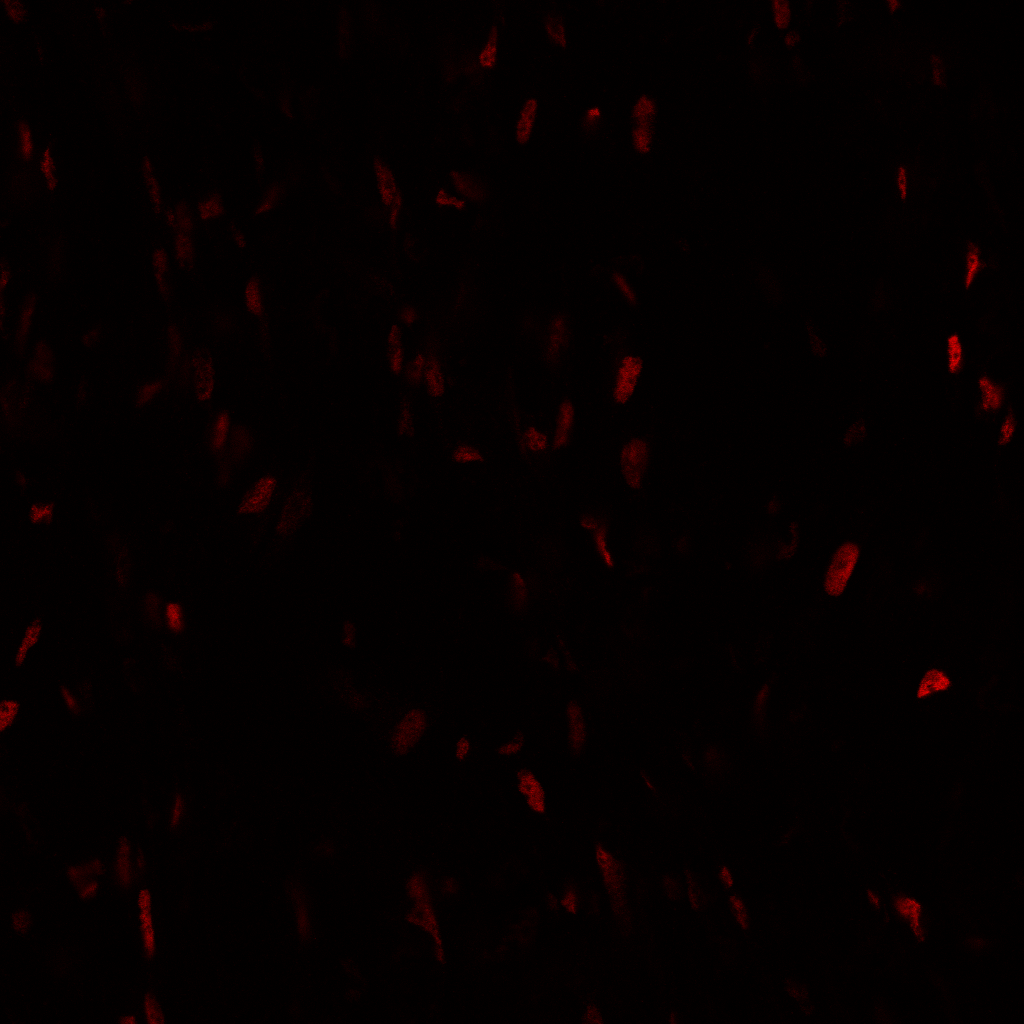

Supplement: Supplementary file 14 — Source Data for Figure 4 [file EMMM-15-e17907-s014.zip › SourceData_Fig_4/Fig_4_SourceData_images/3H/ABT_II_aged_12_dpi_no_reconex_cjun_19.lif_Series001/ABT_II_aged_12_dpi_no_reconex_cjun_19.lif_Series001_z09_ch03.tif]

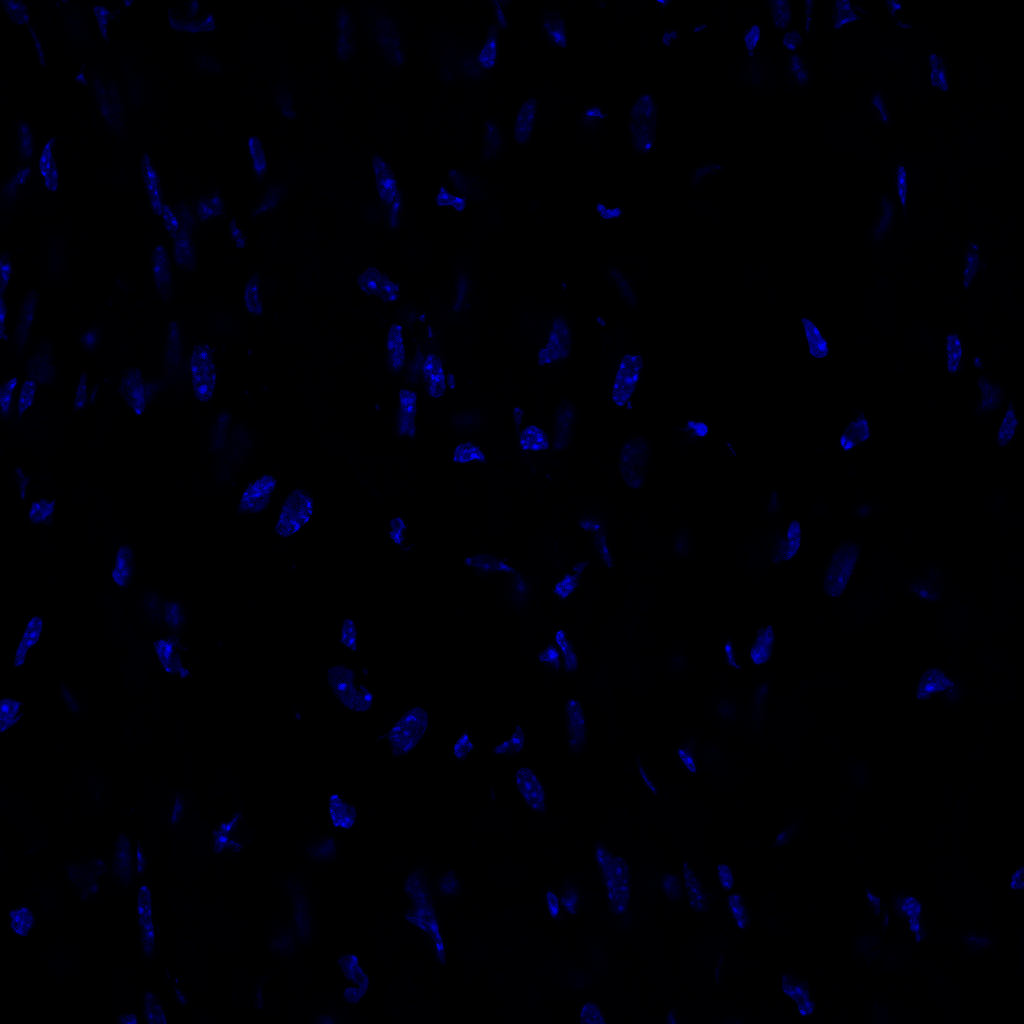

Supplement: Supplementary file 14 — Source Data for Figure 4 [file EMMM-15-e17907-s014.zip › SourceData_Fig_4/Fig_4_SourceData_images/3H/ABT_II_aged_12_dpi_no_reconex_cjun_19.lif_Series001/ABT_II_aged_12_dpi_no_reconex_cjun_19.lif_Series001_z10_ch00.tif]

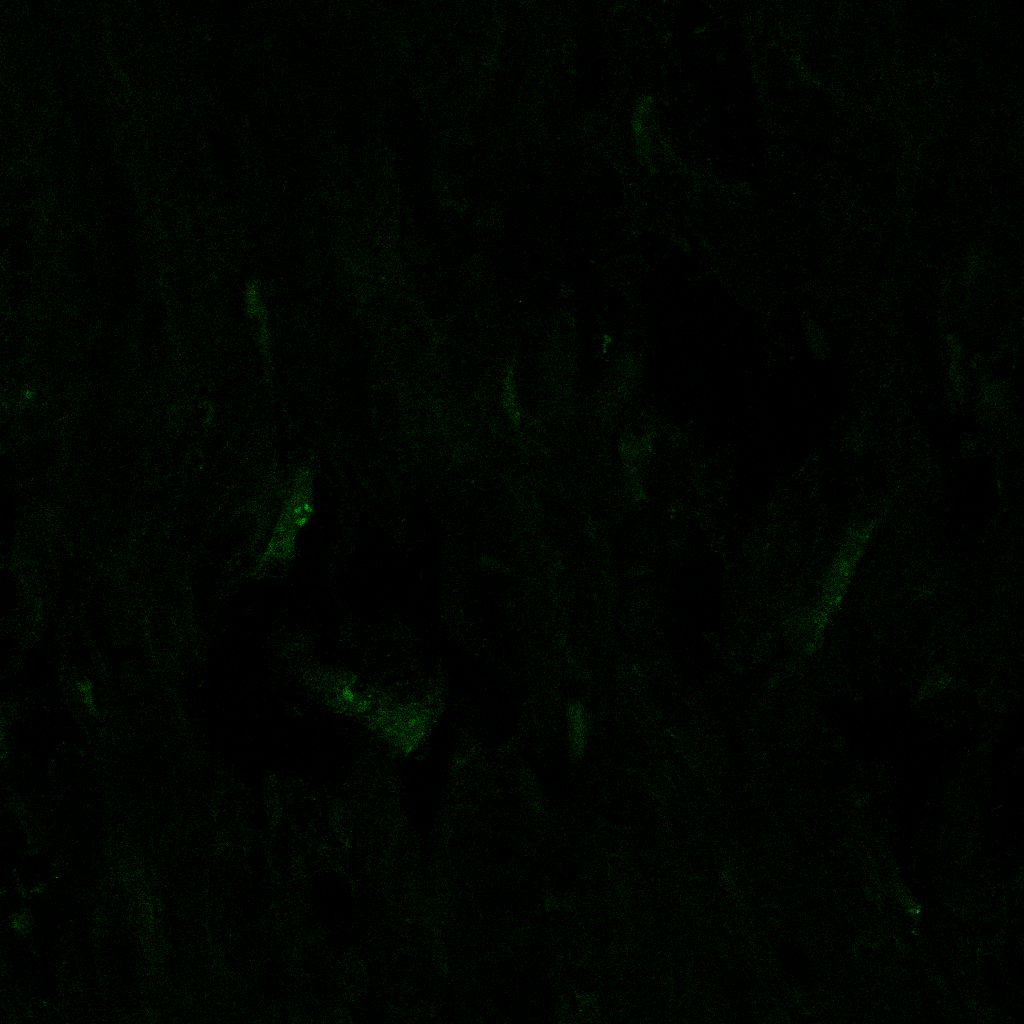

Supplement: Supplementary file 14 — Source Data for Figure 4 [file EMMM-15-e17907-s014.zip › SourceData_Fig_4/Fig_4_SourceData_images/3H/ABT_II_aged_12_dpi_no_reconex_cjun_19.lif_Series001/ABT_II_aged_12_dpi_no_reconex_cjun_19.lif_Series001_z10_ch01.tif]

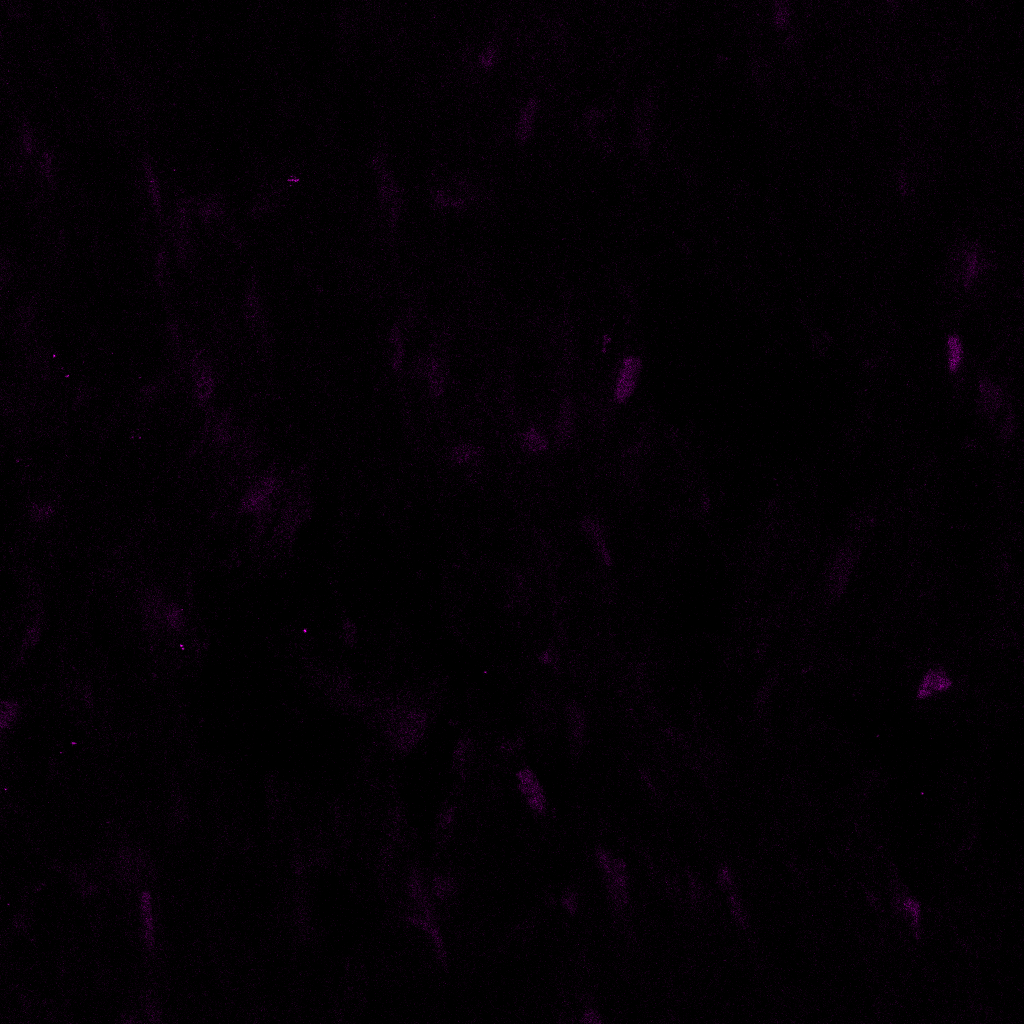

Supplement: Supplementary file 14 — Source Data for Figure 4 [file EMMM-15-e17907-s014.zip › SourceData_Fig_4/Fig_4_SourceData_images/3H/ABT_II_aged_12_dpi_no_reconex_cjun_19.lif_Series001/ABT_II_aged_12_dpi_no_reconex_cjun_19.lif_Series001_z10_ch02.tif]

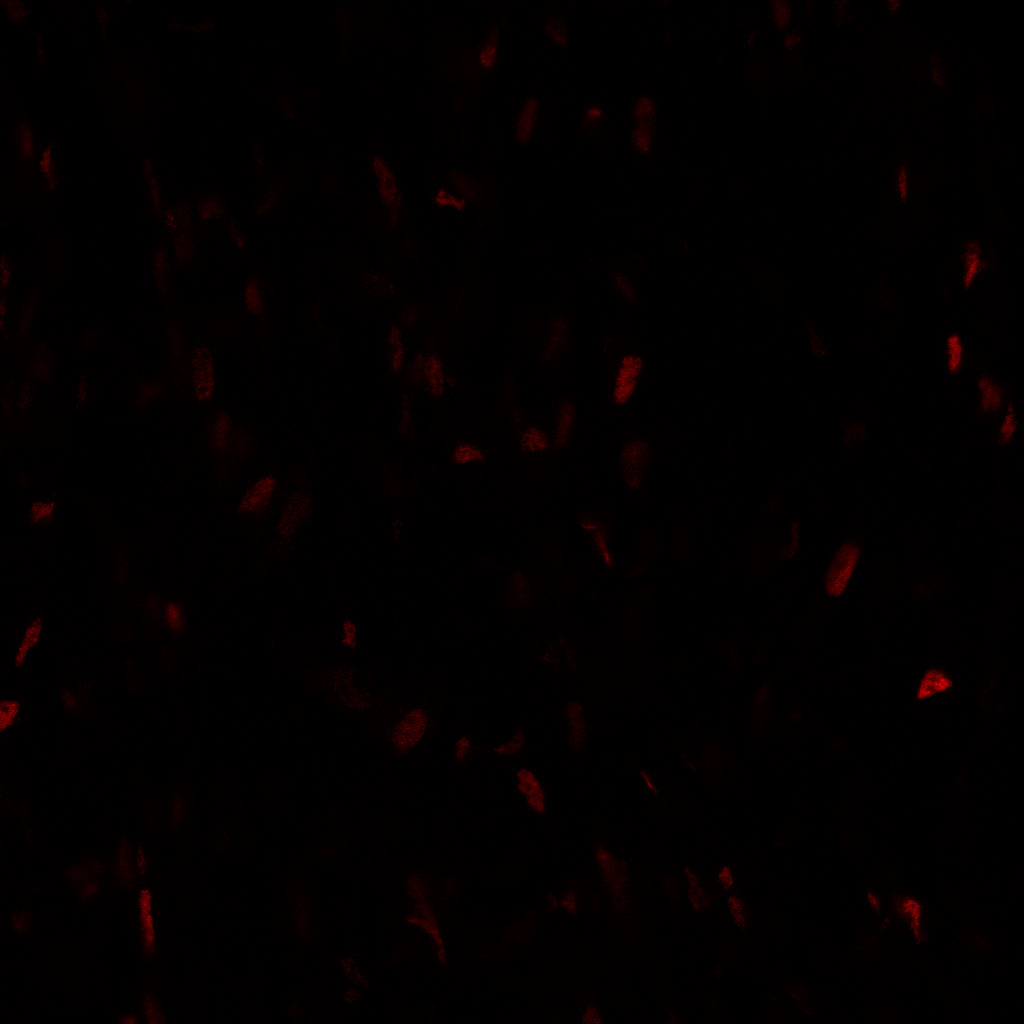

Supplement: Supplementary file 14 — Source Data for Figure 4 [file EMMM-15-e17907-s014.zip › SourceData_Fig_4/Fig_4_SourceData_images/3H/ABT_II_aged_12_dpi_no_reconex_cjun_19.lif_Series001/ABT_II_aged_12_dpi_no_reconex_cjun_19.lif_Series001_z10_ch03.tif]

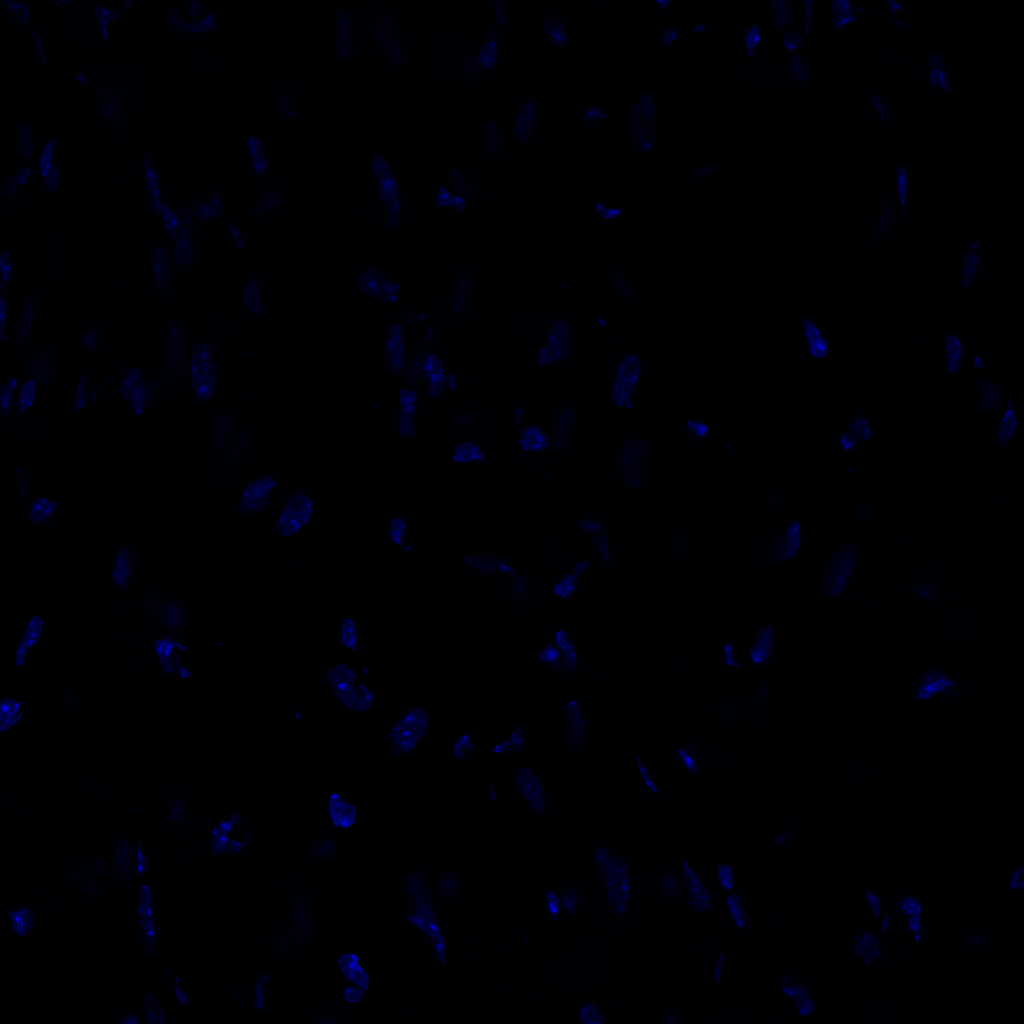

Supplement: Supplementary file 14 — Source Data for Figure 4 [file EMMM-15-e17907-s014.zip › SourceData_Fig_4/Fig_4_SourceData_images/3H/ABT_II_aged_12_dpi_no_reconex_cjun_19.lif_Series001/ABT_II_aged_12_dpi_no_reconex_cjun_19.lif_Series001_z11_ch00.tif]

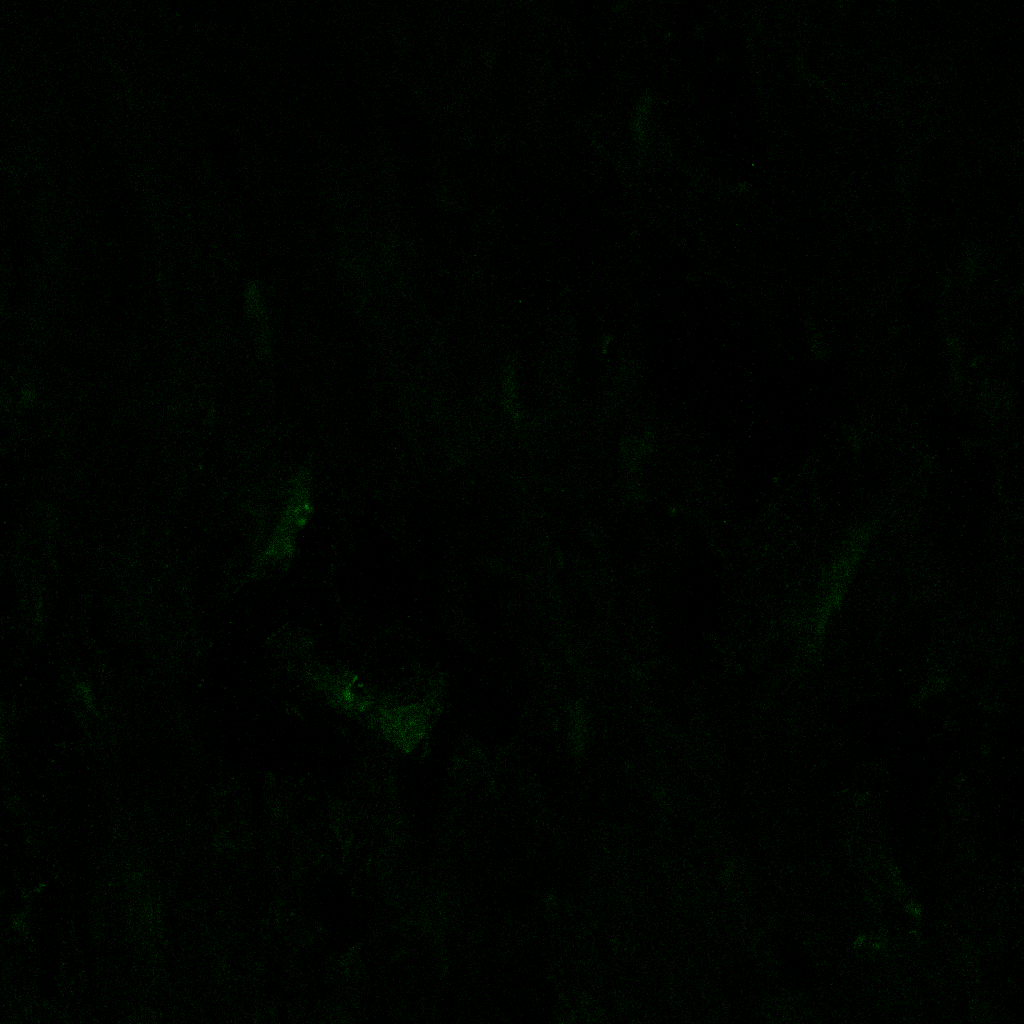

Supplement: Supplementary file 14 — Source Data for Figure 4 [file EMMM-15-e17907-s014.zip › SourceData_Fig_4/Fig_4_SourceData_images/3H/ABT_II_aged_12_dpi_no_reconex_cjun_19.lif_Series001/ABT_II_aged_12_dpi_no_reconex_cjun_19.lif_Series001_z11_ch01.tif]

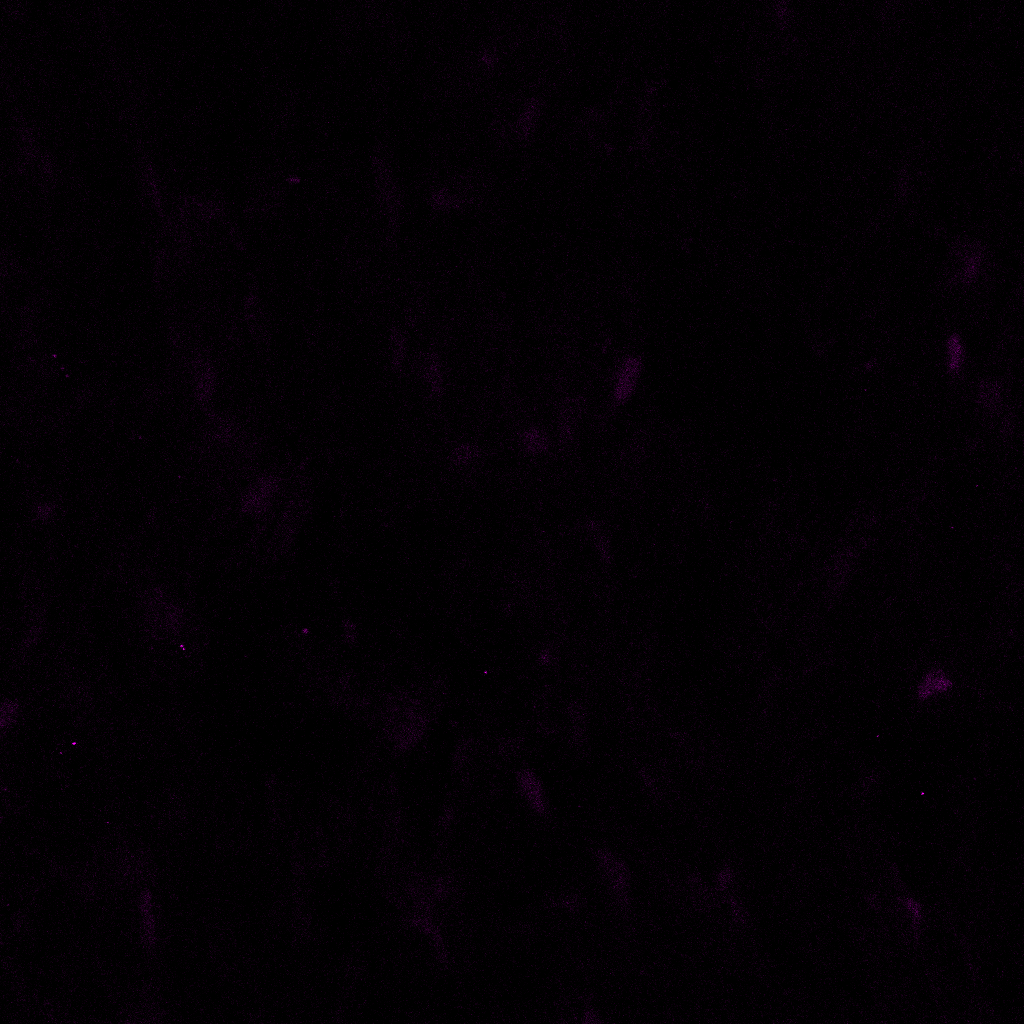

Supplement: Supplementary file 14 — Source Data for Figure 4 [file EMMM-15-e17907-s014.zip › SourceData_Fig_4/Fig_4_SourceData_images/3H/ABT_II_aged_12_dpi_no_reconex_cjun_19.lif_Series001/ABT_II_aged_12_dpi_no_reconex_cjun_19.lif_Series001_z11_ch02.tif]

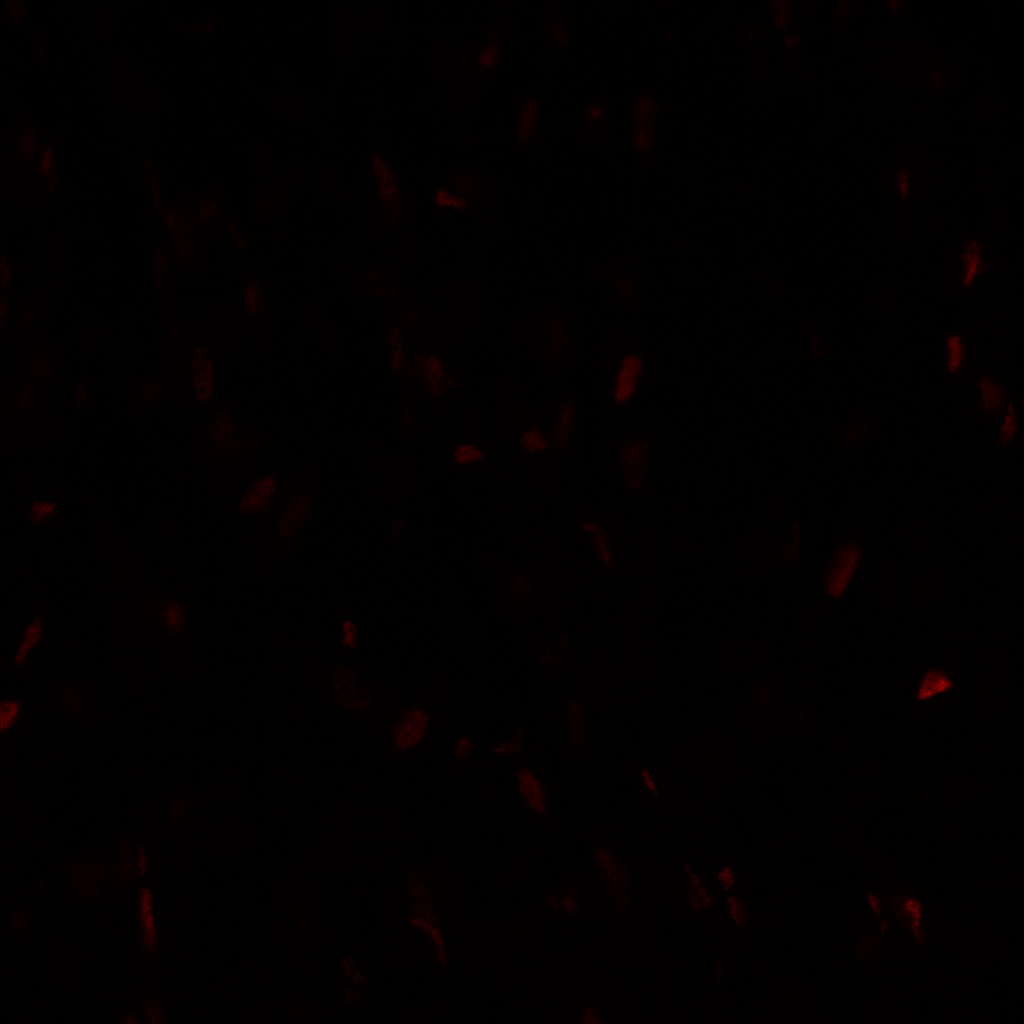

Supplement: Supplementary file 14 — Source Data for Figure 4 [file EMMM-15-e17907-s014.zip › SourceData_Fig_4/Fig_4_SourceData_images/3H/ABT_II_aged_12_dpi_no_reconex_cjun_19.lif_Series001/ABT_II_aged_12_dpi_no_reconex_cjun_19.lif_Series001_z11_ch03.tif]

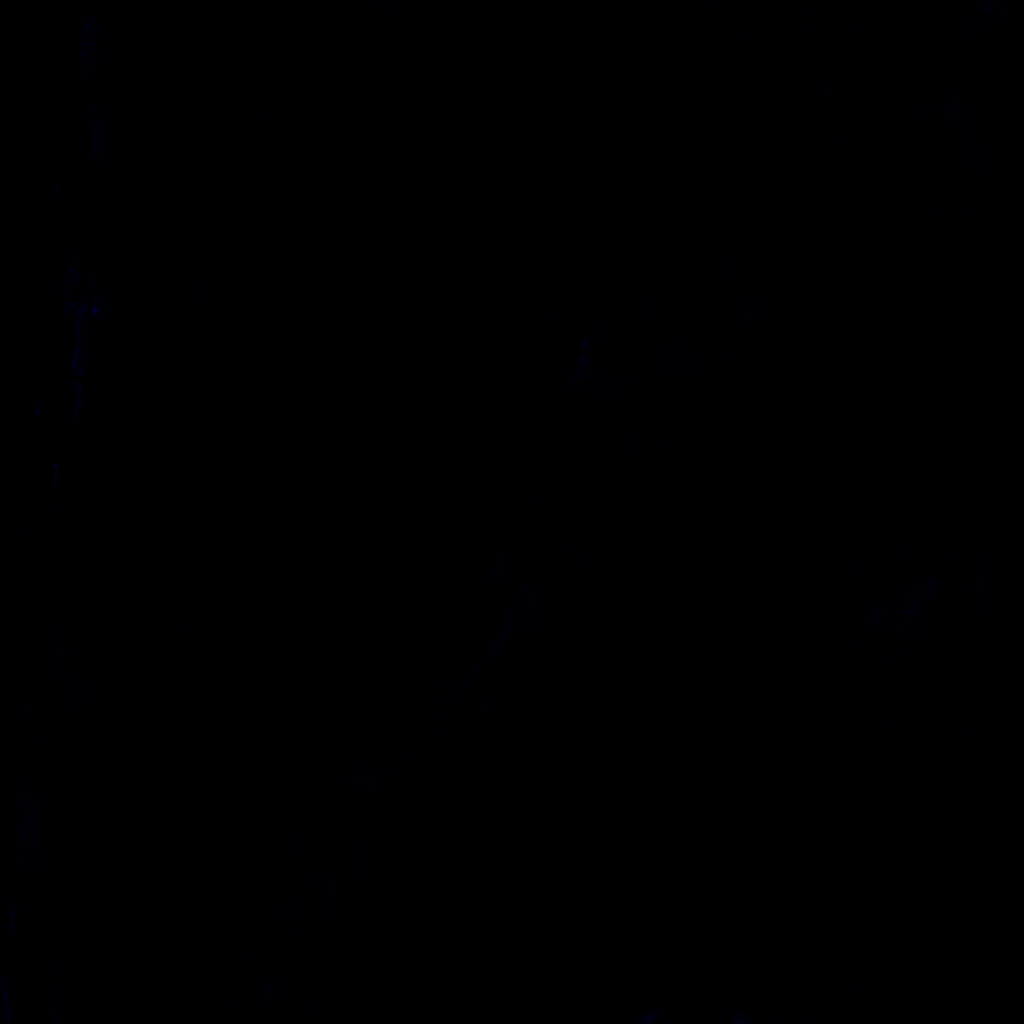

Supplement: Supplementary file 14 — Source Data for Figure 4 [file EMMM-15-e17907-s014.zip › SourceData_Fig_4/Fig_4_SourceData_images/3H/VEH_3_adult_42_dpi_no_reconex_cjun_19.lif_Series001/VEH_3_adult_42_dpi_no_reconex_cjun_19.lif_Series001_z00_ch00.tif]

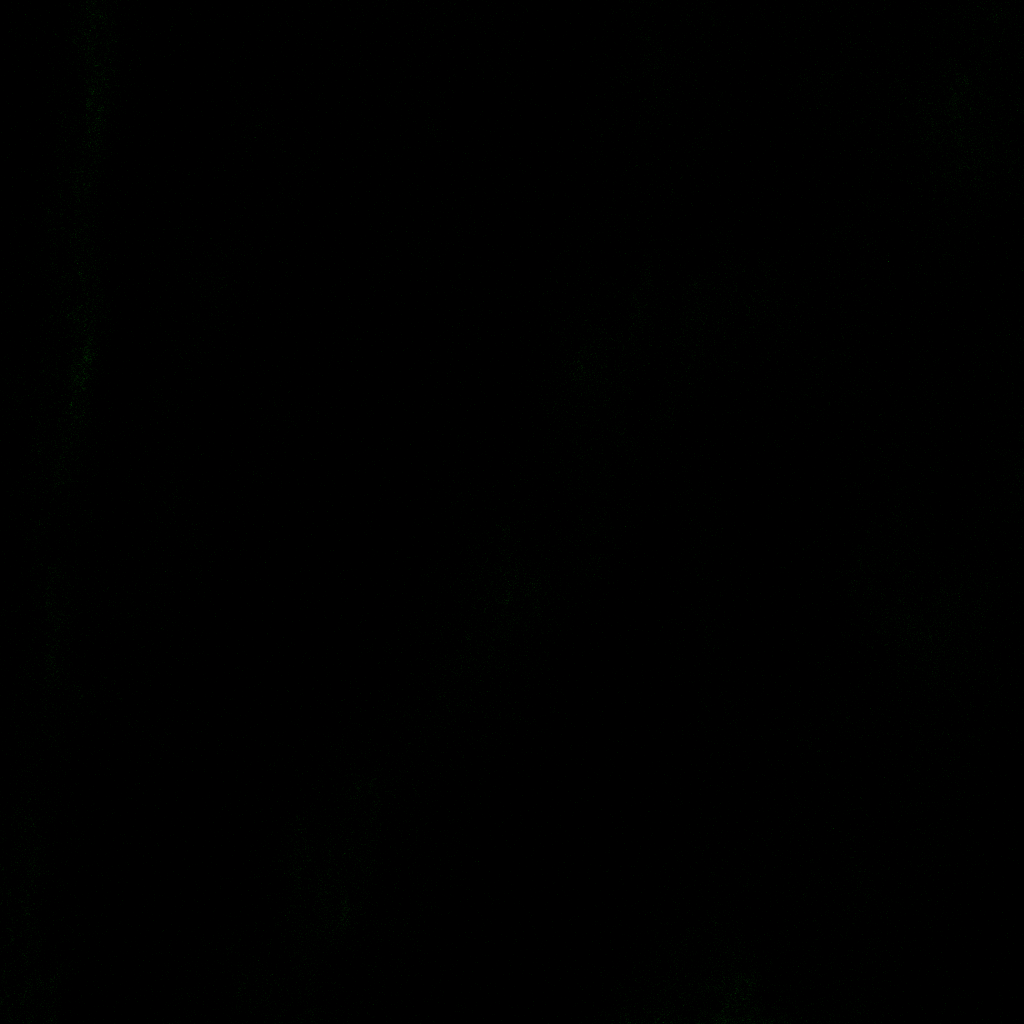

Supplement: Supplementary file 14 — Source Data for Figure 4 [file EMMM-15-e17907-s014.zip › SourceData_Fig_4/Fig_4_SourceData_images/3H/VEH_3_adult_42_dpi_no_reconex_cjun_19.lif_Series001/VEH_3_adult_42_dpi_no_reconex_cjun_19.lif_Series001_z00_ch01.tif]

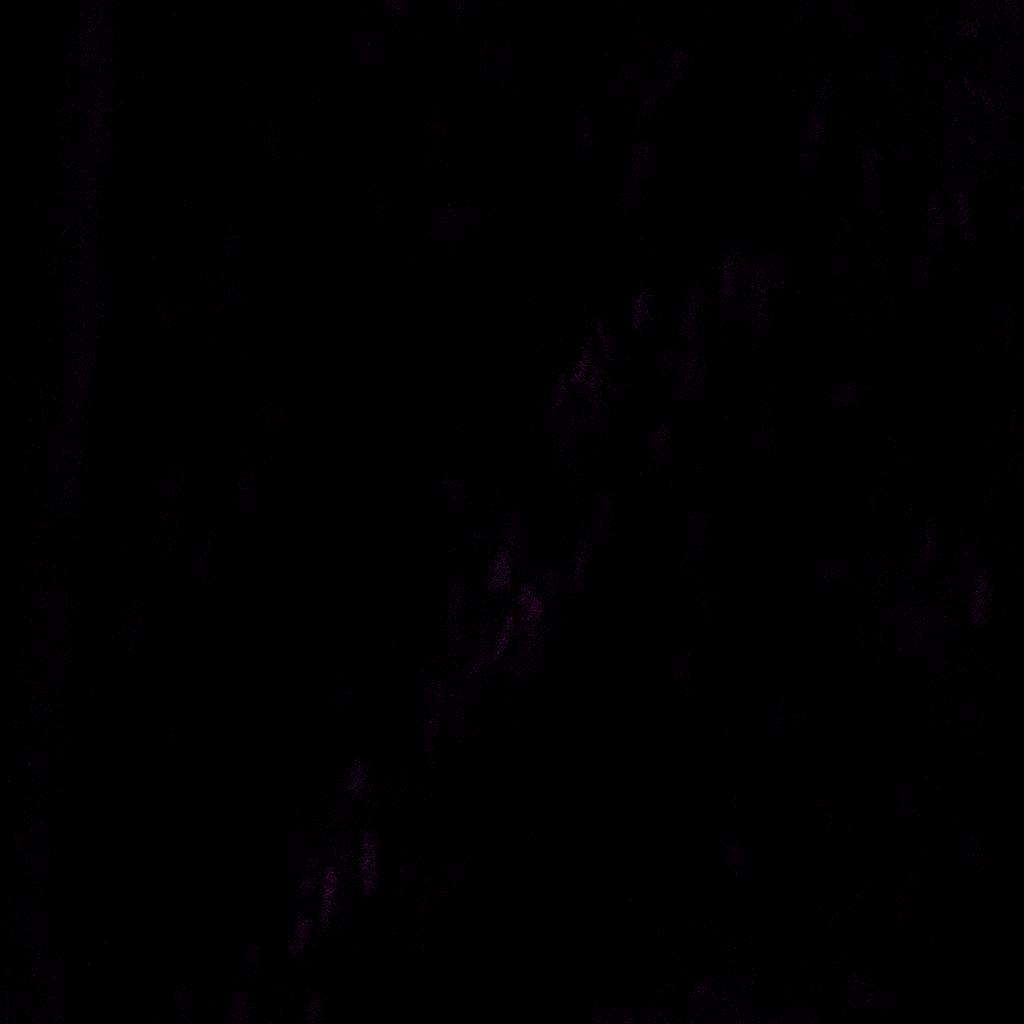

Supplement: Supplementary file 14 — Source Data for Figure 4 [file EMMM-15-e17907-s014.zip › SourceData_Fig_4/Fig_4_SourceData_images/3H/VEH_3_adult_42_dpi_no_reconex_cjun_19.lif_Series001/VEH_3_adult_42_dpi_no_reconex_cjun_19.lif_Series001_z00_ch02.tif]

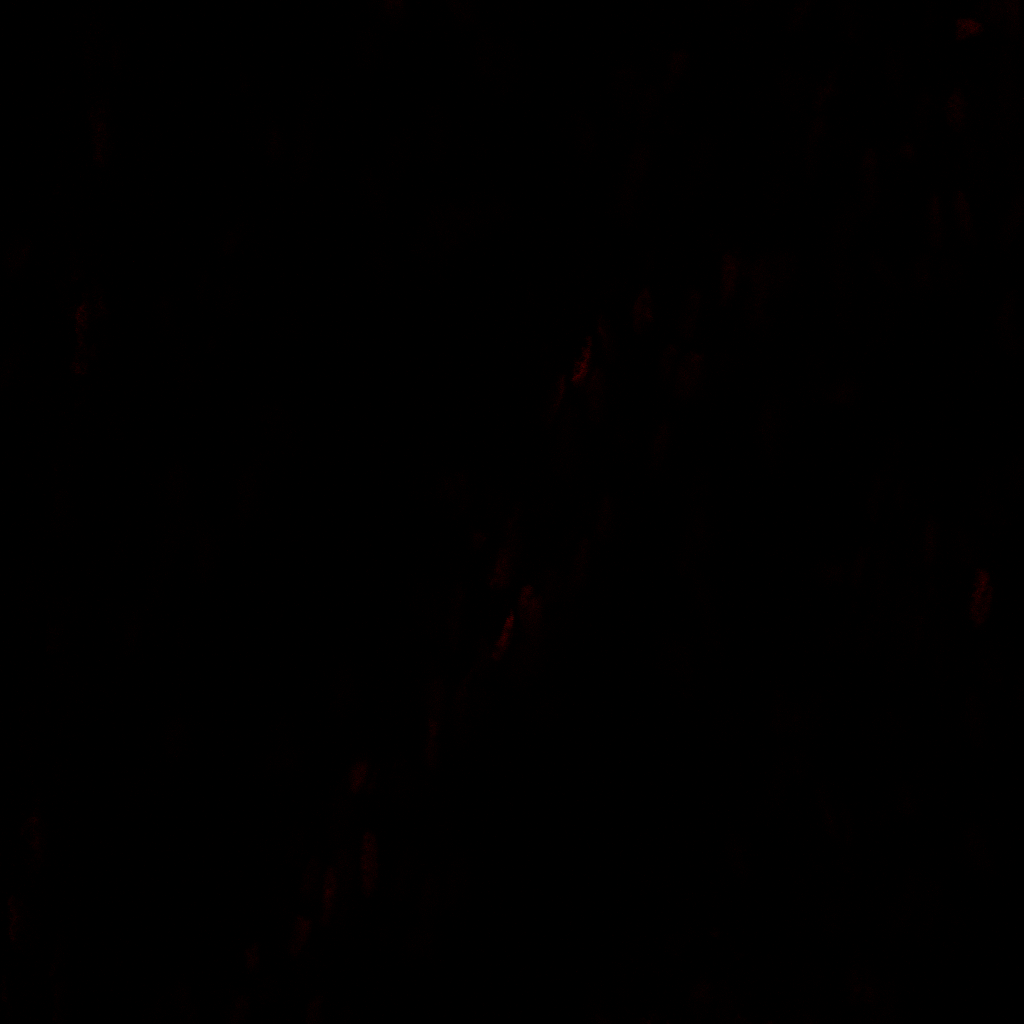

Supplement: Supplementary file 14 — Source Data for Figure 4 [file EMMM-15-e17907-s014.zip › SourceData_Fig_4/Fig_4_SourceData_images/3H/VEH_3_adult_42_dpi_no_reconex_cjun_19.lif_Series001/VEH_3_adult_42_dpi_no_reconex_cjun_19.lif_Series001_z00_ch03.tif]

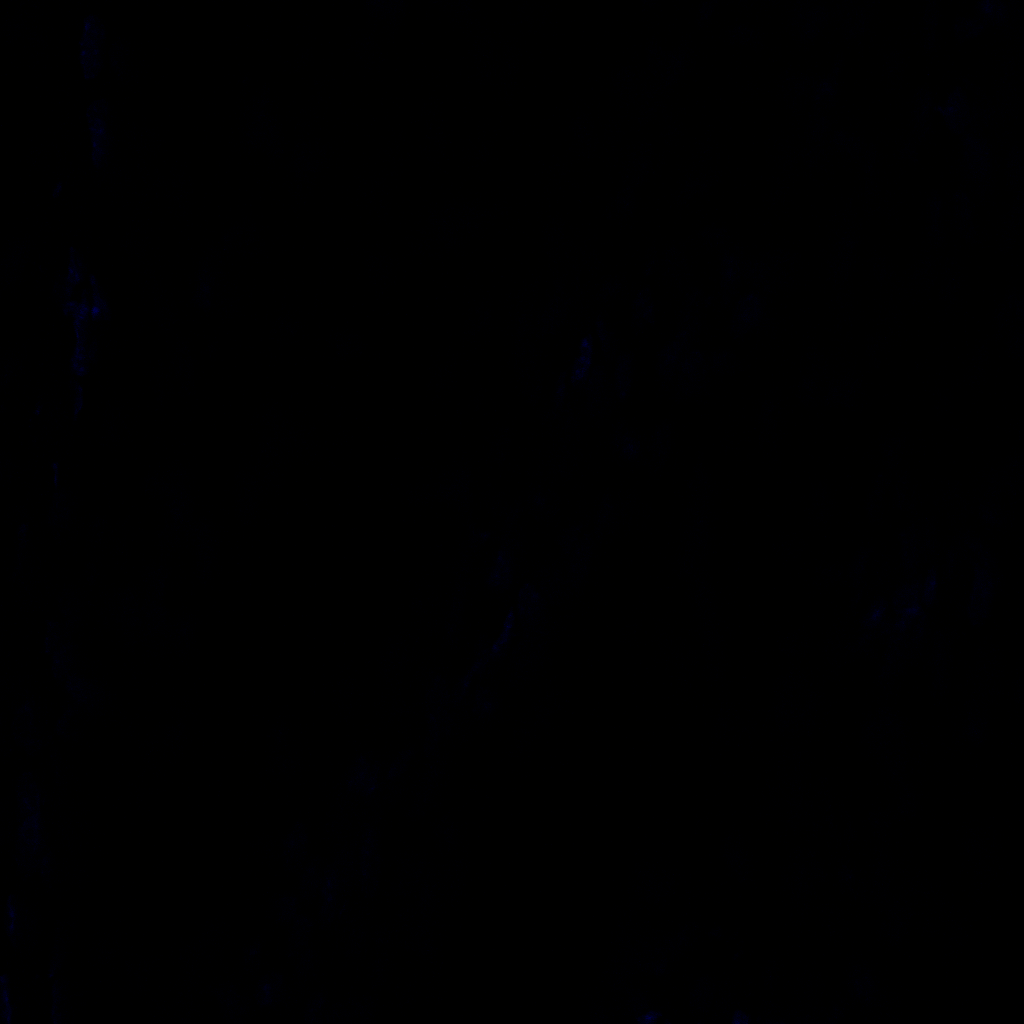

Supplement: Supplementary file 14 — Source Data for Figure 4 [file EMMM-15-e17907-s014.zip › SourceData_Fig_4/Fig_4_SourceData_images/3H/VEH_3_adult_42_dpi_no_reconex_cjun_19.lif_Series001/VEH_3_adult_42_dpi_no_reconex_cjun_19.lif_Series001_z01_ch00.tif]

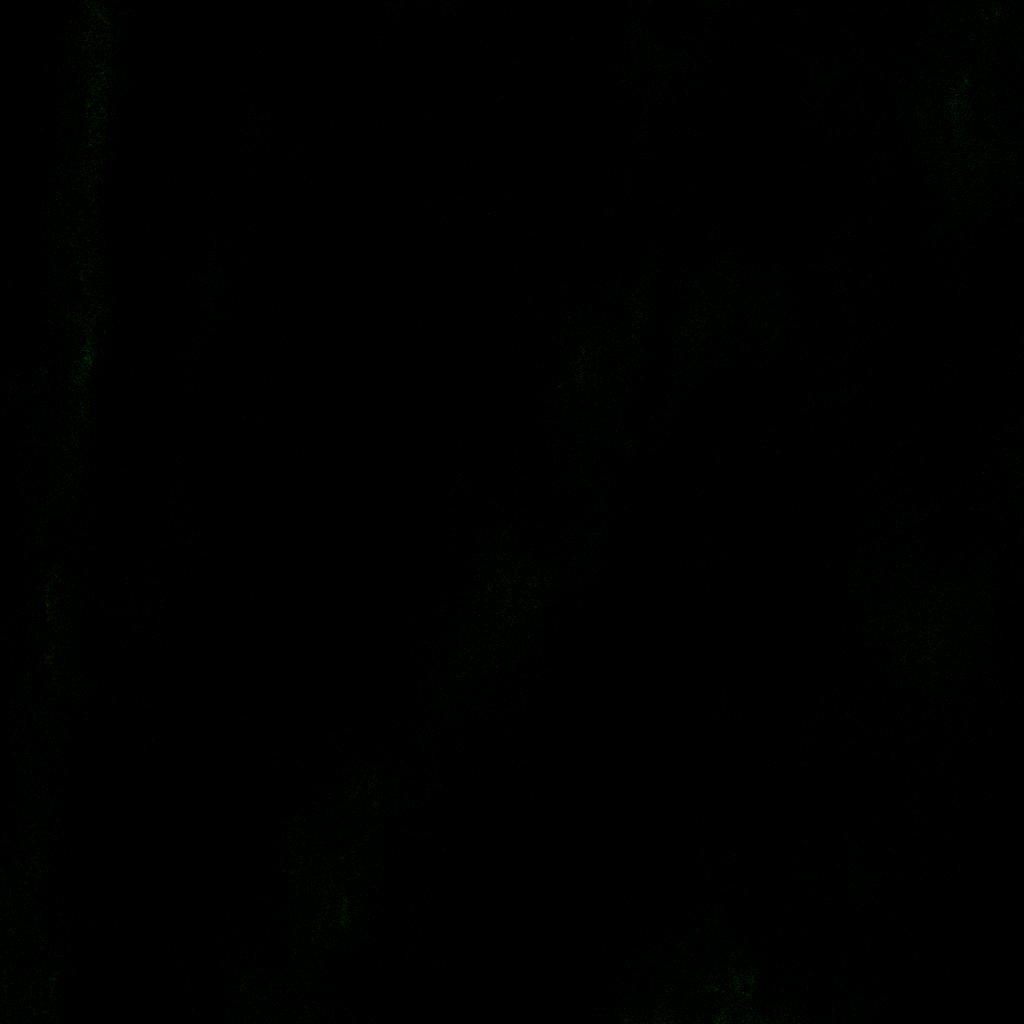

Supplement: Supplementary file 14 — Source Data for Figure 4 [file EMMM-15-e17907-s014.zip › SourceData_Fig_4/Fig_4_SourceData_images/3H/VEH_3_adult_42_dpi_no_reconex_cjun_19.lif_Series001/VEH_3_adult_42_dpi_no_reconex_cjun_19.lif_Series001_z01_ch01.tif]

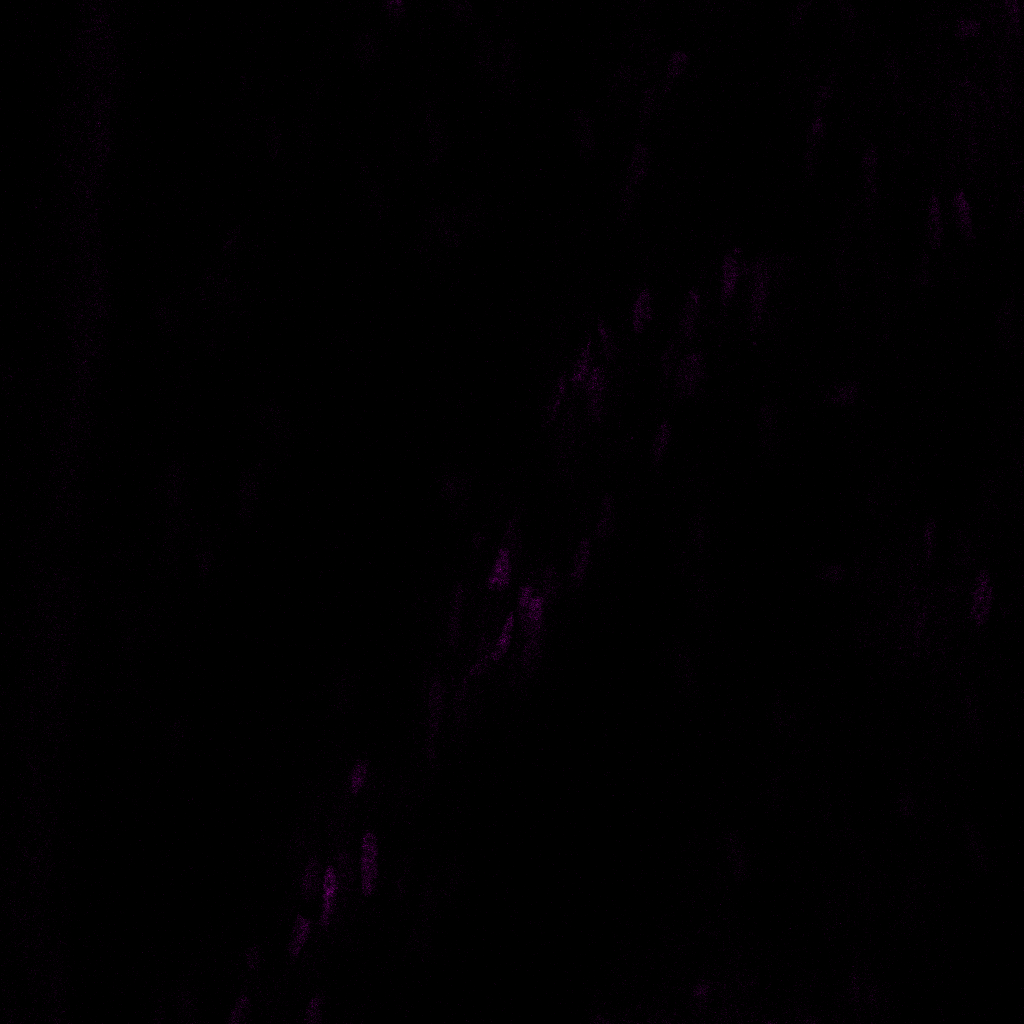

Supplement: Supplementary file 14 — Source Data for Figure 4 [file EMMM-15-e17907-s014.zip › SourceData_Fig_4/Fig_4_SourceData_images/3H/VEH_3_adult_42_dpi_no_reconex_cjun_19.lif_Series001/VEH_3_adult_42_dpi_no_reconex_cjun_19.lif_Series001_z01_ch02.tif]

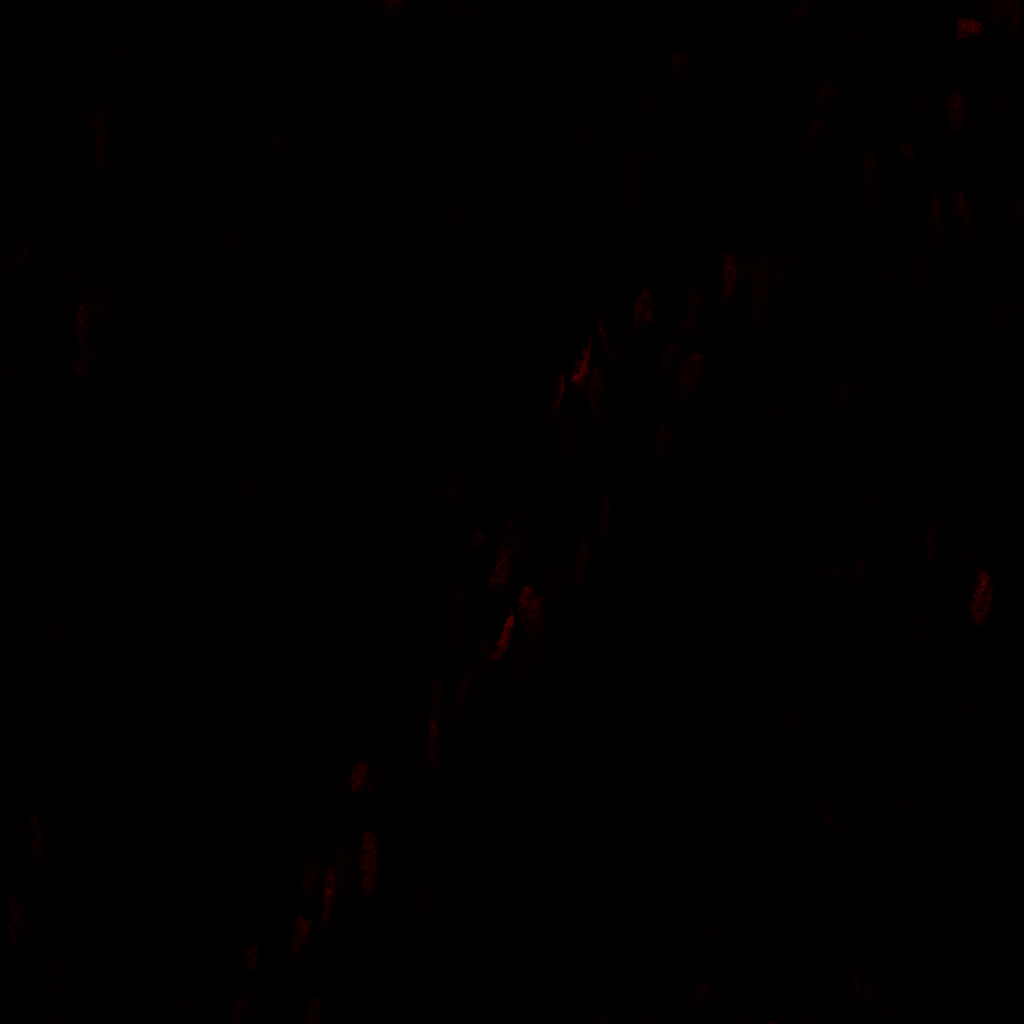

Supplement: Supplementary file 14 — Source Data for Figure 4 [file EMMM-15-e17907-s014.zip › SourceData_Fig_4/Fig_4_SourceData_images/3H/VEH_3_adult_42_dpi_no_reconex_cjun_19.lif_Series001/VEH_3_adult_42_dpi_no_reconex_cjun_19.lif_Series001_z01_ch03.tif]

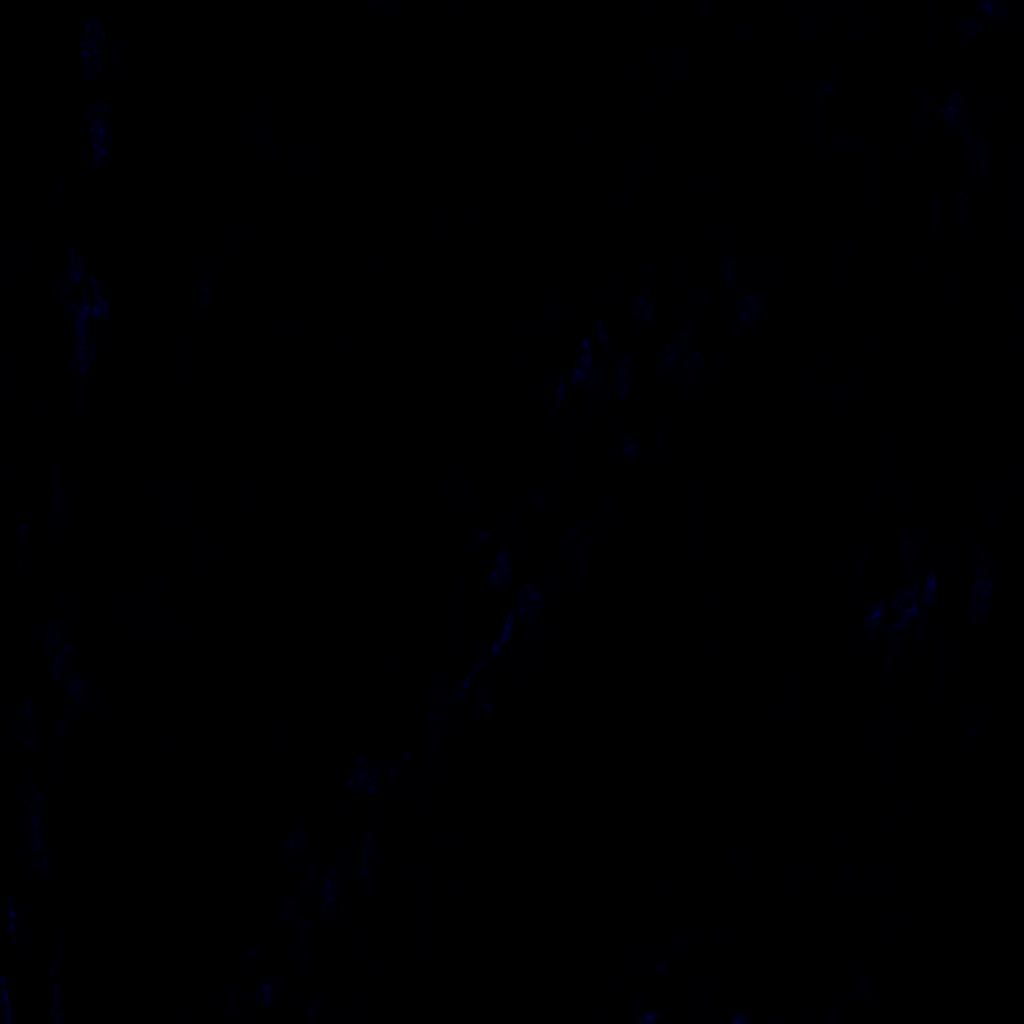

Supplement: Supplementary file 14 — Source Data for Figure 4 [file EMMM-15-e17907-s014.zip › SourceData_Fig_4/Fig_4_SourceData_images/3H/VEH_3_adult_42_dpi_no_reconex_cjun_19.lif_Series001/VEH_3_adult_42_dpi_no_reconex_cjun_19.lif_Series001_z02_ch00.tif]

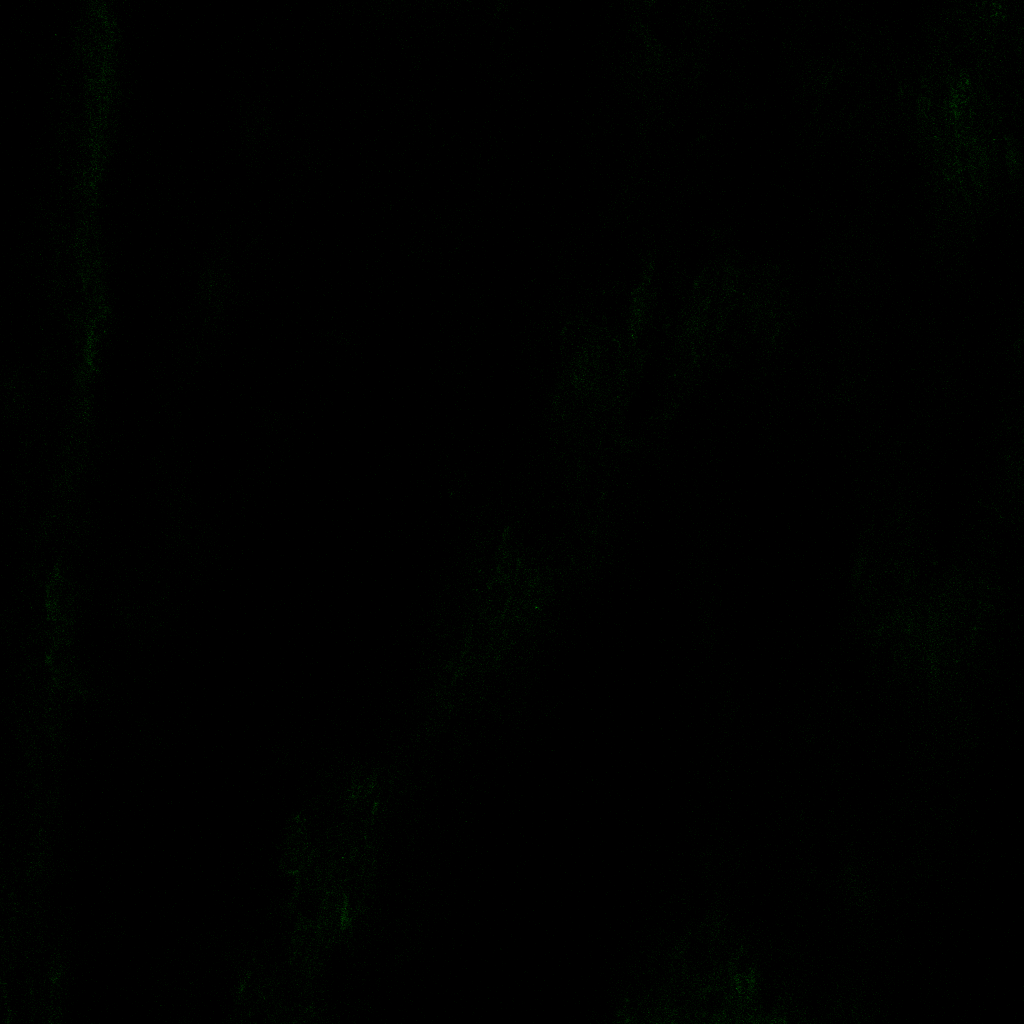

Supplement: Supplementary file 14 — Source Data for Figure 4 [file EMMM-15-e17907-s014.zip › SourceData_Fig_4/Fig_4_SourceData_images/3H/VEH_3_adult_42_dpi_no_reconex_cjun_19.lif_Series001/VEH_3_adult_42_dpi_no_reconex_cjun_19.lif_Series001_z02_ch01.tif]

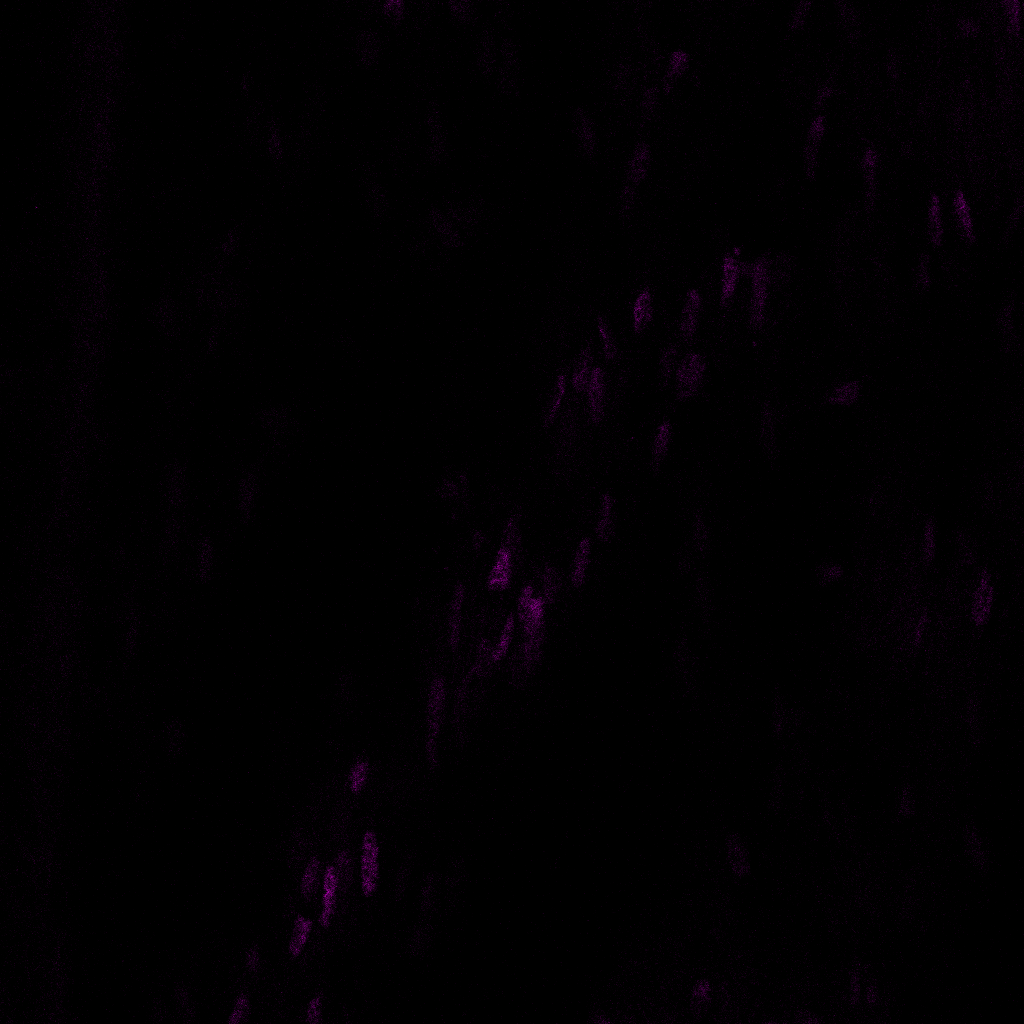

Supplement: Supplementary file 14 — Source Data for Figure 4 [file EMMM-15-e17907-s014.zip › SourceData_Fig_4/Fig_4_SourceData_images/3H/VEH_3_adult_42_dpi_no_reconex_cjun_19.lif_Series001/VEH_3_adult_42_dpi_no_reconex_cjun_19.lif_Series001_z02_ch02.tif]

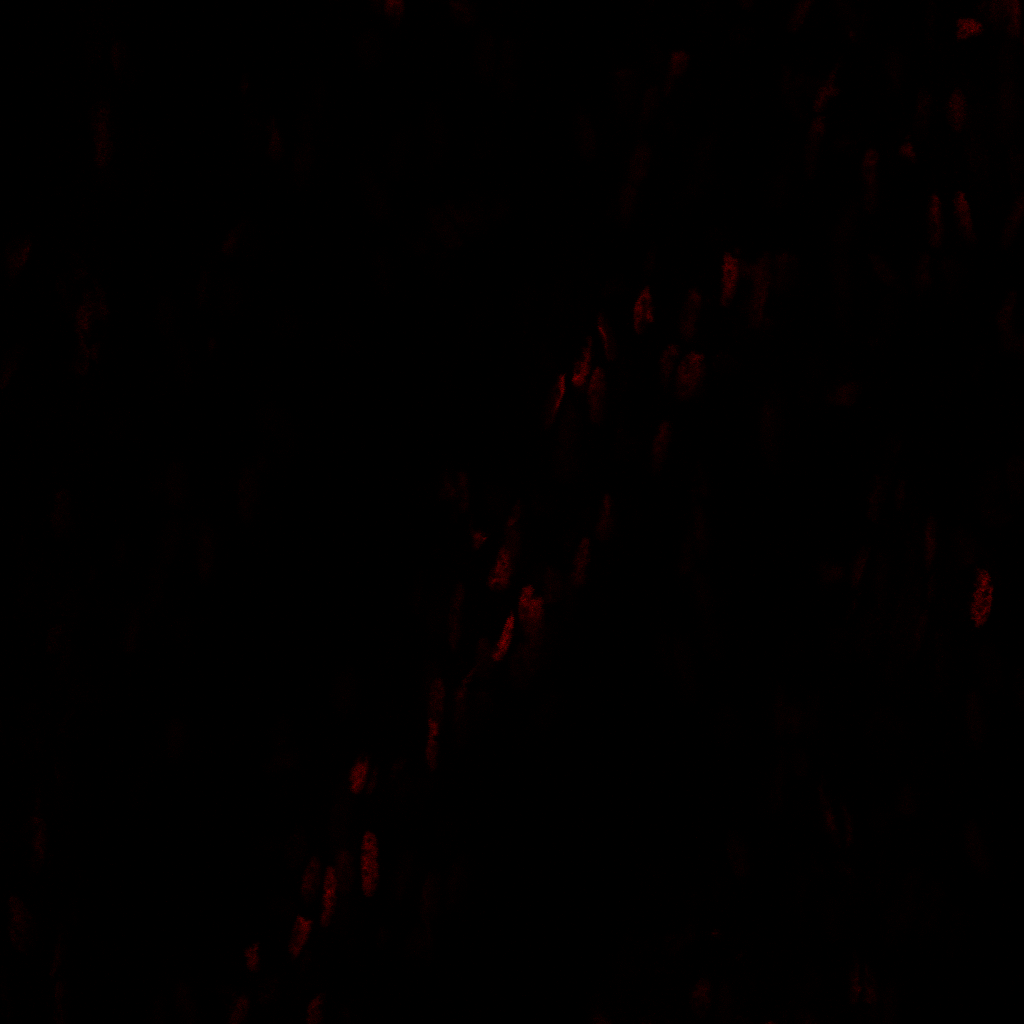

Supplement: Supplementary file 14 — Source Data for Figure 4 [file EMMM-15-e17907-s014.zip › SourceData_Fig_4/Fig_4_SourceData_images/3H/VEH_3_adult_42_dpi_no_reconex_cjun_19.lif_Series001/VEH_3_adult_42_dpi_no_reconex_cjun_19.lif_Series001_z02_ch03.tif]

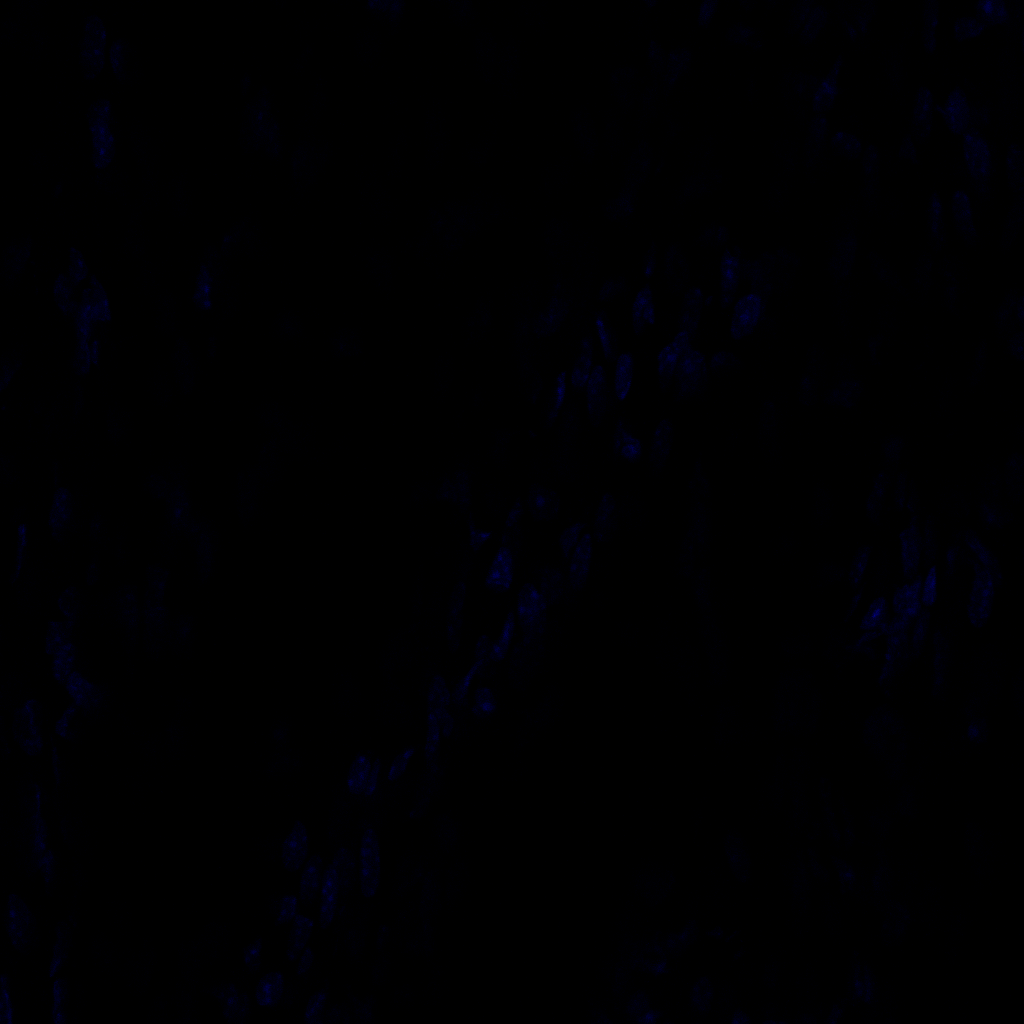

Supplement: Supplementary file 14 — Source Data for Figure 4 [file EMMM-15-e17907-s014.zip › SourceData_Fig_4/Fig_4_SourceData_images/3H/VEH_3_adult_42_dpi_no_reconex_cjun_19.lif_Series001/VEH_3_adult_42_dpi_no_reconex_cjun_19.lif_Series001_z03_ch00.tif]

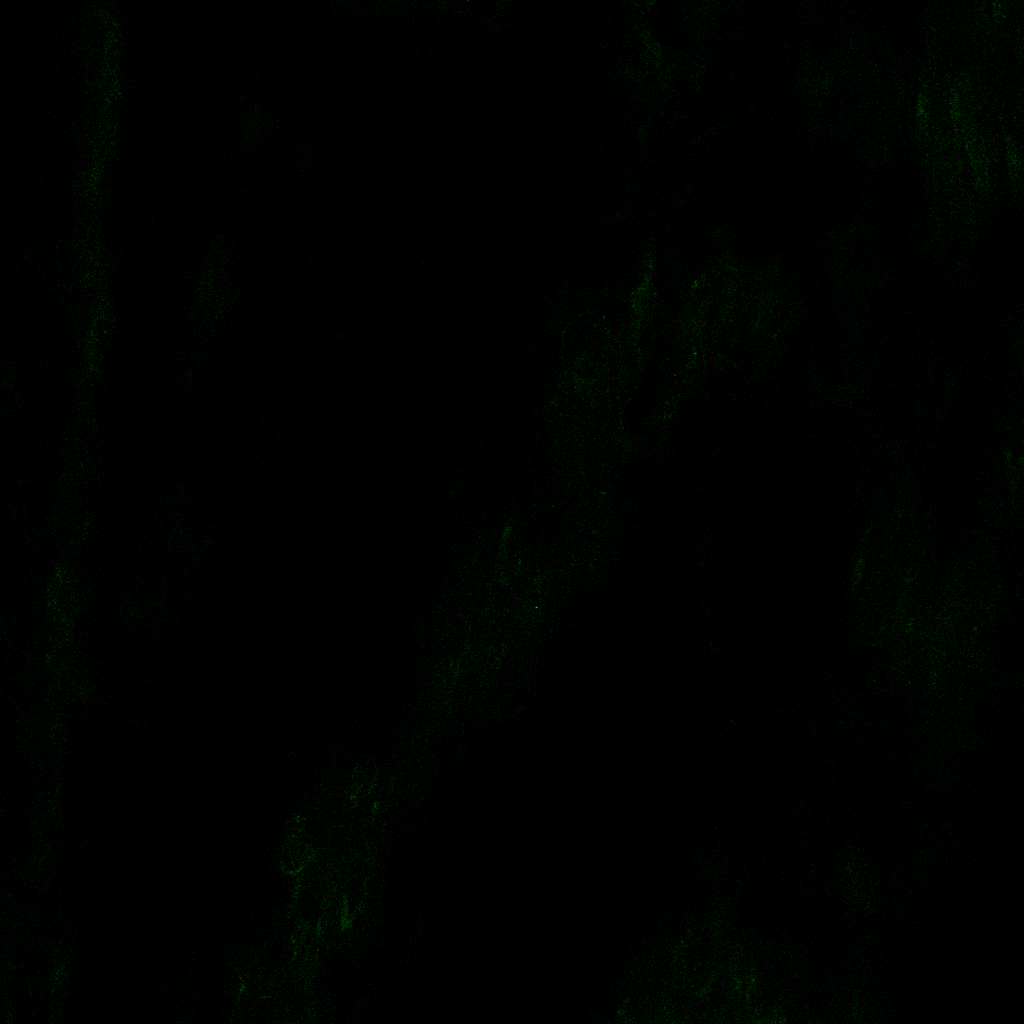

Supplement: Supplementary file 14 — Source Data for Figure 4 [file EMMM-15-e17907-s014.zip › SourceData_Fig_4/Fig_4_SourceData_images/3H/VEH_3_adult_42_dpi_no_reconex_cjun_19.lif_Series001/VEH_3_adult_42_dpi_no_reconex_cjun_19.lif_Series001_z03_ch01.tif]

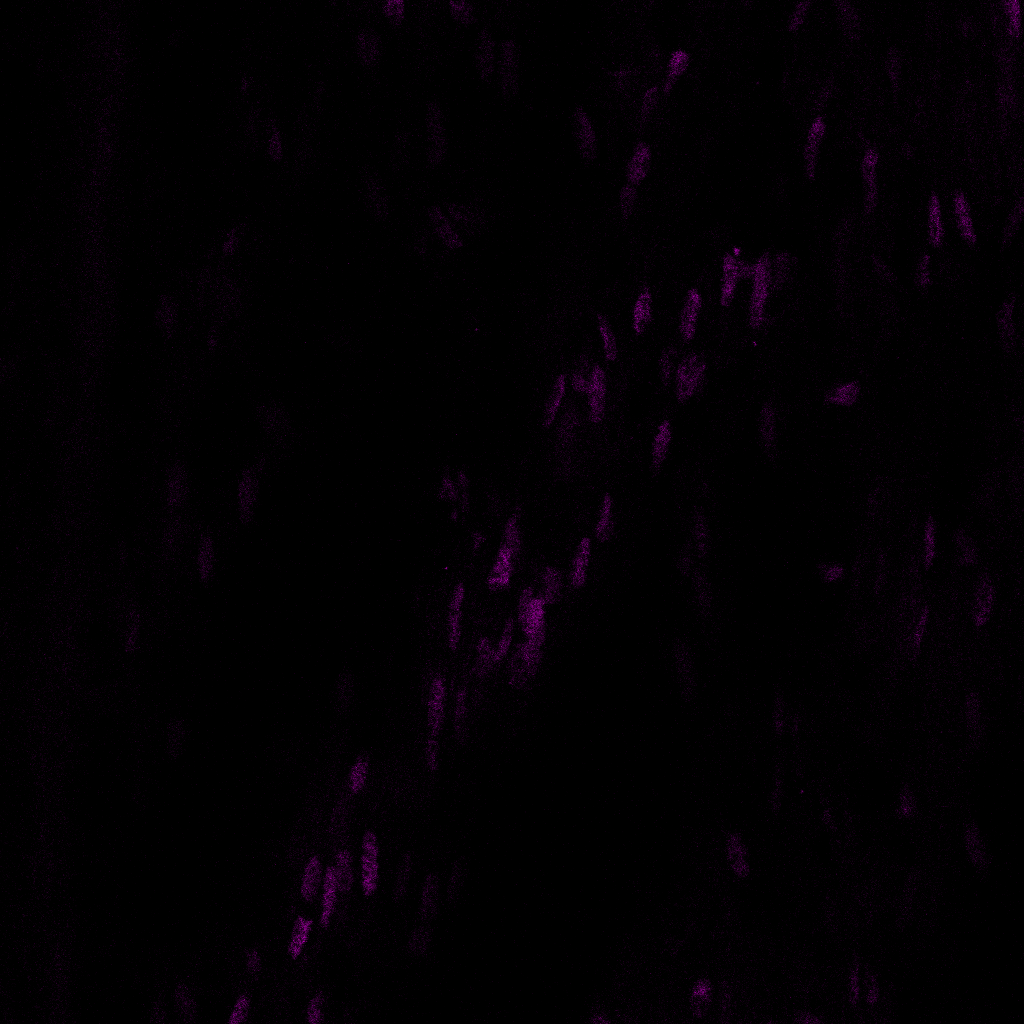

Supplement: Supplementary file 14 — Source Data for Figure 4 [file EMMM-15-e17907-s014.zip › SourceData_Fig_4/Fig_4_SourceData_images/3H/VEH_3_adult_42_dpi_no_reconex_cjun_19.lif_Series001/VEH_3_adult_42_dpi_no_reconex_cjun_19.lif_Series001_z03_ch02.tif]

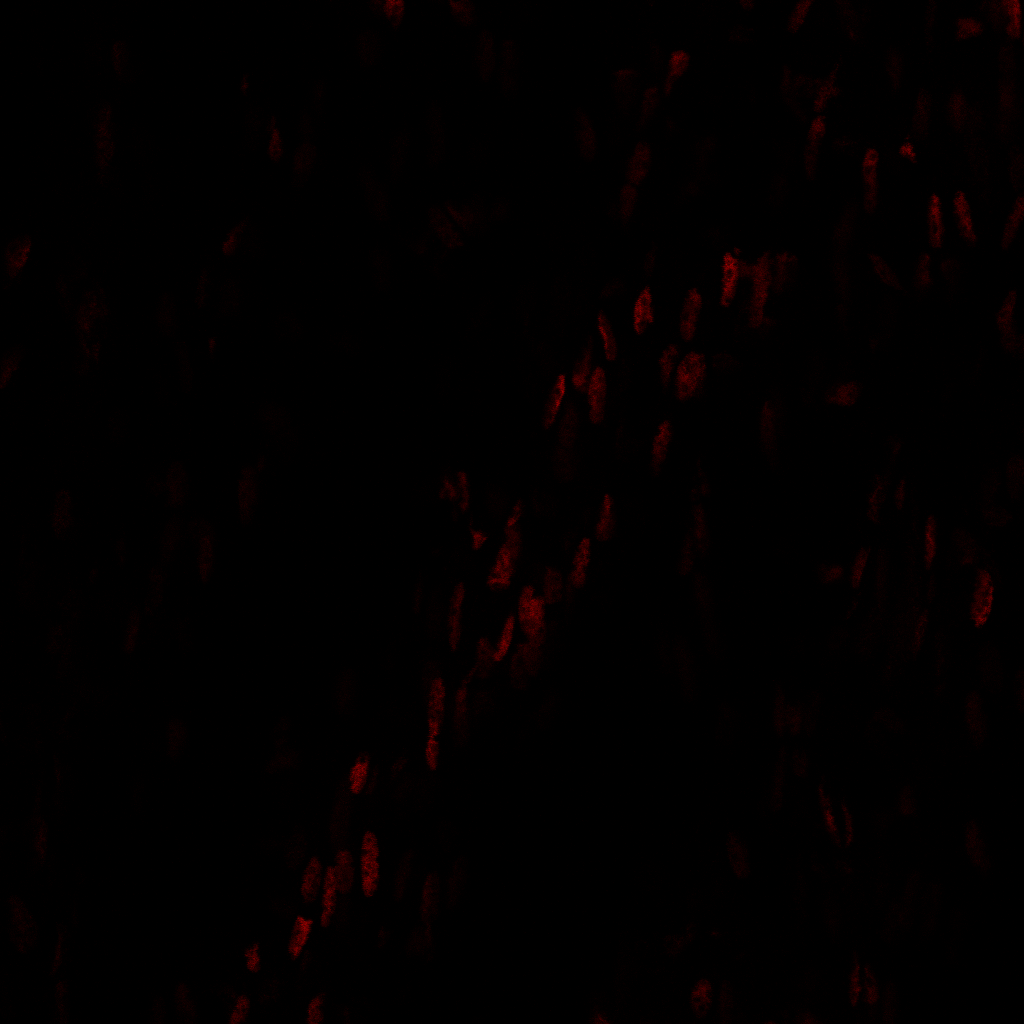

Supplement: Supplementary file 14 — Source Data for Figure 4 [file EMMM-15-e17907-s014.zip › SourceData_Fig_4/Fig_4_SourceData_images/3H/VEH_3_adult_42_dpi_no_reconex_cjun_19.lif_Series001/VEH_3_adult_42_dpi_no_reconex_cjun_19.lif_Series001_z03_ch03.tif]

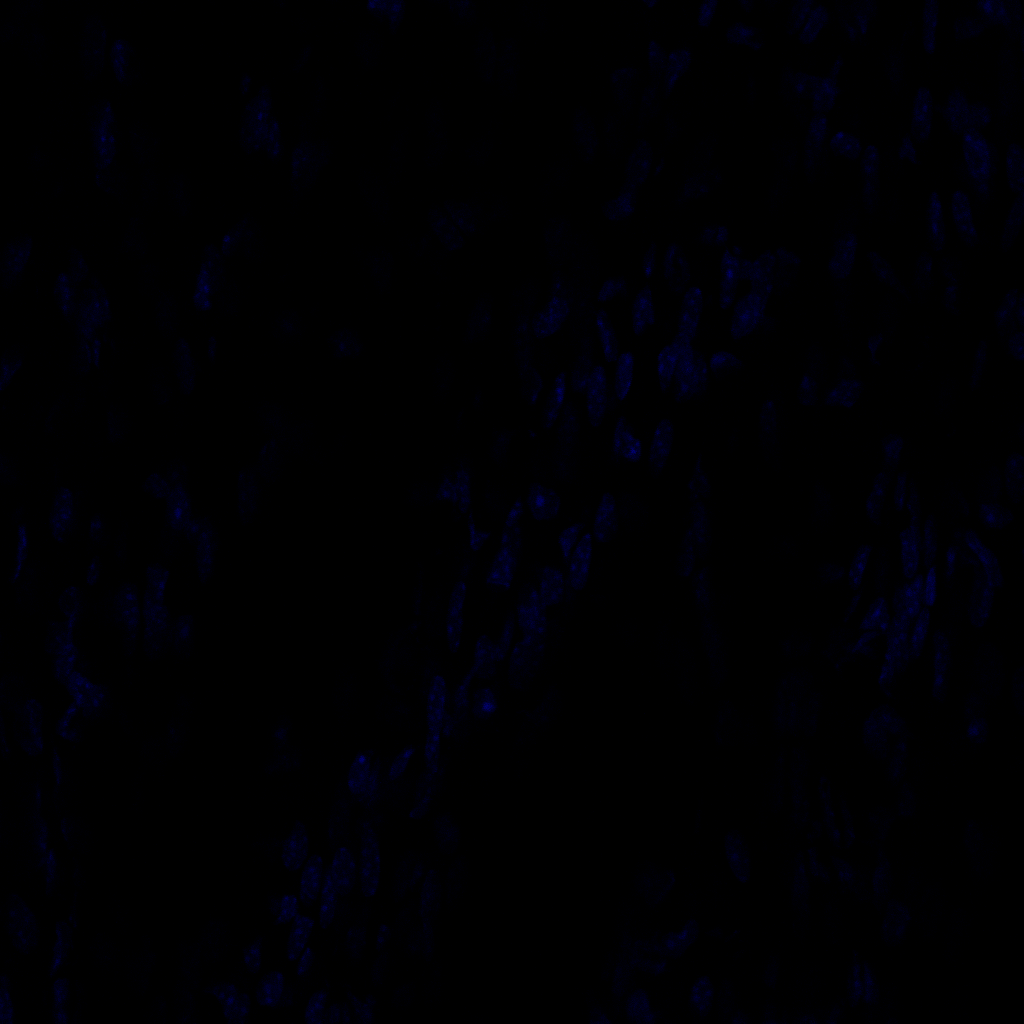

Supplement: Supplementary file 14 — Source Data for Figure 4 [file EMMM-15-e17907-s014.zip › SourceData_Fig_4/Fig_4_SourceData_images/3H/VEH_3_adult_42_dpi_no_reconex_cjun_19.lif_Series001/VEH_3_adult_42_dpi_no_reconex_cjun_19.lif_Series001_z04_ch00.tif]

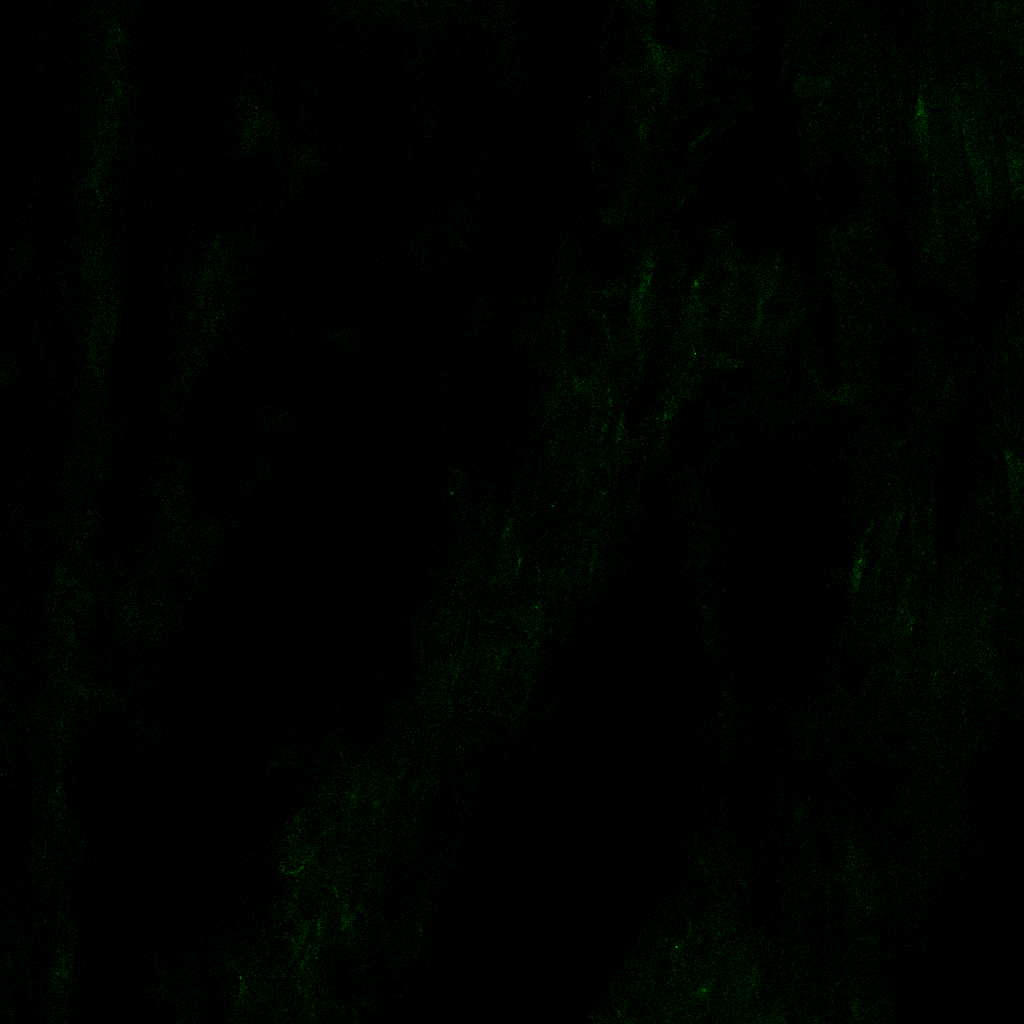

Supplement: Supplementary file 14 — Source Data for Figure 4 [file EMMM-15-e17907-s014.zip › SourceData_Fig_4/Fig_4_SourceData_images/3H/VEH_3_adult_42_dpi_no_reconex_cjun_19.lif_Series001/VEH_3_adult_42_dpi_no_reconex_cjun_19.lif_Series001_z04_ch01.tif]

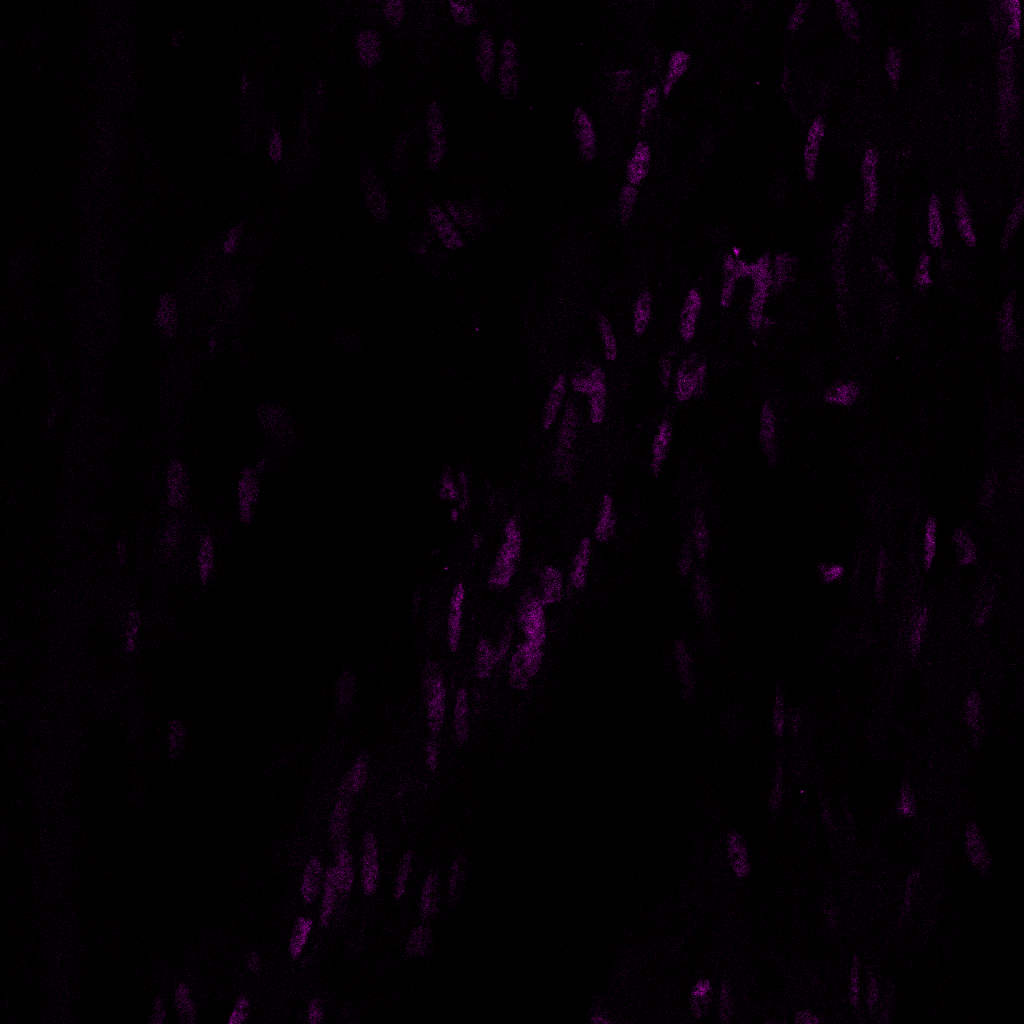

Supplement: Supplementary file 14 — Source Data for Figure 4 [file EMMM-15-e17907-s014.zip › SourceData_Fig_4/Fig_4_SourceData_images/3H/VEH_3_adult_42_dpi_no_reconex_cjun_19.lif_Series001/VEH_3_adult_42_dpi_no_reconex_cjun_19.lif_Series001_z04_ch02.tif]

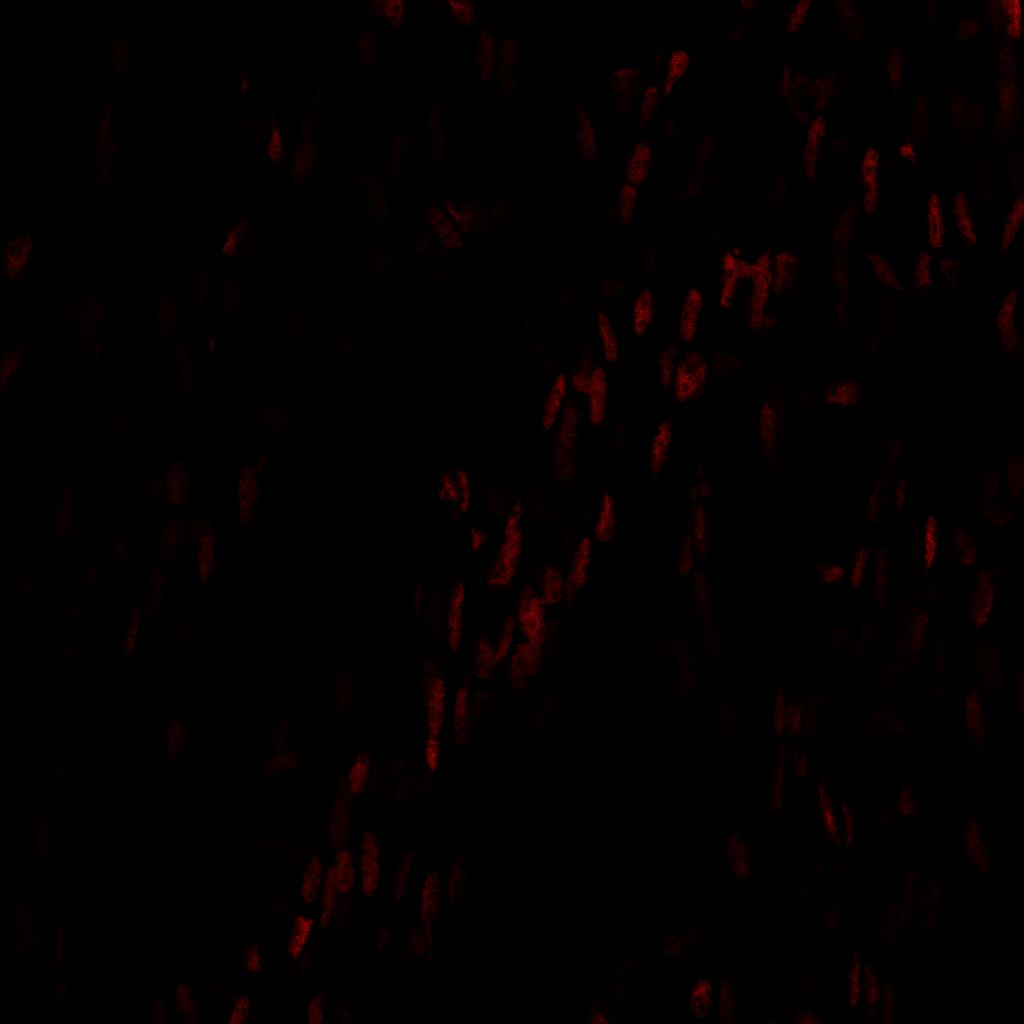

Supplement: Supplementary file 14 — Source Data for Figure 4 [file EMMM-15-e17907-s014.zip › SourceData_Fig_4/Fig_4_SourceData_images/3H/VEH_3_adult_42_dpi_no_reconex_cjun_19.lif_Series001/VEH_3_adult_42_dpi_no_reconex_cjun_19.lif_Series001_z04_ch03.tif]

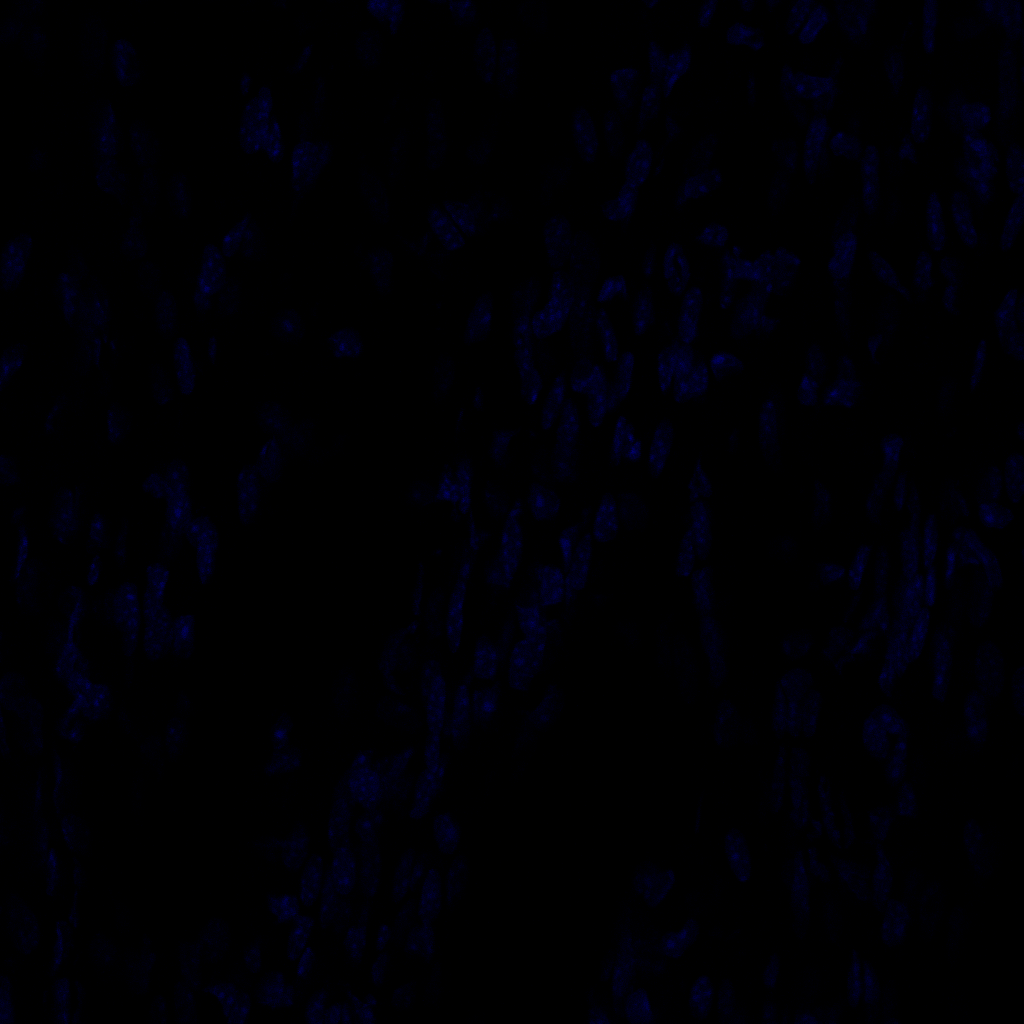

Supplement: Supplementary file 14 — Source Data for Figure 4 [file EMMM-15-e17907-s014.zip › SourceData_Fig_4/Fig_4_SourceData_images/3H/VEH_3_adult_42_dpi_no_reconex_cjun_19.lif_Series001/VEH_3_adult_42_dpi_no_reconex_cjun_19.lif_Series001_z05_ch00.tif]

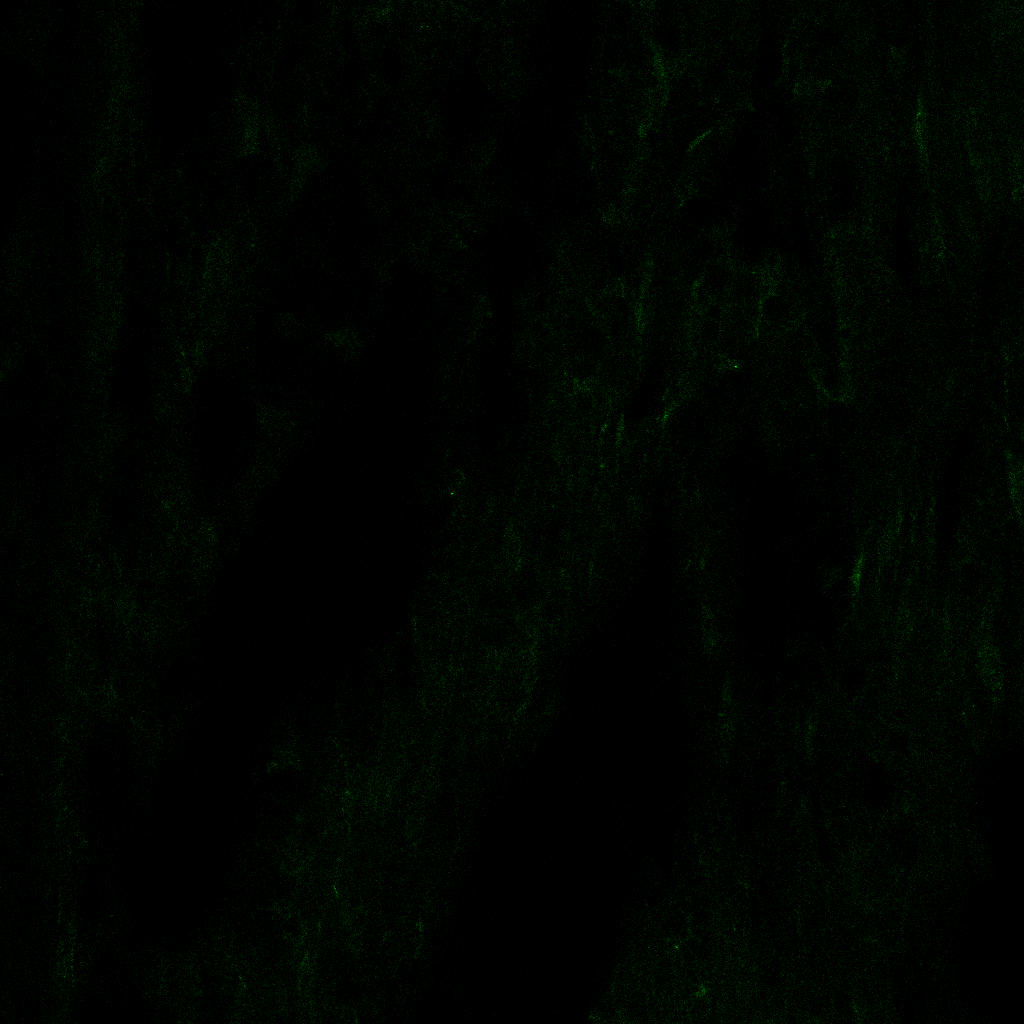

Supplement: Supplementary file 14 — Source Data for Figure 4 [file EMMM-15-e17907-s014.zip › SourceData_Fig_4/Fig_4_SourceData_images/3H/VEH_3_adult_42_dpi_no_reconex_cjun_19.lif_Series001/VEH_3_adult_42_dpi_no_reconex_cjun_19.lif_Series001_z05_ch01.tif]

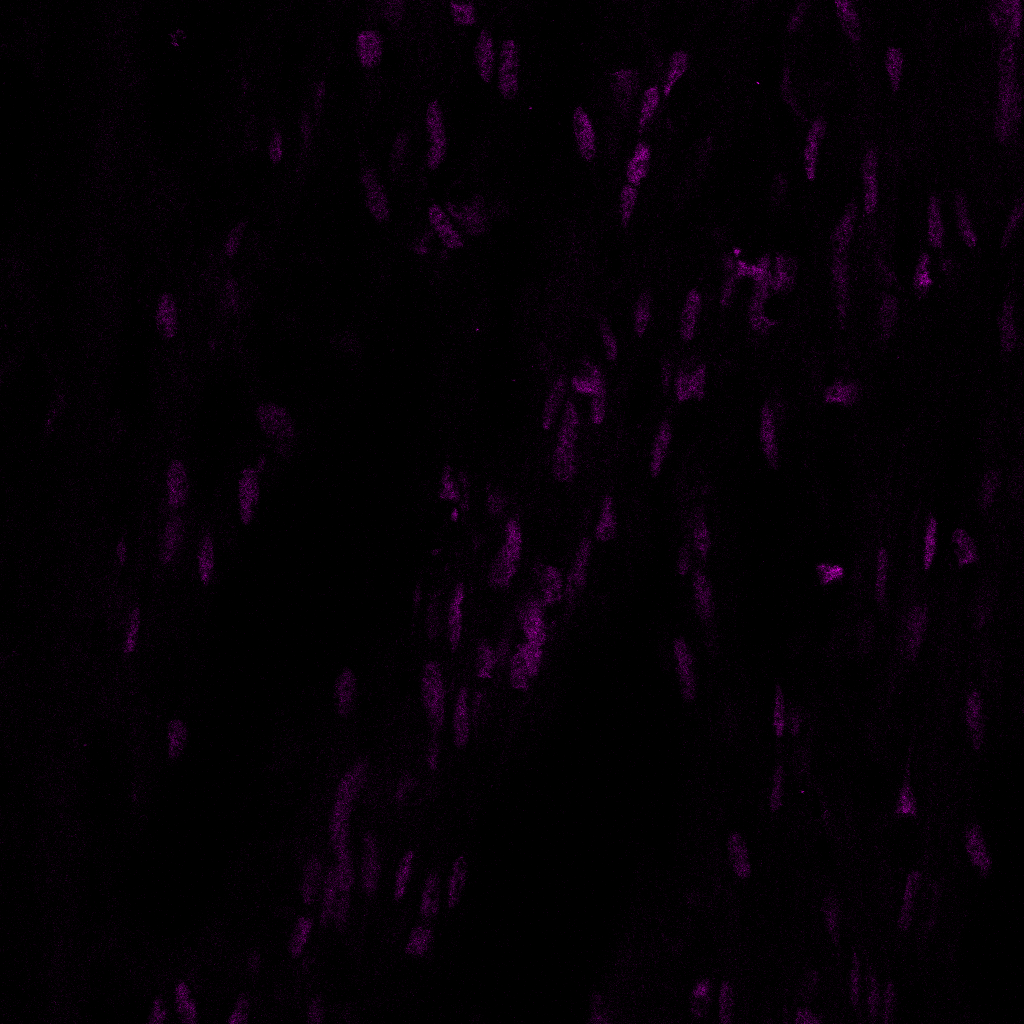

Supplement: Supplementary file 14 — Source Data for Figure 4 [file EMMM-15-e17907-s014.zip › SourceData_Fig_4/Fig_4_SourceData_images/3H/VEH_3_adult_42_dpi_no_reconex_cjun_19.lif_Series001/VEH_3_adult_42_dpi_no_reconex_cjun_19.lif_Series001_z05_ch02.tif]

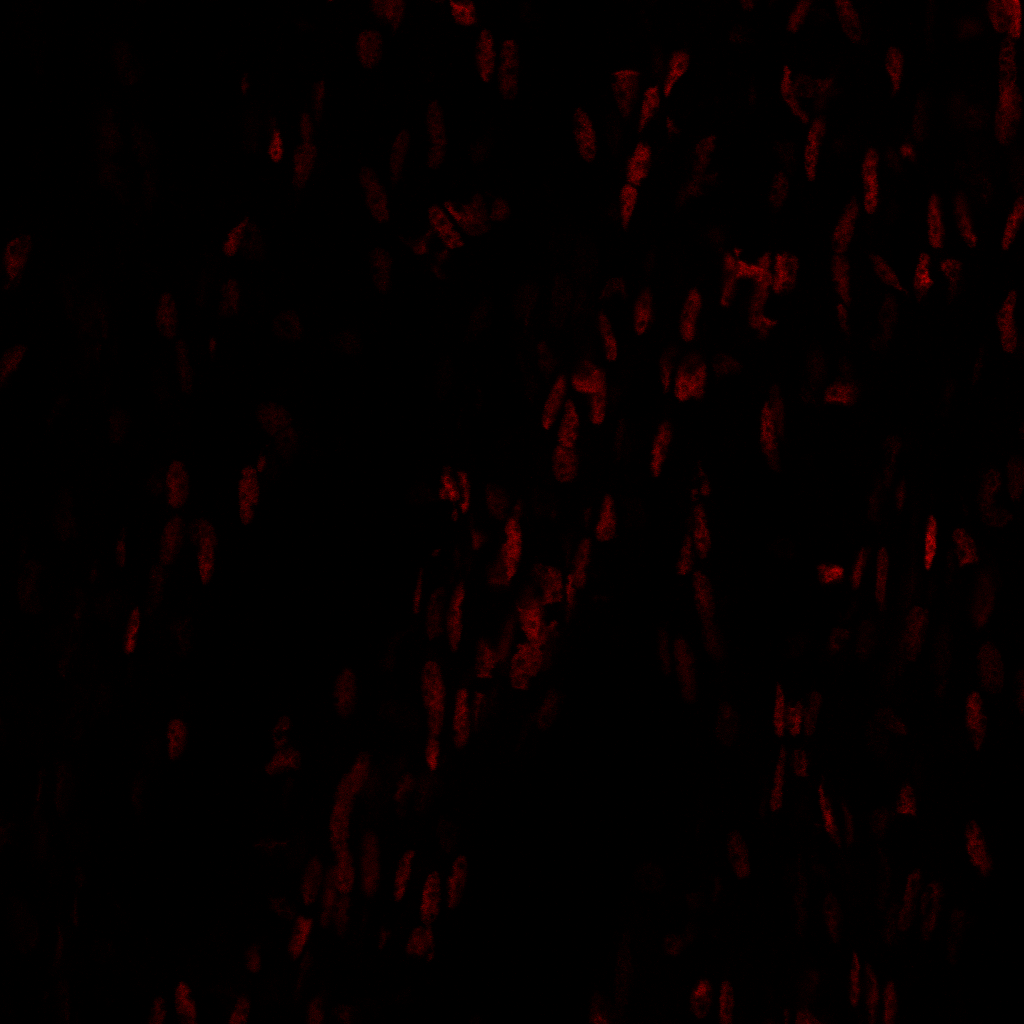

Supplement: Supplementary file 14 — Source Data for Figure 4 [file EMMM-15-e17907-s014.zip › SourceData_Fig_4/Fig_4_SourceData_images/3H/VEH_3_adult_42_dpi_no_reconex_cjun_19.lif_Series001/VEH_3_adult_42_dpi_no_reconex_cjun_19.lif_Series001_z05_ch03.tif]

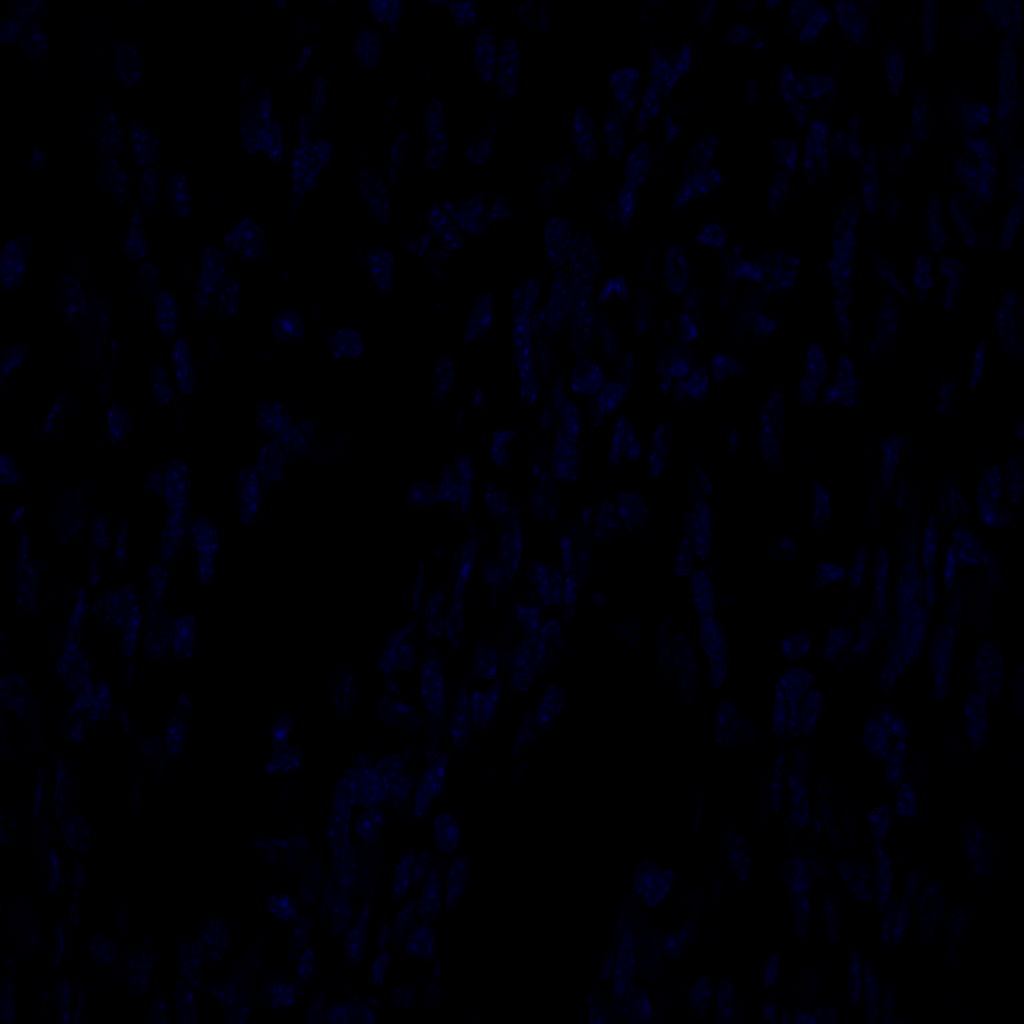

Supplement: Supplementary file 14 — Source Data for Figure 4 [file EMMM-15-e17907-s014.zip › SourceData_Fig_4/Fig_4_SourceData_images/3H/VEH_3_adult_42_dpi_no_reconex_cjun_19.lif_Series001/VEH_3_adult_42_dpi_no_reconex_cjun_19.lif_Series001_z06_ch00.tif]

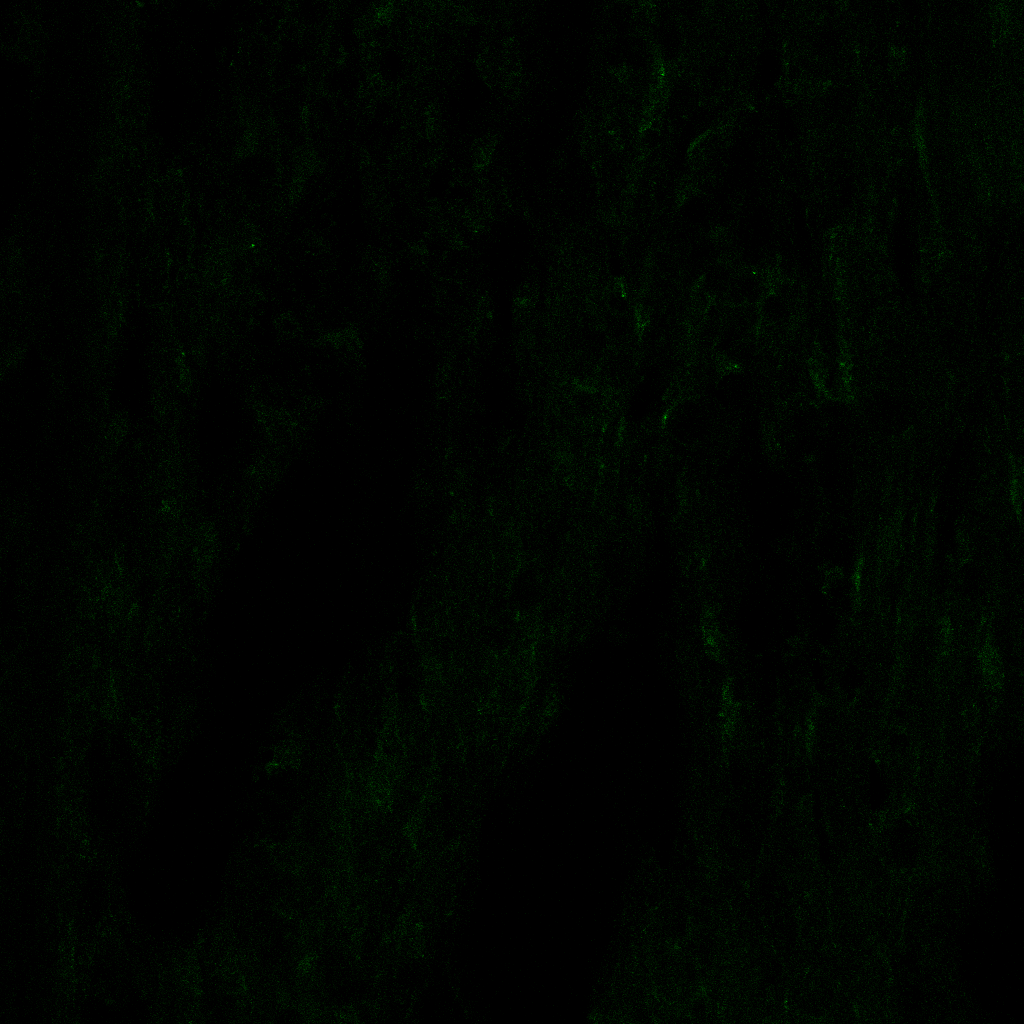

Supplement: Supplementary file 14 — Source Data for Figure 4 [file EMMM-15-e17907-s014.zip › SourceData_Fig_4/Fig_4_SourceData_images/3H/VEH_3_adult_42_dpi_no_reconex_cjun_19.lif_Series001/VEH_3_adult_42_dpi_no_reconex_cjun_19.lif_Series001_z06_ch01.tif]
