# Supplementary material for: Senescent Schwann cells induced by aging and chronic denervation impair axonal regeneration following peripheral nerve injury
Source: EMBO Mol Med. 2023 Oct 20;15(12):e17907. doi: 10.15252/emmm.202317907 (PMC10701627; doi:10.15252/emmm.202317907)
Supplement: Supplementary file 16 [file EMMM-15-e17907-s010.pdf]

EMBO Molecular Medicine

Senescent Schwann cells induced by aging and chronic denervation impair axonal regeneration following peripheral nerve injury

Andrés Fuentes-Flores, Cristian Gerónimo-Olvera, Karina Girardi, David Necuñir-Ibarra, Sandip Kumar Patel, Joanna Bons, Megan Wright, Daniel Geschwind, Ahmet Höke, Jose Gomez-Sanchez, Birgit Schilling, Daniela Rebolledo, Judith Campisi, and Felipe Court
DOI: 10.15252/emmm.202317907

Corresponding author: Felipe Court (felipe.court@umayor.cl)

Review Timeline:

Transfer from Review Commons:	25th Apr 23
Editorial Decision:	29th Apr 23
Revision Received:	18th Jul 23
Editorial Decision:	23rd Aug 23
Revision Received:	14th Sep 23
Accepted:	18th Sep 23

Editor: Kelly Anderson

Transaction Report:

This manuscript was transferred to EMBO Molecular Medicine following peer review at Review Commons.

(Note: With the exception of the correction of typographical or spelling errors that could be a source of ambiguity, letters and reports are not edited. Depending on transfer agreements, referee reports obtained elsewhere may or may not be included in this compilation. Referee reports are anonymous unless the Referee chooses to sign their reports.)

Review #1

1. Evidence, reproducibility and clarity:

Evidence, reproducibility and clarity (Required)

Summary

This manuscript by Fuentes-Flores et al reports that elimination of senescent Schwann cells by systemic senolytic drug treatment or genetic targeting improves nerve regeneration and functional recovery in aging and chronic denervation. This improved regeneration is associated with an upregulation of c-Jun expression. Mechanistically the authors provide data to show that senescent Schwann cells secrete factors that are inhibitory to axon regeneration. These findings are very interesting and move the field forward beyond the notion that Schwann cells fail to support axon regeneration in aged animals and identify potential targets to enhance nerve repair. The use of a senolytic drug to increase regeneration in aged and chronic denervation conditions provides new avenues for clinical interventions. However, some of the claims are overstated since this is not the first characterization of senescent Schwann cells and the manipulations used in the study are not entirely specific for Schwann cells. The manuscript is also poorly written and difficult to follow, given the complex set of surgeries and terminology, and lack of explanation of the rationale for the surgery model used. Figures are poorly labeled and difficult to follow without figure legends, and figure legends do not match the figures. Below are suggestions for improvements:

Major comments:

- In the axon regeneration assays, how is the reconnection site defined in longitudinal images stained for SCG10? Of particular concern is that Figure 1B "adult chronic dmg" nerve section image appears to be identical to the image in Figure 5A "Vehicle adult (47dpi)". However, the reconnection site is located at different sites along the nerve. Also, the scale bar appears identical but the legend states different sizes. In Figure 1 chronic damage is 42dpi, and figure 5 is 47dpi, yet with what appears as the same image.
- The authors need to provide a rationale for the choice of this complex injury model and what are the advantages over other models. Please clearly describe the timepoints for each experiment and why the time points were chosen for analysis. Provide the scheme of injury in the main figures to ease comprehension. A scheme is provided in what appears to be Figure S2, but the legend of Figure S2 does not match. Please compare same time points between aged and adults. Days post injury is sometimes referred to 7 or 42, and it is difficult to follow if it is days post initial transection of the tibial nerve or days post reconnection of transected tibial to peroneal. Revise all figure legends and supplementary figure legends to match figures.
- The authors' main conclusion is that Senescent Schwann cells inhibit axon regeneration. The authors need to tune down this statement and acknowledge that their manipulations are not entirely Schwann cells specific. While the data nicely shows a contribution of senescent Schwann cells, it does not sufficiently acknowledge the possibility that other senescent cells in the nerve contribute to this effect. First, the authors refer in the discussion that 60% of the senescent cells are SOX10 negative, and thus represent other cells beyond Schwann cells. This quantification needs to be shown in Figure 1. Second, the genetic and pharmacology

manipulation eliminate all senescent cells, including Schwann cells. Third, while the culture experiment may be Schwann cells specific, the authors need to provide detailed information on how they purify these cells, how they induce repair Schwann cells (rSC) as claimed in Figure 3, and demonstrate whether these are pure Schwann cells. This is important because other cells in the nerve contribute to nerve repair, including mesenchymal cells. Finally, the claim that using Mpz-cre will lead to c-jun overexpression only in SC also needs to be demonstrated, since Mpz is also expressed in satellite glial cells in the DRG.
- In Figure 5, c-jun is shown after denervation (42 dpi). The results describe 28 days of denervation, 5 days of GCV and 7 days post reconnection, which makes 40 days. If that is not the case, results need to better explain timeline of this procedure. Also, what is the basal c-jun expression in p16-3MR mice? In addition to the number of c-jun positive cells shown in Figure 5G, the authors need to quantify the percent of c-jun puncta that co-localize with Sox10. The size of the c-jun puncta appears different in size in vehicle and GCV, is that an expected phenotype?

Minor comments

- Improve labeling of Figures or at the very least describe in the Figure legend. For example: Figure 5B-C, which of the graphs is from adult mice and which is from aged mice?
- The authors need to carefully describe where the high magnification images were taken in the injured nerve and keep the comparison at same site between groups. Please check the scale bar for each image. For example, the images in Figure 1D/F/I/K used same scale, but the cell size and cell morphology are different. The images for split individual channels need to match the merge channel images. For example, the individual channel and merge images are not properly aligned in Figure 4C, ABY-263 group.
- The analysis method used to quantify axon regeneration should be consistent throughout. For example, in Figure 1C, number of axons/nerve width(um) was used for regeneration assay, but axon density (width corrected) was used in Figure 5B-C in regeneration assay.

Referees cross-commenting

I agree with all Referee #2's comments. Both sets of comments are important, complementary and point to the same major concerns that need to be addressed. Agree as well that both reviewer think this is an interesting and relevant study for the field of nerve repair, if revised appropriately.

2. Significance:

Significance (Required)

The findings reported in this manuscript are very interesting and will move the field of nerve repair forward. This paper will be of interest for basic science audience in the fields of aging and neurobiology and has also potential interest to the broader clinical and translational fields. Indeed, this paper provides data that Schwann cells entering a senescent stage not only fail to support axon regeneration in aged animals, but actively inhibit axon regeneration. While this reviewer raises questions on whether only senescent Schwann cells or other senescent cells in the nerve contribute to this effect, the identification of potential targets to enhance nerve repair is highly significant. Furthermore, the use of an FDA approved drug, currently in clinicals trials for its application as senotherapeutics, to increase axon

regeneration in aged and chronic dennervation conditions will provide new avenues for clinical applications.

3. How much time do you estimate the authors will need to complete the suggested revisions:

Estimated time to Complete Revisions (Required)

(Decision Recommendation)

Between 1 and 3 months

4. *Review Commons* values the work of reviewers and encourages them to get credit for their work. Select 'Yes' below to register your reviewing activity at Web of Science Reviewer Recognition Service (formerly Publons); note that the content of your review will not be visible on Web of Science.

Web of Science Reviewer Recognition

Yes

Review #2

1. Evidence, reproducibility and clarity:

Evidence, reproducibility and clarity (Required)

The reported study by Fuentes-Flores et al. shows that Schwann cells (SCs) in peripheral nerves undergo senescence with aging or in chronic denervation. This senescent SC phenotype correlates with downregulation of c-Jun expression and axon regeneration capacity and consequently, affecting functional recovery. The study has been undertaken by using in vivo mice model of chronically denervated sciatic nerve and in vitro of rat-primary cell cultures (Schwann cell, DRG-explants, and their coculture). Schwann cell exosome manipulation was also included to exploit their released factor as media for cell cultures.

Major comments:

1. Chronic denervated nerve model and Schwann cell phenotype

- Tibial nerve transection and chronic denervated nerve: As the referee have no information about this model (no reference is cited), a detailed description should be provided

highlighting the interest of such model compared to standard sciatic nerve lesion model.
- Should be shown, histological analysis of denervated tibial branch prior reconnection with freshly cut proximal peroneal branch with specific immunostainings of rSCs v.s. sSC associated with dapi nuclear staining (as used along this study). Specific staining for other cells should be also provided (i.e., macrophage and endothelial cells). In simple words, "how chronically denervated nerve looks like and what is his cellular content? This is necessary to responds to the following main referee question: does the increase/decrease of rSCs or sSC under specific condition all through the study concerns the SCs that have migrated from freshly cut peroneal branch into denervated tibial distal branch, or resident SCs that have survived in chronically denervated tibial distal branch. In other words, whether rSCs that migrate (and accompanying regenerating axons) into chronically denervated branch nerve undergo phenotype change into sSC because of the environment of chronically denervated nerve. This is not clearly described or discussed, and remain confusing for reader.
2. Support of up- and down-regulation of gene expression illustrated in fig.4
The conclusions and statements on up- or down-regulation of c-jun, yH2AX, beta-gal, P16, arise from quantitative and qualitative analysis from immunostaining of these specific markers by determining the number of positive cells rSCs vs. sSC. For these strong statements appropriate methods for quantification of protein levels such as western blots are requiered. For example, the statement of down regulation of c-jun expression, the quantitative graph shows strong increase in c-jun cell number under ABT263 treatment bu the histological photo does not illustrate such decrease in number of the cell. It shows rather an increase in brightness of c-jun staining. Thus, only appropriate method for protein level quantification could be conclusive. This would also remove the doubt that some photos are under- or over-exposed as it appears in the figure. For example, in aged animal under vehicle condition, there is no variability in staining intensity. Accordingly, one question to the authors: for quantification of cell number, are weakly stained cells considered as positive cell?
3. The method section should be revised in general
Methods could be described in brief only when are supported by provided refs in which the reader could find details. Several refs are missing, i.e., 4.4 for thermal allodynia, 4.5 for ABT263 gavage administration; senescence induction, ...
Quantification methods should be more detailed, several information are missing and not found in result section or legends (i.e., number of nerve section per animal, neurite length, ...).
4. The use of rat for in vitro DRG and SC culture while in vivo study is undertaken on mice
The switche of species from in vivo to in vitro (mice vs. rat), is not justified as mice DRG and SC culture are also commonly used. In addition, the use of transgenic mice (used here only for in vivo) could also be exploited to address specific and reinforce the data.
5. Use of conditioned exosome/media
Should be explained why the use of exosomes directly in cell culture was not tested. This would be close to physiological condition, regarding the concentration of released factors.
6. The statement on the effect of rSC vs. sSC cell on growth cone dynamic
The provided data illustrated in fig. 3 are not in support that sSC affect growth cone dynamics. Only what would be "suggested" is that the decrease in neurite length could be associated to changes of growth cone morphology, on fixed tissue, that appeared to be affected. If such statement has to be maintained, time-laps is required. The image does not reflect a retracting neurite nor collapsed growth cone. In addition, other mechanisms could be at the basis of observed decrease in neurite length, which are not evaluated here. This is an important point to address as the authors state that sSC relase inhibitory factors.

Other comments

- The surgical description is complicated, also annotation to be added in supp fig #2A; provide
- M&M 4.2: lign #8, error referring to Fig 2A, correct by. Supp fig 2A
- Review refs list, ref#17, full info needed
- Fig 3H would be interesting only if contain a column of the 21 proteins exclusively expressed in senescent-induced SCs
- The immunostaining of Lamin B1 positive nuclear invaginations in fig S4 should better described in results for non-familiar reader
- Title of Fig 3, the expression "neuronal growth" is not appropriate here (neurite outgrowth)

Referees cross-commenting

I agree with referee#1's comments. He/she has raised complementary and important points that should be taken into account by the authors as well as we share the same major concerns. Furthermore, we both expressed the interest of such study if revised appropriately.

2. Significance:

Significance (Required)

Overall, this is an interesting study that undertake fundamental question in the field of nerve physiopathology and also could open a good opportunity in developing therapeutic strategies for translational research. However, additional investigations are needed to support the main conclusions.

3. How much time do you estimate the authors will need to complete the suggested revisions:

Estimated time to Complete Revisions (Required)

(Decision Recommendation)

Cannot tell / Not applicable

4. *Review Commons* values the work of reviewers and encourages them to get credit for their work. Select 'Yes' below to register your reviewing activity at [Web of Science Reviewer Recognition Service] (formerly Publons); note that the content of your review will not be visible on Web of Science.

Web of Science Reviewer Recognition

Yes

Revision Plan

Manuscript number: RC-2022-01784
Corresponding author(s): Felipe, Court

1. General Statements [optional]

We submit a revision plan for our manuscript *"Senescent Schwann cells induced by aging and chronic denervation impair axonal regeneration after peripheral nerve injury"* by Fuentes-Flores *et al*. from the groups of Felipe Court, Judith Campisi, Jose Gomez, and Ahmet Hoke.

One of the greatest challenges in the field of peripheral nerve regeneration is the decrease in the nerve regenerative capacity in aged patients or after delayed repair, a condition also known as chronic denervation. For the last two decades, several research groups have focused on understanding this phenomenon, but the main drivers of unsuccessful regeneration and poor functional recovery have been elusive, remaining an important clinical problem.

In the work described in this manuscript we found an unexpected property of Schwann cells in the denervated nerves. Aged and chronically denervated Schwann cells are not just passive participants in the impaired regeneration process, but they actively inhibit the regeneration of peripheral axons. Using a combination of morphological, behavioral and molecular techniques in a collaborative multi-lab approach we demonstrate for the first time that senescent Schwann cells accumulate in aged or chronically denervated peripheral nerves modifying the nerve environment, increasing proinflammatory and regeneration-inhibitory factors. Elimination of senescent Schwann cells using a systemic intervention with senolytic or genetically targeting p16-positive senescent cells, greatly improve axonal regeneration in both chronic denervation and aging conditions. Importantly, the enhanced axonal regeneration observed after senescent cell elimination is accompanied by improved functional recovery after chronic denervation. Chronic denervation and aging are the main clinical problems associated to peripheral nerve injuries. Our approach, using FDA approved drugs currently in clinicals trials for its application as senotherapeutics, effectively broadens the spectrum of its clinical use and effectiveness.

We foresee this work will be of interest to a wide audience, including experts in nerve regeneration, senescent cells, aging and those studying the effect of chronic insults in regenerative medicine.

We have now received the comments from two reviewers and we are prepared to experimentally approach the issues raised. We thank their criticism and suggestions, as well as their very enthusiastic comments. We are extremely pleased as both reviewers recognized the important implication of this work, from reviewer 1:

"The findings reported in this manuscript are very interesting and will move the field of nerve repair forward. This paper will be of interest for basic science audience in the fields of aging and neurobiology and has also potential interest to the broader clinical and translational fields. Indeed, this paper provides data that Schwann cells entering a senescent stage not only fail to support axon regeneration in aged animals, but actively inhibit axon regeneration…. Furthermore, the use of an FDA approved drug, currently in clinicals trials for its application as senotherapeutics, to increase axon regeneration in aged and chronic denervation conditions will provide new avenues for clinical applications".

Which is backed up by reviewer 2:

"Overall, this is an interesting study that undertake fundamental question in the field of nerve physiopathology and also could open a good opportunity in developing therapeutic strategies for translational research".

We understand the reviewers have raised issues associated with the manuscript format and we are prepared to profoundly edit the manuscript as suggested. In addition, after discussion the experimental issues raised by the reviewers, we are prepared to perform all the experiments and controls suggested (some of them are currently underway), including new animal experiments and *in vitro* work. This information is detailed in the point-by point revision plan below.

Thank you in advance for the consideration and we look forward to hearing from you in due course. Please do not hesitate to contact me if you want to discuss anything associated to the manuscript and the revision plan.

2. Description of the planned revisions

Point-by point Reponses and revision plan in blue

Reviewer #1 (Evidence, reproducibility and clarity (Required)):

Summary

This manuscript by Fuentes-Flores et al reports that elimination of senescent Schwann cells by systemic senolytic drug treatment or genetic targeting improves nerve regeneration and functional recovery in aging and chronic denervation. This improved regeneration is associated with an upregulation of c-Jun expression. Mechanistically the authors provide data to show that senescent Schwann cells secrete factors that are inhibitory to axon regeneration. These findings are very interesting and move the field forward beyond the notion that Schwann cells fail to support axon regeneration in aged animals and identify potential targets to enhance nerve repair. The use of a senolytic drug to increase regeneration in aged and chronic denervation conditions provides new avenues for clinical interventions. However, some of the claims are overstated since this is not the first characterization of senescent Schwann cells and the manipulations used in the study are not entirely specific for Schwann cells. The manuscript is also poorly written and difficult to follow, given the complex set of surgeries and terminology, and lack of explanation of the rationale for the surgery model used. Figures are poorly labeled and difficult to follow without figure legends, and figure legends do not match the figures.

We thank the reviewer for the positive comments, we also acknowledge the problems detected in the manuscript format, including the lack of a clear explanation of the complex procedures used. We are prepared to work carefully on the format, including clear explanations of the procedures and new schemes to complement the text. As detailed below we are also prepared to perform all the experimental work proposed by the reviewer, which will strengthen the conclusion of this manuscript.

Below are suggestions for improvements:

Major comments:

• In the axon regeneration assays, how is the reconnection site defined in longitudinal images stained for SCG10? Of particular concern is that Figure 1B "adult chronic dmg" nerve section image appears to be identical to the image in Figure 5A "Vehicle adult (47dpi)". However, the reconnection site is located at different sites along the nerve. Also, the scale bar appears identical but the legend states different sizes. In Figure 1 chronic damage is 42dpi, and figure 5 is 47dpi, yet with what appears as the same image.

Revisions incorporated in the transferred manuscript, see section 3, below.

• The authors need to provide a rationale for the choice of this complex injury model and what are the advantages over other models. Please clearly describe the timepoints for each experiment and why the time points were chosen for analysis. Provide the scheme of injury in the main Figures to ease comprehension. A scheme is provided in what appears to be Figure S2, but the legend of Figure S2 does not match. Please compare same time points between aged and adults. Days post injury is sometimes referred to 7 or 42, and it is difficult to follow if it is days post initial transection of the tibial nerve or days post reconnection of transected tibial to peroneal. Revise all Figure legends and supplementary Figure legends to match figures.

We thank the reviewer for this comment. In a revised manuscript we will provide a clear explanation for the injury model used, as well as references, including one from the group of Tessa Gordon that describe for the first time this model in rats (PMID: 30215557), and the one applying this model to the mouse from the groups of Rhona Mirsky and Kristjan Jessen (PMID: 33475496). Briefly, this model has two advantages: first, it allows to generate chronic denervation for months and then be able to connect the distal denervated stump with a proximal one without the need of a nerve bridge; And second, neurons in the different groups that have different denervation times (1 week versus 6 weeks) are all damaged at the same time, eliminating variability associated to chronic axonal damage. We will include this information in the results section of a revised manuscript along with the above references.

We will include schemes of the injuries performed in each experiment in each figure, also adding a timeline. This is an excellent suggestion to clearly understand the different procedures performed. We will also check all figures and legends, including correcting the problem detected by the reviewer (legends of Figures 2 and 5 were swapped). We understand the problem of referring to days post-injury, then we will introduce a new form of referring to the initial transection and the experiment, which include reconnection. Adding schemes per figure will also help to understand the different timelines used in different experiments, including the ones using the senolytics.

As detailed above, we will perform a very careful revision of the text and legends for consistency.

• The authors' main conclusion is that Senescent Schwann cells inhibit axon regeneration. The authors need to tune down this statement and acknowledge that their manipulations are not entirely Schwann cells specific. While the data nicely shows a contribution of senescent Schwann cells, it does not sufficiently acknowledge the possibility that other senescent cells in the nerve contribute to this effect. First, the authors refer in the discussion that 60% of the senescent cells are SOX10 negative, and thus represent other cells beyond Schwann cells. This quantification needs to be shown in Figure 1. Second, the genetic and pharmacology manipulation eliminate all senescent cells, including Schwann cells. Third, while the culture experiment may be Schwann cells specific, the authors need to provide detailed information on how they purify these cells, how they induce repair Schwann cells (rSC) as claimed in

Figure 3, and demonstrate whether these are pure Schwann cells. This is important because other cells in the nerve contribute to nerve repair, including mesenchymal cells. Finally, the claim that using Mpz-cre will lead to c-jun overexpression only in SC also needs to be demonstrated, since Mpz is also expressed in satellite glial cells in the DRG.

We thank the reviewer for these comments and suggestions. We will tone down the statement that senescence Schwann cells are the only cell candidates for modulating regeneration. We discussed this in the original manuscript, but we agree we need to review this statement, including new data detailed below.

We will include the data requested by the reviewer (60% SOX-10 negative senescent cells) in a new graph in Figure 1. Also, we are currently performing new experiments and quantifications using specific markers for macrophages, epithelial cells, and fibroblasts, to identify the cell identity of the 40% SOX-10 negative senescent cells in aging and chronic denervation.

Regarding *in vitro* experiments, we will provide detailed methods for Schwann cell purification, and induction of rSC phenotype. Related to the purity of these cultures, in past experiments we have obtained numbers ranging from 95-98% of Schwann cell purity; we will repeat these experiments and quantification for this manuscript and include this data in the method section of a revised text.

Regarding c-jun overexpression in the Mpz-cre, we agree with the reviewer that there is probably overexpression in satellite glial cells in the DRG. Satellite glial cells (SGCs) are a subset of cells in the Schwann cell (SC) lineage that express several early myelination markers, such as Mbp, Mag, and Plp, and the transcription factor Sox10. SGCs express early SC markers, such as CDH19, and are transcriptionally and morphologically similar to SCs, even in the absence of axonal contact. Regarding the possibility that SGCs are contributing to the enhanced regeneration presented by mice with c-jun overexpression, this issue was somehow approached previously by Wagstaff et al. (PMID: 33475496), as they showed that in this mouse strain, increased axonal regeneration was equally observed in sensory neurons, in contact with SGCs, and motor neurons, which are not associated to SGCs. This observation suggests that the effect is associated with c-jun overexpressing Schwann cells in the distal stump. In addition, in our work, the changes in senescent cells observed in the c-jun overexpressed mouse, were associated to the distal nerve stump, which was mechanically separated from the proximal region. We agree it is important to include this discussion, and we will do so in the revised manuscript.

• In Figure 5, c-jun is shown after denervation (42 dpi). The results describe 28 days of denervation, 5 days of GCV and 7 days post reconnection, which makes 40 days. If that is not the case, results need to better explain timeline of this procedure. Also, what is the basal c-jun expression in p16-3MR mice? In addition to the number of c-jun positive cells shown in Figure 5G, the authors need to quantify the percent of c-jun puncta that co-localize with Sox10. The size of the c-jun puncta appears different in size in vehicle and GCV, is that an expected phenotype?

As expressed above, we will include schemes and timelines for all the surgical experiments in a revised manuscript, including detailed information for the experiments using the p16-3MR mice.

Regarding c-jun expression in the p16-3MR mice, we are currently performing the suggested control experiment which is important to draw conclusions of this research. We will use immunofluorescence, but

also include western blots as an extra analytical method in this and other experiments. All this information will be included in a revised manuscript.

The observation of the apparent difference c-jun puncta is intriguing. Is not an expected phenotype, but it will important to check if there is a quantifiable change in the pattern of expression. We will quantify this in the different groups and include the results in a revised Figure 5.

Minor comments

• Improve labeling of Figures or at the very least describe in the Figure legend. For example: Figure 5B-C, which of the graphs is from adult mice and which is from aged mice?

We are sorry about the lack of clear labeling in the figures; we will carefully review all figures in the manuscript and their corresponding legends, adding better labeling. Labelling of Figure 5B-C has been corrected.

• The authors need to carefully describe where the high magnification images were taken in the injured nerve and keep the comparison at same site between groups. Please check the scale bar for each image. For example, the images in Figure 1D/F/I/K used same scale, but the cell size and cell morphology are different. The images for split individual channels need to match the merge channel images. For example, the individual channel and merge images are not properly aligned in Figure 4C, ABY-263 group.

As suggested by the reviewer, we will show the regions in which high magnification were taken. All quantifications were performed in comparable sites, but we will include information in a revised manuscript to clearly describe the methodology used. We will check all scale bars in a revised manuscript.

For the comment on alignment problems we have incorporated this in the transferred manuscript, see section 3 below.

• The analysis method used to quantify axon regeneration should be consistent throughout. For example, in Figure 1C, number of axons/nerve width(um) was used for regeneration assay, but axon density (width corrected) was used in Figure 5B-C in regeneration assay.

Revisions incorporated in the transferred manuscript, see section 3, below.

****Referees cross-commenting****
I agree with all Referee #2's comments. Both sets of comments are important, complementary and point to the same major concerns that need to be addressed. Agree as well that both reviewer think this is an interesting and relevant study for the field of nerve repair, if revised appropriately.

Reviewer #1 (Significance (Required)):
Significance
The findings reported in this manuscript are very interesting and will move the field of nerve repair forward. This paper will be of interest for basic science audience in the fields of aging and neurobiology and has also potential interest to the broader clinical and translational fields. Indeed, this paper provides data that Schwann cells entering a senescent stage not only fail to support axon regeneration in aged animals, but actively inhibit axon regeneration. While this reviewer raises questions on whether only

senescent Schwann cells or other senescent cells in the nerve contribute to this effect, the identification of potential targets to enhance nerve repair is highly significant. Furthermore, the use of an FDA approved drug, currently in clinicals trials for its application as senotherapeutics, to increase axon regeneration in aged and chronic dennervation conditions will provide new avenues for clinical applications.

Reviewer #2 (Evidence, reproducibility and clarity (Required)):

Summary

The reported study by Fuentes-Flores et al. shows that Schwann cells (SCs) in peripheral nerves undergo senescence with aging or in chronic denervation. This senescent SC phenotype correlates with downregulation of c-Jun expression and axon regeneration capacity and consequently, affecting functional recovery. The study has been undertaken by using in vivo mice model of chronically denervated sciatic nerve and in vitro of rat-primary cell cultures (Schwann cell, DRG-explants, and their coculture). Schwann cell exosome manipulation was also included to exploit their released factor as media for cell cultures.

Major comments:

1. Chronic denervated nerve model and Schwann cell phenotype

*Tibial nerve transection and chronic denervated nerve: As the referee have no information about this model (no reference is cited), a detailed description should be provided highlighting the interest of such model compared to standard sciatic nerve lesion model.

We thank the reviewer for this comment. We will provide a clear explanation for the injury model used, as well as references, including one from the group of Tessa Gordon that describe for the first time this model in rats (PMID: 30215557), and the one applying this model to the mouse from the groups of Rhona Mirsky and Kristjan Jessen (PMID: 33475496). Briefly, this model has two advantages: first, it allows to generate a chronic denervation for months and then be able to connect the distal denervated stump with a proximal one without the need of a nerve bridge; second, neurons in the different groups that have different denervation times (1 week versus 6 weeks) are all damaged at the same time, eliminating variability associated to chronic axonal damage. We will include this information in the result section of a revised manuscript along with the above references.

*Should be shown, histological analysis of denervated tibial branch prior reconnection with freshly cut proximal peroneal branch with specific immunostainings of rSCs v.s. sSC associated with dapi nuclear staining (as used along this study). Specific staining for other cells should be also provided (i.e., macrophage and endothelial cells). In simple words, "how chronically denervated nerve looks like and what is his cellular content? This is necessary to responds to the following main referee question: does the increase/decrease of rSCs or sSC under specific condition all through the study concerns the SCs that have migrated from freshly cut peroneal branch into denervated tibial distal branch, or resident SCs that have survived in chronically denervated tibial distal branch. In other words, whether rSCs that migrate (and accompanying regenerating axons) into chronically denervated branch nerve undergo phenotype change into sSC because of the environment of chronically denervated nerve. This is not clearly described or discussed, and remain confusing for reader.

We are sorry about the lack of clarity in the text and figures. The immunostaining analysis of denervated tibial branch prior reconnection is included in the original manuscript, specifically in Figure 1D-K, and Figure 4. In a revised manuscript we will include schemes in the Figures to shown the regions analyzed in each case.

We thank for the suggestion of including staining for other cell types. As suggested by the reviewer we are currently performing these experiments and analysis for macrophages, endothelial cells and fibroblasts, together with staining for cell senescence (p16), in both aged and chronically denervated conditions. We will include this data in a revised manuscript

About the specific question of the reviewer: "*does the increase/decrease of rSCs or sSC under specific condition all through the study concerns the SCs that have migrated from freshly cut peroneal branch into denervated tibial distal branch, or resident SCs that have survived in chronically denervated tibial distal branch*". Our data demonstrate that senescence Schwann cells appear in the distal nerve stump in aged mice and after chronic denervation. The distal stump is physically disconnected from the proximal part of the nerve. Therefore, after reconnection the regenerating axons encounters a tissue which is already populated with senescent cells. To clearly explain this, we will add extra text in the results and discussion section to clarify these findings.

2. Support of up- and down-regulation of gene expression illustrated in fig.4

The conclusions and statements on up- or down-regulation of c-jun, yH2AX, beta-gal, P16, arise from quantitative and qualitative analysis from immunostaining of these specific markers by determining the number of positive cells rSCs vs. sSC. For these strong statements appropriate methods for quantification of protein levels such as western blots are required. For example, the statement of down regulation of c-jun expression, the quantitative graph shows strong increase in c-jun cell number under ABT263 treatment but the histological photo does not illustrate such decrease in number of the cell. It shows rather an increase in brightness of c-jun staining. Thus, only appropriate method for protein level quantification could be conclusive. This would also remove the doubt that some photos are under- or over- exposed as it appears in the figure. For example, in aged animal under vehicle condition, there is no variability in staining intensity. Accordingly, one question to the authors: for quantification of cell number, are weakly stained cells considered as positive cell?

We agree with the reviewer that a more quantitative method is required to complement the immunofluorescence data. As the reviewer correctly states, quantification of the immunofluorescence data corresponds to cell positive for the specific marker, expressed as % of cell positive for that maker. Therefore, we will perform western blot for c-jun, yH2AX, and p16 for the different models, including treatments with senolytics.

Regarding the method for quantification, we performed all these quantifications using Imaris software, in which we set up the same threshold for all conditions for a specific antibody marker. Then, in addition to the quantitative western blot analysis, we will include a graph representing the distribution of the labelling (intensity histogram) for all cell number quantification from immunofluorescence data, comparing control with the experimental condition. Finally, the methods used for quantification will be expanded in a revised manuscript.

3. The method section should be revised in general

Methods could be described in brief only when are supported by provided refs in which the reader could find details. Several refs are missing, i.e., 4.4 for thermal allodynia, 4.5 for ABT263 gavage administration; senescence induction, ...

Quantification methods should be more detailed, several information are missing and not found in result section or legends (i.e., number of nerve section per animal, neurite length, ...).

We completely agree with the reviewer that the method section was not developed adequately in the original manuscript. As described in previous responses, we will detail the methods used, especially those associated with quantifications performed. We will also include references for the different methods used, including those detailed by the reviewer.

4. The use of rat for in vitro DRG and SC culture while in vivo study is undertaken on mice The switch of species from in vivo to in vitro (mice vs. rat), is not justified as mice DRG and SC culture are also commonly used. In addition, the use of transgenic mice (used here only for in vivo) could also be exploited to address specific and reinforce the data.

We agree with the reviewer that using mouse SC in *in vitro* experiments will be a better approximation to support our findings. We have been using rat SC in this and other publications as they were the standard model used in *in vitro* experiments. Nevertheless, as the author states, now there are suitable methods for culturing mouse SC, that we have incorporated in our lab. Therefore, we will perform key *in vitro* experiments using mouse SC together with mouse DRGs.
Regarding the use of the transgenic mice (3MR), we thank the reviewer for this suggestion; we will perform new experiments using SC derived from 3MR mice in order to demonstrate induction of senescence (by expression of the red fluorescent protein in this transgenic line) and senolysis *in vitro*.

5. Use of conditioned exosome/media

Should be explained why the use of exosomes directly in cell culture was not tested. This would be close to physiological condition, regarding the concentration of released factors.

This is an important point that was not explored. As we have plenty of experience using SC-derived exosomes, we will perform the suggested experiments comparing the effect of exosomes from conditioned media from senescent-induced SC and include the results from these experiments in a revised manuscript.

6. The statement on the effect of rSC vs. sSC cell on growth cone dynamic

The provided data illustrated in fig. 3 are not in support that sSC affect growth cone dynamics. Only what would be "suggested" is that the decrease in neurite length could be associated to changes of growth cone morphology, on fixed tissue, that appeared to be affected. If such statement has to be maintained, time-laps is required. The image does not reflect a retracting neurite nor collapsed growth cone. In addition, other mechanisms could be at the basis of observed decrease in neurite length, which are not evaluated here. This is an important point to address as the authors state that sSC release inhibitory factors.

We completely agree with the reviewer: we are not exploring growth cone dynamics. We will change the manner these results are presented as we are not demonstrating a dynamic process in our results. We prefer to modify the text associated with these experiments rather than perform a time-lapse analysis at this moment. This is part of a future exploration we want to achieve, that will take some time to develop, and we consider at this moment lies outside the scope of the present work. Included in section 4, below.

other comments

- The surgical description is complicated, also annotation to be added in supp fig #2A; provide

We will work in the description of the model, including references to other papers using this nerve anastomosis model for assessing regenerative potential. As stated above we will also include schemes in all figures to help the reader with the surgical procedure and different timelines used.

- M&M 4.2: lign #8, error referring to Fig 2A, correct by. Supp fig 2A

Revisions incorporated in the transferred manuscript, see section 3, below.

- Review refs list, ref#17, full info needed

Revisions incorporated in the transferred manuscript, see section 3, below.

- Fig 3H would be interesting only if contain a column of the 21 proteins exclusively expressed in senescent-induced SCs

Revisions incorporated in the transferred manuscript, see section 3, below.

- The immunostaining of Lamin B1 positive nuclear invaginations in fig S4 should better described in results for non-familiar reader

We will describe better this staining pattern in the result section of the revised manuscript.

- Title of Fig 3, the expression "neuronal growth" is not appropriate here (neurite outgrowth)

Revisions incorporated in the transferred manuscript, see section 3 below.

****Referees cross-commenting****
I agree with referee#1's comments. He/she has raised complementary and important points that should be taken into account by the authors as well as we share the same major concerns. Furthermore, we both expressed the interest of such study if revised appropriately.

Reviewer #2 (Significance (Required)):
Significance
Overall, this is an interesting study that undertake fundamental question in the field of nerve physiopathology and also could open a good opportunity in developing therapeutic strategies for translational research. However, additional investigations are needed to support the main conclusions.

3. Description of the revisions that have already been incorporated in the transferred manuscript

Revision Plan

Reviewer 1

Major comments

• In the axon regeneration assays, how is the reconnection site defined in longitudinal images stained for SCG10? Of particular concern is that Figure 1B "adult chronic dmg" nerve section image appears to be identical to the image in Figure 5A "Vehicle adult (47dpi)". However, the reconnection site is located at different sites along the nerve. Also, the scale bar appears identical but the legend states different sizes. In Figure 1 chronic damage is 42dpi, and figure 5 is 47dpi, yet with what appears as the same image.

We thank the reviewer for detecting this issue. The problem arises as the data shown in Figure 1a corresponds to the controls (vehicle) of Figure 5a. The data in Figure 1 is a known phenomenon in the field of peripheral regeneration (i.e., decreased regeneration in aged animals as well as in chronically denervated nerves); nevertheless, we decided to add this data at the start of the manuscript to clearly shown the reader (thinking in a broader scientific audience) the baseline of the evident decrease in axonal regeneration in these two conditions. To make this very clear, we have included the same image Figure 1 and 5 for the controls and detailed this in the legends of both Figure 1 and 5 in the uploaded manuscript.
We have checked the scales and modify the scale in Figure 5 as it was not correct. We have also corrected the nomenclature for days post injury in this image as well as in the corresponding legend.

In addition, for transparency, we have uploaded in a public repository (EBI BioStudies database, https://www.ebi.ac.uk/biostudies/) all the microscopy images used in this work, which is detailed in the uploaded manuscript in a new section named data availability.

Regarding the localization of the reconnection site, this is identified using the whole z-stack of the nerve and not a single section, the region in the z-stack can be recognized using two parameters: the difference in diameter between the proximal and distal stump and by identifying the filament used to suture both stumps. We have included a description of this procedure in the method section of the revised manuscript. As this is not always a perpendicular line, in the revised Figures we have now used an arrowhead to denote the reconnection site.

We are sorry for the confusion generated by the labeling of some images. We will review the text and figures and fix errors. In a revised manuscript we will also add schemes for several figures in order to explain better the experimental procedure and timelines.

Minor comments

• Improve labeling of Figures or at the very least describe in the Figure legend. For example: Figure 5B-C, which of the graphs is from adult mice and which is from aged mice?

We have included the suggested labelling for Figure 5B-C.

• The images for split individual channels need to match the merge channel images. For example, the individual channel and merge images are not properly aligned in Figure 4C, ABY-263 group.

We thank the reviewer for spotting the error in the split channels, we have now fixed this in Fig 4C, but also corrected other alignment problems detected in Fig 4A and 5F.

• The analysis method used to quantify axon regeneration should be consistent throughout. For example, in Figure 1C, number of axons/nerve width(um) was used for regeneration assay, but axon density (width corrected) was used in Figure 5B-C in regeneration assay.

We have included the procedure used to quantify axonal regeneration in the method section of the uploaded manuscript, which is the same throughout the manuscript. We are sorry for the different texts in the axes of graphs included in Figure 1 and Figure 5. In the first version of the figures, we were using the term “axon density (width corrected)”, but then we decided to change it to “number of axons/nerve width (μm)”, which was more precise. Unfortunately, the text of the graph axis in Figure 5 was not changed by mistake. We have now fix this in the revised version.

Reviewer 2

- M&M 4.2: lign #8, error referring to Fig 2A, correct by. Supp fig 2A

We have corrected this error in the uploaded manuscript.

- Review refs list, ref#17, full info needed

We have fixed this reference in the uploaded manuscript.

- Fig 3H would be interesting only if contain a column of the 21 proteins exclusively expressed in senescent-induced SCs

We are sorry about this omission in Figure 3H. We included a list of all the identified proteins of repair and senescent-induced SCs in Supplementary Table 4 (Table S4) of the original manuscript, including the identity of the 21 proteins exclusively expressed in senescent-induced SCs. In the revised version, we have incorporated the information of the 21 proteins in Figure 3H as suggested by the reviewer.

- Title of Fig 3, the expression "neuronal growth" is not appropriate here (neurite outgrowth)

We thank the reviewer for detecting this error, we have changed the expression as suggested in the uploaded manuscript.

Other changes included in the revised manuscript and Figures

1. Numeric data for graphs
We have included a new supplementary excel file: Supplementary Table 8, including the data for each replicate associated to the graphs of text and supplementary figures.

2. Revision Figure 5G.
During our review on all the individual replicates of the manuscript data to upload into BioStudies, the first author noticed that the data in graph of Figure 5G corresponded to a pilot experiment performed to set up the protocol. The final experiment was not included, then we uploaded the correct images and included the final quantification. Data is comparable, and statistical differences remains.

3. Revision Figure 3A.

We have modified the pseudocolors of the S100 antibody channel from magenta to green for ease of visualization. The image and quantification remain exactly the same.

4. Description of analyses that authors prefer not to carry out

Reviewer 2

6. The statement on the effect of rSC vs. sSC cell on growth cone dynamic

The provided data illustrated in fig. 3 are not in support that sSC affect growth cone dynamics. Only what would be "suggested" is that the decrease in neurite length could be associated to changes of growth cone morphology, on fixed tissue, that appeared to be affected. If such statement has to be maintained, time-laps is required. The image does not reflect a retracting neurite nor collapsed growth cone. In addition, other mechanisms could be at the basis of observed decrease in neurite length, which are not evaluated here. This is an important point to address as the authors state that sSC release inhibitory factors.

We completely agree with the reviewer: we are not exploring growth cone dynamics. We will change the manner these results are presented as we are not demonstrating a dynamic process in our results. We prefer to modify the text associated with these experiments rather than perform a time-lapse analysis at this moment. This is part of a future exploration we want to achieve, that will take some time to develop, and we consider at this moment lies outside the scope of the present work.

29th Apr 2023

Dear Dr. Court,

Thank you for submitting your work to EMBO Molecular Medicine. I have now had the chance to read your manuscript, referee comments, your point-by-point response, and discuss this with the rest of the EMM team. Considering the generally positive reports regarding the interest of this work, we are happy to consider a revised version of your manuscript.

Addressing the reviewers' concerns in full will be necessary for further considering the manuscript in our journal, and acceptance of the manuscript will entail a second round of review. EMBO Molecular Medicine encourages a single round of revision only and therefore, acceptance or rejection of the manuscript will depend on the completeness of your responses included in the next, final version of the manuscript. For this reason, and to save you from any frustrations in the end, I would strongly advise against returning an incomplete revision.

I look forward to seeing a revised form of your manuscript as soon as possible.

Yours sincerely,

Kelly

Kelly M Anderson, PhD
Scientific Editor
EMBO Molecular Medicine

*****

When submitting your revised manuscript, please carefully review the instructions that follow below. We perform an initial quality control of all revised manuscripts before re-review; failure to include requested items will delay the evaluation of your revision.

We require:

1) A .docx formatted version of the manuscript text (including legends for main figures, EV figures and tables). Please make sure that the changes are highlighted to be clearly visible.

2) Individual production quality figure files as .eps, .tif, .jpg (one file per figure). For guidance, download the 'Figure Guide PDF': (https://www.embopress.org/page/journal/17574684/authorguide#figureformat).

3) A .docx formatted letter INCLUDING the reviewers' reports and your detailed point-by-point responses to their comments. As part of the EMBO Press transparent editorial process, the point-by-point response is part of the Review Process File (RPF), which will be published alongside your paper.

4) A complete author checklist, which you can download from our author guidelines (https://www.embopress.org/page/journal/17574684/authorguide#submissionofrevisions). Please insert information in the checklist that is also reflected in the manuscript. The completed author checklist will also be part of the RPF.

5) Please note that all corresponding authors are required to supply an ORCID ID for their name upon submission of a revised manuscript.

6) It is mandatory to include a 'Data Availability' section after the Materials and Methods. Before submitting your revision, primary datasets produced in this study need to be deposited in an appropriate public database, and the accession numbers and database listed under 'Data Availability'. Please remember to provide a reviewer password if the datasets are not yet public (see https://www.embopress.org/page/journal/17574684/authorguide#dataavailability).

In case you have no data that requires deposition in a public database, please state so in this section. Note that the Data Availability Section is restricted to new primary data that are part of this study.

7) For data quantification: please specify the name of the statistical test used to generate error bars and P values, the number (n) of independent experiments (specify technical or biological replicates) underlying each data point and the test used to calculate p-values in each figure legend. The figure legends should contain a basic description of n, P and the test applied. Graphs must include a description of the bars and the error bars (s.d., s.e.m.). See also 'Figure Legend' guidelines:

https://www.embopress.org/page/journal/17574684/authorguide#figureformat

8) At EMBO Press we ask authors to provide source data for the main manuscript figures. Our source data coordinator will contact you to discuss which figure panels we would need source data for and will also provide you with helpful tips on how to upload and organize the files.

9) Our journal encourages inclusion of *data citations in the reference list* to directly cite datasets that were re-used and obtained from public databases. Data citations in the article text are distinct from normal bibliographical citations and should directly link to the database records from which the data can be accessed. In the main text, data citations are formatted as follows: "Data ref: Smith et al, 2001" or "Data ref: NCBI Sequence Read Archive PRJNA342805, 2017". In the Reference list, data citations must be labeled with "[DATASET]". A data reference must provide the database name, accession number/identifiers and a resolvable link to the landing page from which the data can be accessed at the end of the reference. Further instructions are available at .

10) We replaced Supplementary Information with Expanded View (EV) Figures and Tables that are collapsible/expandable online. A maximum of 5 EV Figures can be typeset. EV Figures should be cited as 'Figure EV1, Figure EV2" etc... in the text and their respective legends should be included in the main text after the legends of regular figures.

- For the figures that you do NOT wish to display as Expanded View figures, they should be bundled together with their legends in a single PDF file called *Appendix*, which should start with a short Table of Content. Appendix figures should be referred to in the main text as: "Appendix Figure S1, Appendix Figure S2" etc.

- Additional Tables/Datasets should be labeled and referred to as Table EV1, Dataset EV1, etc. Legends have to be provided in a separate tab in case of .xls files. Alternatively, the legend can be supplied as a separate text file (README) and zipped together with the Table/Dataset file.

See detailed instructions here:

.

11) The paper explained: EMBO Molecular Medicine articles are accompanied by a summary of the articles to emphasize the major findings in the paper and their medical implications for the non-specialist reader. Please provide a draft summary of your article highlighting

- the medical issue you are addressing,

- the results obtained and

- their clinical impact.

This may be edited to ensure that readers understand the significance and context of the research. Please refer to any of our published articles for an example.

12) For more information: There is space at the end of each article to list relevant web links for further consultation by our readers. Could you identify some relevant ones and provide such information as well? Some examples are patient associations, relevant databases, OMIM/proteins/genes links, author's websites, etc...

13) Author contributions: the contribution of every author must be detailed in a separate section (before the acknowledgments).

14) A Conflict of Interest statement should be provided in the main text.

15) Every published paper now includes a 'Synopsis' to further enhance discoverability. Synopses are displayed on the journal webpage and are freely accessible to all readers. They include a short stand first (maximum of 300 characters, including space) as well as 2-5 one-sentences bullet points that summarizes the paper. Please write the bullet points to summarize the key NEW findings. They should be designed to be complementary to the abstract - i.e. not repeat the same text. We encourage inclusion of key acronyms and quantitative information (maximum of 30 words / bullet point). Please use the passive voice. Please attach these in a separate file or send them by email, we will incorporate them accordingly.

Please also suggest a striking image or visual abstract to illustrate your article as a PNG file 550 px wide x 300-600 px high.

EMBO Molecular Medicine has a "scooping protection" policy, whereby similar findings that are published by others during review or revision are not a criterion for rejection. Should you decide to submit a revised version, I do ask that you get in touch after three months if you have not completed it, to update us on the status.

***

Rev_Com_number: RC-2022-01784
New_manu_number: EMM-2023-17907
Corr_author: Court
Title: Senescent Schwann cells induced by aging and chronic denervation impair axonal regeneration after peripheral nerve injury

EMBOMolMEd
Manuscript number: RC-2022-01784
Corresponding author(s): Felipe, Court

Point-by point Reponses in blue

Reviewer #1 (Evidence, reproducibility and clarity (Required)):

Summary
This manuscript by Fuentes-Flores et al reports that elimination of senescent Schwann cells by systemic senolytic drug treatment or genetic targeting improves nerve regeneration and functional recovery in aging and chronic denervation. This improved regeneration is associated with an upregulation of c-Jun expression. Mechanistically the authors provide data to show that senescent Schwann cells secrete factors that are inhibitory to axon regeneration. These findings are very interesting and move the field forward beyond the notion that Schwann cells fail to support axon regeneration in aged animals and identify potential targets to enhance nerve repair. The use of a senolytic drug to increase regeneration in aged and chronic denervation conditions provides new avenues for clinical interventions. However, some of the claims are overstated since this is not the first characterization of senescent Schwann cells and the manipulations used in the study are not entirely specific for Schwann cells. The manuscript is also poorly written and difficult to follow, given the complex set of surgeries and terminology, and lack of explanation of the rationale for the surgery model used. Figures are poorly labeled and difficult to follow without figure legends, and figure legends do not match the figures.

We thank the reviewer for the positive comments, we also thanks for the careful identification of format problems in the manuscript, including the lack of a clear explanation of the complex procedures used. We have now worked very carefully on the format, including clear explanations of the procedures and new schemes added to most of the figures to complement the text. As detailed below, we have performed all the experimental work proposed by the reviewer, which have strengthened the conclusions of this manuscript.
In the revised manuscript we have highlighted in yellow all changes detailed below.

Below are suggestions for improvements:

Major comments:

• In the axon regeneration assays, how is the reconnection site defined in longitudinal images stained for SCG10? Of particular concern is that Figure 1B "adult chronic dmg" nerve section image appears to be identical to the image in Figure 5A "Vehicle adult (47dpi)". However, the reconnection site is located at different sites along the nerve. Also, the scale bar appears identical but the legend states different sizes. In Figure 1 chronic damage is 42dpi, and figure 5 is 47dpi, yet with what appears as the same image.

We thank the reviewer for detecting this issue. The problem arises as the data shown in Figure 1a corresponds to the controls (vehicle) of Figure 5a. The data in Figure 1 is a known phenomenon in the field of peripheral regeneration (i.e., decreased regeneration in aged animals as well as in chronically denervated nerves); nevertheless, we decided to add this data at the start of the manuscript to clearly shown the reader (thinking in a broader scientific audience) the clear decrease in axonal regeneration in these two conditions. <u>To make this very clear, we have now included the same image in Figure 1 and 5</u>

for the controls and detailed this in the legends of both Figure 1 and 5 in the uploaded manuscript. In addition, for transparency, we have uploaded in a public repository (EBI BioStudies database, https://www.ebi.ac.uk/biostudies/) all the microscopy images used in this work, which is detailed in the uploaded manuscript in a new section named data availability.

We have now checked scale bars in all panels and modify the scale in Figure 5 as it was not correct. We have also corrected the nomenclature for days post injury in this image as well as in the corresponding legend.

Regarding the localization of the reconnection site, this is identified using the whole z-stack of the nerve and not a single section, the region in the z-stack can be recognized using two parameters: the difference in diameter between the proximal and distal stump and by identifying the filament used to suture both stumps. We have included a description of this procedure in the method section of the revised manuscript. As this is not always a perpendicular line, in the revised Figures we have now used an arrowhead to denote the reconnection site.

We are sorry for the confusion generated by the labeling of some images. We have now reviewed the text and figures and fix the detected errors. In the revised manuscript we have now included schemes in Figure 1, 4 and 5 in order to explain better the experimental procedure and timelines associated to the experiments performed *in vivo*.

• The authors need to provide a rationale for the choice of this complex injury model and what are the advantages over other models. Please clearly describe the timepoints for each experiment and why the time points were chosen for analysis. Provide the scheme of injury in the main Figures to ease comprehension. A scheme is provided in what appears to be Figure S2, but the legend of Figure S2 does not match. Please compare same time points between aged and adults. Days post injury is sometimes referred to 7 or 42, and it is difficult to follow if it is days post initial transection of the tibial nerve or days post reconnection of transected tibial to peroneal. Revise all Figure legends and supplementary Figure legends to match figures.

We thank the reviewer for this comment. In the revised manuscript we have now provided in the result section a clear explanation for the injury model used, included the reference of this method for mouse shown by the groups of Rhona Mirsky and Kristjan Jessen (PMID: 33475496). As stated now in the revised manuscript, this model has two advantages: first, it allows to generate chronic denervation for months and then be able to connect the distal denervated stump with a proximal one without the need of a nerve bridge; And second, neurons in the different groups that have different denervation times (1 week versus 6 weeks) are all damaged at the same time, eliminating variability associated to chronic axonal (neuronal) damage.

As suggested by the reviewer, we have now included schemes of the injuries performed in each experiment in Figure 1, 4 and 5, also adding a timeline for all complex procedures. This is an excellent suggestion to clearly understand the different procedures performed. We have also checked all figures and legends, and corrected minor errors detected, including the problem detected by the reviewer (legends of Figures 2 and 5 were swapped). We understand the problem of referring to days post-injury, then we have now introduced a new form of referring to the initial transection, and reconnection, which is detailed in the results section referring to the new panel A in Figure 1, an scheme that is used throughout the manuscript to explain the different procedures. Adding schemes per figure will also help to understand the different timelines used in different experiments, including the ones using senolytics.

We have also performed a very careful revision of the text and legends for consistency, all changes have been highlighted in yellow in the revised manuscript.

• The authors' main conclusion is that Senescent Schwann cells inhibit axon regeneration. The authors need to tune down this statement and acknowledge that their manipulations are not entirely Schwann cells specific. While the data nicely shows a contribution of senescent Schwann cells, it does not sufficiently acknowledge the possibility that other senescent cells in the nerve contribute to this effect. First, the authors refer in the discussion that 60% of the senescent cells are SOX10 negative, and thus represent other cells beyond Schwann cells. This quantification needs to be shown in Figure 1. Second, the genetic and pharmacology manipulation eliminate all senescent cells, including Schwann cells. Third, while the culture experiment may be Schwann cells specific, the authors need to provide detailed information on how they purify these cells, how they induce repair Schwann cells (rSC) as claimed in Figure 3, and demonstrate whether these are pure Schwann cells. This is important because other cells in the nerve contribute to nerve repair, including mesenchymal cells. Finally, the claim that using Mpz-cre will lead to c-jun overexpression only in SC also needs to be demonstrated, since Mpz is also expressed in satellite glial cells in the DRG.

We thank the reviewer for these comments and suggestions. We have now toned down the statement that senescence Schwann cells are the only cell candidates for modulating regeneration, including new data in Figure 1 (panel J) and text in the result and discussion section as detailed below.

We have now performed a detailed quantification of other populations of senescent cells that appear in chronic denervation and aging after nerve damage (as well as in non-damaged conditions in adult and aged mice). To this end, we have used specific markers for macrophages, endothelial cells and fibroblast, in addition to Schwann cells, together with the senescent cell marker p16. We have included this data in a revised Figure 1, panel J, as well as a new supplementary Figure 2 containing the immunofluorescence staining for each cell type together with p16 and DAPI nuclei staining. These results are presented in the result section but also discussed with new text in the discussion section.

Regarding *in vitro* experiments, we have now included detailed methods for Schwann cell purification, and induction of the rSC phenotype. Related to the purity of these cultures, we have now included in the method section the purity of our cultures, which for rat Schwann cells corresponds to 99,3% ± 0,2% (Mean ± SD, N=3).

Related to c-jun overexpression in the Mpz-cre, we agree with the reviewer that there is probably overexpression in satellite glial cells in the DRG. We have now included this information in the result section (section 2.1) of the revised manuscript. Satellite glial cells (SGCs) are a subset of cells in the Schwann cell (SC) lineage that express several early myelination markers, such as Mbp, Mag, and Plp, and the transcription factor Sox10. SGCs express early SC markers, such as CDH19, and are transcriptionally and morphologically similar to SCs, even in the absence of axonal contact. Regarding the possibility that SGCs are contributing to the enhanced regeneration presented by mice with c-jun overexpression, this issue was somehow approached previously by Wagstaff et al. (PMID: 33475496), as they showed that in this mouse strain, increased axonal regeneration was equally observed in sensory neurons, in contact with SGCs, and motor neurons, which are not associated to SGCs. This observation suggests that the effect is associated with c-jun overexpressing Schwann cells in the distal stump. In addition, in our work, the changes in senescent cells observed in the c-jun overexpressed mouse, were associated to the distal nerve stump, which was mechanically separated from the proximal region.

• In Figure 5, c-jun is shown after denervation (42 dpi). The results describe 28 days of denervation, 5 days of GCV and 7 days post reconnection, which makes 40 days. If that is not the case, results need to better explain timeline of this procedure. Also, what is the basal c-jun expression in p16-3MR mice? In addition to the number of c-jun positive cells shown in Figure 5G, the authors need to quantify the percent of c-jun puncta that co-localize with Sox10. The size of the c-jun puncta appears different in size in vehicle and GCV, is that an expected phenotype?

We are sorry about these problems in the nomenclature. We have now reviewed all the manuscript for consistency and we have corrected the errors detected by the reviewer. We have now included schemes and timelines for all the surgical experiments in the revised manuscript (in Figures 1, 4 and 5), including detailed information for the experiments using the p16-3MR mice (Figure 5A).

Regarding c-Jun expression in the p16-3MR mice, we have performed the suggested control in basal conditions. Using Western blot and densitometric analysis we show in Supplementary Figure 8B that the basal c-Jun expression is comparable between p16-3MR and wild type mice.

We have checked c-Jun puncta in Figure 5G without a clear tendency or significative difference between vehicle and GCV-treated mice.

Minor comments

• Improve labeling of Figures or at the very least describe in the Figure legend. For example: Figure 5B-C, which of the graphs is from adult mice and which is from aged mice?

We are sorry about the lack of clear labeling in the figures; we have now carefully reviewed all figures in the manuscript and their corresponding legends, including adding better labeling to all graphs in Figure 5.

• The authors need to carefully describe where the high magnification images were taken in the injured nerve and keep the comparison at same site between groups. Please check the scale bar for each image. For example, the images in Figure 1D/F/I/K used same scale, but the cell size and cell morphology are different. The images for split individual channels need to match the merge channel images. For example, the individual channel and merge images are not properly aligned in Figure 4C, ABY-263 group.

We have now included in the method section of the revised manuscript a text describing the region in which the high magnification images were took, which always corresponds to a fixed distance distal to the injury/reconnection site: *Images were taken approximately 200~300 mm from the transection site on a Leica SPEII confocal microscope.*

We have checked scale bars for all microscopy images, and corrected the problem detected by the reviewer in Figure 1 but also some other small error in other Figures, we are sorry for this problem in scale bars, but is now carefully reviewed and corrected.

We thank the reviewer for spotting the error in the split channels, we have now fixed this in Fig 4C, but also corrected other alignment problems detected in Fig 4A and 5F.

• The analysis method used to quantify axon regeneration should be consistent throughout. For example, in Figure 1C, number of axons/nerve width(um) was used for regeneration assay, but axon density (width corrected) was used in Figure 5B-C in regeneration assay.

We have included the procedure used to quantify axonal regeneration in the method section of the uploaded manuscript, which is the same throughout the manuscript. We are sorry for the different texts in the axes of graphs included in Figure 1 and Figure 5. In the first version of the figures, we were using the term “axon density (width corrected)”, but then we decided to change it to “number of axons/nerve width (μm)”, which was more precise but both indicates the same analytical method. Unfortunately, the text of the graph axis in Figure 5 was not changed by mistake. We have now fix this in the revised version.

****Referees cross-commenting****
I agree with all Referee #2's comments. Both sets of comments are important, complementary and point to the same major concerns that need to be addressed. Agree as well that both reviewer think this is an interesting and relevant study for the field of nerve repair, if revised appropriately.

Reviewer #1 (Significance (Required)):
Significance
The findings reported in this manuscript are very interesting and will move the field of nerve repair forward. This paper will be of interest for basic science audience in the fields of aging and neurobiology and has also potential interest to the broader clinical and translational fields. Indeed, this paper provides data that Schwann cells entering a senescent stage not only fail to support axon regeneration in aged animals, but actively inhibit axon regeneration. While this reviewer raises questions on whether only senescent Schwann cells or other senescent cells in the nerve contribute to this effect, the identification of potential targets to enhance nerve repair is highly significant. Furthermore, the use of an FDA approved drug, currently in clinicals trials for its application as senotherapeutics, to increase axon regeneration in aged and chronic dennervation conditions will provide new avenues for clinical applications.

Reviewer #2 (Evidence, reproducibility and clarity (Required)):

Summary

The reported study by Fuentes-Flores et al. shows that Schwann cells (SCs) in peripheral nerves undergo senescence with aging or in chronic denervation. This senescent SC phenotype correlates with downregulation of c-Jun expression and axon regeneration capacity and consequently, affecting functional recovery. The study has been undertaken by using in vivo mice model of chronically denervated sciatic nerve and in vitro of rat-primary cell cultures (Schwann cell, DRG-explants, and their coculture). Schwann cell exosome manipulation was also included to exploit their released factor as media for cell cultures.

We thank the reviewer for the positive comments on our manuscript and for a detailed review of our work. We have now performed all the experiments suggested by the reviewer. Below is a detailed response to each issue raised by the reviewer.
In the revised manuscript we have highlighted in yellow all changes detailed below.

Major comments:

1. Chronic denervated nerve model and Schwann cell phenotype

*Tibial nerve transection and chronic denervated nerve: As the referee have no information about this model (no reference is cited), a detailed description should be provided highlighting the interest of such model compared to standard sciatic nerve lesion model.

We thank the reviewer for this comment. In the revised manuscript we have now provided in the result section a clear explanation for the injury model used, included the reference of this method for mouse shown by the groups of Rhona Mirsky and Kristjan Jessen (PMID: 33475496). Briefly, this model has two advantages: first, it allows to generate chronic denervation for months and then be able to connect the distal denervated stump with a proximal one without the need of a nerve bridge; And second, neurons in the different groups that have different denervation times (1 week versus 6 weeks) are all damaged at the same time, eliminating variability associated to chronic neuronal damage.

*Should be shown, histological analysis of denervated tibial branch prior reconnection with freshly cut proximal peroneal branch with specific immunostainings of rSCs v.s. sSC associated with dapi nuclear staining (as used along this study). Specific staining for other cells should be also provided (i.e., macrophage and endothelial cells). In simple words, "how chronically denervated nerve looks like and what is his cellular content? This is necessary to responds to the following main referee question: does the increase/decrease of rSCs or sSC under specific condition all through the study concerns the SCs that have migrated from freshly cut peroneal branch into denervated tibial distal branch, or resident SCs that have survived in chronically denervated tibial distal branch. In other words, whether rSCs that migrate (and accompanying regenerating axons) into chronically denervated branch nerve undergo phenotype change into sSC because of the environment of chronically denervated nerve. This is not clearly described or discussed, and remain confusing for reader.

We are sorry about the lack of clarity in the text and figures. The immunostaining analysis of denervated tibial branch prior to reconnection is included in the original manuscript, specifically in Figure 1E-F, and all images shown in Figure 4. In the revised manuscript we have now included schemes in the Figures to shown the regions analyzed in each case, this not only for Figure 1 but also for experimental procedures shown in Figure 4 and 5, together with timelines for these experiments.

We have now performed a detailed quantification of other populations of senescent cells that appear in chronic denervation and aging after nerve damage (as well as in non-damaged conditions in adult and aged mice). To this end, we have used specific markers for macrophages, endothelial cells and fibroblast, in addition to Schwann cells, together with the senescent cell marker p16. We have included this data in a revised Figure 1, panel J, as well as a new supplementary Figure 2 containing the immunofluorescence staining for each cell type together with p16 and DAPI nuclei staining. These results are presented in the result section but also discussed with new text in the discussion section.

About the specific question of the reviewer: "*does the increase/decrease of rSCs or sSC under specific condition all through the study concerns the SCs that have migrated from freshly cut peroneal branch into denervated tibial distal branch, or resident SCs that have survived in chronically denervated tibial distal branch*". Our data demonstrate that senescence Schwann cells appear in the distal nerve stump in aged mice and after chronic denervation. After nerve transection, the distal stump is physically disconnected from the proximal part of the nerve. Therefore, after reconnection the regenerating axons encounters a tissue which is already populated with senescent cells. We have now included text explained this in the result and discussion section of the revised manuscript.

2. Support of up- and down-regulation of gene expression illustrated in fig.4

The conclusions and statements on up- or down-regulation of c-jun, yH2AX, beta-gal, P16, arise from quantitative and qualitative analysis from immunostaining of these specific markers by determining the number of positive cells rSCs vs. sSC. For these strong statements appropriate methods for quantification of protein levels such as western blots are required. For example, the statement of down regulation of c-jun expression, the quantitative graph shows strong increase in c-jun cell number under ABT263 treatment but the histological photo does not illustrate such decrease in number of the cell. It shows rather an increase in brightness of c-jun staining. Thus, only appropriate method for protein level quantification could be conclusive. This would also remove the doubt that some photos are under- or over- exposed as it appears in the figure. For example, in aged animal under vehicle condition, there is no variability in staining intensity. Accordingly, one question to the authors: for quantification of cell number, are weakly stained cells considered as positive cell?

We agree with the reviewer that a more quantitative method will nicely complement the immunofluorescence data. As the reviewer correctly states, quantification of the immunofluorescence data corresponds to cell positive for the specific marker, expressed as % of cell positive for that maker. We have now performed Western blot analysis for the senescent marker p16, demonstrating that at the protein level p16 increases after chronic denervation in adult animals, and decreases almost 3 times after treatment with the senolytic ABT-263, which roughly corresponds to the results obtained by immunofluorescence analysis. This data has been included in the revised manuscript and in Figure 4J. We have also used Western blot analysis for c-Jun, demonstrating that it decreases after chronic denervation in adult animals or acute denervation in aged mice (Figure 1C). In addition, using this technique we have found that after senolytic treatment with ABT-263, c-Jun increases significatively (Figure 4K). We thanks the reviewer for this suggestion as now a second analytical technique support our finding.

Regarding the method for quantification, we performed all these quantifications using Imaris software as detailed in the method section, in which we set up the same threshold for all conditions for a specific antibody marker. We have now included as supplementary information (Supplementary Figure 9) the distribution of the label (intensity histogram) in individual nuclei cells for the different markers and

conditions, comparing the control and the experimental distribution. Finally, the methods used for quantification has been detailed in the methods section of the revised manuscript.

3. The method section should be revised in general

Methods could be described in brief only when are supported by provided refs in which the reader could find details. Several refs are missing, i.e., 4.4 for thermal allodynia, 4.5 for ABT263 gavage administration; senescence induction, ...
Quantification methods should be more detailed, several information are missing and not found in result section or legends (i.e., number of nerve section per animal, neurite length, ...).

We completely agree with the reviewer that the method section was not developed adequately in the original manuscript. We have now detailed the methods used, especially those associated with the quantifications performed. We have also included in the legends, detailed information of the group numbers and statistics performed. Importantly, we have now included references for the different methods used, including those methods detailed by the reviewer.

4. The use of rat for in vitro DRG and SC culture while in vivo study is undertaken on mice The switch of species from in vivo to in vitro (mice vs. rat), is not justified as mice DRG and SC culture are also commonly used. In addition, the use of transgenic mice (used here only for in vivo) could also be exploited to address specific and reinforce the data.

We agree with the reviewer that using mouse SC in *in vitro* experiments will be a better approximation to compare with the *in vivo* data. We have been using rat SC in this and other publications as they were the standard model used in *in vitro* experiments. We have now incorporated methods for mouse SC culture to approach the reviewer issue. We have performed the most important results from the *in vitro* model using primary cultures of mouse Schwann cells. We have been able to induce mouse rSC into a senescent phenotype by using doxorubicin, showing that they express markers of cell senescence. In addition, mouse senescent cells (as the rat siSC) also shown a decrease in the expression of c-Jun. This data is now in Supplementary Figure 6 of the revised manuscript. Furthermore, conditioned media from mouse siSC inhibited axonal growth of mouse DRG explants, and modify the growth cone morphology, this data has been included in Supplementary Figure 7 of the revised manuscript.
Regarding the use of the transgenic mice (3MR), we thank the reviewer for this suggestion; we have purified primary cultures of SCs derived from 3MR mice and induce senescence using doxorubicin. As expected, these senescence-induced SCs express red fluorescent protein (RFP; in the p16-3MR, senescence induction triggers the expression of RFP), and they are eliminated after ganciclovir treatment. These data have been incorporated in the revised manuscript in the result section and in Supplementary Figure 8. These results better define that the *in vitro* model induces SCs in to a *bona fide* p16 senescent phenotype. In addition, it reinforces the *in vivo* data demonstrating elimination of senescent Schwann cells *in vivo* in the 3MR mouse model after ganciclovir treatment.

5. Use of conditioned exosome/media

Should be explained why the use of exosomes directly in cell culture was not tested. This would be close to physiological condition, regarding the concentration of released factors.

We have now tested if exosomes derived from conditioned media of senescent Schwann cells have an effect over axonal growth. To our surprise, purified exosomes do not inhibit axonal growth which suggests that is another soluble factor that is associated to this effect. This is very interesting taking into account

that the proteomic results from the conditioned media have several candidates that might be responsible. We understand it will be important to search for the mechanisms associated to this effect, that is something we are starting to perform, but it could take some time to pinpoint the mechanisms associated to this senescent-induced growth inhibition.

6. The statement on the effect of rSC vs. sSC cell on growth cone dynamic

The provided data illustrated in fig. 3 are not in support that sSC affect growth cone dynamics. Only what would be "suggested" is that the decrease in neurite length could be associated to changes of growth cone morphology, on fixed tissue, that appeared to be affected. If such statement has to be maintained, time-laps is required. The image does not reflect a retracting neurite nor collapsed growth cone. In addition, other mechanisms could be at the basis of observed decrease in neurite length, which are not evaluated here. This is an important point to address as the authors state that sSC release inhibitory factors.

We completely agree with the reviewer that with the experiments provided we are not exploring growth cone dynamics. We have now changed the manner these results are presented as we are not demonstrating a dynamic process in our results. We prefer to modify the text associated with these experiments rather than perform time-lapse analysis at this moment. This is part of a future exploration we want to achieve, that will take some time to develop, and we consider at this moment this lies outside the scope of the present work.

other comments

- The surgical description is complicated, also annotation to be added in supp fig #2A; provide

We have now included a better description of the surgical procedure together with a more detailed scheme (originally in supplementary Figure 2) as a new panel in Figure 1. In addition, timelines for the *in vivo* procedures have been included in Figure 1, 4 and 5.

- M&M 4.2: lign #8, error referring to Fig 2A, correct by. Supp fig 2A

We have corrected this error in the uploaded manuscript.

- Review refs list, ref#17, full info needed

We have corrected this error in the uploaded manuscript.

- Fig 3H would be interesting only if contain a column of the 21 proteins exclusively expressed in senescent-induced SCs

We are sorry about this omission in Figure 3. In the revised version, we have incorporated the information of the 21 proteins in the revised Figure 3J as suggested by the reviewer. We have also included a list of all the identified proteins of repair and senescent-induced SCs in Supplementary Table 4 (Table S4) of the manuscript, including the identity of the 21 proteins exclusively expressed in senescent-induced SCs.

- The immunostaining of Lamin B1 positive nuclear invaginations in fig S4 should better described in results for non-familiar reader

We have now described this staining pattern in the result section of the revised manuscript and included a reference (reference 42 of the revised manuscript).

- Title of Fig 3, the expression "neuronal growth" is not appropriate here (neurite outgrowth)

We thank the reviewer for detecting this error, we have changed the expression as suggested in the uploaded manuscript.

Referees cross-commenting
I agree with referee#1's comments. He/she has raised complementary and important points that should be taken into account by the authors as well as we share the same major concerns. Furthermore, we both expressed the interest of such study if revised appropriately.

Reviewer #2 (Significance (Required)):
Significance
Overall, this is an interesting study that undertake fundamental question in the field of nerve physiopathology and also could open a good opportunity in developing therapeutic strategies for translational research. However, additional investigations are needed to support the main conclusions.

Other changes included in the revised manuscript and Figures not requested by reviewers

1. Numeric data for graphs
We have included a new supplementary excel file: Supplementary Table 8, including the data for each replicate associated to the graphs of text and supplementary figures.

2. Revision Figure 5G.
During our review on all the individual replicates of the manuscript data to upload into BioStudies, the first author noticed that the data in graph of Figure 5G corresponded to a pilot experiment performed to set up the protocol. The final experiment was not included, then we uploaded the correct images and included the final quantification. Data is comparable, and statistical differences remains.

3. Revision Figure 3A.
We have modified the pseudocolors of the S100 antibody channel from magenta to green for ease of visualization. The image and quantification remain exactly the same.

23rd Aug 2023

Dear Felipe,

Congratulations on a great revision! Overall, the referee was positive. However, there remain a few editorial items that we ask you to attend to in a revised version.

1. Please add up to five keywords, which may or may not appear in the title, should be given in alphabetical order, below the abstract, each separated by a slash (/).

2. Please resolve this discrepancy in an author name: Manuscript: David Ñecuñir vs. in eJP: David Necuñir-Ibarra

3. Please add the following funding to eJP online: NCRR shared instrumentation grant 1S10 OD028654

4. Please review our formatting requirements for references online. After 10 author names, et al should be used and these should be listed alphabetically.

5. Please review our new policy on conflict of interests on the EMBO author guide website and update the title of this section to: Disclosure and competing interests statement

6. There is space at the end of each article to list relevant web links for further consultation by our readers. Could you identify some relevant ones and provide such information as well? Some examples are patient associations, relevant databases, OMIM/proteins/genes links, author's websites, etc. This section is called "For more information" and can be useful for readers, but is not required.

7. EMBO Molecular Medicine articles are accompanied by a structured summary of the article to emphasize the major findings of the paper and their medical implications for the non-specialist reader called "The Paper Explained". Please provide a summary accessible to non-specialists and specialists alike, highlighting the medical issue you are addressing (heading: PROBLEM), the results obtained (heading: RESULTS), and their clinical impact (heading: IMPACT). This may be edited to ensure that readers understand the significance and context of the research. Please refer to any of our published primary research articles for an example.

8. Please rename Table S5 and S6 to EV TableS5 and S6 and upload as Expanded view. Table S3, S4, and S7 should be uploaded as datasets and named Dataset EV#. Table S1 and S2 have multiple columns so should also be uploaded as Datasets and the associated callouts in the main manuscript should be updated as well.

9. We include a synopsis of the paper (see http://emboj.embopress.org/). Please provide me with a general summary statement and 3-5 bullet points that capture the key findings of the paper.

10. We also need a summary figure for the synopsis. The size should be 550 wide by 200-440 high (pixels). You can also use something from the figures if that is easier.

11. All figures must be referred to in the main manuscript and in chronological order. Please add a call out to Table S5 and S7 in the manuscript text.

12. Please move the Figure legends, they should go after the References section.

13. There are 9 Suppl. figures uploaded and a file with Suppl. figure legends; you can have up to 5 EV figures upldd as figure files and the corresponding legends in the main ms included below the main figure legends; the remaining figures should be renamed to Appendix Figure S# and should be provided in an Appendix file with a Table of contents and page numbers
I thank you for the opportunity to consider your work for publication and I look forward to your revision.

Kind regards,
Kelly

Kelly M Anderson, PhD
Scientific Editor
EMBO Molecular Medicine

*** Instructions to submit your revised manuscript ***

*** PLEASE NOTE *** As part of the EMBO Publications transparent editorial process initiative (see our Editorial at https://www.embopress.org/doi/pdf/10.1002/emmm.201000094), EMBO Molecular Medicine will publish online a Review Process File to accompany accepted manuscripts.

In the event of acceptance, this file will be published in conjunction with your paper and will include the anonymous referee reports, your point-by-point response and all pertinent correspondence relating to the manuscript. If you do NOT want this file to be published, please inform the editorial office at contact@embomolmed.org.

***** Reviewer's comments *****

Referee #2 (Comments on Novelty/Model System for Author):

This is a revised version of an interesting study with potential clinical relevance. Scientific novelty and relevance have been acknowledged by both previous referees and are strengthened significantly by the additional data supplied and the improved descriptions and discussion.

Referee #2 (Remarks for Author):

The manuscript has been greatly improved by the inclusion of additional evaluations and data and by the in-depth revision of the text in all parts, incl. Fig. legends and Methods. The requests for changes, additions and clarifications by both previous referees have been fulfilled for each point of the referee's comments.

The authors addressed the remaining minor issues.

18th Sep 2023

Dear Felipe,

Congratulations on an excellent manuscript, I am pleased to inform you that your manuscript has been accepted for publication in EMBO Molecular Medicine. Thank you for your comprehensive response to the referee concerns and for providing detailed source data. It has been a pleasure to work with you to get this to the acceptance stage.

I will begin the final checks on your manuscript before submitting to the publisher next week. Once at the publisher, it will take about 3 weeks for your manuscript to be published online. As a reminder, the entire review process including referee concerns and your point-by-point response will be available to readers.

I will be in touch throughout the final editorial process until publication. In the meantime, I hope you find time to celebrate!

Yours sincerely,
Kelly

Kelly M Anderson, PhD
Scientific Editor
EMBO Molecular Medicine
Follow us on Twitter @EmboMolMed

*** *** *** IMPORTANT INFORMATION *** *** ***

Your article cannot be published until the publisher has received the appropriate signed license agreement. Once your article has been received by Wiley for production you will receive an email from Wiley's Author Services system, which will ask you to log in and will present you with the appropriate license for completion.

Should you be planning a Press Release on your article, please get in contact with embomolmed@wiley.com as early as possible, in order to coordinate publication and release dates.

PROOFS

You will receive the proofs by e-mail approximately 2 weeks after all relevant files have been sent to our Production Office. Please return them within 48 hours and if there should be any problems, please contact the production office at embopressproduction@wiley.com.

Please inform us if there is likely to be any difficulty in reaching you at the above address at that time. Failure to meet our deadlines may result in a delay of publication.

All further communications concerning your paper proofs should quote reference number EMM-2023-17907-V3 and be directed to the production office at embopressproduction@wiley.com.

EMBO Press Author Checklist

Corresponding Author Name: Felipe Court	
Journal Submitted to: EMBO Molecular Medicine	
Manuscript Number: EMM-2023-17907-V2	

USEFUL LINKS FOR COMPLETING THIS FORM
The EMBO Journal - Author Guidelines
EMBO Reports - Author Guidelines
Molecular Systems Biology - Author Guidelines
EMBO Molecular Medicine - Author Guidelines

Reporting Checklist for Life Science Articles (updated January
This checklist is adapted from Materials Design Analysis Reporting (MDAR) Checklist for Authors. MDAR establishes a minimum set of requirements in transparent reporting in the life sciences (see Statement of Task: 10.31222/osf.io/9sm4x). Please follow the journal's guidelines in preparing your
Please note that a copy of this checklist will be published alongside your article.

Abridged guidelines for figures
1. Data
The data shown in figures should satisfy the following conditions:
- the data were obtained and processed according to the field's best practice and are presented to reflect the results of the experiments in an accurate and unbiased manner.
- ideally, figure panels should include only measurements that are directly comparable to each other and obtained with the same assay.
- plots include clearly labeled error bars for independent experiments and sample sizes. Unless justified, error bars should not be shown for technical
- if n<5, the individual data points from each experiment should be plotted. Any statistical test employed should be justified.
- Source Data should be included to report the data underlying figures according to the guidelines set out in the authorship guidelines on Data

2. Captions
Each figure caption should contain the following information, for each panel where they are relevant:
- a specification of the experimental system investigated (eg cell line, species name).
- the assay(s) and method(s) used to carry out the reported observations and measurements.
- an explicit mention of the biological and chemical entity(ies) that are being measured.
- an explicit mention of the biological and chemical entity(ies) that are altered/varied/perturbed in a controlled manner.
- the exact sample size (n) for each experimental group/condition, given as a number, not a range;
- a description of the sample collection allowing the reader to understand whether the samples represent technical or biological replicates (including how many animals, litters, cultures, etc.).
- a statement of how many times the experiment shown was independently replicated in the laboratory.
- definitions of statistical methods and measures:
  - common tests, such as t-test (please specify whether paired vs. unpaired), simple χ^2 tests, Wilcoxon and Mann-Whitney tests, can be unambiguously identified by name only, but more complex techniques should be described in the methods section;
  - are tests one-sided or two-sided?
  - are there adjustments for multiple comparisons?
  - exact statistical test results, e.g., P values = x but not P values < x;
  - definition of 'center values' as median or average;
  - definition of error bars as s.d. or s.e.m.

Please complete ALL of the questions below.
Select "Not Applicable" only when the requested information is not relevant for your study.

Materials

Newly Created Materials	Information included in the manuscript?	In which section is the information available? (Reagents and Tools Table, Materials and Methods, Figures, Data Availability Section)
New materials and reagents need to be available; do any restrictions apply?	Not Applicable	

Antibodies	Information included in the manuscript?	In which section is the information available? (Reagents and Tools Table, Materials and Methods, Figures, Data Availability Section)
For **antibodies** provide the following information:<br>- Commercial antibodies: RRID (if possible) or supplier name, catalogue number and or/clone number<br>- Non-commercial: RRID or citation	Yes	Reagents tools table

DNA and RNA sequences	Information included in the manuscript?	In which section is the information available? (Reagents and Tools Table, Materials and Methods, Figures, Data Availability Section)
**Short novel DNA or RNA including primers, probes**: provide the sequences.	Yes	TableS5_Fuentes_taqmanArray

Cell materials	Information included in the manuscript?	In which section is the information available? (Reagents and Tools Table, Materials and Methods, Figures, Data Availability Section)
**Cell lines:** Provide species information, strain. Provide accession number in repository **OR** supplier name, catalog number, clone number, and/**OR** RRID.	Yes	Material and Methods
**Primary cultures:** Provide species, strain, sex of origin, genetic modification status.	Yes	Material and Methods
Report if the cell lines were recently **authenticated** (e.g., by STR profiling) and tested for mycoplasma contamination.	Not Applicable	

Experimental animals	Information included in the manuscript?	In which section is the information available? (Reagents and Tools Table, Materials and Methods, Figures, Data Availability Section)
**Laboratory animals or Model organisms:** Provide species, strain, sex, age, genetic modification status. Provide accession number in repository **OR** supplier name, catalog number, clone number, **OR** RRID.	Yes	Material and Methods
**Animal observed in or captured from the field:** Provide species, sex, and age where possible**.**	Not Applicable	
Please detail **housing and husbandry conditions**.	Yes	Material and Methods

Plants and microbes	Information included in the manuscript?	In which section is the information available? (Reagents and Tools Table, Materials and Methods, Figures, Data Availability Section)
**Plants:** provide species and strain, ecotype and cultivar where relevant, unique accession number if available, and source (including location for collected wild specimens).	Not Applicable	
**Microbes:** provide species and strain, unique accession number if available, and source.	Not Applicable	

Human research participants	Information included in the manuscript?	In which section is the information available? (Reagents and Tools Table, Materials and Methods, Figures, Data Availability Section)
If collected and within the bounds of privacy constraints report on age, sex and gender or ethnicity for all study participants.	Not Applicable	

Core facilities	Information included in the manuscript?	In which section is the information available? (Reagents and Tools Table, Materials and Methods, Figures, Data Availability Section)
If your work benefited from core facilities, was their service mentioned in the acknowledgments section?	Not Applicable	

Design

Study protocol	Information included in the manuscript?	In which section is the information available? (Reagents and Tools Table, Materials and Methods, Figures, Data Availability Section)
If study protocol has been **pre-registered, provide DOI in the manuscript**. For clinical trials, provide the trial registration number **OR** cite DOI.	Not Applicable	
Report the **clinical trial registration number** (at ClinicalTrials.gov or equivalent), where applicable.	Not Applicable	

Laboratory protocol	Information included in the manuscript?	In which section is the information available? (Reagents and Tools Table, Materials and Methods, Figures, Data Availability Section)
Provide DOI OR other citation details if **external detailed step-by-step protocols** are available.	Yes	Material and Methods and References

Experimental study design and statistics	Information included in the manuscript?	In which section is the information available? (Reagents and Tools Table, Materials and Methods, Figures, Data Availability Section)
Include a statement about **sample size** estimate even if no statistical methods were used.	Yes	Figures and Figure captions
Were any steps taken to minimize the effects of subjective bias when allocating animals/samples to treatment (e.g. **randomization procedure**)? If yes, have they been described?	Yes	Materials and Methods
Include a statement about **blinding** even if no blinding was done.	Yes	Materials and Methods
Describe **inclusion/exclusion criteria** if samples or animals were excluded from the analysis. Were the criteria pre-established?<br><br>If sample or data points were omitted from analysis, report if this was due to attrition or intentional exclusion and provide justification.	Yes	Materials and Methods
For every figure, are **statistical tests** justified as appropriate? Do the data meet the assumptions of the tests (e.g., normal distribution)? Describe any methods used to assess it. Is there an estimate of variation within each group of data? Is the variance similar between the groups that are being statistically compared?	Yes	Materials and Methods

Sample definition and in-laboratory replication	Information included in the manuscript?	In which section is the information available? (Reagents and Tools Table, Materials and Methods, Figures, Data Availability Section)
In the figure legends: state number of times the experiment was **replicated** in laboratory.	Yes	Figure legends
In the figure legends: define whether data describe **technical or biological replicates**.	Yes	Figure legends

Ethics

Ethics	Information included in the manuscript?	In which section is the information available? (Reagents and Tools Table, Materials and Methods, Figures, Data Availability Section)
Studies involving **human participants**: State details of **authority granting ethics approval** (IRB or equivalent committee(s), provide reference number for approval.	Not Applicable	
Studies involving **human participants**: Include a statement confirming that **informed consent** was obtained from all subjects and that the experiments conformed to the principles set out in the WMA Declaration of Helsinki and the Department of Health and Human Services Belmont Report.	Not Applicable	
Studies involving **human participants: For publication of patient photos**, include a statement confirming that consent to publish was obtained.	Not Applicable	
Studies involving experimental **animals**: State details of **authority granting ethics approval** (IRB or equivalent committee(s), provide reference number for approval. Include a statement of compliance with ethical regulations.	Yes	Material and Methods
Studies involving **specimen and field samples:** State if relevant **permits** obtained, provide details of authority approving study; if none were required, explain why.	Not Applicable	

Dual Use Research of Concern (DURC)	Information included in the manuscript?	In which section is the information available? (Reagents and Tools Table, Materials and Methods, Figures, Data Availability Section)
Could your study fall under dual use research restrictions? Please check biosecurity documents and list of **select agents and toxins** (CDC): https://www.selectagents.gov/sat/list.htm	Not Applicable	
If you used a select agent, is the security level of the lab appropriate and reported in the manuscript?	Not Applicable	
If a study is subject to dual use research of concern regulations, is the name of the **authority granting approval and reference number** for the regulatory approval provided in the manuscript?	Not Applicable	

Reporting

The MDAR framework recommends adoption of discipline-specific guidelines, established and endorsed through community initiatives. Journals have their own policy about requiring specific guidelines and recommendations to complement MDAR.

Adherence to community standards	Information included in the manuscript?	In which section is the information available? (Reagents and Tools Table, Materials and Methods, Figures, Data Availability Section)
State if relevant guidelines or checklists (e.g., **ICMJE, MIBBI, ARRIVE, PRISMA**) have been followed or provided.	Not Applicable	
For **tumor marker prognostic studies,** we recommend that you follow the **REMARK** reporting guidelines (see link list at top right). See author guidelines, under 'Reporting Guidelines'. Please confirm you have followed these guidelines.	Not Applicable	
For **phase II and III randomized controlled trials**, please refer to the **CONSORT** flow diagram (see link list at top right) and submit the CONSORT checklist (see link list at top right) with your submission. See author guidelines, under 'Reporting Guidelines'. Please confirm you have submitted this list.	Not Applicable	

Data Availability

Data availability	Information included in the manuscript?	In which section is the information available? (Reagents and Tools Table, Materials and Methods, Figures, Data Availability Section)
Have **primary datasets** been deposited according to the journal's guidelines (see 'Data Deposition' section) and the respective accession numbers provided in the Data Availability Section?	Yes	Dataset in graphs, are all available in Table S8.Raw data for all the images used in this work are available in the EBI BioStudies database (https://www.ebi.ac.uk/biostudies/) with
Were **human clinical and genomic datasets** deposited in a public access-controlled repository in accordance to ethical obligations to the patients and to the applicable consent agreement?	Not Applicable	
Are **computational models** that are central and integral to a study available without restrictions in a machine-readable form? Were the relevant accession numbers or links provided?	Not Applicable	
If publicly available data were reused, provide the respective **data citations in the reference list**.	Yes	Main text, figures and reference list
