# Supplementary material for: Senescent Schwann cells induced by aging and chronic denervation impair axonal regeneration following peripheral nerve injury
Source: EMBO Mol Med. 2023 Oct 20;15(12):e17907. doi: 10.15252/emmm.202317907 (PMC10701627; doi:10.15252/emmm.202317907)

Expanded View Fig 5

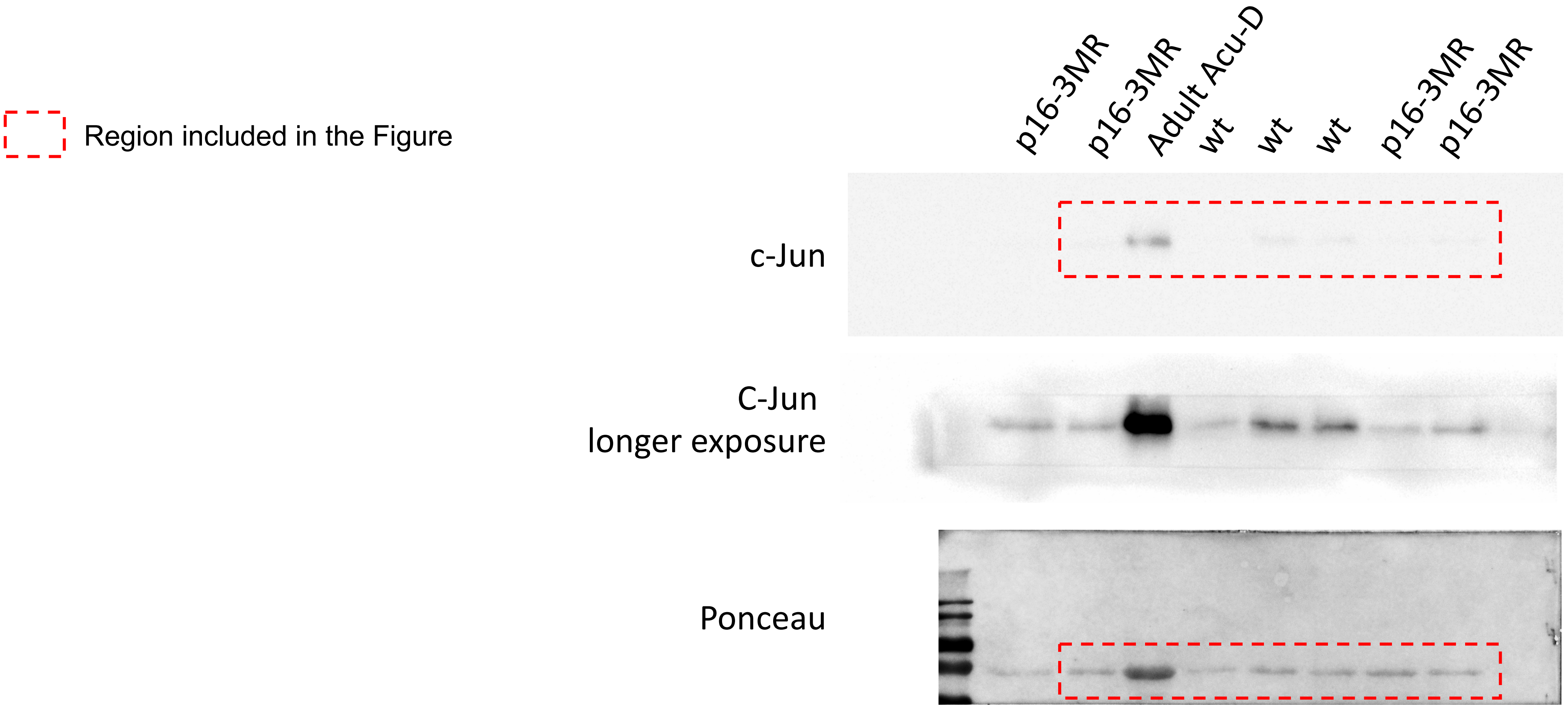

Supplement: Supplementary file 9 — Source Data for Expanded View [file EMMM-15-e17907-s012.zip › SourceData_Fig_EV5.zip!/SourceData_Fig EV5/SourceData_Figure EV5_wb.pdf]
